# The burden and etiologies of diarrhea in Asia and its countries from 1990 to 2021 and the forecast to 2040: analyses informed by the global burden of disease study 2021

**DOI:** 10.3389/fpubh.2025.1651315

**Published:** 2025-08-06

**Authors:** Shan Liu, Zhiping Wu, Qingyu An, Jun Wu, Jinjian Bai, Wei Sun, Linan Guo, Luxi Gong

**Affiliations:** ^1^Emergency Department, Dalian Center for Disease Control and Prevention, Dalian, China; ^2^Department of Experimental Teaching Center of Public Health, School of Public Health, Dalian Medical University, Dalian, China

**Keywords:** diarrhea, GBD, Asia, joinpoint, BAPC

## Abstract

**Background:**

This study aimed to analyze the burden, temporal trends and etiologies of diarrhea from 1990 to 2021, and to forecast the burden from 2022 to 2040 in Asia and Asian countries.

**Methods:**

Data were sourced from the Global Burden of Diseases (GBD) 2021 study. Temporal trends from 1990 to 2021 were analyzed using estimated annual percentage change (EAPC) values. Spearman’s rank test was performed to evaluate the association between diarrhea burdens and socio-demographic index (SDI). Joinpoint analysis was applied to estimate the trends of age-standardized rates (ASRs) from 1990 to 2021. To forecast the burden for diarrhea from 2022 to 2040, we used Bayesian Age-Period-Cohort (BAPC) model. Furthermore, we generated heatmaps to visualize the 13 etiologies of diarrhea.

**Results:**

Overall burden showed that the ASRs in Asia decreased from 1990 to 2021, with EAPCs of −0.40, −0.55, −5.70, and −5.47, respectively. The ASRs demonstrated statistically significant negative correlations with SDI in 2021. The incidence and prevalence rates in 0–9 years old and 90+ years old and the DALY rates and mortality rates in all age groups in 2021 were decreased compared with 1990. Temporal joinpoint analysis revealed that after 2019, the age-standardized incidence rate (ASIR) of diarrhea exhibited a significant upward trend in Asia and some Asian countries. Based on the BAPC model analysis, the ASIR and the age-standardized prevalence rate of diarrhea in Asia are predicted to decrease initially and then increase. The age-standardized DALY rate (ASDR) and the age-standardized mortality rate (ASMR) of diarrhea in Asia are predicted to demonstrate a consistent declining trend. Rotavirus showed the highest ASDRs in both 1990 and 2021 among 13 diarrheal etiologies, while norovirus replaced rotavirus as the leading cause of ASMR by 2021.

**Conclusion:**

The study demonstrates an overall declining trend in the burden of diarrhea in Asia due to urbanization, economic growth, and public health interventions. However, significant challenges persist in some countries and specific population groups. Socioeconomic status exerts a substantial influence on disease burden, highlighting the urgent need for enhanced healthcare resource allocation in some countries.

## 1 Introduction

Diarrhea is defined as having three or more watery/loose stools within 24 h or or increased bowel movement frequency beyond one’s normal pattern. It is usually a symptom of gastrointestinal infection, which can be caused by a variety of bacterial, viral and parasitic organisms (1). These pathogens cause diarrhea symptoms in the body by utilizing mechanisms such as attachment, invasion, intracellular motility, toxin secretion, and interference with host cell functions (2, 3). For most patients, severe dehydration and fluid loss were the main causes of dying from diarrhea (4). Based on fundamental pathological and physiological changes, World Health Organization (WHO) classified diarrhea into acute watery diarrhea (such as cholera), acute bloody diarrhea (such as dysentery) and persistent diarrhea, also known as chronic diarrhea (5).

In 2021, diarrhea accounted for 4.67 billion (95% UI: 4.11–5.22 billion) cases globally. Diarrheal diseases, together with upper respiratory infections and oral disorders, comprised the Level 3 causes with the highest global incidence in 2021. These three causes also had the highest incidence globally for all sexes combined since 2010 (6). However, the mortality burden of diarrhea has shown a substantial decline over the past three decades, with its ranking among global leading causes of death dropping from fifth in 1990 to fourteenth in 2021 (7). This transition reflects significant advancements in global diarrhea treatment and case management strategies. Evidence demonstrates that the burden of diarrhea falls disproportionately on regions lacking proper healthcare, clean water, and sanitation, particularly affecting low-income and marginalized groups (8).

In the Asian continent, the burden of diarrhea is alarmingly high, skewed toward South and Southeast Asian countries (9). From 1990 to 2021, Asia has undergone rapid urbanization, economic growth, and public health interventions, which may have influenced the epidemiology of diarrhea. However, disparities persist, with some regions experiencing declining trends due to improved water, sanitation, and hygiene (WASH) programs, while others still face high transmission rates due to persistent risk factors (10). In addition, climatic factors such as rising temperatures (11), rising amount of rainfall (12), moderate/strong El Nino events (13), as well as social factors such as increased population density (14) can all lead to an increase in the incidence of diarrhea. With the intensification of global warming, the frequency, intensity, and duration of heatwaves in Asia have increased significantly. Countries such as India, Pakistan, China, and Japan have experienced a rise in extreme high-temperature weather events (15–17). Additionally, the population of central and southern Asia has shown a marked increase, rising from 1,297,934 thousand in 1995 to 2,020,052 thousand in 2024 (18). In South Asia, more than 2,000 children die from diarrhea daily, a mortality burden surpassing the combined deaths from Human Immunodeficiency Virus/Acquired Immune Deficiency Syndrome, malaria, and measles in pediatric populations (19).

Previous studies have reported the disease burden and temporal trends of diarrhea in global regions or individual countries, but no comprehensive analysis has been conducted targeting the entirety of Asia and all Asian countries (8, 20, 21). This study was based on data from the global Burden of Diseases, Injuries, and Risk Factors Study (GBD) 2021. In this study, we summarized the data and trends of incidence, prevalence, disability-adjusted life years (DALY) and mortality caused by diarrhea from 1990 to 2021 in Asia and Asian countries. We computed the estimated annual percentage change (EAPC) and performed joinpoint regression analysis from 1990 to 2021. Additionally, we forecast the disease burden of diarrhea disease in the next 19 years (2022–2040) based on bayesian age-period-cohort (BAPC) model. This study aimed to conduct a comprehensive investigation and analysis of the burdens of diarrhea, with the goal of proposing more targeted national public health recommendations.

## 2 Materials and methods

### 2.1 Data sources

The data used in this study were all sourced from the GBD 2021 database. This database uses standardized methods to assess the disease burden of 288 causes of death, 371 diseases and injuries and 88 risk factors in 204 countries and territories, including subnational estimates for 21 countries and territories. Detailed information on data sources, estimation methods, and other relevant information has been elaborately published in previous GBD research series studies (1, 22, 23). Briefly, extensive representative population data were utilized to estimate disease incidence and prevalence rates. These data were derived from published registration and cohort study reports, administrative health data and reports, as well as population surveys, etc.

For this study, we selected annual values and rates of incidence, prevalence, DALY, mortality of diarrhea in Asia and all 48 Asian countries from 1990 to 2021 from the Global Health Data Exchange website.^1^ This research relies on an open-access database and does not require ethical approval. We reported the age—standardized rates (ASRs) of incidence, prevalence, DALY, and mortality of diarrhea in Asia and Asian countries from 1990 to 2021. Besides, the socio-demographic index (SDI) data was collected for individual Asian countries to analyze the association between SDI and ASRs of diarrhea (24). Additionally, we obtained age-stratified data on incidence, prevalence, DALY and mortality rates across consecutive 5-year age groups (from <5 to 95+ years) to compare the burden of diarrhea between 1990 and 2021. Furthermore, we acquired GBD world population age-standard data, Asian population data (including country-specific estimates), and Population Forecasts (2017–2100) data to BAPC models for predicting diarrhea burdens from 2022 to 2040. Finally, this study identified 13 etiologies contributing to the DALY and mortality of diarrhea to analyze the disease burden across different etiologies.

### 2.2 Case definition

In GBD 2021, diarrhea was classified as part of infectious diseases, mainly covering cases of acute diarrhea caused by pathogens such as *Rotavirus*, *Norovirus* and *Vibrio cholerae* (25). GBD 2021 uses the International statistical classification of diseases and related health problems 10th revision (ICD - 10) to classify diarrhea and mainly covers the following categories: A00 (Cholera), A01 (Typhoid and paratyphoid fevers), A02 (Other Salmonella infections), A03 (Shigellosis), A04 (Other bacterial intestinal infections), A05 (Other bacterial foodborne intoxications, not elsewhere classified), A06 (Amoebiasis), A07 (Other protozoal intestinal diseases), A08 (Viral and other specified intestinal infections), A09 (Other gastroenteritis and colitis of infectious and unspecified origin) (26).

### 2.3 Data analysis

The study firstly presented the number and ASRs of incidence, prevalence, DALY and mortality of diarrhea in 1990 and 2021. Additionally, temporal trends from 1990 to 2021 were analyzed by calculating EAPC through a log-linear regression model, expressed mathematically as:

ln(rate)=α+β×year+ε

In our analysis, rate represents either the age-standardized incidence rate (ASIR), prevalence rate (ASPR), disability-adjusted life year rate (ASDR), and mortality rate (ASMR). EAPC value was calculated through the transformation (27):

$\text{EAPC}=100\times (e^{\beta }-1)$

with 95% confidence intervals (CI) obtained from the linear regression model. Besides, spearman’s rank test was performed to evaluate the association between ASRs and SDI in 2021.

Joinpoint regression is a statistical methodology designed to analyze temporal trend variations in time-series data. It identifies one or more inflection points within the dataset, partitioning the time series into distinct segments and fitting linear or log-linear models to each phase. This model can flexibly identify multiple trend change points in the data, and precisely provide the slope of each trend segment and the confidence interval of the change point. Moreover, it can be visually visualized to make the trend changes clear at a glance (28, 29). Considering that diarrhea does not have annual cyclical variations and this study aims to explore the temporal trend of diarrhea from 1990 to 2021, the joinpoint model was selected for trend analysis. Joinpoint regression analysis applies segmented log-linear modeling, expressed as ln(y) = β × x + constant, to identify significant trend change points in temporal patterns. The final model calculated the annual percentage change (APC) and average annual percentage change (AAPC). The APC was calculated as:

$\mathrm{APC}=(e^{\beta }-1)\times 100\%$

where β is the slope coefficient in the logarithmic linear regression model:

ln(y)=β×x+constant

with y representing the observed value and x representing time. The AAPC summarized the overall trend by weighting each segment’s APC according to its duration (30, 31). The model was constructed using Joinpoint software (version 5.1.0).

To predict the ASRs for diarrhea from 2022 to 2040, we used BAPC model based on BAPC and integrated nested laplace approximation packages in the R software. The BAPC model was constructed based on the traditional generalized linear model within the bayesian framework, and it can dynamically integrate age, period, and cohort effects. This enables the model to more comprehensively capture the complex dynamics of disease burden, which may not be possible with traditional statistical methods. In addition, by accounting for multidimensional time effects, the BAPC model can more accurately predict future trends in the burden of disease. This is crucial for public health decision-making, as it can help policymakers develop more effective interventions (32, 33). Considering that diarrhea does not have annual cyclical variations and is highly influenced by demographic factors, coupled with the long-term prediction requirements of this study, the BAPC model was selected over conventional time-series forecasting approaches (such as ARIMA and Holt-Winters).

Furthermore, we generated heatmaps to visualize the distribution of 13 diarrhea-associated etiologies in Asia and Asian countries, in order to compare their relative rankings in contributing to DALY and mortality between 1990 and 2021.

All statistical computations and data processing were performed using R software (version 4.2.1), except for joinpoint analysis. A *p*-value of <0.05 was considered statistically significant.

## 3 Results

### 3.1 Overall burden of diarrhea

#### 3.1.1 Overall burden of diarrhea in Asia

**Figure 1:**
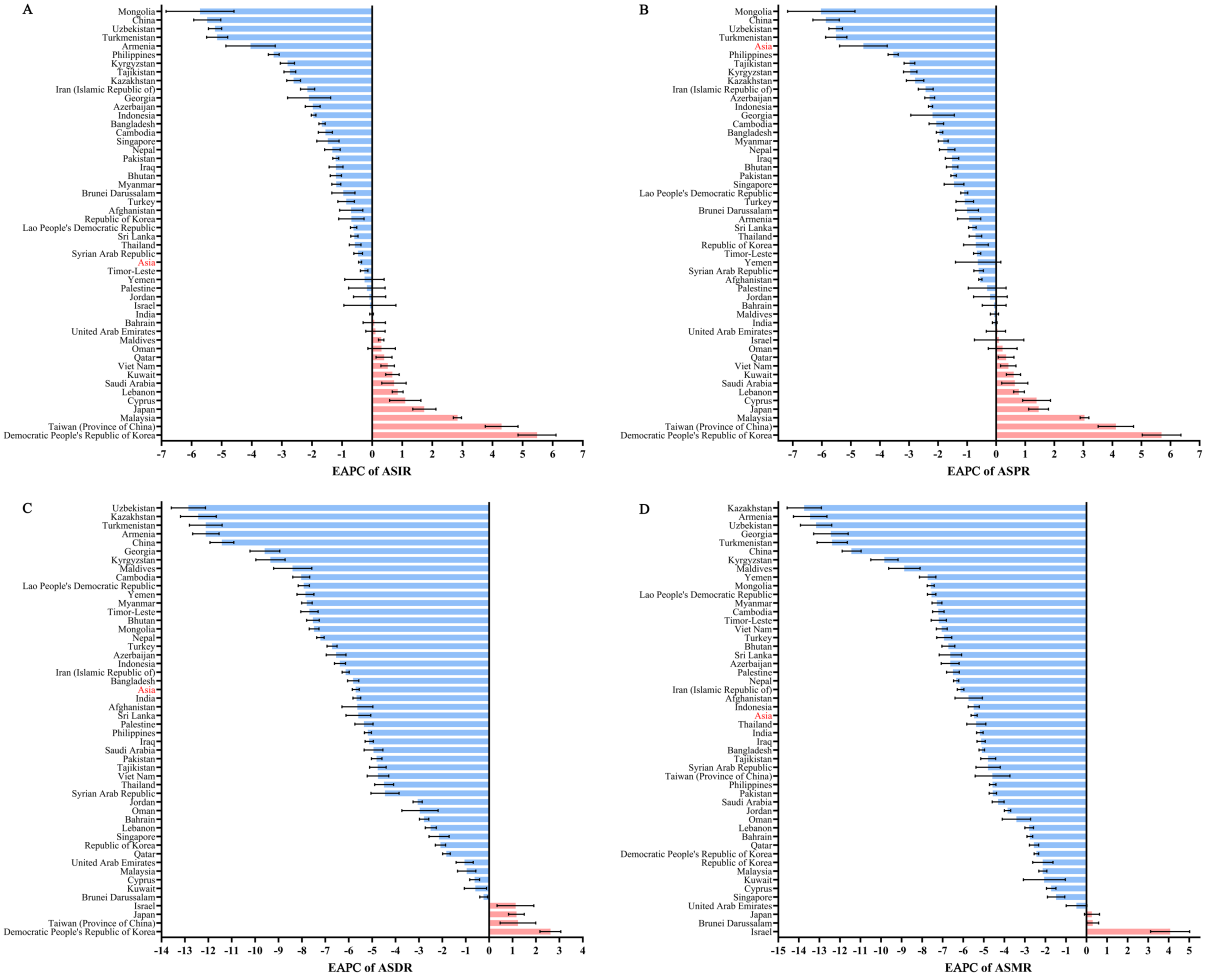
EAPC of ASRs for diarrhea in Asia and Asian countries from 1990 to 2021. **(A)** EAPC of ASIR for diarrhea; **(B)** EAPC of ASPR for diarrhea; **(C)** EAPC of ASDR for diarrhea; **(D)** EAPC of ASMR for diarrhea. EAPC, estimated annual percentage change; ASRs, age-standardized rates; ASIR, age-standardized incidence rate; ASPR, age-standardized prevalence rate; ASDR, age-standardized DALYs rate; ASMR, age-standardized mortality rate.

From 1990 to 2021, the number of incident and prevalent cases of diarrhea in Asia increased, while the age-standardized incidence rate (ASIR) and the age-standardized prevalence rate (ASPR) showed a downward trend. In addition, from 1990 to 2021, the number of DALY and death of diarrhea in Asia decreased, as well as the age-standardized disability-adjusted life year rate (ASDR) and the age-standardized mortality rate (ASMR) showed a downward trend. For the incidence of diarrhea, there were 2,822,383,440.00 cases in 2021 (95%UI: 2,508,634,651.00 to 3,153,683,173.00), and the ASIR decreased from 71,237.70 in 1990 to 63,104.52 in 2021 (EAPC = −0.40 [95%CI: −0.45 to −0.35]). For the prevalence of diarrhea, there were 43009539.24 cases in 2021 (95%UI: 38,968,233.67 to 47,490,424.90), and the ASPR decreased from 1,123.53 in 1990 to 960.71 in 2021 (EAPC = −0.55 [95%CI: −0.60 to −0.49]). For the DALYs of diarrhea, there were 25,276,357.42 cases in 2021 (95%UI: 19,419,622.49 to 35,042,574.14), and the ASDR decreased from 3,642.92 in 1990 to 614.49 in 2021 (EAPC = −5.70 [95%CI: −5.85 to −5.55]). For the mortality of diarrhea, there were 669,760.85 cases in 2021 (95%UI: 432,680.63 to 1,025,709.00), and the ASMR decreased from 86.91 in 1990 to 15.53 in 2021 (EAPC = −5.47 [95%CI: −5.62 to −5.32]). In different Asian regions, the highest ASIR (121,489.61 [95% UI: 108,598.96 to 134,981.39]), ASPR (1,857.37 [95% UI: 1,693.29 to 2,029.27]), ASDR (1,351.35 [95% UI: 981.62 to 1,948.74]), and ASMR (47.72 [95% UI: 30.52 to 75.95]) were all in South Asia in 2021. From 1990 to 2021, the ASIR (EAPC = 1.49 [95%CI: 1.11 to 1.86]), ASPR (EAPC = 1.23 [95%CI: 0.91 to 1.56]), and ASDR (EAPC = 0.57 [95%CI: 0.28 to 0.85]) in the High-income Asia Pacific region increased, while they decreased in all other regions (Figure 1; Table 1).

**Table 1:** Analysis of burdens of diarrhea in Asia and its regions from 1990 to 2021.

Regions	1990		2021		1990–2021
	Cases No. (95%UI)	Age-standardized rate per 100,000 No. (95%UI)	Cases No. (95%UI)	Age-standardized rate per 100,000 No. (95%UI)	EAPC No. (95%CI)
Incidence					
Asia	2,402,499,924.00 (2,056,038,539.00 to 2,737,005,360.00)	71,237.70 (61,659.66 to 80,907.39)	2,822,383,440.00 (2,508,634,651.00 to 3,153,683,173.00)	63,104.52 (55,880.78 to 70,754.31)	−0.40 (−0.45 to −0.35)
Central Asia	22,126,148.11 (19,230,931.09 to 25,015,467.66)	25,482.00 (22,210.82 to 28,897.23)	8,499,446.97 (7,101,928.02 to 9,993,822.96)	8,828.98 (7,338.64 to 10,393.63)	−3.35 (−3.49 to −3.22)
East Asia	297,583,014.53 (246,135,688.32 to 352,413,811.04)	25,263.17 (20,941.29 to 29,838.11)	101,160,862.80 (85,502,657.48 to 118,327,464.80)	7,703.50 (6,377.99 to 9,167.49)	−4.40 (−4.73 to −4.06)
South Asia	1,529,238,534.61 (1,299,660,182.78 to 1,737,502,789.85)	128,123.11 (112,430.41 to 144,337.51)	2,209.192,323.00 (1,965,020,024.00 to 2,469,561,679.00)	121,489.61 (108,598.96 to 134,981.39)	−0.23 (−0.29 to −0.18)
Southeast Asia	420,445,471.03 (360,117,688.99 to 481,486,288.12)	82,572.88 (72,303.90 to 93,680.92)	357,634,673.20 (313,060,456.60 to 401,985,422.60)	54,344.12 (47,487.96 to 61,100.31)	−1.40 (−1.46 to −1.33)
High-income Asia Pacific	41,652,276.72 (33,867,891.25 to 49,620,447.94)	27,758.11 (22,376.07 to 33,584.45)	53,655,850.25 (46,067,352.25 to 61,882,344.01)	37,567.37 (30,698.20 to 44,975.26)	1.49 (1.11 to 1.86)
Prevalence					
Asia	37,904,069.2 (32,982,586.18 to 43,144,289.5)	1,123.53 (990.28 to 1,266.98)	43,009,539.24 (38,968,233.67 to 47,490,424.90)	960.71 (867.61 to 1,063.68)	−0.55 (−0.60 to −0.49)
Central Asia	371,220.62 (336,361.14 to 410,645.23)	426.91 (386.50 to 473.53)	136,131.04 (115,820.55 to 158,840.14)	141.67 (120.09 to 165.61)	−3.56 (−3.66 to −3.45)
East Asia	4,877,123.06 (4,134,186.64 to 5,793,429.48)	413.68 (349.66 to 491.17)	1,517,726.82 (1,288,107.33 to 1,766,142.63)	116.21 (97.24 to 137.05)	−4.68 (−5.01 to −4.35)
South Asia	23,514,359.98 (20,532,225.50 to 26,842,145.17)	1,971.36 (1,770.28 to 2,195.22)	33,781,280.29 (30,657,717.76 to 37,097,378.12)	1,857.37 (1,693.29 to 2,029.27)	−0.30 (−0.37 to −0.23)
Southeast Asia	6,973,072.48 (6,087,393.53 to 7,921,960.13)	1,369.55 (1,218.49 to 1,531.94)	5,388,198.44 (4,888,193.03 to 6,011,728.05)	820.46 (737.99 to 918.79)	−1.69 (−1.75 to −1.62)
High-income Asia Pacific	696,530.47 (568,241.44 to 836,180.02)	458.84 (373.48 to 556.14)	829,328.76 (728,317.78 to 942,393.69)	585.85 (486.33 to 692.83)	1.23 (0.91 to 1.56)
DALY					
Asia	115,630,578.10 (94,035,364.55 to 139,271,832.70)	3,642.92 (2,971.09 to 4,536.20)	25,276,357.42 (19,419,622.49 to 35,042,574.14)	614.49 (479.7 to 825.94)	−5.70 (−5.85 to −5.55)
Central Asia	1,292,224.97 (1,167,496.84 to 1,437,578.71)	1,384.22 (1,252.12 to 1,538.59)	178,319.26 (134,196.89 to 236,318.40)	180.12 (135.68 to 238.49)	−7.63 (−8.01 to −7.24)
East Asia	7,713,544.72 (5,966,377.23 to 9,467,219.89)	681.69 (526.20 to 838.12)	329,624.03 (263,967.37 to 426,510.14)	29.90 (24.17 to 36.99)	−10.90 (−11.40 to −10.40)
South Asia	83,347,993.98 (68,177,644.06 to 101,366,583.50)	7,630.49 (6,111.88 to 9,999.63)	20,403,658.98 (15,058,272.99 to 29,118,599.87)	1,351.35 (981.62 to 1,948.74)	−5.51 (−5.67 to −5.36)
Southeast Asia	20,268,906.22 (14,324,898.07 to 26,429,937.96)	4,208.73 (2,932.65 to 5,788.35)	3,278,818.79 (2,442,886.30 to 4,121,222.61)	551.20 (409.23 to 684.77)	−6.40 (−6.58 to −6.21)
High-income Asia Pacific	121,485.62 (95,073.64 to 154,933.92)	83.70 (64.81 to 107.43)	156,794.45 (126,356.27 to 197,862.08)	85.57 (62.77 to 115.51)	0.57 (0.28 to 0.85)
Mortality					
Asia	1,944,051.00 (1,510,471.38 to 2,598,657.28)	86.91 (64.30 to 120.61)	669,760.85 (432,680.63 to 1,025,709.00)	15.53 (10.30 to 23.3)	−5.47 (−5.62 to −5.32)
Central Asia	14,265.15 (12,909.13 to 15,884.46)	15.52 (14.08 to 17.26)	1,912.68 (1,412.35 to 2,560.64)	1.95 (1.45 to 2.60)	−7.82 (−8.22 to −7.41)
East Asia	91,454.62 (67,838.70 to 113,072.60)	9.01 (6.50 to 11.39)	4,500.46 (2,880.10 to 7,644.60)	0.34 (0.24 to 0.51)	−11.31 (−11.76 to −10.85)
South Asia	1,487,203.59 (1,163,614.35 to 1,996,412.11)	230.96 (169.95 to 330.02)	571,732.43 (368,043.02 to 902,337.04)	47.72 (30.52 to 75.95)	−5.05 (−5.21 to −4.89)
Southeast Asia	317,054.16 (211,742.42 to 461,496.81)	98.38 (57.22 to 152.66)	75,513.40 (41,685.69 to 100,810.27)	14.22 (7.59 to 19.25)	−6.03 (−6.23 to −5.83)
High-income Asia Pacific	1,617.76 (1,292.34 to 1,917.47)	1.08 (0.87 to 1.26)	4,822.55 (3,683.86 to 6,008.50)	0.80 (0.64 to 1.00)	−0.31 (−0.59 to −0.02)

DALY, disability-adjusted life years; EAPC, estimated annual percentage change; 95% UI, 95% uncertainty interval; 95% CI, 95% confidence interval.

#### 3.1.2 Overall burden of diarrhea in Asian countries

From the perspective of countries, the highest ASIR (135,663.73 [95% UI: 121,215.14 to 151,088.39]), ASPR (29,167,174.06 [95% UI: 26,514,236.97 to 31,996,718.62]), ASDR (291,671,74.06 [95% UI: 26,514,236.97 to 31,996,718.62]) and ASMR (496,724.97 [95% UI: 303,569.73 to 791,339.64]) were all in India in 2021. From 1990 to 2021, Democratic People’s Republic of Korea experienced the highest EPCA in ASIR (5.48 [95%CI: 4.85 to 6.11]), ASPR (5.69 [95%CI: 5.03 to 6.36]) and ASDR (2.62 [95%CI: 2.17 to 3.06]). However, Israel experienced the highest EPCA (4.07 [95%CI: 3.12 to 5.02]) in the ASMR (Figure 1; Supplementary Table S1).

### 3.2 Correlation between the burden of diarrhea and SDI

**Figure 2:**
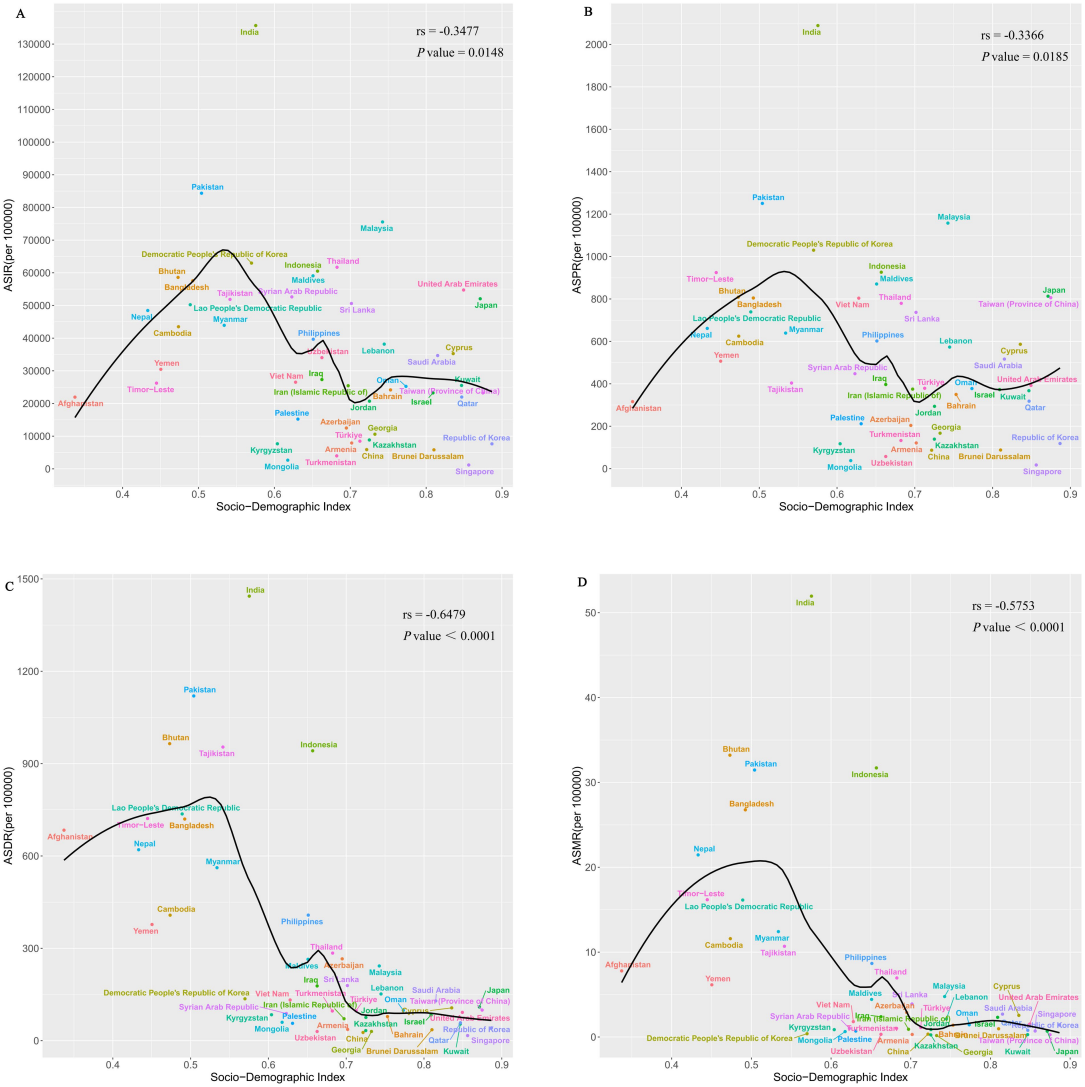
Spearman’s rank tests between ASRs and SDI in Asia in 2021. **(A)** Spearman’s rank tests between ASIR and SDI; **(B)** Spearman’s rank tests between ASPR and SDI; **(C)** Spearman’s rank tests between ASDR and SDI; **(D)** Spearman’s rank tests between ASMR and SDI. ASRs, age-standardized rates; SDI, socio-demographic index; ASIR, age-standardized incidence rate; ASPR, age-standardized prevalence rate; ASDR, age-standardized DALYs rate; ASMR, age-standardized mortality rate.

Figure 2 showed the association between ASRs and SDI. The ASIR, ASPR, ASDR and ASMR were all inversely U-shaped correlated with SDI. Spearman’s rank tests showed significant negative correlations between ASRs and SDI in 2021 (ASIR: rs = −0.3477, *p* = 0.0148; ASPR: rs = −0.3366, *p* = 0.0185; ASDR: rs = −0.6479, *p* < 0.0001; ASMR: rs = −0.5753, *p* < 0.0001), which meant more economically developed countries had lower burdens of diarrhea.

### 3.3 Age diversity and time trends in the burden of diarrhea

#### 3.3.1 Age diversity and time trends in the burden of diarrhea in Asia

**Figure 3:**
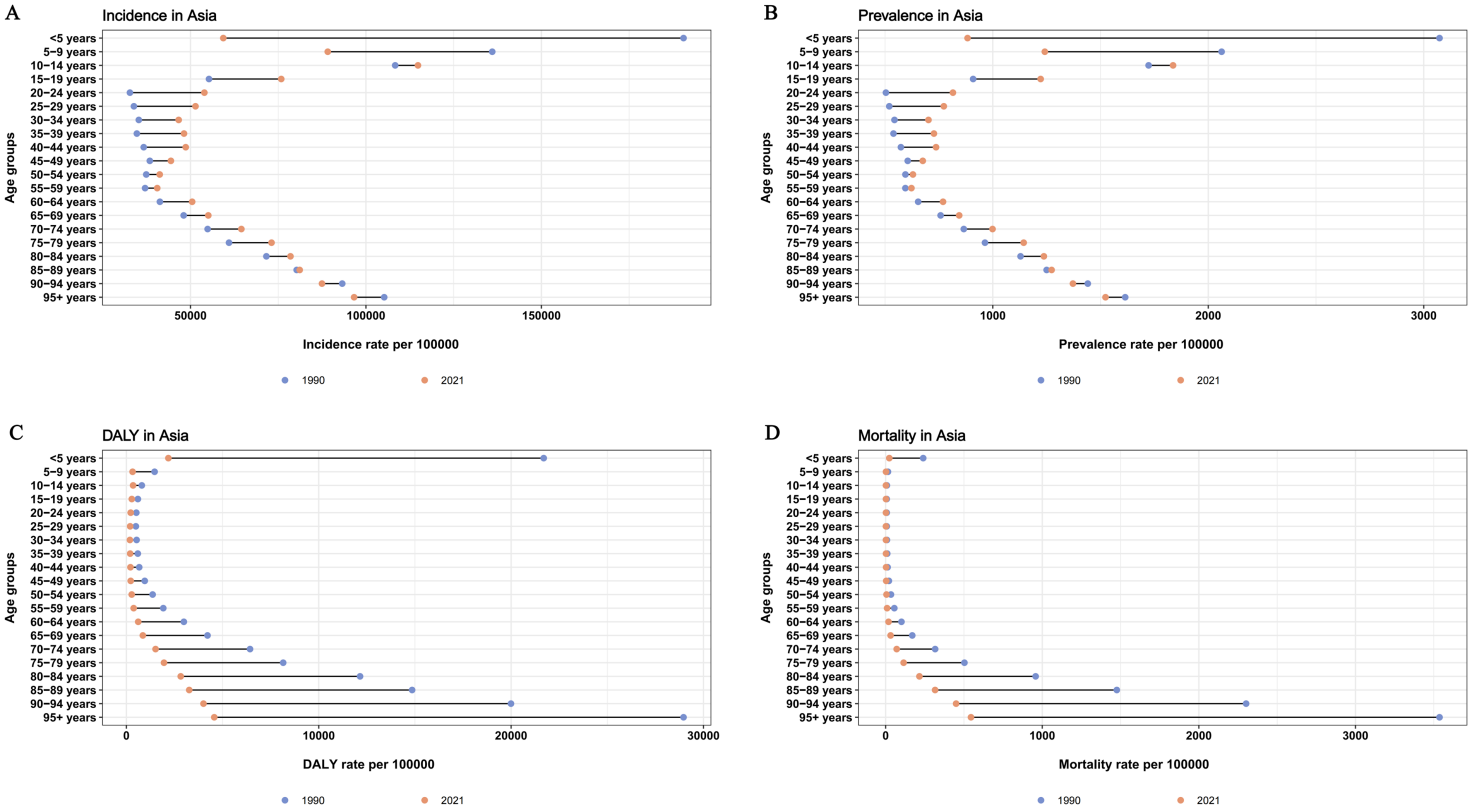
Age diversities of diarrhea burden and time trends in Asia. **(A)** Age diversities of incidence; **(B)** Age diversities of prevalence; **(C)** Age diversities of DALY rate; **(D)** Age diversities of mortality. DALY, disability-adjusted life year.

From 1990 to 2021, the highest incidence and prevalence rates shifted from the <5 years group to the 10–14 years group. In 1990 and 2021, the age group with the highest DALY rate and mortality rate were all the 95+ years group. Compared with 1990, the incidence and prevalence rates in the <5 years, 5–9 years, 90–94 years, and 95+ years age groups all decreased in 2021, while the other age groups showed varying degrees of increase. However, DALY rates and mortality rates in 2021 decreased across all age groups compared to 1990 (Figure 3).

#### 3.3.2 Age diversity and time trends in the burden of diarrhea in Asian countries

In 2021, the incidence and prevalence of diarrhea in <5 years groups decreased in all Asian countries compared to 1990, except in Cyprus, Japan, Malaysia, and Taiwan (Province of China). Besides, the incidence and prevalence of diarrheal diseases in 95 + years age groups increased in all Asian countries compared to 1990, except in Bhutan, Brunei Darussalam, China, Japan, Pakistan, Phillippines, Singapore and Turkmenistan. For DALY and Mortality of diarrhea, rates for all age groups decreased in 2021 compared to 1990, except in Bahrain, Cyprus, Democratic People’s Republic of Korea, Israel, Japan, Kuwait, Lebanon, Malaysia, Syrian Arab Republic, Taiwan, United Arab Emirates (Supplementary Figures S1–S49).

### 3.4 Temporal joinpoint analysis of the burden of diarrhea

#### 3.4.1 Temporal joinpoint analysis of the burden of diarrhea in Asia

**Figure 4:**
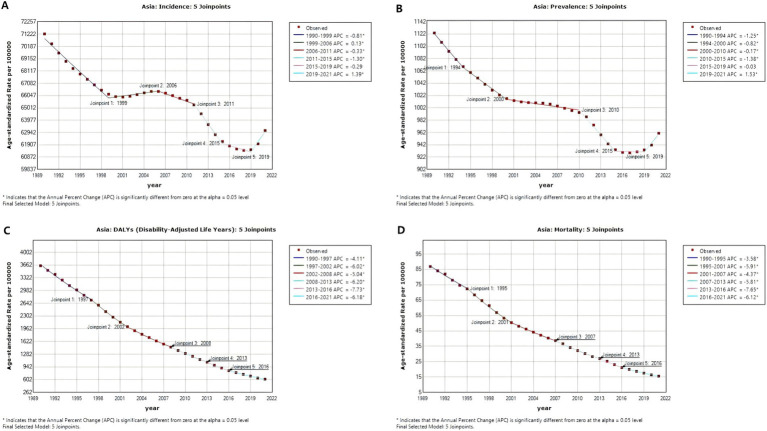
Temporal joinpoint analysis of diarrhea in Asia from 1990 to 2021. **(A)** Temporal joinpoint analysis of ASIR; **(B)** Temporal joinpoint analysis of ASPR; **(C)** Temporal joinpoint analysis of ASDR; **(D)** Temporal joinpoint analysis of ASMR. APC, annual percentage change; ASIR, age-standardized incidence rate; ASPR, age-standardized prevalence rate; ASDR, age-standardized DALYs rate; ASMR, age-standardized mortality rate.

As shown in Figure 4 and Supplementary Table S2, ASIR of diarrhea showed a significant decline from 1990 to 1999 (APC_1990–1999_ = −0.81 [95% CI: −0.87 to −0.75]), followed by a slight increase from 1999 to 2006 (APC_1999–2006_ = 0.13 [95% CI: 0.02 to 0.23]), then continued to decline from 2006 to 2015 (APC_2006–2011_ = −0.33 [95% CI: −0.53 to −0.23]; APC_2011–2015_ = −1.30 [95% CI: −1.60 to −0.99]), stabilized between 2015 and 2019, and exhibited a significant upward trend from 2019 to 2021 (APC_2019–2021_ = 1.39 [95% CI: 0.80 to 1.99]). The ASPR of diarrhea showed a continuous downward trend from 1990 to 2015 (APC_1990–1994_ = −1.25 [95% CI: −1.39 to −1.12]; APC_1994–2000_ = −0.82 [95% CI: −0.91 to −0.73]; APC_2000–2010_ = −0.17 [95% CI: −0.20 to −0.14]; APC_2010–2015_ = −1.38 [95% CI: −1.50 to −0.25]), stabilized between 2015 and 2019, and exhibited a significant upward trend from 2019 to 2021 (APC_2019–2021_ = 1.53 [95% CI: 1.10 to 1.89]). The ASDR and ASMR of diarrhea has decreased continuously in the past 21 years (AAPC_ASDR_ = −5.62 [95% CI: −5.82 to −5.43]; AAPC_ASMR_ = −5.43 [95% CI: −5.65 to −5.20]).

#### 3.4.2 Temporal joinpoint analysis of the burden of diarrhea in Asian countries

As shown in Supplementary Figures S50–S98 and Supplementary Table S2, in contrast to the previous downward trend, ASIR has shown an upward trend after 2019 in Cambodia (APC_2015–2019_ = −1.38; APC_2019–2021_ = 2.29), Jordan (APC_2015–2019_ = −11.32; APC_2019–2021_ = 1.16), Lao People’s Democratic Republic (APC_2016–2019_ = −0.61; APC_2019–2021_ = 0.94), Maldives (APC_2011–2019_ = 0.00; APC_2019–2021_ = 2.53), Pakistan (APC_2012–2019_ = −2.11; APC_2019–2021_ = 0.10), Thailand (APC_2015–2019_ = −0.20; APC_2019–2021_ = 2.59), Turkey (APC_2014–2019_ = −4.92; APC_2019–2021_ = 0.89), United Arab Emirates (APC_2014–2019_ = −4.81; APC_2019–2021_ = 1.77). Although there was a previous upward trend, ASIR has exhibited a more significant increase after 2019 in Bhutan (APC_2015–2019_ = 0.40; APC_2019–2021_ = 2.73), India (APC_2015–2019_ = 0.62; APC_2019–2021_ = 2.72), Malaysia (APC_2014–2019_ = 1.12; APC_2019–2021_ = 2.69), Singapore (APC_2016–2019_ = 1.02; APC_2019–2021_ = 5.61), Sri Lanka (APC_2015–2019_ = 0.51; APC_2019–2021_ = 2.89). In addition, compared to the previous downward trend, the significance of the decline in ASIR has diminished after 2019 in Cyprus (APC_2015–2019_ = −8.02; APC_2019–2021_ = −1.54), Georgia (APC_2015–2019_ = −13.22; APC_2019–2021_ = −5.10), Israel (APC_2014–2019_ = −8.92; APC_2019–2021_ = −2.21), Palestine (APC_2016–2019_ = −14.01; APC_2019–2021_ = −6.74), Tajikistan (APC_2015–2019_ = −5.14; APC_2019–2021_ = −1.03), Uzbekistan (APC_2015–2019_ = −9.35; APC_2019–2021_ = −2.24).

### 3.5 Prediction of the burden of diarrhea from 2022 to 2040

#### 3.5.1 Prediction of the burden of diarrhea in Asia from 2022 to 2040

**Figure 5:**
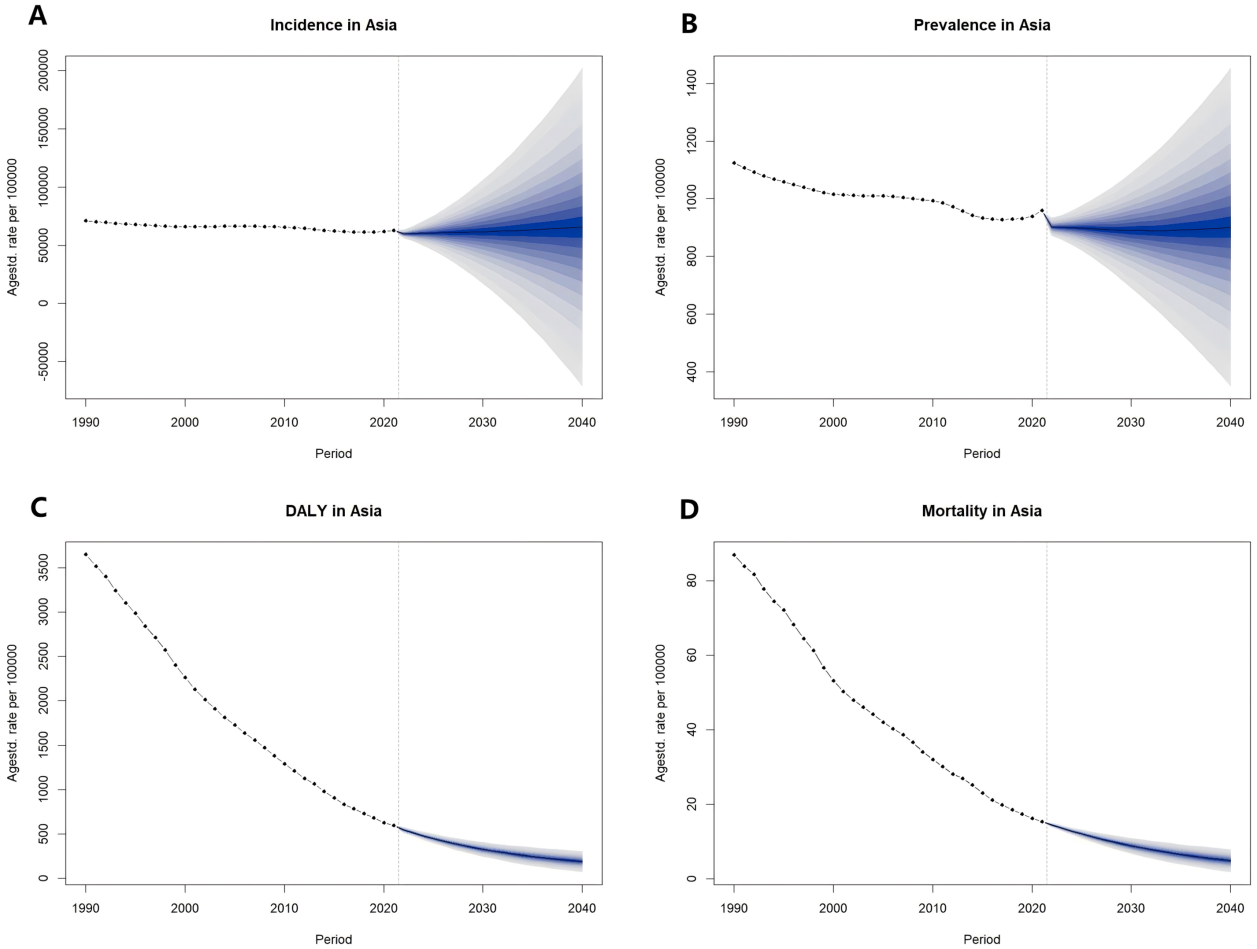
Prediction of diarrhea burden in Asia from 2022 to 2040. **(A)** Prediction of ASIR from 2022 to 2040; **(B)** Prediction of ASPR from 2022 to 2040; **(C)** Prediction of ASDR from 2022 to 2040; **(D)** Prediction of ASMR from 2022 to 2040. DALY, disability-adjusted life year; ASIR, age-standardized incidence rate; ASPR, age-standardized prevalence rate; ASDR, age-standardized DALYs rate; ASMR, age-standardized mortality rate.

Based on the BAPC model analysis, ASIR and ASPR of diarrhea in Asia were predicted to decrease initially and then increase. The ASIR was predicted to decrease to the lowest value (59,232.94 [95%CI: 39,835.16 to 78,630.72]) in 2029 and the ASPR was predicted to decrease to the lowest value (889.70 [95%CI: 608.48 to 1170.93]) in 2033. The ASDR and ASMR of diarrhea in Asia were predicted to demonstrate a consistent declining trend and reach the lowest values in 2040 (ASDR = 190.12 [95%CI: 73.71 to 306.53]; ASMR = 4.84 [95%CI: 1.84 to 7.85]) (Figure 5; Supplementary Tables S3, S4).

#### 3.5.2 Prediction of the burden of diarrhea in Asian countries from 2022 to 2040

The ASIR of diarrhea from 2022 to 2040 was predicted to demonstrate a consistent increasing trend in Bahrain, India, Indonesia, Malaysia, Oman, Qatar, Saudi Arabia, Singapore, Sri Lanka, Taiwan, and Thailand, whereas an initial decline followed by an increasing trend was predicted in Bhutan, Cambodia, Pakistan, and Syrian Arab Republic. In other countries, the ASIR was predicted to demonstrate a consistent declining trend.

The ASPR of diarrhea from 2022 to 2040 was predicted to demonstrate a consistent increasing trend in Bahrain, Bhutan, India, Malaysia, Oman, Qatar, Saudi Arabia, Singapore, Sri Lanka, Taiwan, and Thailand, whereas an initial decline followed by an increasing trend was predicted in Cambodia, Pakistan, Syrian Arab Republic and United Arab Emirates. In other countries, the ASPR was predicted to demonstrate a consistent declining trend.

The ASDR of diarrhea from 2022 to 2040 was predicted to demonstrate an initial decline followed by an increasing trend in Bahrain, Qatar, Saudi Arabia, and Thailand, whereas a consistent declining trend was predicted in other countries.

The ASMR of diarrhea from 2022 to 2040 was predicted to demonstrate a consistent increasing trend in Bahrain and Kuwait, whereas a consistent declining trend was predicted in other countries (Supplementary Figures S99–S147; Supplementary Tables S3, S4).

### 3.6 Etiologies for the cause of the burden of diarrhea in 1990 and 2021

#### 3.6.1 Etiologies for the cause of the burden of diarrhea in Asia in 1990 and 2021

**Figure 6:**
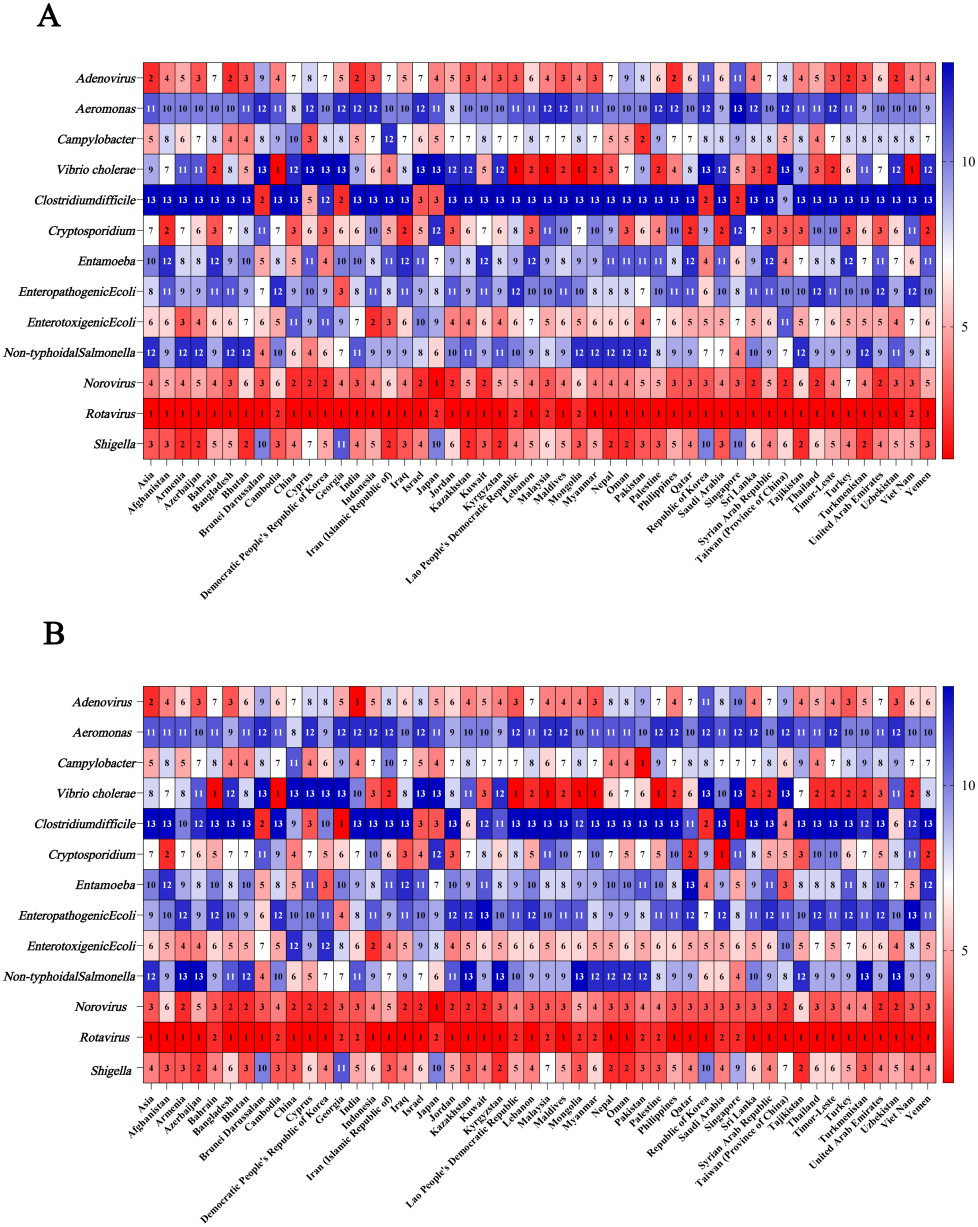
Ranking of etiologies for the cause of DALY of diarrheal disease in Asia and Asian countries in 1990 and 2021. **(A)** Ranking of etiologies for the cause of DALY of diarrheal disease in 1990; **(B)** Ranking of etiologies for the cause of DALY of diarrheal disease in 2021. DALY, disability-adjusted life years.

In 1990 and 2021, the ASDR caused by *Norovirus* was the highest (ASDR_1990_ = 911.86 [95% UI: 702.56 to 1,150.42]; ASDR_2021_ = 100.47 [95% UI: 75.28 to 139.55]), followed by *Adenovirus* (ASDR_1990_ = 590.61 [95% UI: 360.35 to 906.92]; ASDR_2021_ = 92.25 [95% UI: 55.13 to 144.00]) (Figure 6; Supplementary Table S5).

**Figure 7:**
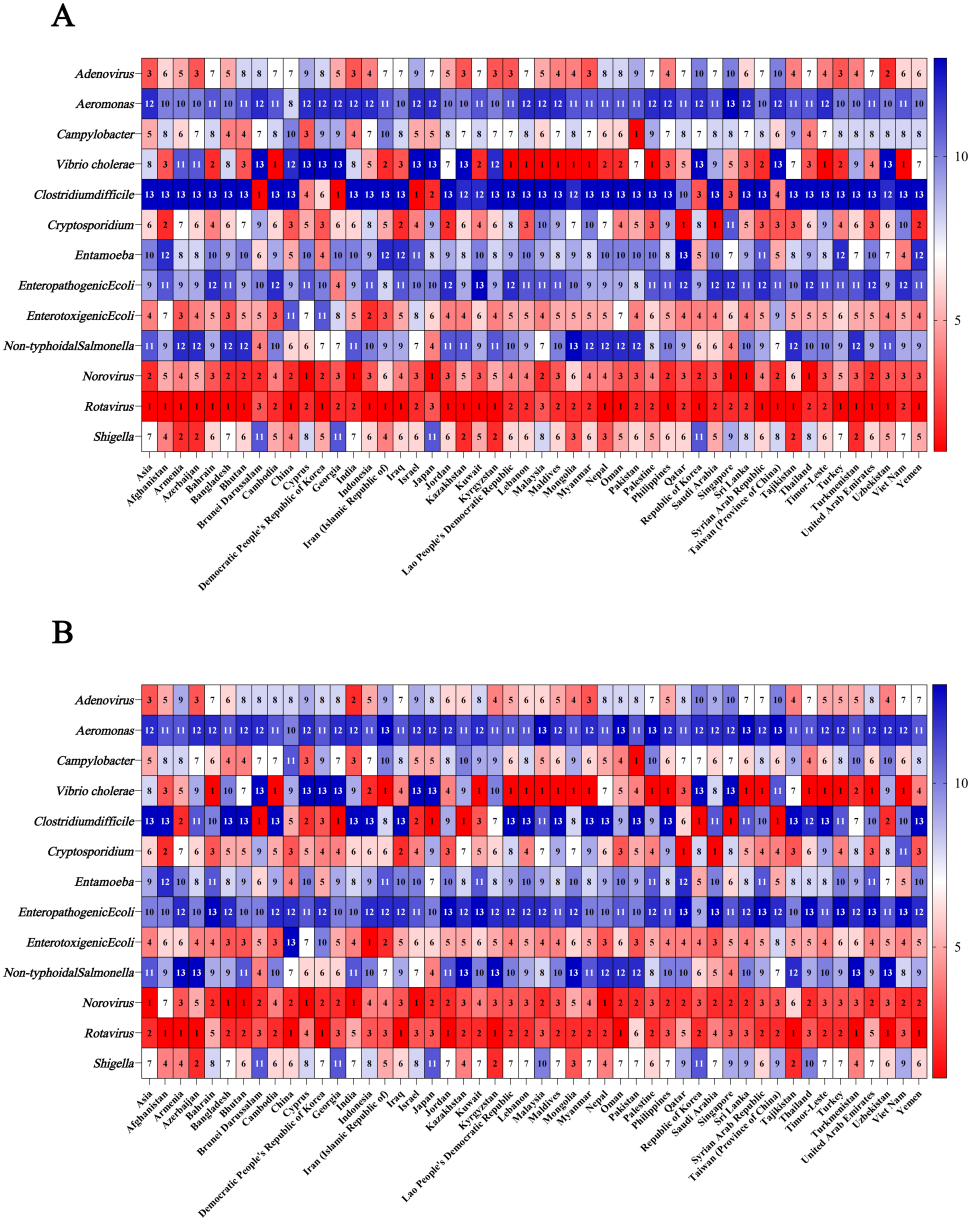
Ranking of etiologies for the cause of mortality of diarrheal disease in Asia and Asian countries in 1990 and 2021. **(A)** Ranking of etiologies for the cause of mortality in 1990; **(B)** Ranking of etiologies for the cause of mortality in 2021.

Among the 13 etiologies of diarrhea in 1990, the ASMR caused by *Rotavirus* was the highest (13.01 [95% UI: 10.06 to 16.73]), followed by *Norovirus* (9.17 [95% UI: 1.93 to 16.52]). In 2021, the ASMR caused by *Norovirus* was the highest (1.69 [95% UI: 0.30 to 3.25]), followed by *Rotavirus* (1.55 [95% UI: 1.12 to 2.25]) (Figure 7; Supplementary Table S5).

#### 3.6.2 Etiologies for the cause of the burden of diarrhea in Asian countries in 1990 and 2021

Among the 13 etiologies of diarrhea in 1990 and 2021, *Rotavirus* was the etiology that ranked among the top 2 causes of ASDR in all Asian countries. Besides, *Rorovirus* was the etiology that ranked among the top 6 causes of ASDR in all Asian countries. In addition, in Bahrain, Cambodia, Lao People’s Democratic Republic, Lebanon, Malaysia, Maldives, Mongolia, Myanmar, Palestine, Sri Lanka, Syrian Arab Republic, Thailand, Timor-Leste, and Viet Nam, *Vibrio cholerae* was the etiology that ranked among the top 2 causes of ASDR in both 1990 and 2021 (Figure 6; Supplementary Table S5).

As for ASMR in 1990, *Rotavirus* was the etiology that ranked among the top 2 causes and *Norovirus* was still the etiology that ranked among the top 6 causes in all countries. In addition, in Afghanistan, Bahrain, Cambodia, Iran (Islamic Republic of), Kuwait, Lao People’s Democratic Republic, Lebanon, Malaysia, Maldives, Mongolia, Myanmar, Palestine, Philippines, Sri Lanka, Syrian Arab Republic, Thailand, Timor-Leste, Turkey, and Viet Nam, *Vibrio cholerae* was the etiology that ranked among the top 3 causes of ASMR in both 1990 and 2021 (Figure 7; Supplementary Table S5).

## 4 Discussion

This study systematically analyzed the overall and age-specific incidence, prevalence, DALY, and mortality rates of diarrhea, examined their long-term trends and intercountry variations across Asian nations, and forecasted the disease burden from 2022 to 2040 for Asia and individual Asian countries. We also assessed different etiologies of diarrhea in Asia and Asian countries.

The ASRs of diarrhea in Asia were all decreased from 1990 to 2021. This is inseparable from a series of comprehensive measures taken by the WHO against diarrhea. In this regard, WHO supports countries in improving their regulatory systems for diarrhea prevention and control by issuing health guidance documents, best practice guidelines, norms, and standards. The key areas include drinking water safety, sanitation facility safety (such as toilets and sewage treatment), recreational water quality (34). WASH interventions have also achieved significant results. Previous study has shown that WASH interventions reduced the risk of diarrhea among children in low- and middle-income countries. Sanitation interventions can reduce diarrhea risk by 24% (35). In addition, Vaccine promotion is also a major reason for the decrease in disease burden. In 2009, WHO recommended the inclusion of rotavirus vaccines in all national immunization programs (36). However, the number of incident and prevalent cases of diarrhea in Asia continued to increase from 1990 to 2021. This may be attributed to the increase of population. According to data from the United Nations Population Division, the total population of Asia in 2021 amounted to 4,704,826.92 thousand, a 48% increase from 1990 (3,179,270.79 thousand) (37). Population growth generally has a more direct impact on the number of diarrhea cases.

There were some South Asia and Southeast Asia countries still need to concentrate on the control of diarrhea, such as India, Pakistan and Malaysia. Primarily, Mamta D. Sharma et al. suggested that open defecation, poor toilet conditions, and inadequate hand hygiene as primary contributors to the increased incidence of diarrhea (38). According to 2017 data, approximately 51% of India’s population practiced open defecation, while only 31% had access to improved sanitation facilities (39). Subsequently, Aatishya Mohanty et al. posit that the differences between collectivism (characterized by values such as obedience, tradition, interdependence, cooperation and collective action) and individualism are also one of the factors contributing to regional variations in diarrhea incidence rates. After controlling for multiple confounding factors including demographic characteristics, health status, poverty levels, and historical indicators, the findings demonstrate that higher collectivism is associated with improved WASH practices, ultimately leading to reduced the prevalence of diarrhea (40). Moreover, located in South Asia, India has a tropical monsoon climate characterized by year-round high temperatures, distinct wet and dry seasons, and abundant rainfall during monsoon periods. High temperatures can accelerate bacterial proliferation in drinking water and animal hosts, promote food spoilage, and foster the growth of diarrheal pathogens (41). Heavy rainfall and flooding can disseminate bacterial pathogens and contaminate unprotected drinking water sources, thereby increasing population exposure to contaminated water (42). The alternating monsoon climate (rainy and dry seasons) can also indirectly increase the burden of diarrhea by affecting food security, leading to shortages or contamination of drinking water, affecting the gross domestic product, and limiting public health inputs (43, 44).

In our study, we also found a statistically significant negative association between SDI and ASRs, indicating that countries with low-middle socioeconomic development tend to exhibit a higher burden of diarrhea. This result was consistent with the findings of Chen et al. (45). Analyzing the underlying reasons, the issue of safe drinking water in underdeveloped countries and regions has not been well resolved, and unsafe WASH is a major risk factor for diarrhea (45). Secondly, malnutrition (stunted growth, emaciation, and underweight) is a key risk factor for diarrhea in children under 5 years old (46), and children in low-income countries have significantly higher rates of low birth weight and malnutrition than those in high-income countries (47), making them more susceptible to diarrhea caused by pathogens. Thirdly, most countries with low SDI are located in South Asia and Southeast Asia, a region dominated by tropical monsoon/rainforest climates. The high temperatures and extreme rainfall events caused by these climates can lead to an increase in cases of diarrhea (48, 49). Furthermore, in South and Southeast Asia, cultural factors such as multiple religions and pilgrimage activities are prevalent. Due to limited food hygiene conditions during religious rituals and the heightened risk of pathogen transmission from mass gatherings, these practices are prone to trigger diarrheal diseases and foodborne illnesses (50, 51).

From the perspective of the age diversity of the diarrhea disease burden in Asia, the threat of diarrhea disease to children under 5 years old has decreased. However, it still has a relatively significant impact on the health of the older adults population over 90 years old. Troeger et al. discovered through a global multi - regional study that although the disease burden of diarrhea has been decreasing year by year, it remains a major health threat to children under 5 years old and the older adults over 70 years old (especially those over 90 years old) (8). A study on the burden of diarrhea in the Eastern Mediterranean region from 1990 to 2015 also demonstrated that among the 17 countries in this region, the mortality rate was higher in the 70 + age group than the under-5 age group (52). These were all consistent with the findings of our study. The older adults may face a higher risk of diarrhea due to immunosenescence and comorbidities (53). Therefore, special attention may be required during the treatment process. Our study also indicated that the incidence and prevalence of diarrhea have increased in the population aged 10–89 years, particularly in the 10–14-year—old age group. One key reason is that children in this age group are of school age, and their collective living environment, primarily at school, may increase their exposure to contaminated water sources or cross-infection risks, particularly in areas with poor infrastructure (54). Another contributing reason is that some low- and middle-income countries, children of this age may begin to take on household chores such as fetching water, cleaning kitchen utensils and cooking, thereby increasing their exposure to untreated water sources (55). It is also important to note that climatic factors may also contribute to the increased incidence and prevalence of diarrhea among adolescents aged 10–14. Drought conditions reduce water availability, potentially forcing affected households to rely on unsafe water sources. Adolescents are particularly vulnerable to contaminated water due to their higher per-unit-body-weight requirements for both food and water compared to adults and children (56).

Temporal joinpoint analysis revealed that between 2011 and 2015, the ASIR of diarrhea showed a marked decline compared to previous trends. Similarly, the ASPR of diarrhea showed a marked decline compared to previous trends between 2010 and 2015. This change may be associated with the cascading effects triggered by the pandemic during 2009–2010. One possible explanation is that during the H1N1 influenza pandemic, the prioritization of medical resources toward respiratory disease control may have reduced the completeness of diarrheal case reporting and decreased laboratory testing for diarrheal pathogens, collectively resulting in artificially low statistical data. Additionally, increased handwashing during the pandemic may have temporarily reduced the incidence of bacterial diarrheal diseases, such as shigellosis. Previous studies have shown that promoting handwashing with soap can reduce the risk of diarrhea by 30% (35). It is also worth noting that lockdowns or travel restrictions during the H1N1 pandemic may have temporarily reduced population mobility, which could also be one of the factors contributing to the observed decline in diarrhea incidence and prevalence.

Temporal joinpoint analysis also revealed that the ASIR and ASPR of diarrhea in Asia and some Asian countries demonstrated a significant resurgence from 2019 to 2021. This may be attributable to the coronavirus disease 2019 (COVID-19) pandemic. During the COVID-19 pandemic, to meet the surging demand for COVID-19 testing, many countries have adopted multiplex polymerase chain reaction technology, which enables simultaneous detection of multiple pathogens from a single sample (57). This development has produced significant spillover effects for diarrheal pathogen detection, markedly improving identification rates for *rotavirus*, *norovirus* and other pathogens. Besides, the COVID-19 pandemic has directly and indirectly affected immunization programs. In many countries, temporary disruptions to *rotavirus* vaccination programs occurred due to lockdown measures, resource shortages, parental avoidance of medical care, and supply chain interruptions, resulting in increased diarrheal cases (58–60).

Based on BAPC, the ASIR of diarrhea in Asia is predicted to decrease from 2022 to 2029 and then increase from 2029 to 2040. The ASPR of diarrhea in Asia is predicted to decrease from 2022 to 2033 and then increase from 2033 to 2040. The ASDR and ASMR are predicted to decrease continuously from 2022 to 2040. The predicted decrease in the burden of diarrhea may be attributed to the following factors. From the perspective of demography, GBD’s Population Forecasts demonstrated that the total population of Asia will increase from 4,741,493,402 in 2022 to 5,078,683,510 in 2040 as a result of longer life expectancy. However, the under-5 population will decrease from 276,370,289.4 in 2022 to 219,772,097.3 in 2040 as a result of the decline in fertility rate (61). A shift in age structure can reduce the predicted diarrheal rates. From the perspective of policy intervention, the United Nations proposed the “Goal 6: Ensure access to water and sanitation for all (SDG 6)” in the “Transforming our World: The 2030 Agenda for Sustainable Development.” This goal aims to ensure equitable access to safe and affordable drinking water and basic sanitation for all people worldwide and to improve the sustainability of water resource management (62). This goal is of great significance for curbing the breeding and transmission of diarrhea-related pathogens and reducing the risk of diarrhea. From the perspective of vaccination, rotavirus vaccines had been introduced in 110 countries (accounting for 56% of all countries) for the prevention of diarrhea as of May 6, 2021 (63). In the future, as the effectiveness and safety of the rotavirus vaccine continue to improve and new vaccines are developed, the vaccination rate may increase and the incidence of diarrhea will decrease.

Among the 13 etiologies of diarrhea in Asia, the etiology associated with the highest ASDR in both 1990 and 2021 were all *Rotavirus*; however, the etiology associated with the highest ASMR shifted from *Rotavirus* in 1990 to *Norovirus* in 2021. *Norovirus* is the causative agent of diarrhea worldwide, as well as the majority of sporadic acute gastroenteritis cases and outbreak incidents (64, 65). The high disease burden of *Norovirus* may be attributed to the following reasons. Foremost among them is that *Norovirus* has an extremely low infectious dose, requiring only 18–1,000 viral particles to cause infection (66). In contrast, bacterial pathogens such as *Salmonella* typically require an inoculum of hundreds to thousands of bacterial cells to establish infection (67). Secondly, *Norovirus* demonstrates remarkable environmental resilience, exhibiting resistance to alcohol-based disinfectants (68), tolerance to freezing temperatures (69), and the ability to persist on inanimate surfaces for over 2 weeks (70). Notably, *Norovirus* exhibits high genetic variability, particularly the GII.4 genotype (the predominant global epidemic strain), which evades immune recognition through both antigenic drift and antigenic shift (71, 72). *Rotavirus* is also a leading cause of diarrhea, particularly in young children under 5 years of age (73, 74). WHO recommended in 2008 that *Rotavirus* diarrhea vaccination be prioritized, based on studies of the global disease burden (75). In countries where *Rotavirus* vaccines have been included in national immunization programs, the disease burden of *Rotavirus*-associated diarrhea has significantly decreased (76). With the declining incidence of *Rotavirus*-associated disease, Norovirus has increasingly emerged as the leading causative agent of severe acute gastroenteritis in children across multiple countries (77).

In Afghanistan, Bahrain, Cambodia, and other 21 countries, *Vibrio cholerae* was the etiology that ranked among the top 3 causes of ASMR in both 1990 and 2021. The outbreak and spread of cholera can lead to extremely high incidence and mortality rates, leading a severe threat to public health and social stability. South Asia, particularly the Ganges-Brahmaputra Delta, has long been regarded as the “cholera hotspot.” Evidence confirms that six of the seven historically recorded cholera pandemics originated from this region (78). In addition, Asia ranks as the second highest region for reported cholera cases globally in recent years, surpassed only by Africa (79). The increasing ease of human mobility has significantly elevated the risk of cross-border cholera transmission from endemic zones, while *Vibrio Cholerae* finds more opportunities to proliferate and spread in areas with suboptimal sanitation infrastructure. These realities underscore the critical necessity for coordinated global action in cholera monitoring and containment. We therefore recommend that countries maintain sustained vigilance and strengthen surveillance systems, enabling the development of rapid response protocols to minimize the disease burden caused by this ancient scourge.

Apart from *Vibrio cholerae*, other bacteria such as *Shigella* are also significant pathogens contributing to the burden of diarrhea. In countries including Azerbaijan, Nepal, Oman, and Tajikistan, *Shigella*-associated diarrhea ranks among the top three causes of diarrhea DALY across 13 etiologies. The high drug resistance of *Shigella* may be the main reason for its high diarrhea burden. Currently, the global resistance rate of *Shigella* spp. to first-line antibiotics (such as ciprofloxacin and azithromycin) exceeds 50% (80). Moreover, extensively drug-resistant (XDR) *Shigella*, which is resistant to both fluoroquinolones, third-generation cephalosporins, and azithromycin, has emerged, and international travel and migration may accelerate the spread of XDR strains (81).

## 5 Conclusion

Compared with 1990, the burden of diarrhea in Asia in 2021 showed a significant decrease, except for some southeast Asian countries. The burden of diarrhea exhibited a significant negative association with socioeconomic development levels across Asian countries. The age group with the highest incidence and prevalence of diarrhea was the 10–14 years old group in 2021. Based on the BAPC model, both of the overall trends of ASIR and ASPR for diarrhea in Asia from 2022 to 2040 are predicted to initially decrease and then increase. Among the etiologies of diarrhea, the etiology associated with the highest ASDR in both 1990 and 2021 were all *rotavirus*. The etiology associated with the highest ASMR was *Norovirus* in 2021, which replaced *Rotavirus* in 1990. In the future, countries should continue to advance WASH initiatives, enhancing clean water supply and sewage treatment infrastructure in rural areas and slums and improving the construction and maintenance of public toilets, particularly in low- and middle-income countries. Additionally, countries should actively establish diarrheal pathogen surveillance networks (e.g., for *Rotavirus*, *Vibrio Cholerae*, *Norovirus*, etc.), with a particular focus on high-risk regions and seasonal outbreaks. Vaccination against *Norovirus* and *Rotavirus* should be further expanded, with additional promotion of oral cholera vaccines in cholera-endemic regions (e.g., Afghanistan, Bahrain, Cambodia). Moreover, age-specific interventions should be implemented to reduce the burden of diarrhea. For school-aged children and adolescents, interventions should focus on reducing cluster infections in educational settings through improved WASH infrastructure, including drinking water facilities and sanitation. For older adults, priority should be given to mitigating diarrhea-associated DALY and mortality burden.

## Data Availability

Publicly available datasets were analyzed in this study. This data can be found here: https://www.healthdata.org/.

## Footnotes

^1^ https://vizhub.healthdata.org/gbd-results/
