# Supplementary material for: The burden and etiologies of diarrhea in Asia and its countries from 1990 to 2021 and the forecast to 2040: analyses informed by the global burden of disease study 2021
Source: Front Public Health. 2025 Aug 6;13:1651315. doi: 10.3389/fpubh.2025.1651315 (PMC12364947; doi:10.3389/fpubh.2025.1651315)
Supplement: Supplementary file 1 [file Data_Sheet_1.PDF]

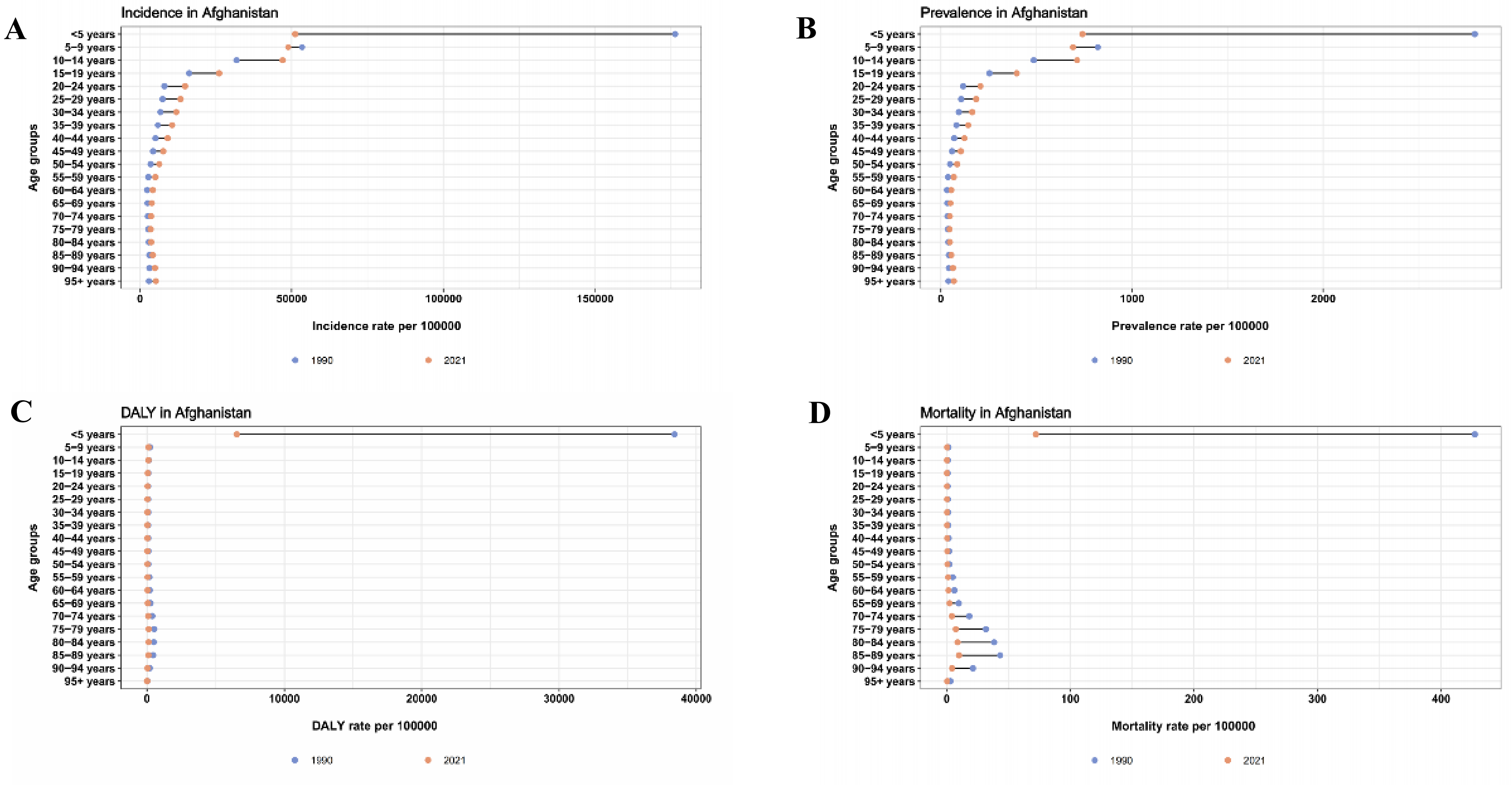

Figure S1: Age diversities of diarrheal diseases burden and time trends in Afghanistan.

(A) Age diversities of incidence; (B) Age diversities of prevalence; (C) Age diversities of DALY rate; (D) Age diversities of mortality.

DALY, disability-adjusted life year.

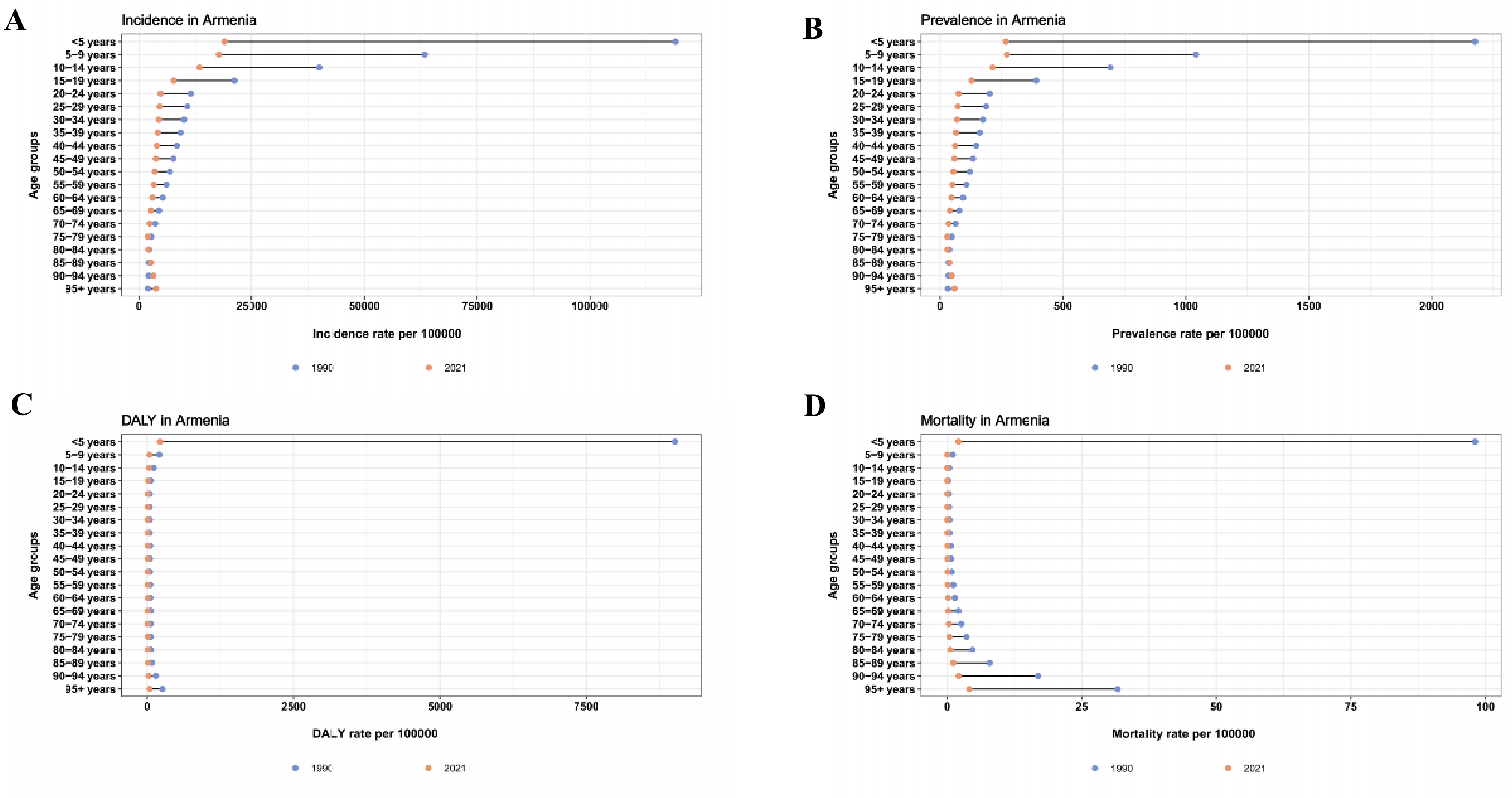

Figure S2: Age diversities of diarrheal diseases burden and time trends in Armenia.

(A) Age diversities of incidence; (B) Age diversities of prevalence; (C) Age diversities of DALY rate; (D) Age diversities of mortality.

DALY, disability-adjusted life year.

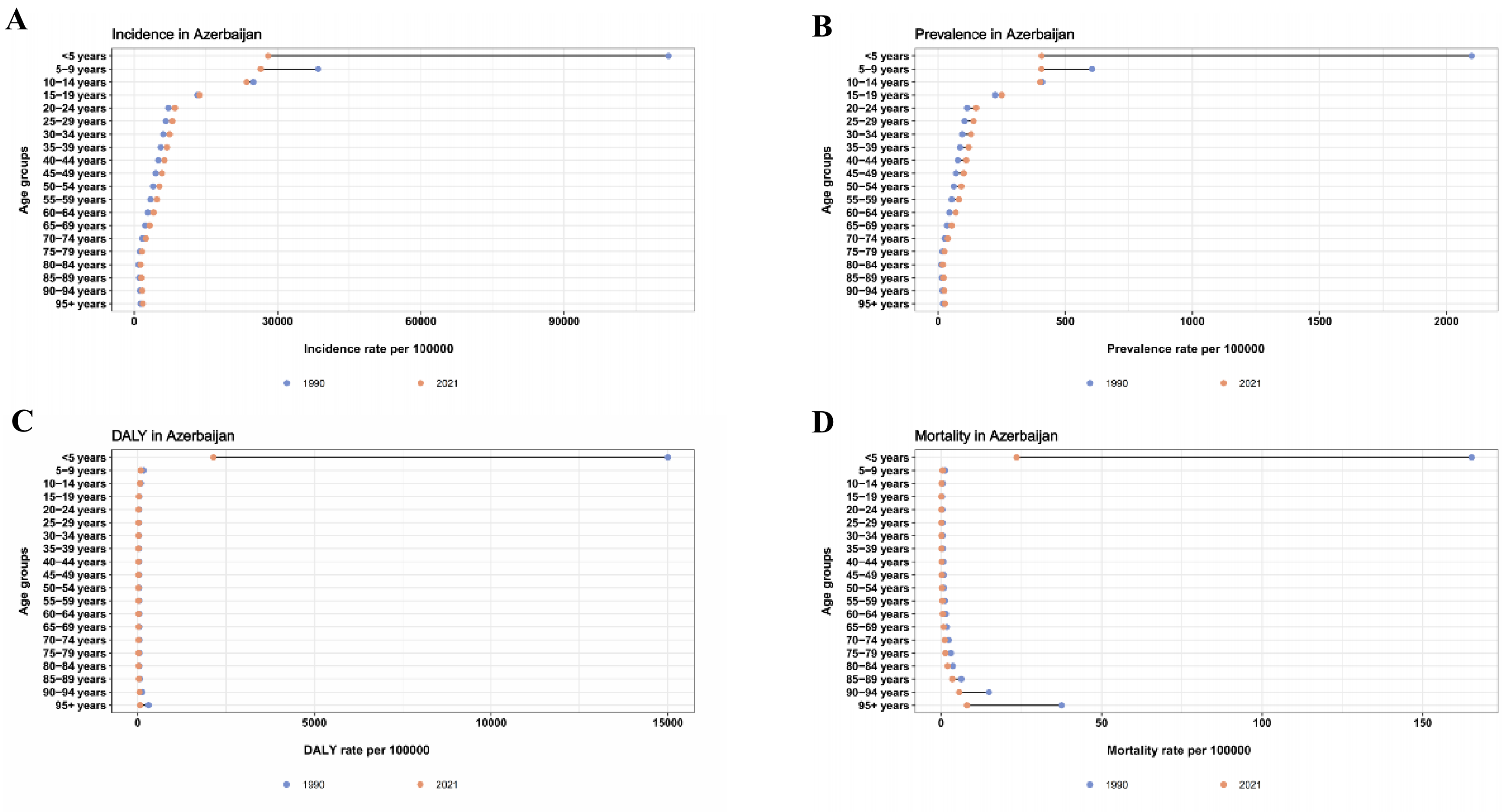

Figure S3: Age diversities of diarrheal diseases burden and time trends in Azerbaijan.

(A) Age diversities of incidence; (B) Age diversities of prevalence; (C) Age diversities of DALY rate; (D) Age diversities of mortality.

DALY, disability-adjusted life year.

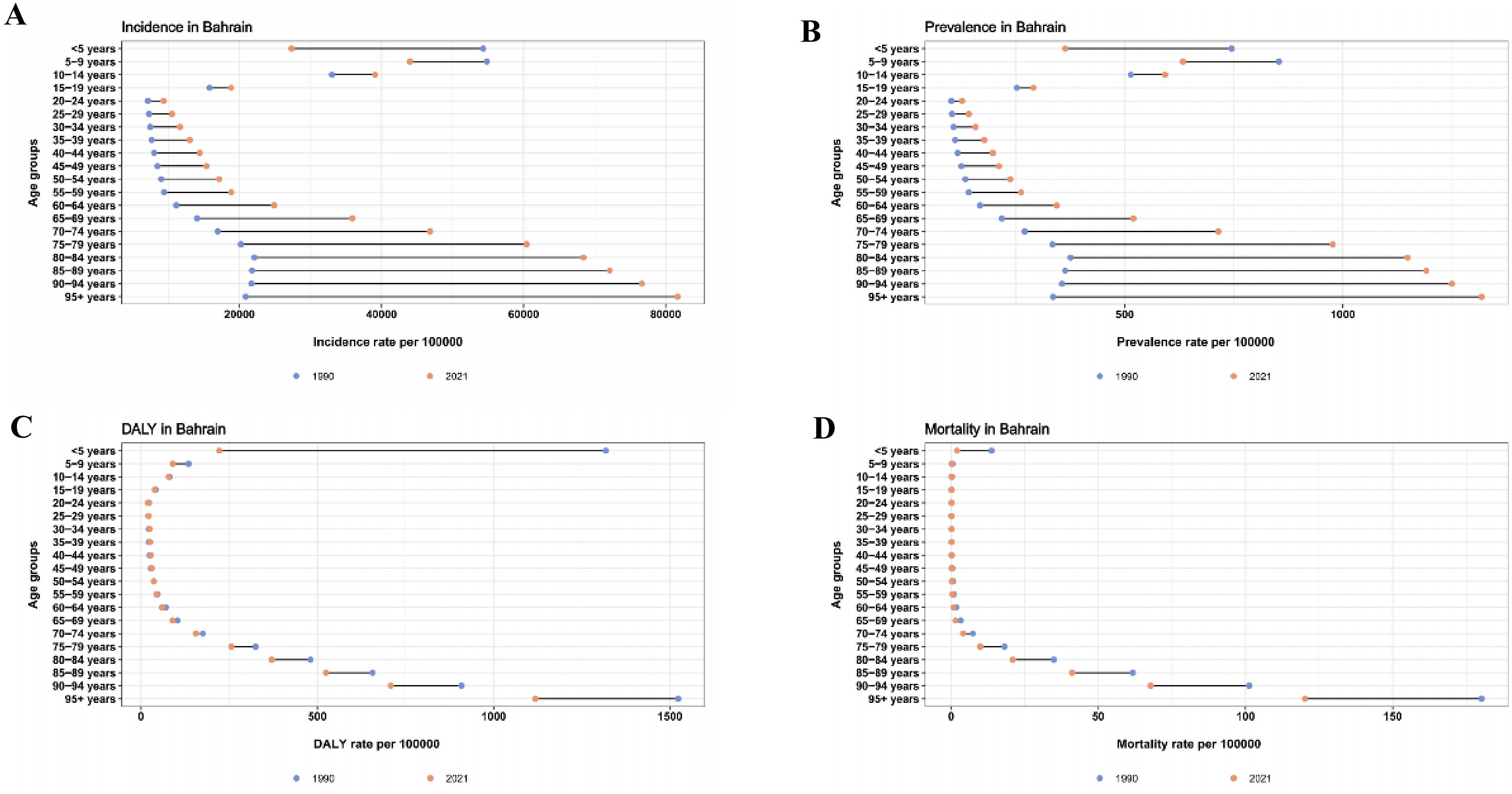

Figure S4: Age diversities of diarrheal diseases burden and time trends in Bahrain.

(A) Age diversities of incidence; (B) Age diversities of prevalence; (C) Age diversities of DALY rate; (D) Age diversities of mortality.

DALY, disability-adjusted life year.

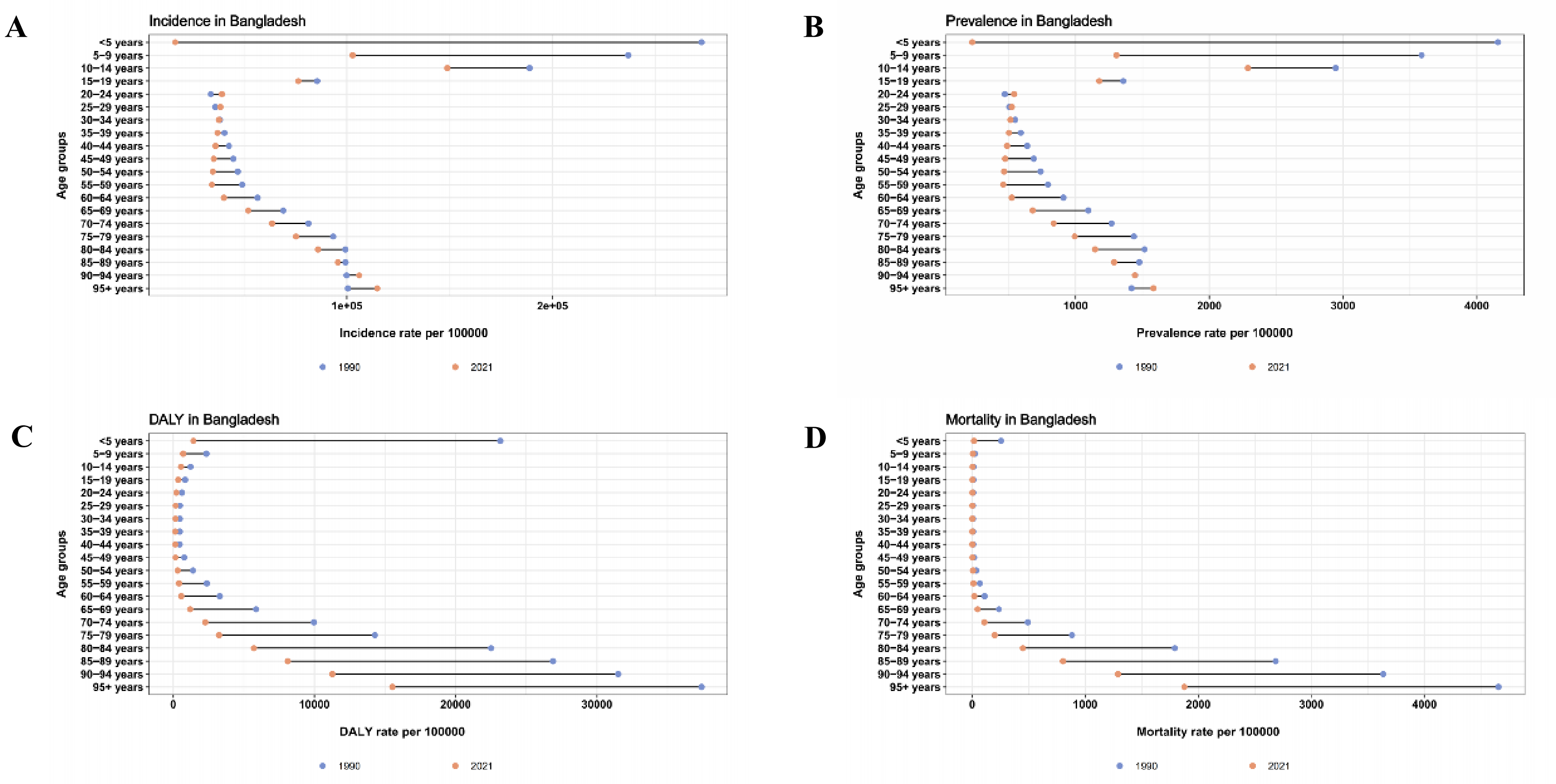

Figure S5: Age diversities of diarrheal diseases burden and time trends in Bangladesh.

(A) Age diversities of incidence; (B) Age diversities of prevalence; (C) Age diversities of DALY rate; (D) Age diversities of mortality.

DALY, disability-adjusted life year.

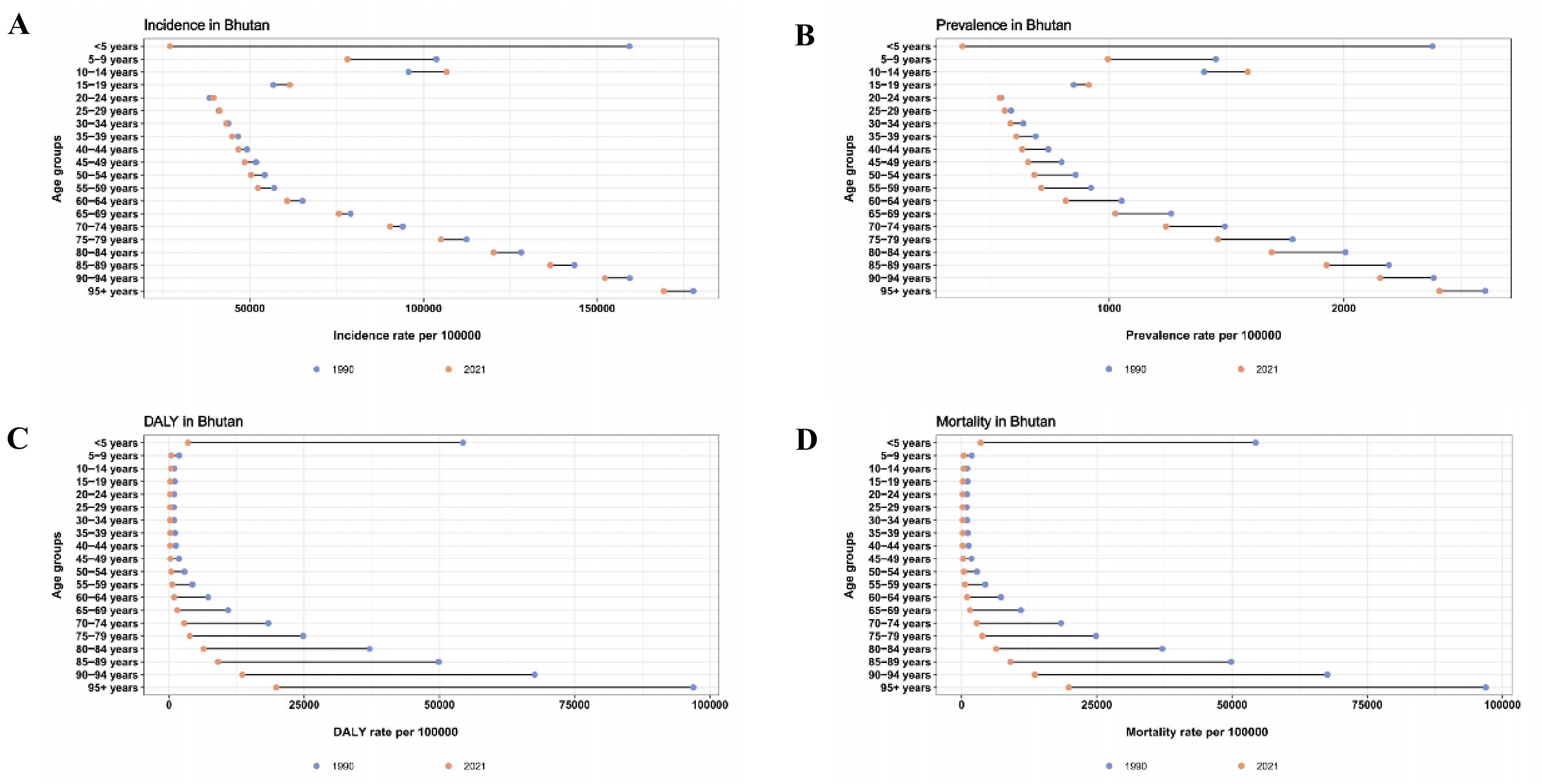

Figure S6: Age diversities of diarrheal diseases burden and time trends in Bhutan.

(A) Age diversities of incidence; (B) Age diversities of prevalence; (C) Age diversities of DALY rate; (D) Age diversities of mortality.

DALY, disability-adjusted life year.

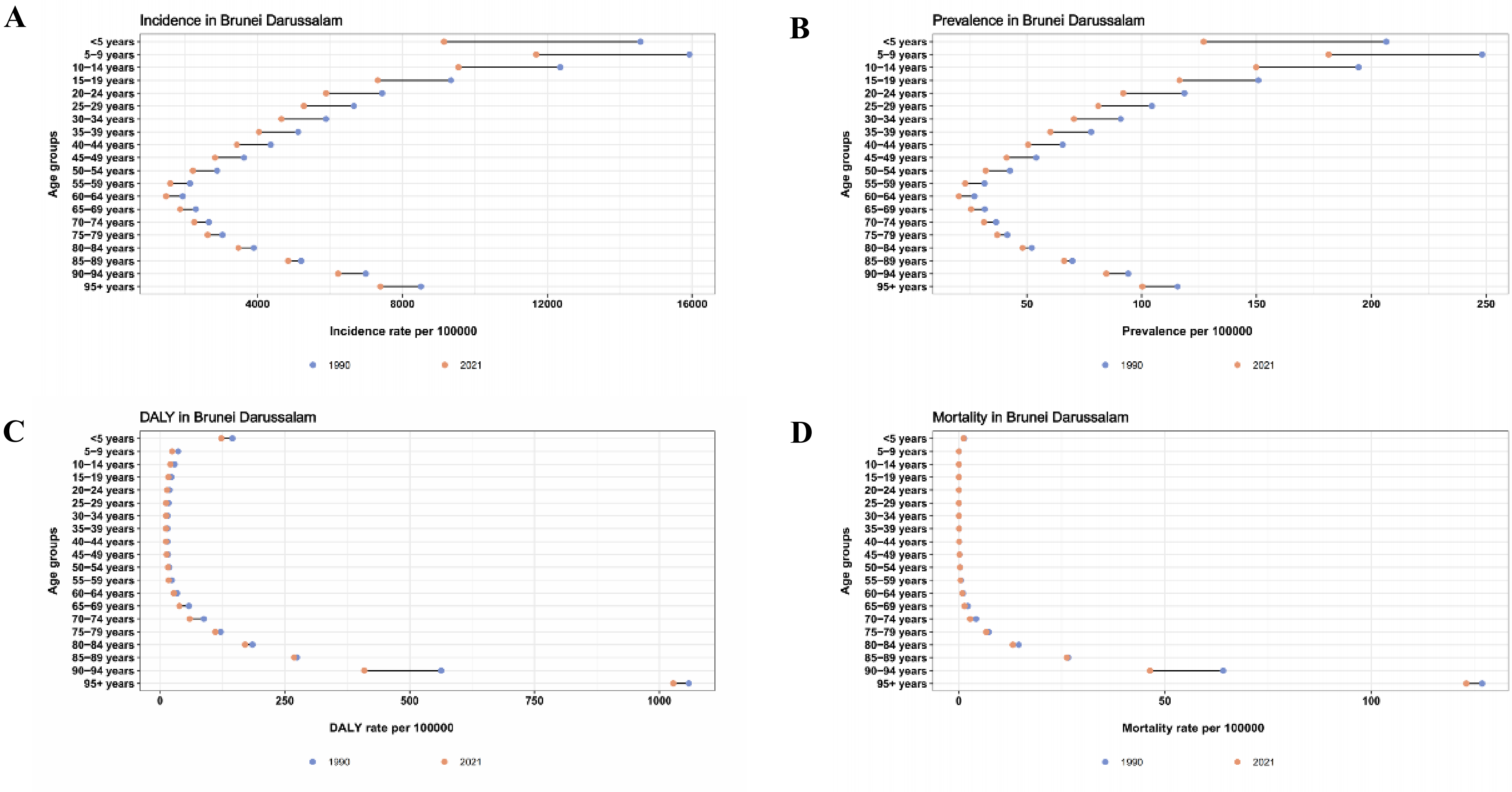

Figure S7: Age diversities of diarrheal diseases burden and time trends in Brunei Darussalam.

(A) Age diversities of incidence; (B) Age diversities of prevalence; (C) Age diversities of DALY rate; (D) Age diversities of mortality.

DALY, disability-adjusted life year.

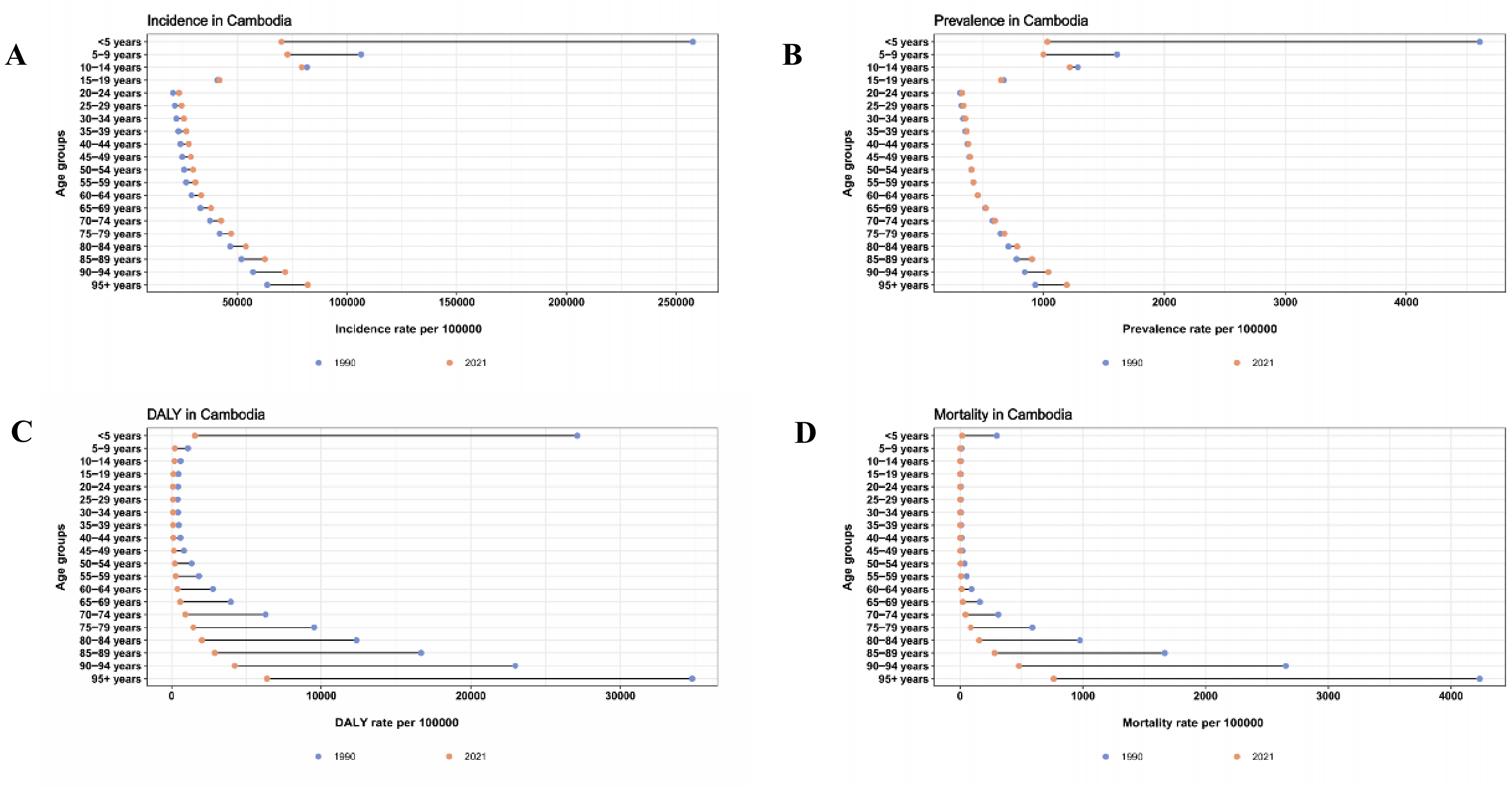

Figure S8: Age diversities of diarrheal diseases burden and time trends in Cambodia.

(A) Age diversities of incidence; (B) Age diversities of prevalence; (C) Age diversities of DALY rate; (D) Age diversities of mortality.

DALY, disability-adjusted life year.

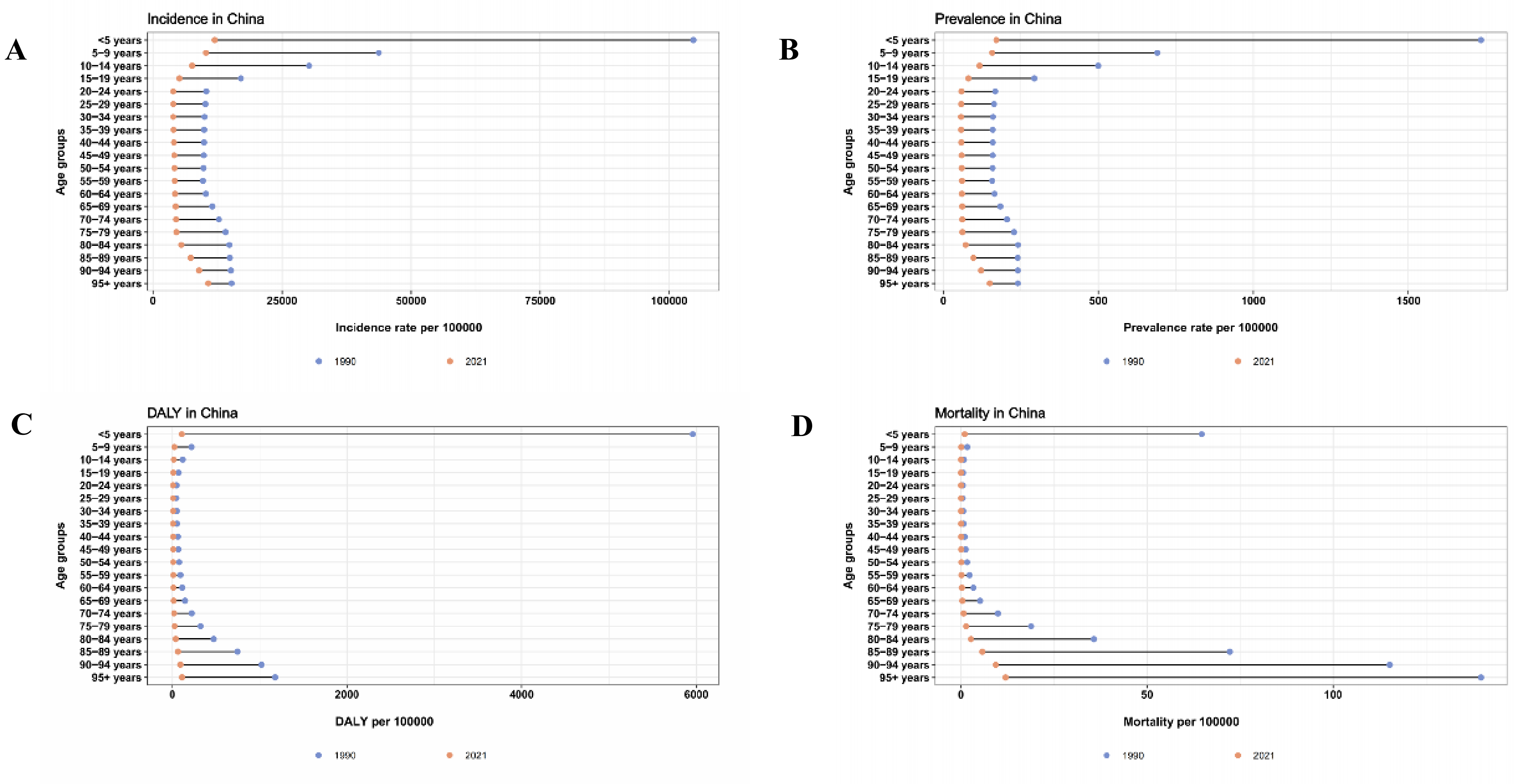

Figure S9: Age diversities of diarrheal diseases burden and time trends in China.

(A) Age diversities of incidence; (B) Age diversities of prevalence; (C) Age diversities of DALY rate; (D) Age diversities of mortality.

DALY, disability-adjusted life year.

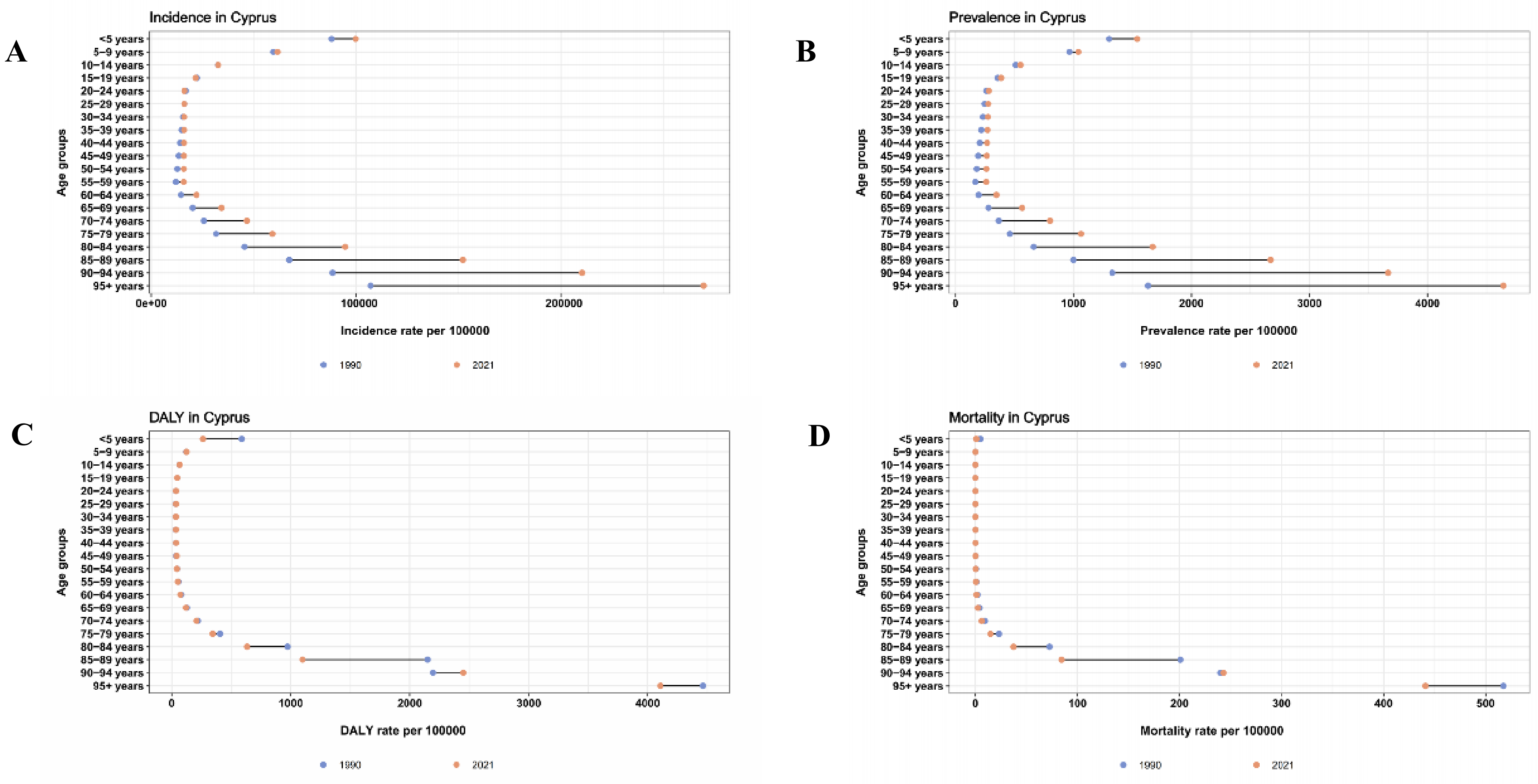

Figure S10: Age diversities of diarrheal diseases burden and time trends in Cyprus.

(A) Age diversities of incidence; (B) Age diversities of prevalence; (C) Age diversities of DALY rate; (D) Age diversities of mortality.

DALY, disability-adjusted life year.

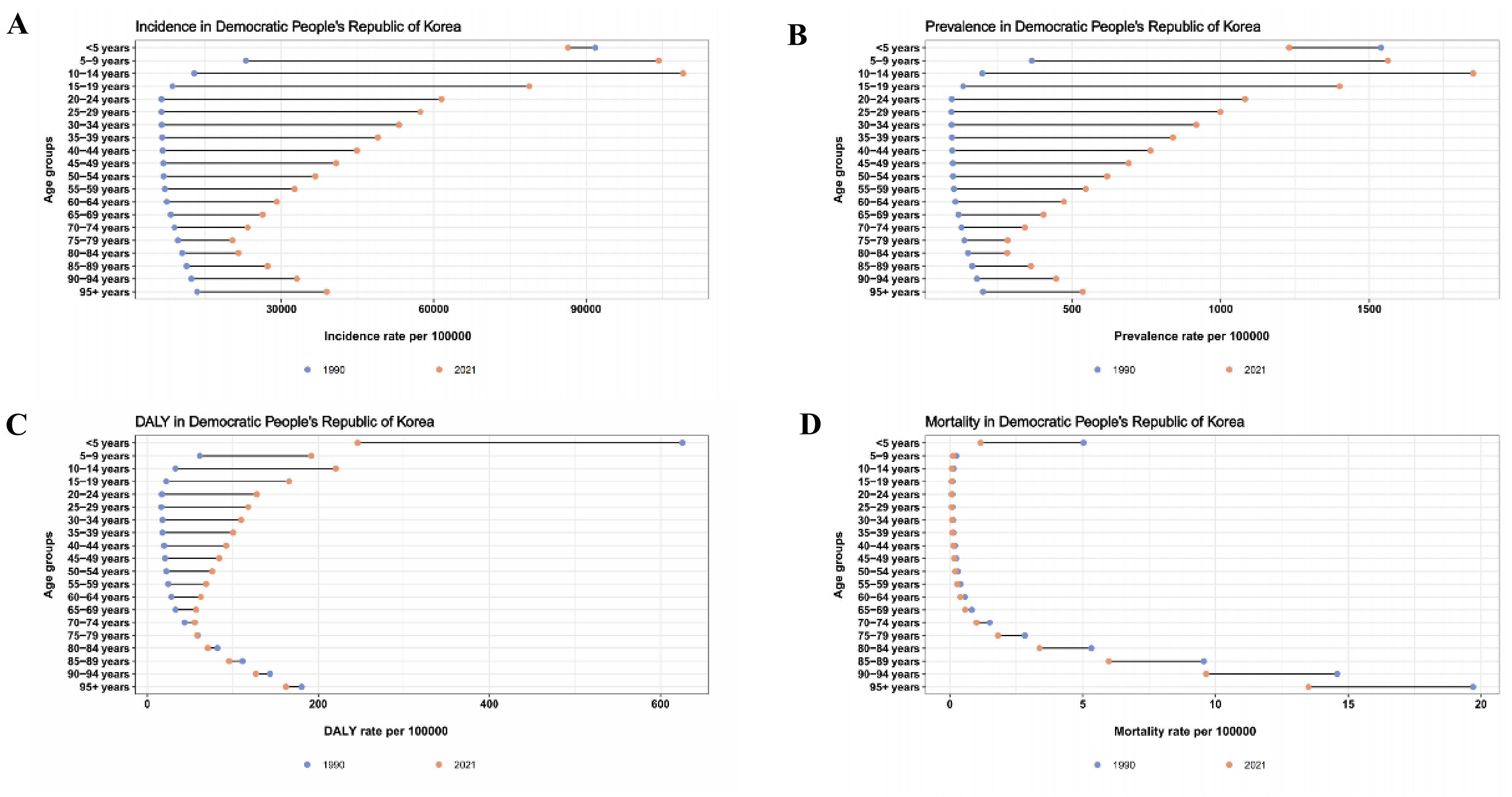

Figure S11: Age diversities of diarrheal diseases burden and time trends in Democratic People's Republic of Korea.
(A) Age diversities of incidence; (B) Age diversities of prevalence; (C) Age diversities of DALY rate; (D) Age diversities of mortality.
DALY, disability-adjusted life year.

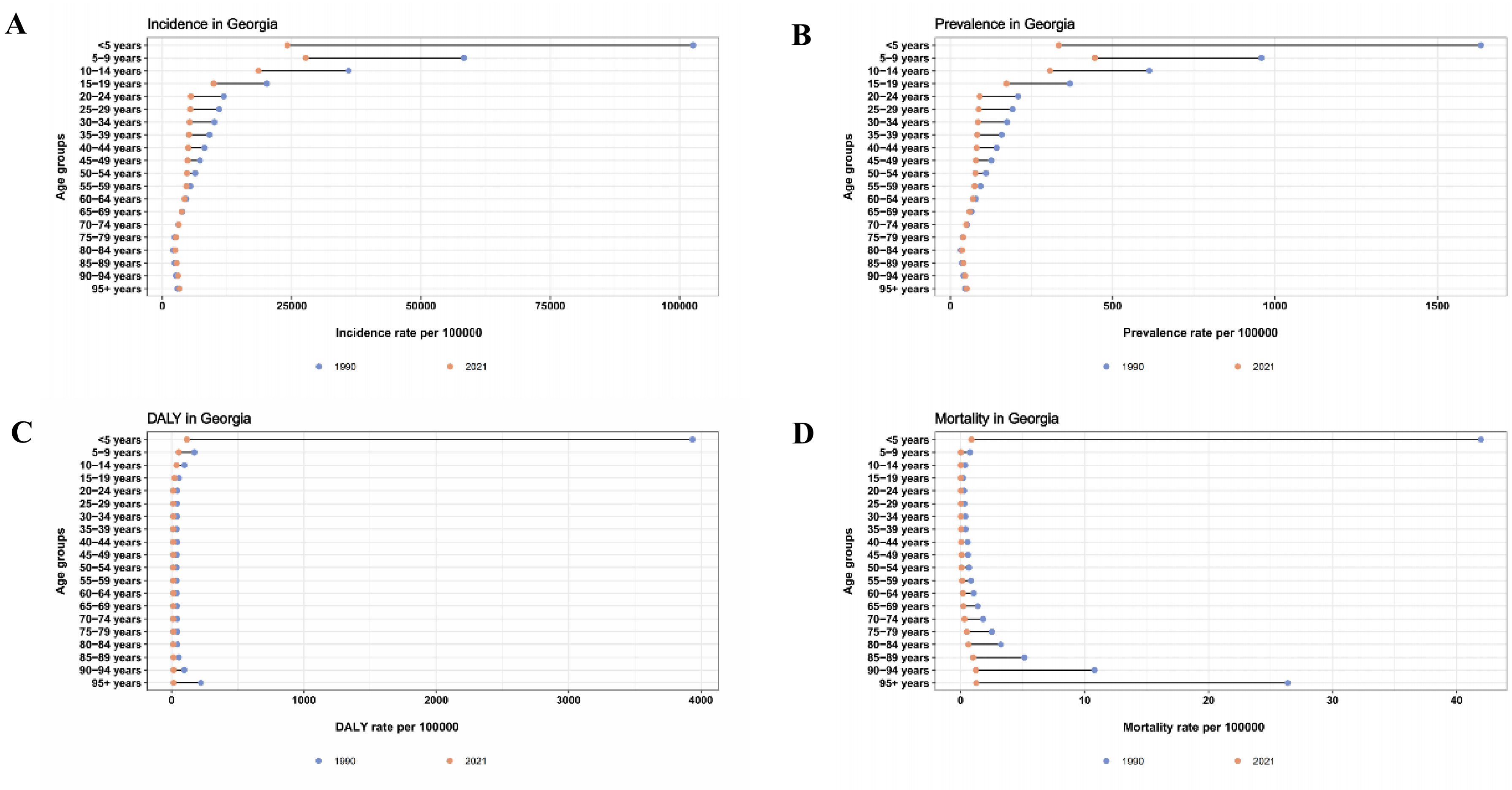

Figure S12: Age diversities of diarrheal diseases burden and time trends in Georgia.

(A) Age diversities of incidence; (B) Age diversities of prevalence; (C) Age diversities of DALY rate; (D) Age diversities of mortality.

DALY, disability-adjusted life year.

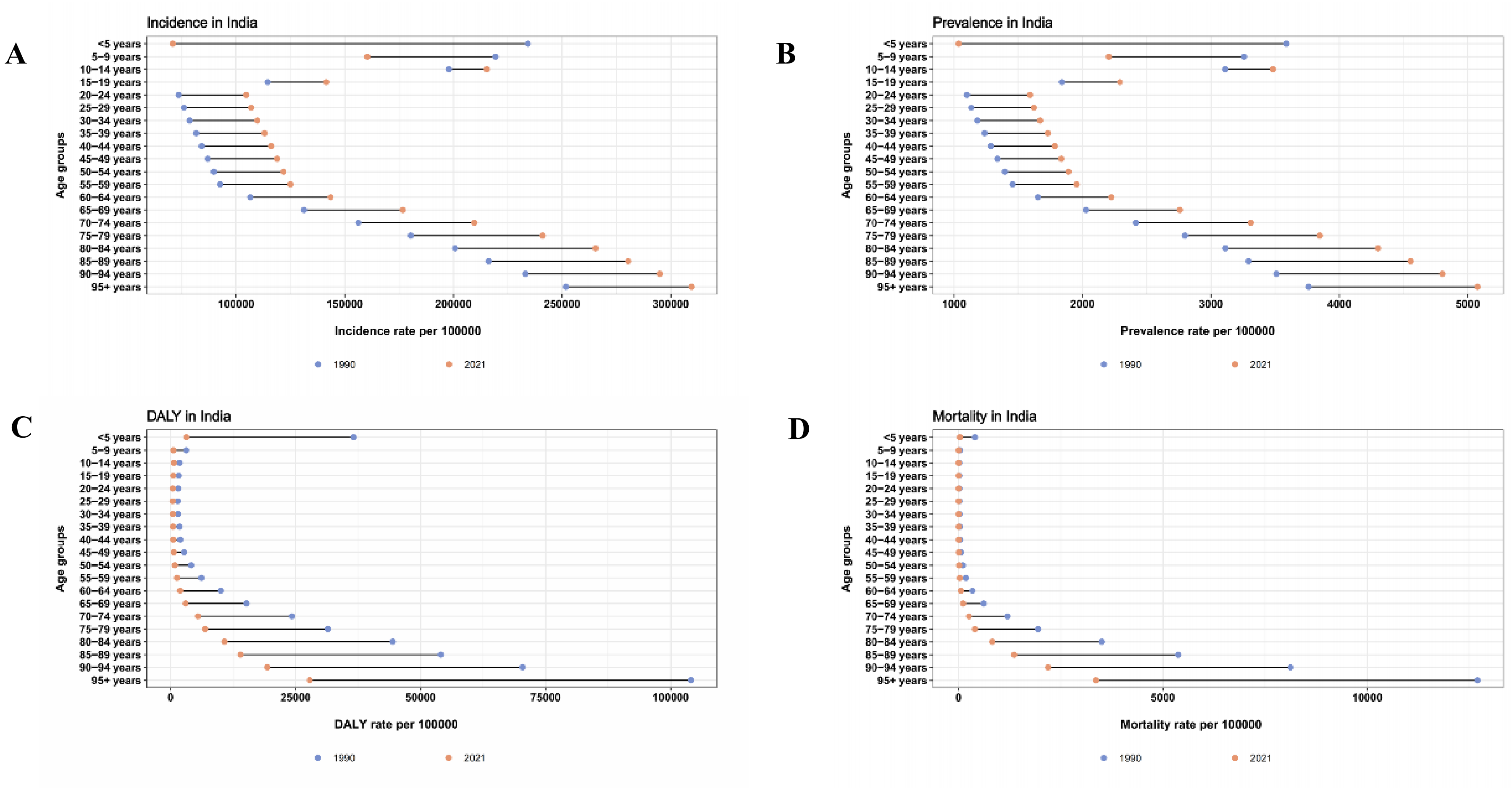

Figure S13: Age diversities of diarrheal diseases burden and time trends in India.

(A) Age diversities of incidence; (B) Age diversities of prevalence; (C) Age diversities of DALY rate; (D) Age diversities of mortality.

DALY, disability-adjusted life year.

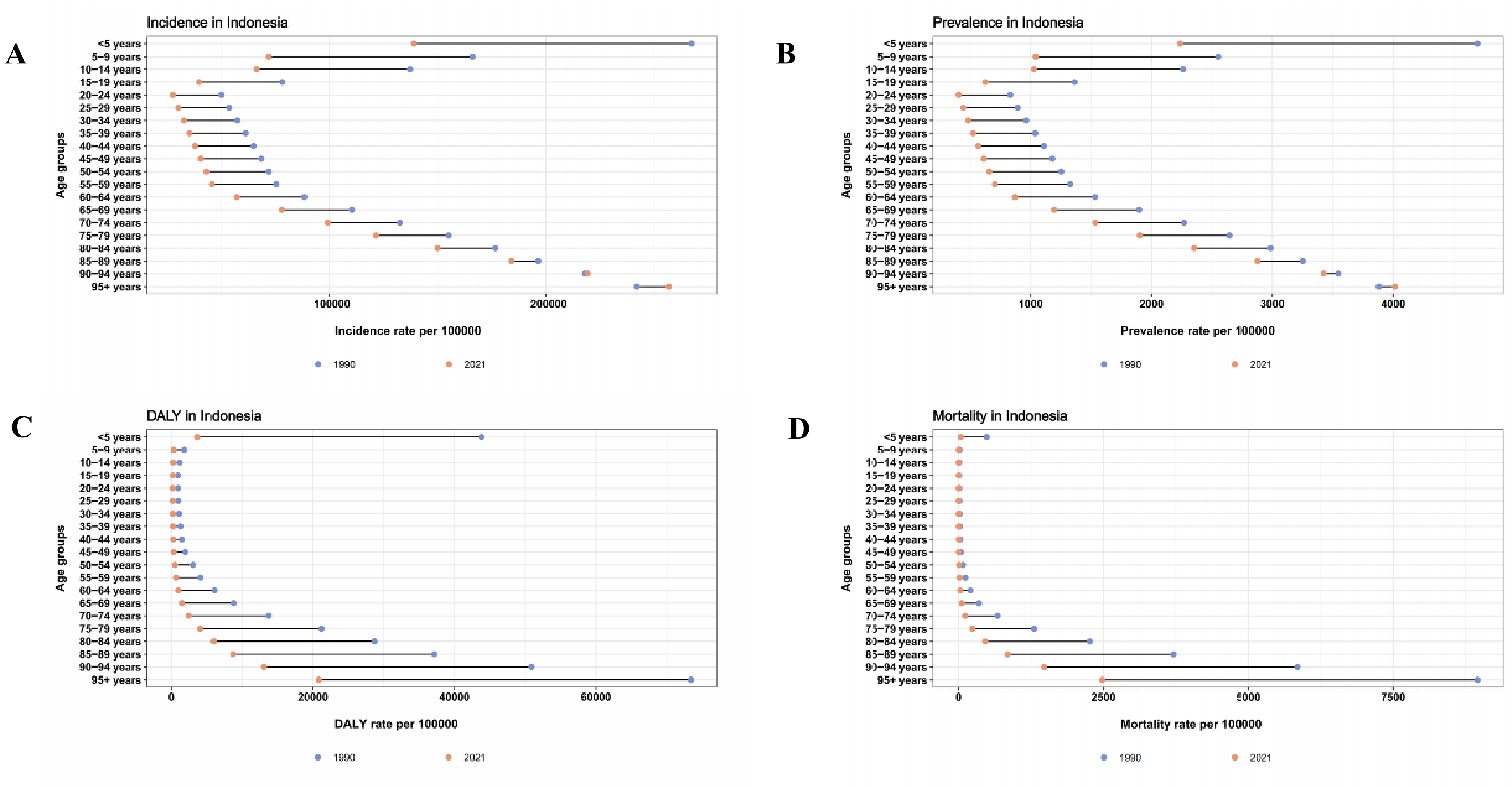

Figure S14: Age diversities of diarrheal diseases burden and time trends in Indonesia.

(A) Age diversities of incidence; (B) Age diversities of prevalence; (C) Age diversities of DALY rate; (D) Age diversities of mortality.

DALY, disability-adjusted life year.

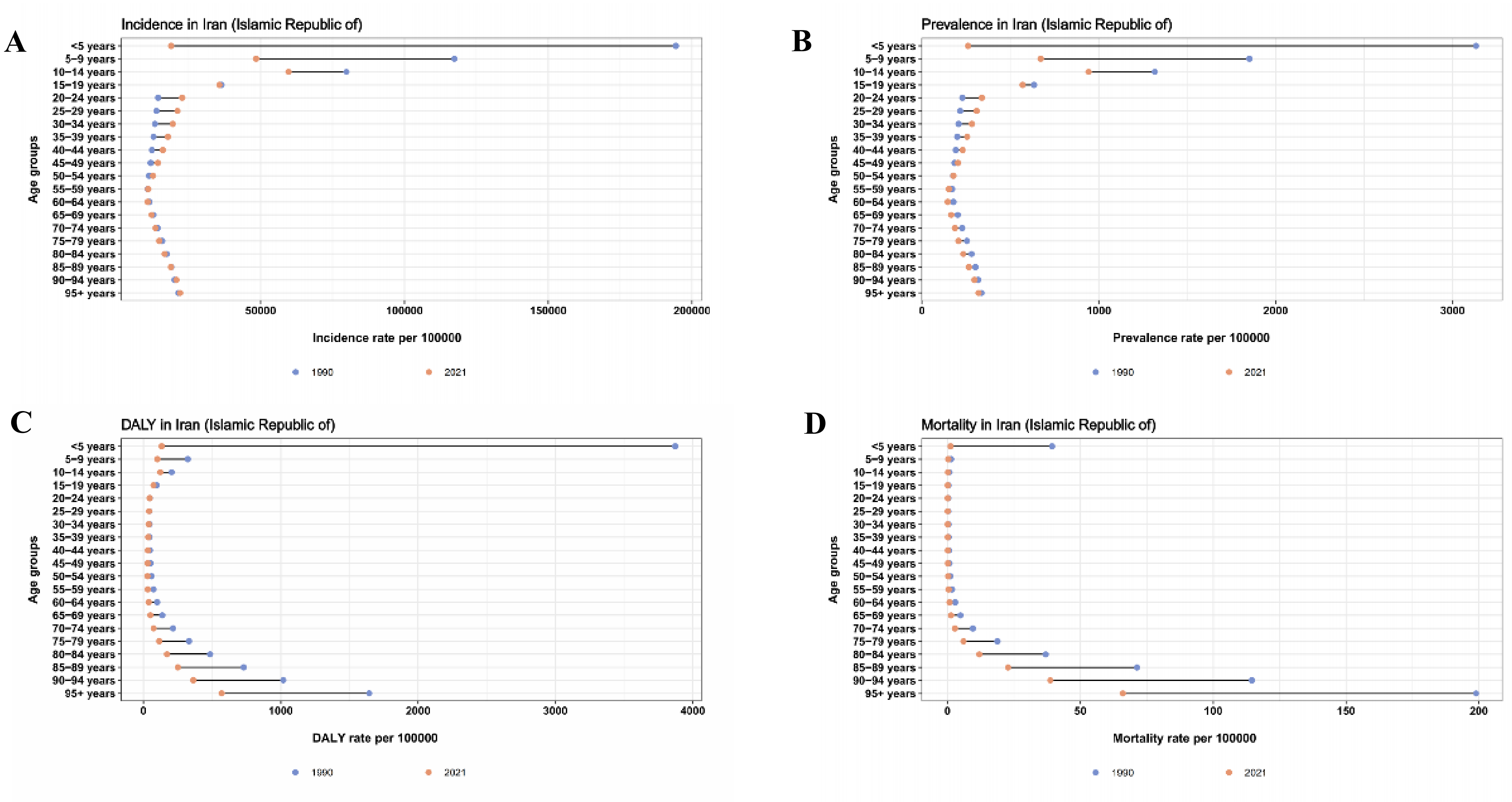

Figure S15: Age diversities of diarrheal diseases burden and time trends in Iran (Islamic Republic of).

(A) Age diversities of incidence; (B) Age diversities of prevalence; (C) Age diversities of DALY rate; (D) Age diversities of mortality.

DALY, disability-adjusted life year.

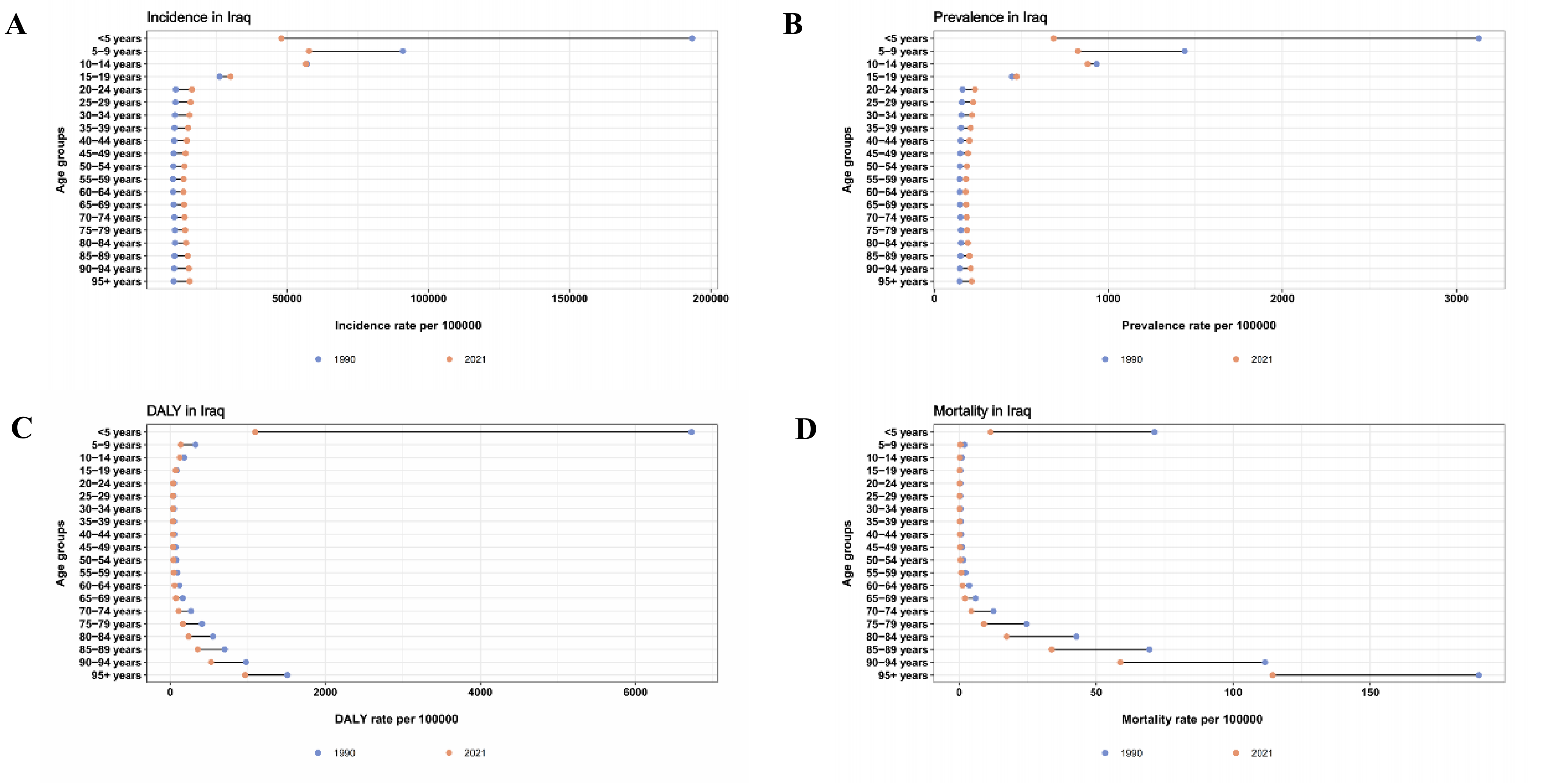

Figure S16: Age diversities of diarrheal diseases burden and time trends in Iraq.

(A) Age diversities of incidence; (B) Age diversities of prevalence; (C) Age diversities of DALY rate; (D) Age diversities of mortality.

DALY, disability-adjusted life year.

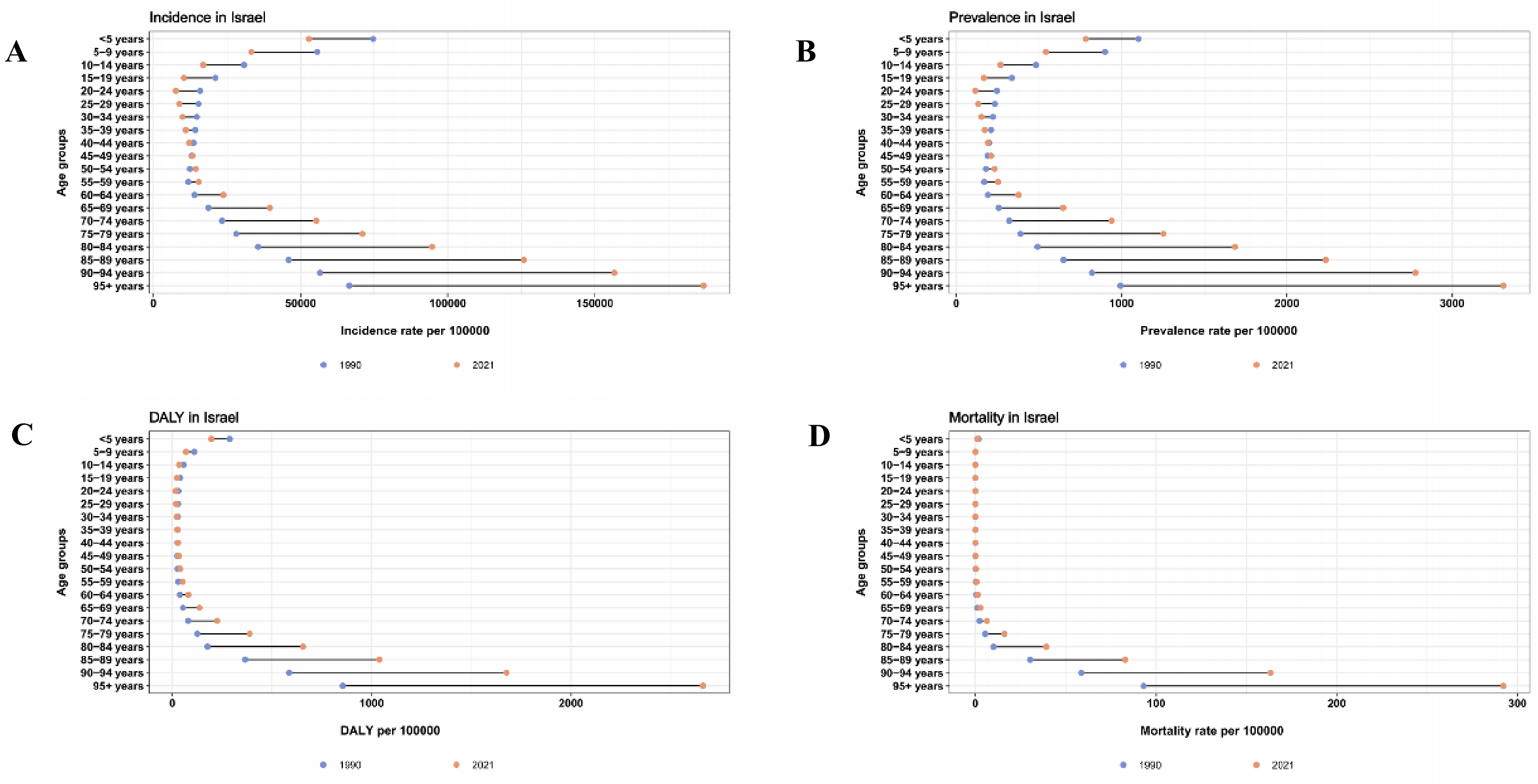

Figure S17: Age diversities of diarrheal diseases burden and time trends in Israel.

(A) Age diversities of incidence; (B) Age diversities of prevalence; (C) Age diversities of DALY rate; (D) Age diversities of mortality.

DALY, disability-adjusted life year.

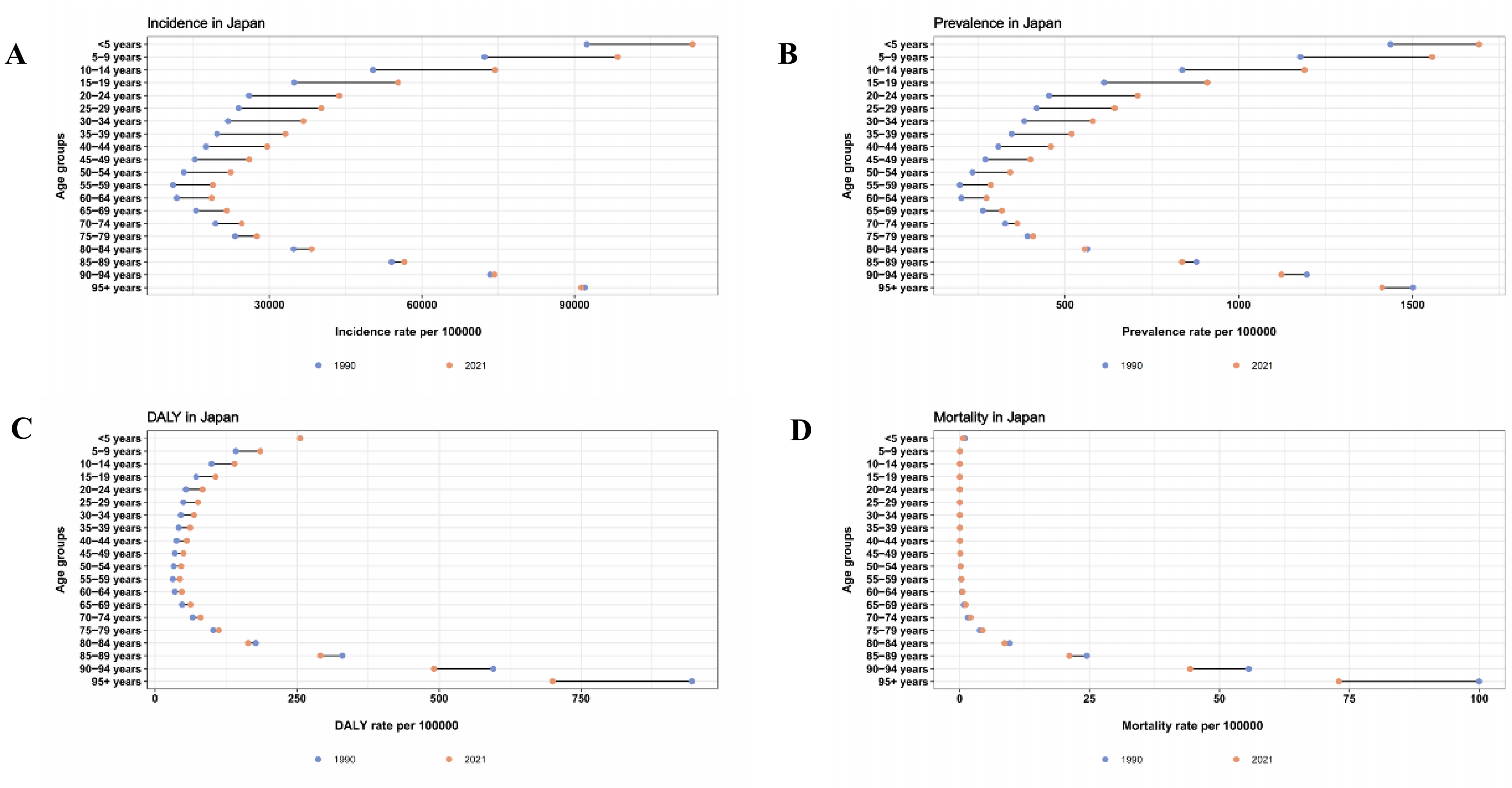

Figure S18: Age diversities of diarrheal diseases burden and time trends in Japan.

(A) Age diversities of incidence; (B) Age diversities of prevalence; (C) Age diversities of DALY rate; (D) Age diversities of mortality.

DALY, disability-adjusted life year.

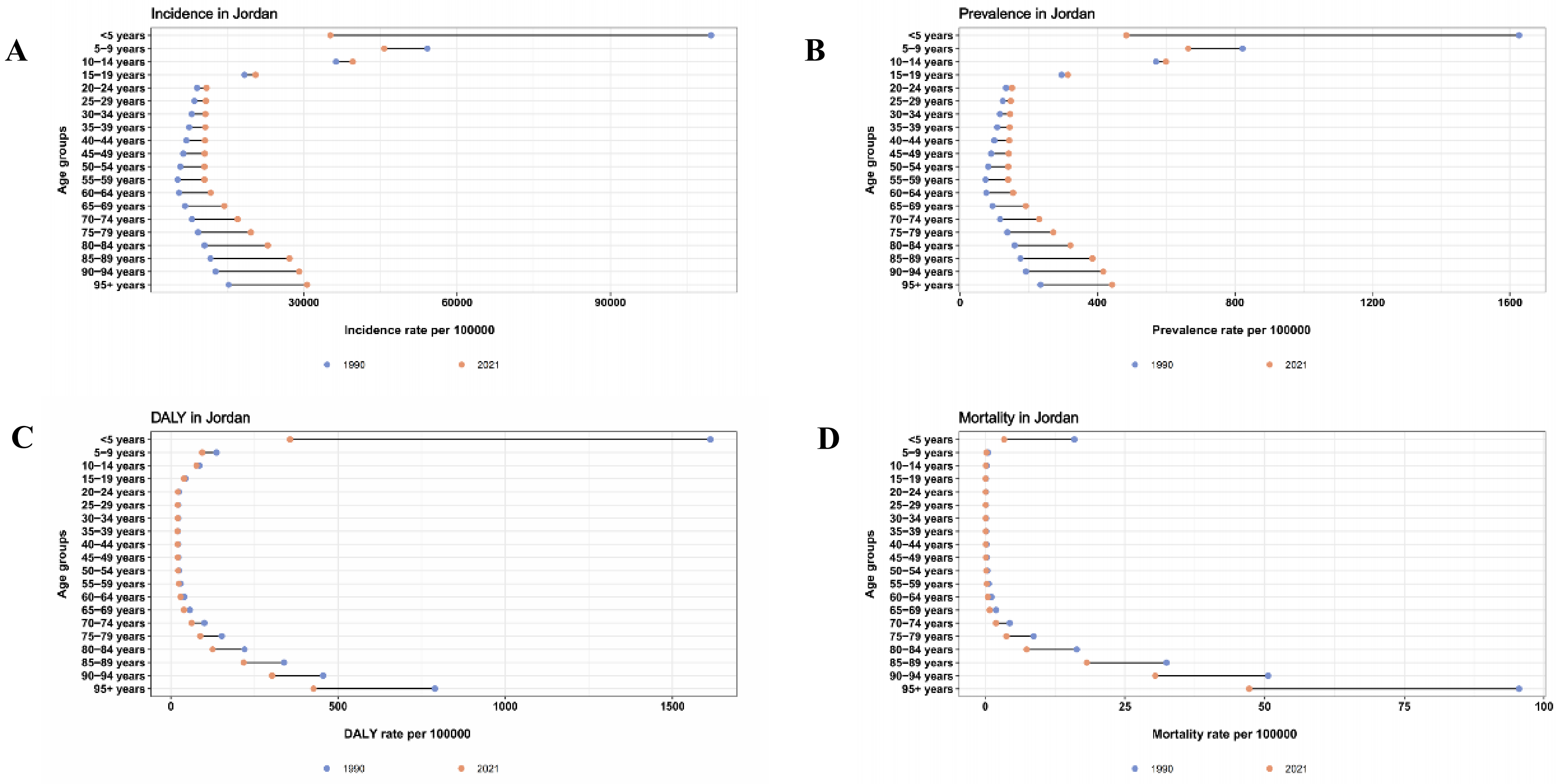

Figure S19: Age diversities of diarrheal diseases burden and time trends in Jordan.

(A) Age diversities of incidence; (B) Age diversities of prevalence; (C) Age diversities of DALY rate; (D) Age diversities of mortality.

DALY, disability-adjusted life year.

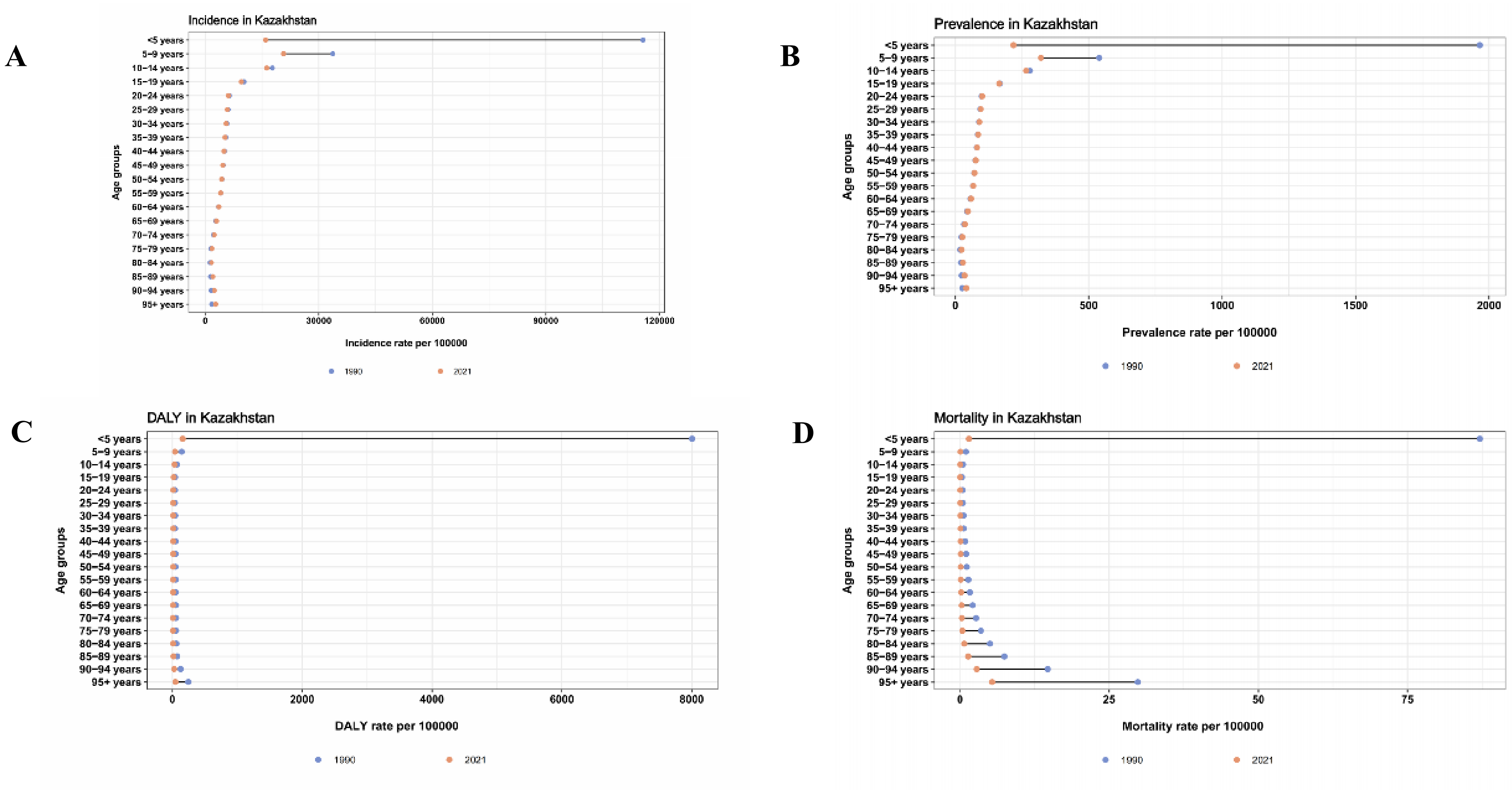

Figure S20: Age diversities of diarrheal diseases burden and time trends in Kazakhstan.

(A) Age diversities of incidence; (B) Age diversities of prevalence; (C) Age diversities of DALY rate; (D) Age diversities of mortality.

DALY, disability-adjusted life year.

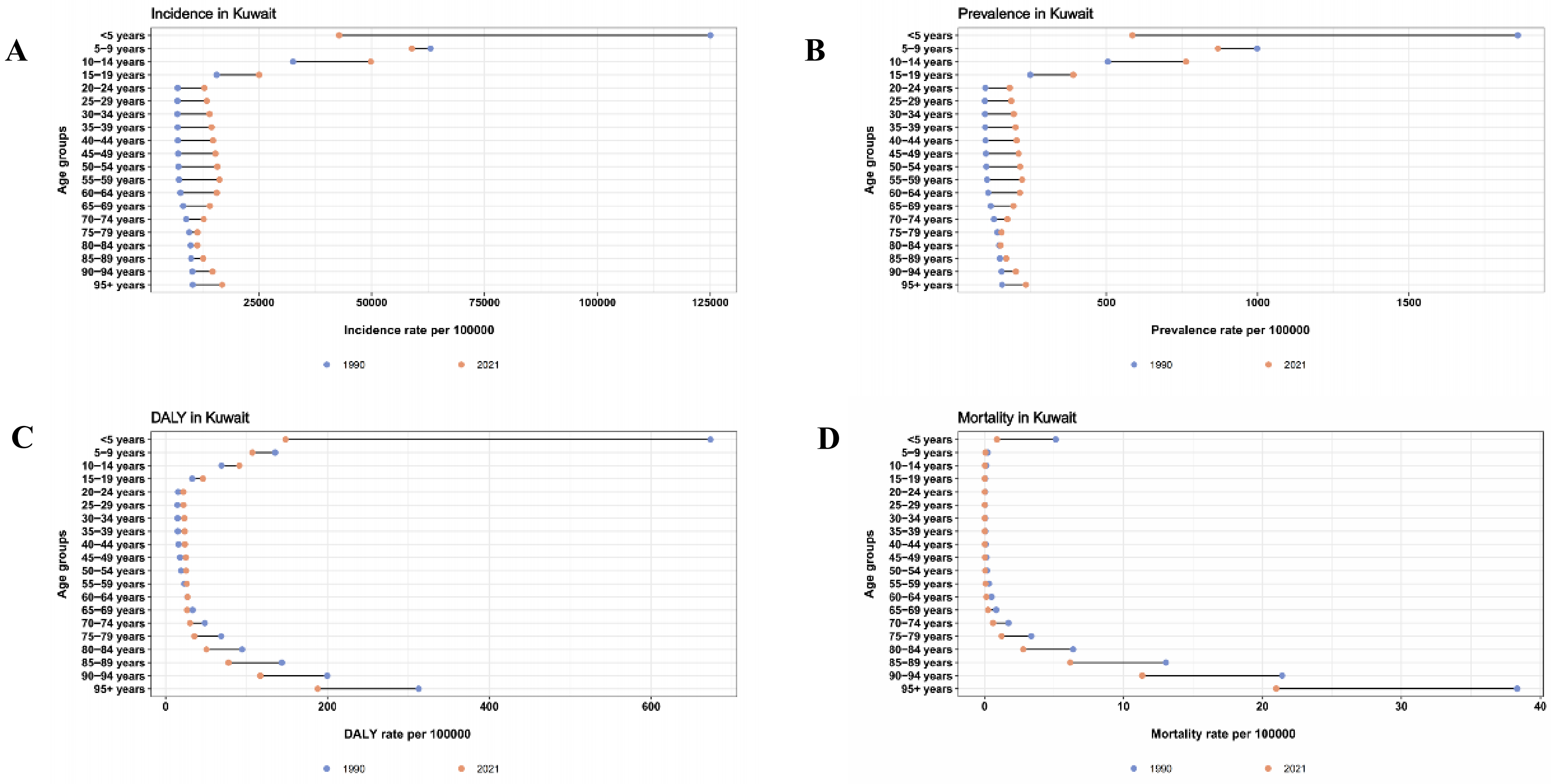

Figure S21: Age diversities of diarrheal diseases burden and time trends in Kuwait.

(A) Age diversities of incidence; (B) Age diversities of prevalence; (C) Age diversities of DALY rate; (D) Age diversities of mortality.

DALY, disability-adjusted life year.

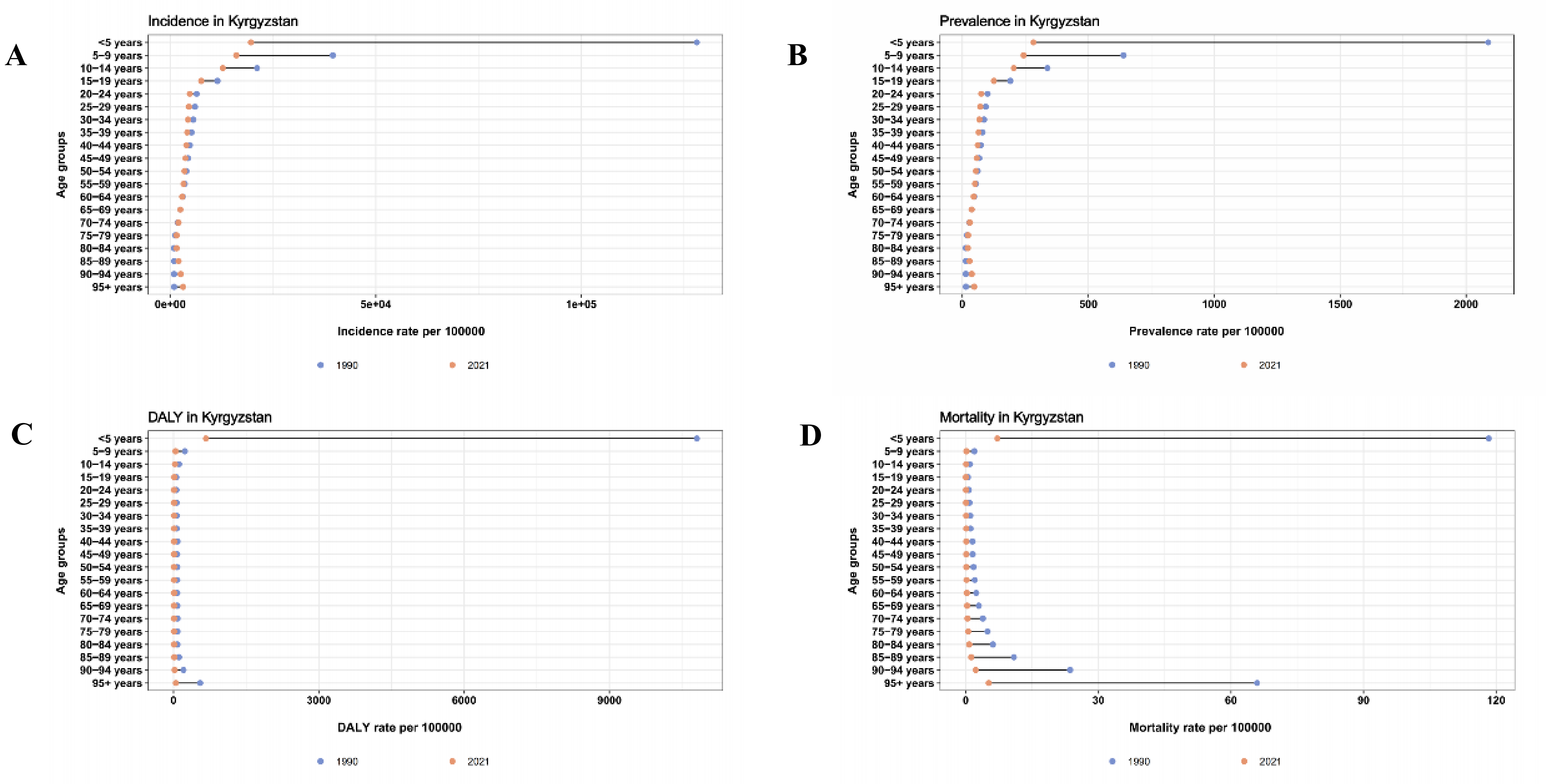

Figure S22: Age diversities of diarrheal diseases burden and time trends in Kyrgyzstan.

(A) Age diversities of incidence; (B) Age diversities of prevalence; (C) Age diversities of DALY rate; (D) Age diversities of mortality.

DALY, disability-adjusted life year.

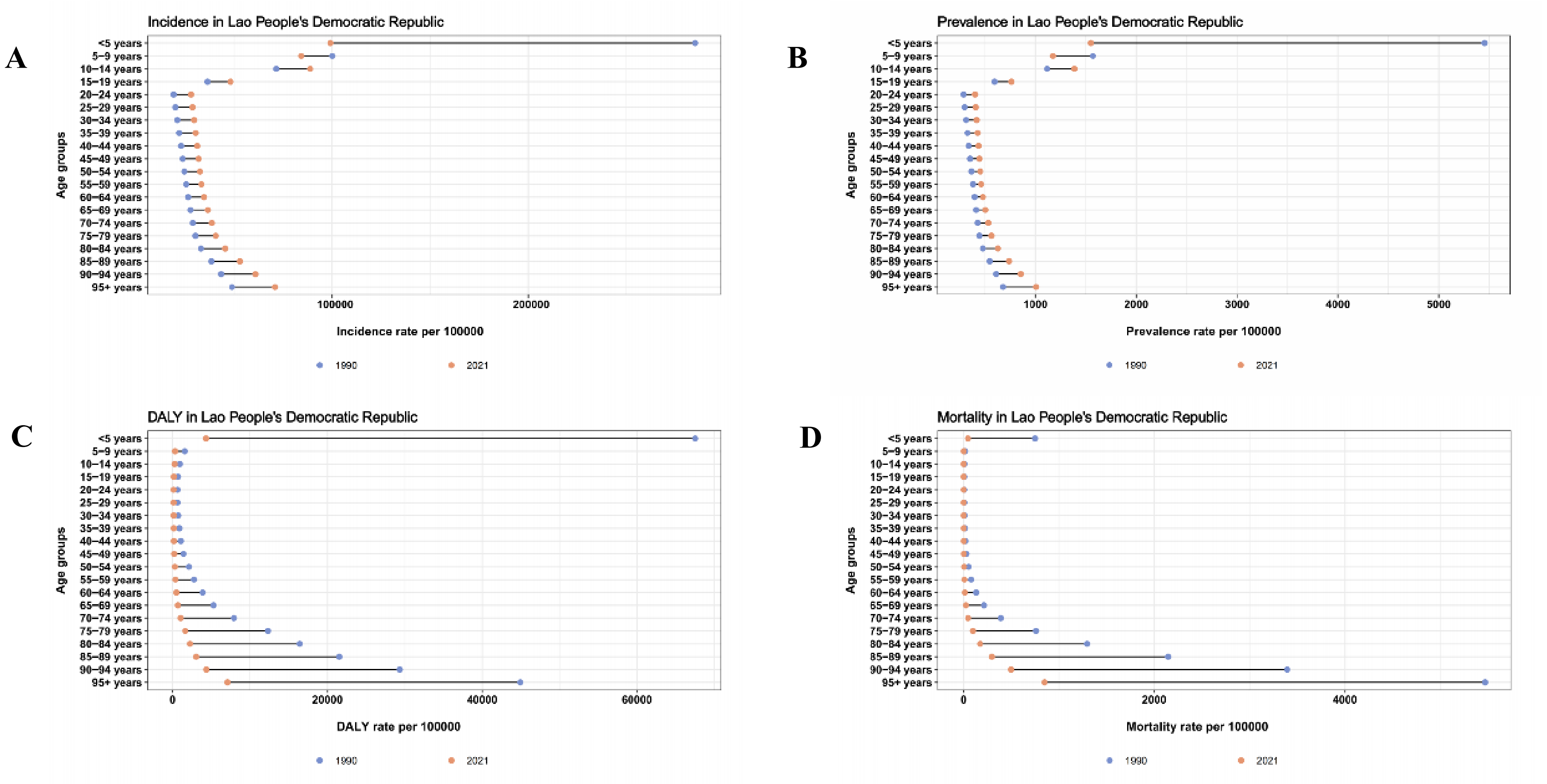

Figure S23: Age diversities of diarrheal diseases burden and time trends in Lao People's Democratic Republic.
(A) Age diversities of incidence; (B) Age diversities of prevalence; (C) Age diversities of DALY rate; (D) Age diversities of mortality.
DALY, disability-adjusted life year.

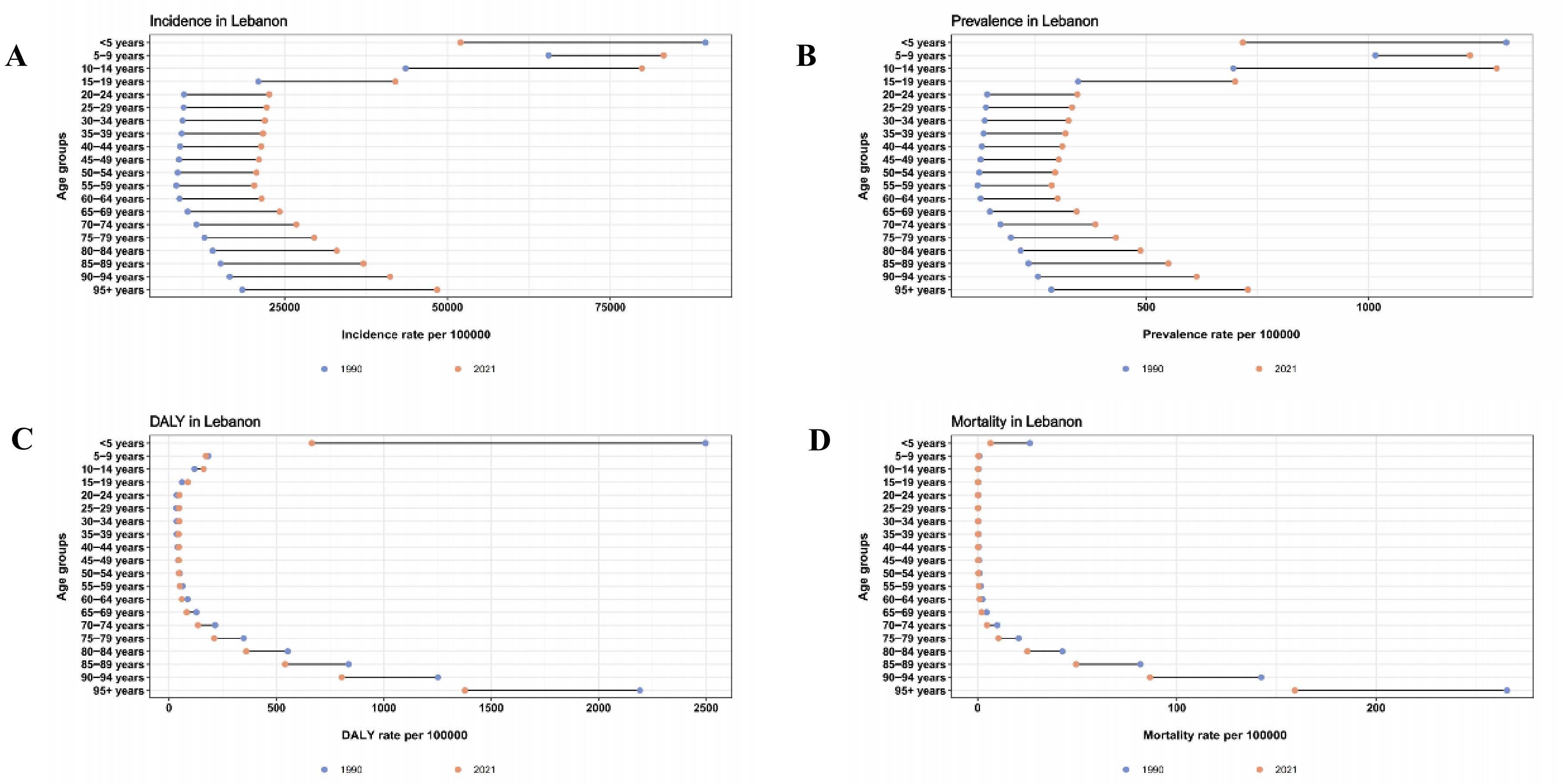

Figure S24: Age diversities of diarrheal diseases burden and time trends in Lebanon.

(A) Age diversities of incidence; (B) Age diversities of prevalence; (C) Age diversities of DALY rate; (D) Age diversities of mortality.

DALY, disability-adjusted life year.

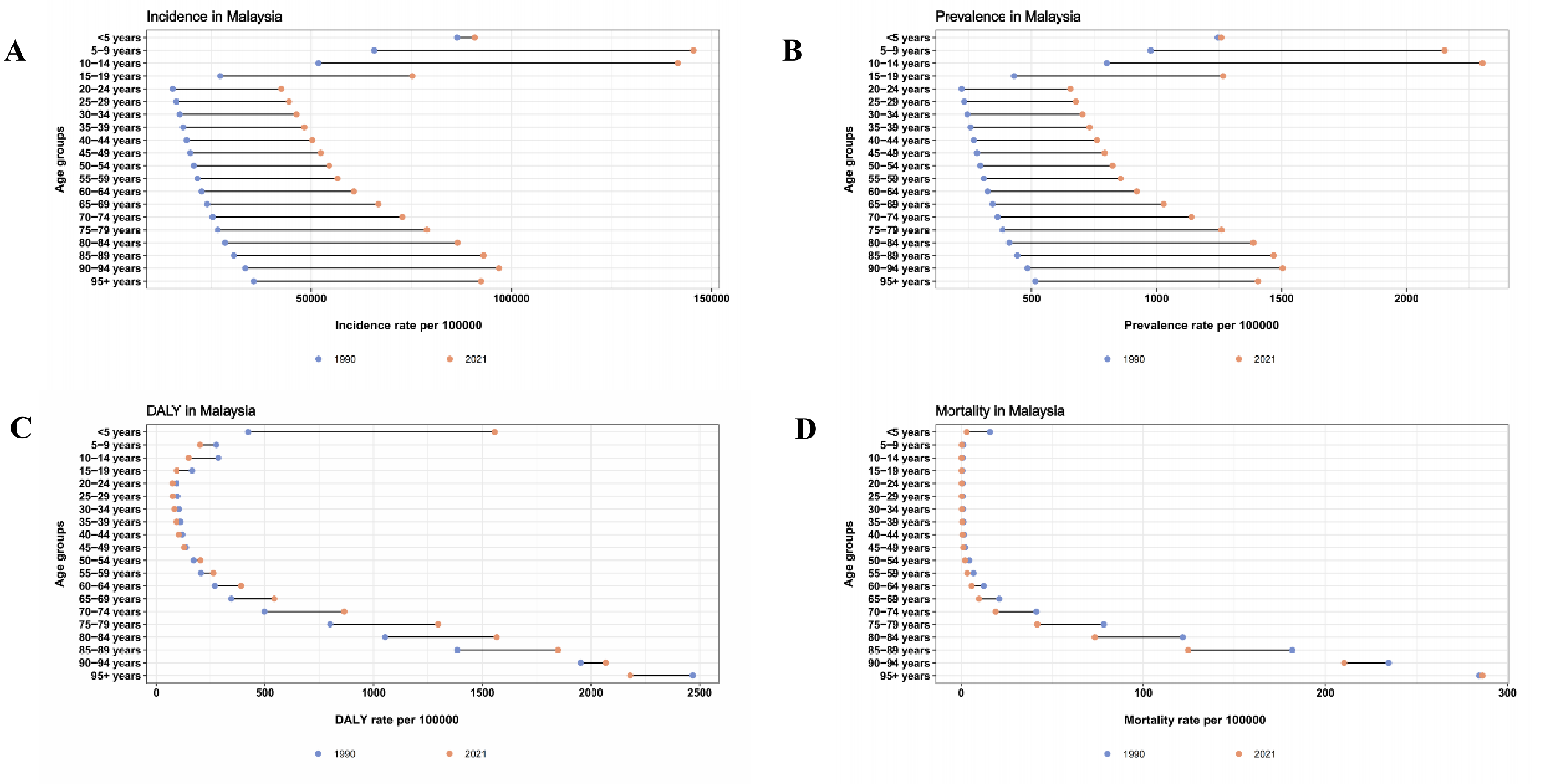

Figure S25: Age diversities of diarrheal diseases burden and time trends in Malaysia.

(A) Age diversities of incidence; (B) Age diversities of prevalence; (C) Age diversities of DALY rate; (D) Age diversities of mortality.

DALY, disability-adjusted life year.

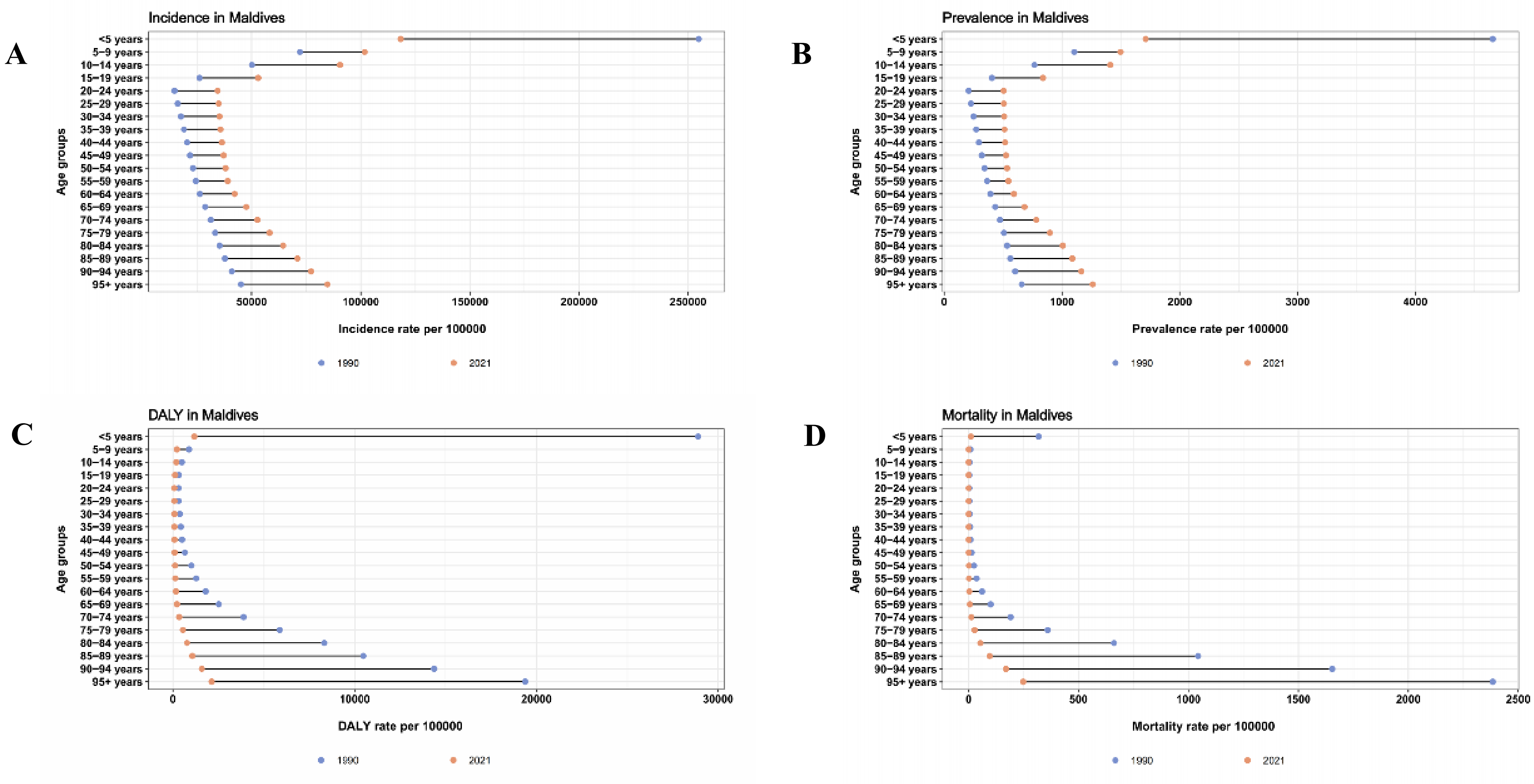

Figure S26: Age diversities of diarrheal diseases burden and time trends in Maldives.

(A) Age diversities of incidence; (B) Age diversities of prevalence; (C) Age diversities of DALY rate; (D) Age diversities of mortality.

DALY, disability-adjusted life year.

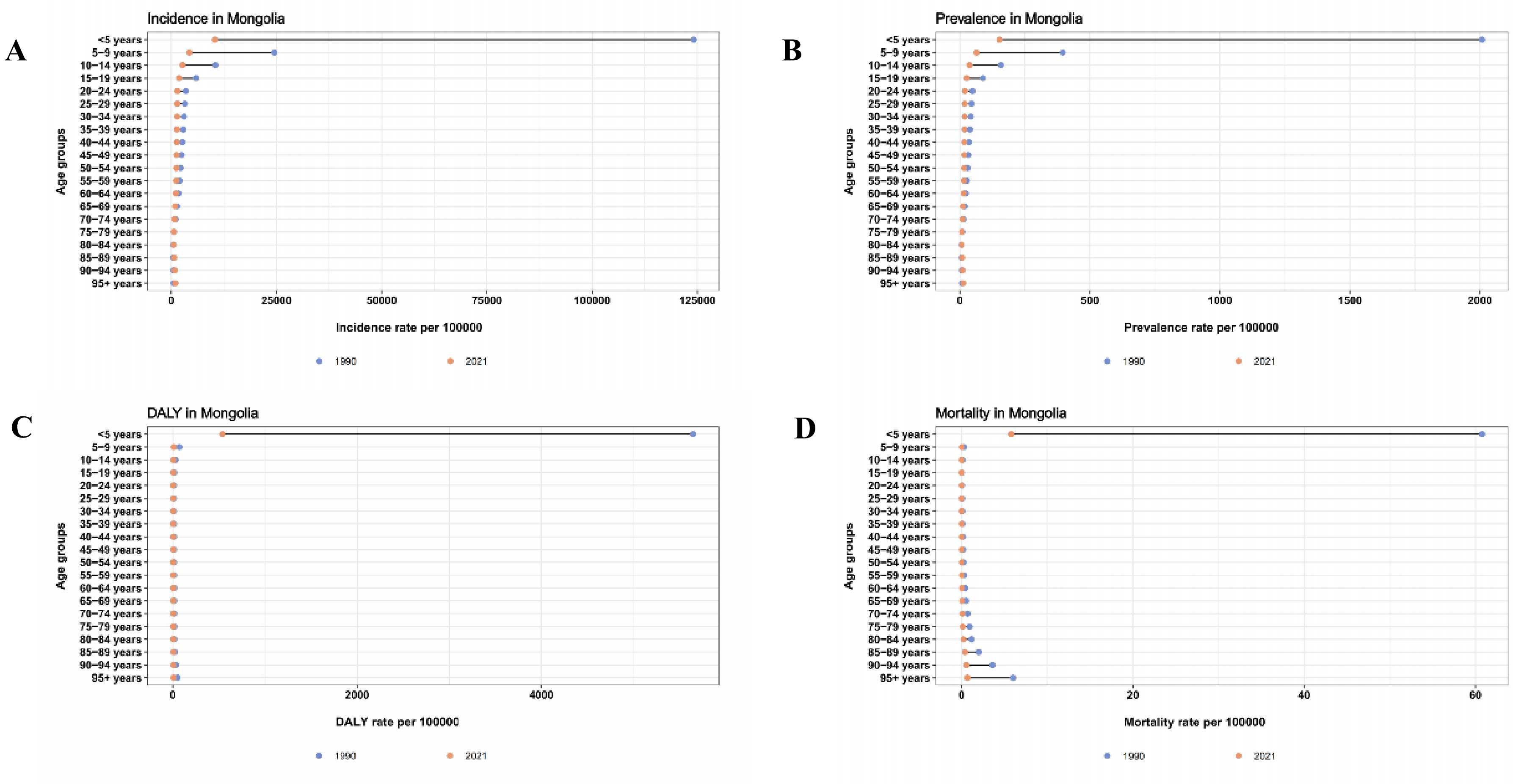

Figure S27: Age diversities of diarrheal diseases burden and time trends in Mongolia.

(A) Age diversities of incidence; (B) Age diversities of prevalence; (C) Age diversities of DALY rate; (D) Age diversities of mortality.

DALY, disability-adjusted life year.

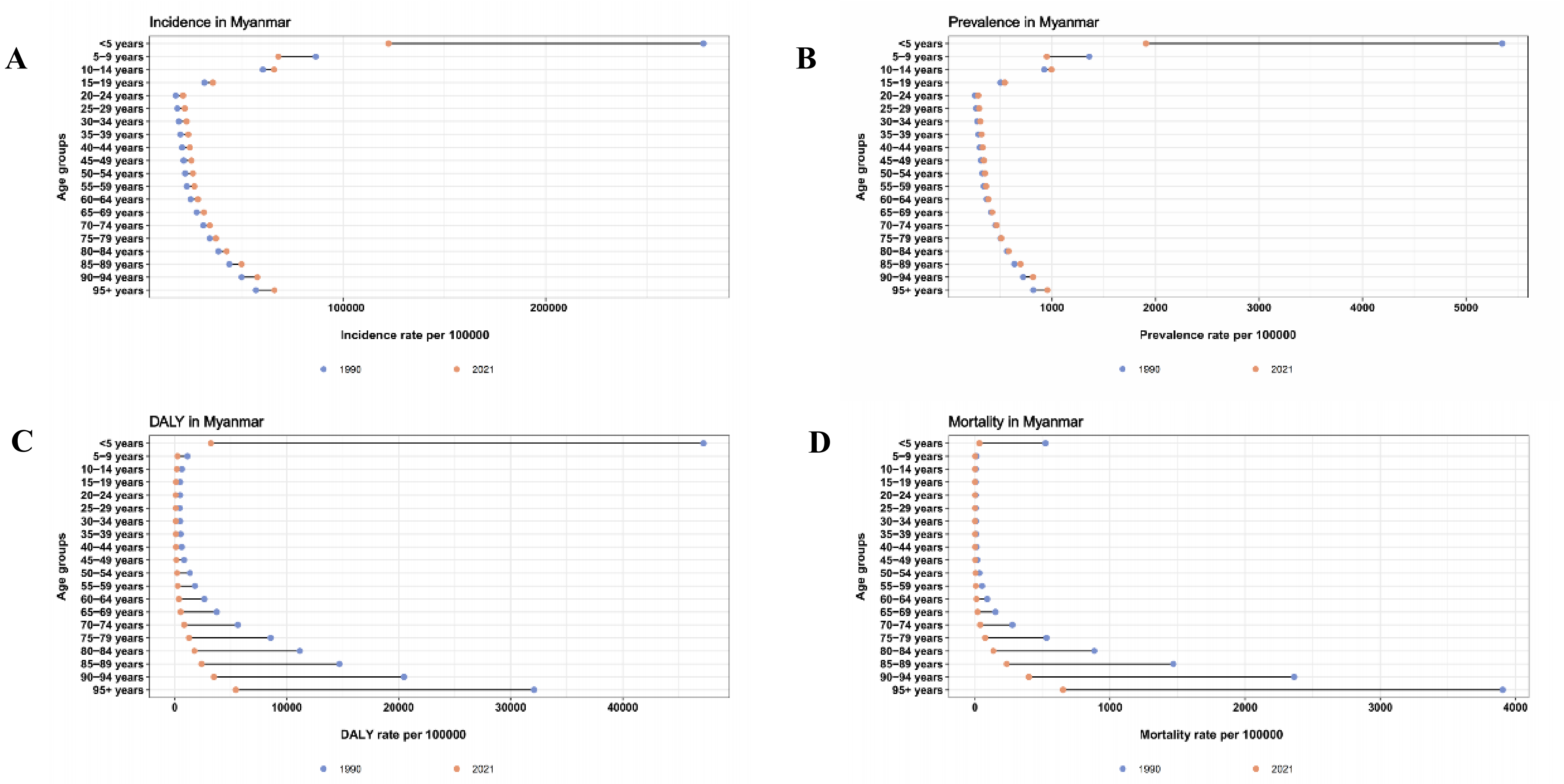

Figure S28: Age diversities of diarrheal diseases burden and time trends in Myanmar.

(A) Age diversities of incidence; (B) Age diversities of prevalence; (C) Age diversities of DALY rate; (D) Age diversities of mortality.

DALY, disability-adjusted life year.

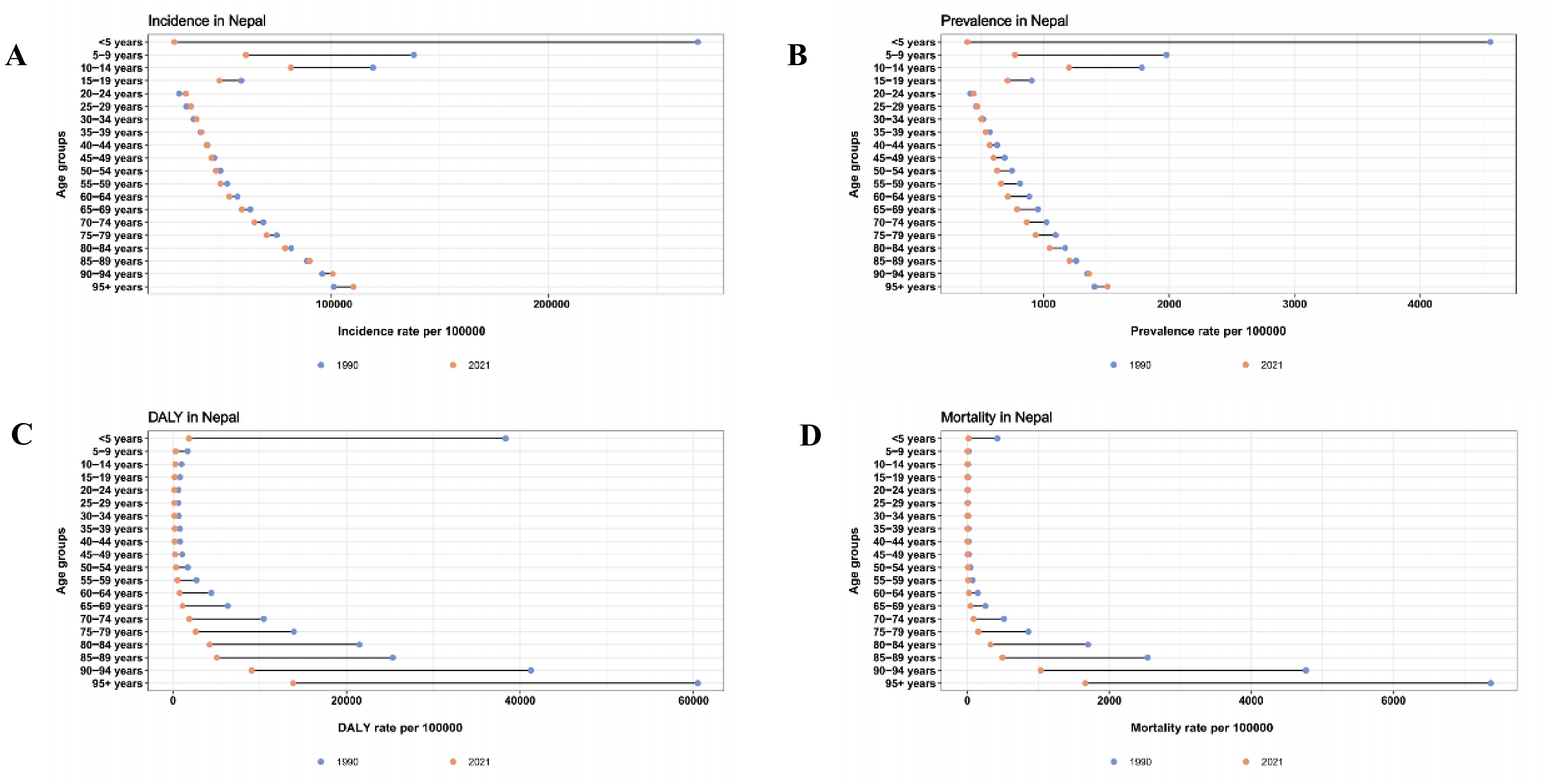

Figure S29: Age diversities of diarrheal diseases burden and time trends in Nepal.

(A) Age diversities of incidence; (B) Age diversities of prevalence; (C) Age diversities of DALY rate; (D) Age diversities of mortality.

DALY, disability-adjusted life year.

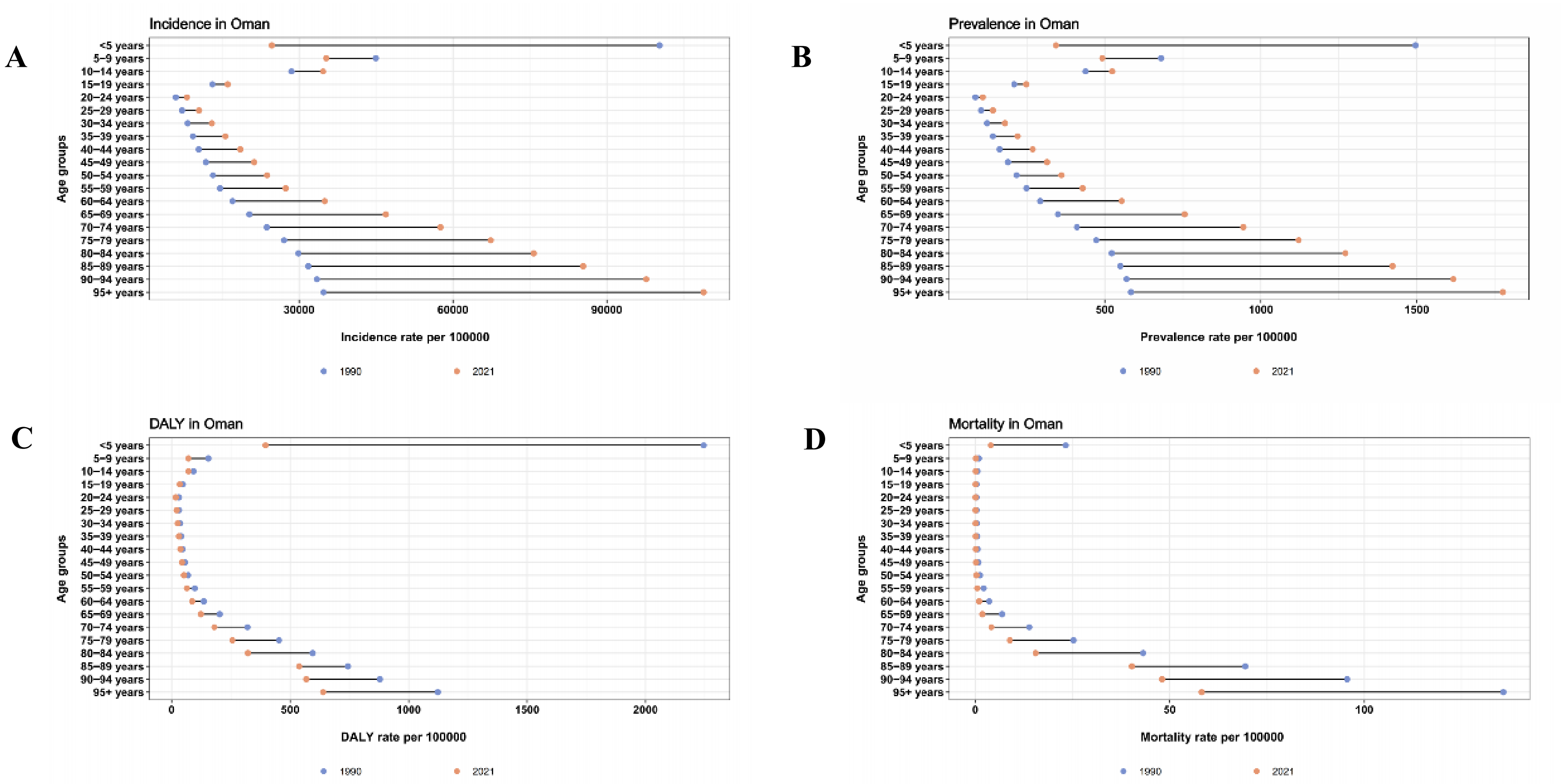

Figure S30: Age diversities of diarrheal diseases burden and time trends in Oman.

(A) Age diversities of incidence; (B) Age diversities of prevalence; (C) Age diversities of DALY rate; (D) Age diversities of mortality.

DALY, disability-adjusted life year.

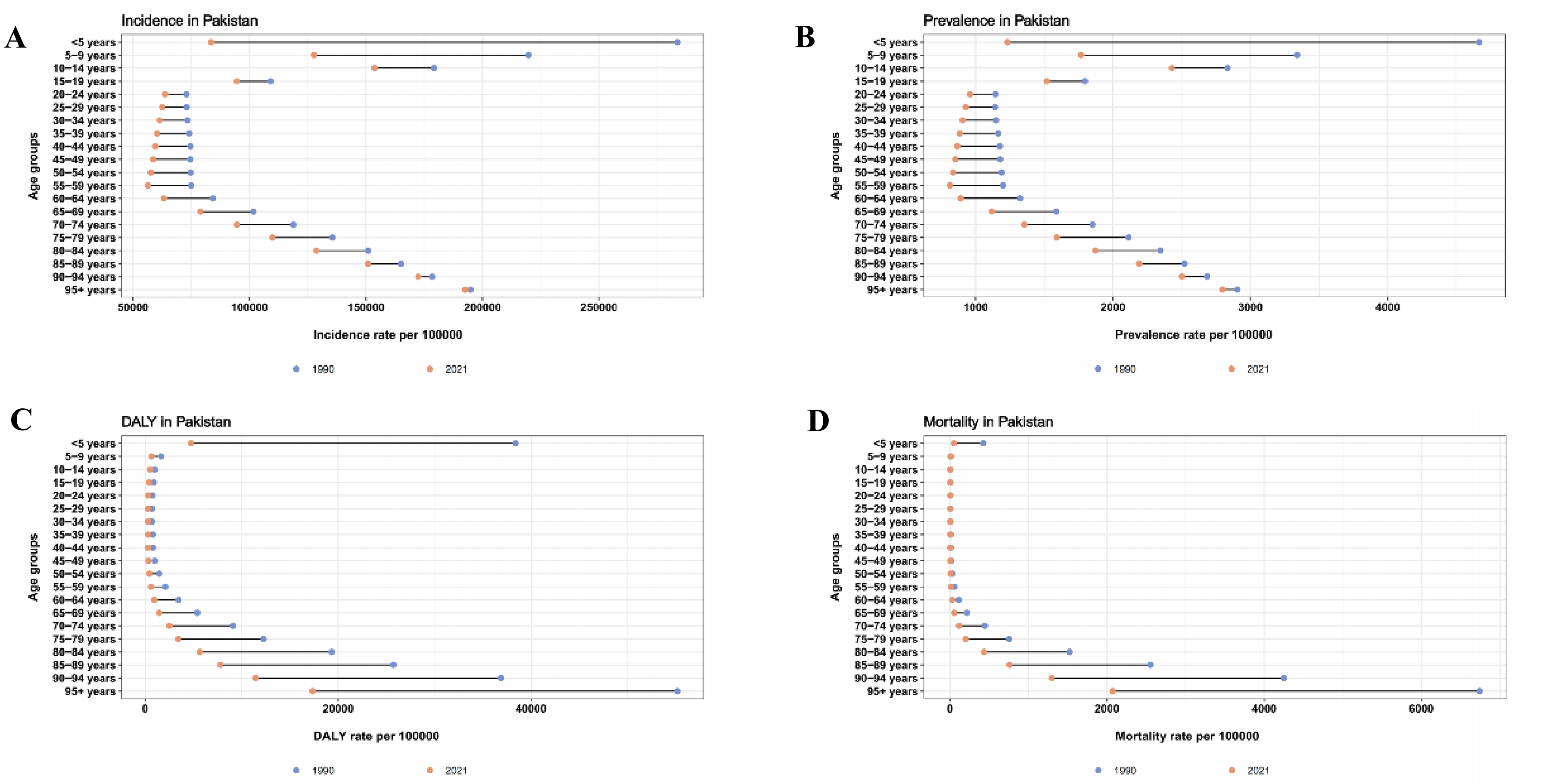

Figure S31: Age diversities of diarrheal diseases burden and time trends in Pakistan.

(A) Age diversities of incidence; (B) Age diversities of prevalence; (C) Age diversities of DALY rate; (D) Age diversities of mortality.

DALY, disability-adjusted life year.

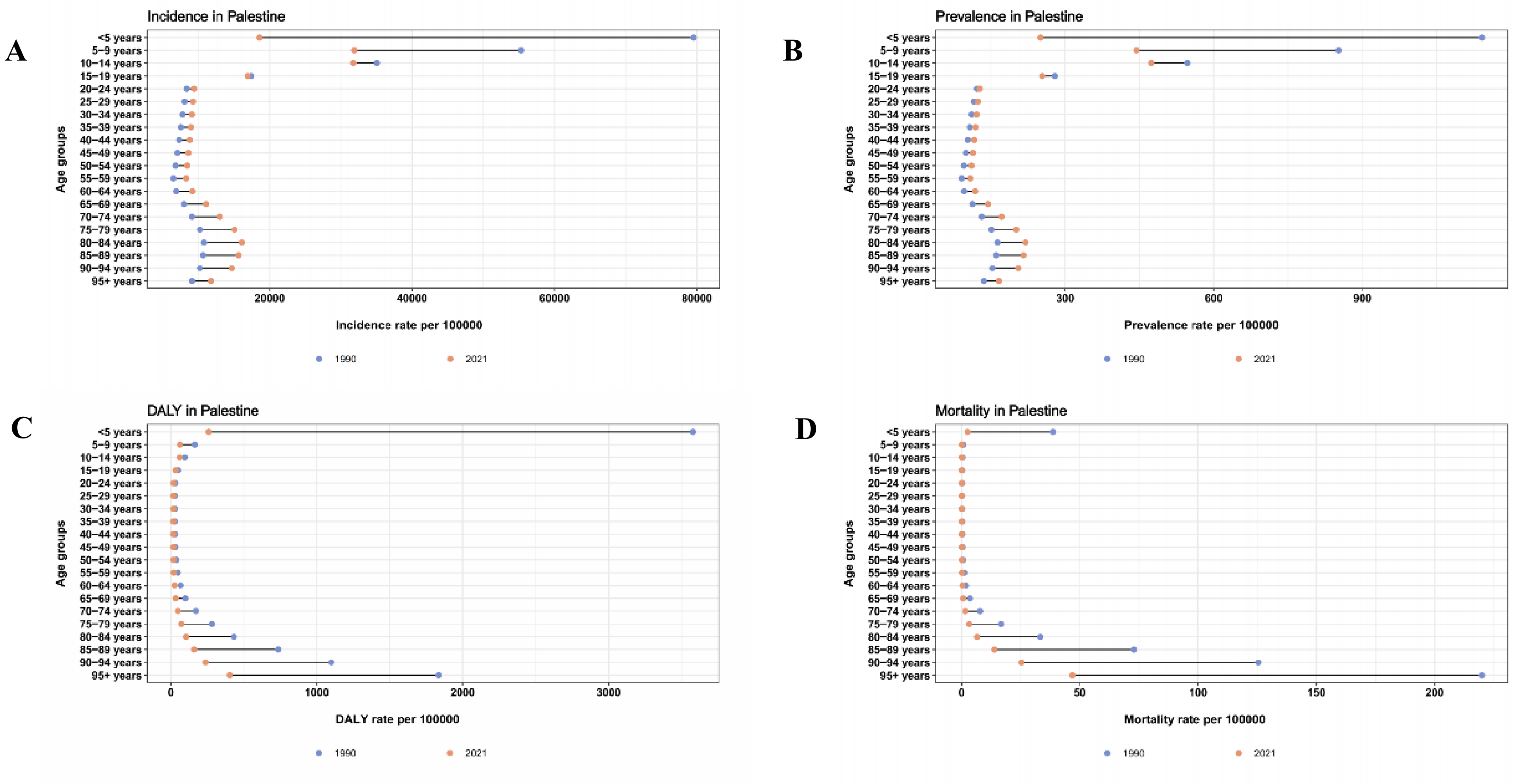

Figure S32: Age diversities of diarrheal diseases burden and time trends in Palestine.

(A) Age diversities of incidence; (B) Age diversities of prevalence; (C) Age diversities of DALY rate; (D) Age diversities of mortality.

DALY, disability-adjusted life year.

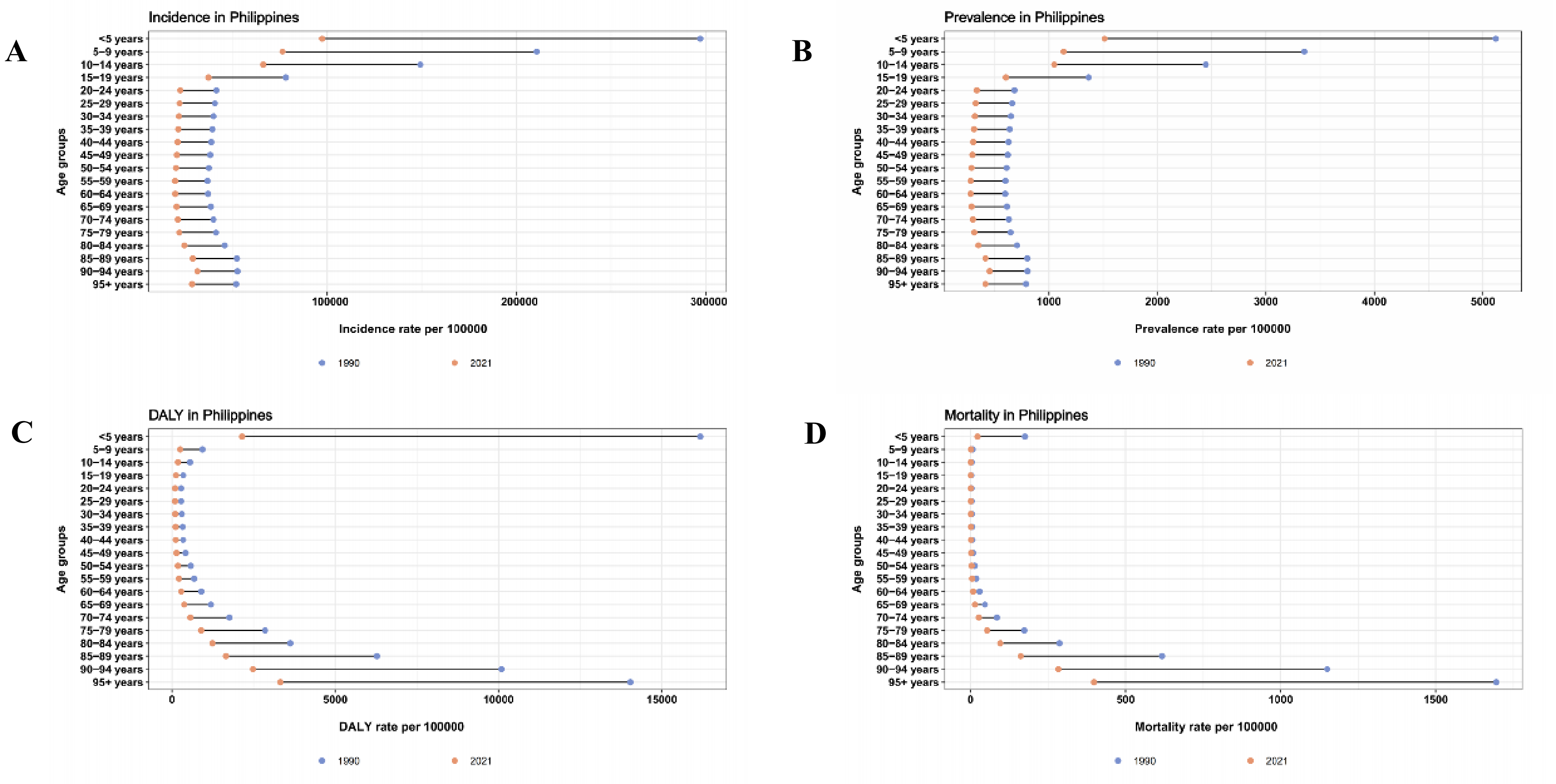

Figure S33: Age diversities of diarrheal diseases burden and time trends in Philippines.

(A) Age diversities of incidence; (B) Age diversities of prevalence; (C) Age diversities of DALY rate; (D) Age diversities of mortality.

DALY, disability-adjusted life year.

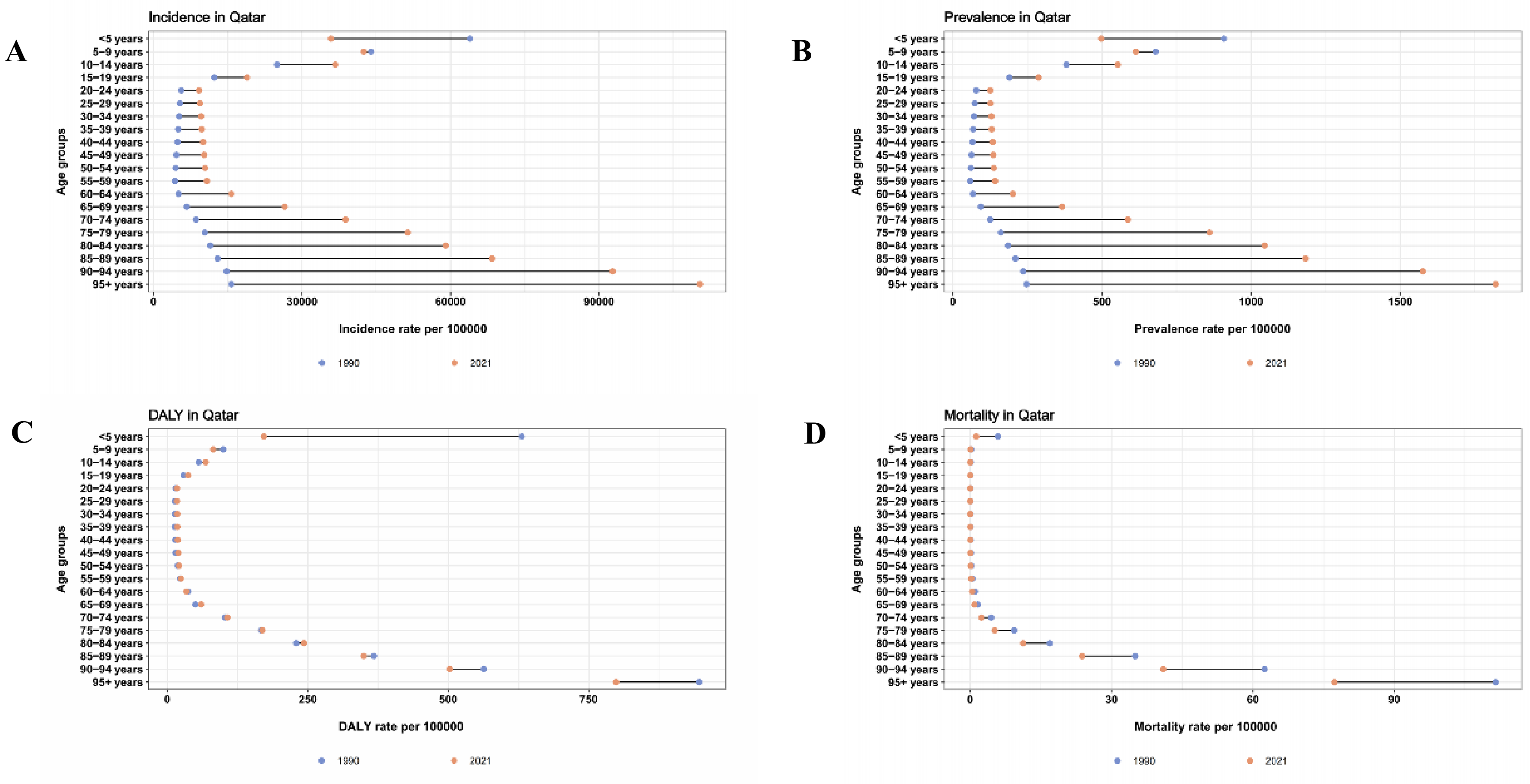

Figure S34: Age diversities of diarrheal diseases burden and time trends in Qatar.

(A) Age diversities of incidence; (B) Age diversities of prevalence; (C) Age diversities of DALY rate; (D) Age diversities of mortality.

DALY, disability-adjusted life year.

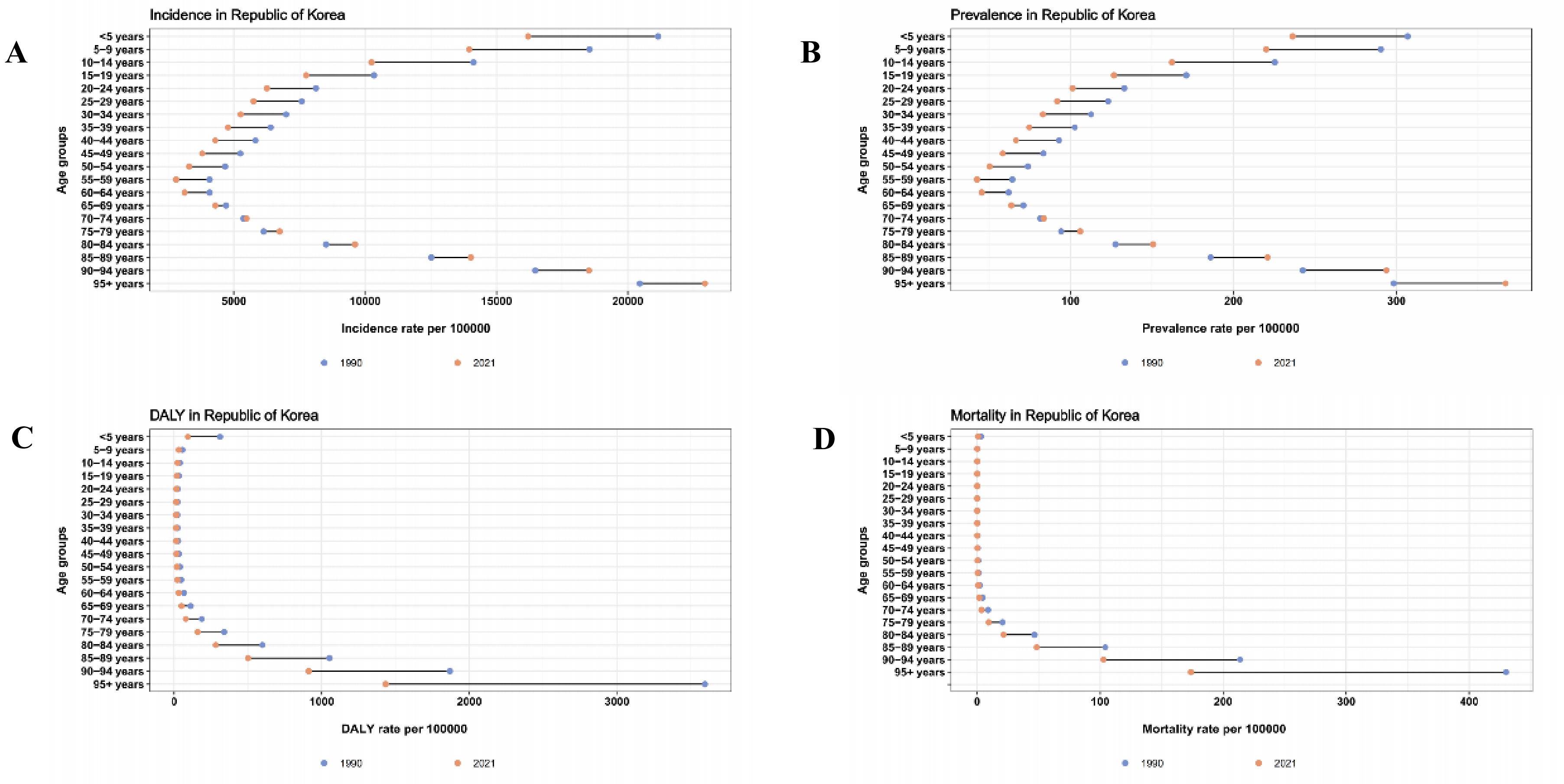

Figure S35: Age diversities of diarrheal diseases burden and time trends in Republic of Korea.

(A) Age diversities of incidence; (B) Age diversities of prevalence; (C) Age diversities of DALY rate; (D) Age diversities of mortality.

DALY, disability-adjusted life year.

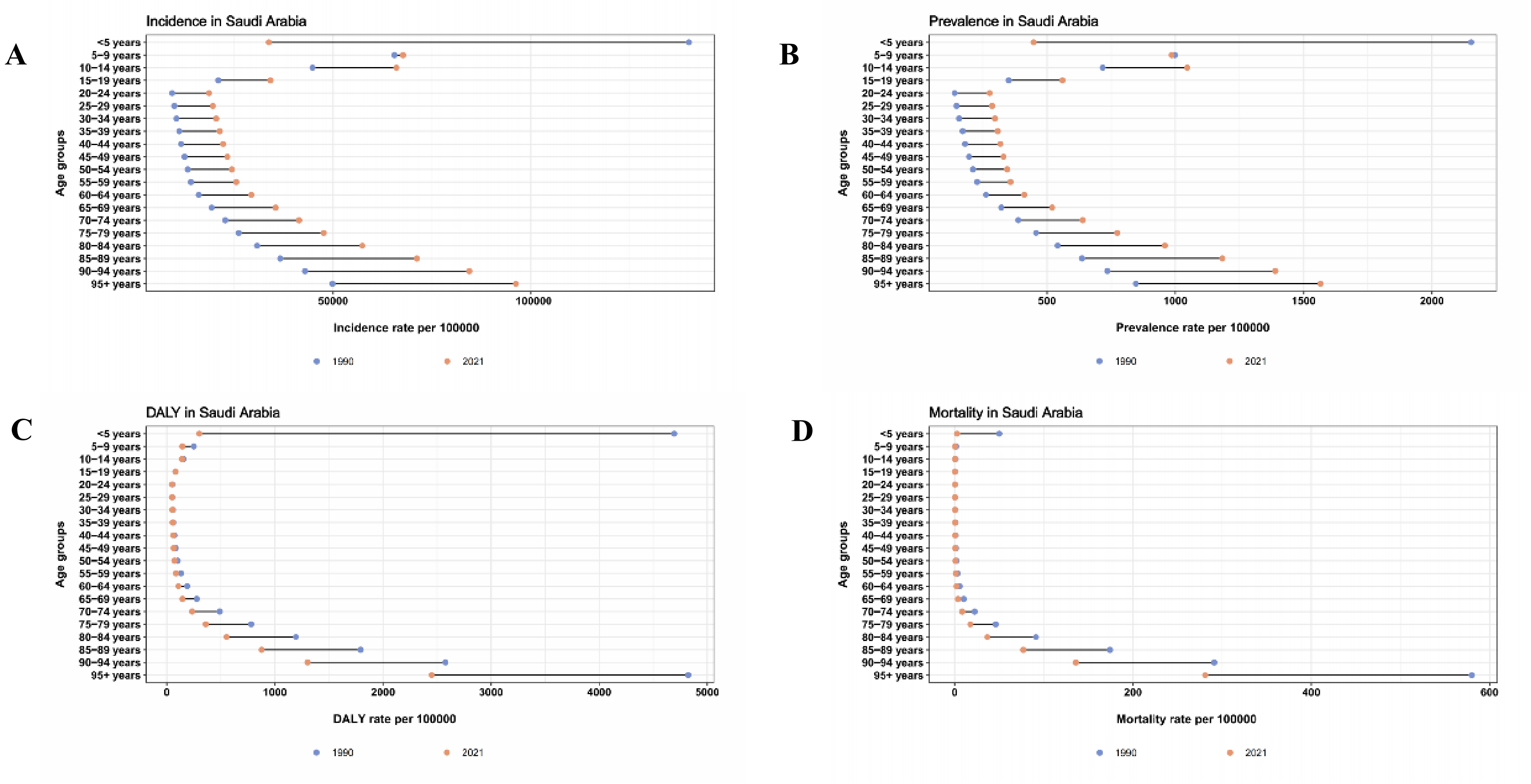

Figure S36: Age diversities of diarrheal diseases burden and time trends in Saudi Arabia.

(A) Age diversities of incidence; (B) Age diversities of prevalence; (C) Age diversities of DALY rate; (D) Age diversities of mortality.

DALY, disability-adjusted life year.

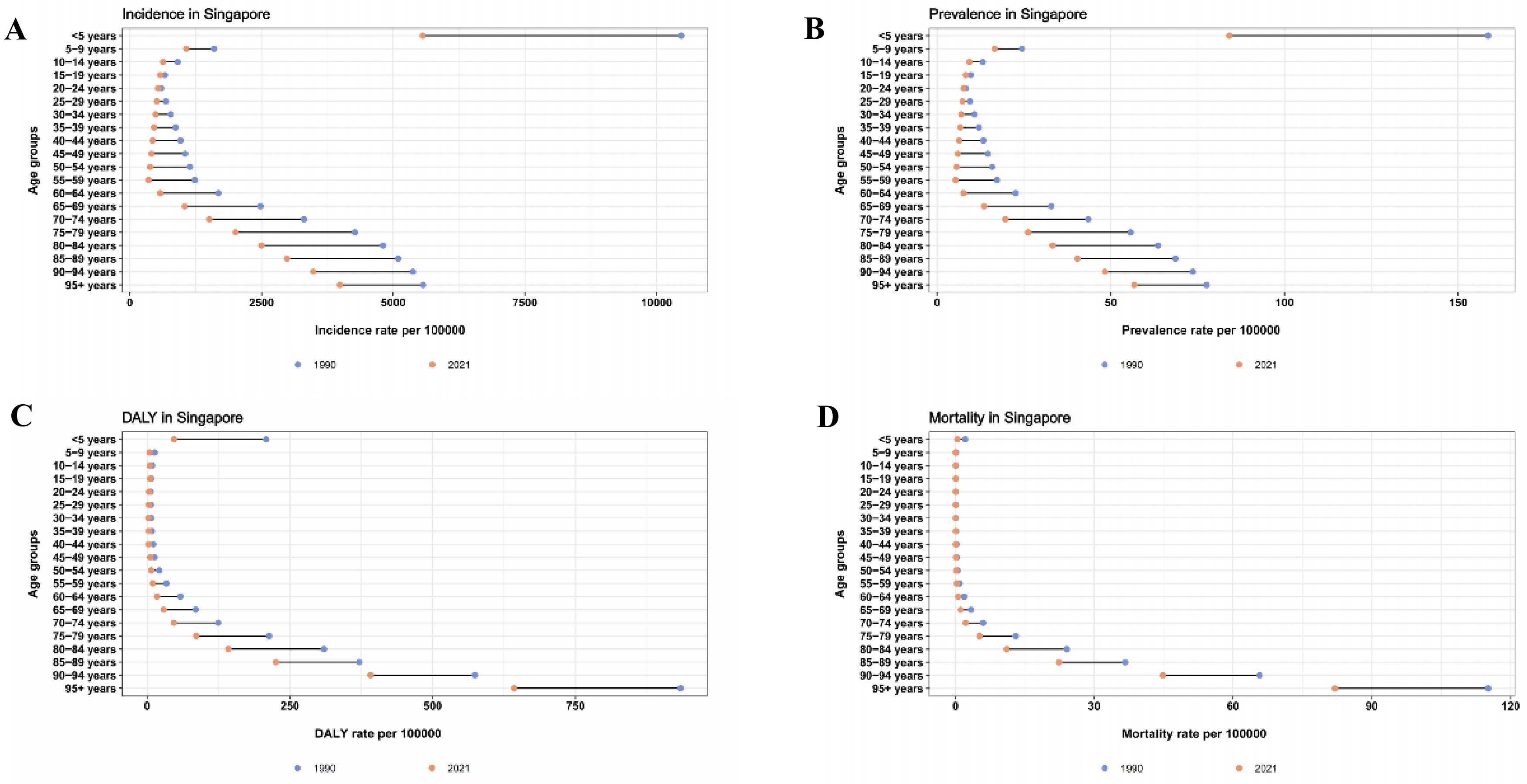

Figure S37: Age diversities of diarrheal diseases burden and time trends in Singapore.
(A) Age diversities of incidence; (B) Age diversities of prevalence; (C) Age diversities of DALY rate; (D) Age diversities of mortality.
DALY, disability-adjusted life year.

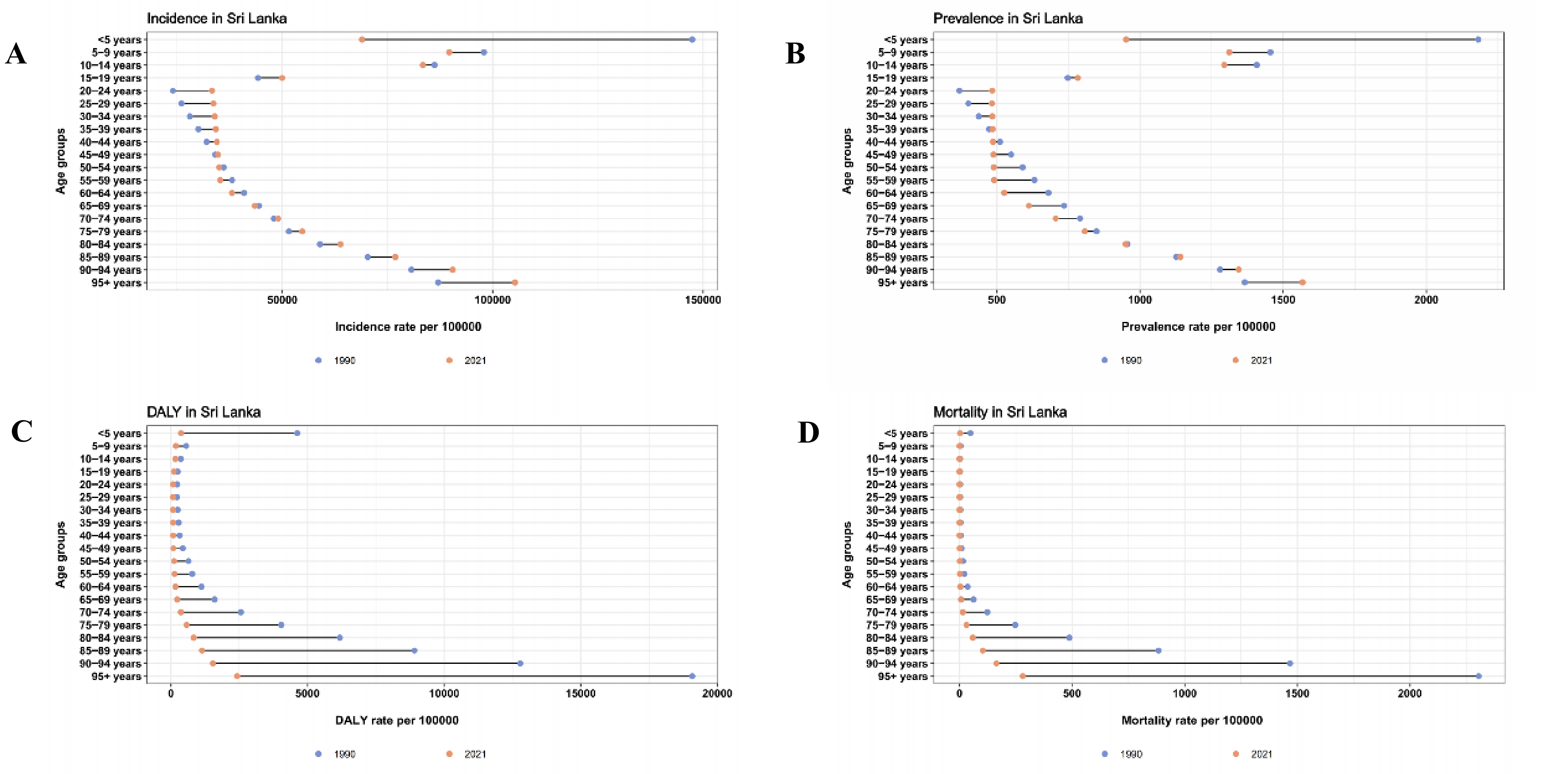

Figure S38: Age diversities of diarrheal diseases burden and time trends in Sri Lanka.

(A) Age diversities of incidence; (B) Age diversities of prevalence; (C) Age diversities of DALY rate; (D) Age diversities of mortality.

DALY, disability-adjusted life year.

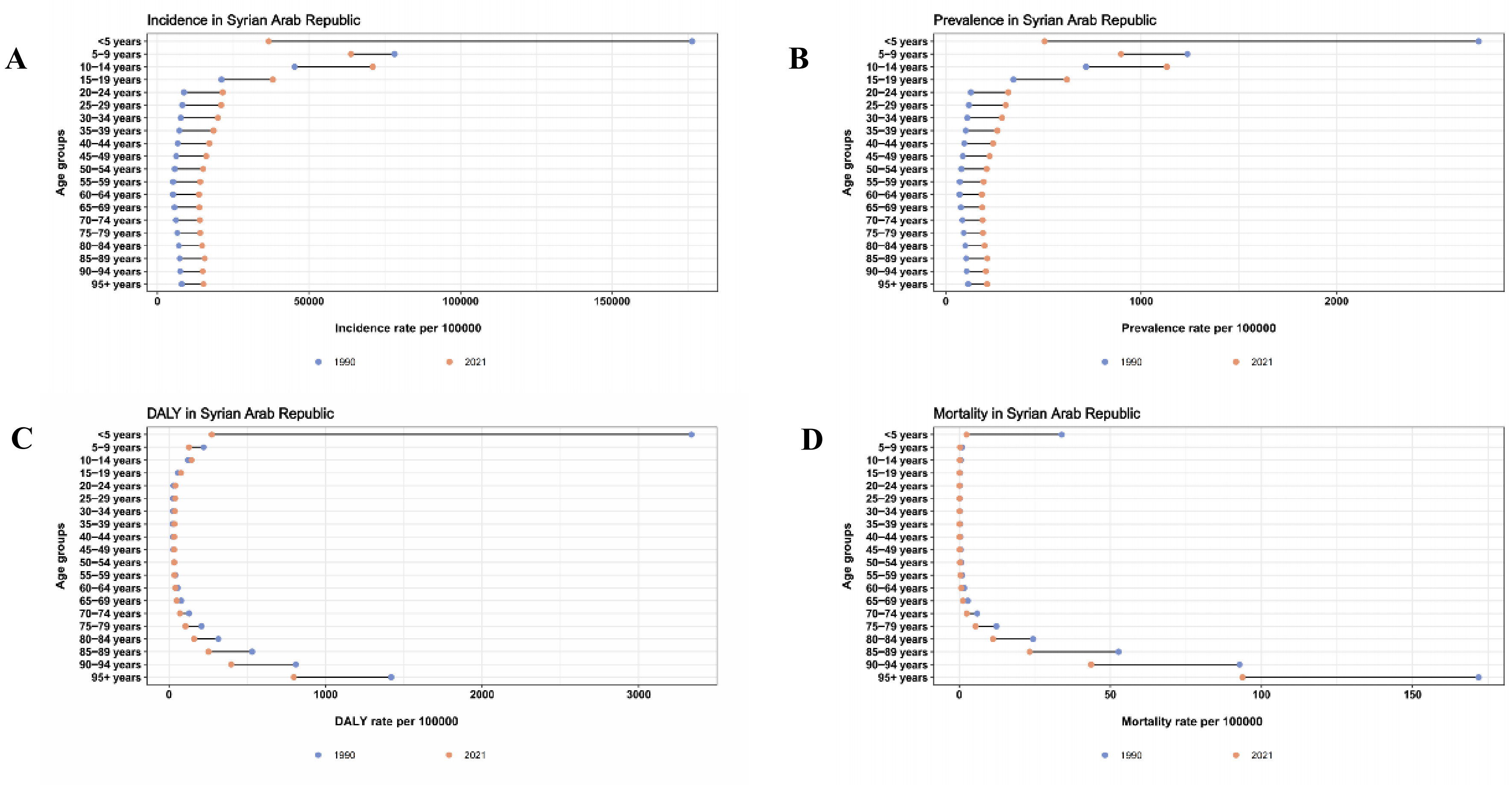

Figure S39: Age diversities of diarrheal diseases burden and time trends in Syrian Arab Republic.

(A) Age diversities of incidence; (B) Age diversities of prevalence; (C) Age diversities of DALY rate; (D) Age diversities of mortality.

DALY, disability-adjusted life year.

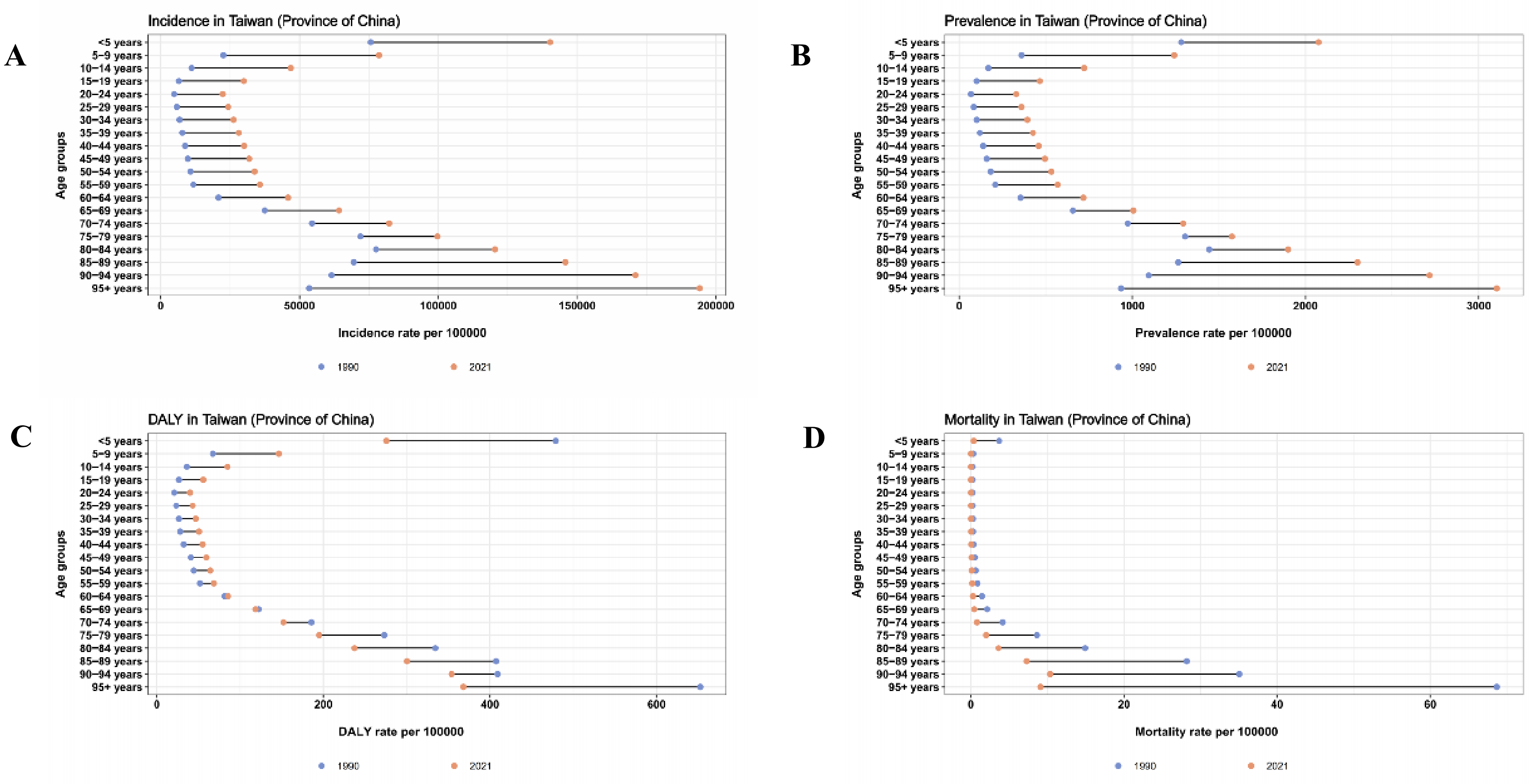

Figure S40: Age diversities of diarrheal diseases burden and time trends in Taiwan (Province of China).

(A) Age diversities of incidence; (B) Age diversities of prevalence; (C) Age diversities of DALY rate; (D) Age diversities of mortality.

DALY, disability-adjusted life year.

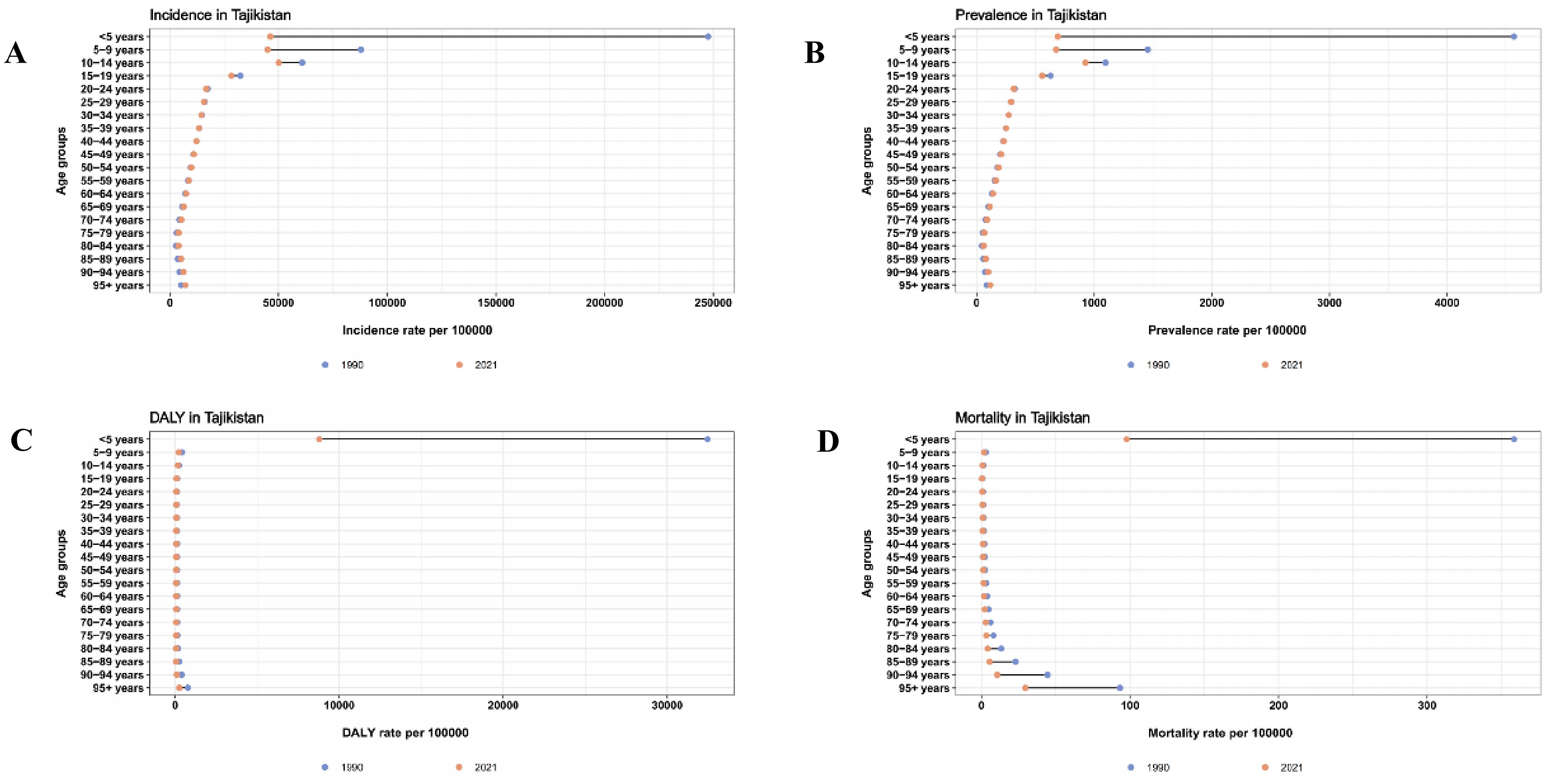

Figure S41: Age diversities of diarrheal diseases burden and time trends in Tajikistan.

(A) Age diversities of incidence; (B) Age diversities of prevalence; (C) Age diversities of DALY rate; (D) Age diversities of mortality.

DALY, disability-adjusted life year.

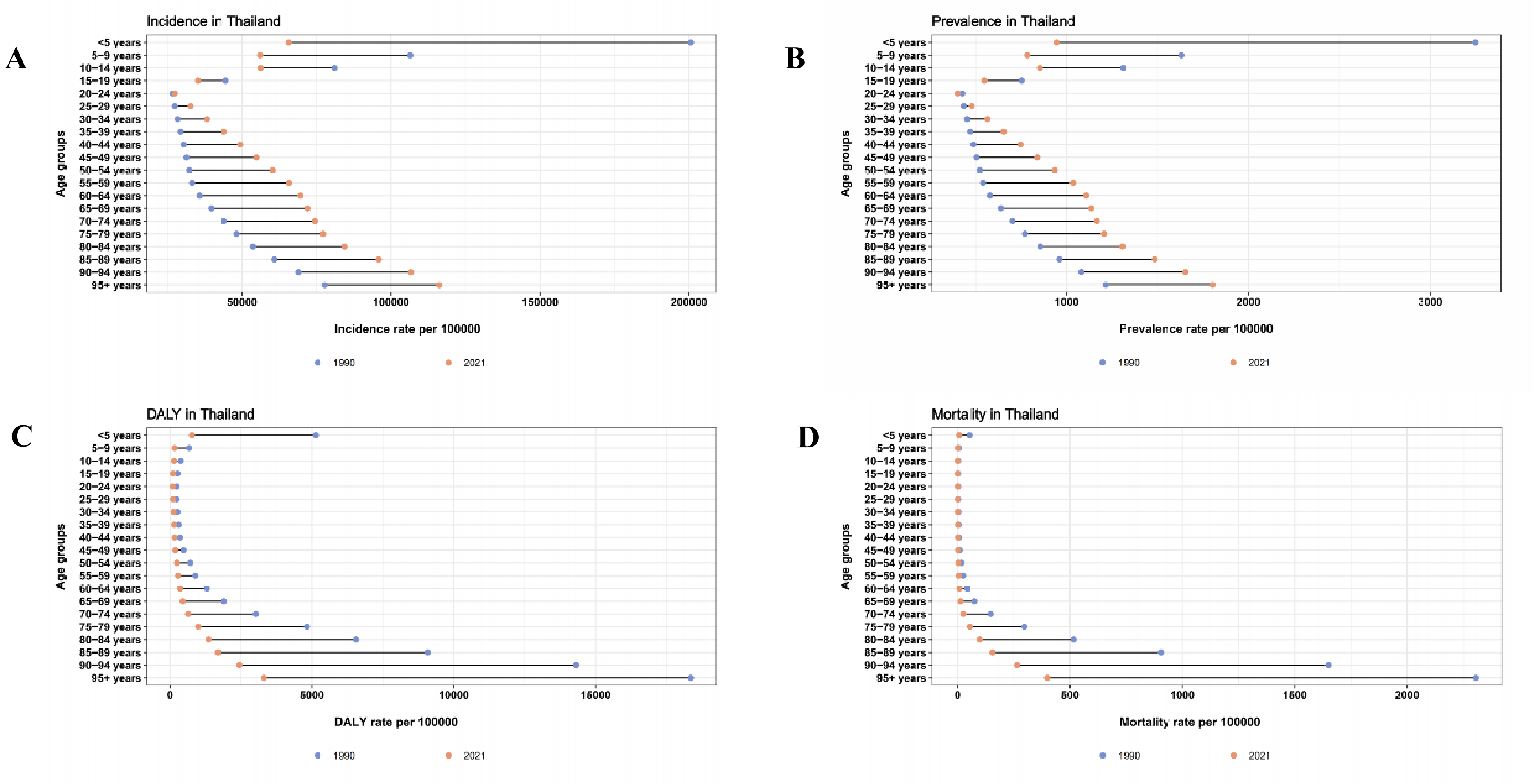

Figure S42: Age diversities of diarrheal diseases burden and time trends in Thailand.

(A) Age diversities of incidence; (B) Age diversities of prevalence; (C) Age diversities of DALY rate; (D) Age diversities of mortality.

DALY, disability-adjusted life year.

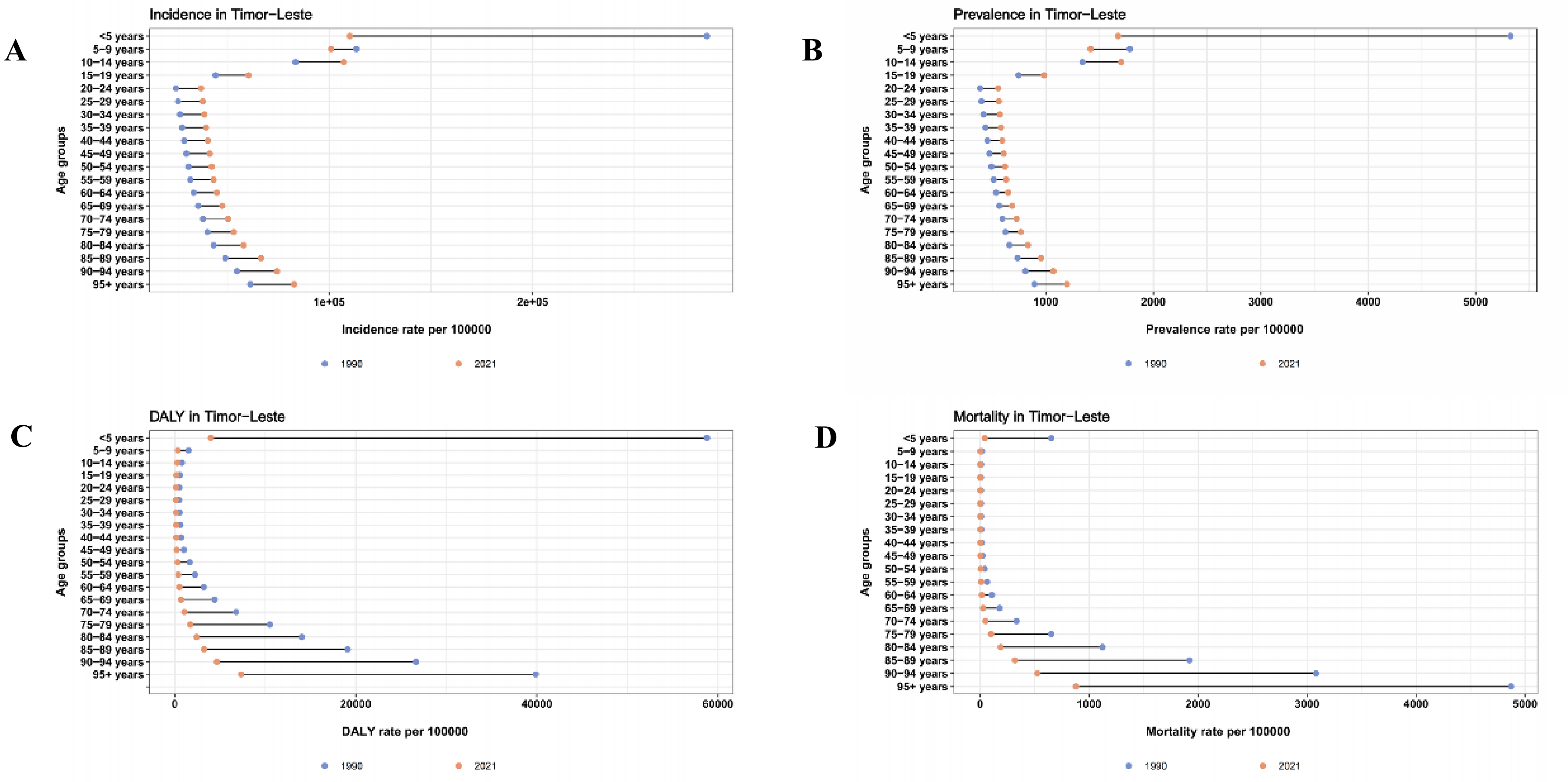

Figure S43: Age diversities of diarrheal diseases burden and time trends in Timor-Leste.

(A) Age diversities of incidence; (B) Age diversities of prevalence; (C) Age diversities of DALY rate; (D) Age diversities of mortality.

DALY, disability-adjusted life year.

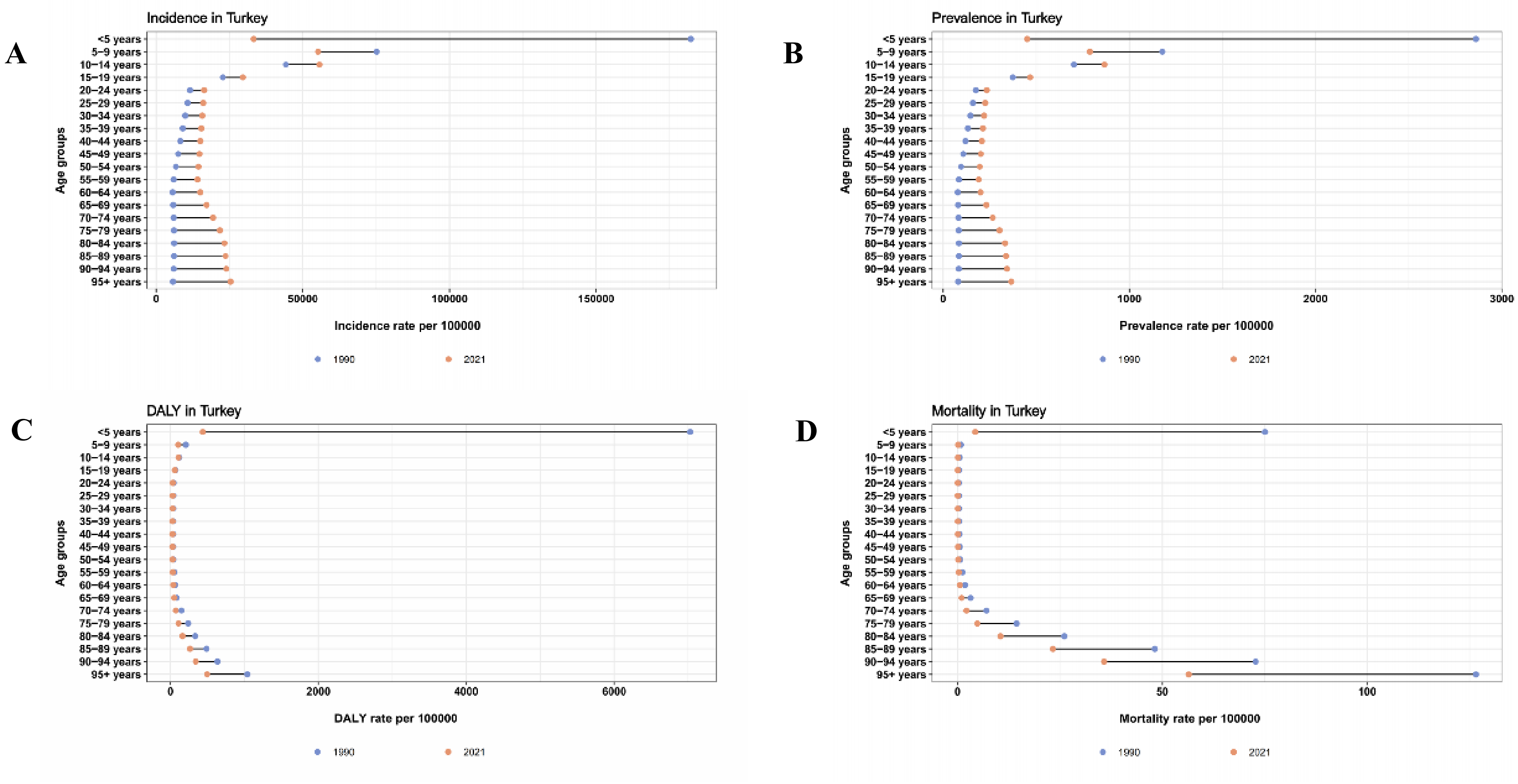

Figure S44: Age diversities of diarrheal diseases burden and time trends in Turkey.

(A) Age diversities of incidence; (B) Age diversities of prevalence; (C) Age diversities of DALY rate; (D) Age diversities of mortality.

DALY, disability-adjusted life year.

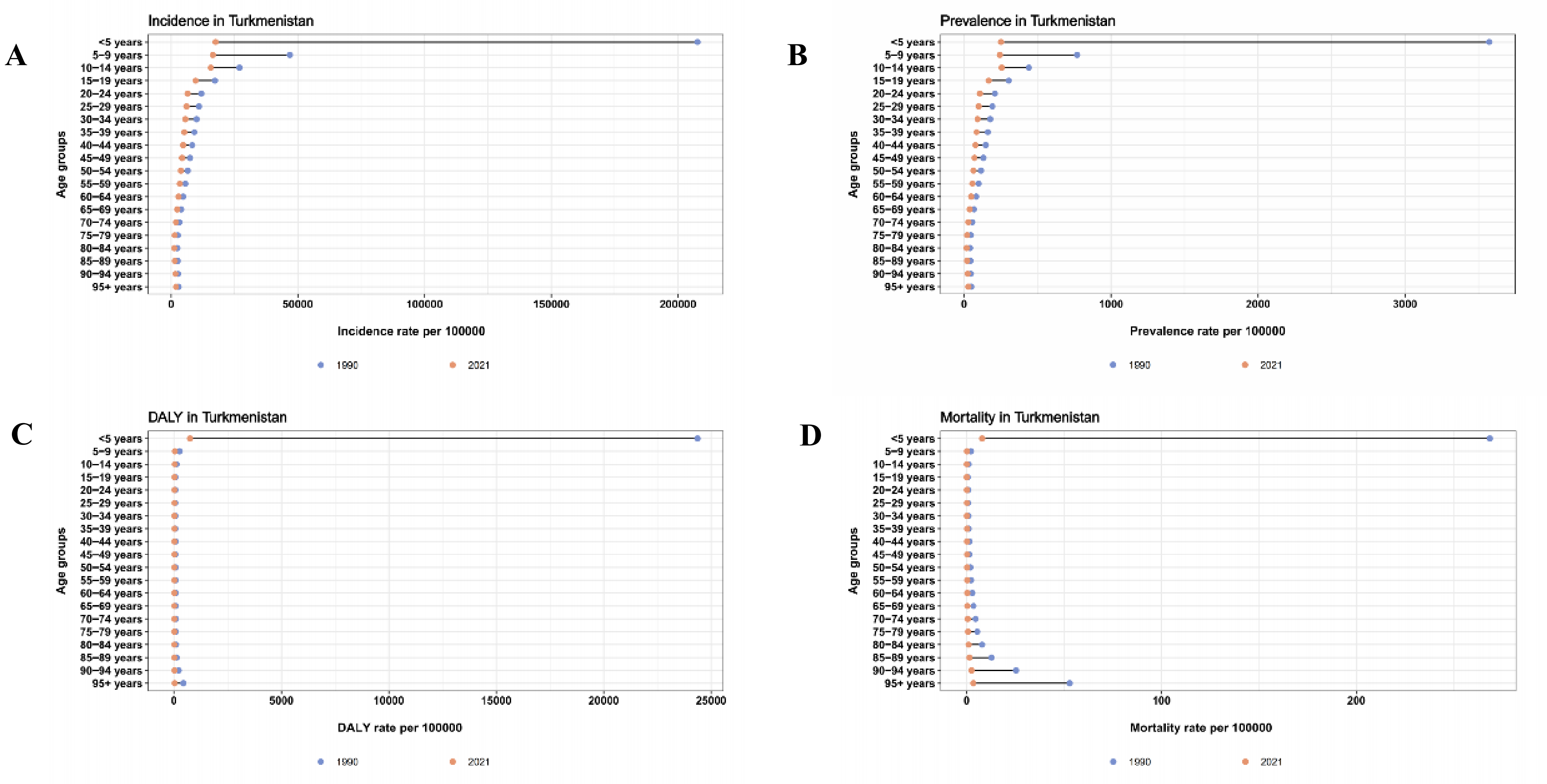

Figure S45: Age diversities of diarrheal diseases burden and time trends in Turkmenistan.

(A) Age diversities of incidence; (B) Age diversities of prevalence; (C) Age diversities of DALY rate; (D) Age diversities of mortality.

DALY, disability-adjusted life year.

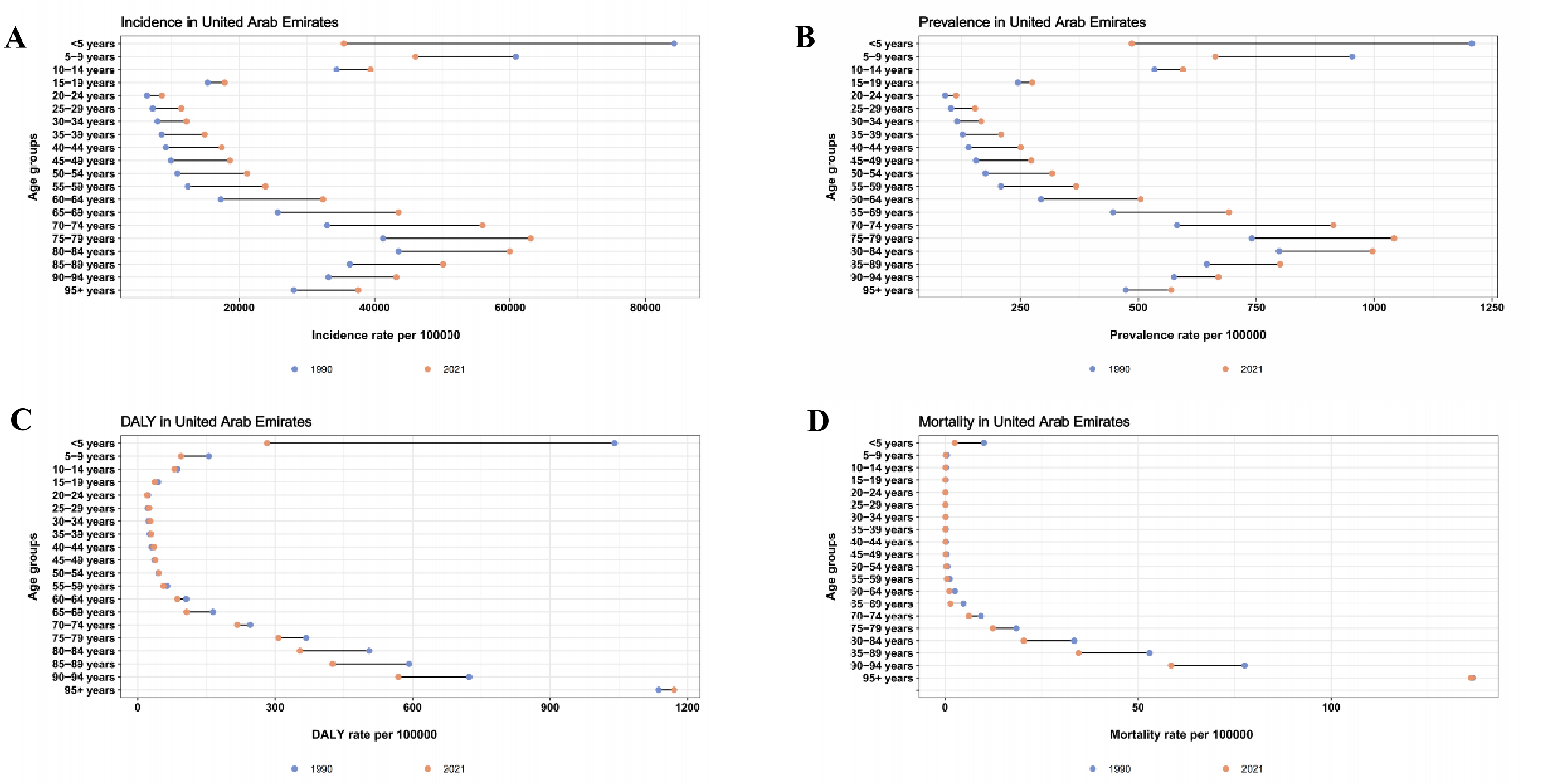

Figure S46: Age diversities of diarrheal diseases burden and time trends in United Arab Emirates.

(A) Age diversities of incidence; (B) Age diversities of prevalence; (C) Age diversities of DALY rate; (D) Age diversities of mortality.

DALY, disability-adjusted life year.

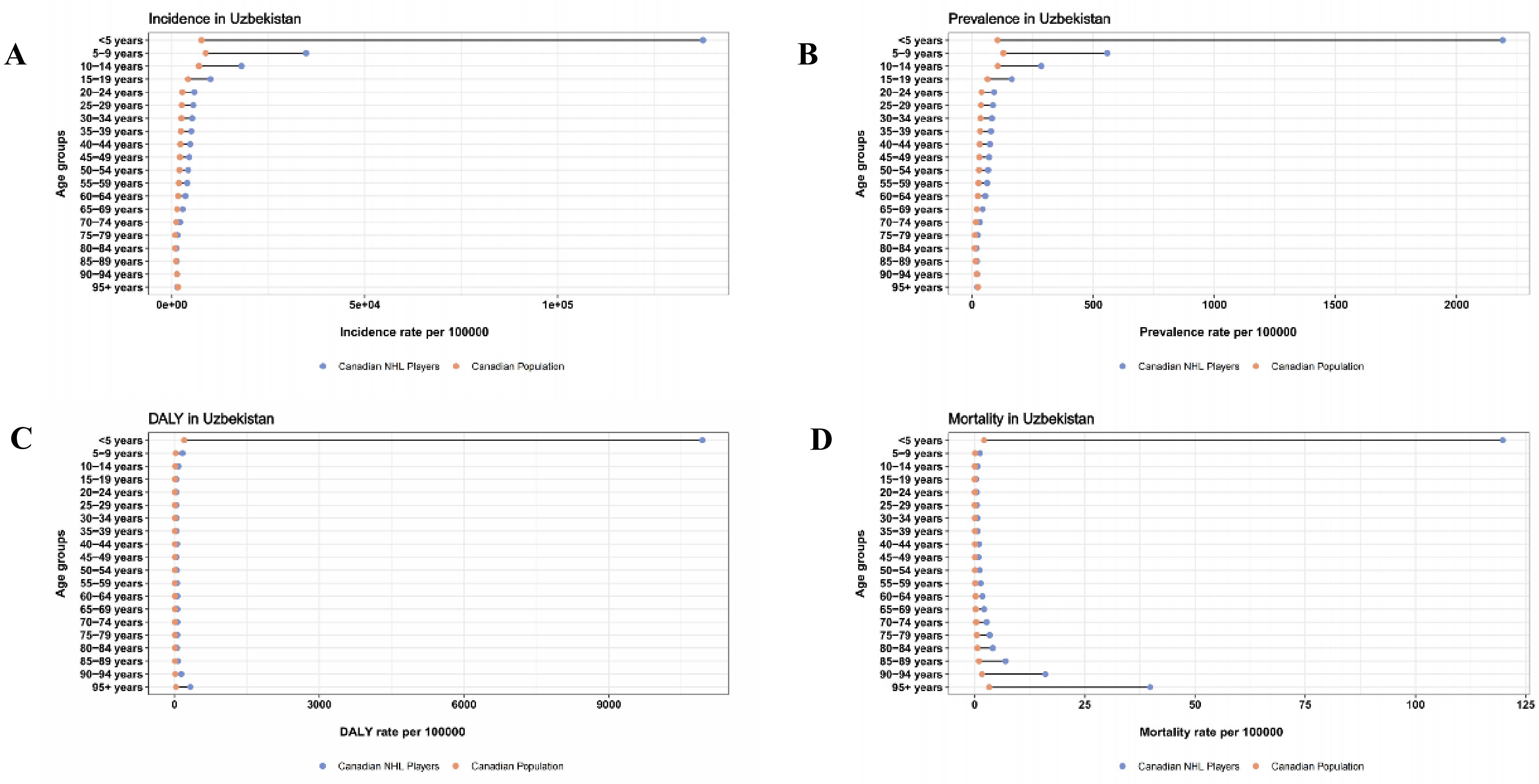

Figure S47: Age diversities of diarrheal diseases burden and time trends in Uzbekistan.

(A) Age diversities of incidence; (B) Age diversities of prevalence; (C) Age diversities of DALY rate; (D) Age diversities of mortality.

DALY, disability-adjusted life year.

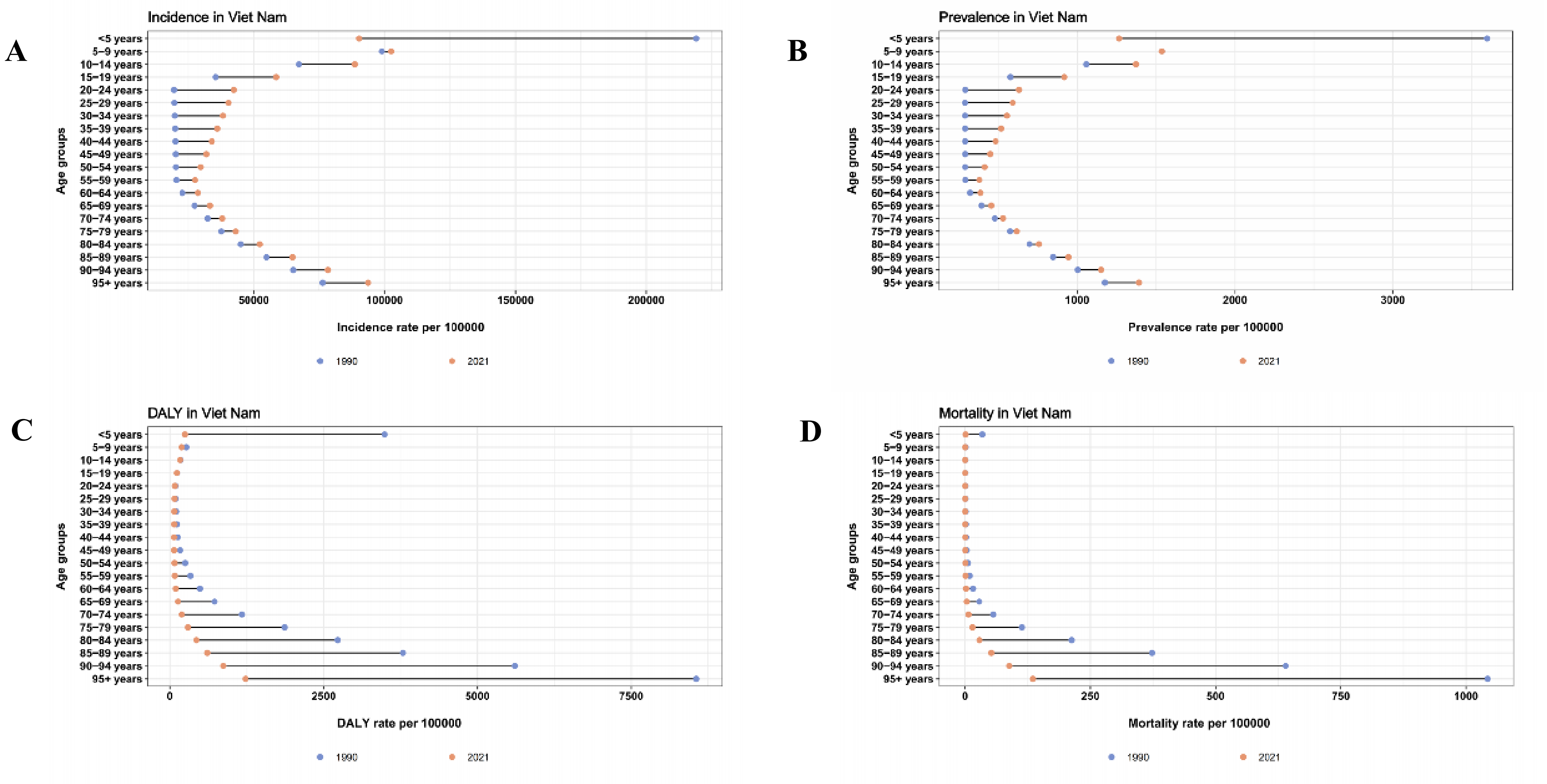

Figure S48: Age diversities of diarrheal diseases burden and time trends in Viet Nam.

(A) Age diversities of incidence; (B) Age diversities of prevalence; (C) Age diversities of DALY rate; (D) Age diversities of mortality.

DALY, disability-adjusted life year.

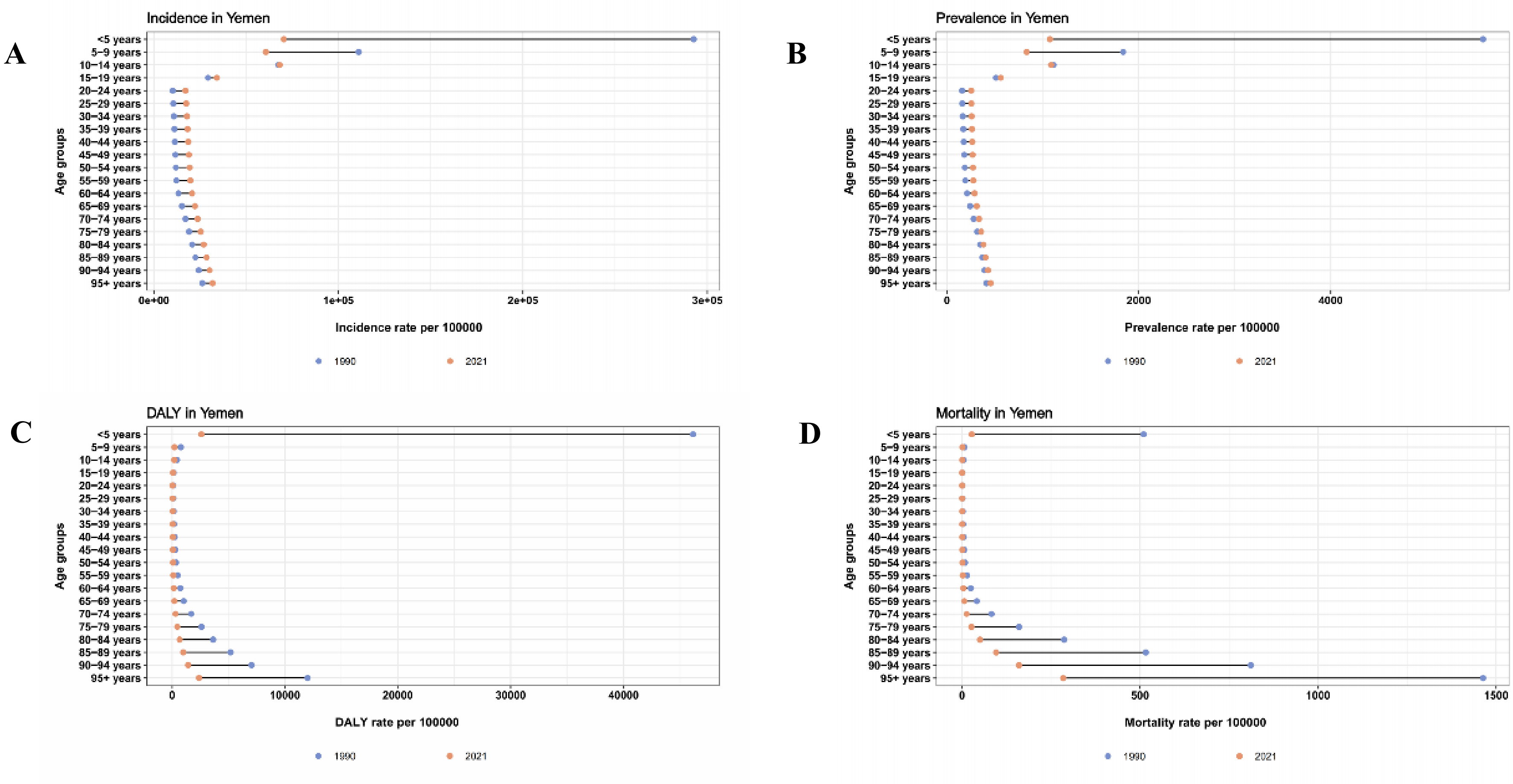

Figure S49: Age diversities of diarrheal diseases burden and time trends in Yemen.

(A) Age diversities of incidence; (B) Age diversities of prevalence; (C) Age diversities of DALY rate; (D) Age diversities of mortality.

DALY, disability-adjusted life year.

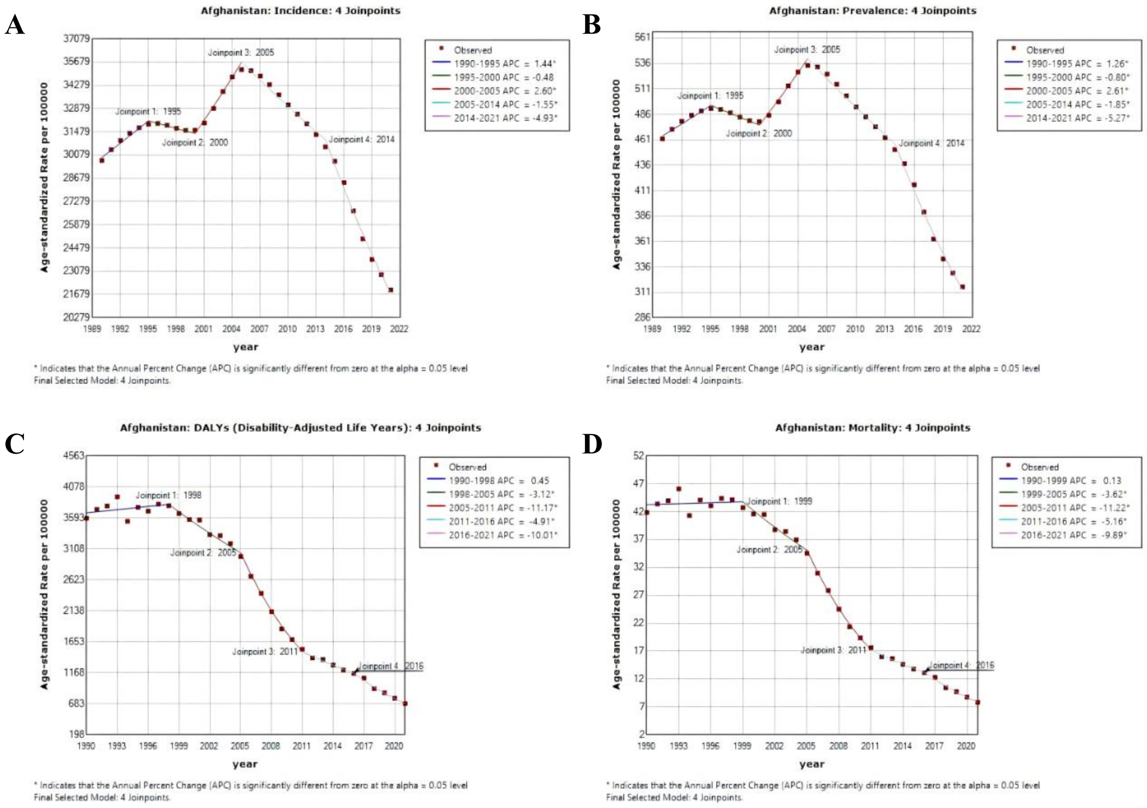

Figure S50: Temporal joinpoint analysis of diarrheal diseases in Afghanistan from 1990 to 2021.

(A) Temporal joinpoint analysis of ASIR; (B) Temporal joinpoint analysis of ASPR; (C) Temporal joinpoint analysis of ASDR; (D) Temporal joinpoint analysis of ASMR.

APC, annual percentage change; ASIR, age-standardized incidence rate; ASPR, age-standardized prevalence rate; ASDR, age-standardized DALYs rate; ASMR, age-standardized mortality rate.

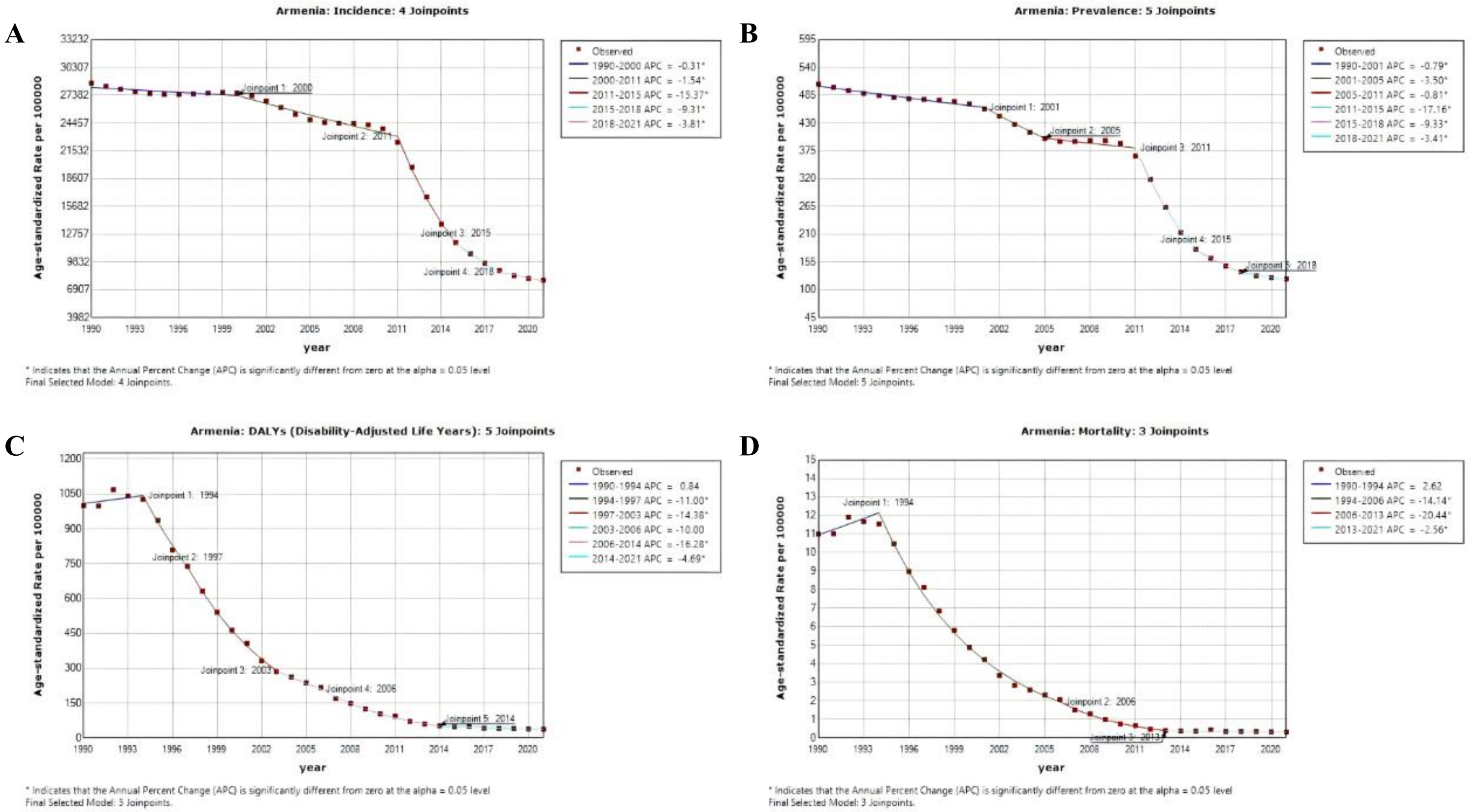

Figure S51: Temporal joinpoint analysis of diarrheal diseases in Armenia from 1990 to 2021.

(A) Temporal joinpoint analysis of ASIR; (B) Temporal joinpoint analysis of ASPR; (C) Temporal joinpoint analysis of ASDR; (D) Temporal joinpoint analysis of ASMR.

APC, annual percentage change; ASIR, age-standardized incidence rate; ASPR, age-standardized prevalence rate; ASDR, age-standardized DALYs rate; ASMR, age-standardized mortality rate.

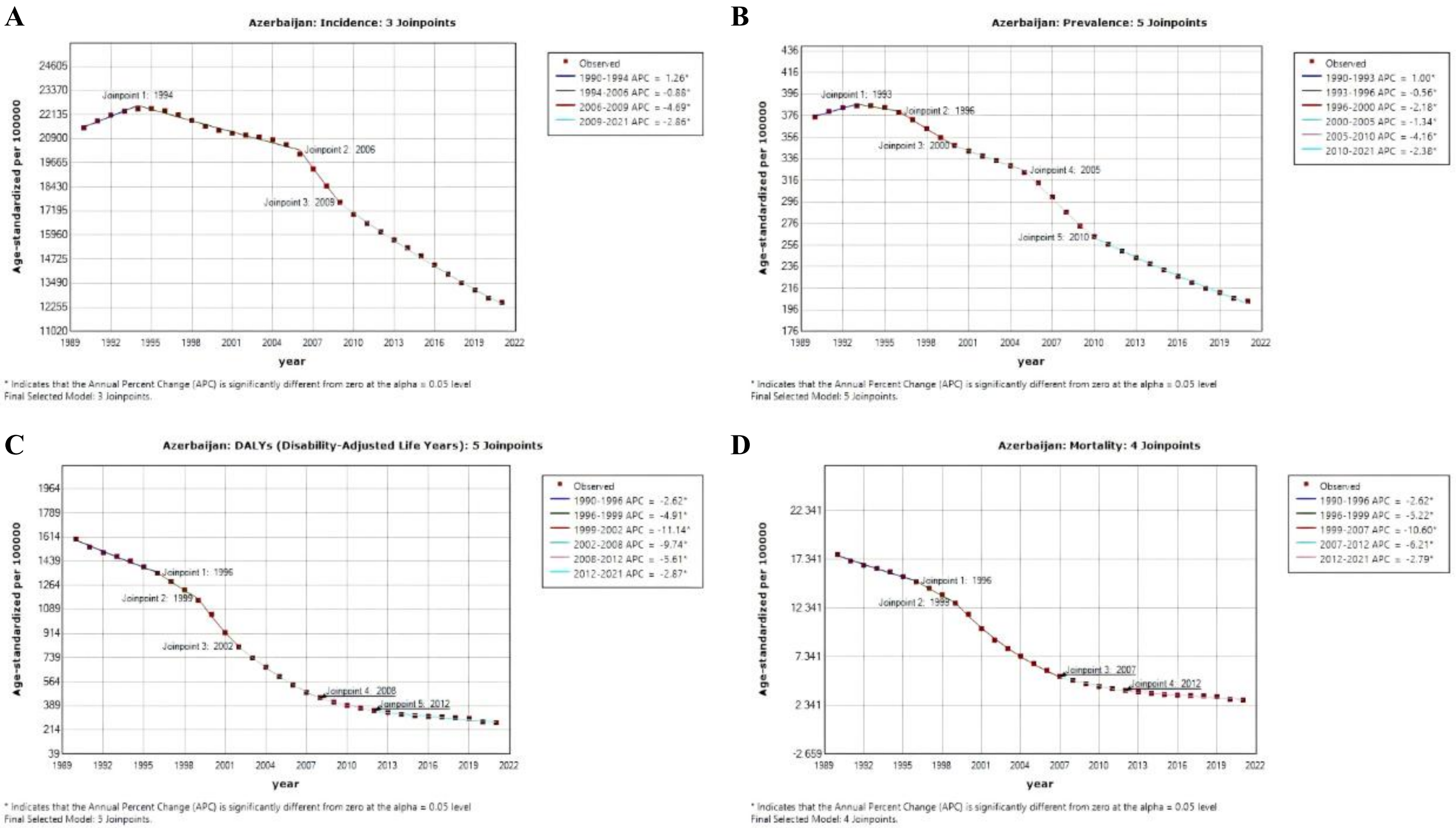

Figure S52: Temporal joinpoint analysis of diarrheal diseases in Azerbaijan from 1990 to 2021.

(A) Temporal joinpoint analysis of ASIR; (B) Temporal joinpoint analysis of ASPR; (C) Temporal joinpoint analysis of ASDR; (D) Temporal joinpoint analysis of ASMR.

APC, annual percentage change; ASIR, age-standardized incidence rate; ASPR, age-standardized prevalence rate; ASDR, age-standardized DALYs rate; ASMR, age-standardized mortality rate.

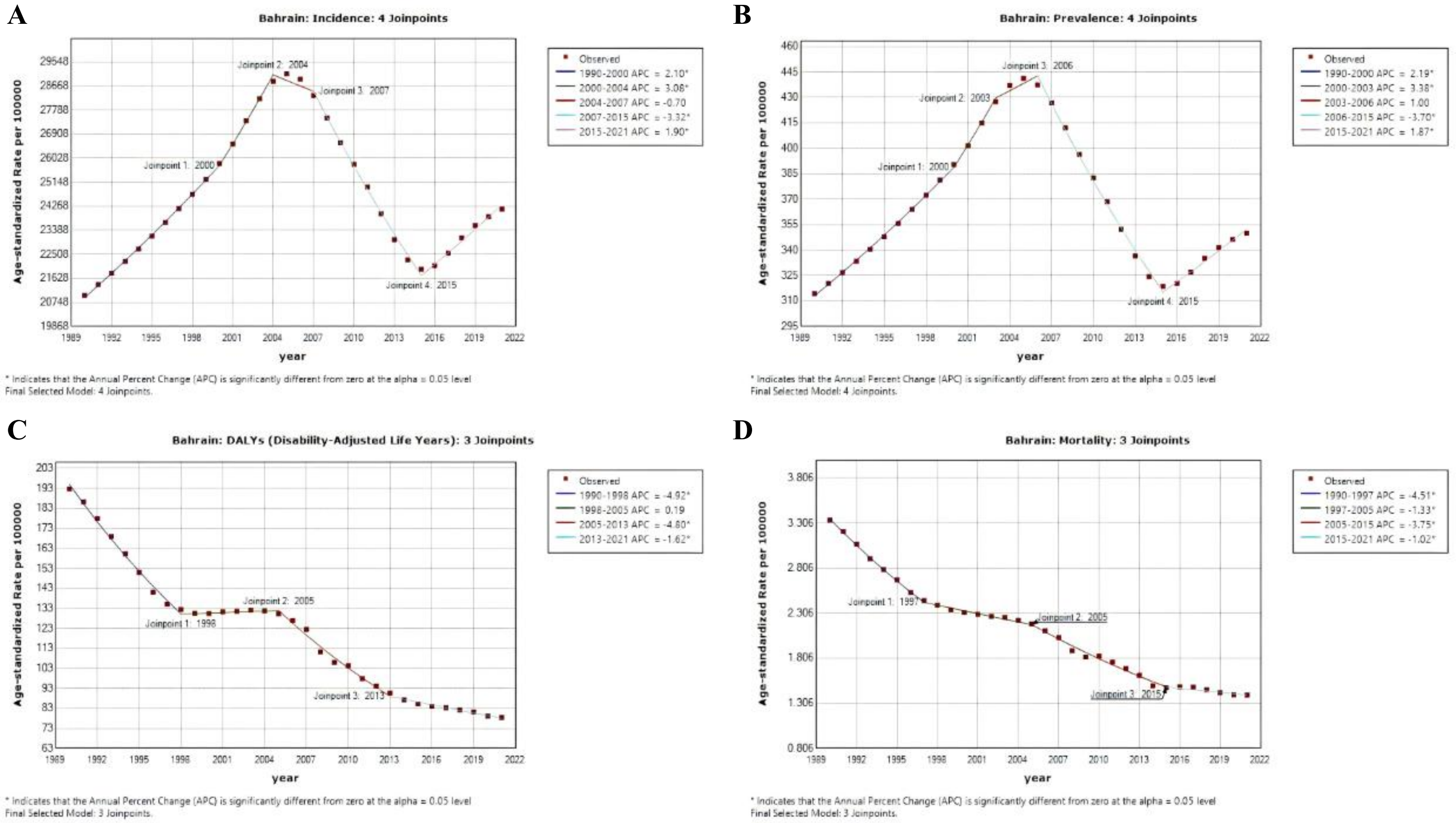

Figure S53: Temporal joinpoint analysis of diarrheal diseases in Bahrain from 1990 to 2021.

(A) Temporal joinpoint analysis of ASIR; (B) Temporal joinpoint analysis of ASPR; (C) Temporal joinpoint analysis of ASDR; (D) Temporal joinpoint analysis of ASMR.

APC, annual percentage change; ASIR, age-standardized incidence rate; ASPR, age-standardized prevalence rate; ASDR, age-standardized DALYs rate; ASMR, age-standardized mortality rate.

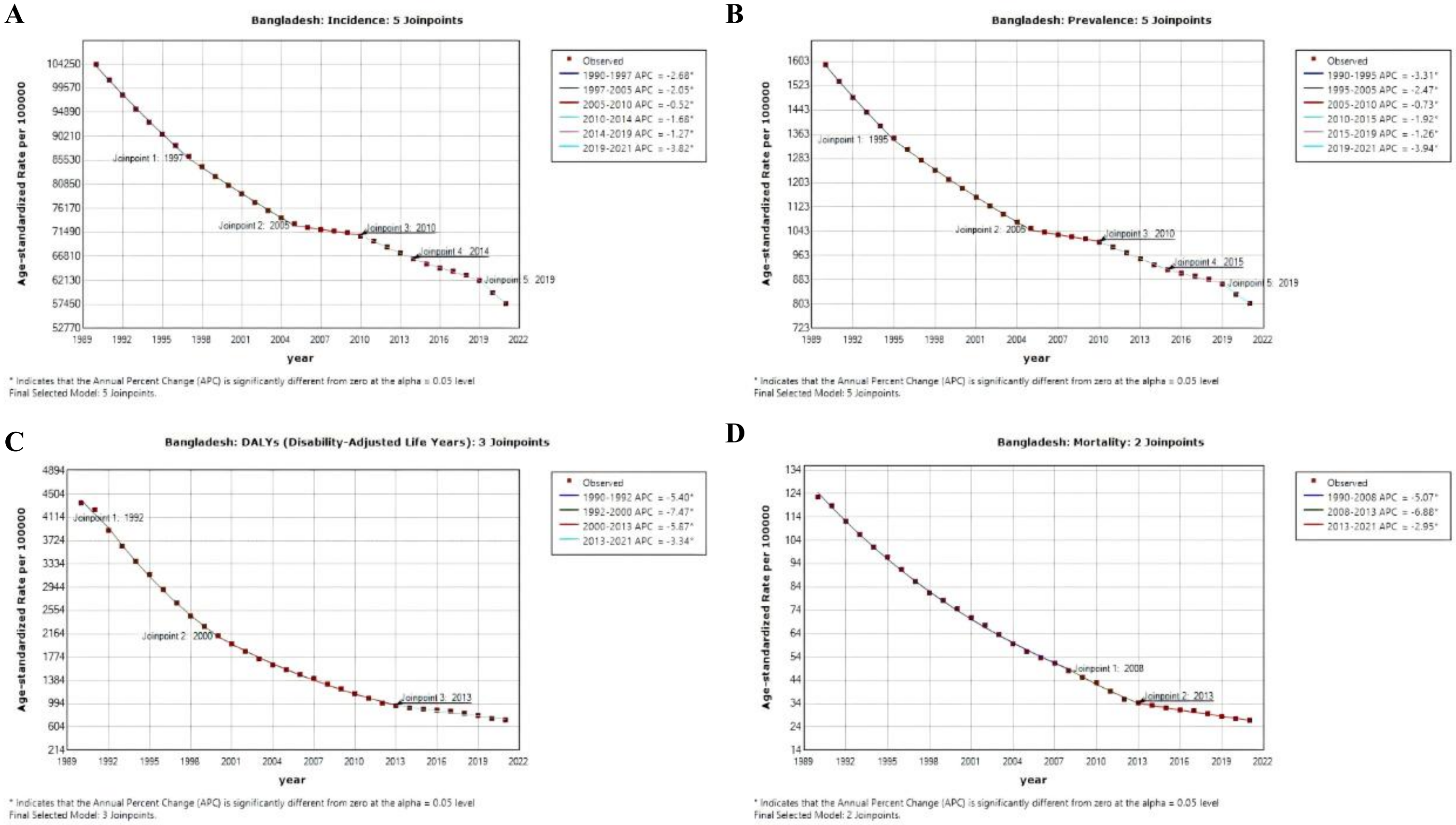

Figure S54: Temporal joinpoint analysis of diarrheal diseases in Bangladesh from 1990 to 2021.

(A) Temporal joinpoint analysis of ASIR; (B) Temporal joinpoint analysis of ASPR; (C) Temporal joinpoint analysis of ASDR; (D) Temporal joinpoint analysis of ASMR.

APC, annual percentage change; ASIR, age-standardized incidence rate; ASPR, age-standardized prevalence rate; ASDR, age-standardized DALYs rate; ASMR, age-standardized mortality rate.

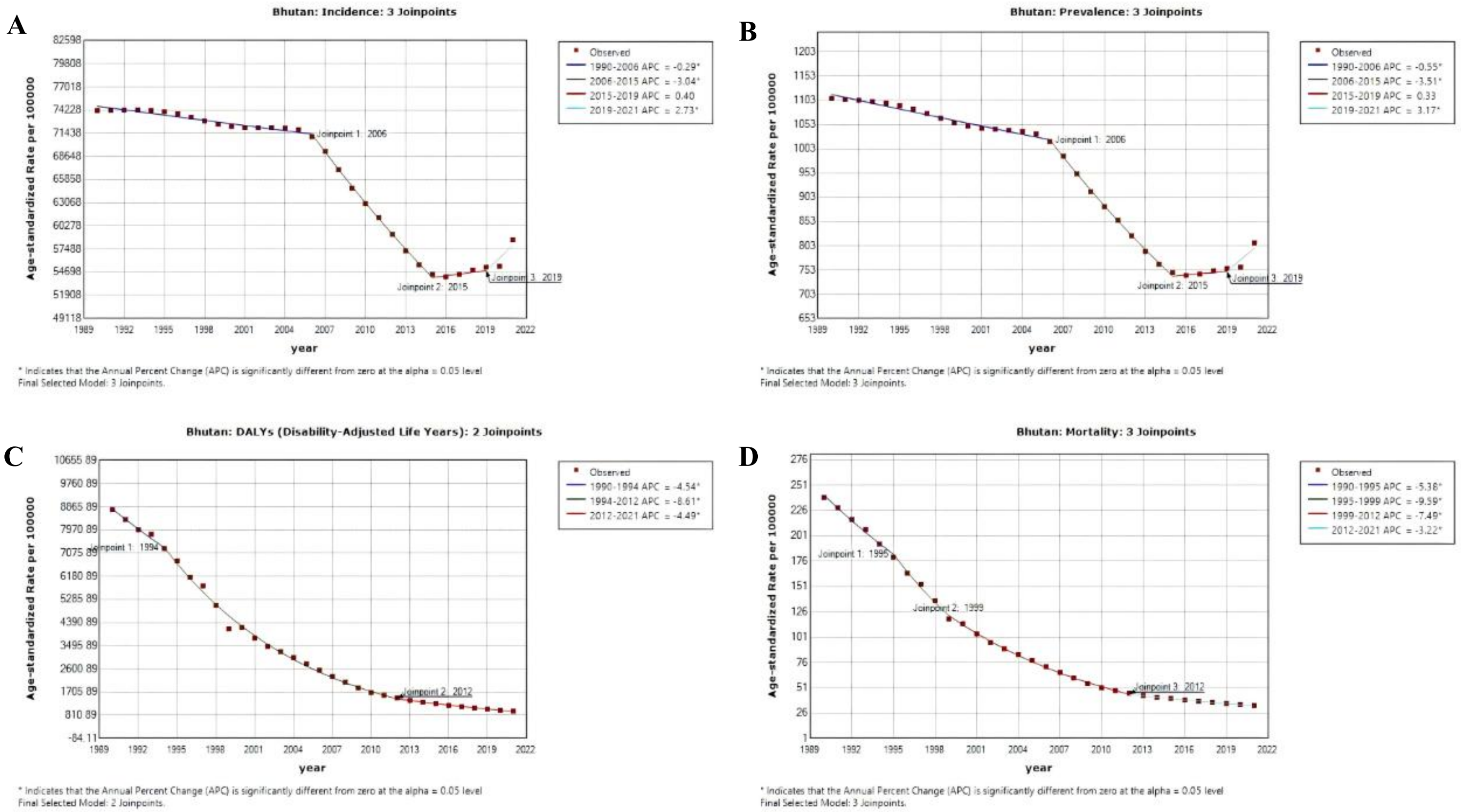

Figure S55: Temporal joinpoint analysis of diarrheal diseases in Bhutan from 1990 to 2021.

(A) Temporal joinpoint analysis of ASIR; (B) Temporal joinpoint analysis of ASPR; (C) Temporal joinpoint analysis of ASDR; (D) Temporal joinpoint analysis of ASMR.

APC, annual percentage change; ASIR, age-standardized incidence rate; ASPR, age-standardized prevalence rate; ASDR, age-standardized DALYs rate; ASMR, age-standardized mortality rate.

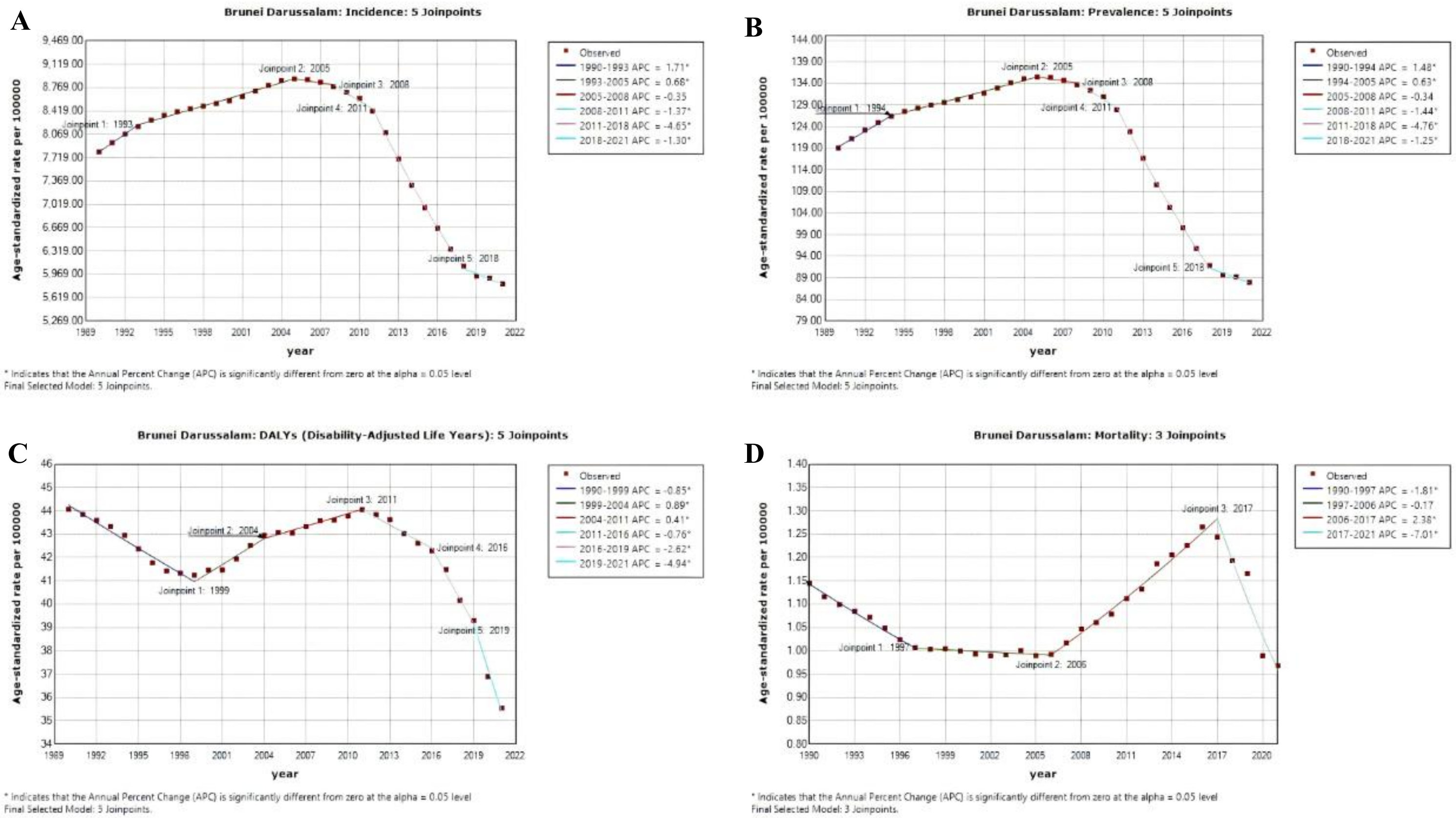

Figure S56: Temporal joinpoint analysis of diarrheal diseases in Brunei Darussalam from 1990 to 2021.

(A) Temporal joinpoint analysis of ASIR; (B) Temporal joinpoint analysis of ASPR; (C) Temporal joinpoint analysis of ASDR; (D) Temporal joinpoint analysis of ASMR.

APC, annual percentage change; ASIR, age-standardized incidence rate; ASPR, age-standardized prevalence rate; ASDR, age-standardized DALYs rate; ASMR, age-standardized mortality rate.

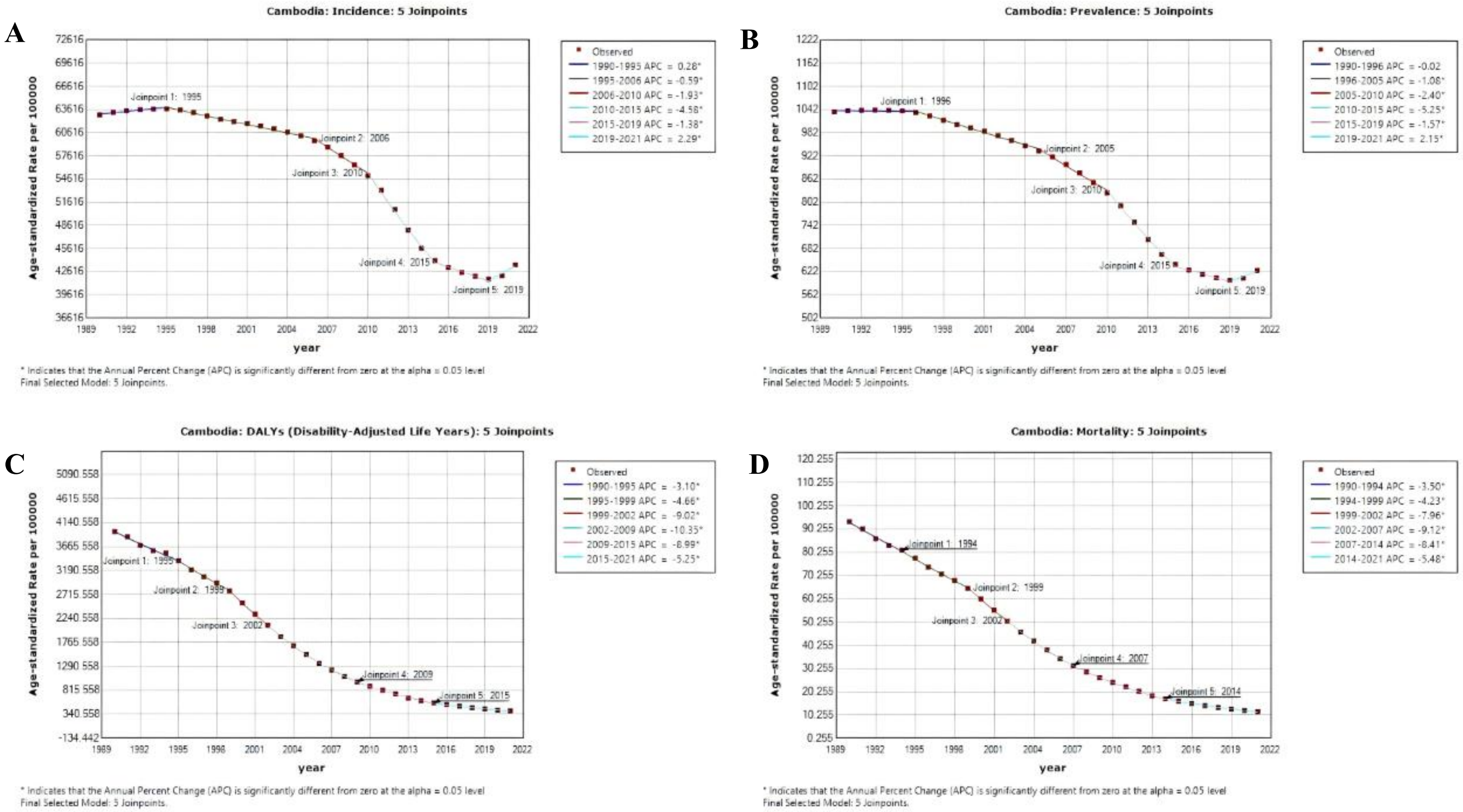

Figure S57: Temporal joinpoint analysis of diarrheal diseases in Cambodia from 1990 to 2021.

(A) Temporal joinpoint analysis of ASIR; (B) Temporal joinpoint analysis of ASPR; (C) Temporal joinpoint analysis of ASDR; (D) Temporal joinpoint analysis of ASMR.

APC, annual percentage change; ASIR, age-standardized incidence rate; ASPR, age-standardized prevalence rate; ASDR, age-standardized DALYs rate; ASMR, age-standardized mortality rate.

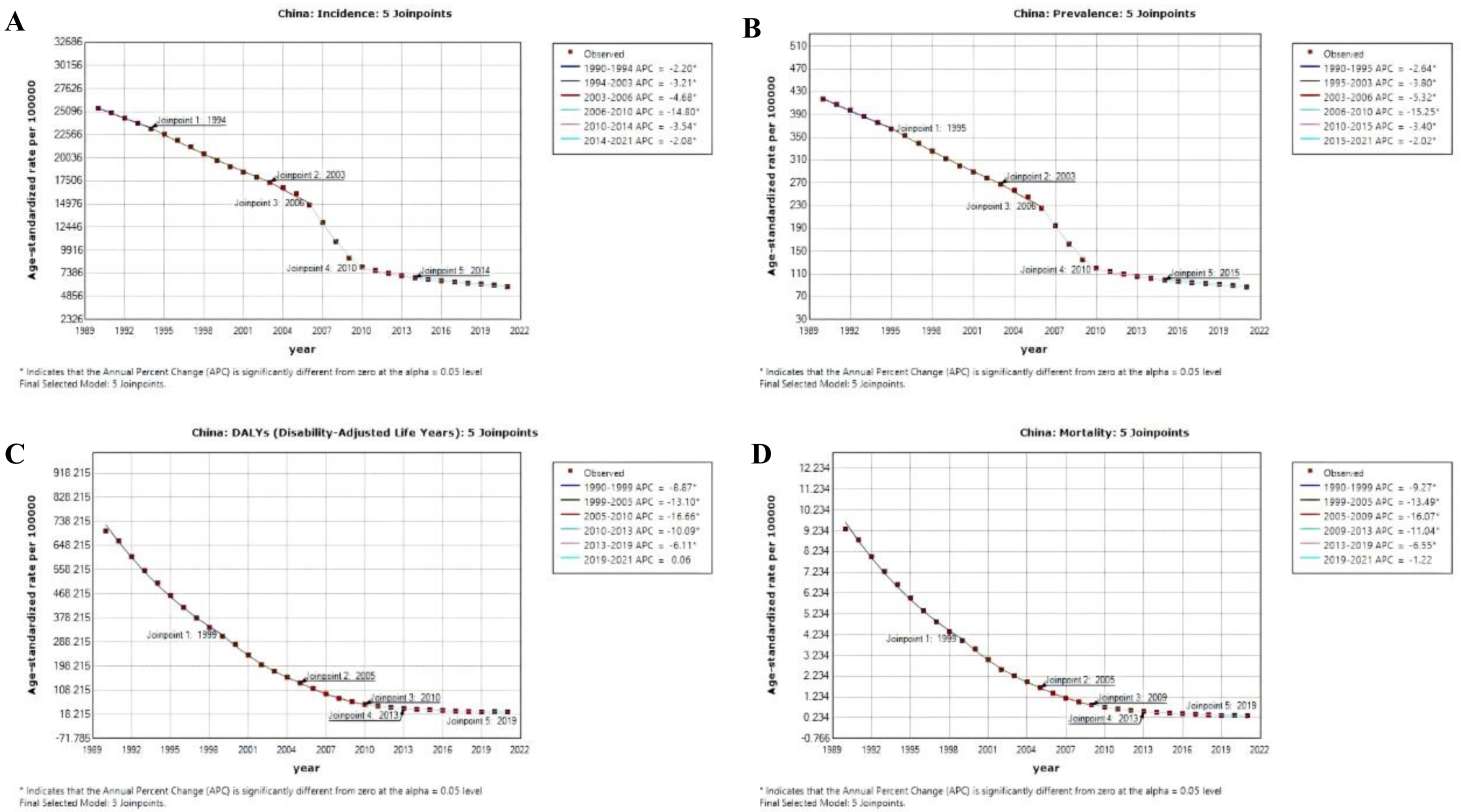

Figure S58: Temporal joinpoint analysis of diarrheal diseases in China from 1990 to 2021.

(A) Temporal joinpoint analysis of ASIR; (B) Temporal joinpoint analysis of ASPR; (C) Temporal joinpoint analysis of ASDR; (D) Temporal joinpoint analysis of ASMR.

APC, annual percentage change; ASIR, age-standardized incidence rate; ASPR, age-standardized prevalence rate; ASDR, age-standardized DALYs rate; ASMR, age-standardized mortality rate.

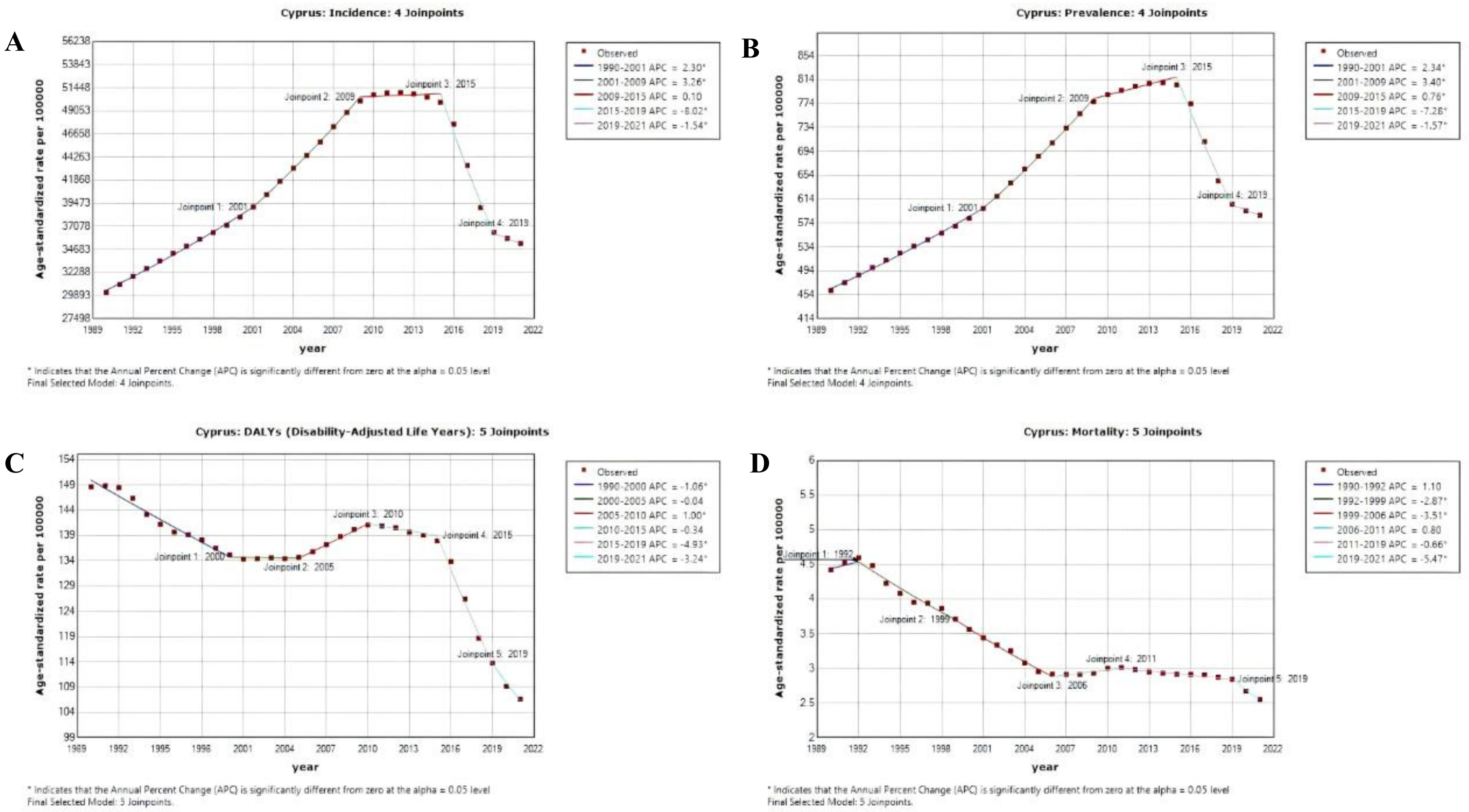

Figure S59: Temporal joinpoint analysis of diarrheal diseases in Cyprus from 1990 to 2021.

(A) Temporal joinpoint analysis of ASIR; (B) Temporal joinpoint analysis of ASPR; (C) Temporal joinpoint analysis of ASDR; (D) Temporal joinpoint analysis of ASMR.

APC, annual percentage change; ASIR, age-standardized incidence rate; ASPR, age-standardized prevalence rate; ASDR, age-standardized DALYs rate; ASMR, age-standardized mortality rate.

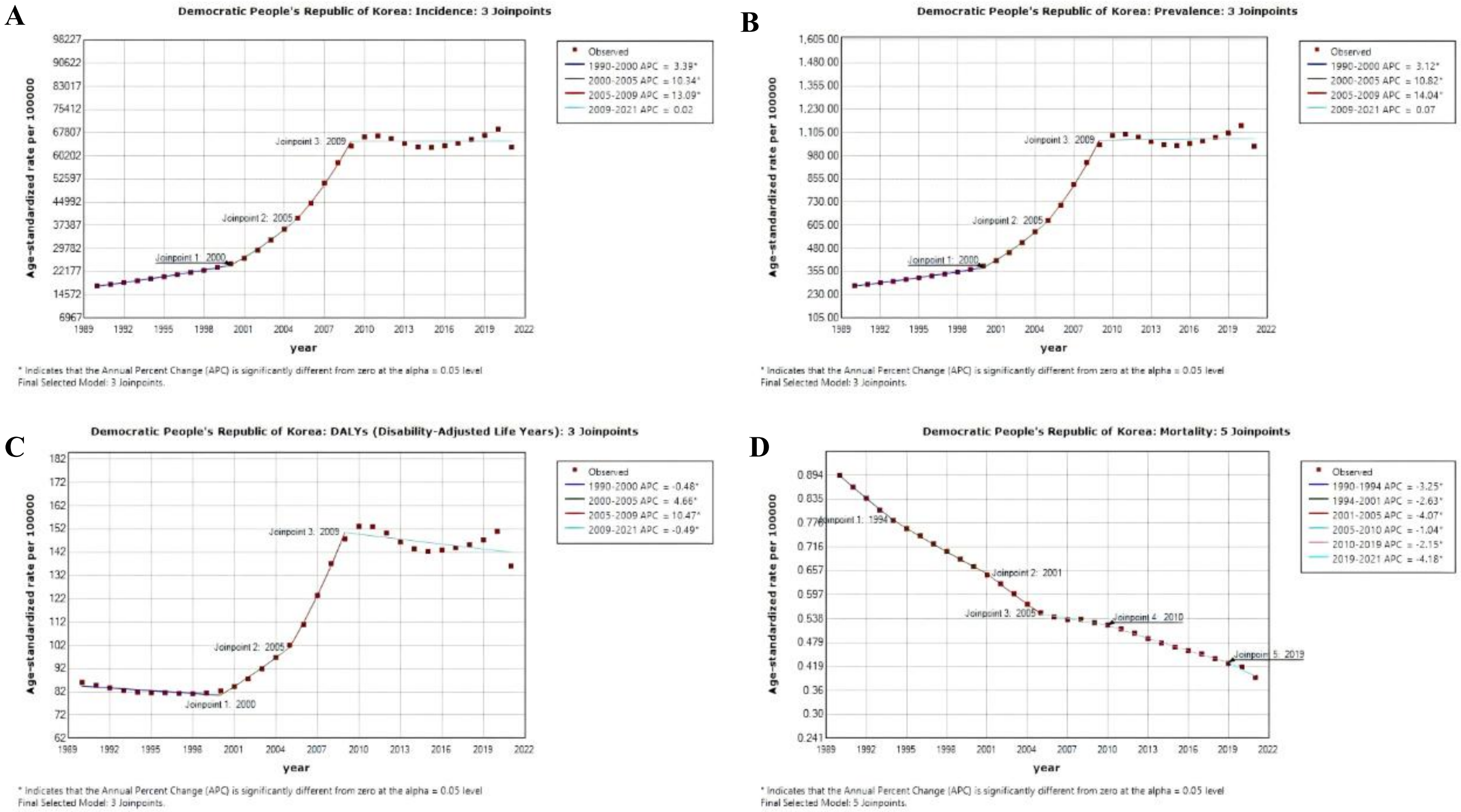

Figure S60: Temporal joinpoint analysis of diarrheal diseases in Democratic People's Republic of Korea from 1990 to 2021.

(A) Temporal joinpoint analysis of ASIR; (B) Temporal joinpoint analysis of ASPR; (C) Temporal joinpoint analysis of ASDR; (D) Temporal joinpoint analysis of ASMR.

APC, annual percentage change; ASIR, age-standardized incidence rate; ASPR, age-standardized prevalence rate; ASDR, age-standardized DALYs rate; ASMR, age-standardized mortality rate.

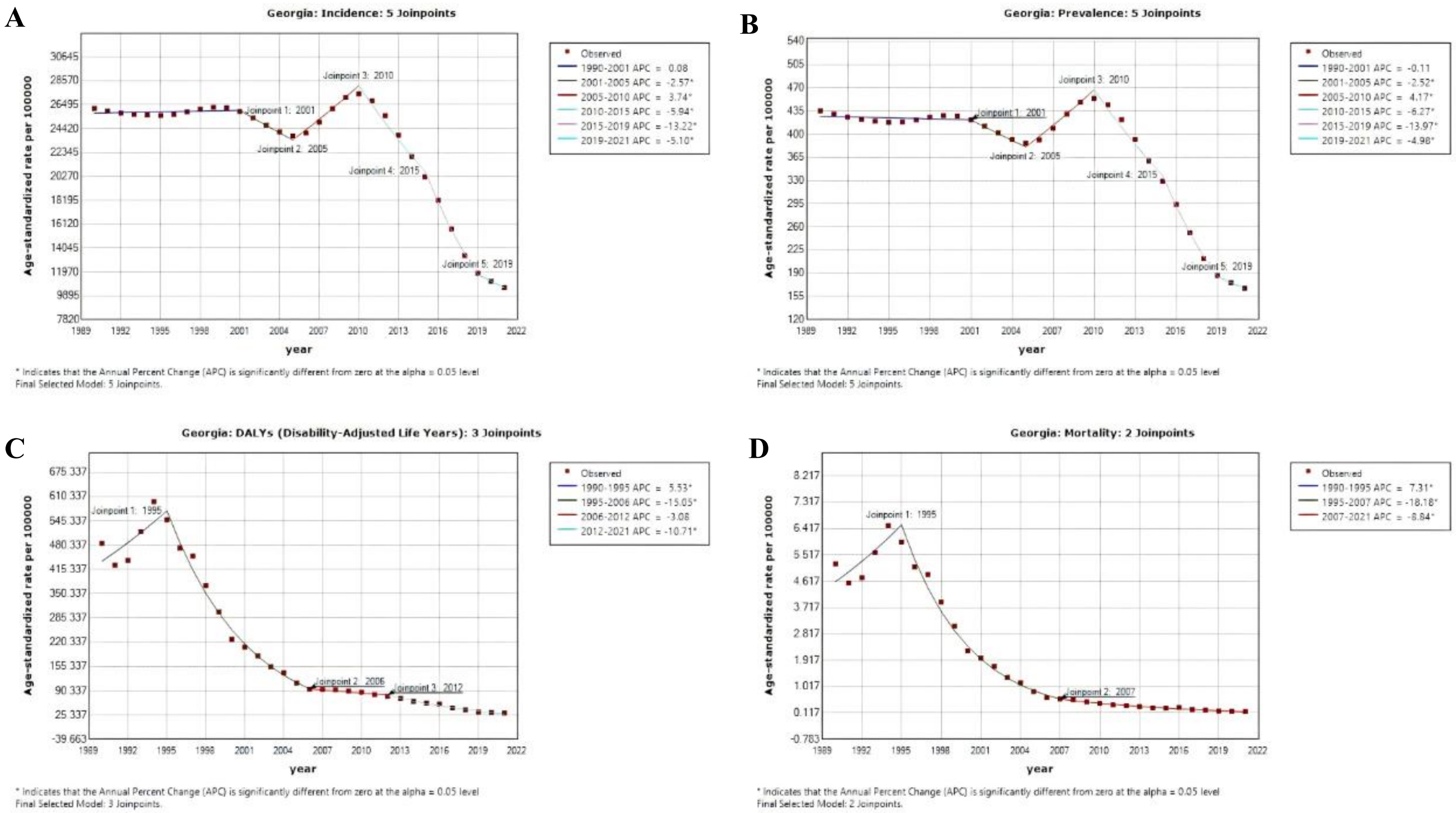

Figure S61: Temporal joinpoint analysis of diarrheal diseases in Georgia from 1990 to 2021.

(A) Temporal joinpoint analysis of ASIR; (B) Temporal joinpoint analysis of ASPR; (C) Temporal joinpoint analysis of ASDR; (D) Temporal joinpoint analysis of ASMR.

APC, annual percentage change; ASIR, age-standardized incidence rate; ASPR, age-standardized prevalence rate; ASDR, age-standardized DALYs rate; ASMR, age-standardized mortality rate.

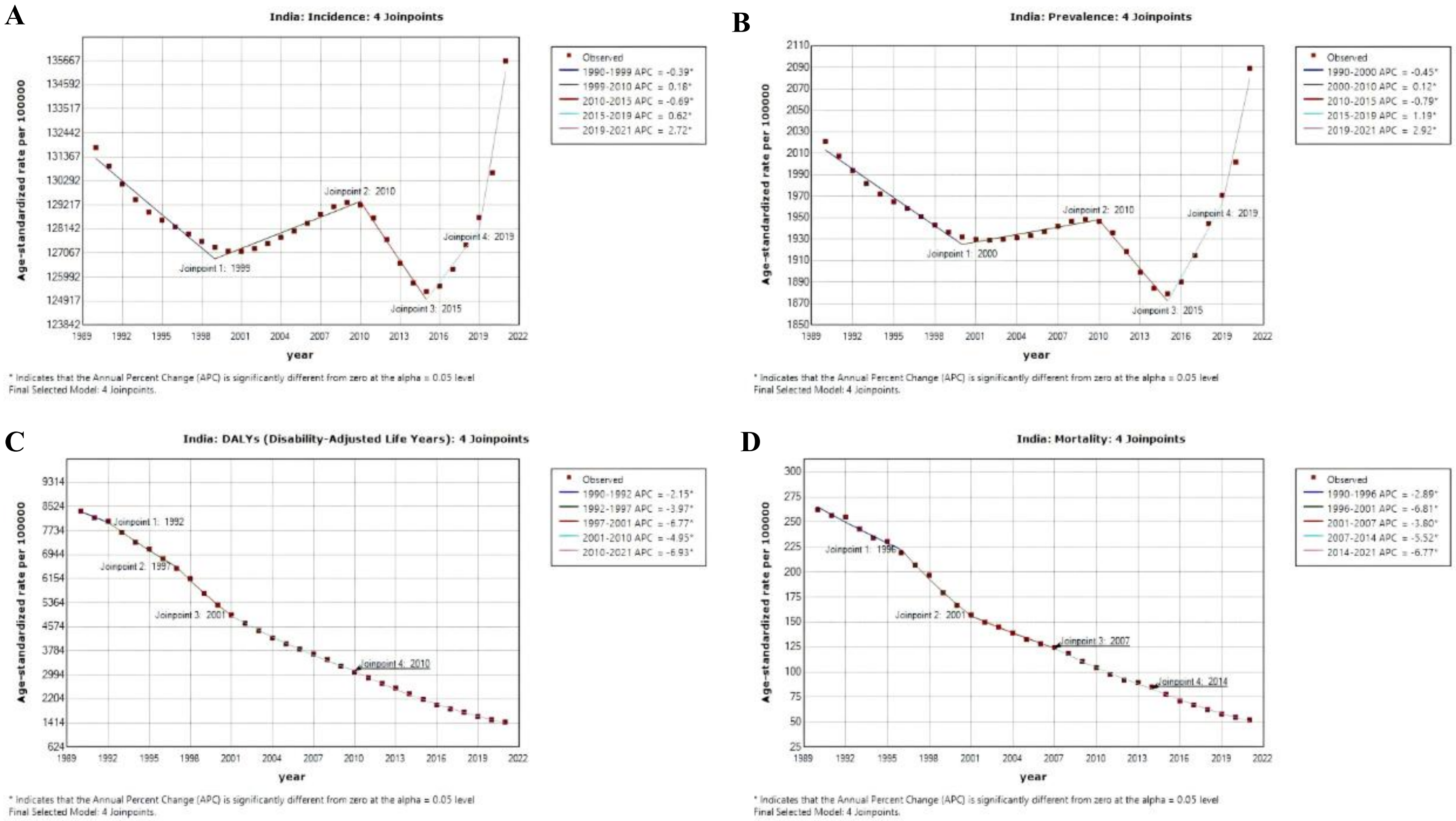

Figure S62: Temporal joinpoint analysis of diarrheal diseases in India from 1990 to 2021.

(A) Temporal joinpoint analysis of ASIR; (B) Temporal joinpoint analysis of ASPR; (C) Temporal joinpoint analysis of ASDR; (D) Temporal joinpoint analysis of ASMR.

APC, annual percentage change; ASIR, age-standardized incidence rate; ASPR, age-standardized prevalence rate; ASDR, age-standardized DALYs rate; ASMR, age-standardized mortality rate.

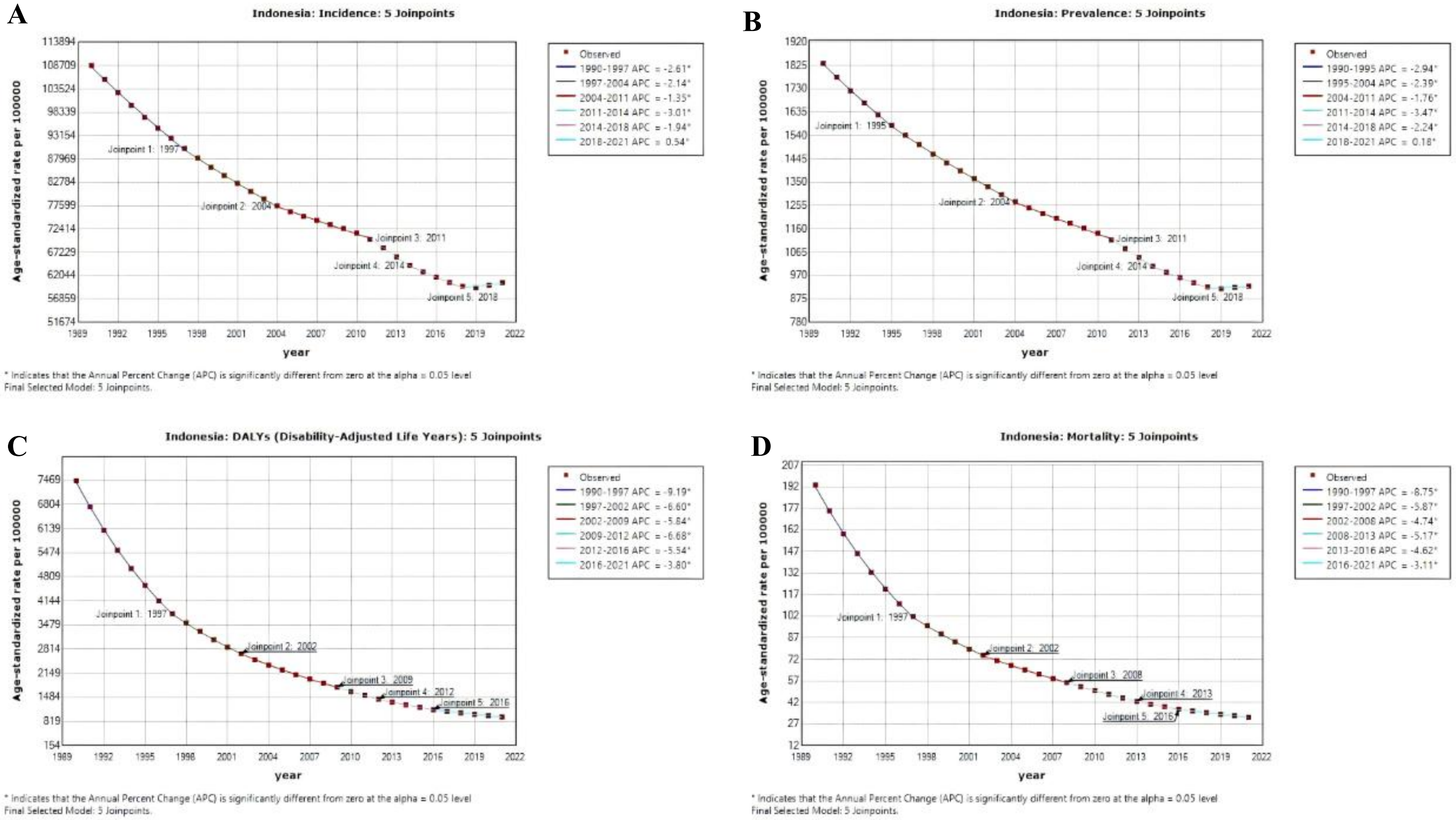

Figure S63: Temporal joinpoint analysis of diarrheal diseases in Indonesia from 1990 to 2021.

(A) Temporal joinpoint analysis of ASIR; (B) Temporal joinpoint analysis of ASPR; (C) Temporal joinpoint analysis of ASDR; (D) Temporal joinpoint analysis of ASMR.

APC, annual percentage change; ASIR, age-standardized incidence rate; ASPR, age-standardized prevalence rate; ASDR, age-standardized DALYs rate; ASMR, age-standardized mortality rate.

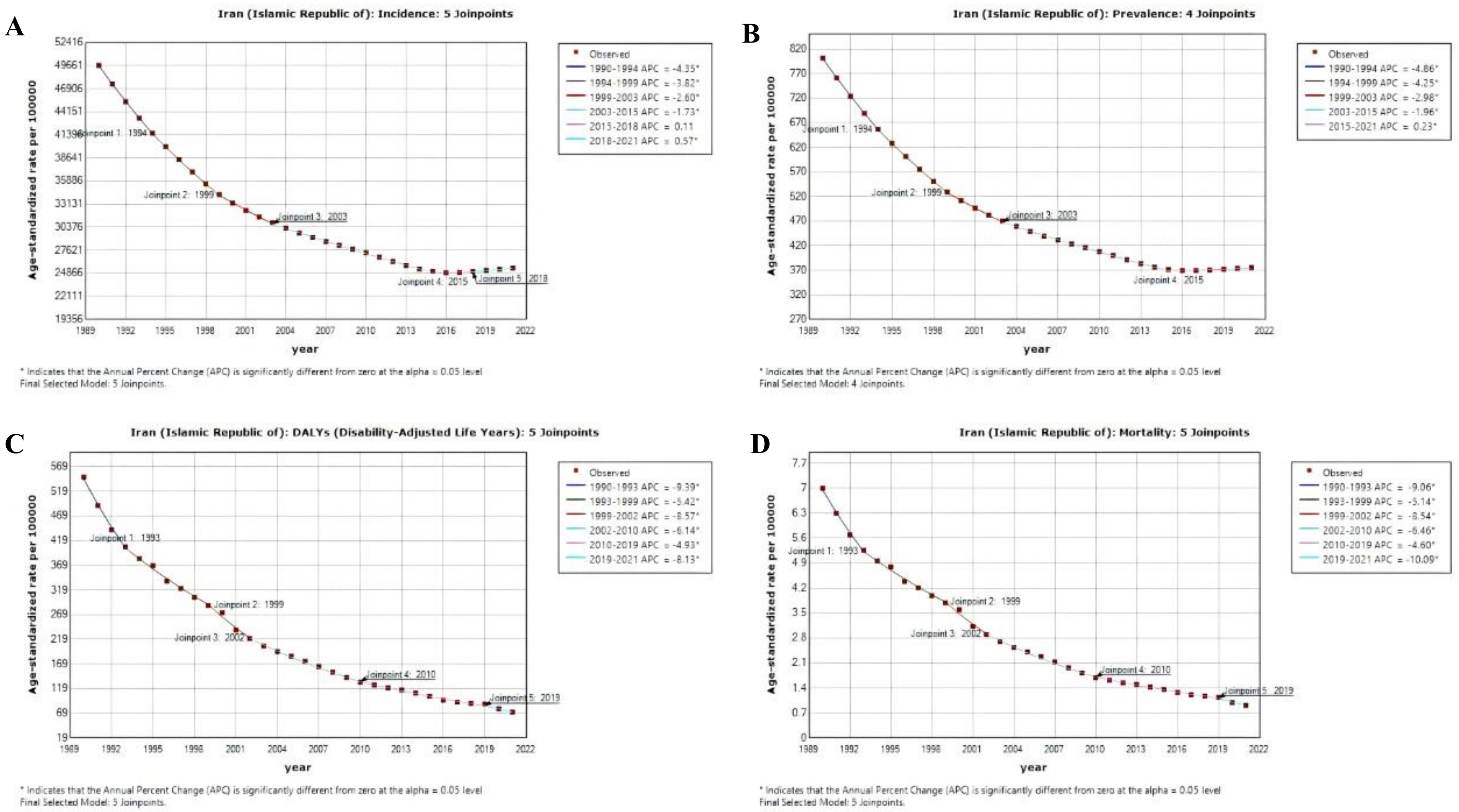

Figure S64: Temporal joinpoint analysis of diarrheal diseases in Iran (Islamic Republic of) from 1990 to 2021.

(A) Temporal joinpoint analysis of ASIR; (B) Temporal joinpoint analysis of ASPR; (C) Temporal joinpoint analysis of ASDR; (D) Temporal joinpoint analysis of ASMR.

APC, annual percentage change; ASIR, age-standardized incidence rate; ASPR, age-standardized prevalence rate; ASDR, age-standardized DALYs rate; ASMR, age-standardized mortality rate.

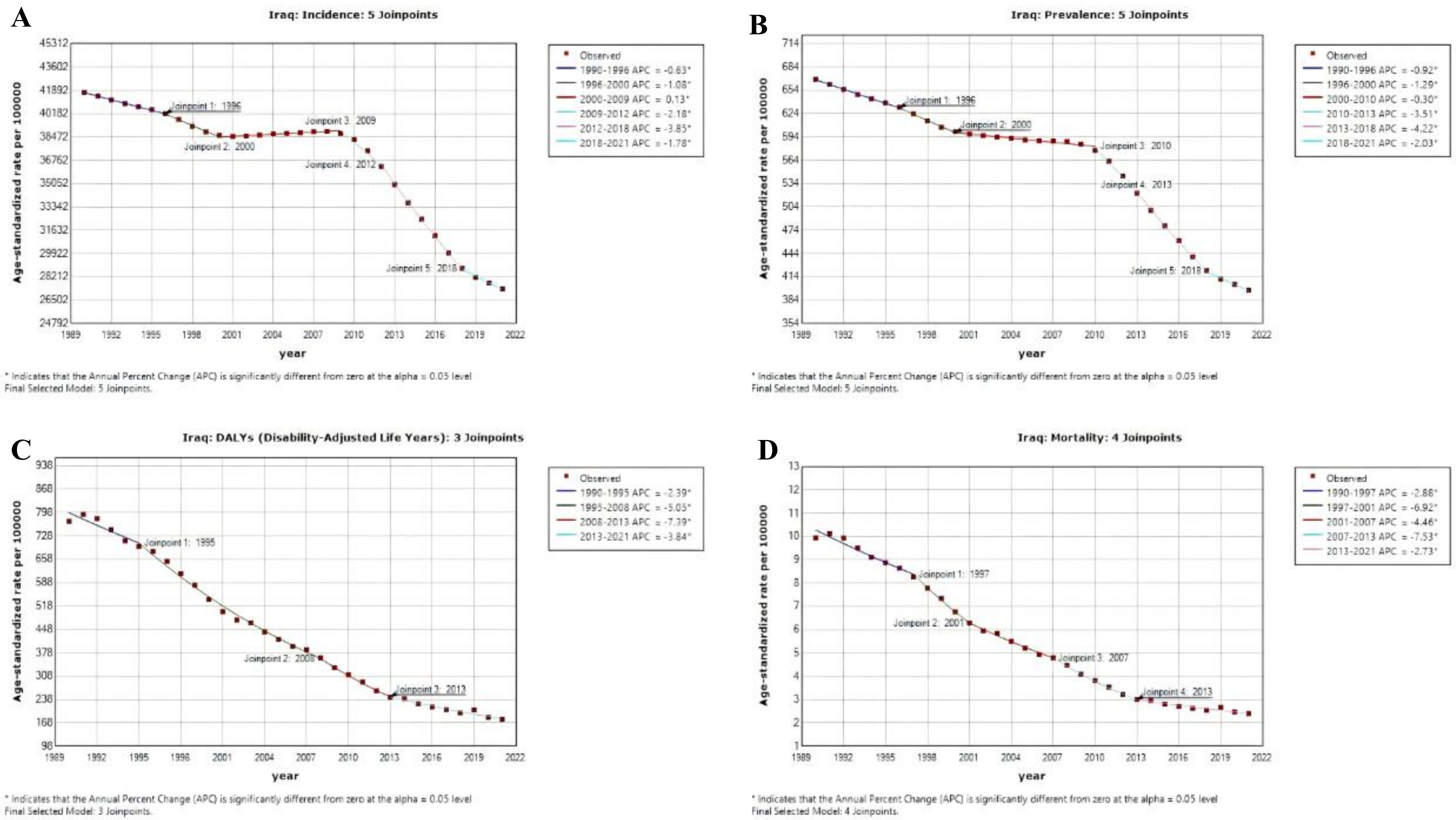

Figure S65: Temporal joinpoint analysis of diarrheal diseases in Iraq from 1990 to 2021.

(A) Temporal joinpoint analysis of ASIR; (B) Temporal joinpoint analysis of ASPR; (C) Temporal joinpoint analysis of ASDR; (D) Temporal joinpoint analysis of ASMR.

APC, annual percentage change; ASIR, age-standardized incidence rate; ASPR, age-standardized prevalence rate; ASDR, age-standardized DALYs rate; ASMR, age-standardized mortality rate.

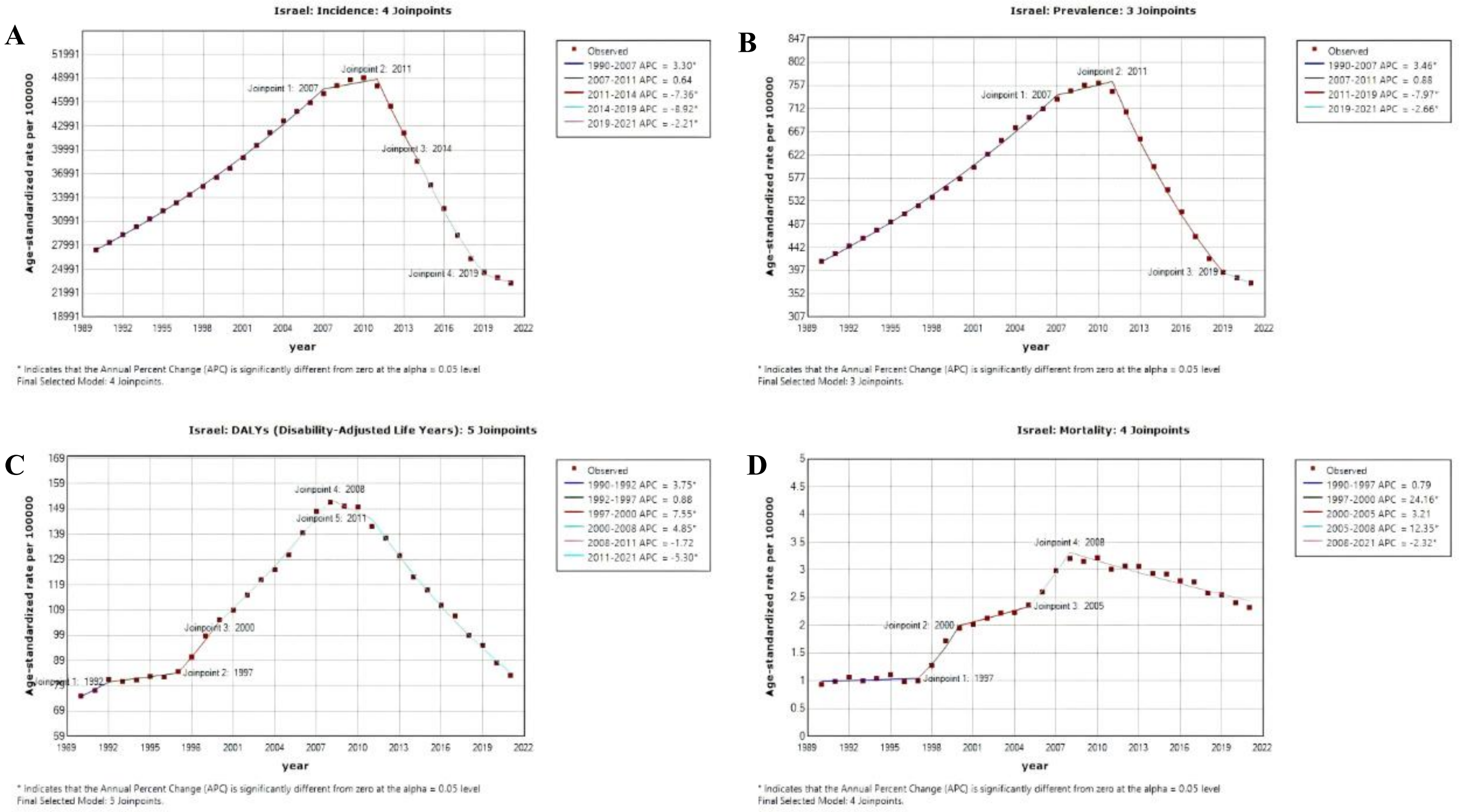

Figure S66: Temporal joinpoint analysis of diarrheal diseases in Israel from 1990 to 2021.

(A) Temporal joinpoint analysis of ASIR; (B) Temporal joinpoint analysis of ASPR; (C) Temporal joinpoint analysis of ASDR; (D) Temporal joinpoint analysis of ASMR.

APC, annual percentage change; ASIR, age-standardized incidence rate; ASPR, age-standardized prevalence rate; ASDR, age-standardized DALYs rate; ASMR, age-standardized mortality rate.

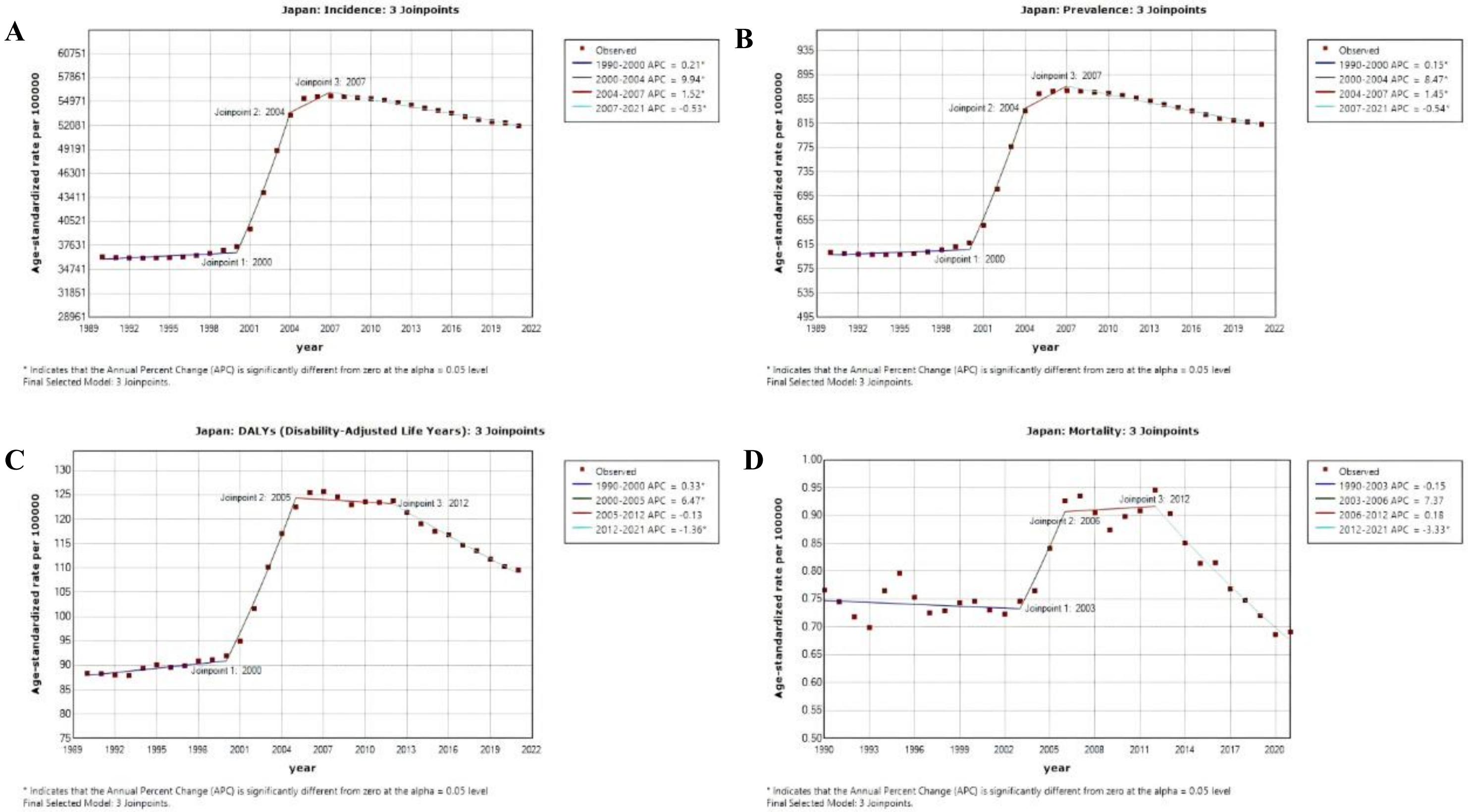

Figure S67: Temporal joinpoint analysis of diarrheal diseases in Japan from 1990 to 2021.

(A) Temporal joinpoint analysis of ASIR; (B) Temporal joinpoint analysis of ASPR; (C) Temporal joinpoint analysis of ASDR; (D) Temporal joinpoint analysis of ASMR.

APC, annual percentage change; ASIR, age-standardized incidence rate; ASPR, age-standardized prevalence rate; ASDR, age-standardized DALYs rate; ASMR, age-standardized mortality rate.

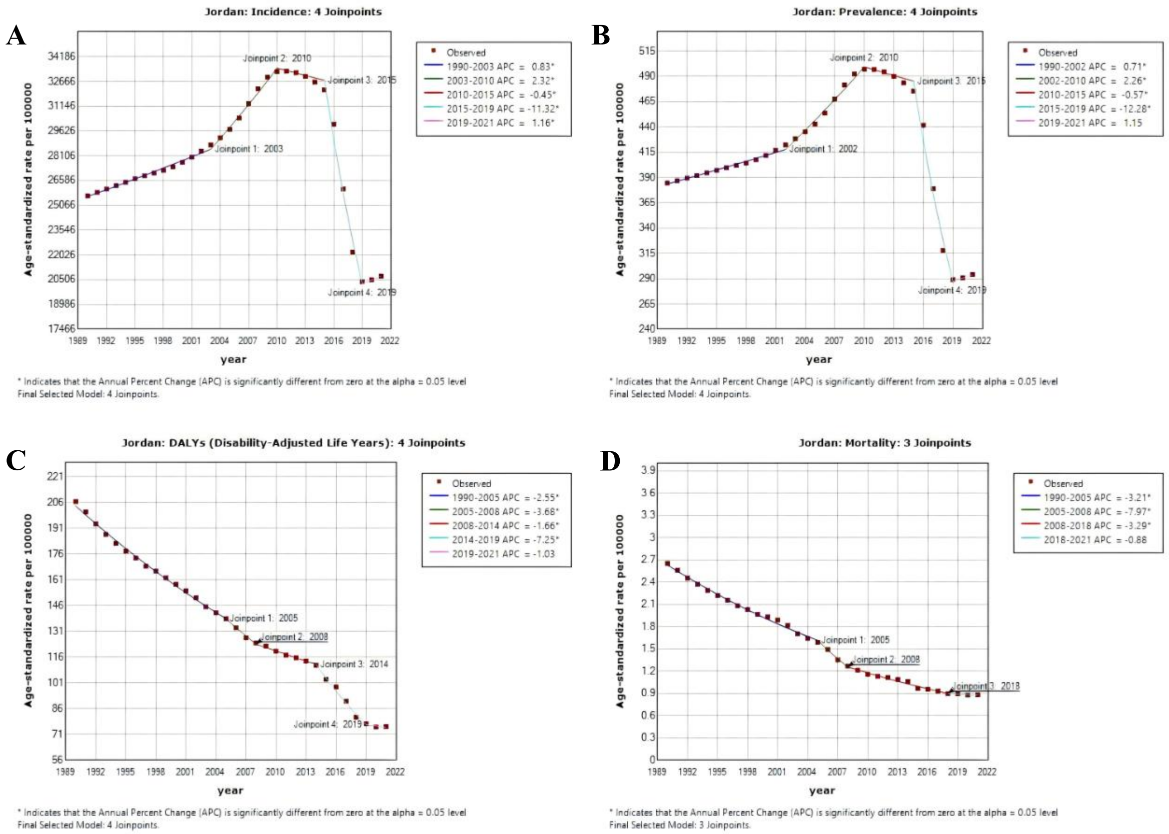

Figure S68: Temporal joinpoint analysis of diarrheal diseases in Jordan from 1990 to 2021.

(A) Temporal joinpoint analysis of ASIR; (B) Temporal joinpoint analysis of ASPR; (C) Temporal joinpoint analysis of ASDR; (D) Temporal joinpoint analysis of ASMR.

APC, annual percentage change; ASIR, age-standardized incidence rate; ASPR, age-standardized prevalence rate; ASDR, age-standardized DALYs rate; ASMR, age-standardized mortality rate.

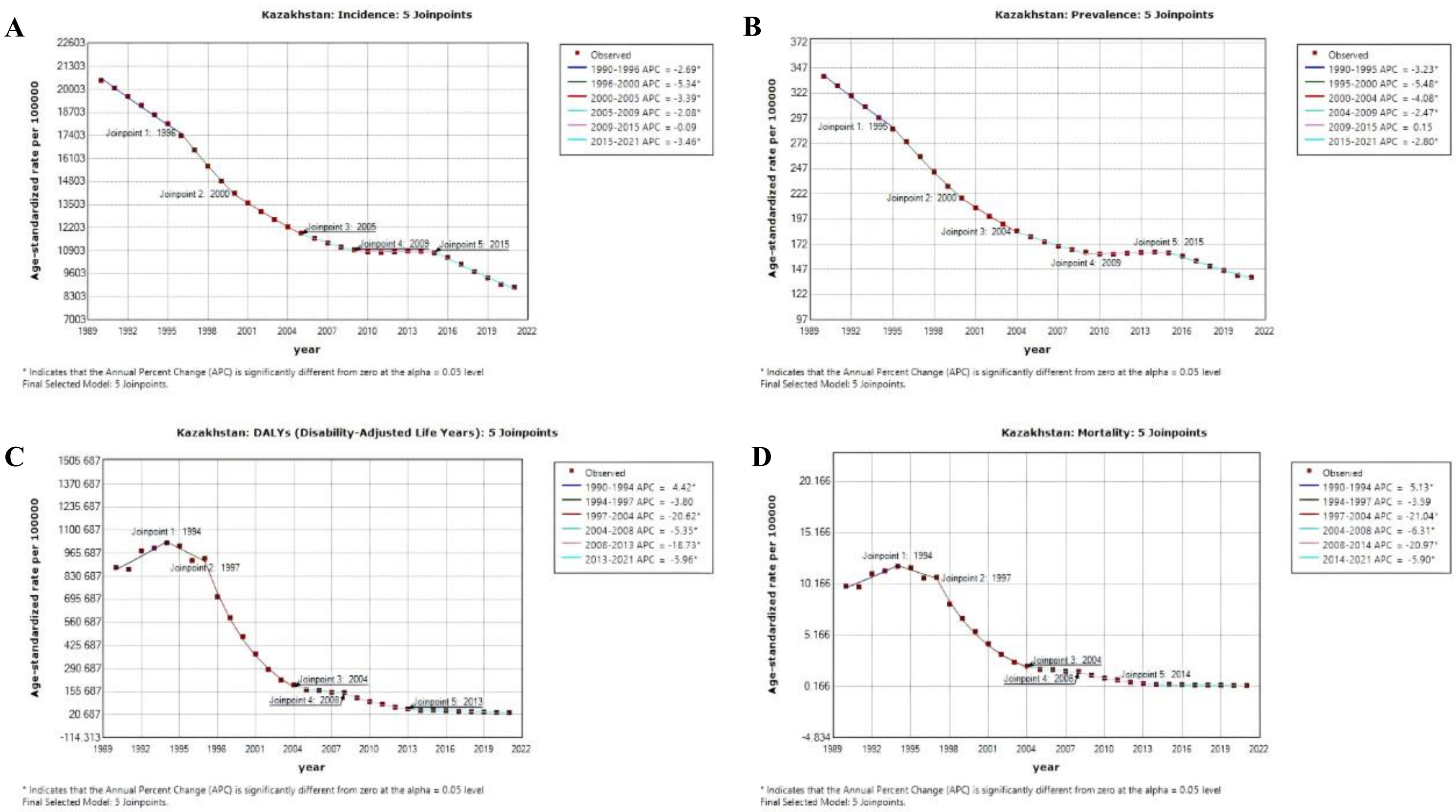

Figure S69: Temporal joinpoint analysis of diarrheal diseases in Kazakhstan from 1990 to 2021.

(A) Temporal joinpoint analysis of ASIR; (B) Temporal joinpoint analysis of ASPR; (C) Temporal joinpoint analysis of ASDR; (D) Temporal joinpoint analysis of ASMR.

APC, annual percentage change; ASIR, age-standardized incidence rate; ASPR, age-standardized prevalence rate; ASDR, age-standardized DALYs rate; ASMR, age-standardized mortality rate.

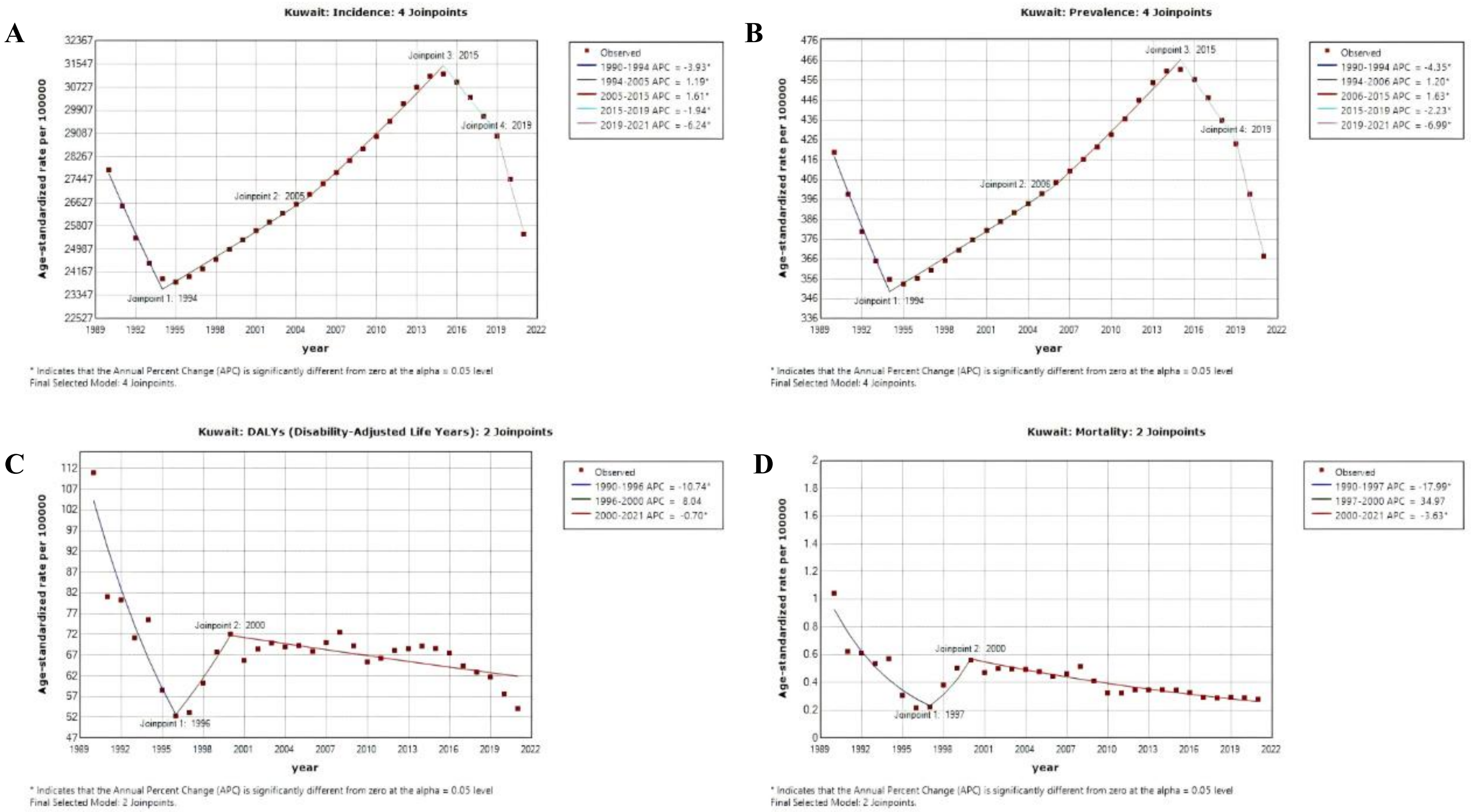

Figure S70: Temporal joinpoint analysis of diarrheal diseases in Kuwait from 1990 to 2021.

(A) Temporal joinpoint analysis of ASIR; (B) Temporal joinpoint analysis of ASPR; (C) Temporal joinpoint analysis of ASDR; (D) Temporal joinpoint analysis of ASMR.

APC, annual percentage change; ASIR, age-standardized incidence rate; ASPR, age-standardized prevalence rate; ASDR, age-standardized DALYs rate; ASMR, age-standardized mortality rate.

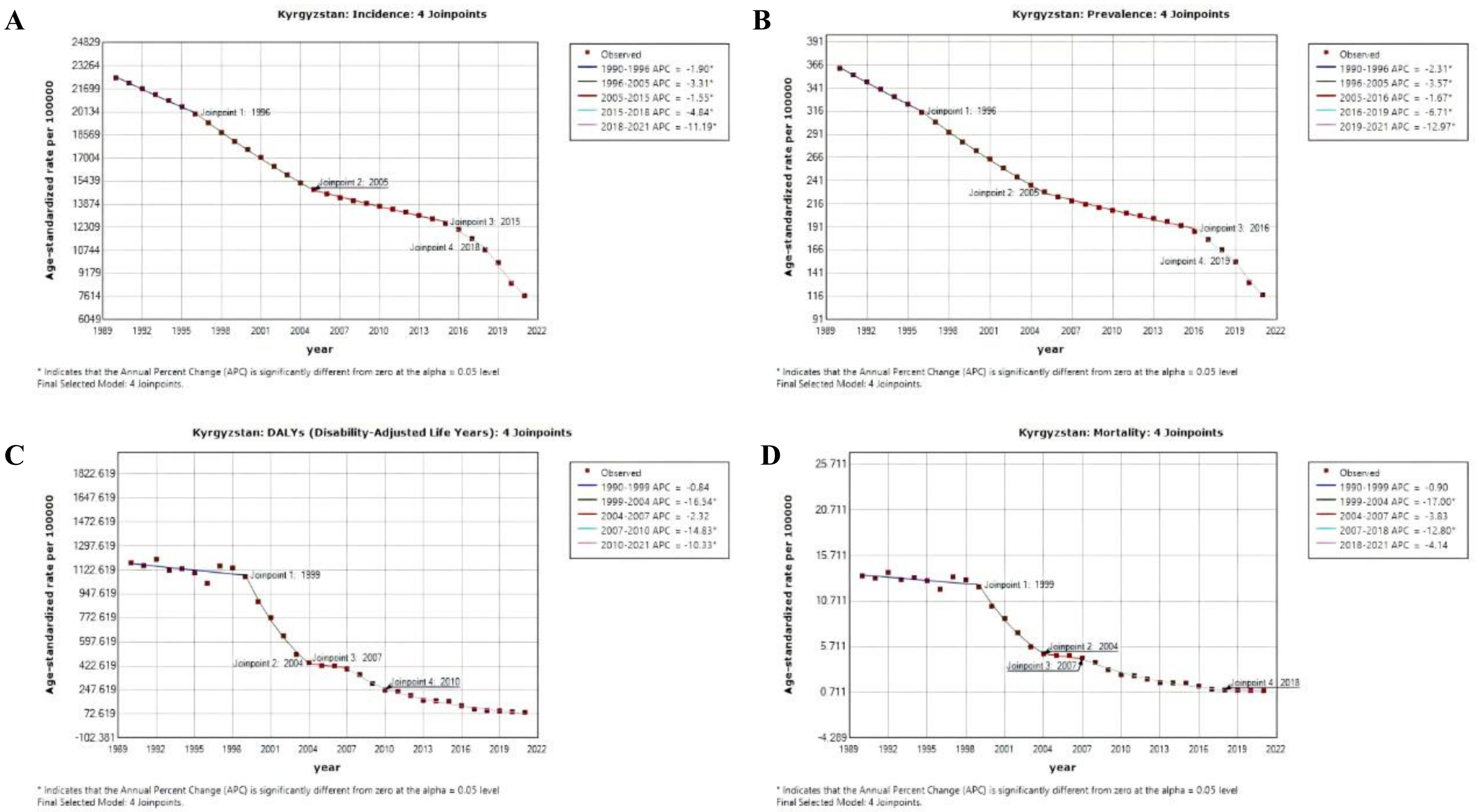

Figure S71: Temporal joinpoint analysis of diarrheal diseases in Kyrgyzstan from 1990 to 2021.

(A) Temporal joinpoint analysis of ASIR; (B) Temporal joinpoint analysis of ASPR; (C) Temporal joinpoint analysis of ASDR; (D) Temporal joinpoint analysis of ASMR.

APC, annual percentage change; ASIR, age-standardized incidence rate; ASPR, age-standardized prevalence rate; ASDR, age-standardized DALYs rate; ASMR, age-standardized mortality rate.

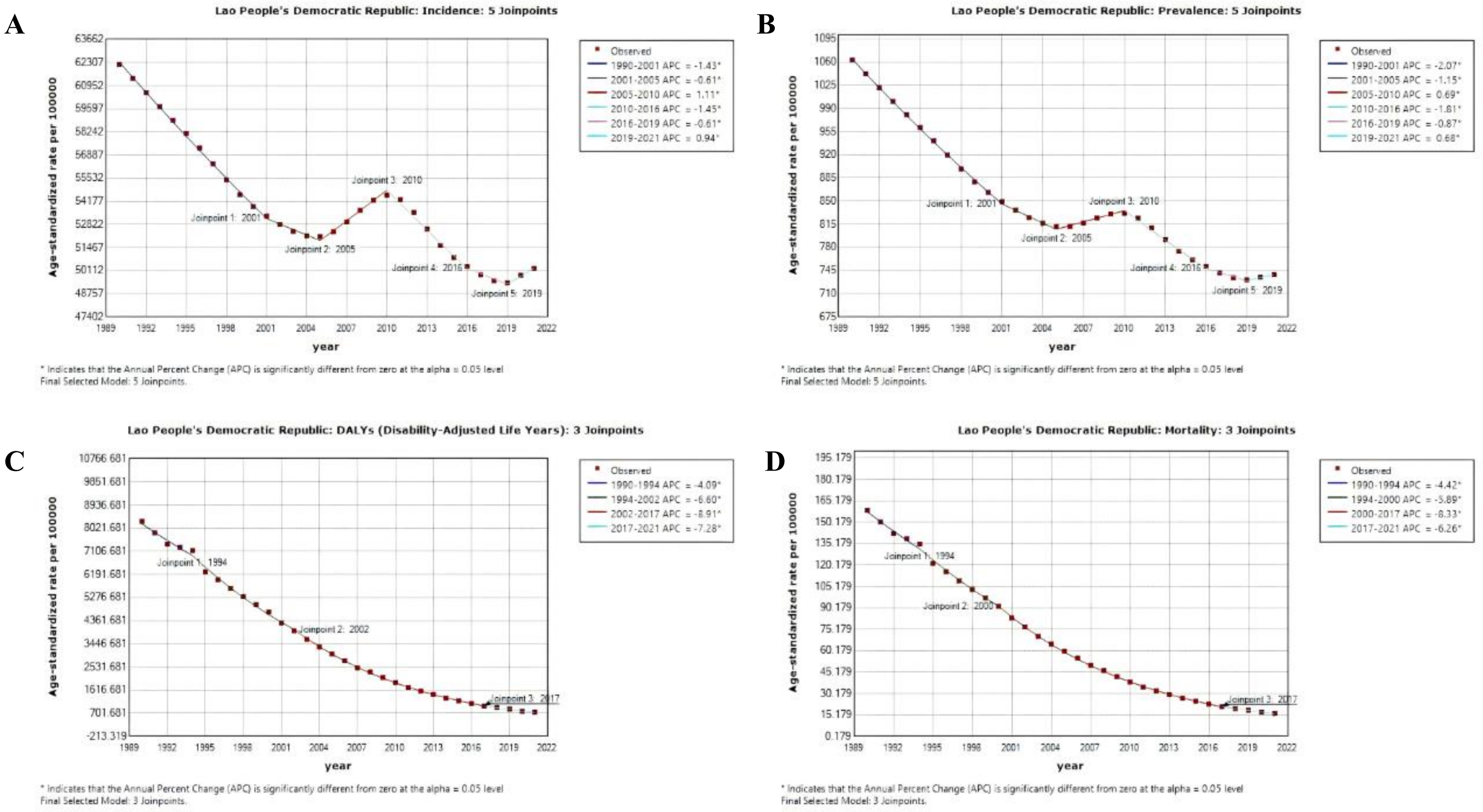

Figure S72: Temporal joinpoint analysis of diarrheal diseases in Lao People's Democratic Republic from 1990 to 2021.

(A) Temporal joinpoint analysis of ASIR; (B) Temporal joinpoint analysis of ASPR; (C) Temporal joinpoint analysis of ASDR; (D) Temporal joinpoint analysis of ASMR.

APC, annual percentage change; ASIR, age-standardized incidence rate; ASPR, age-standardized prevalence rate; ASDR, age-standardized DALYs rate; ASMR, age-standardized mortality rate.

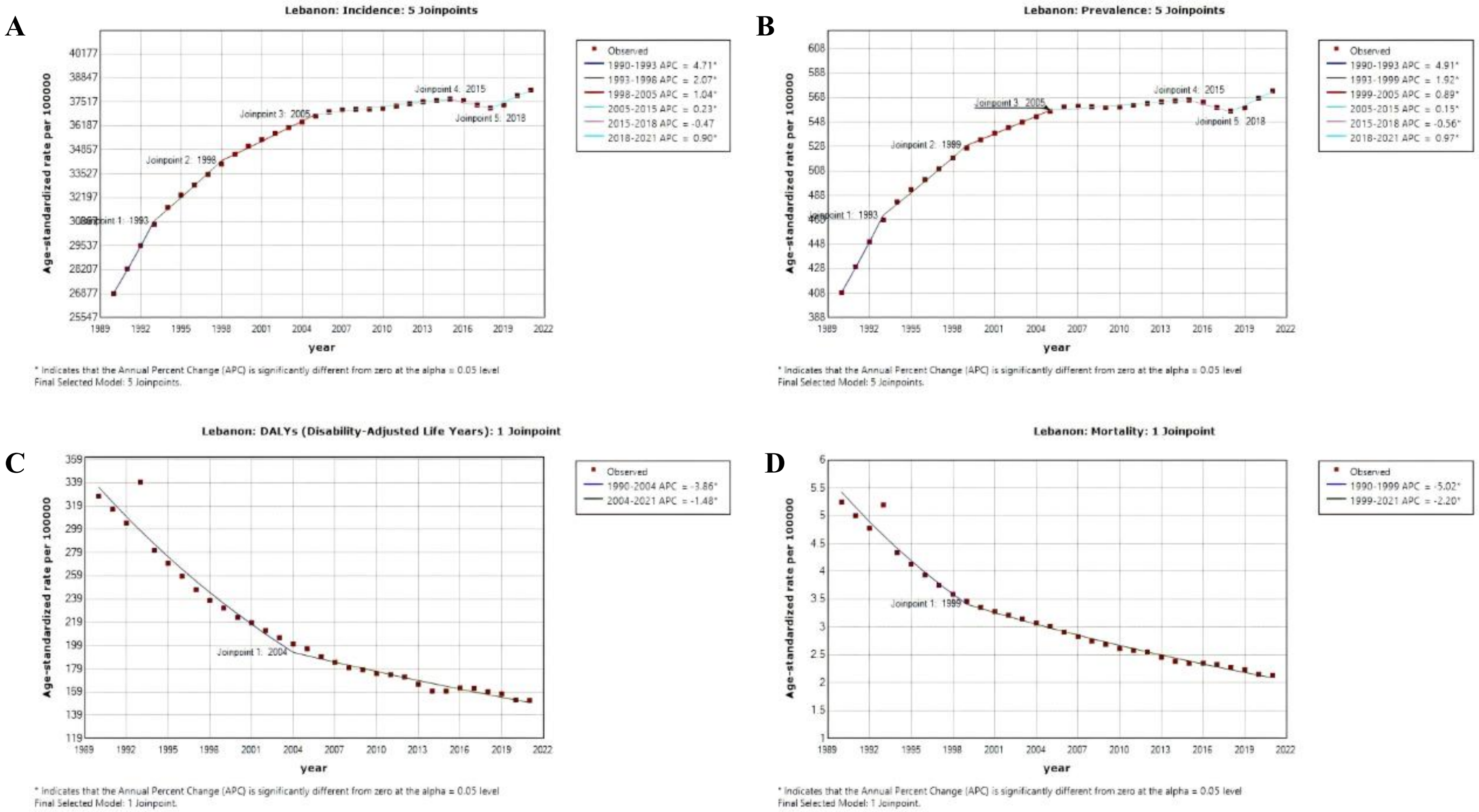

Figure S73: Temporal joinpoint analysis of diarrheal diseases in Lebanon from 1990 to 2021.

(A) Temporal joinpoint analysis of ASIR; (B) Temporal joinpoint analysis of ASPR; (C) Temporal joinpoint analysis of ASDR; (D) Temporal joinpoint analysis of ASMR.

APC, annual percentage change; ASIR, age-standardized incidence rate; ASPR, age-standardized prevalence rate; ASDR, age-standardized DALYs rate; ASMR, age-standardized mortality rate.

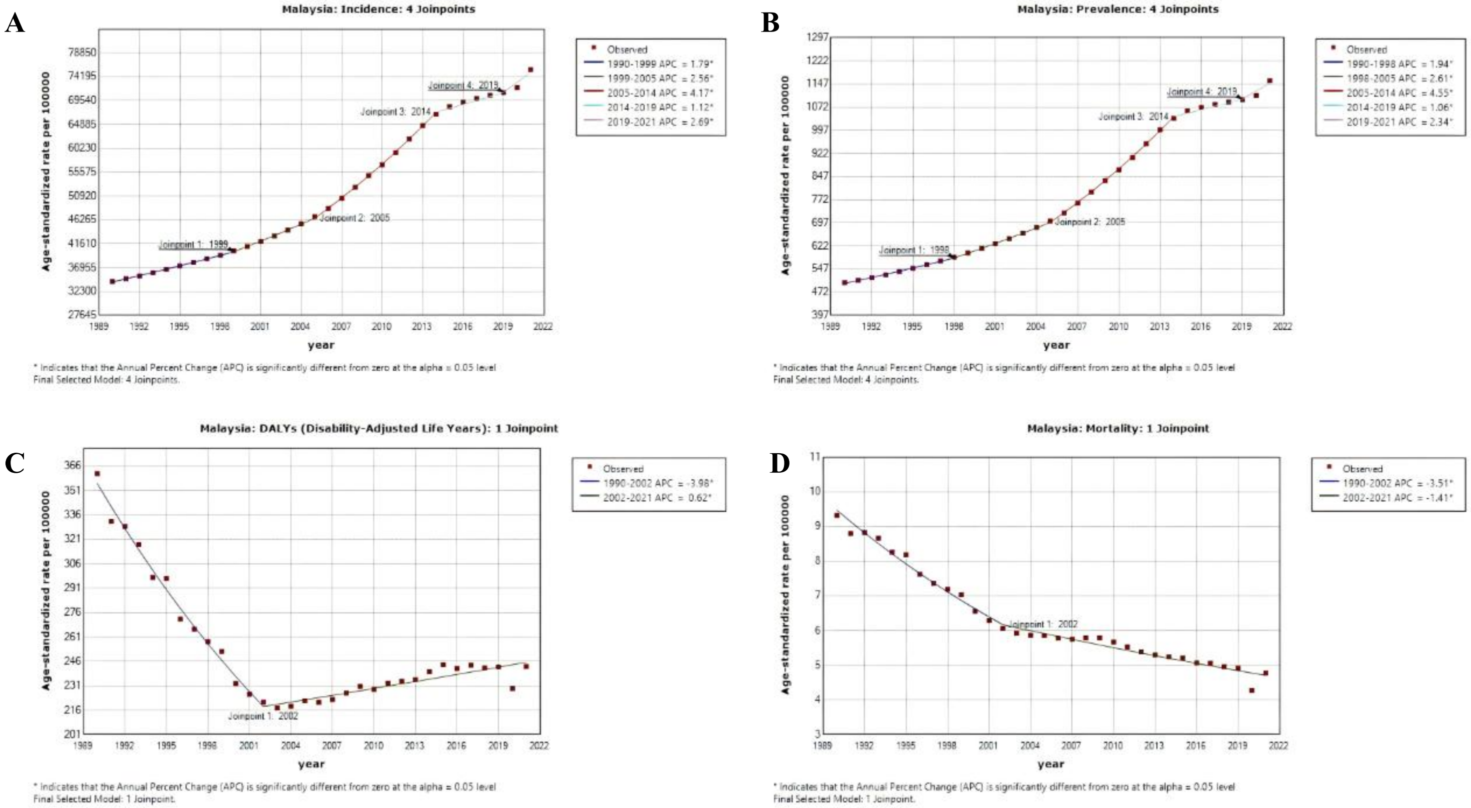

Figure S74: Temporal joinpoint analysis of diarrheal diseases in Malaysia from 1990 to 2021.

(A) Temporal joinpoint analysis of ASIR; (B) Temporal joinpoint analysis of ASPR; (C) Temporal joinpoint analysis of ASDR; (D) Temporal joinpoint analysis of ASMR.

APC, annual percentage change; ASIR, age-standardized incidence rate; ASPR, age-standardized prevalence rate; ASDR, age-standardized DALYs rate; ASMR, age-standardized mortality rate.

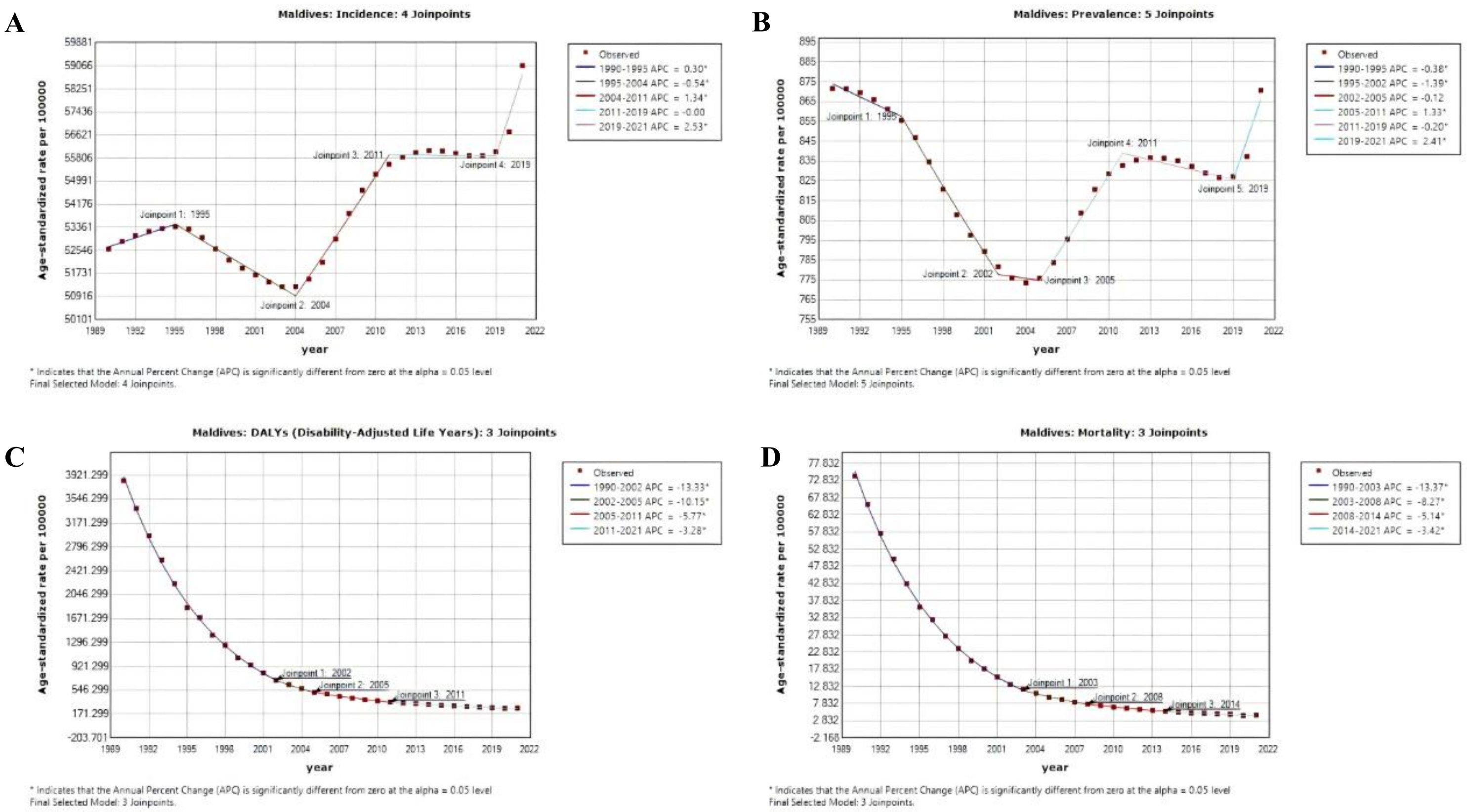

Figure S75: Temporal joinpoint analysis of diarrheal diseases in Maldives from 1990 to 2021.

(A) Temporal joinpoint analysis of ASIR; (B) Temporal joinpoint analysis of ASPR; (C) Temporal joinpoint analysis of ASDR; (D) Temporal joinpoint analysis of ASMR.

APC, annual percentage change; ASIR, age-standardized incidence rate; ASPR, age-standardized prevalence rate; ASDR, age-standardized DALYs rate; ASMR, age-standardized mortality rate.

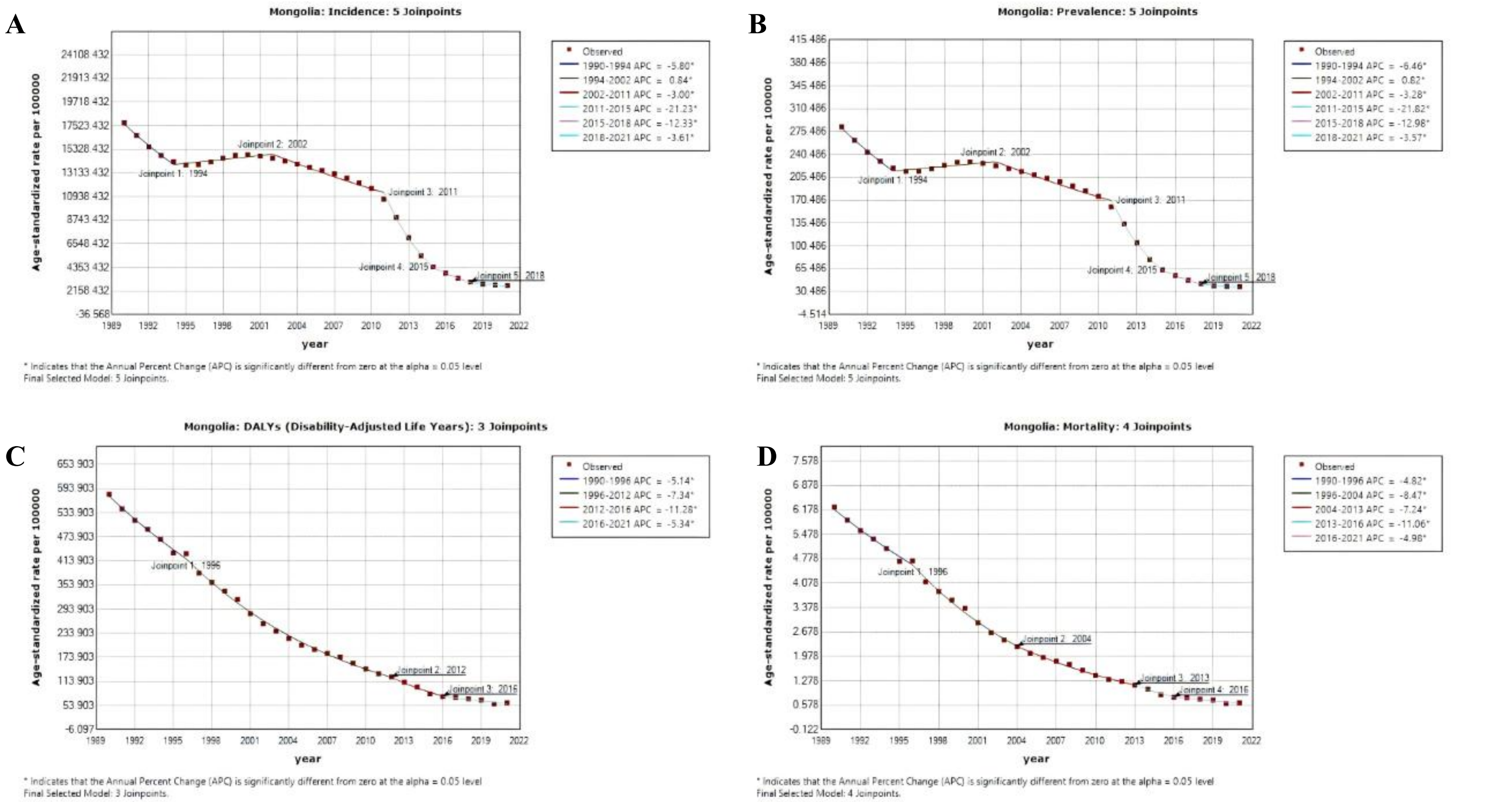

Figure S76: Temporal joinpoint analysis of diarrheal diseases in Mongolia from 1990 to 2021.

(A) Temporal joinpoint analysis of ASIR; (B) Temporal joinpoint analysis of ASPR; (C) Temporal joinpoint analysis of ASDR; (D) Temporal joinpoint analysis of ASMR.

APC, annual percentage change; ASIR, age-standardized incidence rate; ASPR, age-standardized prevalence rate; ASDR, age-standardized DALYs rate; ASMR, age-standardized mortality rate.

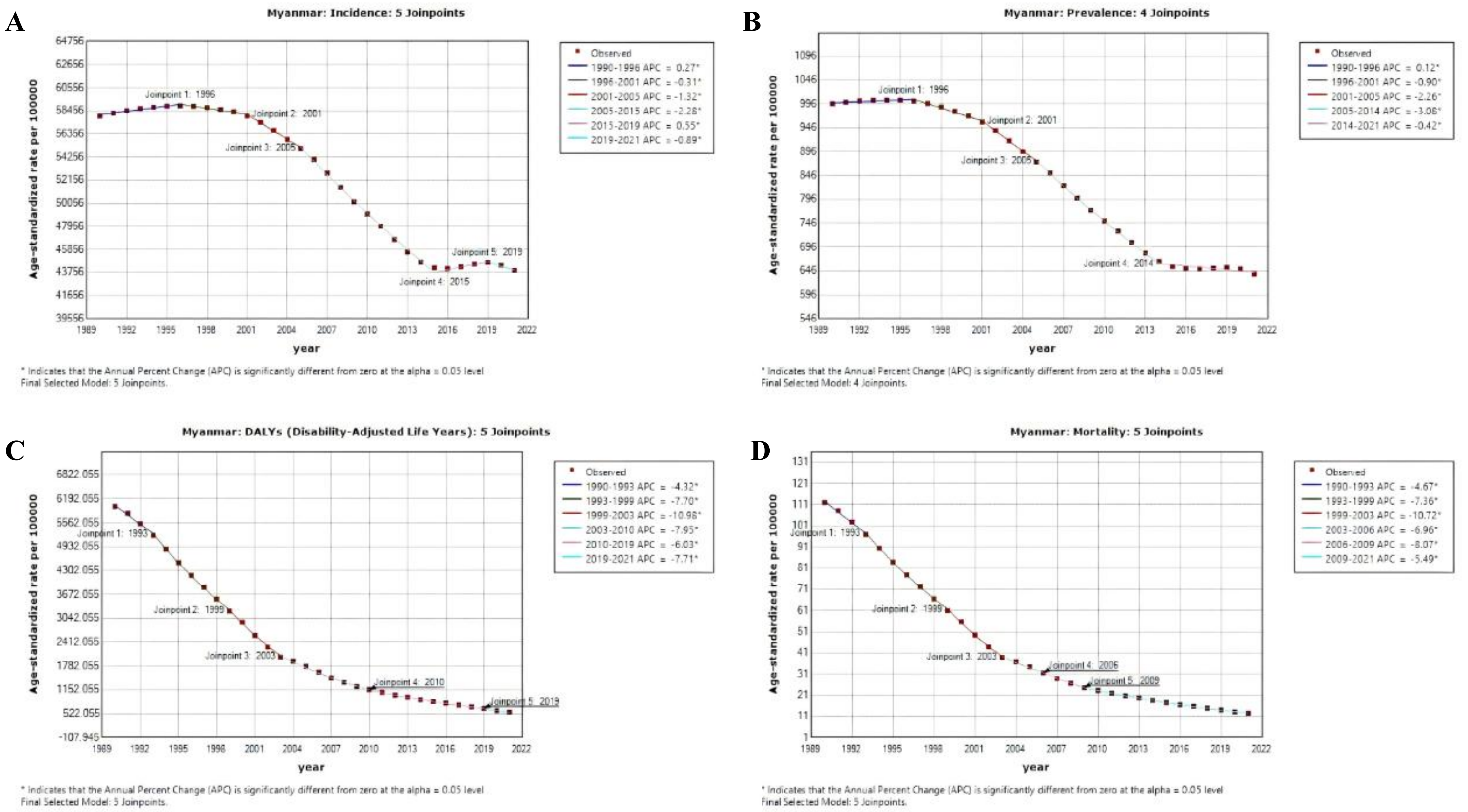

Figure S77: Temporal joinpoint analysis of diarrheal diseases in Myanmar from 1990 to 2021.

(A) Temporal joinpoint analysis of ASIR; (B) Temporal joinpoint analysis of ASPR; (C) Temporal joinpoint analysis of ASDR; (D) Temporal joinpoint analysis of ASMR.

APC, annual percentage change; ASIR, age-standardized incidence rate; ASPR, age-standardized prevalence rate; ASDR, age-standardized DALYs rate; ASMR, age-standardized mortality rate.

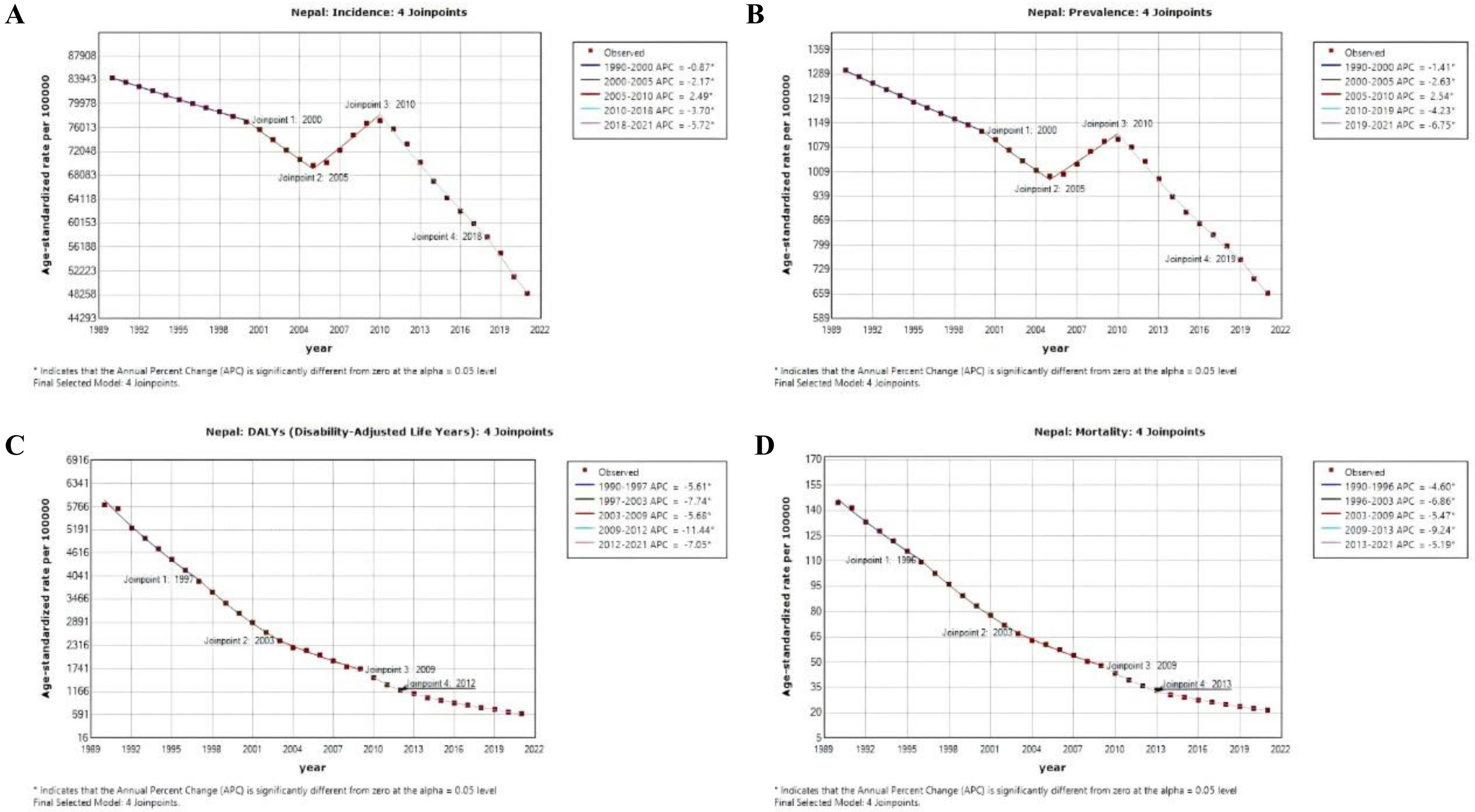

Figure S78: Temporal joinpoint analysis of diarrheal diseases in Nepal from 1990 to 2021.

(A) Temporal joinpoint analysis of ASIR; (B) Temporal joinpoint analysis of ASPR; (C) Temporal joinpoint analysis of ASDR; (D) Temporal joinpoint analysis of ASMR.

APC, annual percentage change; ASIR, age-standardized incidence rate; ASPR, age-standardized prevalence rate; ASDR, age-standardized DALYs rate; ASMR, age-standardized mortality rate.

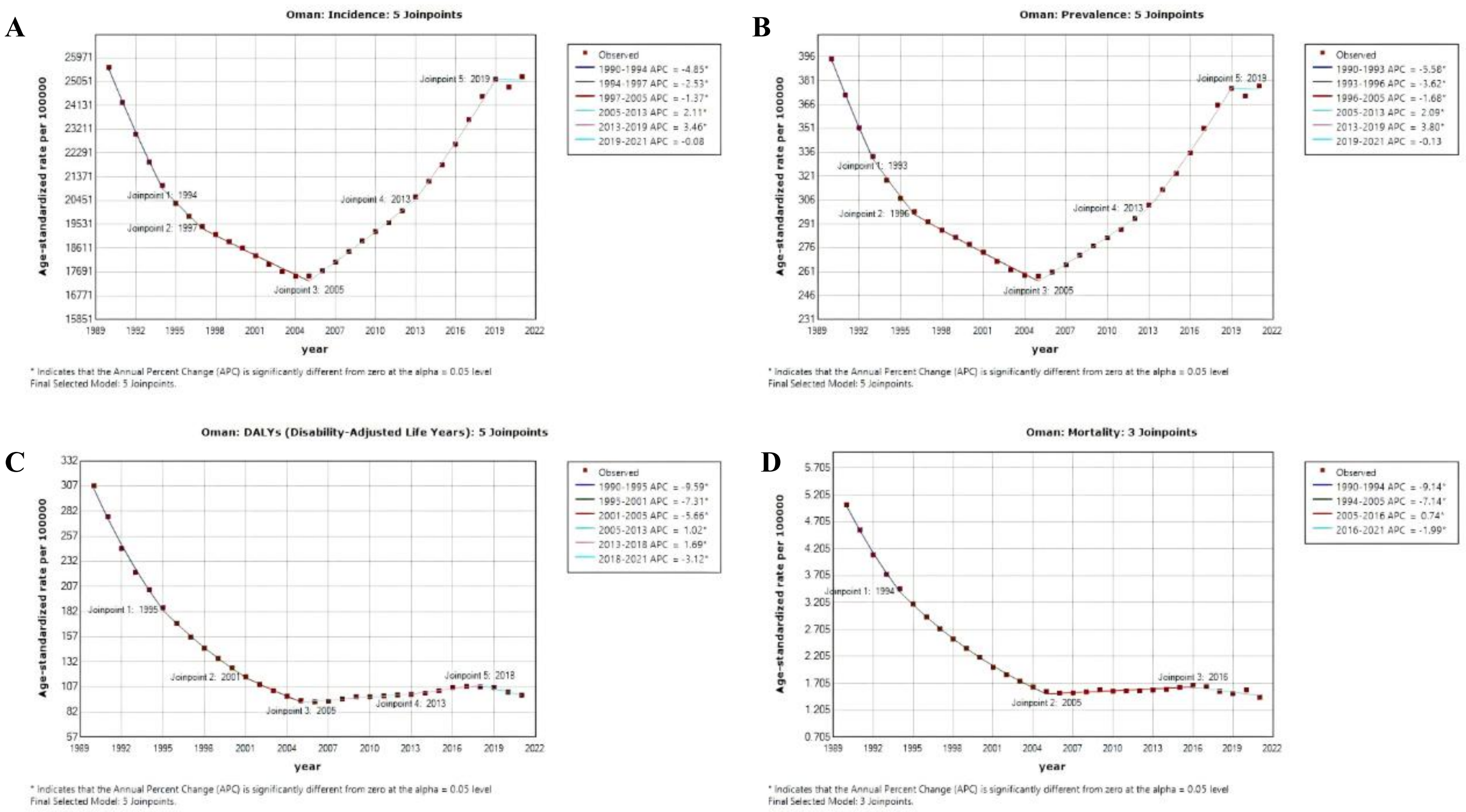

Figure S79: Temporal joinpoint analysis of diarrheal diseases in Oman from 1990 to 2021.

(A) Temporal joinpoint analysis of ASIR; (B) Temporal joinpoint analysis of ASPR; (C) Temporal joinpoint analysis of ASDR; (D) Temporal joinpoint analysis of ASMR.

APC, annual percentage change; ASIR, age-standardized incidence rate; ASPR, age-standardized prevalence rate; ASDR, age-standardized DALYs rate; ASMR, age-standardized mortality rate.

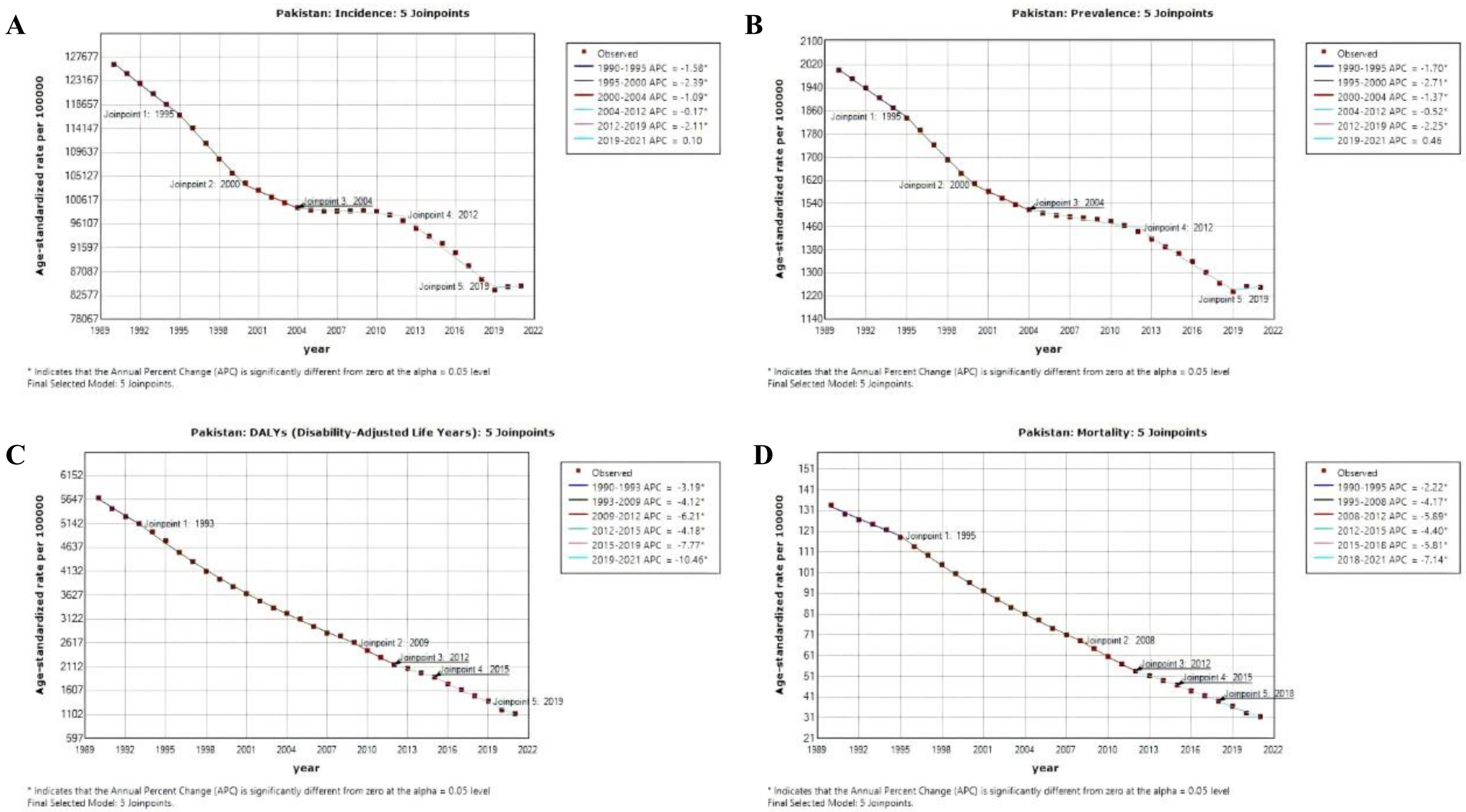

Figure S80: Temporal joinpoint analysis of diarrheal diseases in Pakistan from 1990 to 2021.

(A) Temporal joinpoint analysis of ASIR; (B) Temporal joinpoint analysis of ASPR; (C) Temporal joinpoint analysis of ASDR; (D) Temporal joinpoint analysis of ASMR.

APC, annual percentage change; ASIR, age-standardized incidence rate; ASPR, age-standardized prevalence rate; ASDR, age-standardized DALYs rate; ASMR, age-standardized mortality rate.

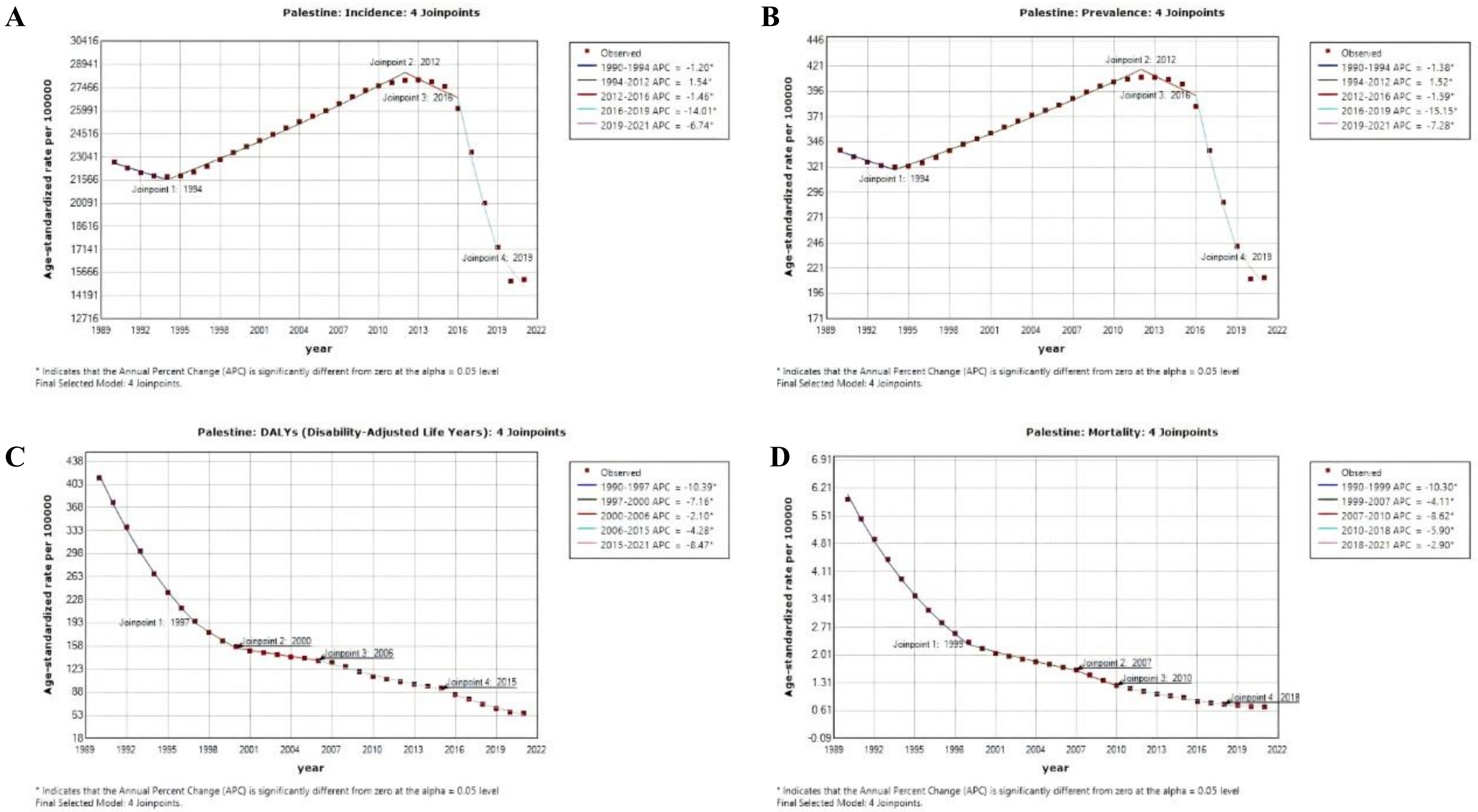

Figure S81: Temporal joinpoint analysis of diarrheal diseases in Palestine from 1990 to 2021.

(A) Temporal joinpoint analysis of ASIR; (B) Temporal joinpoint analysis of ASPR; (C) Temporal joinpoint analysis of ASDR; (D) Temporal joinpoint analysis of ASMR.

APC, annual percentage change; ASIR, age-standardized incidence rate; ASPR, age-standardized prevalence rate; ASDR, age-standardized DALYs rate; ASMR, age-standardized mortality rate.

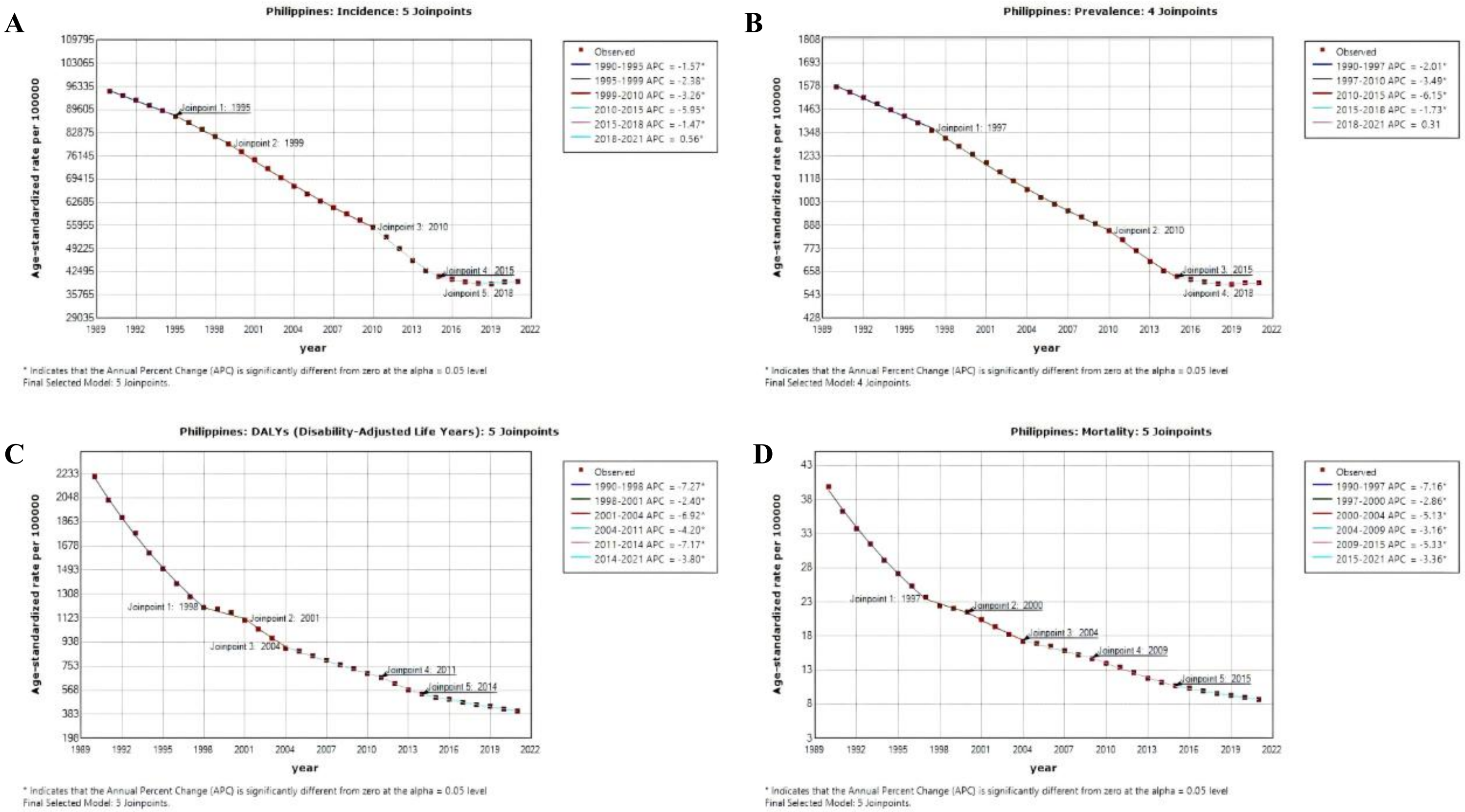

Figure S82: Temporal joinpoint analysis of diarrheal diseases in Philippines from 1990 to 2021.

(A) Temporal joinpoint analysis of ASIR; (B) Temporal joinpoint analysis of ASPR; (C) Temporal joinpoint analysis of ASDR; (D) Temporal joinpoint analysis of ASMR.

APC, annual percentage change; ASIR, age-standardized incidence rate; ASPR, age-standardized prevalence rate; ASDR, age-standardized DALYs rate; ASMR, age-standardized mortality rate.

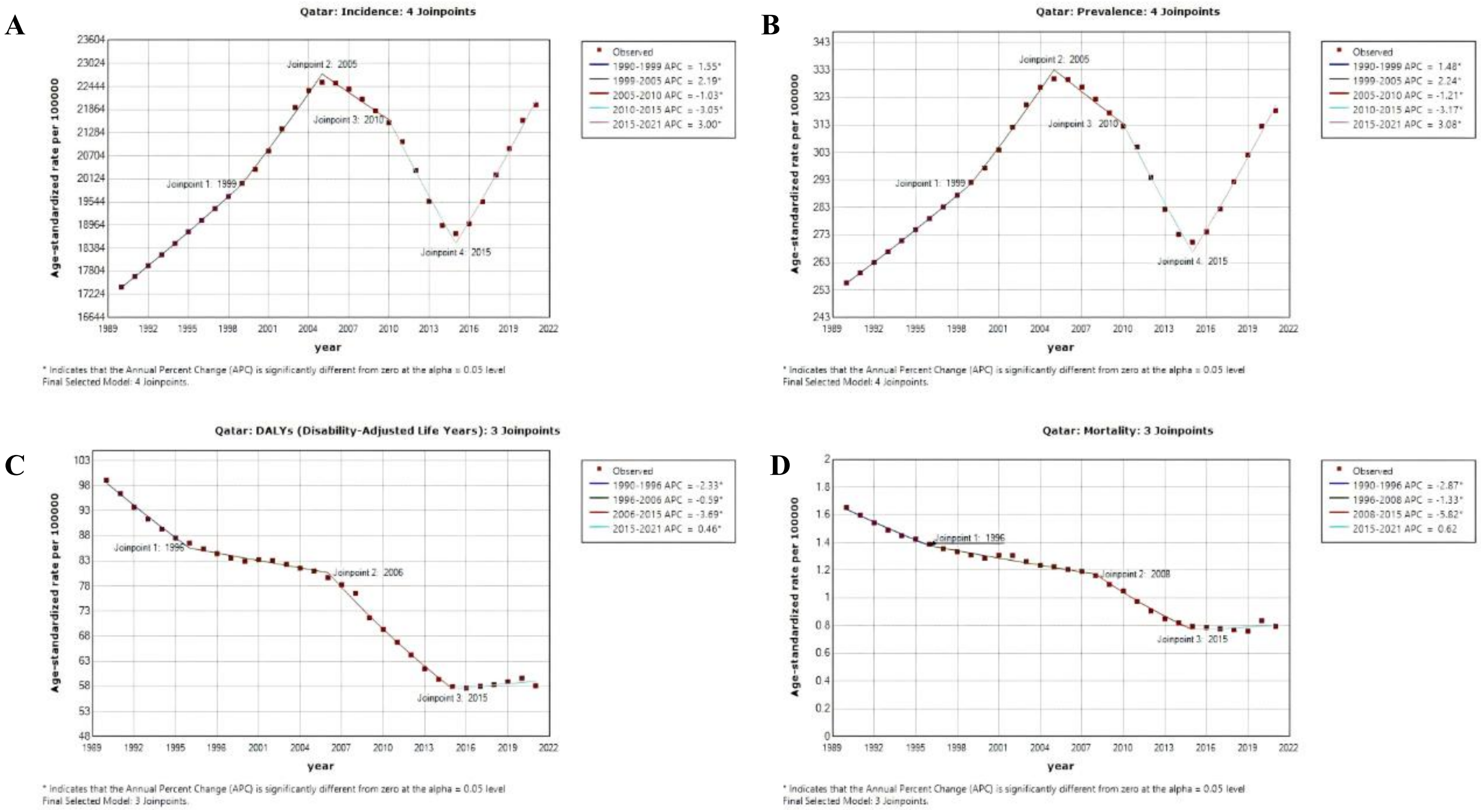

Figure S83: Temporal joinpoint analysis of diarrheal diseases in Qatar from 1990 to 2021.

(A) Temporal joinpoint analysis of ASIR; (B) Temporal joinpoint analysis of ASPR; (C) Temporal joinpoint analysis of ASDR; (D) Temporal joinpoint analysis of ASMR.

APC, annual percentage change; ASIR, age-standardized incidence rate; ASPR, age-standardized prevalence rate; ASDR, age-standardized DALYs rate; ASMR, age-standardized mortality rate.

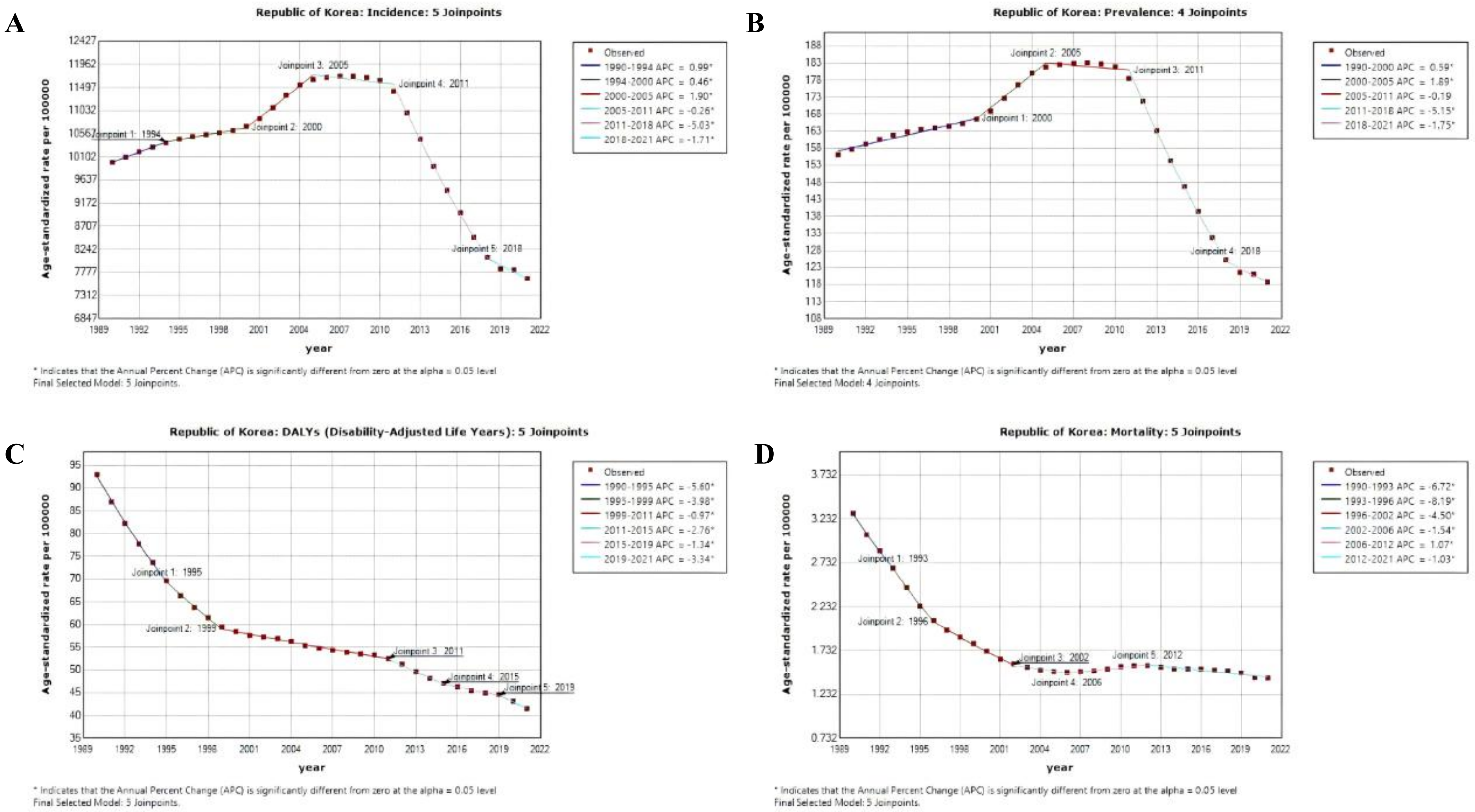

Figure S84: Temporal joinpoint analysis of diarrheal diseases in Republic of Korea from 1990 to 2021.

(A) Temporal joinpoint analysis of ASIR; (B) Temporal joinpoint analysis of ASPR; (C) Temporal joinpoint analysis of ASDR; (D) Temporal joinpoint analysis of ASMR.

APC, annual percentage change; ASIR, age-standardized incidence rate; ASPR, age-standardized prevalence rate; ASDR, age-standardized DALYs rate; ASMR, age-standardized mortality rate.

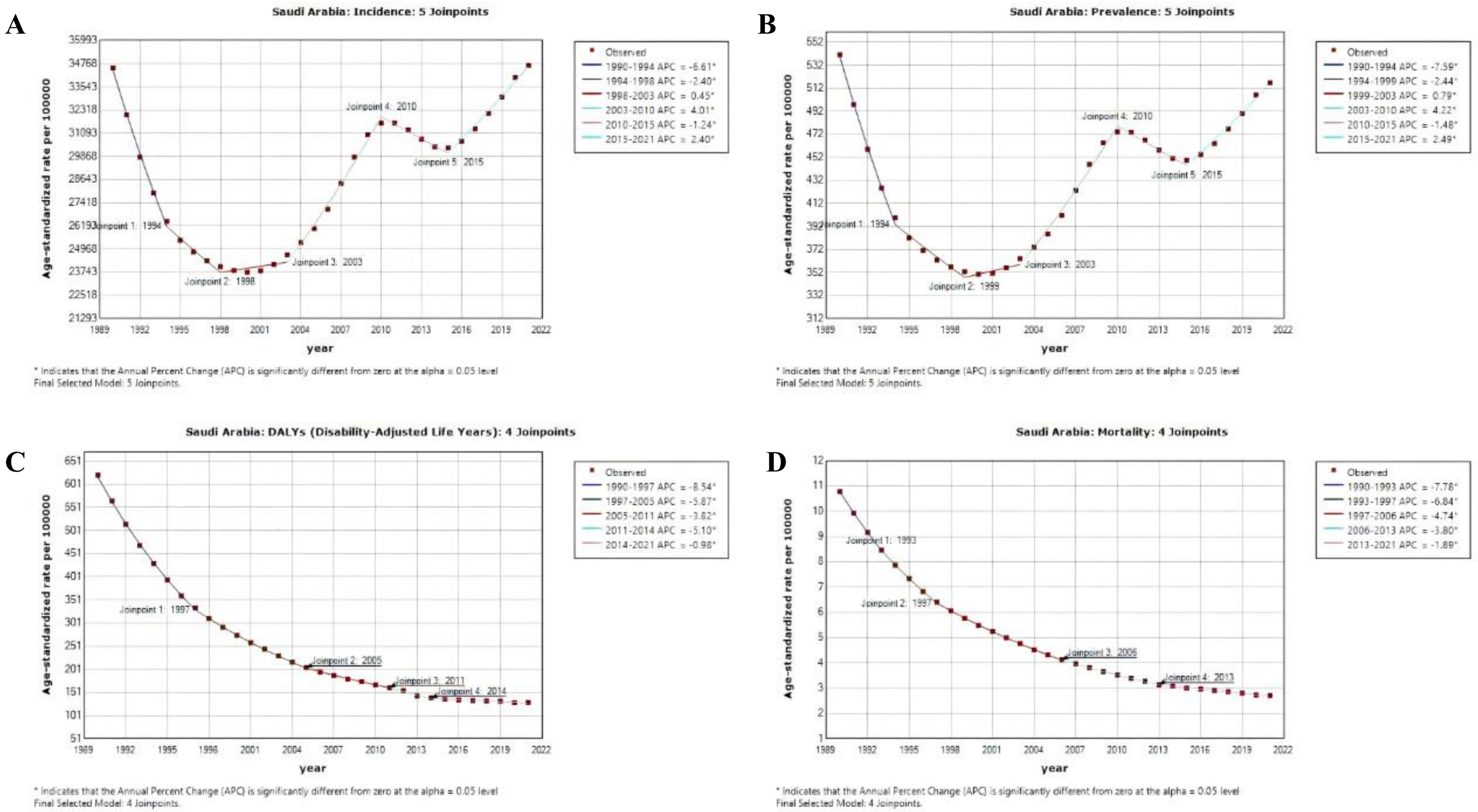

Figure S85: Temporal joinpoint analysis of diarrheal diseases in Saudi Arabia from 1990 to 2021.

(A) Temporal joinpoint analysis of ASIR; (B) Temporal joinpoint analysis of ASPR; (C) Temporal joinpoint analysis of ASDR; (D) Temporal joinpoint analysis of ASMR.

APC, annual percentage change; ASIR, age-standardized incidence rate; ASPR, age-standardized prevalence rate; ASDR, age-standardized DALYs rate; ASMR, age-standardized mortality rate.

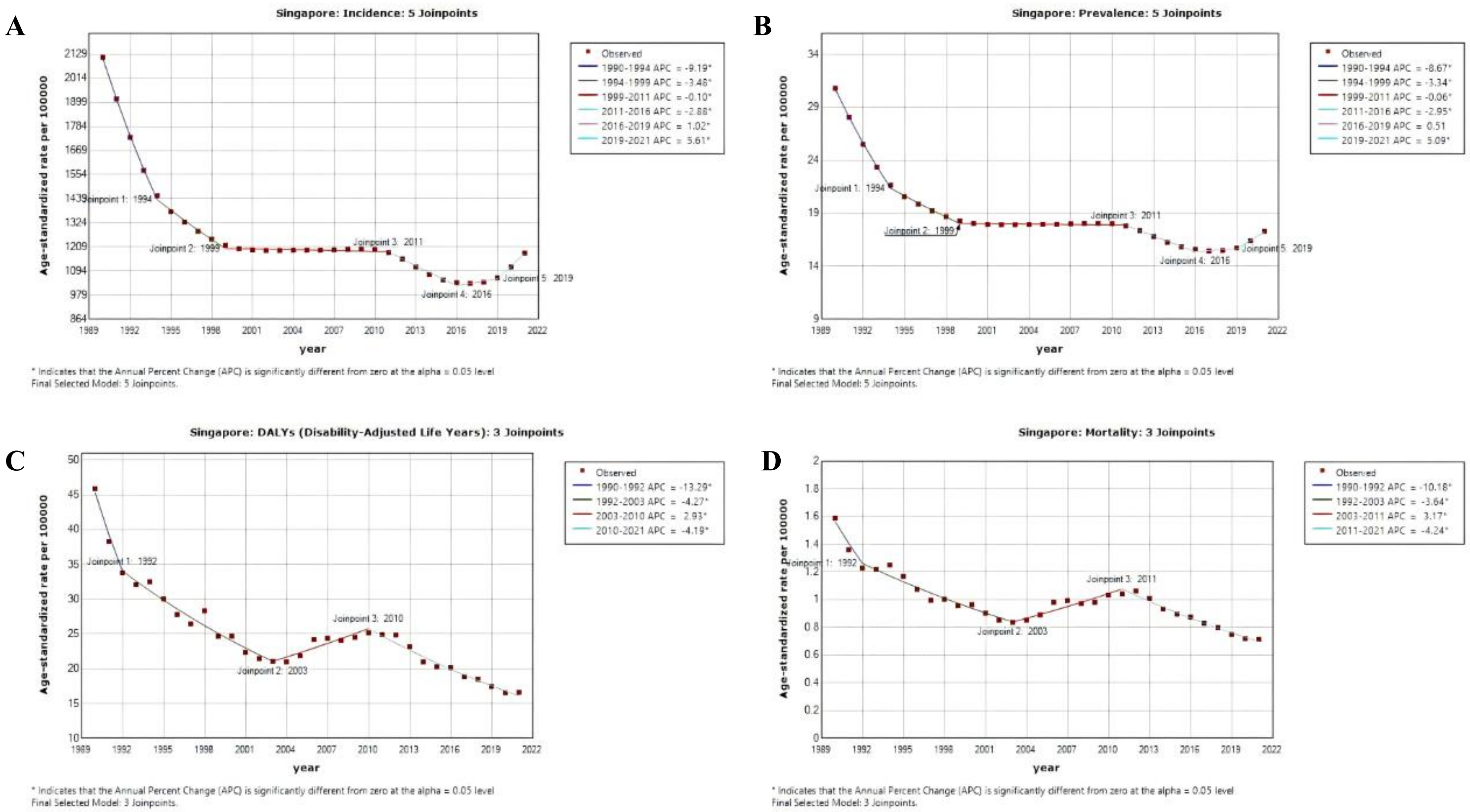

Figure S86: Temporal joinpoint analysis of diarrheal diseases in Singapore from 1990 to 2021.

(A) Temporal joinpoint analysis of ASIR; (B) Temporal joinpoint analysis of ASPR; (C) Temporal joinpoint analysis of ASDR; (D) Temporal joinpoint analysis of ASMR.

APC, annual percentage change; ASIR, age-standardized incidence rate; ASPR, age-standardized prevalence rate; ASDR, age-standardized DALYs rate; ASMR, age-standardized mortality rate.

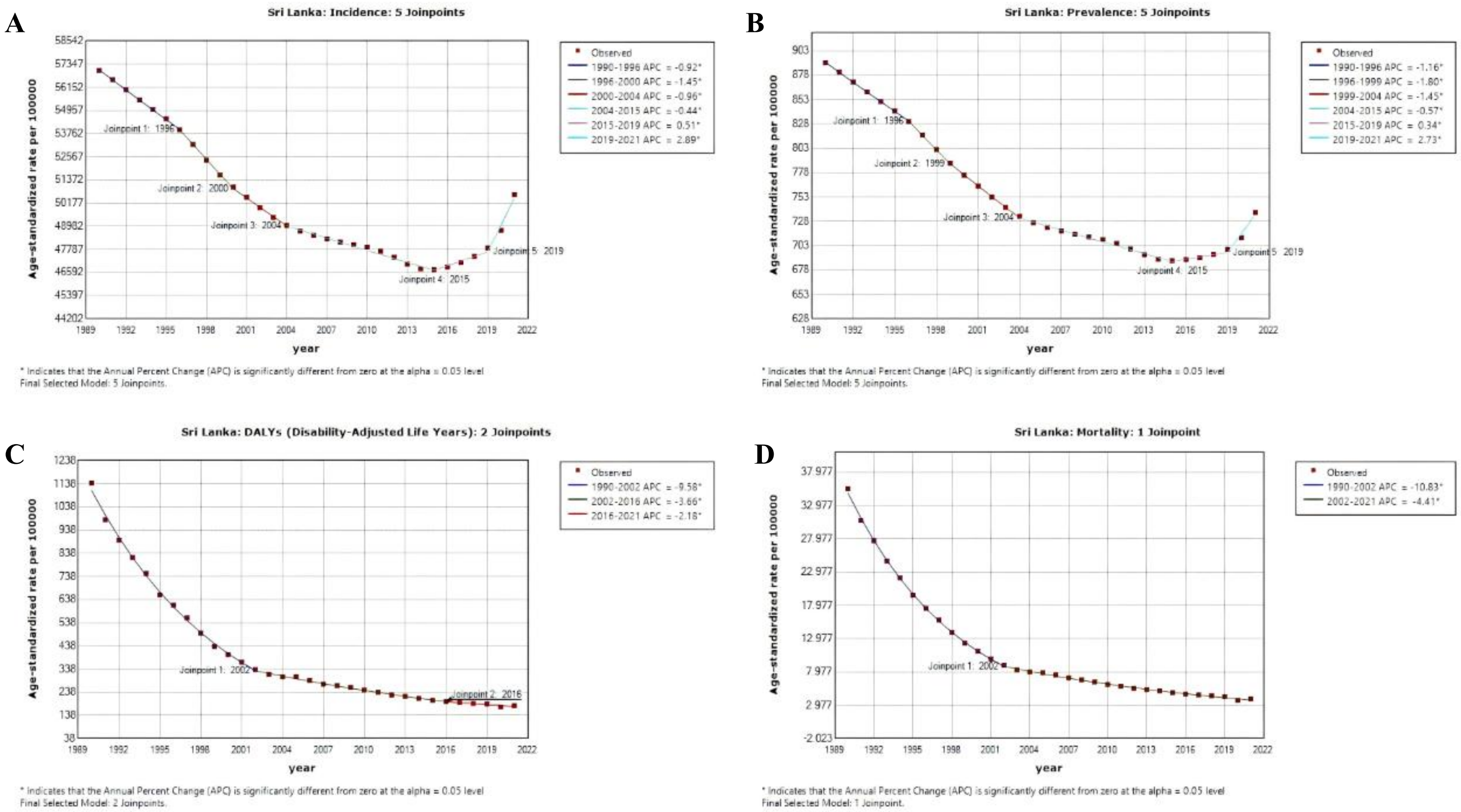

Figure S87: Temporal joinpoint analysis of diarrheal diseases in Sri Lanka from 1990 to 2021.

(A) Temporal joinpoint analysis of ASIR; (B) Temporal joinpoint analysis of ASPR; (C) Temporal joinpoint analysis of ASDR; (D) Temporal joinpoint analysis of ASMR.

APC, annual percentage change; ASIR, age-standardized incidence rate; ASPR, age-standardized prevalence rate; ASDR, age-standardized DALYs rate; ASMR, age-standardized mortality rate.

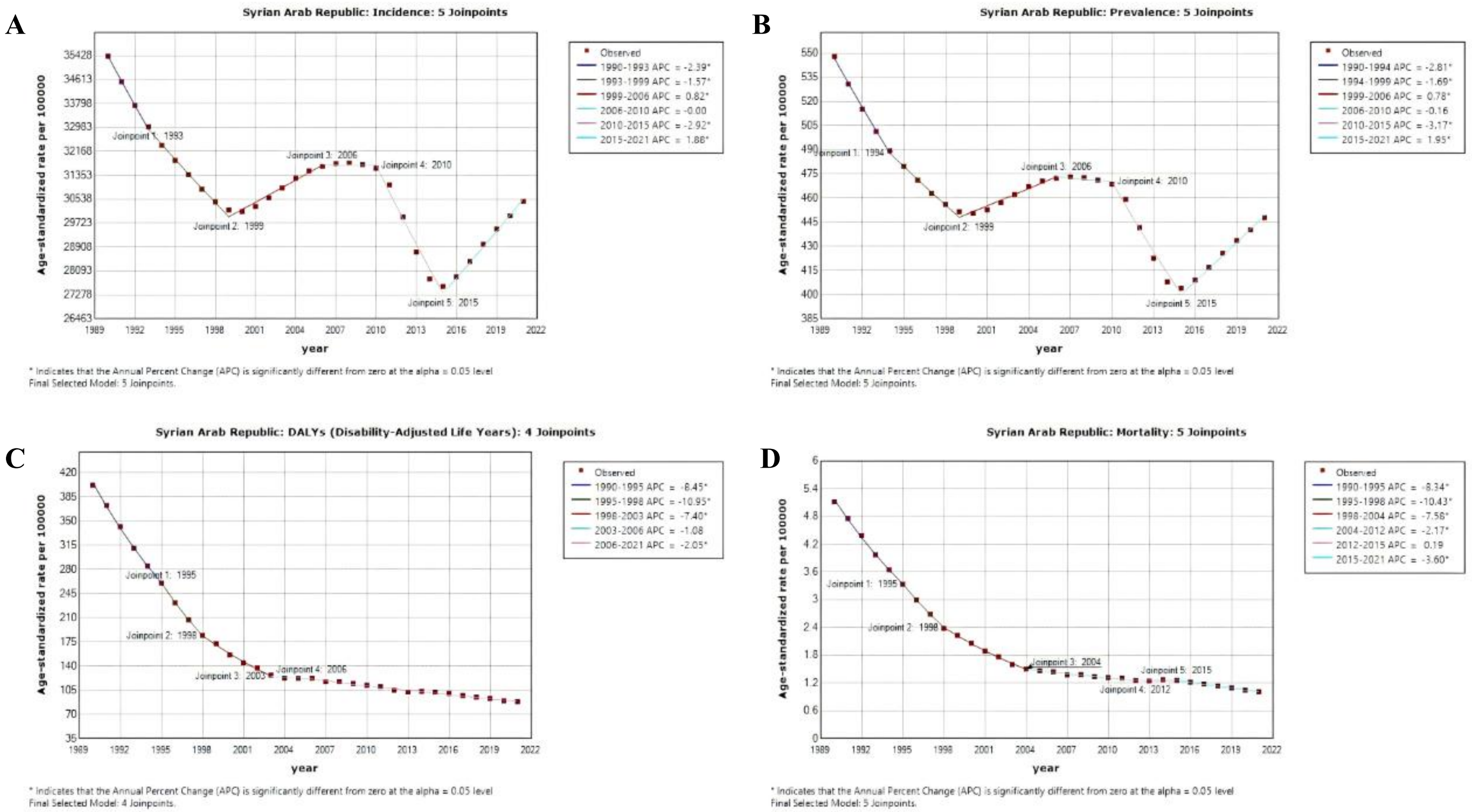

Figure S88: Temporal joinpoint analysis of diarrheal diseases in Syrian Arab Republic from 1990 to 2021.

(A) Temporal joinpoint analysis of ASIR; (B) Temporal joinpoint analysis of ASPR; (C) Temporal joinpoint analysis of ASDR; (D) Temporal joinpoint analysis of ASMR.

APC, annual percentage change; ASIR, age-standardized incidence rate; ASPR, age-standardized prevalence rate; ASDR, age-standardized DALYs rate; ASMR, age-standardized mortality rate.

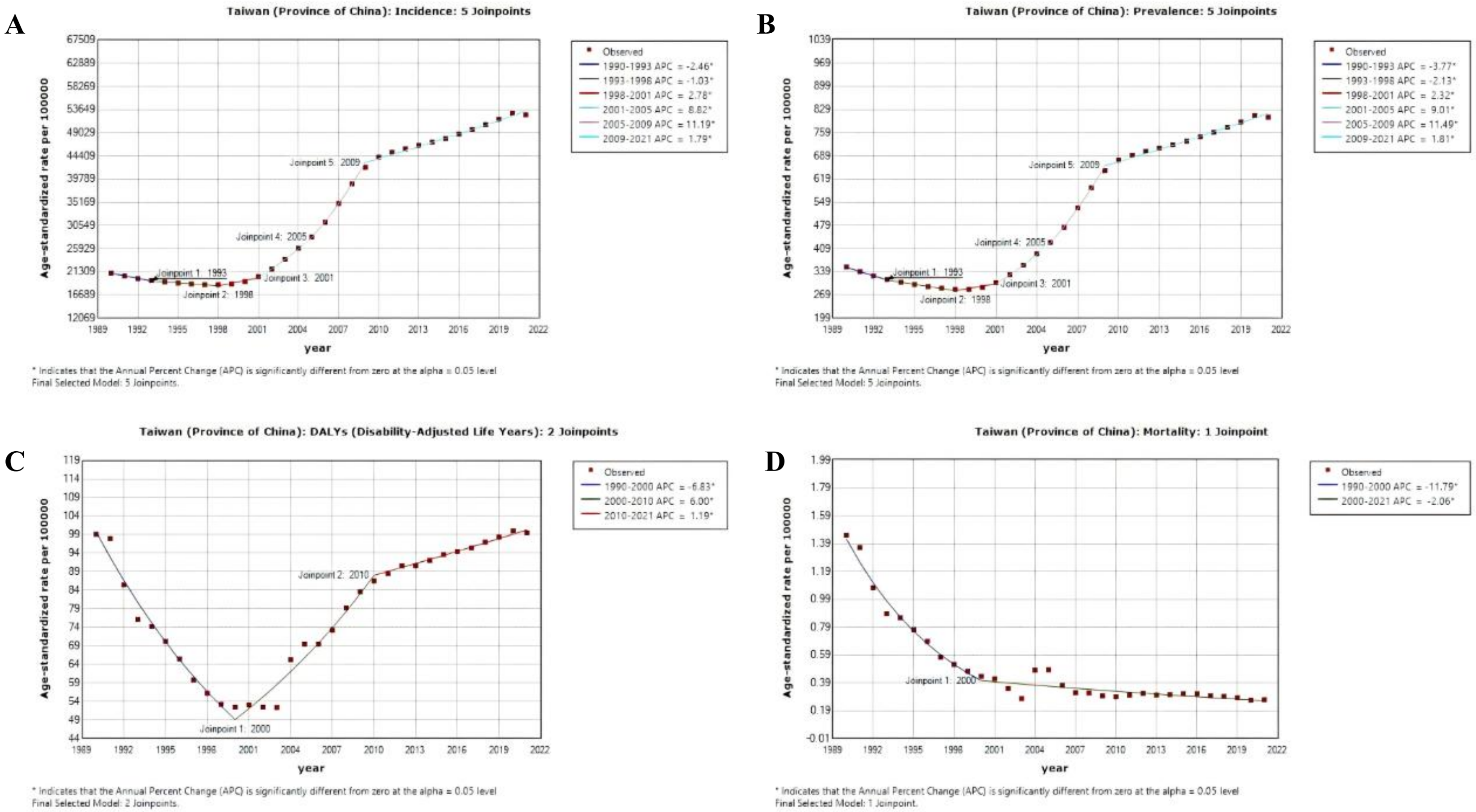

Figure S89: Temporal joinpoint analysis of diarrheal diseases in Taiwan (Province of China) from 1990 to 2021.

(A) Temporal joinpoint analysis of ASIR; (B) Temporal joinpoint analysis of ASPR; (C) Temporal joinpoint analysis of ASDR; (D) Temporal joinpoint analysis of ASMR.

APC, annual percentage change; ASIR, age-standardized incidence rate; ASPR, age-standardized prevalence rate; ASDR, age-standardized DALYs rate; ASMR, age-standardized mortality rate.

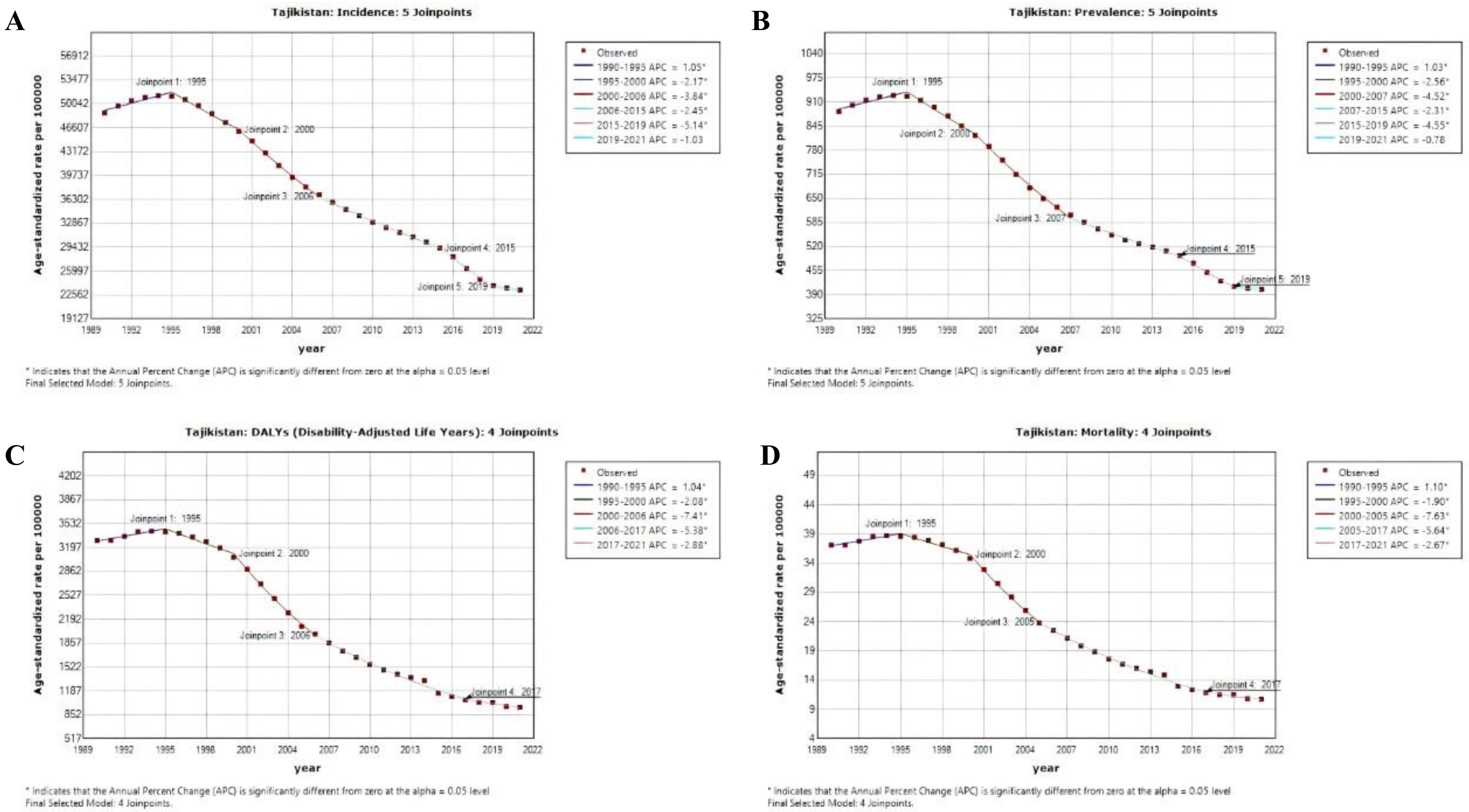

Figure S90: Temporal joinpoint analysis of diarrheal diseases in Tajikistan from 1990 to 2021.

(A) Temporal joinpoint analysis of ASIR; (B) Temporal joinpoint analysis of ASPR; (C) Temporal joinpoint analysis of ASDR; (D) Temporal joinpoint analysis of ASMR.

APC, annual percentage change; ASIR, age-standardized incidence rate; ASPR, age-standardized prevalence rate; ASDR, age-standardized DALYs rate; ASMR, age-standardized mortality rate.

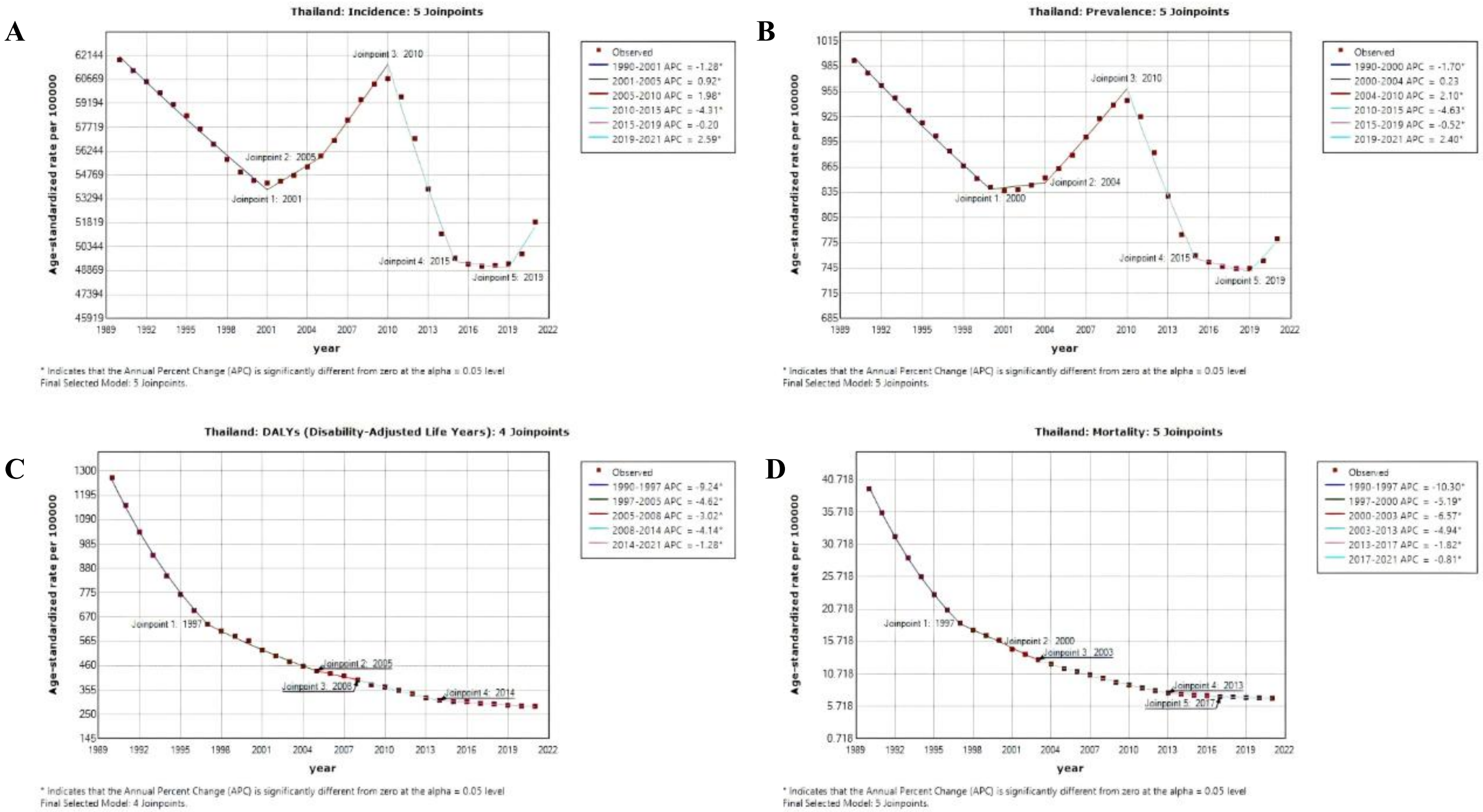

Figure S91: Temporal joinpoint analysis of diarrheal diseases in Thailand from 1990 to 2021.

(A) Temporal joinpoint analysis of ASIR; (B) Temporal joinpoint analysis of ASPR; (C) Temporal joinpoint analysis of ASDR; (D) Temporal joinpoint analysis of ASMR.

APC, annual percentage change; ASIR, age-standardized incidence rate; ASPR, age-standardized prevalence rate; ASDR, age-standardized DALYs rate; ASMR, age-standardized mortality rate.

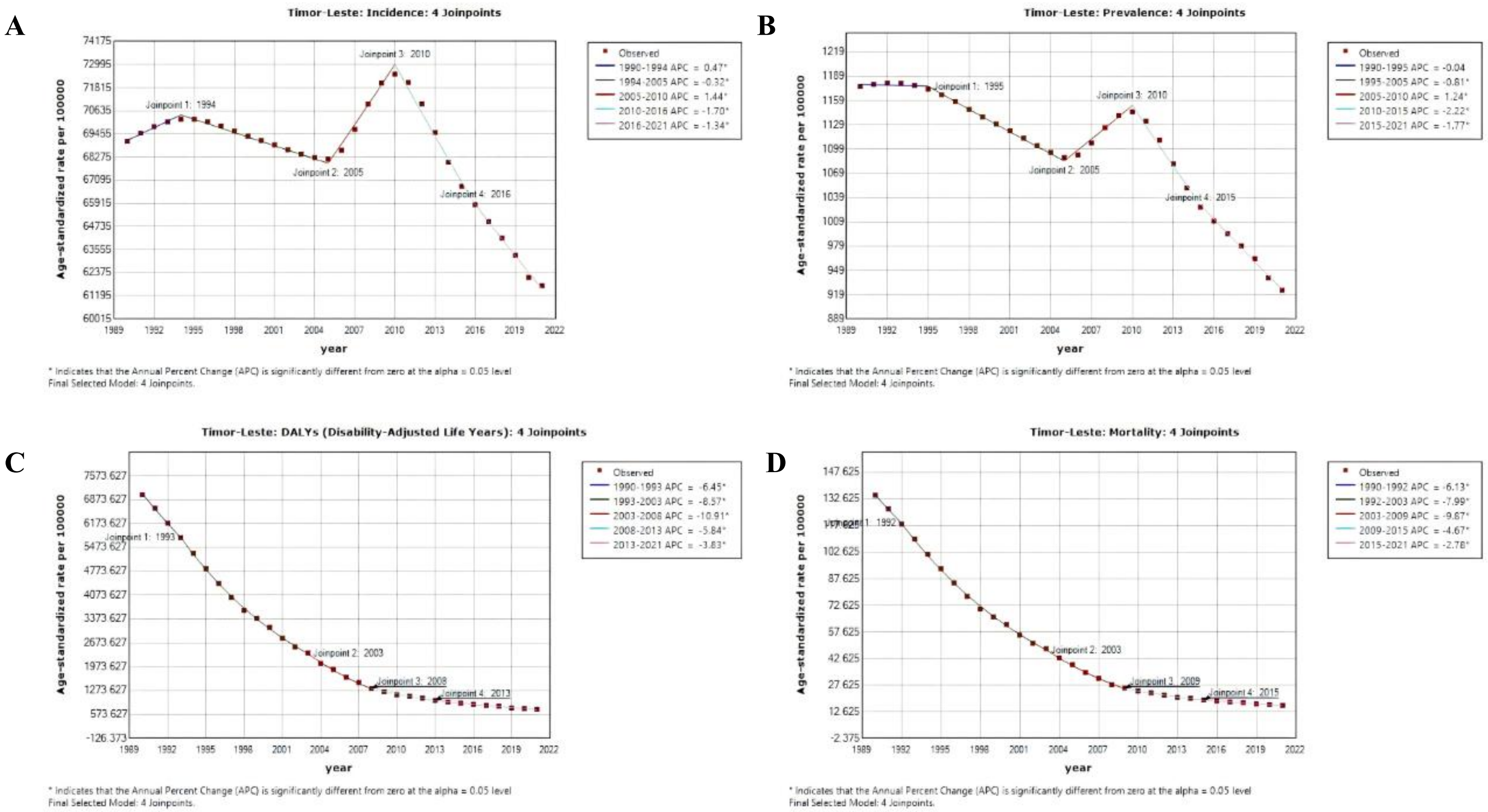

Figure S92: Temporal joinpoint analysis of diarrheal diseases in Timor-Leste from 1990 to 2021.

(A) Temporal joinpoint analysis of ASIR; (B) Temporal joinpoint analysis of ASPR; (C) Temporal joinpoint analysis of ASDR; (D) Temporal joinpoint analysis of ASMR.

APC, annual percentage change; ASIR, age-standardized incidence rate; ASPR, age-standardized prevalence rate; ASDR, age-standardized DALYs rate; ASMR, age-standardized mortality rate.

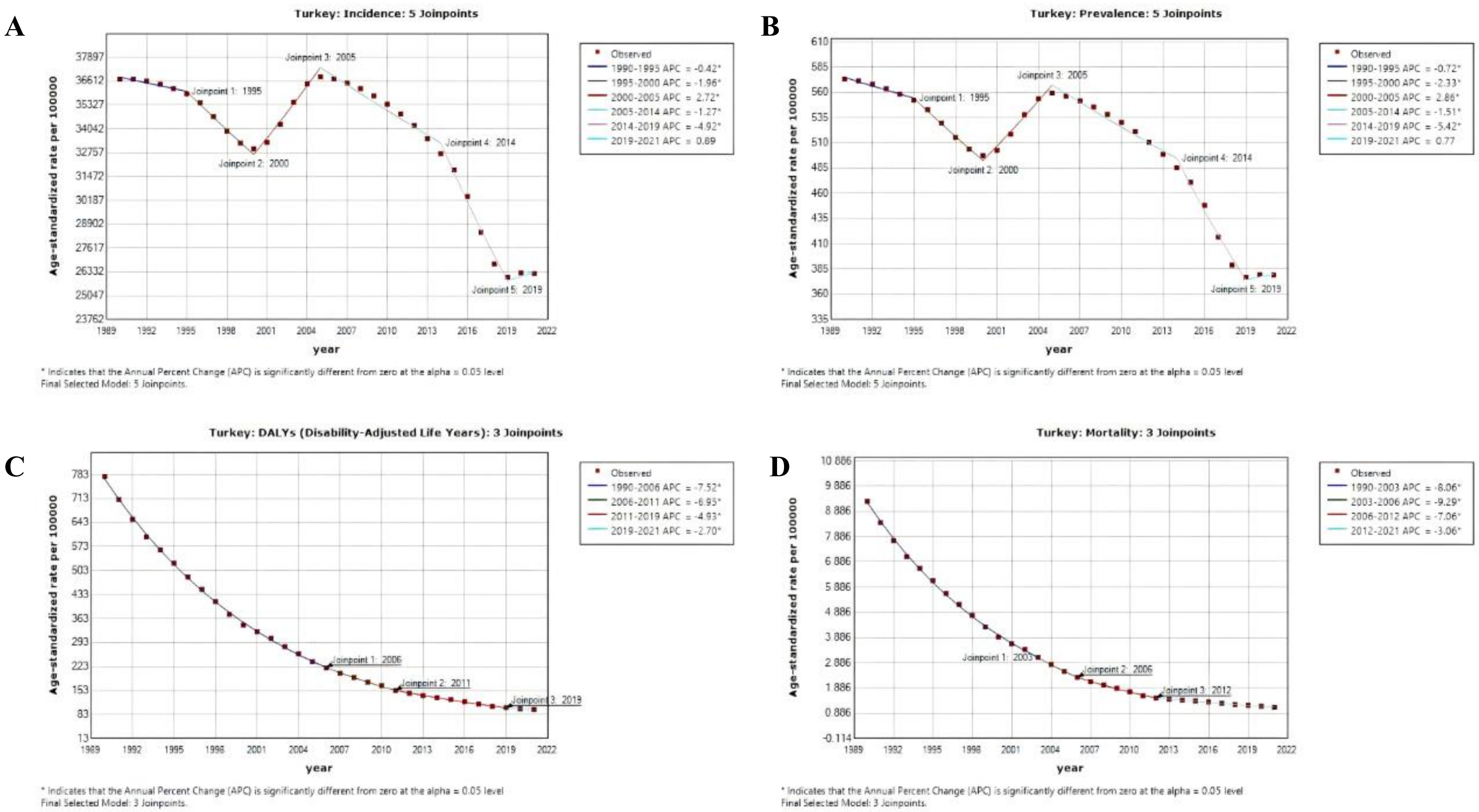

Figure S93: Temporal joinpoint analysis of diarrheal diseases in Turkey from 1990 to 2021.

(A) Temporal joinpoint analysis of ASIR; (B) Temporal joinpoint analysis of ASPR; (C) Temporal joinpoint analysis of ASDR; (D) Temporal joinpoint analysis of ASMR.

APC, annual percentage change; ASIR, age-standardized incidence rate; ASPR, age-standardized prevalence rate; ASDR, age-standardized DALYs rate; ASMR, age-standardized mortality rate.

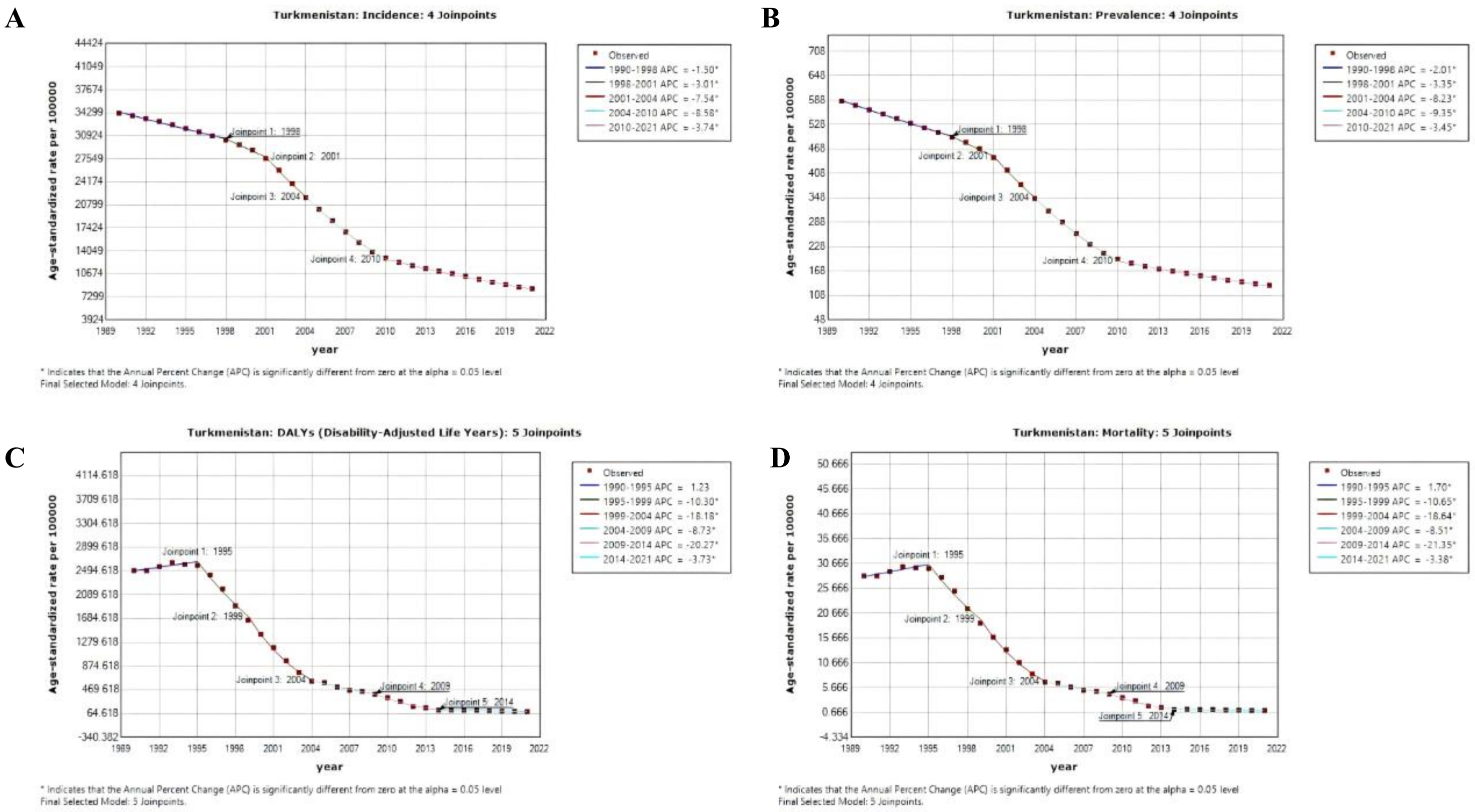

Figure S94: Temporal joinpoint analysis of diarrheal diseases in Turkmenistan from 1990 to 2021.

(A) Temporal joinpoint analysis of ASIR; (B) Temporal joinpoint analysis of ASPR; (C) Temporal joinpoint analysis of ASDR; (D) Temporal joinpoint analysis of ASMR.

APC, annual percentage change; ASIR, age-standardized incidence rate; ASPR, age-standardized prevalence rate; ASDR, age-standardized DALYs rate; ASMR, age-standardized mortality rate.

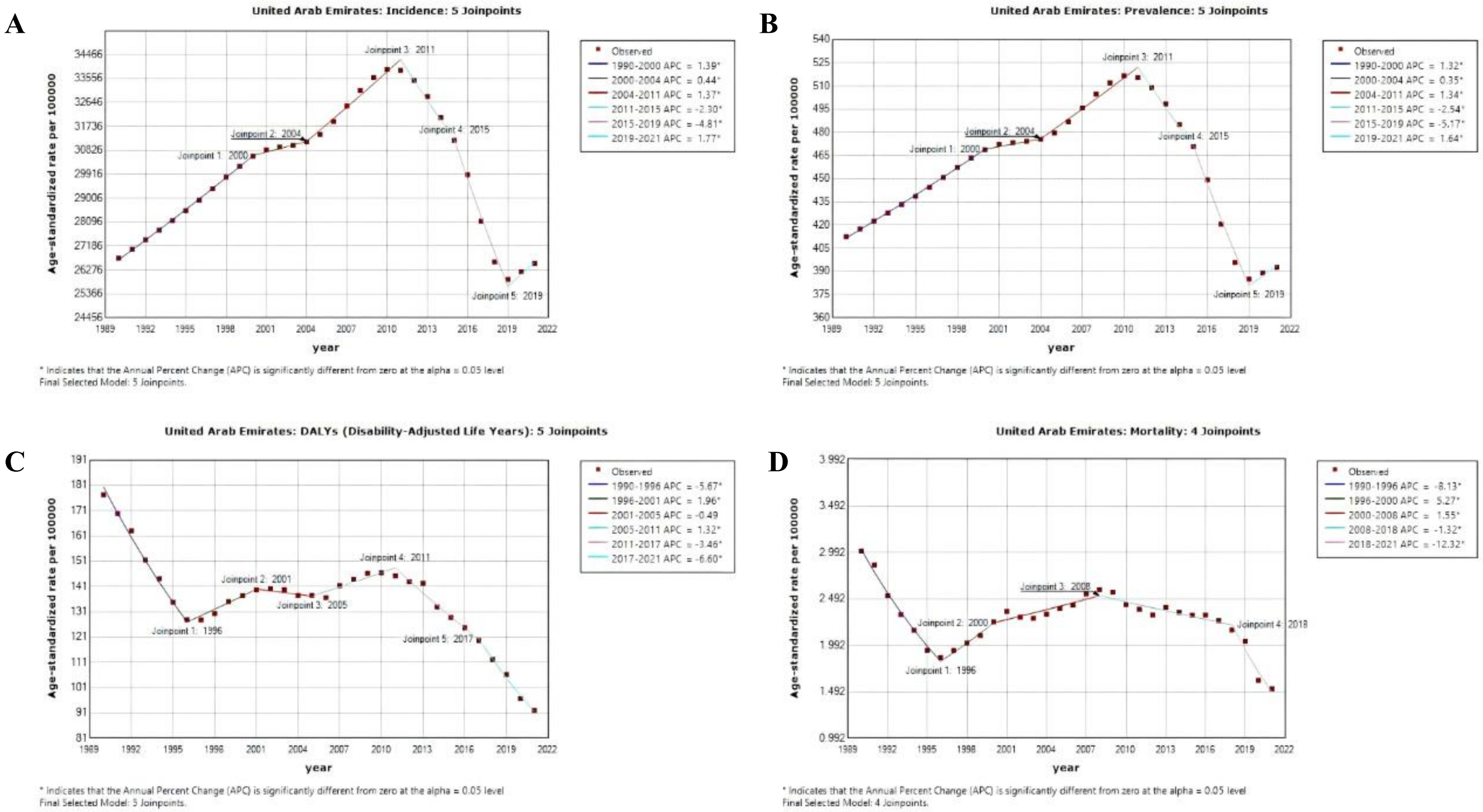

Figure S95: Temporal joinpoint analysis of diarrheal diseases in United Arab Emirates from 1990 to 2021.

(A) Temporal joinpoint analysis of ASIR; (B) Temporal joinpoint analysis of ASPR; (C) Temporal joinpoint analysis of ASDR; (D) Temporal joinpoint analysis of ASMR.

APC, annual percentage change; ASIR, age-standardized incidence rate; ASPR, age-standardized prevalence rate; ASDR, age-standardized DALYs rate; ASMR, age-standardized mortality rate.

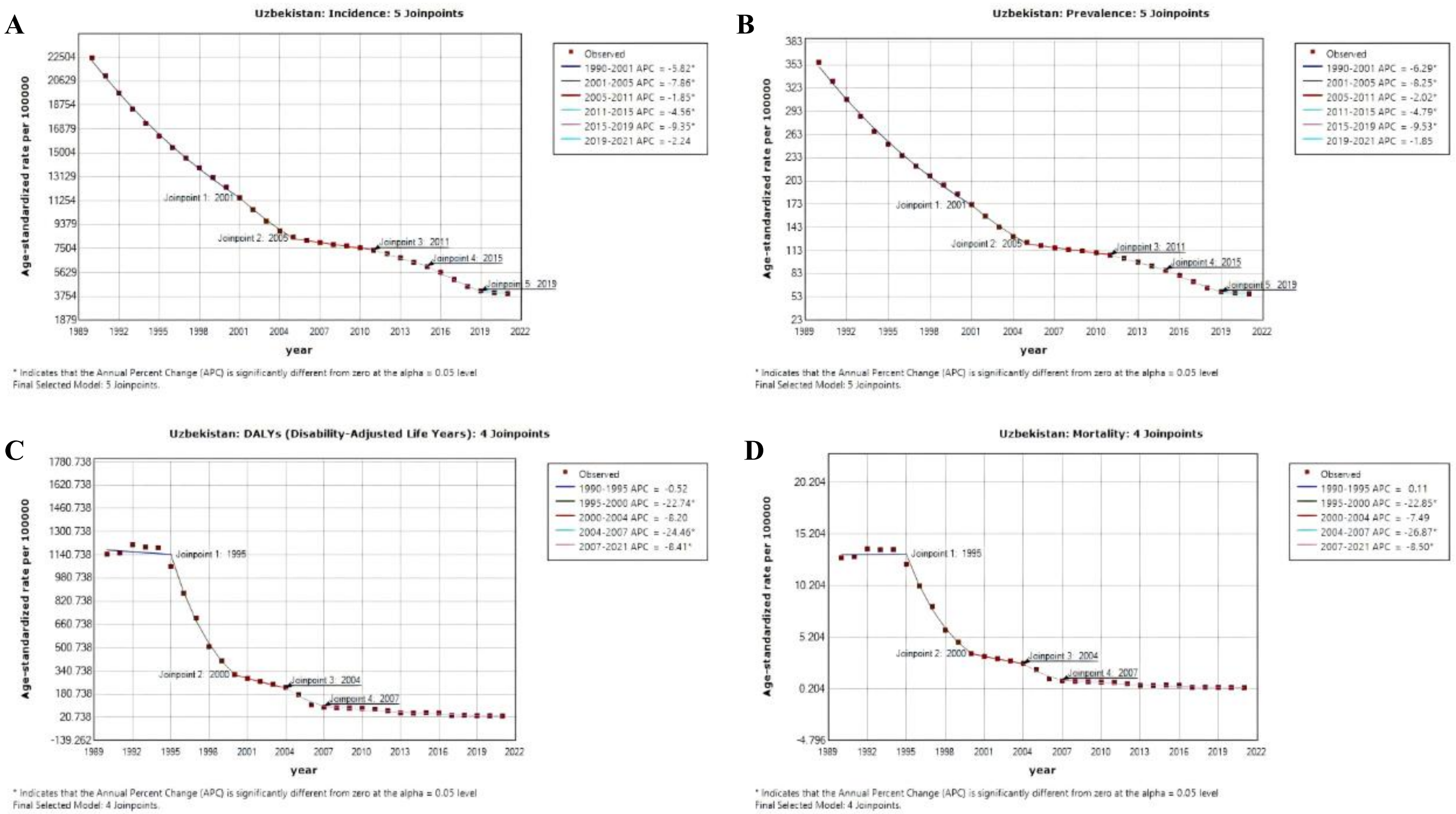

Figure S96: Temporal joinpoint analysis of diarrheal diseases in Uzbekistan from 1990 to 2021.

(A) Temporal joinpoint analysis of ASIR; (B) Temporal joinpoint analysis of ASPR; (C) Temporal joinpoint analysis of ASDR; (D) Temporal joinpoint analysis of ASMR.

APC, annual percentage change; ASIR, age-standardized incidence rate; ASPR, age-standardized prevalence rate; ASDR, age-standardized DALYs rate; ASMR, age-standardized mortality rate.

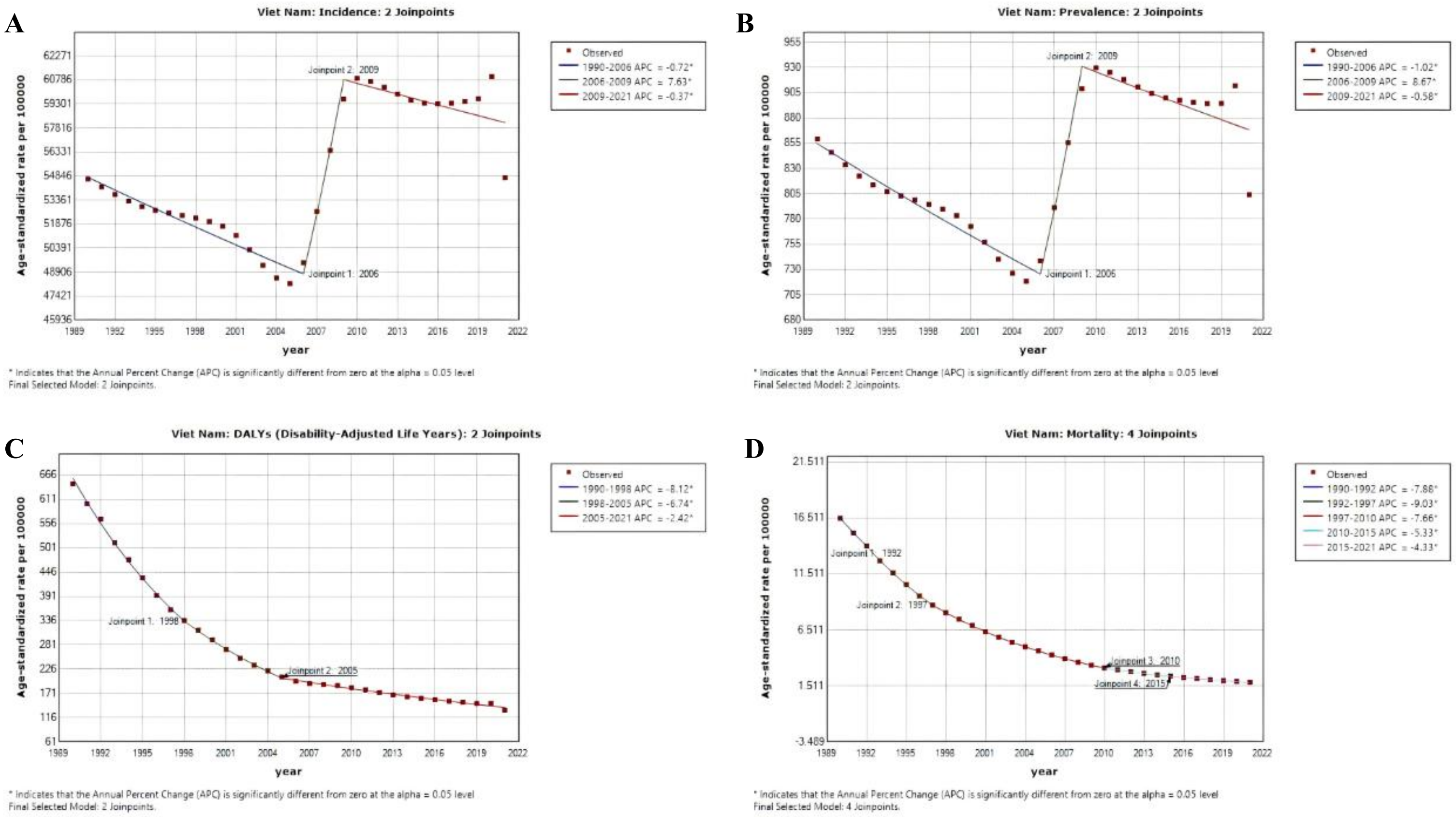

Figure S97: Temporal joinpoint analysis of diarrheal diseases in Viet Nam from 1990 to 2021.

(A) Temporal joinpoint analysis of ASIR; (B) Temporal joinpoint analysis of ASPR; (C) Temporal joinpoint analysis of ASDR; (D) Temporal joinpoint analysis of ASMR.

APC, annual percentage change; ASIR, age-standardized incidence rate; ASPR, age-standardized prevalence rate; ASDR, age-standardized DALYs rate; ASMR, age-standardized mortality rate.

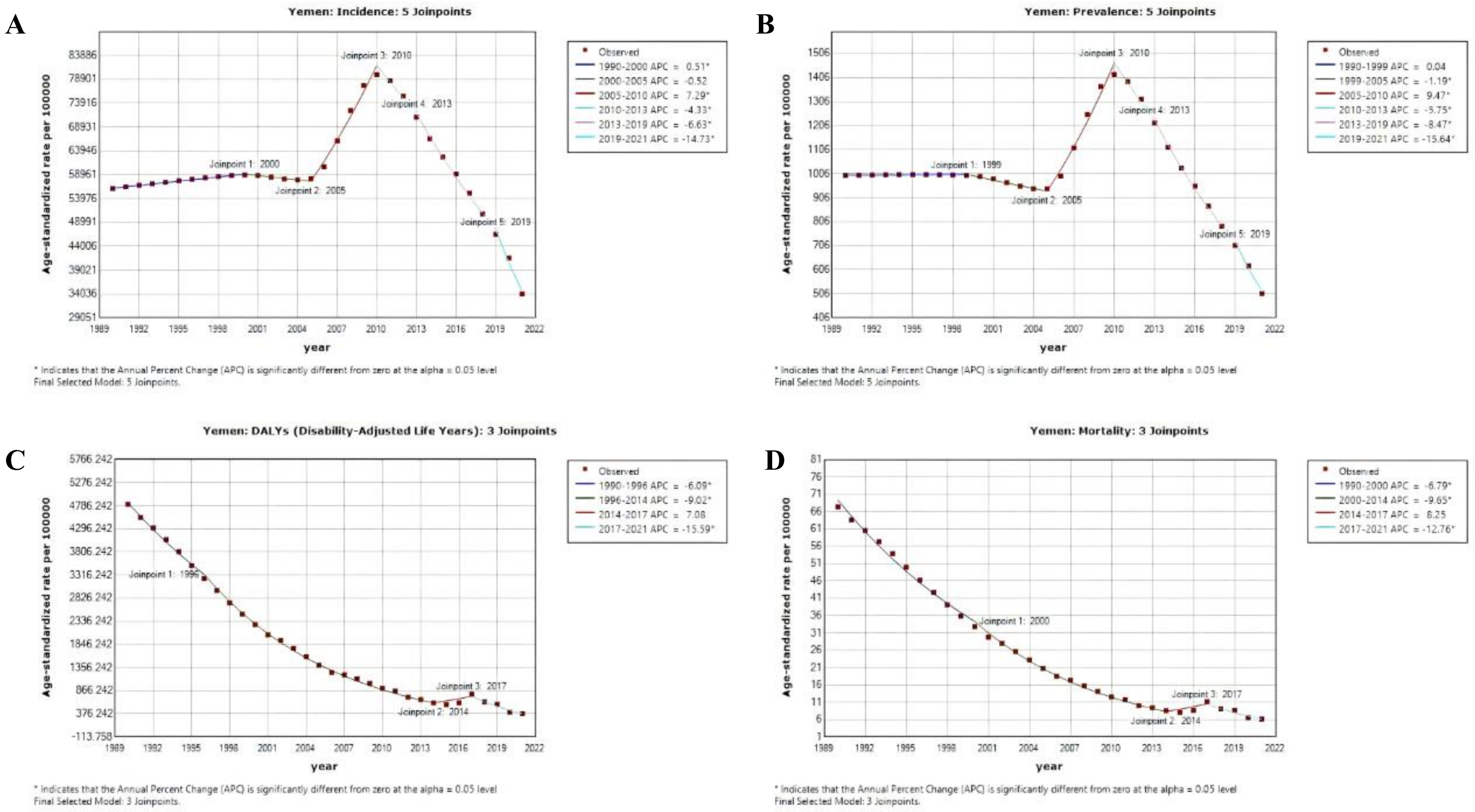

Figure S98: Temporal joinpoint analysis of diarrheal diseases in Yemen from 1990 to 2021.

(A) Temporal joinpoint analysis of ASIR; (B) Temporal joinpoint analysis of ASPR; (C) Temporal joinpoint analysis of ASDR; (D) Temporal joinpoint analysis of ASMR.

APC, annual percentage change; ASIR, age-standardized incidence rate; ASPR, age-standardized prevalence rate; ASDR, age-standardized DALYs rate; ASMR, age-standardized mortality rate.

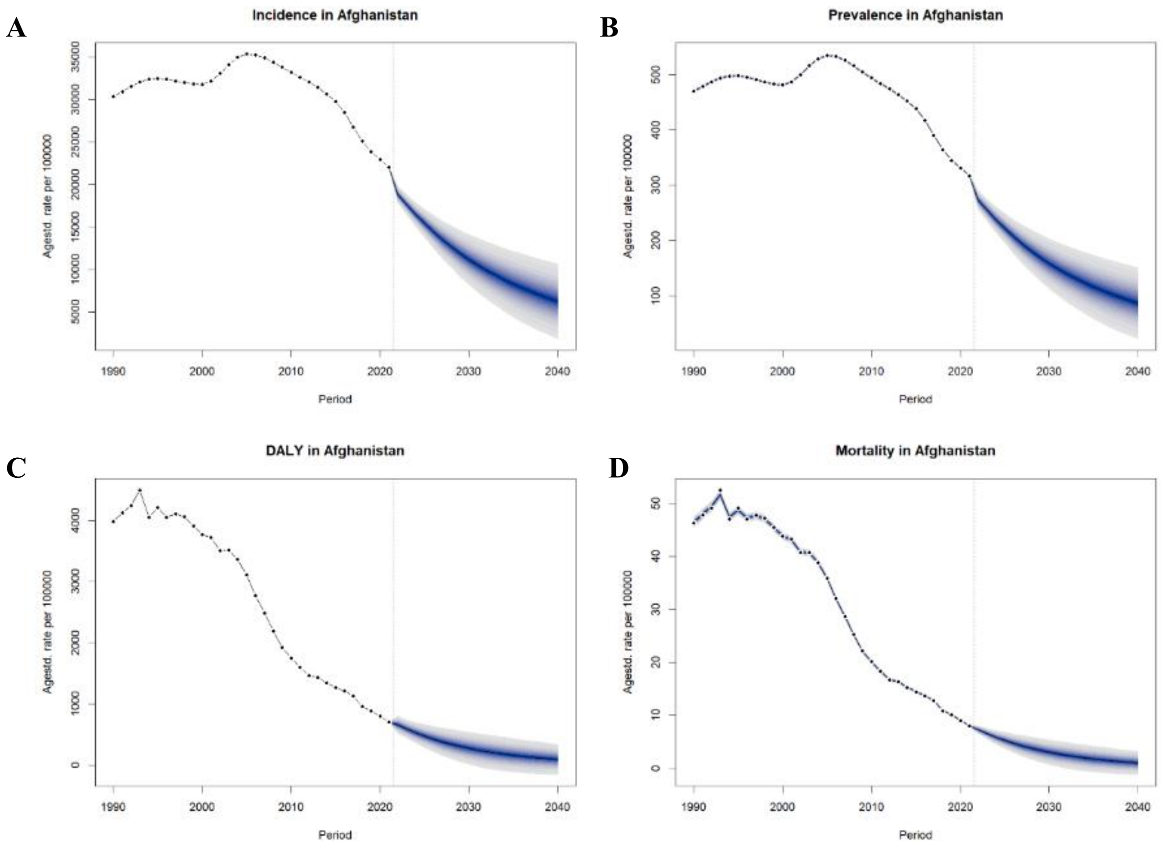

Figure S99: Prediction of diarrheal diseases burden in Afghanistan from 2022 to 2040.

(A) Prediction of ASIR from 2022 to 2040; (B) Prediction of ASPR from 2022 to 2040; (C) Prediction of ASDR from 2022 to 2040; (D) Prediction of ASMR from 2022 to 2040.

DALY, disability-adjusted life year; ASIR, age-standardized incidence rate; ASPR, age-standardized prevalence rate; ASDR, age-standardized DALYs rate; ASMR, age-standardized mortality rate.

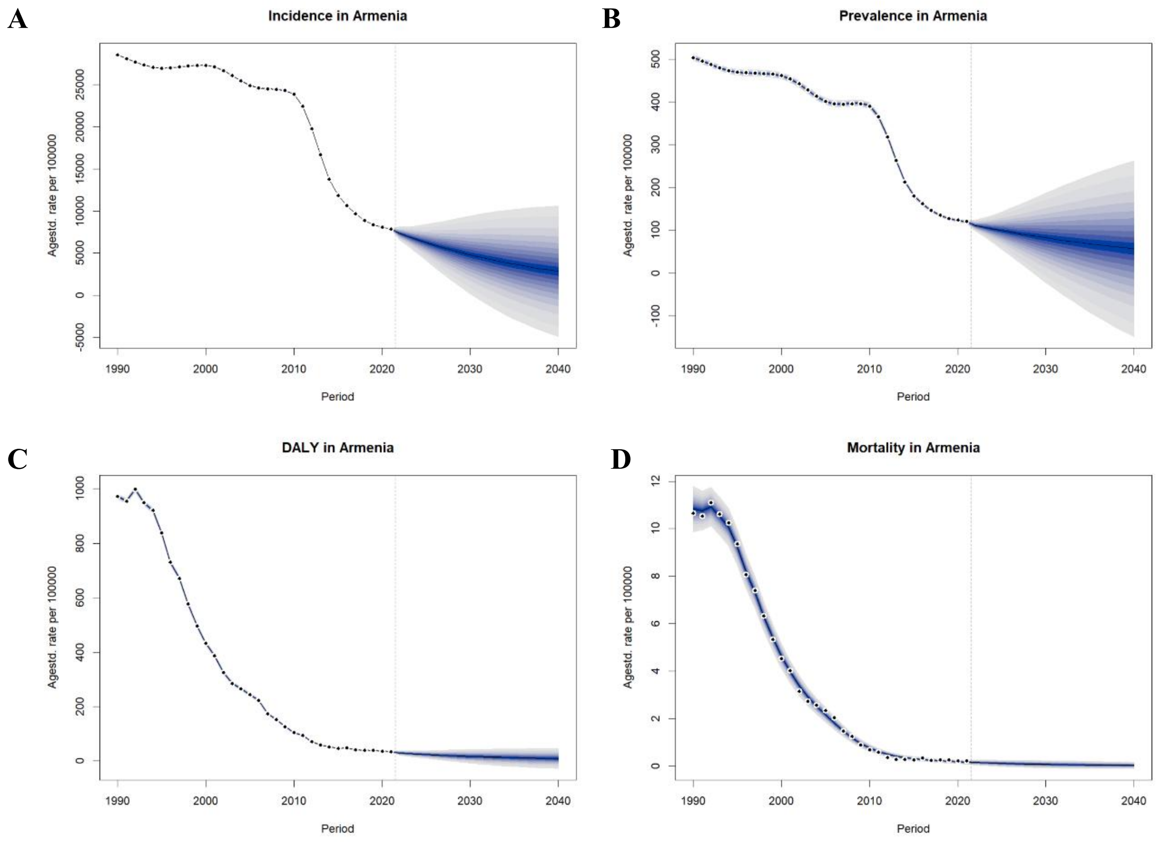

Figure S100: Prediction of diarrheal diseases burden in Armenia from 2022 to 2040.

(A) Prediction of ASIR from 2022 to 2040; (B) Prediction of ASPR from 2022 to 2040; (C) Prediction of ASDR from 2022 to 2040; (D) Prediction of ASMR from 2022 to 2040.

DALY, disability-adjusted life year; ASIR, age-standardized incidence rate; ASPR, age-standardized prevalence rate; ASDR, age-standardized DALYs rate; ASMR, age-standardized mortality rate.

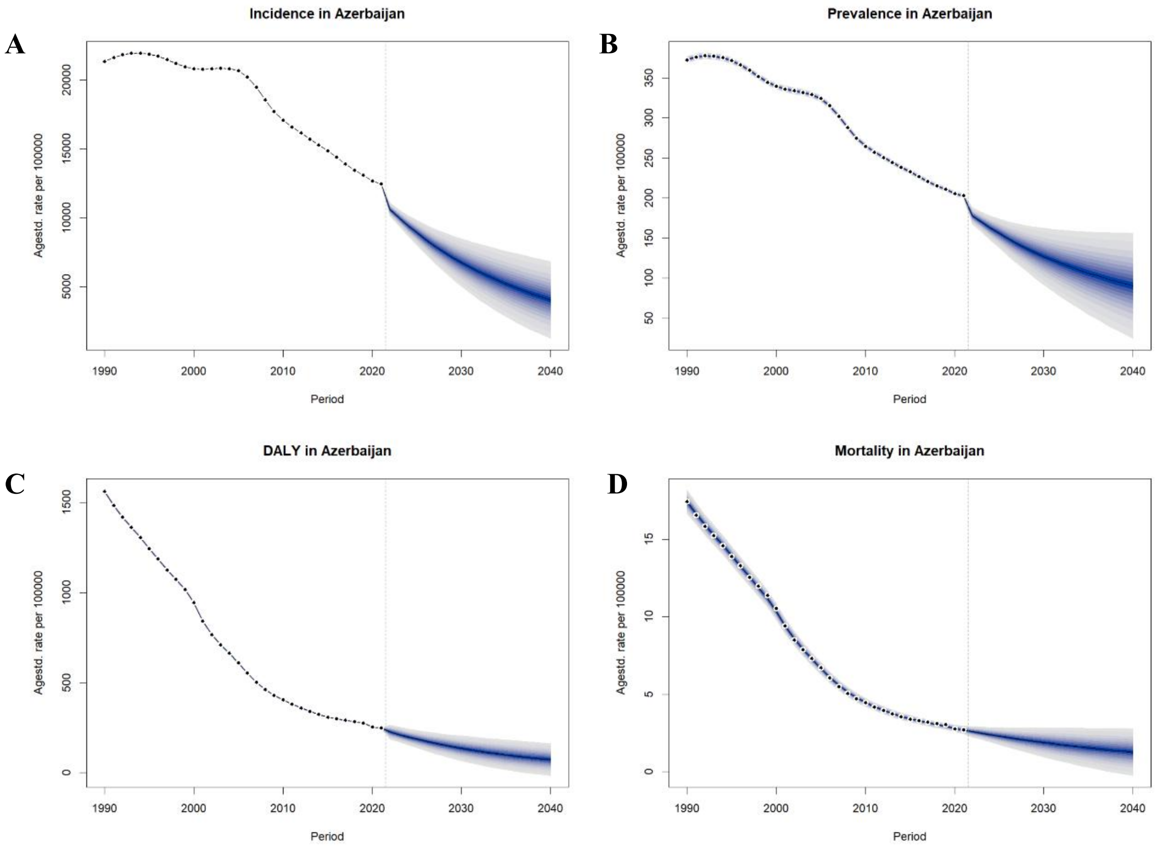

Figure S101: Prediction of diarrheal diseases burden in Azerbaijan from 2022 to 2040.

(A) Prediction of ASIR from 2022 to 2040; (B) Prediction of ASPR from 2022 to 2040; (C) Prediction of ASDR from 2022 to 2040; (D) Prediction of ASMR from 2022 to 2040.

DALY, disability-adjusted life year; ASIR, age-standardized incidence rate; ASPR, age-standardized prevalence rate; ASDR, age-standardized DALYs rate; ASMR, age-standardized mortality rate.

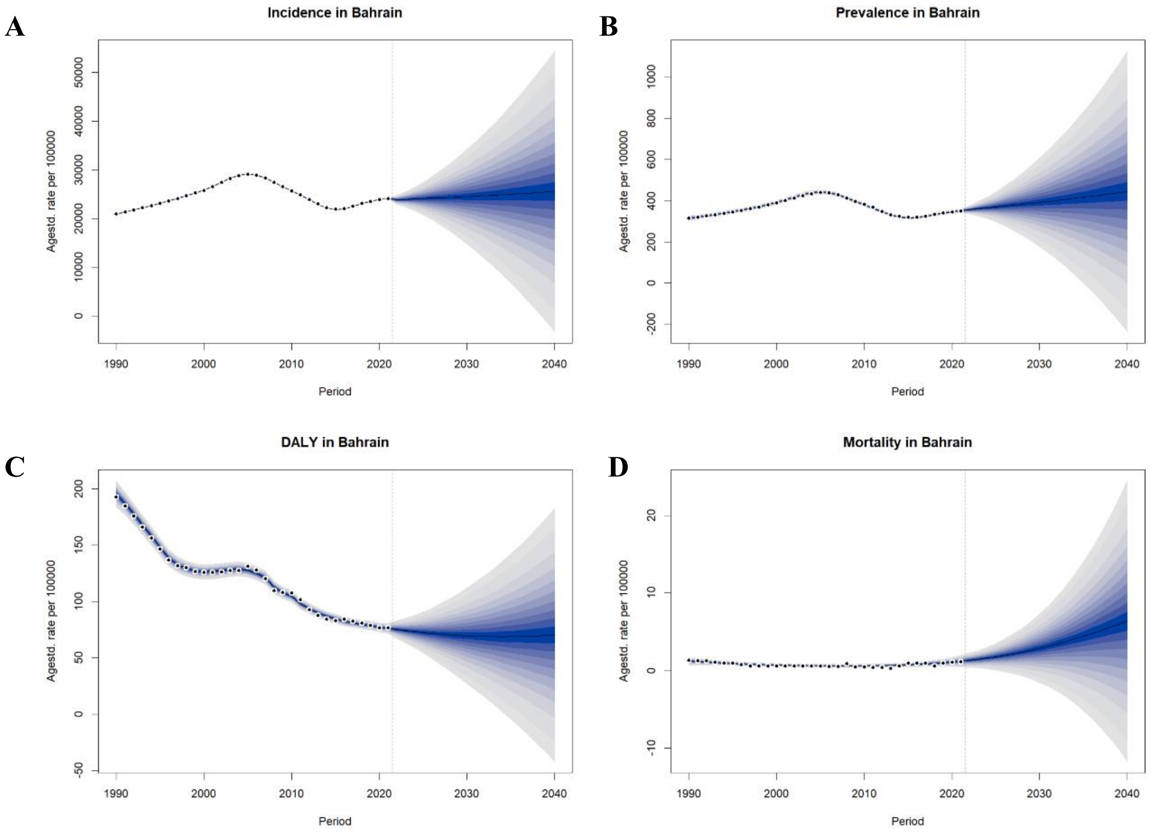

Figure S102: Prediction of diarrheal diseases burden in Bahrain from 2022 to 2040.

(A) Prediction of ASIR from 2022 to 2040; (B) Prediction of ASPR from 2022 to 2040; (C) Prediction of ASDR from 2022 to 2040; (D) Prediction of ASMR from 2022 to 2040.

DALY, disability-adjusted life year; ASIR, age-standardized incidence rate; ASPR, age-standardized prevalence rate; ASDR, age-standardized DALYs rate; ASMR, age-standardized mortality rate.

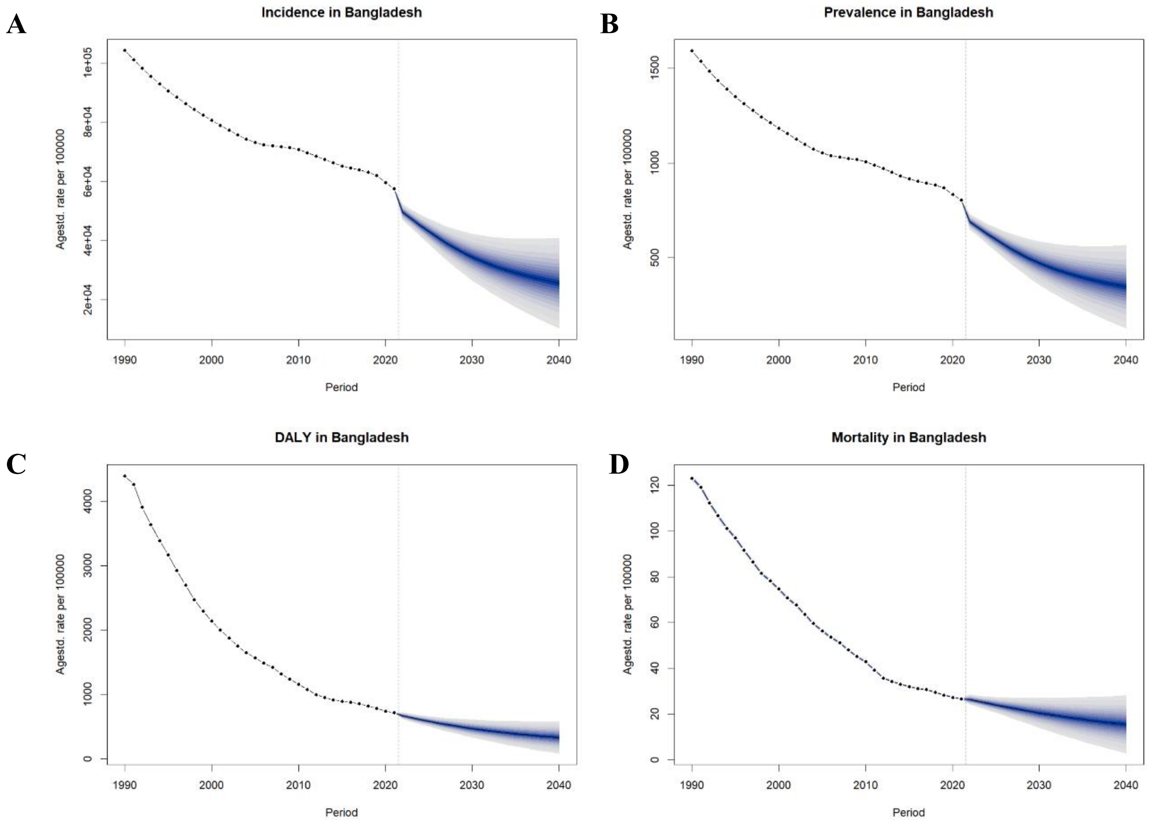

Figure S103: Prediction of diarrheal diseases burden in Bangladesh from 2022 to 2040.

(A) Prediction of ASIR from 2022 to 2040; (B) Prediction of ASPR from 2022 to 2040; (C) Prediction of ASDR from 2022 to 2040; (D) Prediction of ASMR from 2022 to 2040.

DALY, disability-adjusted life year; ASIR, age-standardized incidence rate; ASPR, age-standardized prevalence rate; ASDR, age-standardized DALYs rate; ASMR, age-standardized mortality rate.

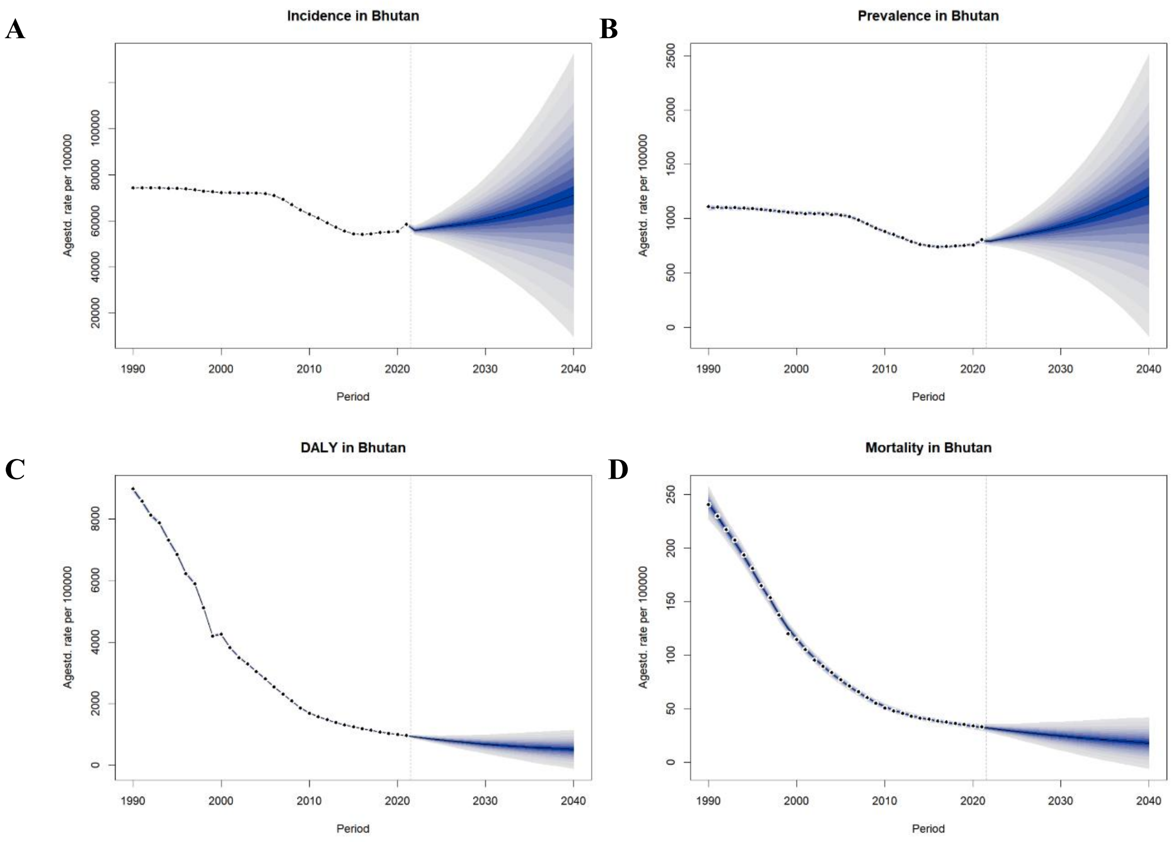

Figure S104: Prediction of diarrheal diseases burden in Bhutan from 2022 to 2040.

(A) Prediction of ASIR from 2022 to 2040; (B) Prediction of ASPR from 2022 to 2040; (C) Prediction of ASDR from 2022 to 2040; (D) Prediction of ASMR from 2022 to 2040.

DALY, disability-adjusted life year; ASIR, age-standardized incidence rate; ASPR, age-standardized prevalence rate; ASDR, age-standardized DALYs rate; ASMR, age-standardized mortality rate.

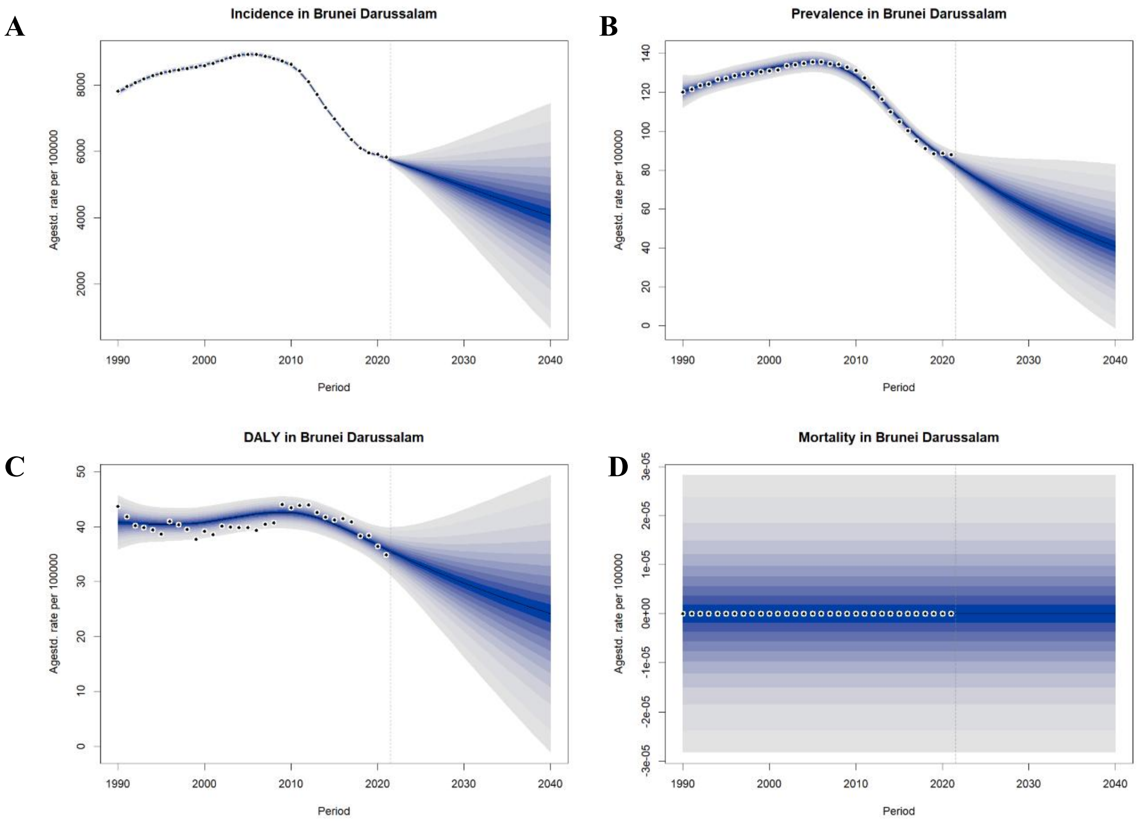

Figure S105: Prediction of diarrheal diseases burden in Brunei Darussalam from 2022 to 2040.

(A) Prediction of ASIR from 2022 to 2040; (B) Prediction of ASPR from 2022 to 2040; (C) Prediction of ASDR from 2022 to 2040; (D) Prediction of ASMR from 2022 to 2040.

DALY, disability-adjusted life year; ASIR, age-standardized incidence rate; ASPR, age-standardized prevalence rate; ASDR, age-standardized DALYs rate; ASMR, age-standardized mortality rate.

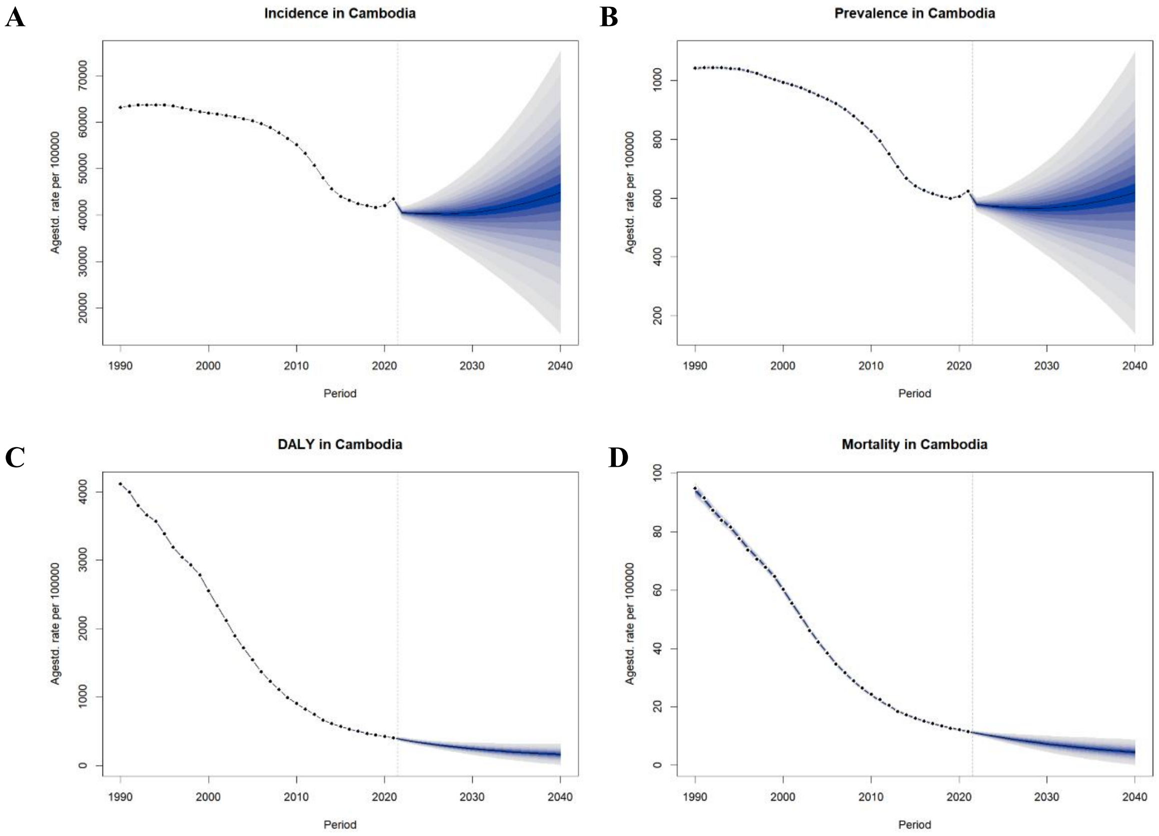

Figure S106: Prediction of diarrheal diseases burden in Cambodia from 2022 to 2040.

(A) Prediction of ASIR from 2022 to 2040; (B) Prediction of ASPR from 2022 to 2040; (C) Prediction of ASDR from 2022 to 2040; (D) Prediction of ASMR from 2022 to 2040.

DALY, disability-adjusted life year; ASIR, age-standardized incidence rate; ASPR, age-standardized prevalence rate; ASDR, age-standardized DALYs rate; ASMR, age-standardized mortality rate.

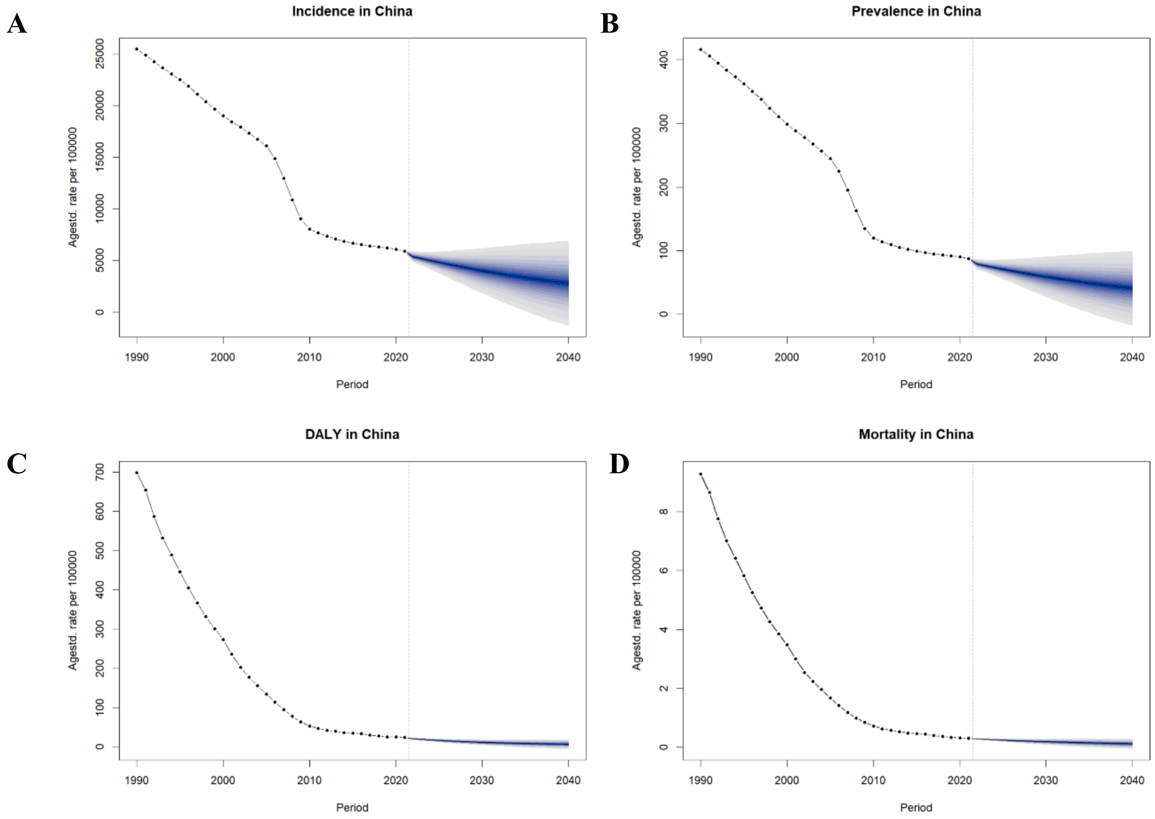

Figure S107: Prediction of diarrheal diseases burden in China from 2022 to 2040.

(A) Prediction of ASIR from 2022 to 2040; (B) Prediction of ASPR from 2022 to 2040; (C) Prediction of ASDR from 2022 to 2040; (D) Prediction of ASMR from 2022 to 2040.

DALY, disability-adjusted life year; ASIR, age-standardized incidence rate; ASPR, age-standardized prevalence rate; ASDR, age-standardized DALYs rate; ASMR, age-standardized mortality rate.

A

B

C

D

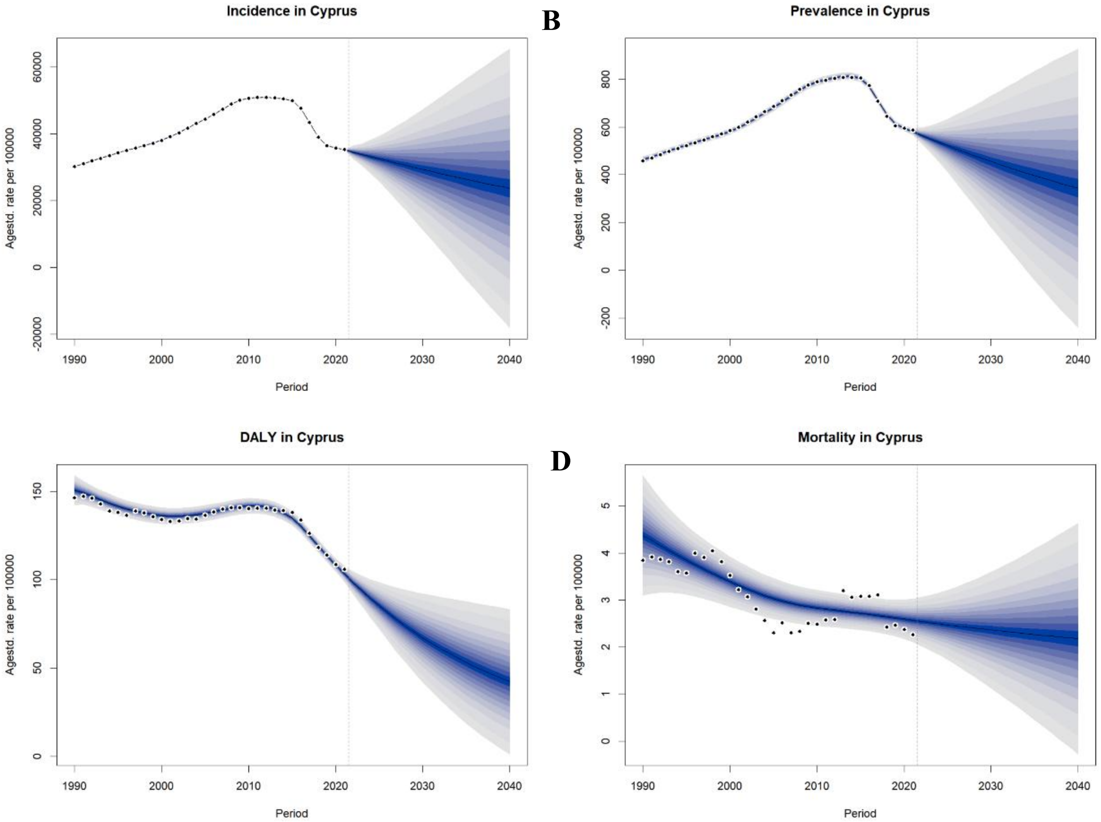

Figure S108: Prediction of diarrheal diseases burden in Cyprus from 2022 to 2040.

(A) Prediction of ASIR from 2022 to 2040; (B) Prediction of ASPR from 2022 to 2040; (C) Prediction of ASDR from 2022 to 2040; (D) Prediction of ASMR from 2022 to 2040.

DALY, disability-adjusted life year; ASIR, age-standardized incidence rate; ASPR, age-standardized prevalence rate; ASDR, age-standardized DALYs rate; ASMR, age-standardized mortality rate.

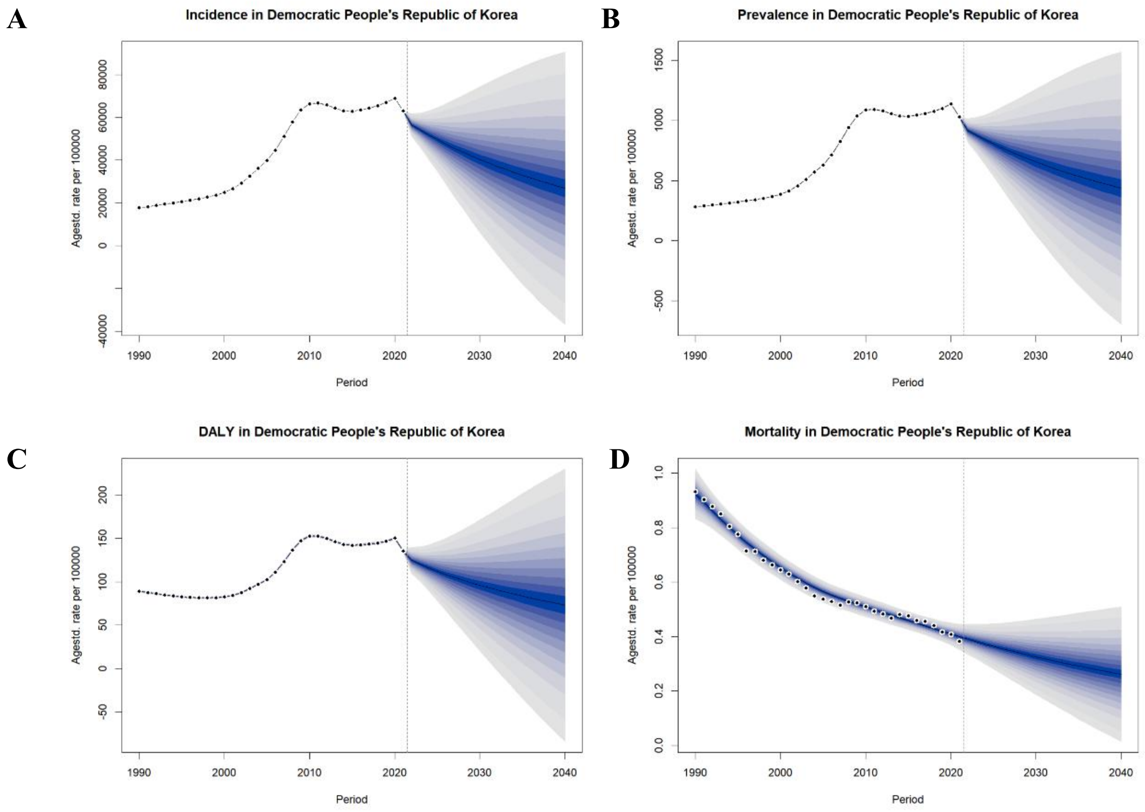

Figure S109: Prediction of diarrheal diseases burden in Democratic People's Republic of Korea from 2022 to 2040.

(A) Prediction of ASIR from 2022 to 2040; (B) Prediction of ASPR from 2022 to 2040; (C) Prediction of ASDR from 2022 to 2040; (D) Prediction of ASMR from 2022 to 2040.

DALY, disability-adjusted life year; ASIR, age-standardized incidence rate; ASPR, age-standardized prevalence rate; ASDR, age-standardized DALYs rate; ASMR, age-standardized mortality rate.

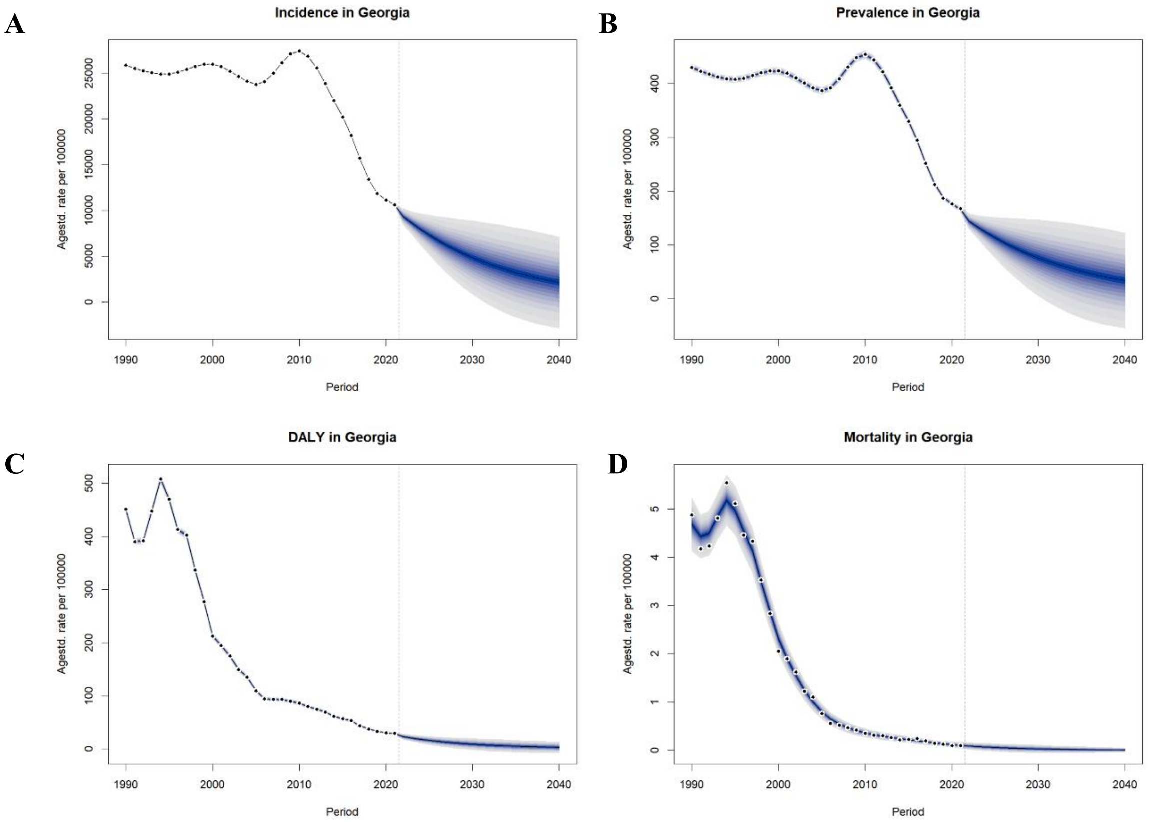

Figure S110: Prediction of diarrheal diseases burden in Georgia from 2022 to 2040.

(A) Prediction of ASIR from 2022 to 2040; (B) Prediction of ASPR from 2022 to 2040; (C) Prediction of ASDR from 2022 to 2040; (D) Prediction of ASMR from 2022 to 2040.

DALY, disability-adjusted life year; ASIR, age-standardized incidence rate; ASPR, age-standardized prevalence rate; ASDR, age-standardized DALYs rate; ASMR, age-standardized mortality rate.

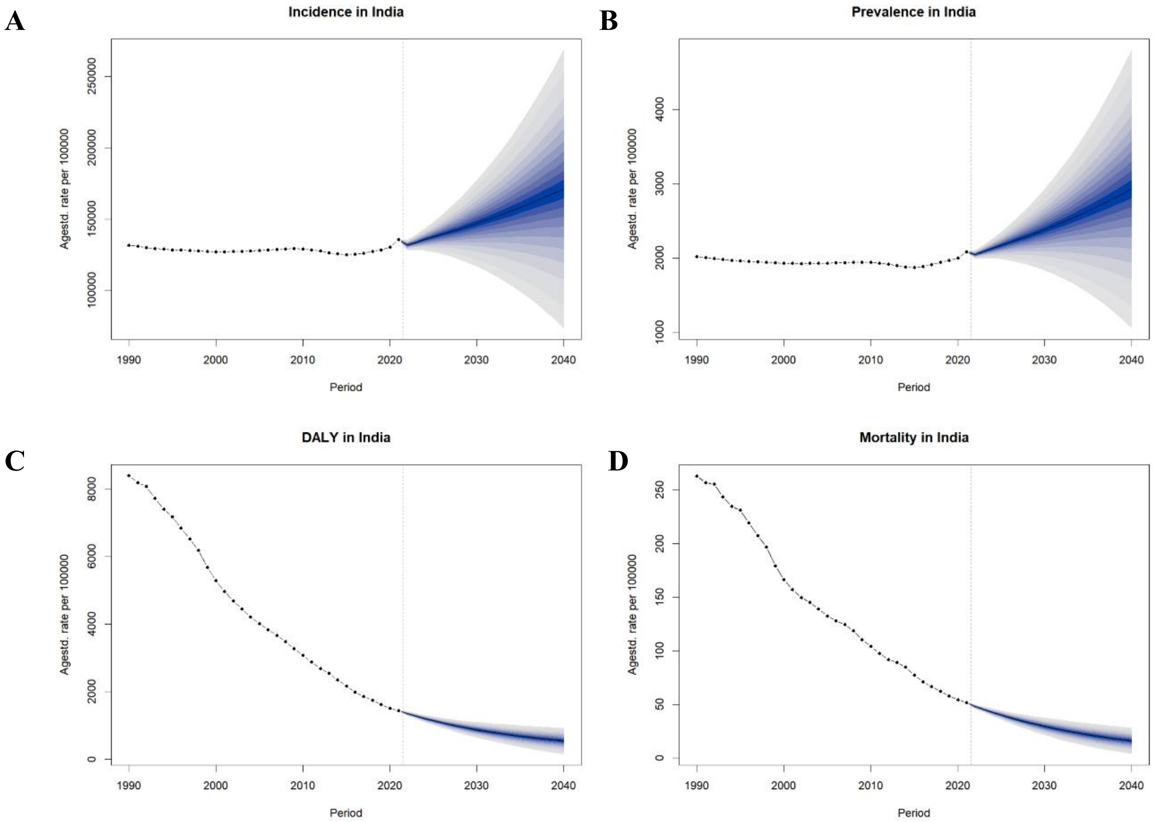

Figure S111: Prediction of diarrheal diseases burden in India from 2022 to 2040.

(A) Prediction of ASIR from 2022 to 2040; (B) Prediction of ASPR from 2022 to 2040; (C) Prediction of ASDR from 2022 to 2040; (D) Prediction of ASMR from 2022 to 2040.

DALY, disability-adjusted life year; ASIR, age-standardized incidence rate; ASPR, age-standardized prevalence rate; ASDR, age-standardized DALYs rate; ASMR, age-standardized mortality rate.

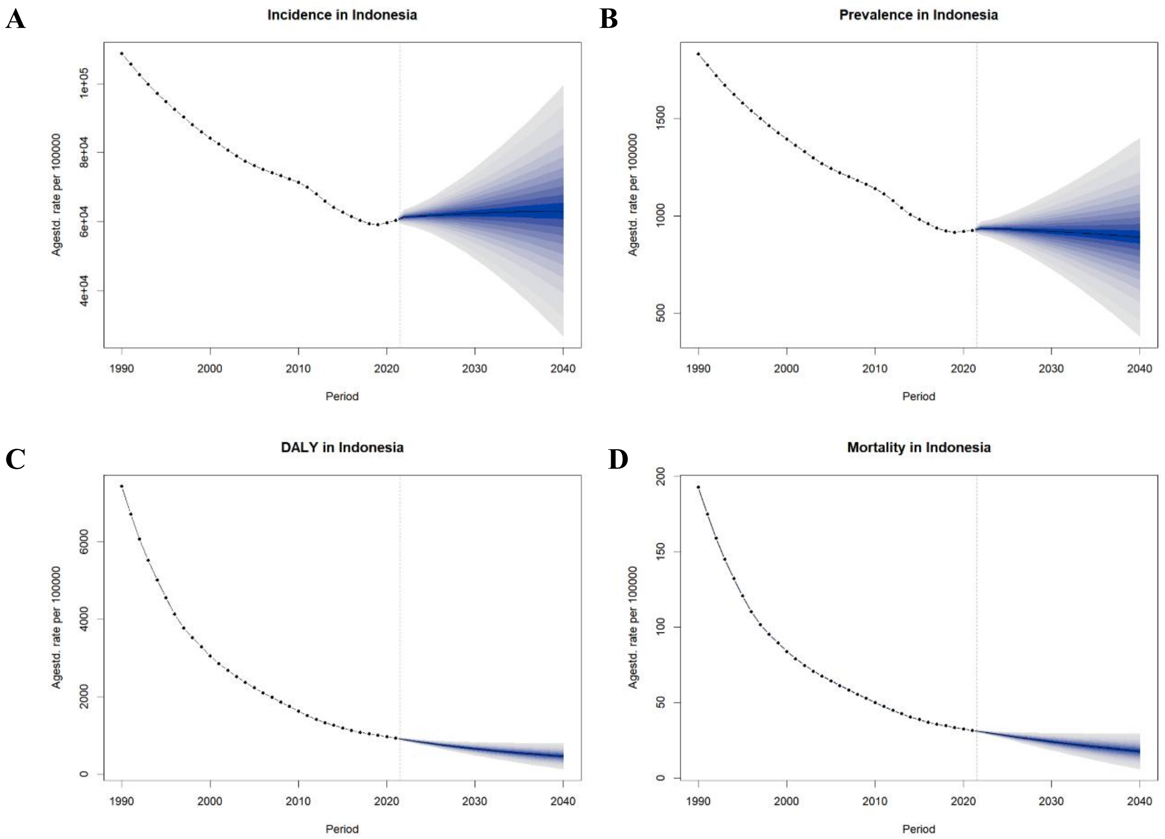

Figure S112: Prediction of diarrheal diseases burden in Indonesia from 2022 to 2040.

(A) Prediction of ASIR from 2022 to 2040; (B) Prediction of ASPR from 2022 to 2040; (C) Prediction of ASDR from 2022 to 2040; (D) Prediction of ASMR from 2022 to 2040.

DALY, disability-adjusted life year; ASIR, age-standardized incidence rate; ASPR, age-standardized prevalence rate; ASDR, age-standardized DALYs rate; ASMR, age-standardized mortality rate.

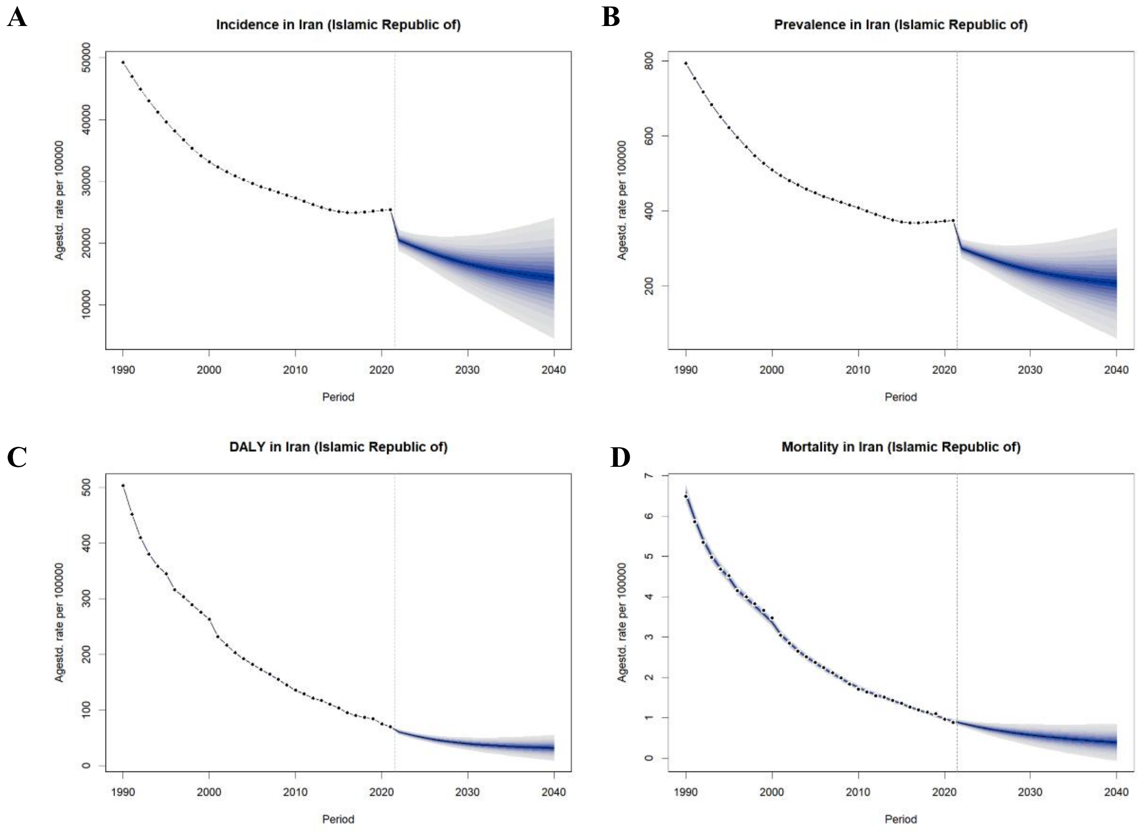

Figure S113: Prediction of diarrheal diseases burden in Iran (Islamic Republic of) from 2022 to 2040.

(A) Prediction of ASIR from 2022 to 2040; (B) Prediction of ASPR from 2022 to 2040; (C) Prediction of ASDR from 2022 to 2040; (D) Prediction of ASMR from 2022 to 2040.

DALY, disability-adjusted life year; ASIR, age-standardized incidence rate; ASPR, age-standardized prevalence rate; ASDR, age-standardized DALYs rate; ASMR, age-standardized mortality rate.

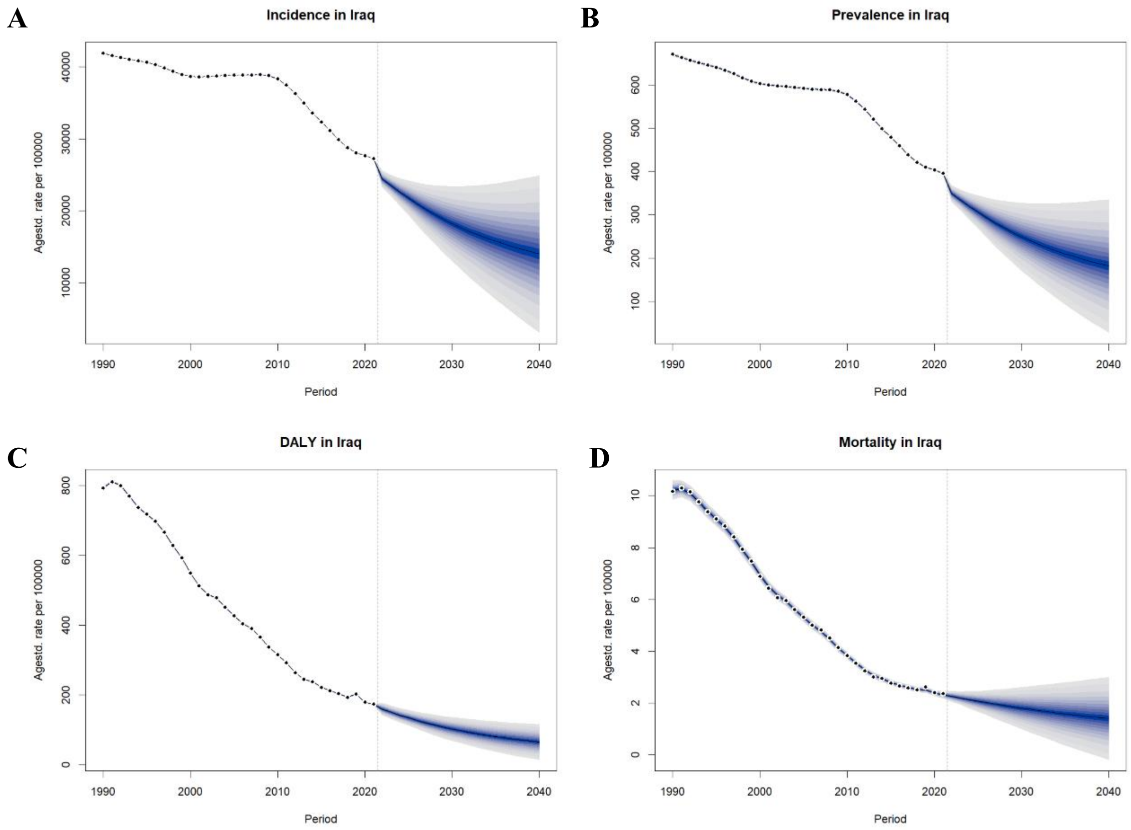

Figure S114: Prediction of diarrheal diseases burden in Iraq from 2022 to 2040.

(A) Prediction of ASIR from 2022 to 2040; (B) Prediction of ASPR from 2022 to 2040; (C) Prediction of ASDR from 2022 to 2040; (D) Prediction of ASMR from 2022 to 2040.

DALY, disability-adjusted life year; ASIR, age-standardized incidence rate; ASPR, age-standardized prevalence rate; ASDR, age-standardized DALYs rate; ASMR, age-standardized mortality rate.

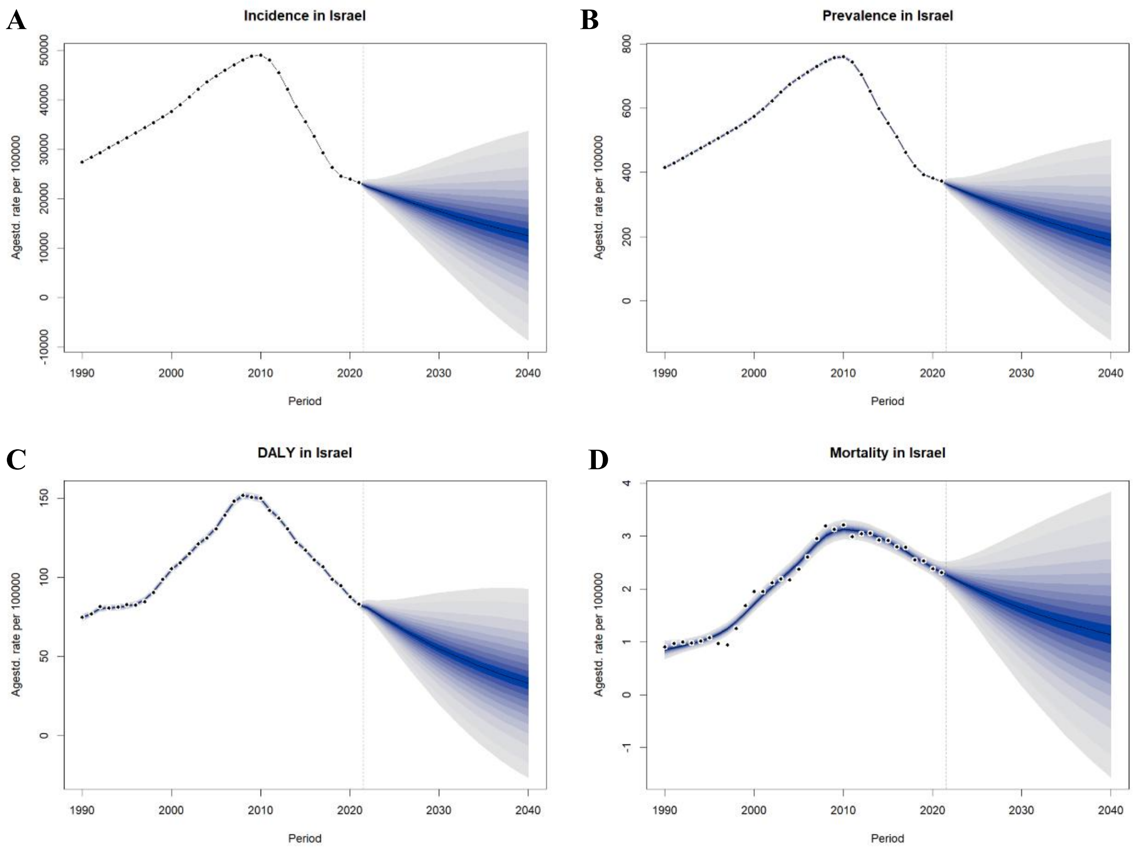

Figure S115: Prediction of diarrheal diseases burden in Israel from 2022 to 2040.

(A) Prediction of ASIR from 2022 to 2040; (B) Prediction of ASPR from 2022 to 2040; (C) Prediction of ASDR from 2022 to 2040; (D) Prediction of ASMR from 2022 to 2040.

DALY, disability-adjusted life year; ASIR, age-standardized incidence rate; ASPR, age-standardized prevalence rate; ASDR, age-standardized DALYs rate; ASMR, age-standardized mortality rate.

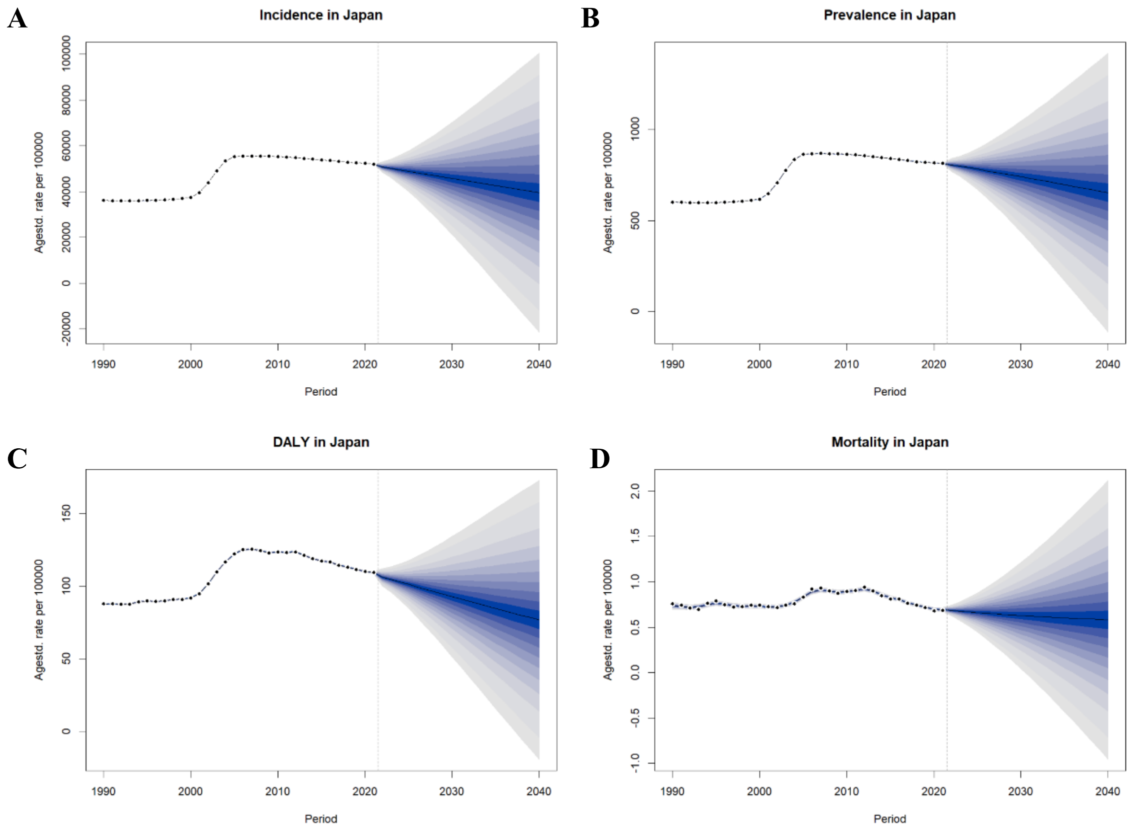

Figure S116: Prediction of diarrheal diseases burden in Japan from 2022 to 2040.

(A) Prediction of ASIR from 2022 to 2040; (B) Prediction of ASPR from 2022 to 2040; (C) Prediction of ASDR from 2022 to 2040; (D) Prediction of ASMR from 2022 to 2040.

DALY, disability-adjusted life year; ASIR, age-standardized incidence rate; ASPR, age-standardized prevalence rate; ASDR, age-standardized DALYs rate; ASMR, age-standardized mortality rate.

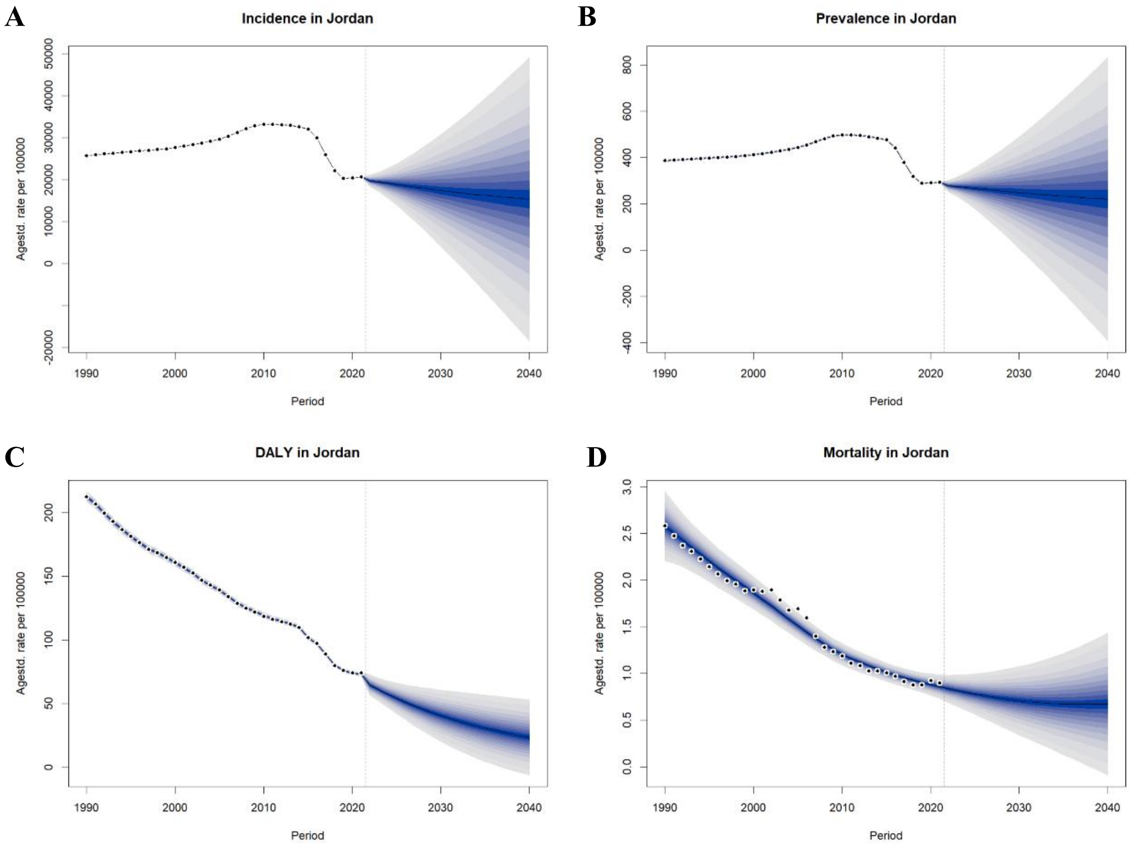

Figure S117: Prediction of diarrheal diseases burden in Jordan from 2022 to 2040.

(A) Prediction of ASIR from 2022 to 2040; (B) Prediction of ASPR from 2022 to 2040; (C) Prediction of ASDR from 2022 to 2040; (D) Prediction of ASMR from 2022 to 2040.

DALY, disability-adjusted life year; ASIR, age-standardized incidence rate; ASPR, age-standardized prevalence rate; ASDR, age-standardized DALYs rate; ASMR, age-standardized mortality rate.

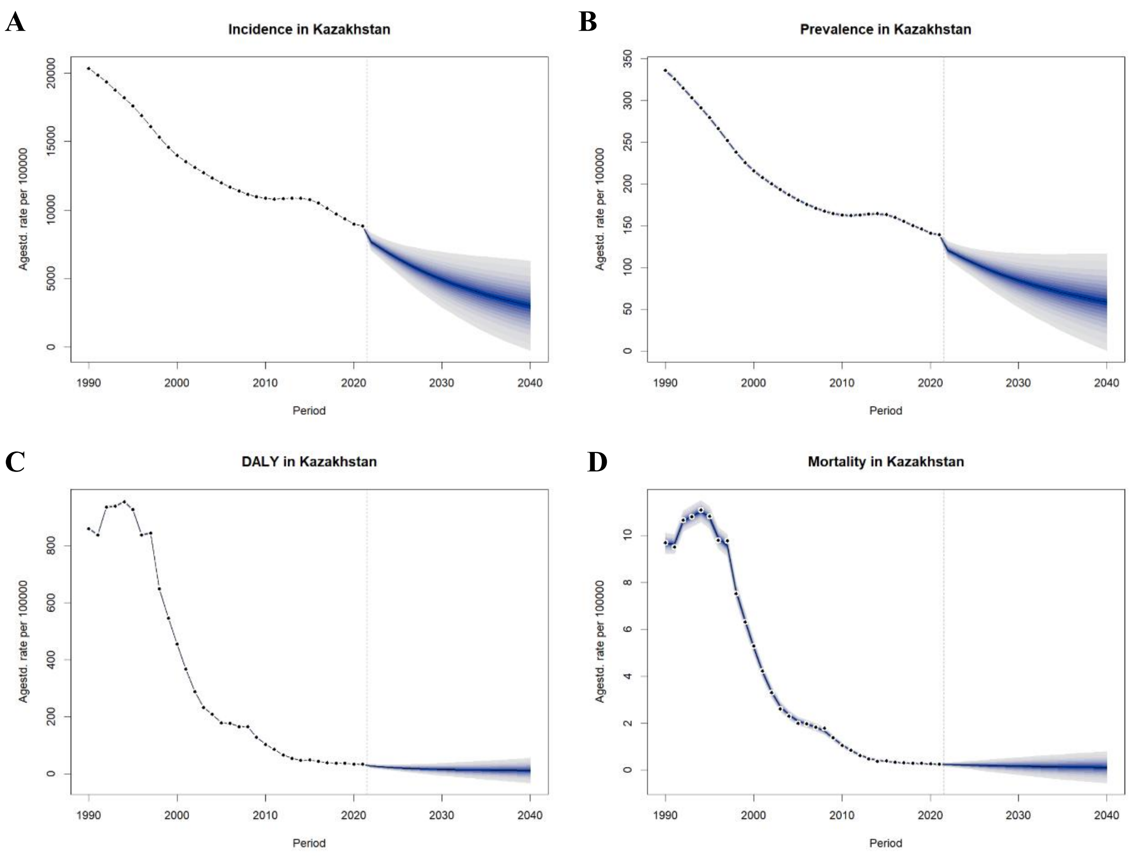

Figure S118: Prediction of diarrheal diseases burden in Kazakhstan from 2022 to 2040.

(A) Prediction of ASIR from 2022 to 2040; (B) Prediction of ASPR from 2022 to 2040; (C) Prediction of ASDR from 2022 to 2040; (D) Prediction of ASMR from 2022 to 2040.

DALY, disability-adjusted life year; ASIR, age-standardized incidence rate; ASPR, age-standardized prevalence rate; ASDR, age-standardized DALYs rate; ASMR, age-standardized mortality rate.

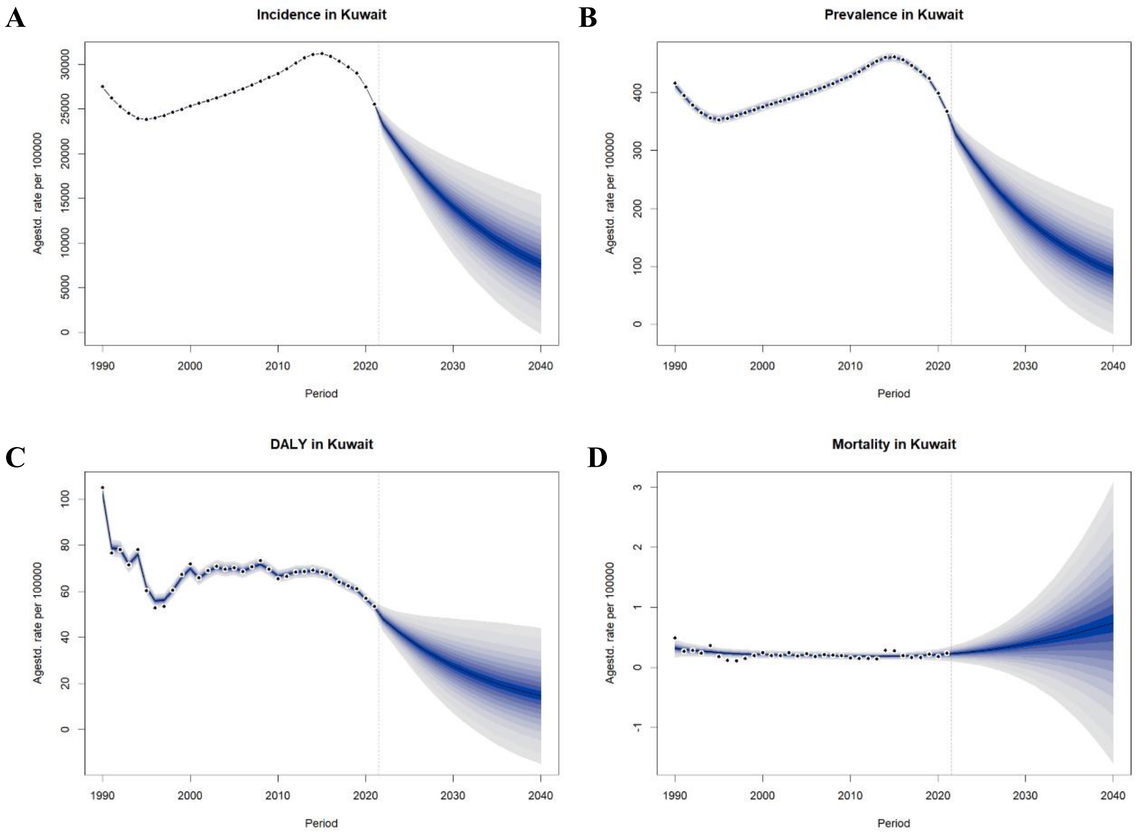

Figure S119: Prediction of diarrheal diseases burden in Kuwait from 2022 to 2040.

(A) Prediction of ASIR from 2022 to 2040; (B) Prediction of ASPR from 2022 to 2040; (C) Prediction of ASDR from 2022 to 2040; (D) Prediction of ASMR from 2022 to 2040.

DALY, disability-adjusted life year; ASIR, age-standardized incidence rate; ASPR, age-standardized prevalence rate; ASDR, age-standardized DALYs rate; ASMR, age-standardized mortality rate.

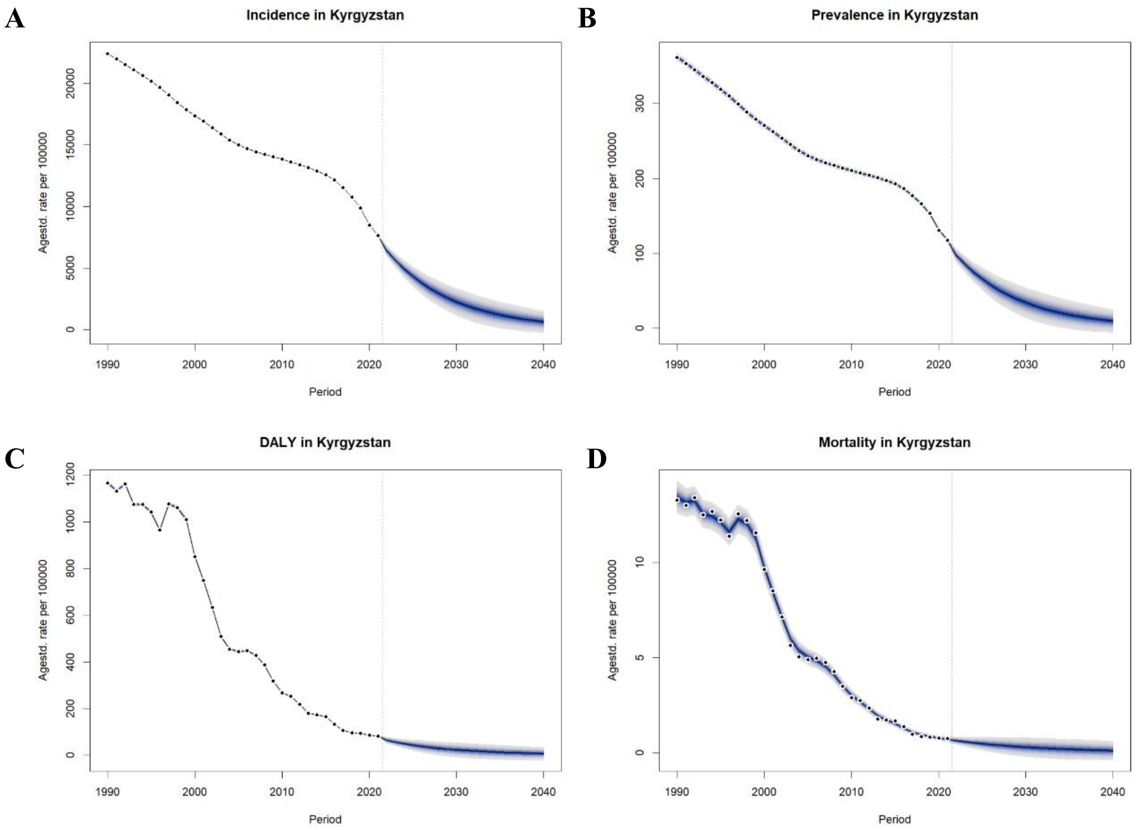

Figure S120: Prediction of diarrheal diseases burden in Kyrgyzstan from 2022 to 2040.

(A) Prediction of ASIR from 2022 to 2040; (B) Prediction of ASPR from 2022 to 2040; (C) Prediction of ASDR from 2022 to 2040; (D) Prediction of ASMR from 2022 to 2040.

DALY, disability-adjusted life year; ASIR, age-standardized incidence rate; ASPR, age-standardized prevalence rate; ASDR, age-standardized DALYs rate; ASMR, age-standardized mortality rate.

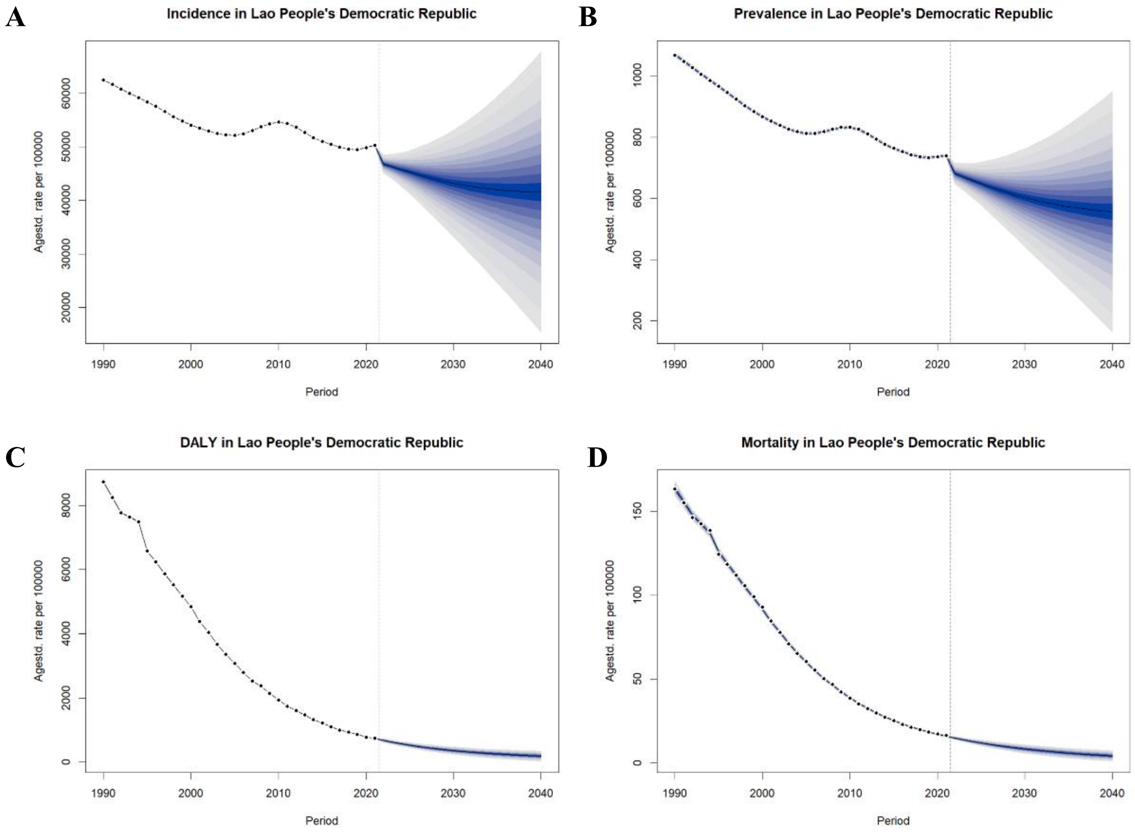

Figure S121: Prediction of diarrheal diseases burden in Lao People's Democratic Republic from 2022 to 2040.

(A) Prediction of ASIR from 2022 to 2040; (B) Prediction of ASPR from 2022 to 2040; (C) Prediction of ASDR from 2022 to 2040; (D) Prediction of ASMR from 2022 to 2040.

DALY, disability-adjusted life year; ASIR, age-standardized incidence rate; ASPR, age-standardized prevalence rate; ASDR, age-standardized DALYs rate; ASMR, age-standardized mortality rate.

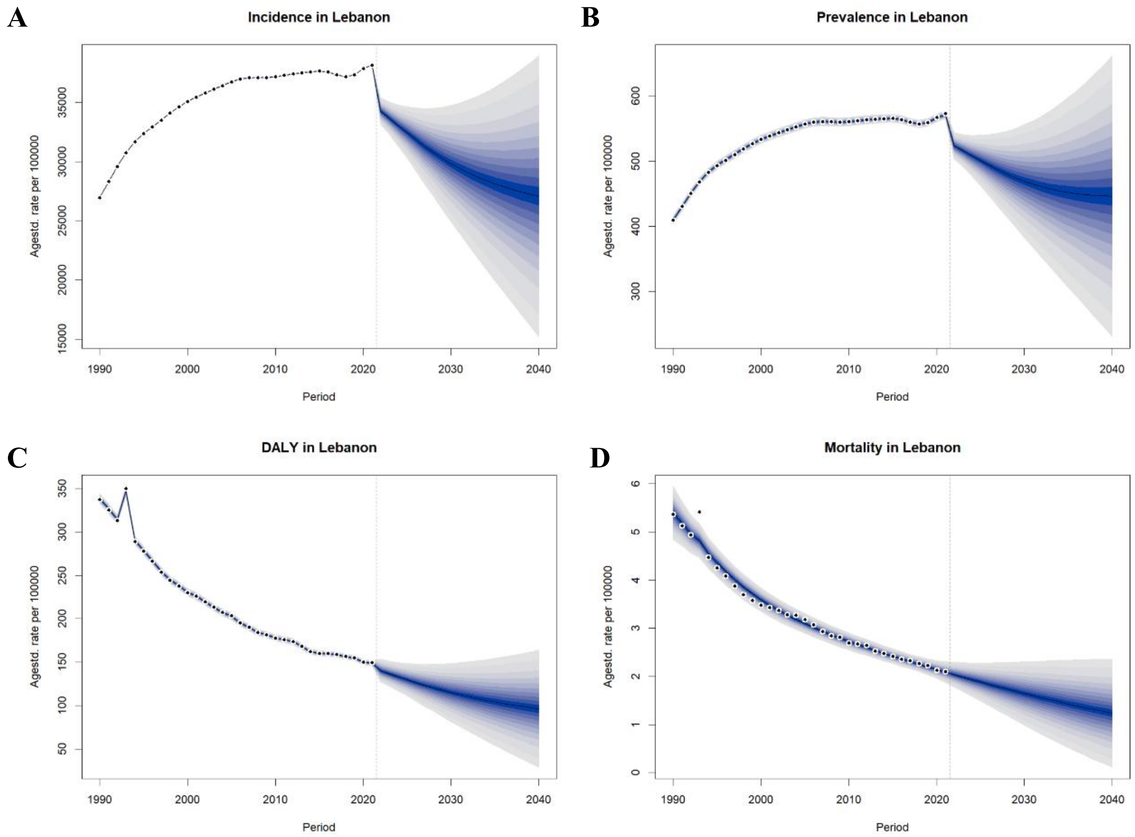

Figure S122: Prediction of diarrheal diseases burden in Lebanon from 2022 to 2040.

(A) Prediction of ASIR from 2022 to 2040; (B) Prediction of ASPR from 2022 to 2040; (C) Prediction of ASDR from 2022 to 2040; (D) Prediction of ASMR from 2022 to 2040.

DALY, disability-adjusted life year; ASIR, age-standardized incidence rate; ASPR, age-standardized prevalence rate; ASDR, age-standardized DALYs rate; ASMR, age-standardized mortality rate.

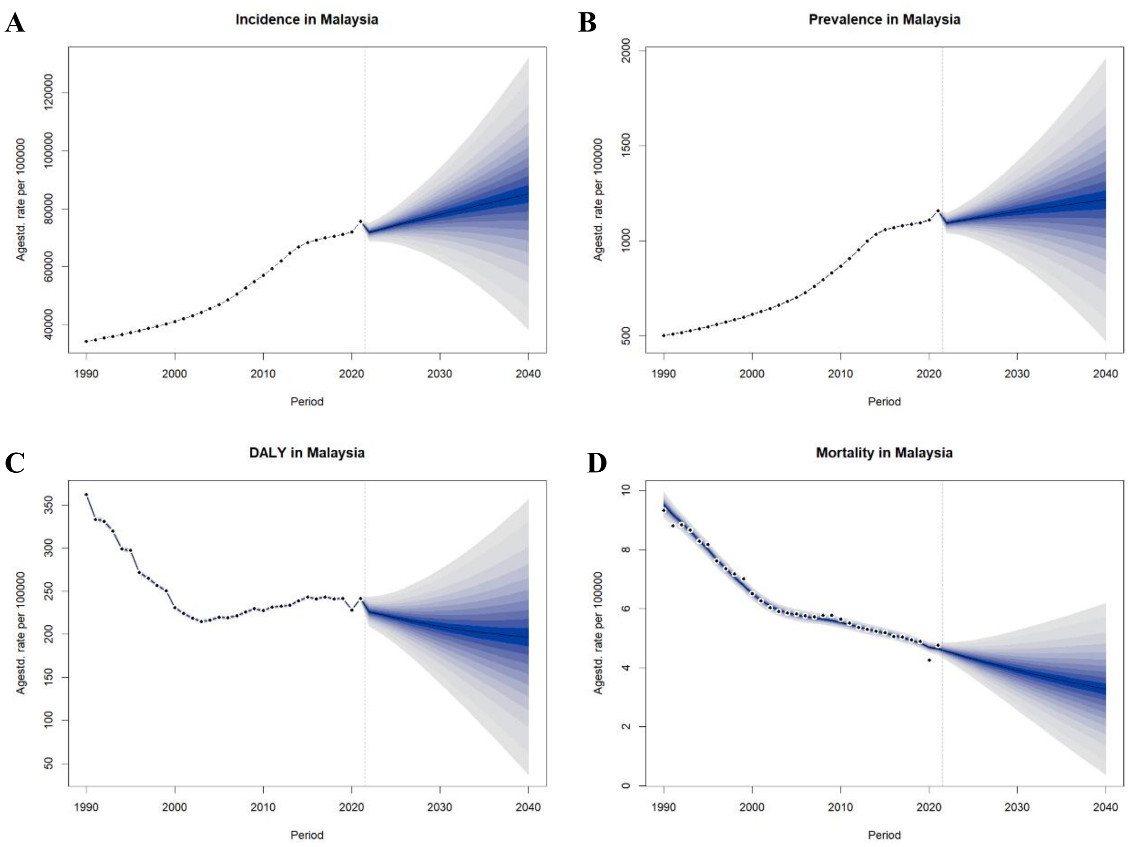

Figure S123: Prediction of diarrheal diseases burden in Malaysia from 2022 to 2040.

(A) Prediction of ASIR from 2022 to 2040; (B) Prediction of ASPR from 2022 to 2040; (C) Prediction of ASDR from 2022 to 2040; (D) Prediction of ASMR from 2022 to 2040.

DALY, disability-adjusted life year; ASIR, age-standardized incidence rate; ASPR, age-standardized prevalence rate; ASDR, age-standardized DALYs rate; ASMR, age-standardized mortality rate.

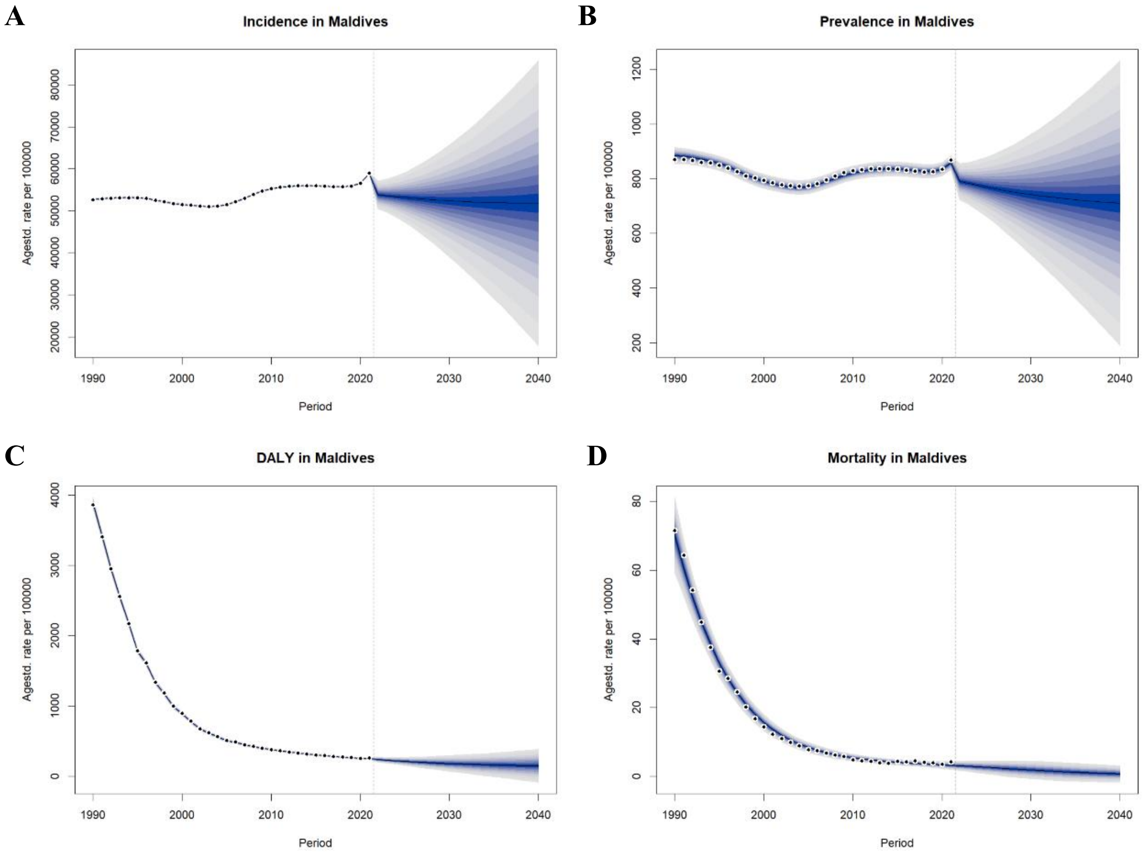

Figure S124: Prediction of diarrheal diseases burden in Maldives from 2022 to 2040.

(A) Prediction of ASIR from 2022 to 2040; (B) Prediction of ASPR from 2022 to 2040; (C) Prediction of ASDR from 2022 to 2040; (D) Prediction of ASMR from 2022 to 2040.

DALY, disability-adjusted life year; ASIR, age-standardized incidence rate; ASPR, age-standardized prevalence rate; ASDR, age-standardized DALYs rate; ASMR, age-standardized mortality rate.

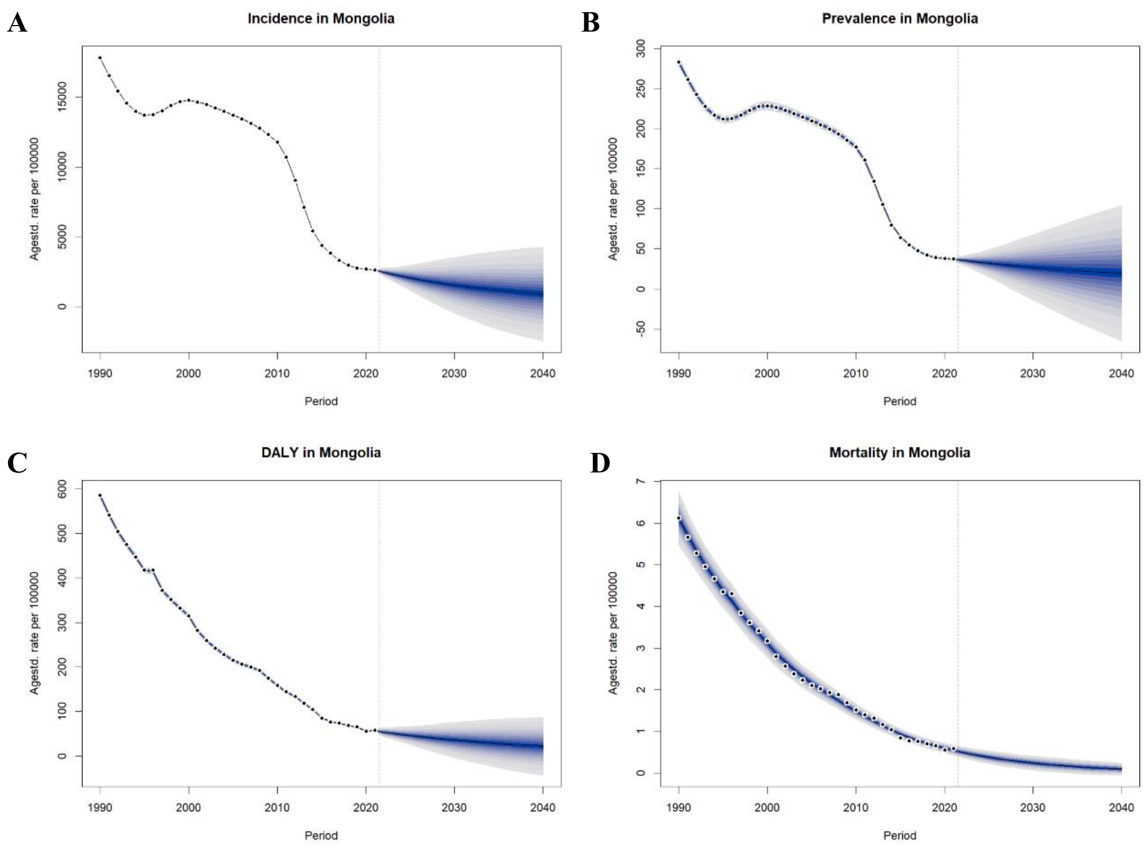

Figure S125: Prediction of diarrheal diseases burden in Mongolia from 2022 to 2040.

(A) Prediction of ASIR from 2022 to 2040; (B) Prediction of ASPR from 2022 to 2040; (C) Prediction of ASDR from 2022 to 2040; (D) Prediction of ASMR from 2022 to 2040.

DALY, disability-adjusted life year; ASIR, age-standardized incidence rate; ASPR, age-standardized prevalence rate; ASDR, age-standardized DALYs rate; ASMR, age-standardized mortality rate.

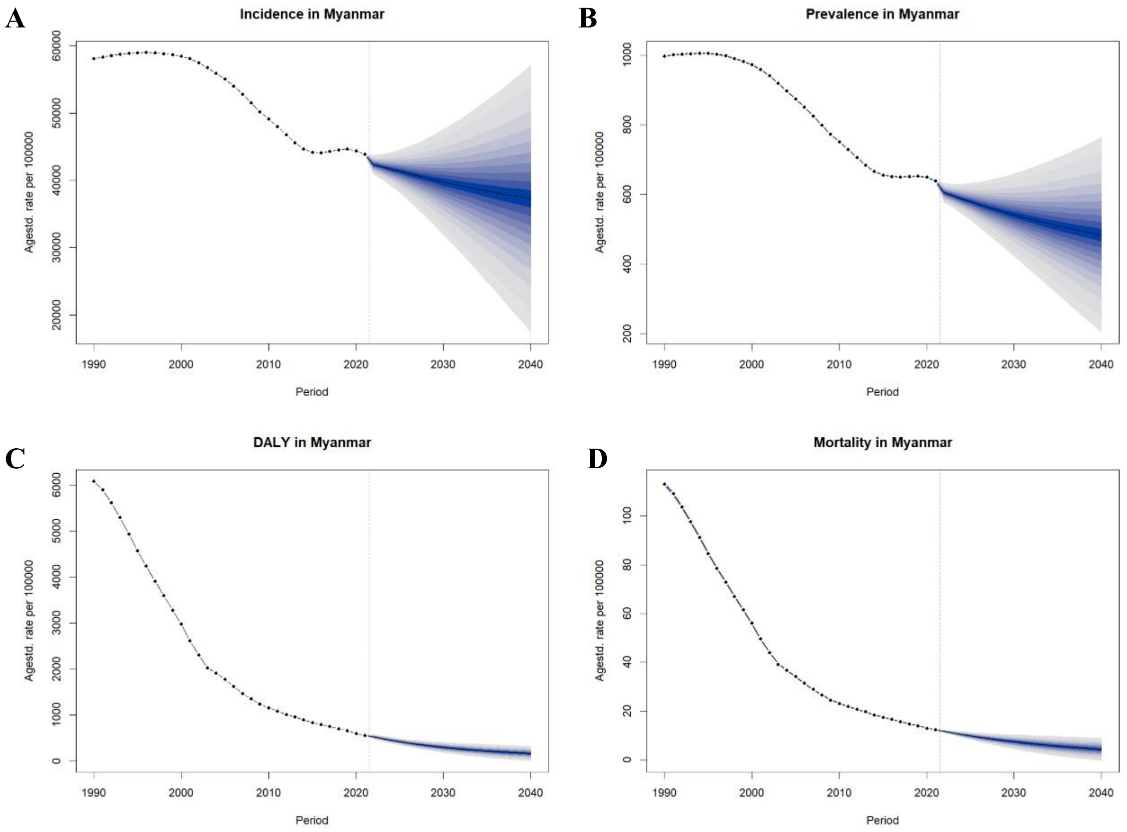

Figure S126: Prediction of diarrheal diseases burden in Myanmar from 2022 to 2040.

(A) Prediction of ASIR from 2022 to 2040; (B) Prediction of ASPR from 2022 to 2040; (C) Prediction of ASDR from 2022 to 2040; (D) Prediction of ASMR from 2022 to 2040.

DALY, disability-adjusted life year; ASIR, age-standardized incidence rate; ASPR, age-standardized prevalence rate; ASDR, age-standardized DALYs rate; ASMR, age-standardized mortality rate.

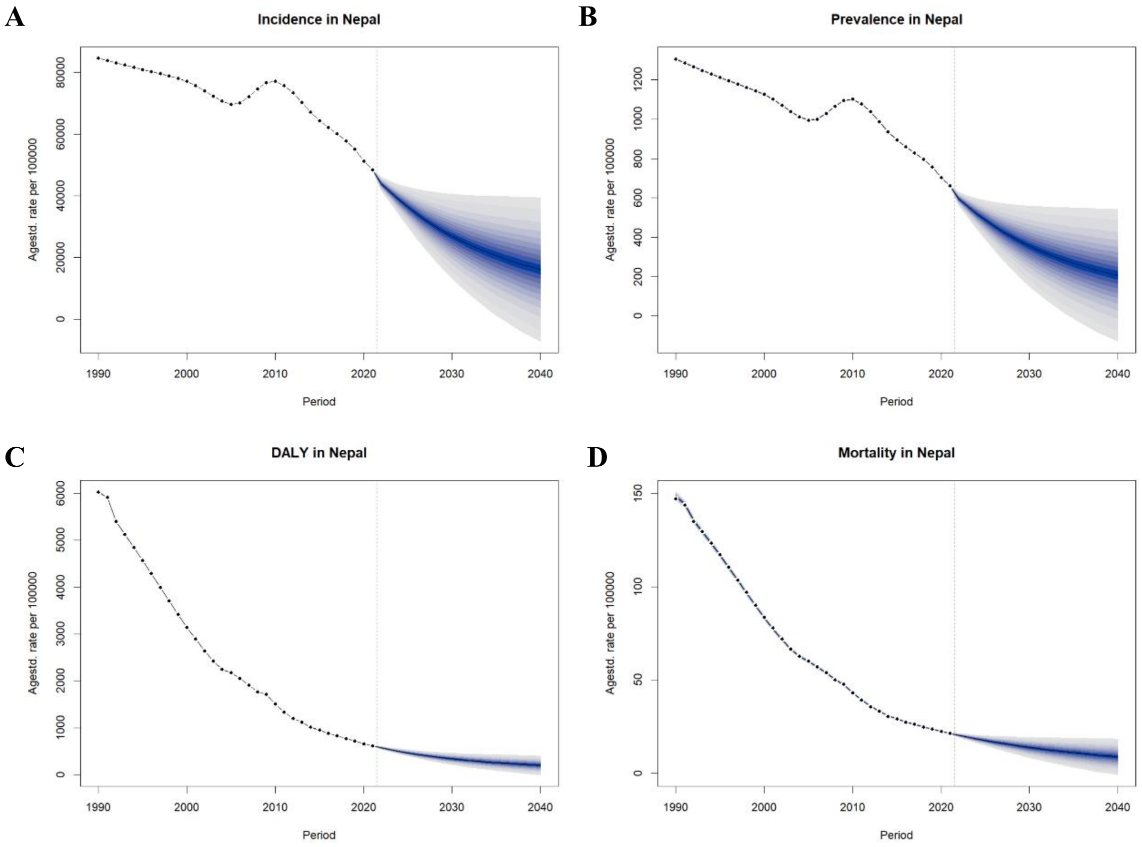

Figure S127: Prediction of diarrheal diseases burden in Nepal from 2022 to 2040.

(A) Prediction of ASIR from 2022 to 2040; (B) Prediction of ASPR from 2022 to 2040; (C) Prediction of ASDR from 2022 to 2040; (D) Prediction of ASMR from 2022 to 2040.

DALY, disability-adjusted life year; ASIR, age-standardized incidence rate; ASPR, age-standardized prevalence rate; ASDR, age-standardized DALYs rate; ASMR, age-standardized mortality rate.

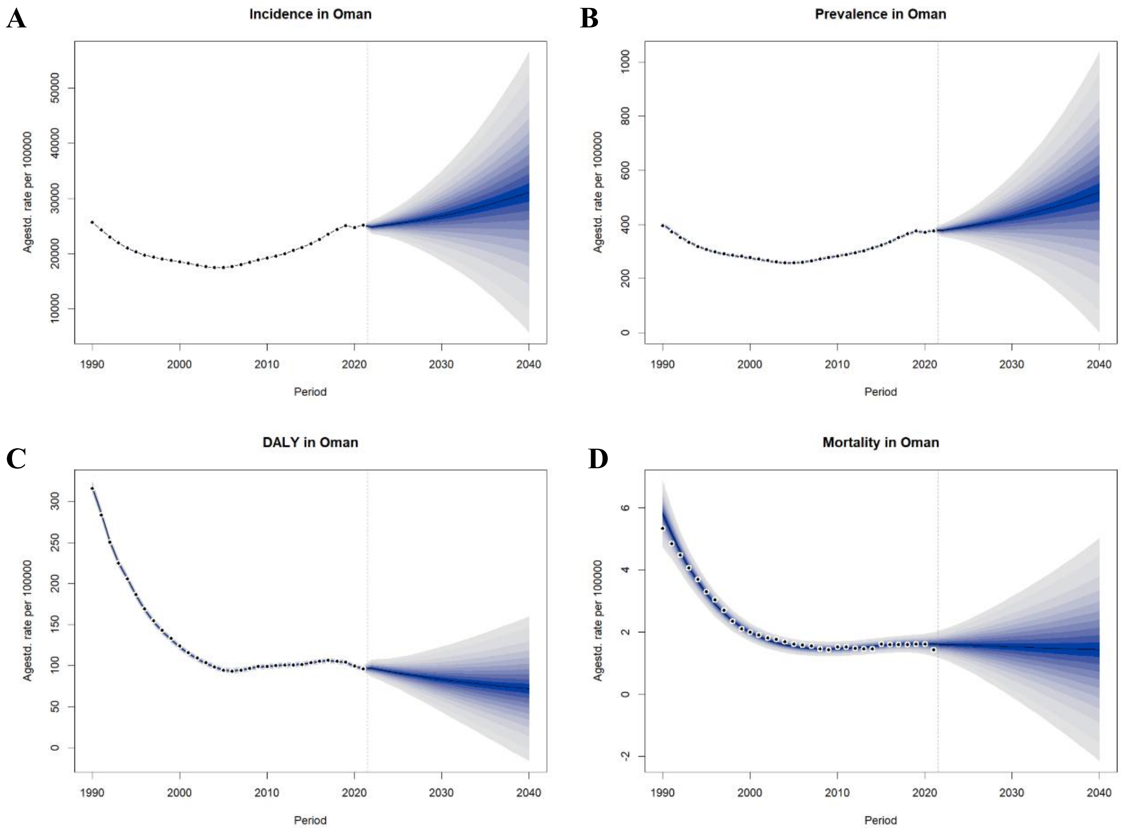

Figure S128: Prediction of diarrheal diseases burden in Oman from 2022 to 2040.

(A) Prediction of ASIR from 2022 to 2040; (B) Prediction of ASPR from 2022 to 2040; (C) Prediction of ASDR from 2022 to 2040; (D) Prediction of ASMR from 2022 to 2040.

DALY, disability-adjusted life year; ASIR, age-standardized incidence rate; ASPR, age-standardized prevalence rate; ASDR, age-standardized DALYs rate; ASMR, age-standardized mortality rate.

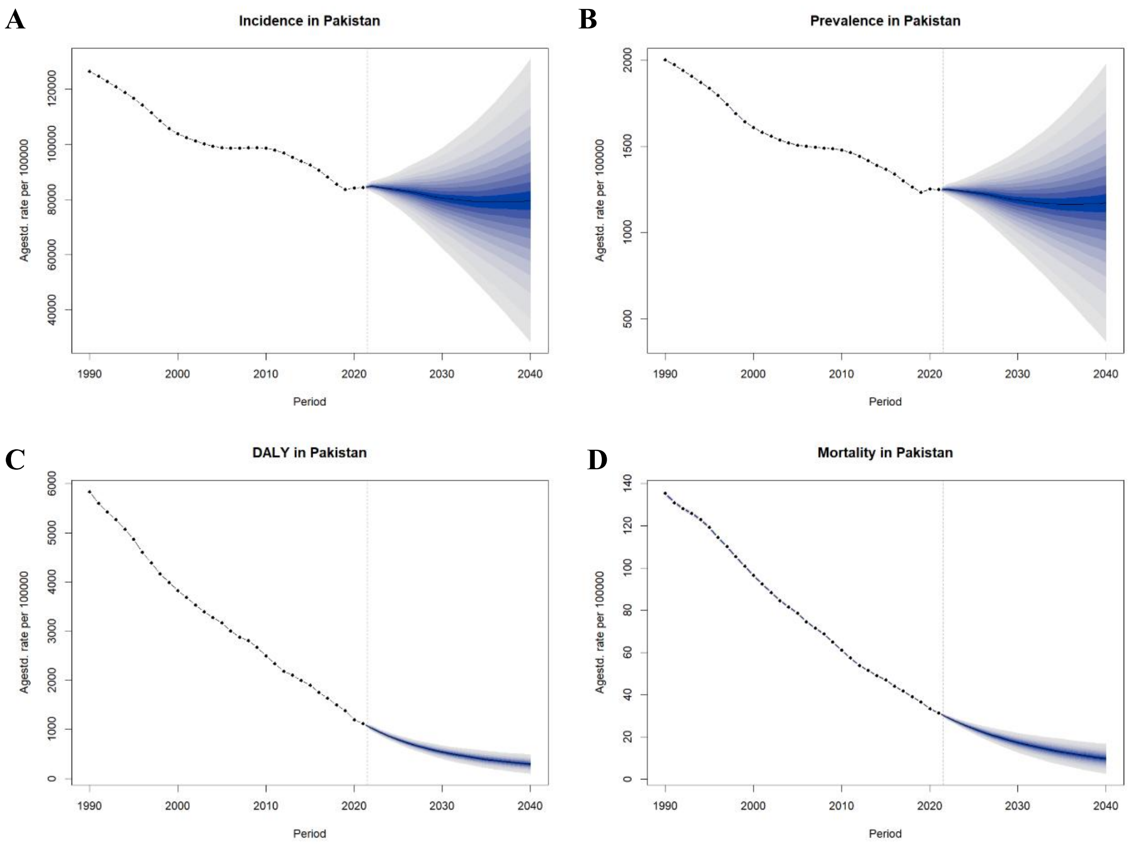

Figure S129: Prediction of diarrheal diseases burden in Pakistan from 2022 to 2040.

(A) Prediction of ASIR from 2022 to 2040; (B) Prediction of ASPR from 2022 to 2040; (C) Prediction of ASDR from 2022 to 2040; (D) Prediction of ASMR from 2022 to 2040.

DALY, disability-adjusted life year; ASIR, age-standardized incidence rate; ASPR, age-standardized prevalence rate; ASDR, age-standardized DALYs rate; ASMR, age-standardized mortality rate.

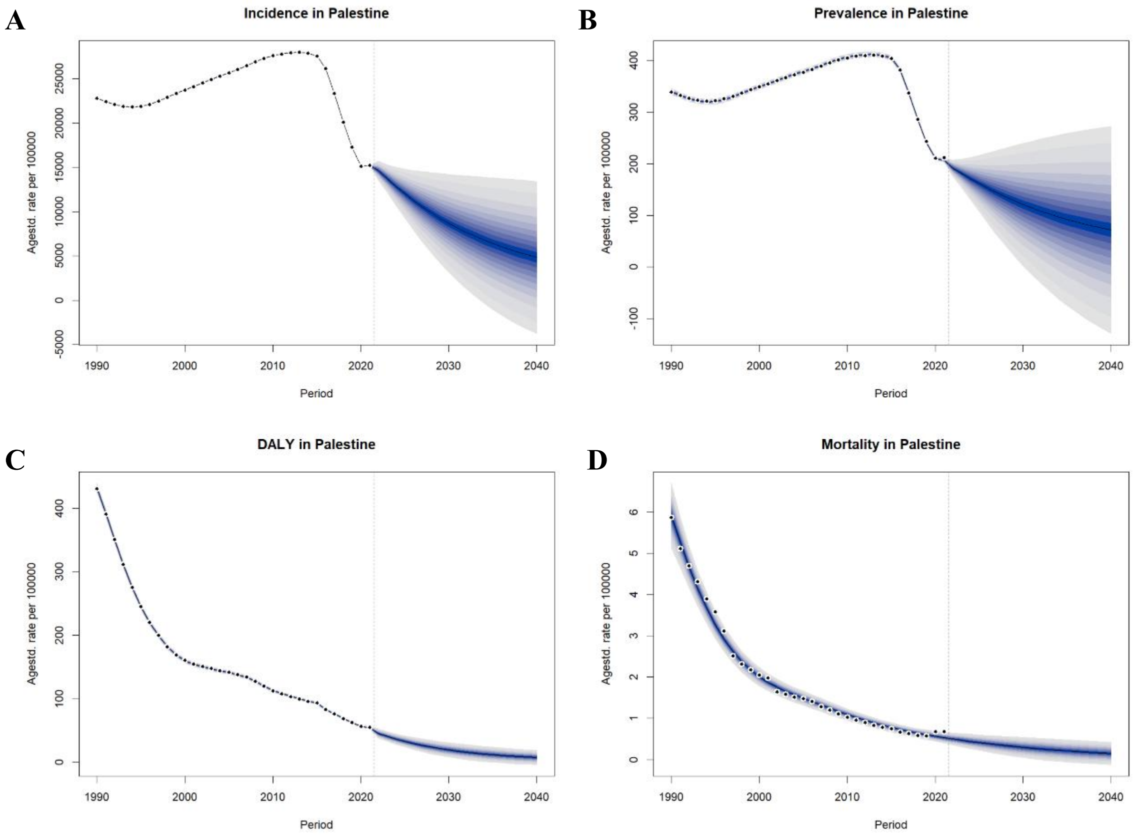

Figure S130: Prediction of diarrheal diseases burden in Palestine from 2022 to 2040.

(A) Prediction of ASIR from 2022 to 2040; (B) Prediction of ASPR from 2022 to 2040; (C) Prediction of ASDR from 2022 to 2040; (D) Prediction of ASMR from 2022 to 2040.

DALY, disability-adjusted life year; ASIR, age-standardized incidence rate; ASPR, age-standardized prevalence rate; ASDR, age-standardized DALYs rate; ASMR, age-standardized mortality rate.

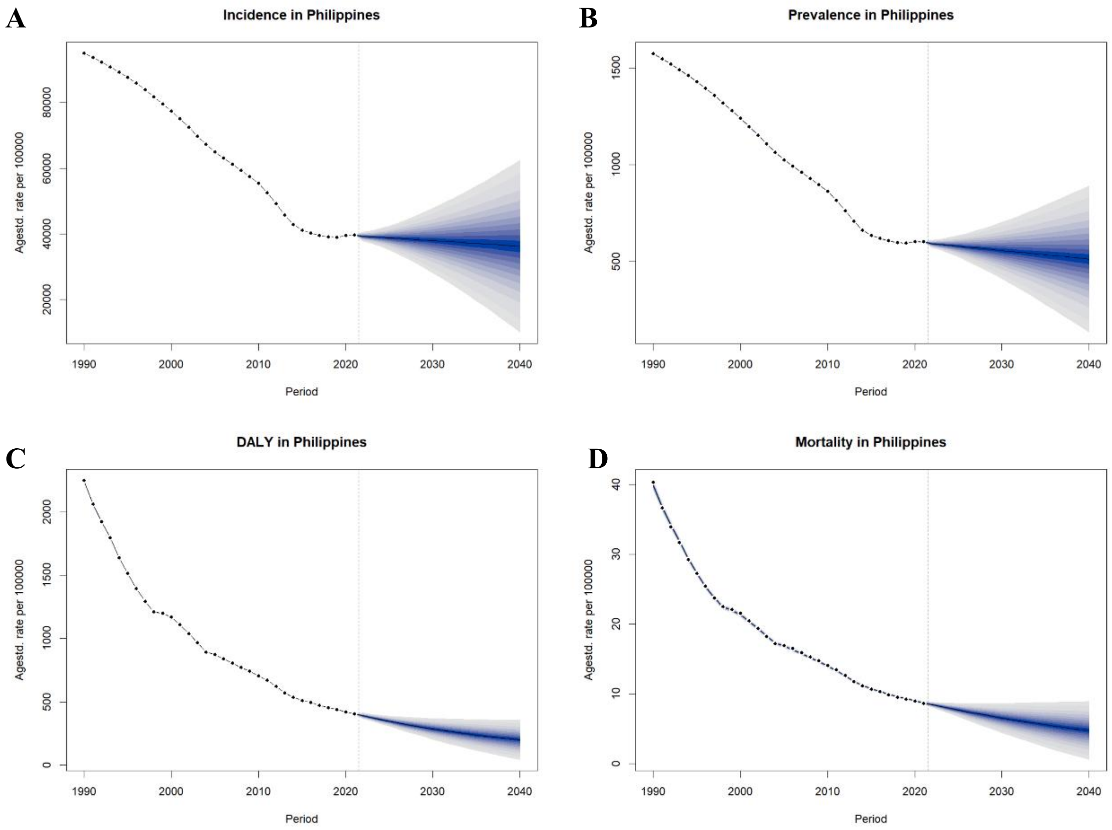

Figure S131: Prediction of diarrheal diseases burden in Philippines from 2022 to 2040.

(A) Prediction of ASIR from 2022 to 2040; (B) Prediction of ASPR from 2022 to 2040; (C) Prediction of ASDR from 2022 to 2040; (D) Prediction of ASMR from 2022 to 2040.

DALY, disability-adjusted life year; ASIR, age-standardized incidence rate; ASPR, age-standardized prevalence rate; ASDR, age-standardized DALYs rate; ASMR, age-standardized mortality rate.

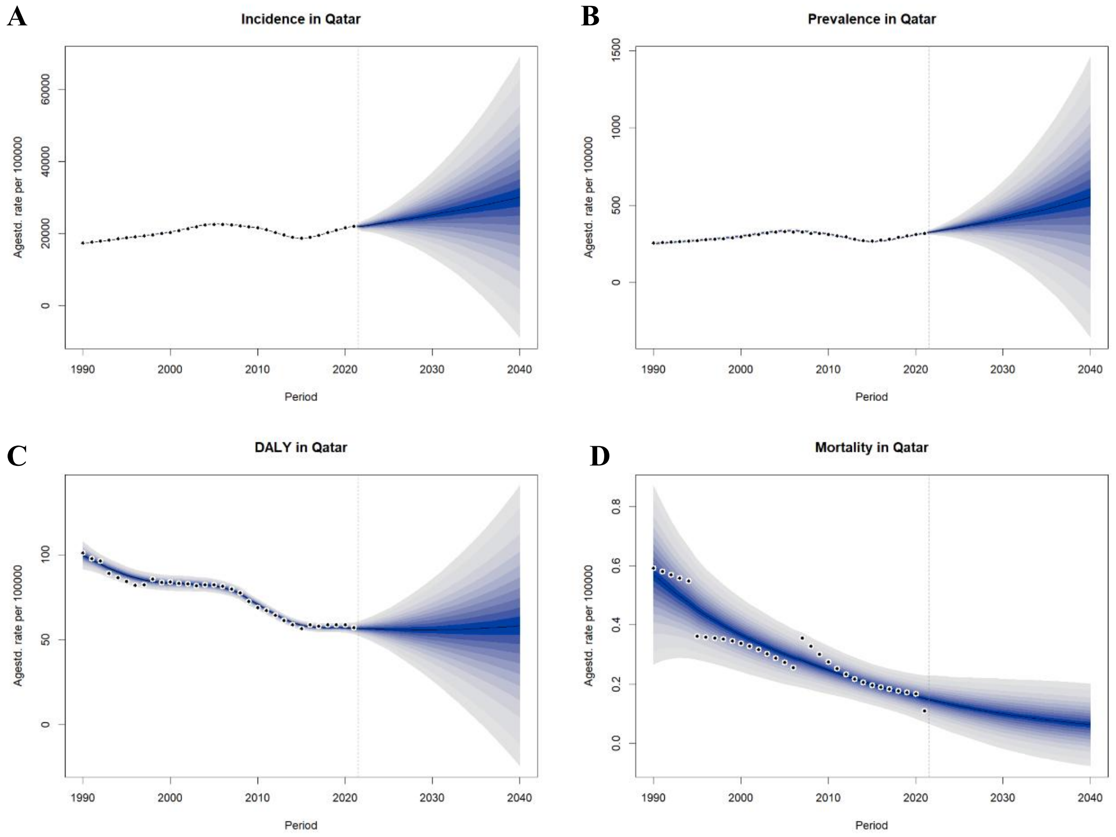

Figure S132: Prediction of diarrheal diseases burden in Qatar from 2022 to 2040.

(A) Prediction of ASIR from 2022 to 2040; (B) Prediction of ASPR from 2022 to 2040; (C) Prediction of ASDR from 2022 to 2040; (D) Prediction of ASMR from 2022 to 2040.

DALY, disability-adjusted life year; ASIR, age-standardized incidence rate; ASPR, age-standardized prevalence rate; ASDR, age-standardized DALYs rate; ASMR, age-standardized mortality rate.

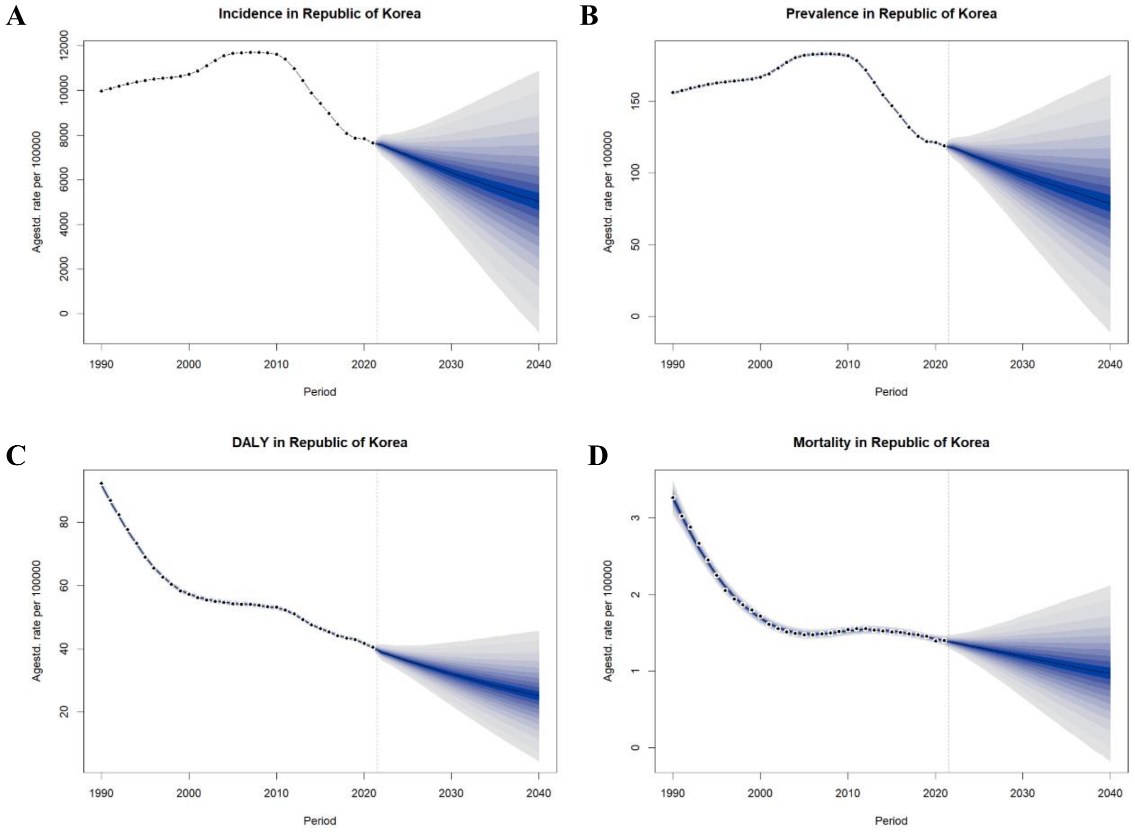

Figure S133: Prediction of diarrheal diseases burden in Republic of Korea from 2022 to 2040.

(A) Prediction of ASIR from 2022 to 2040; (B) Prediction of ASPR from 2022 to 2040; (C) Prediction of ASDR from 2022 to 2040; (D) Prediction of ASMR from 2022 to 2040.

DALY, disability-adjusted life year; ASIR, age-standardized incidence rate; ASPR, age-standardized prevalence rate; ASDR, age-standardized DALYs rate; ASMR, age-standardized mortality rate.

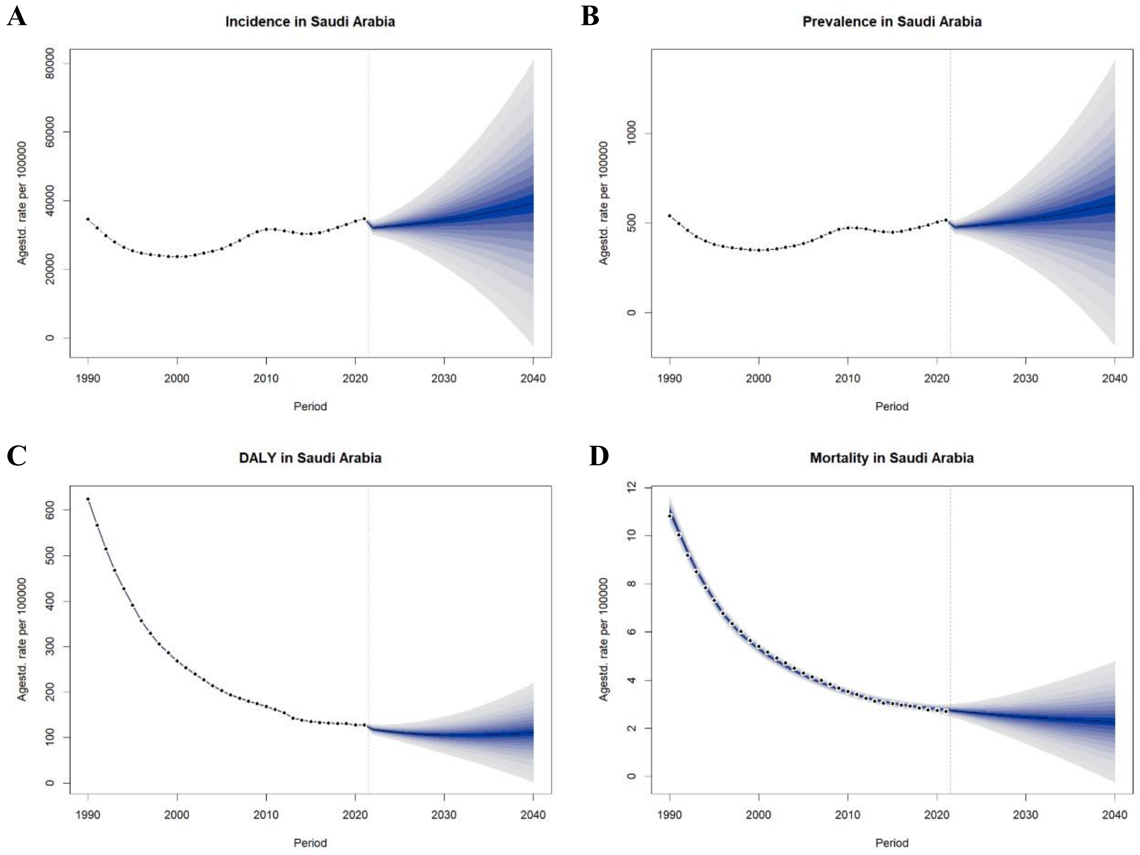

Figure S134: Prediction of diarrheal diseases burden in Saudi Arabia from 2022 to 2040.

(A) Prediction of ASIR from 2022 to 2040; (B) Prediction of ASPR from 2022 to 2040; (C) Prediction of ASDR from 2022 to 2040; (D) Prediction of ASMR from 2022 to 2040.

DALY, disability-adjusted life year; ASIR, age-standardized incidence rate; ASPR, age-standardized prevalence rate; ASDR, age-standardized DALYs rate; ASMR, age-standardized mortality rate.

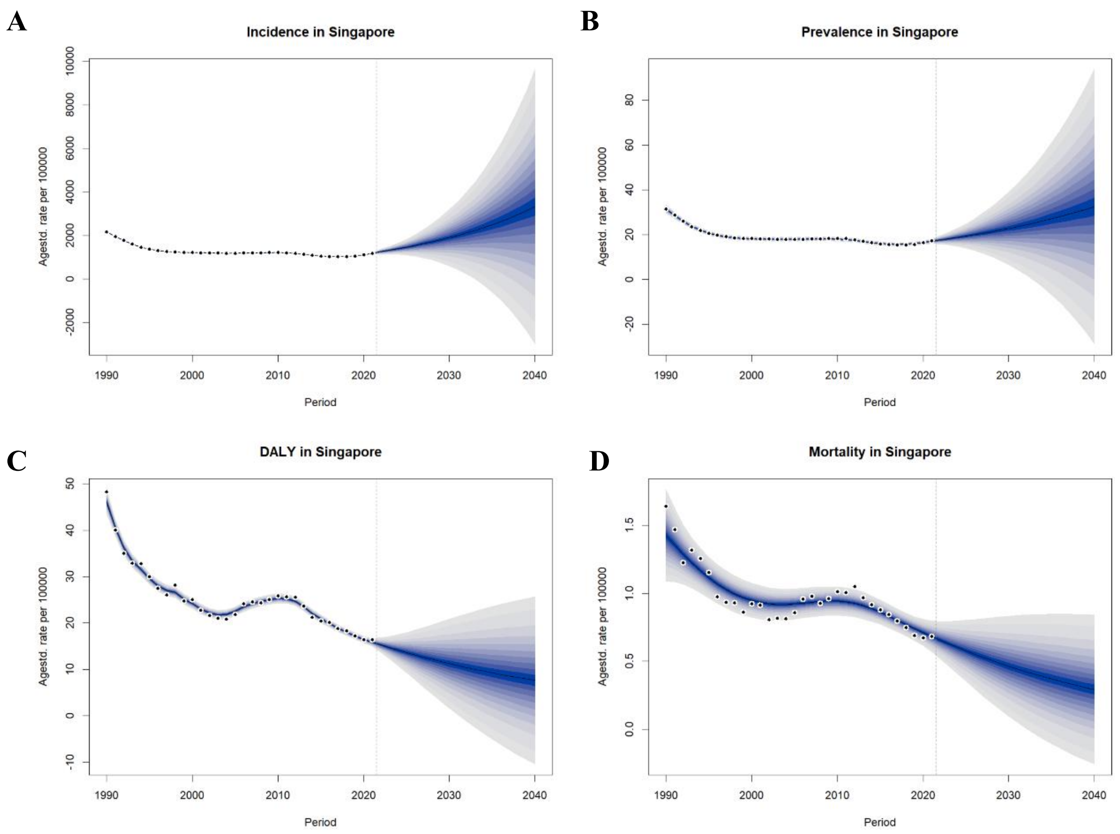

Figure S135: Prediction of diarrheal diseases burden in Singapore from 2022 to 2040.

(A) Prediction of ASIR from 2022 to 2040; (B) Prediction of ASPR from 2022 to 2040; (C) Prediction of ASDR from 2022 to 2040; (D) Prediction of ASMR from 2022 to 2040.

DALY, disability-adjusted life year; ASIR, age-standardized incidence rate; ASPR, age-standardized prevalence rate; ASDR, age-standardized DALYs rate; ASMR, age-standardized mortality rate.

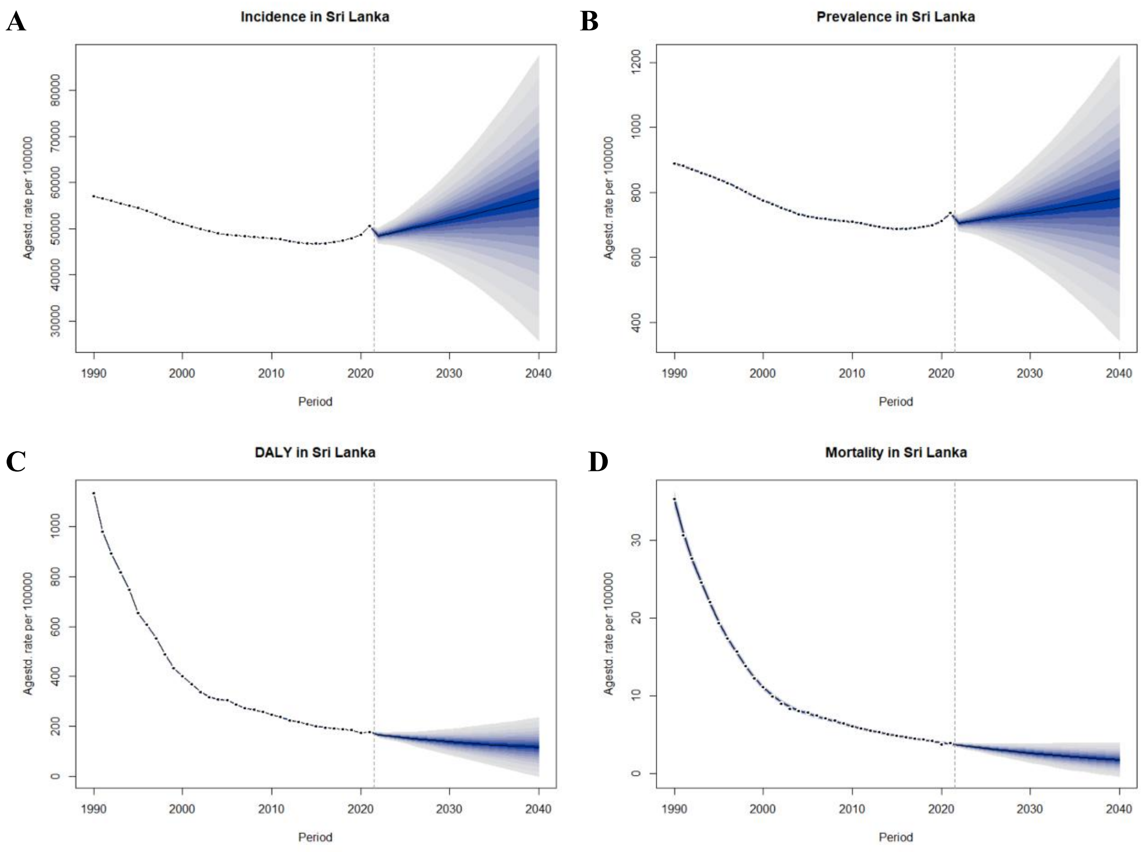

Figure S136: Prediction of diarrheal diseases burden in Sri Lanka from 2022 to 2040.

(A) Prediction of ASIR from 2022 to 2040; (B) Prediction of ASPR from 2022 to 2040; (C) Prediction of ASDR from 2022 to 2040; (D) Prediction of ASMR from 2022 to 2040.

DALY, disability-adjusted life year; ASIR, age-standardized incidence rate; ASPR, age-standardized prevalence rate; ASDR, age-standardized DALYs rate; ASMR, age-standardized mortality rate.

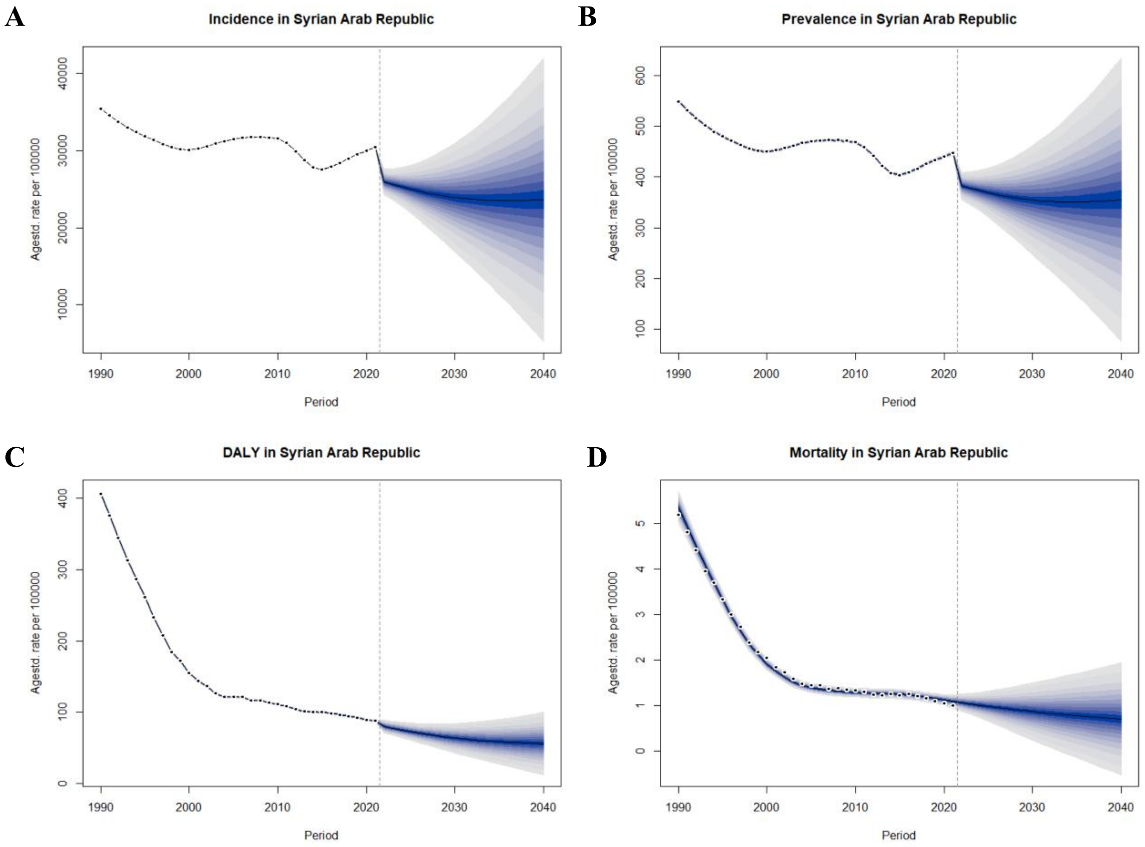

Figure S137: Prediction of diarrheal diseases burden in Syrian Arab Republic from 2022 to 2040.

(A) Prediction of ASIR from 2022 to 2040; (B) Prediction of ASPR from 2022 to 2040; (C) Prediction of ASDR from 2022 to 2040; (D) Prediction of ASMR from 2022 to 2040.

DALY, disability-adjusted life year; ASIR, age-standardized incidence rate; ASPR, age-standardized prevalence rate; ASDR, age-standardized DALYs rate; ASMR, age-standardized mortality rate.

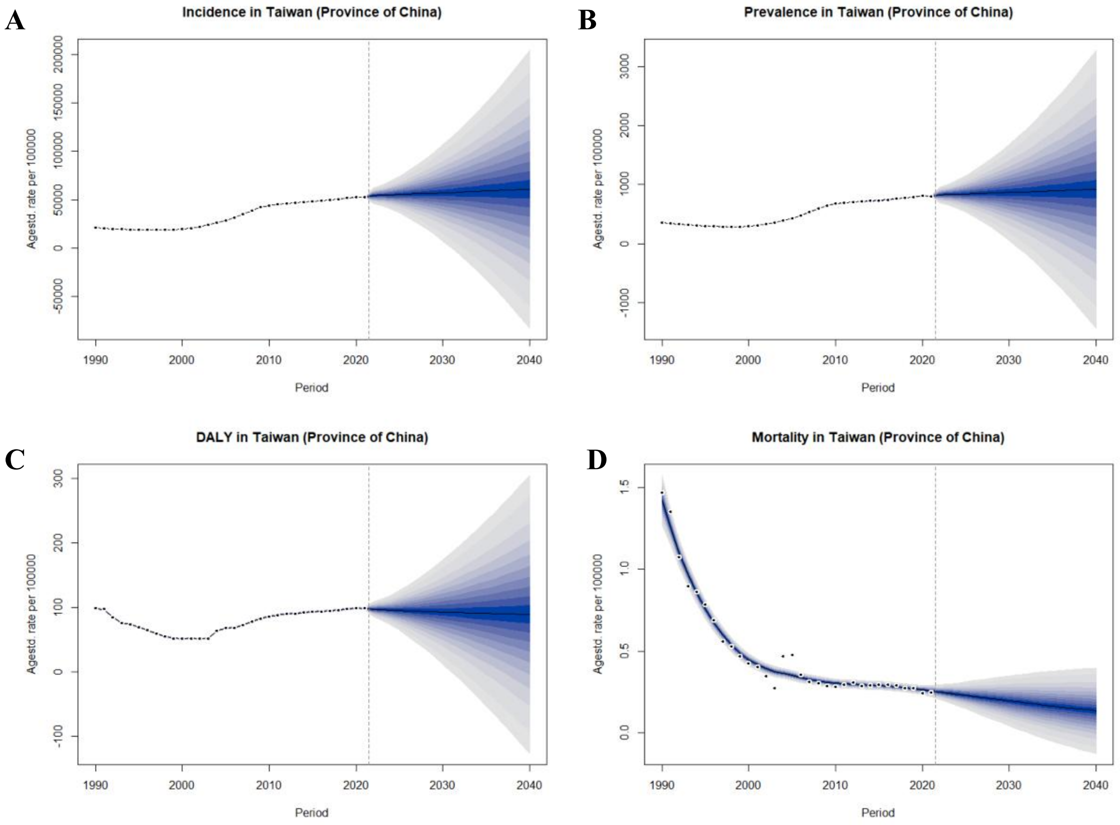

Figure S138: Prediction of diarrheal diseases burden in Taiwan (Province of China) from 2022 to 2040.

(A) Prediction of ASIR from 2022 to 2040; (B) Prediction of ASPR from 2022 to 2040; (C) Prediction of ASDR from 2022 to 2040; (D) Prediction of ASMR from 2022 to 2040.

DALY, disability-adjusted life year; ASIR, age-standardized incidence rate; ASPR, age-standardized prevalence rate; ASDR, age-standardized DALYs rate; ASMR, age-standardized mortality rate.

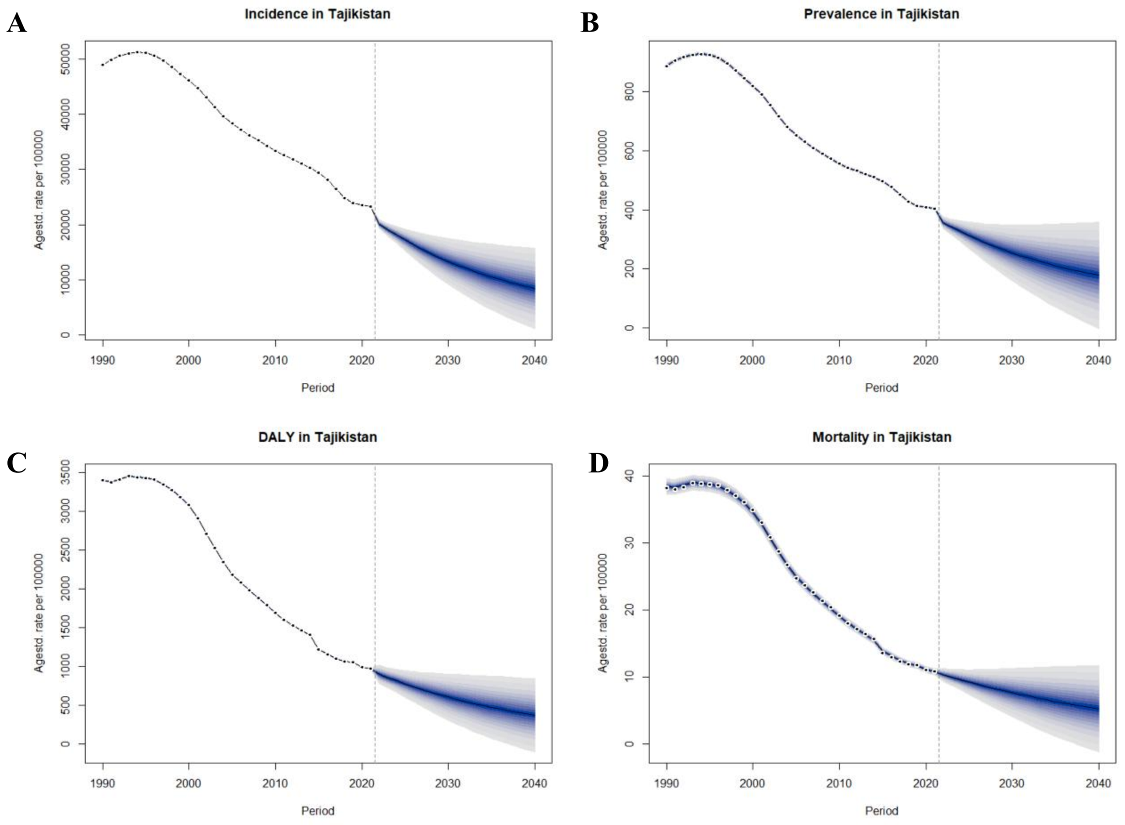

Figure S139: Prediction of diarrheal diseases burden in Tajikistan from 2022 to 2040.

(A) Prediction of ASIR from 2022 to 2040; (B) Prediction of ASPR from 2022 to 2040; (C) Prediction of ASDR from 2022 to 2040; (D) Prediction of ASMR from 2022 to 2040.

DALY, disability-adjusted life year; ASIR, age-standardized incidence rate; ASPR, age-standardized prevalence rate; ASDR, age-standardized DALYs rate; ASMR, age-standardized mortality rate.

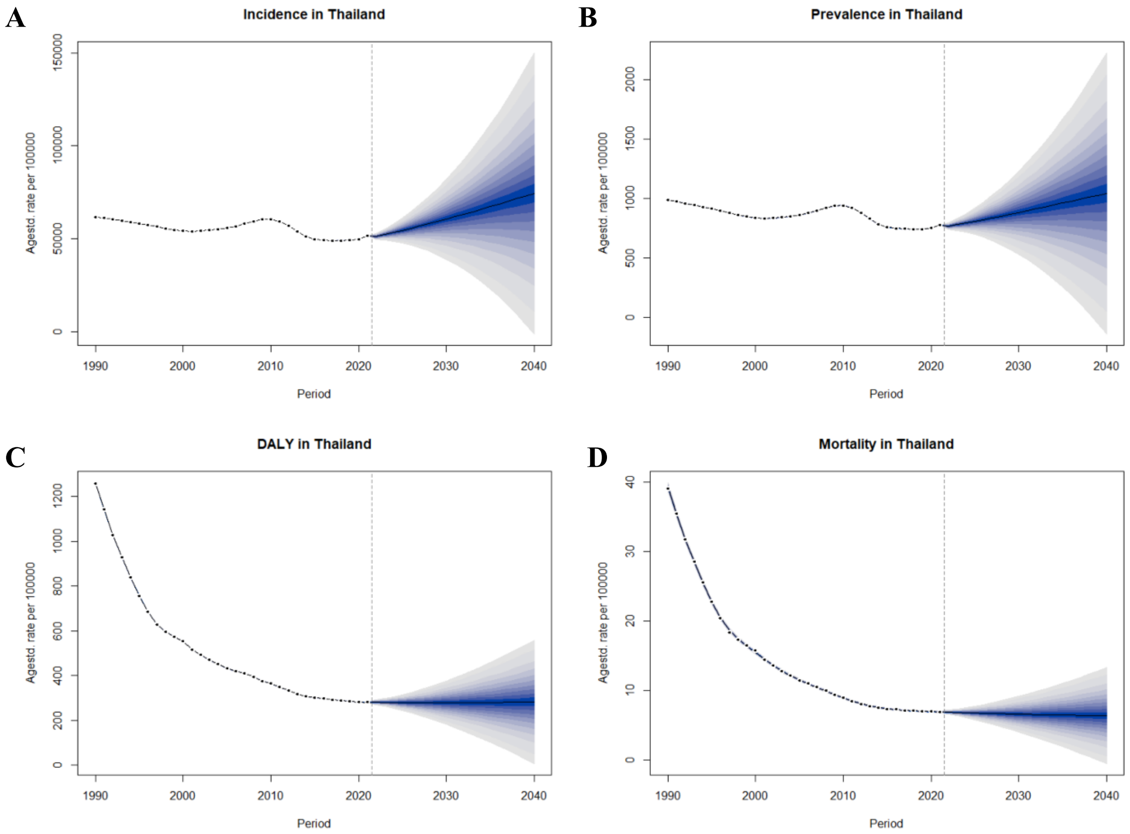

Figure S140: Prediction of diarrheal diseases burden in Thailand from 2022 to 2040.

(A) Prediction of ASIR from 2022 to 2040; (B) Prediction of ASPR from 2022 to 2040; (C) Prediction of ASDR from 2022 to 2040; (D) Prediction of ASMR from 2022 to 2040.

DALY, disability-adjusted life year; ASIR, age-standardized incidence rate; ASPR, age-standardized prevalence rate; ASDR, age-standardized DALYs rate; ASMR, age-standardized mortality rate.

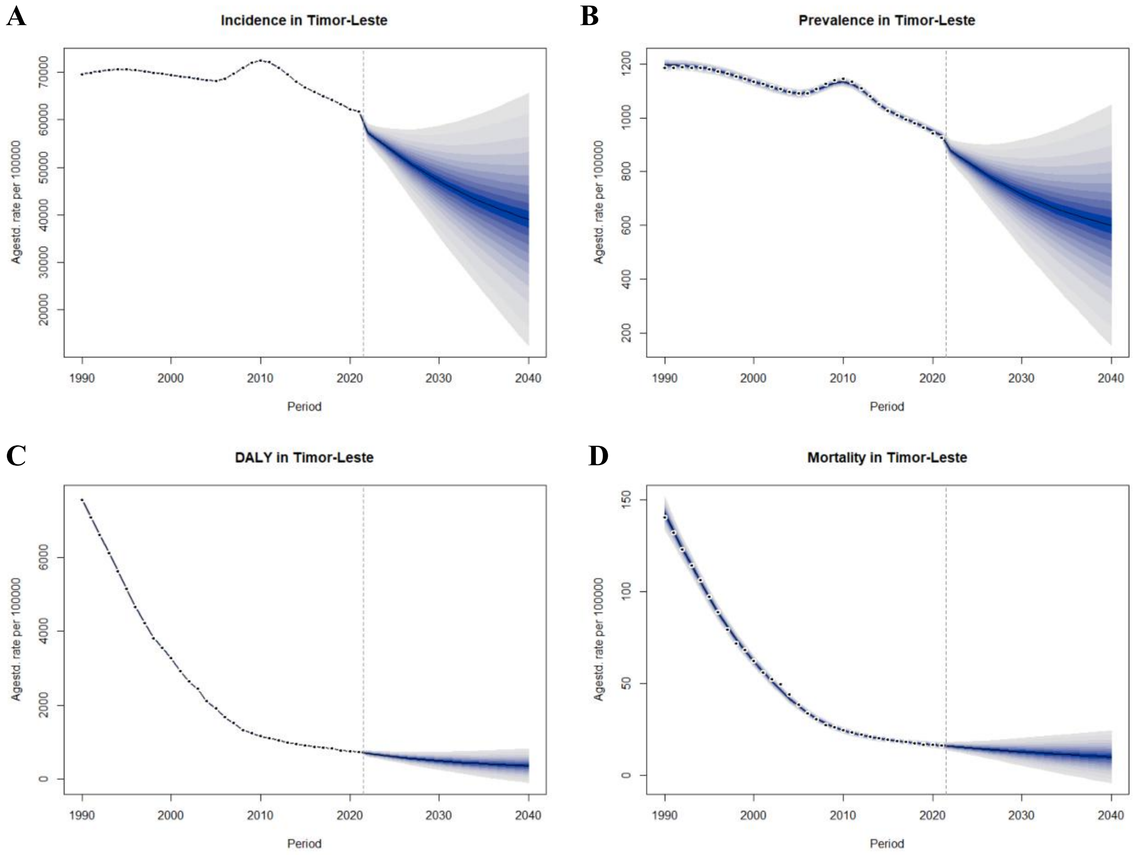

Figure S141: Prediction of diarrheal diseases burden in Timor-Leste from 2022 to 2040.

(A) Prediction of ASIR from 2022 to 2040; (B) Prediction of ASPR from 2022 to 2040; (C) Prediction of ASDR from 2022 to 2040; (D) Prediction of ASMR from 2022 to 2040.

DALY, disability-adjusted life year; ASIR, age-standardized incidence rate; ASPR, age-standardized prevalence rate; ASDR, age-standardized DALYs rate; ASMR, age-standardized mortality rate.

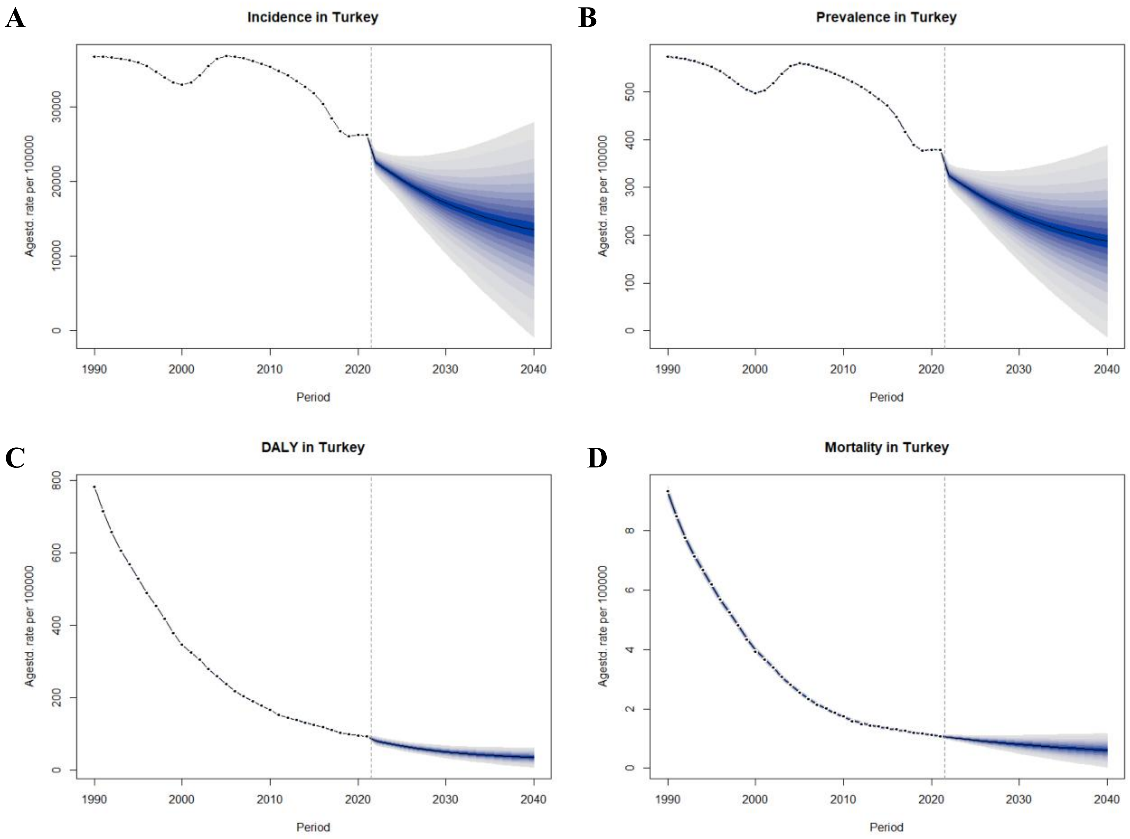

Figure S142: Prediction of diarrheal diseases burden in Turkey from 2022 to 2040.

(A) Prediction of ASIR from 2022 to 2040; (B) Prediction of ASPR from 2022 to 2040; (C) Prediction of ASDR from 2022 to 2040; (D) Prediction of ASMR from 2022 to 2040.

DALY, disability-adjusted life year; ASIR, age-standardized incidence rate; ASPR, age-standardized prevalence rate; ASDR, age-standardized DALYs rate; ASMR, age-standardized mortality rate.

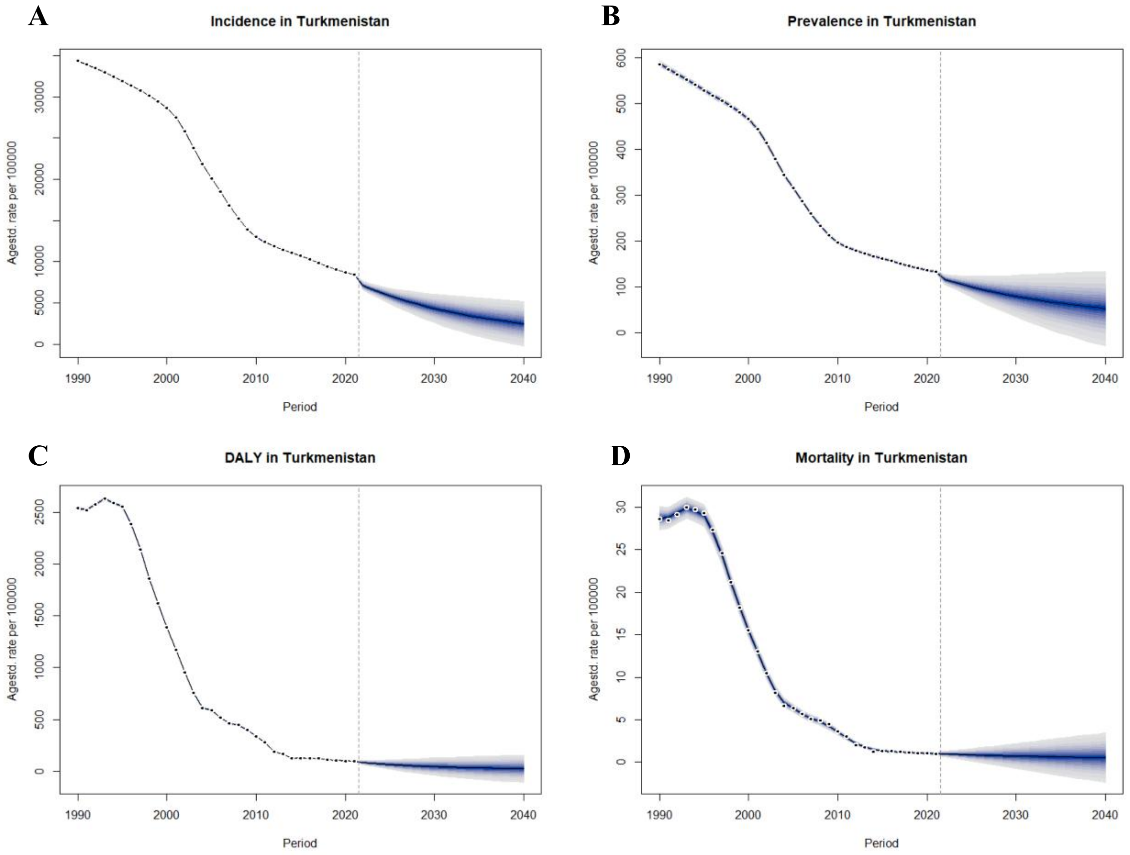

Figure S143: Prediction of diarrheal diseases burden in Turkmenistan from 2022 to 2040.

(A) Prediction of ASIR from 2022 to 2040; (B) Prediction of ASPR from 2022 to 2040; (C) Prediction of ASDR from 2022 to 2040; (D) Prediction of ASMR from 2022 to 2040.

DALY, disability-adjusted life year; ASIR, age-standardized incidence rate; ASPR, age-standardized prevalence rate; ASDR, age-standardized DALYs rate; ASMR, age-standardized mortality rate.

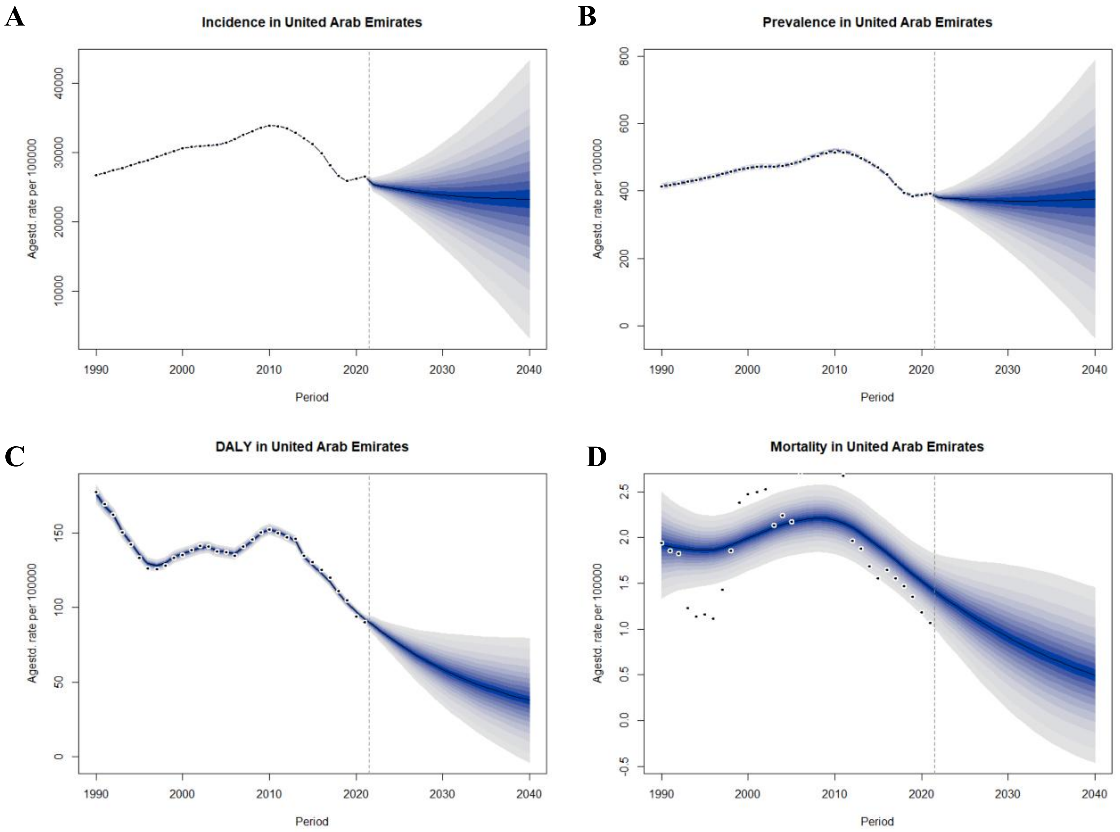

Figure S144: Prediction of diarrheal diseases burden in United Arab Emirates from 2022 to 2040.

(A) Prediction of ASIR from 2022 to 2040; (B) Prediction of ASPR from 2022 to 2040; (C) Prediction of ASDR from 2022 to 2040; (D) Prediction of ASMR from 2022 to 2040.

DALY, disability-adjusted life year; ASIR, age-standardized incidence rate; ASPR, age-standardized prevalence rate; ASDR, age-standardized DALYs rate; ASMR, age-standardized mortality rate.

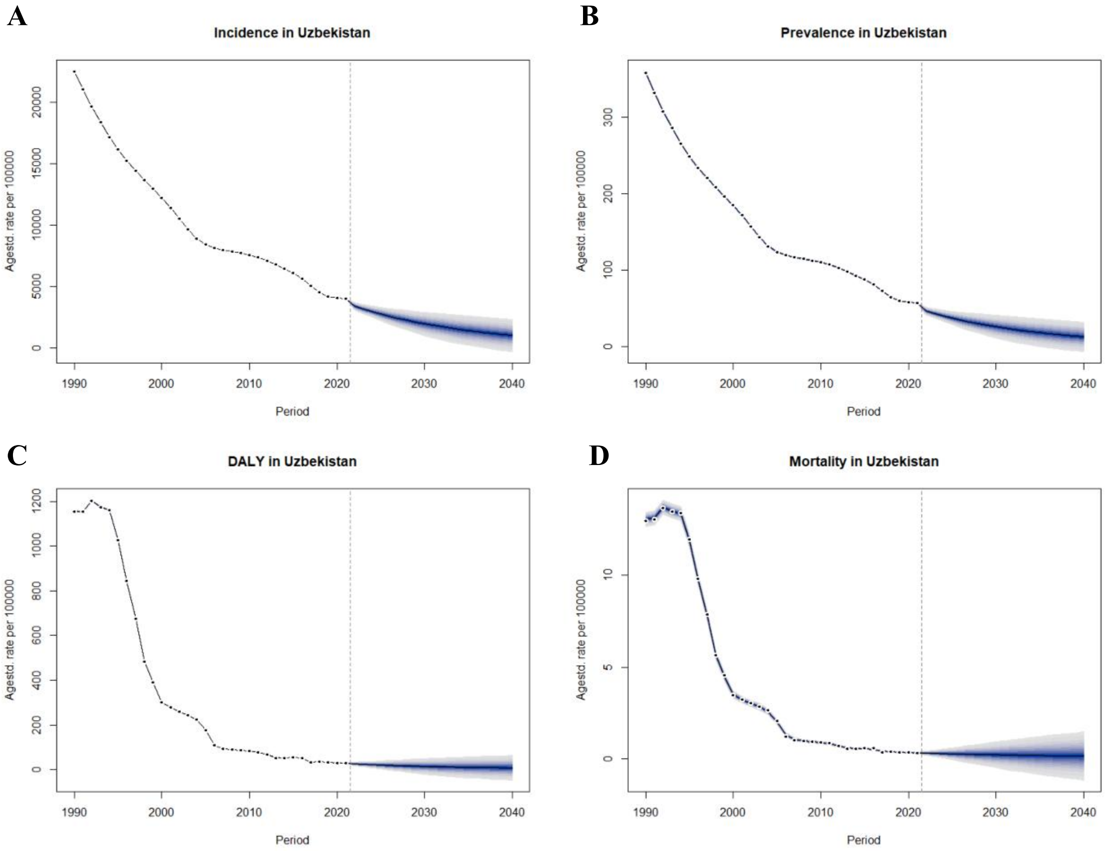

Figure S145: Prediction of diarrheal diseases burden in Uzbekistan from 2022 to 2040.

(A) Prediction of ASIR from 2022 to 2040; (B) Prediction of ASPR from 2022 to 2040; (C) Prediction of ASDR from 2022 to 2040; (D) Prediction of ASMR from 2022 to 2040.

DALY, disability-adjusted life year; ASIR, age-standardized incidence rate; ASPR, age-standardized prevalence rate; ASDR, age-standardized DALYs rate; ASMR, age-standardized mortality rate.

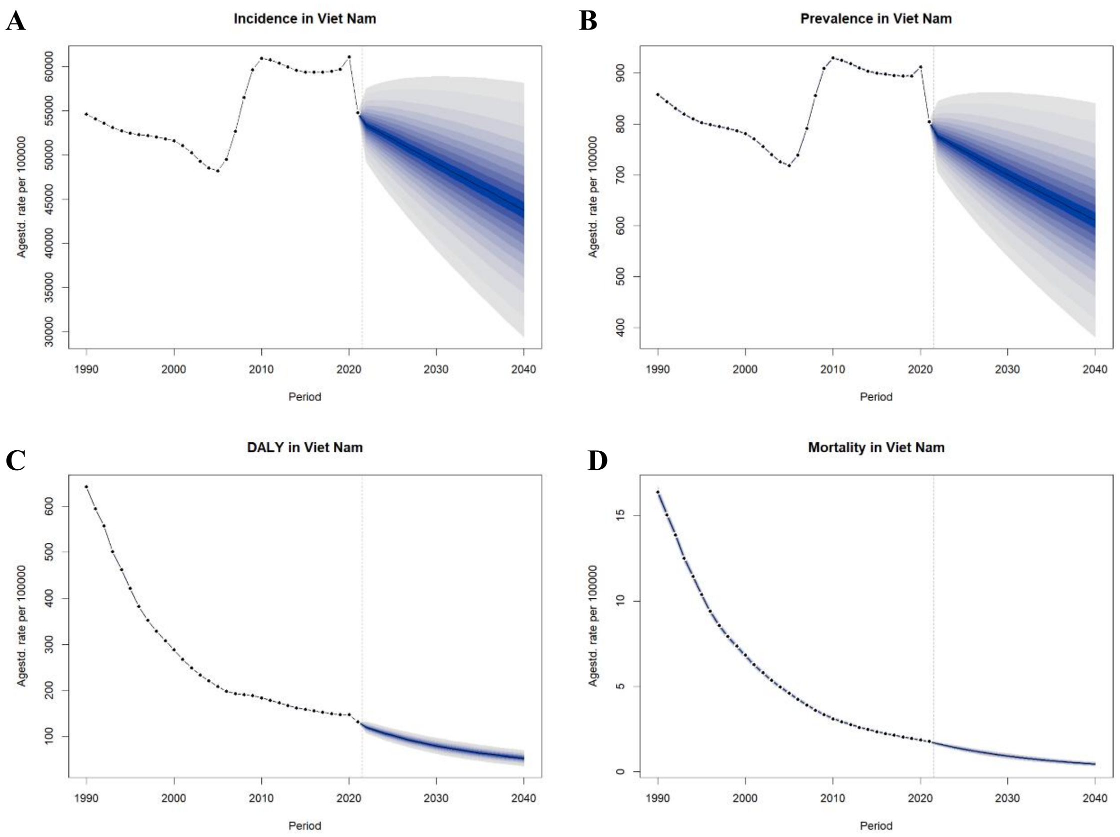

Figure S146: Prediction of diarrheal diseases burden in Viet Nam from 2022 to 2040.

(A) Prediction of ASIR from 2022 to 2040; (B) Prediction of ASPR from 2022 to 2040; (C) Prediction of ASDR from 2022 to 2040; (D) Prediction of ASMR from 2022 to 2040.

DALY, disability-adjusted life year; ASIR, age-standardized incidence rate; ASPR, age-standardized prevalence rate; ASDR, age-standardized DALYs rate; ASMR, age-standardized mortality rate.

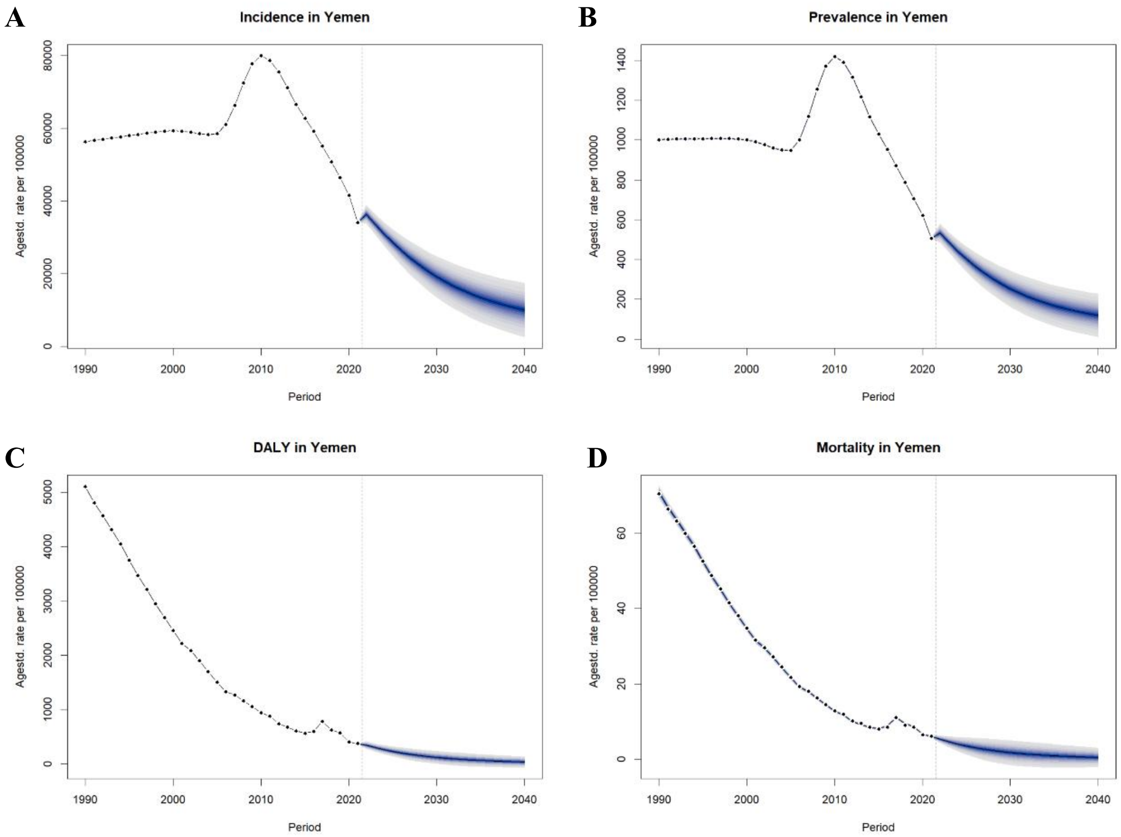

Figure S147: Prediction of diarrheal diseases burden in Yemen from 2022 to 2040.

(A) Prediction of ASIR from 2022 to 2040; (B) Prediction of ASPR from 2022 to 2040; (C) Prediction of ASDR from 2022 to 2040; (D) Prediction of ASMR from 2022 to 2040.

DALY, disability-adjusted life year; ASIR, age-standardized incidence rate; ASPR, age-standardized prevalence rate; ASDR, age-standardized DALYs rate; ASMR, age-standardized mortality rate.
