# Supplementary material for: The burden and etiologies of diarrhea in Asia and its countries from 1990 to 2021 and the forecast to 2040: analyses informed by the global burden of disease study 2021
Source: Front Public Health. 2025 Aug 6;13:1651315. doi: 10.3389/fpubh.2025.1651315 (PMC12364947; doi:10.3389/fpubh.2025.1651315)
Supplement: Supplementary file 2 [file Table_1.DOCX]

**Table S1** Analysis of burdens of diarrhoeal disease in Asian countries from 1990 to 2021.

|  | **1990** | | **2021** | | **1990-2021** |
| --- | --- | --- | --- | --- | --- |
|  | **Cases**  **No. (95%UI)** | **Age-standardized rate per 100,000**  **No. (95%UI)** | **Cases**  **No. (95%UI)** | **Age-standardized rate per 100,000**  **No. (95%UI)** | **EAPC**  **No. (95%CI)** |
| **Incidence** |  |  |  |  |  |
| Afghanistan | 4,560,012.13  (3,720,005.24 to 5,402,394.88) | 29,745.80  (24,351.11 to 35,349.90) | 9,390,999.42  (7,612,355.08 to 11,398,930.55) | 21,949.66  (17,913.75 to 26,487.01) | -0.70  (-1.08 to -0.31) |
| Armenia | 1,040,613.24  (878,931.37 to 1,218,533.56) | 28,671.53  (24,218.08to 33,629.50) | 1,94,590.55  (158,114.20 to 234,008.18) | 7,911.12  (6,251.08 to 9,629.79) | -4.04  (-4.86 to -3.22) |
| Azerbaijan | 1,813,601.84  (1,550,032.92 to 2,105,198.84) | 21,443.65  (18,378.96 to 24,833.26) | 1,165,876.63  (940,015.29 to 1,408,633.14) | 12,498.70  (9,971.94 to 15,323.75) | -1.97  (-2.22 yo -1.73) |
| Bahrain | 109,622.01  (83,553.46 to 138,951.35) | 20,995.13  (16,366.13 to 26,330.37) | 300,502.77  (244,308.82 to 362,656.55) | 24,157.10  (19,488.44 to 29,232.11) | 0.07  (-0.30 to 0.44) |
| Bangladesh | 146,839,970.40  (127,053,871.40 to 166,390,538.50) | 104,236.29  (91,879.26 to 117,495.07) | 95,629,822.31  (84,833,049.06 to 107,515,096.10) | 57,555.70  (51,117.41 to 64,561.91) | -1.67  (-1.77 to -1.56) |
| Bhutan | 502,540.00  (431,152.78 to 576,982.20) | 74,156.49  (64,888.27 to 83,943.68) | 428,091.16  (380,842.94 to 483,334.30) | 58,591.87  (52,114.66 to 66,354.10) | -1.20  (-1.39 to -1.02) |
| Brunei Darussalam | 23,108.30  (17,899.85 to 28,206.95) | 7,802.70  (6,111.69 to 9,499.77) | 24,319.99  (19,508.42 to 29,587.40) | 5,823.68  (4,505.22 to 7,139.25) | -0.96  (-1.34 to -0.57) |
| Cambodia | 8,928,183.13  (7,843,300.40 to 10,091,921.41) | 62,928.76  (55,928.42 to 70,564.97) | 7,376,649.04  (6,345,873.44 to 8,529,857.15) | 43,496.34  (37,813.10 to 49,956.76) | -1.55  (-1.79 to -1.32) |
| China | 290,107,918.90  (239,596,093.60 to 343,888,620.50) | 25,458.31  (21,095.18 to 30,121.15) | 75,037,050.53  (63,261,023.05 to 89,043,888.41) | 5,894.36  (4,820.23 to 7,058.86) | -5.48  (-5.93 to -5.03) |
| Cyprus | 218,720.55  (177,011.05 to 268,719.67) | 30,222.60  (24,235.75 to 37,479.55) | 422,187.12  (356,029.29 to 494,596.29) | 35,271.86  (28,535.69 to 42,753.00) | 1.10  (0.58 to 1.62) |
| Democratic People's  Republic of Korea | 3,835,185.41  (3,170,519.58 to 4,506,078.76) | 17,492.19  (14,450.74 to 20,567.35) | 14,734,809.33  (12,074,982.97 to 17,845,342.36) | 62,969.13  (51,190.05 to 76,385.54) | 5.48  (4.85 to 6.11) |
| Georgia | 1,284,130.59  (1,076,929.83 to 1,516,049.56) | 26,171.95  (21,898.32 to 30,871.36) | 315,953.05  (264,604.73 to 372,235.39) | 10,599.83  (8,830.80 to 12,639.36) | -2.10  (-2.81 to -1.38) |
| India | 1,195,128,112.00  (1,008,092,682.00 to 1,365,734,656.00) | 131,781.54  (115,054.45 to 148,777.47) | 1,892,784,379.00  (1,681,365,634.00 to 2,123,201,151.00) | 135,663.73  (121,215.14 to 151,088.39) | -0.02  (-0.08 to 0.04) |
| Indonesia | 208,792,494.90  (178,723,932.90 to 242,406,822.00) | 108,721.73  (95,048.55 to 124,554.91) | 152,667,385.10  (134,724,996.40 to 170,998,565.50) | 60,510.08  (53,612.65 to 67,659.13) | -1.95  (-2.02 to -1.87) |
| Iran  (Islamic Republic of) | 39,386,818.61  (30,459,199.31 to 48,025,850.62) | 49,662.42  (39,603.36 to 59,302.12) | 20,632,723.21  (16,646,115.17 to 25,506,459.81) | 25,443.41  (20,356.20 to 31,683.30) | -2.15  (-2.38 to -1.91) |
| Iraq | 11,286,966.61  (9,293,854.32 to 13,402,253.66) | 41,737.23  (34,844.34 to 49,168.82) | 12,082,765.90  (9,628,724.45 to 15,017,732.96) | 27,327.42  (22,324.33 to 33,426.71) | -1.20  (-1.43 to -0.97) |
| Israel | 1,400,645.70  (1,108,629.54 to 1,742,776.75) | 27,393.57  (22,004.19 to 33,869.00) | 2,422,696.89  (2,081,574.63 to 2,785,549.25) | 23,247.59  (19,638.82 to 26,981.58) | -0.07  (-0.94 to 0.79) |
| Japan | 37,277,371.76  (30,377,869.66 to 44,409,804.74) | 36,230.04  (29,038.80 to 43,981.21) | 50,547,588.54  (43,482,461.52 to 58,339,457.45) | 52,056.72  (42,482.47 to 62,453.02) | 1.73  (1.35 to 2.12) |
| Jordan | 1,344,399.59  (1,084,756.42 to 1,620,192.30) | 25,641.66  (20,990.93 to 30,556.41) | 2,563,498.80  (2,008,175.27 to 3,188,940.72) | 20,714.22  (16,702.27 to 25,032.93) | -0.09  (-0.62 to 0.45) |
| Kazakhstan | 3,676,923.45  (3,118,021.03 to 4,221,355.78) | 20,505.23  (17,417.66 to 23,548.65) | 1,663,955.90  (1,307,115.00 to 2,066,981.57) | 8,836.52  (6,893.67 to 11,025.44) | -2.61  (-2.84 -2.38) |
| Kuwait | 520,653.57  (397,924.91 to 646,145.03) | 27,796.36  (21,582.29 to 34,146.83) | 1,006,205.43  (801,790.93 to 1,229,271.02) | 25,521.68  (19,998.09 to 31,728.89) | 0.67  (0.45 to 0.90) |
| Kyrgyzstan | 1,308,208.11  (1,126,020.62 to 1,507,790.11) | 22,444.31  (19,264.74 to 25,900.81) | 569,930.07  (455,357.12 to 698,754.36) | 7,675.73  (6,186.05 to 9,350.69) | -2.81  (-3.05 to -2.58) |
| Lao People's  Democratic Republic | 3,590,293.59  (3,218,422.50 to 3,997,721.93) | 62,188.25  (55,868.51 to 69,032.93) | 3,784,081.74  (3,330,208.87 to 4,258,035.98) | 50,233.37  (44,154.47 to 56,298.02) | -0.62  (-0.72 to -0.51) |
| Lebanon | 926,366.26  (720,132.04 to 1,135,043.07) | 26,877.77  (21,167.93 to 32,645.13) | 1,963,987.26  (1,573,202.31 to 2,414,124.97) | 38,164.35  (30,105.65 to 47,353.59) | 0.85  (0.67 to 1.03) |
| Malaysia | 6,742,574.10  (5,547,516.18 to 8,098,475.54) | 34,228.58  (28,820.30 to 40,654.77) | 22,767,935.78  (18,709,474.91 to 27,397,367.98) | 75,561.89  (61,794.00 to 90,578.66) | 2.84  (2.70 to 2.97) |
| Maldives | 170,199.21  (147,721.15 to 193,602.56) | 52,589.71  (46,686.19 to 58,992.55) | 263,562.56  (222,762.65 to 309,942.55) | 59,074.79  (49,407.54 to 69,773.19) | 0.30  (0.21 to 0.39) |
| Mongolia | 563,295.83  (455,389.65 to 678,468.19) | 17,769.04  (14,418.14 to 21,279.61) | 95,672.86  (81,535.61 to 110,078.30) | 2,649.83  (2,267.84 to 3,044.34) | -5.72  (-6.85 to -4.59) |
| Myanmar | 26,924,860.09  (24,099,245.07 to 30,271,819.40) | 57,986.82  (52,191.57 to 64,641.93) | 24,109,191.78  (21,066,717.81 to 27,265,385.38) | 43,924.22  (38,529.35 to 49,461.53) | -1.19  (-1.34 to -1.04) |
| Nepal | 20,407,314.99  (18,008,223.34 to 23,120,242.11) | 84,227.20  (75,093.03 to 94,187.79) | 14,832,549.59  (13,260,812.62 to 16,572,375.40) | 48,460.72  (43,679.60 to 53,836.18) | -1.32  (-1.58 to -1.06) |
| Oman | 638,456.56  (526,746.72 to 751,762.00) | 25,598.06  (21,799.12 to 29,870.84) | 983,101.03  (822,397.79 to 1,167,027.77) | 25,240.49  (21,582.13 to 29,923.23) | 0.31  (-0.14 to 0.77) |
| Pakistan | 166,360,597.40  (140,346,841.20 to 192,518,604.90) | 126,277.45  (109,139.77 to 144,475.67) | 205,517,481.00  (176,492,606.50 to 237,929,043.60) | 84,353.41  (74,031.41 to 95,743.39) | -1.21  (-1.31 to -1.11) |
| Palestine | 682,946.85  (534,636.93 to 826,914.07) | 22,726.77  (18,163.41 to 27,266.21) | 856,687.85  (651,939.89 to 1,092,885.04) | 15,222.34  (12,041.77 to 19,021.67) | -0.17  (-0.78 to 0.43) |
| Philippines | 74,049,455.16  (61,720,886.44 to 86,644,749.34) | 94,947.95  (80,200.09 to 109,893.11) | 45,791,416.59  (38,519,998.44 to 53,371,596.98) | 39,668.37  (33,616.47 to 45,841.68) | -3.27  (-3.45 to -3.09) |
| Qatar | 77,039.68  (60,451.96 to 96,124.91) | 17,401.76  (13,614.36 to 21,846.49) | 454,281.10  (372,476.67 to 554,393.15) | 21,978.63  (18,056.10 to 26,673.03) | 0.40  (0.13 to 0.66) |
| Republic of Korea | 4,300,063.83  (3,391,274.65 to 5,192,095.37) | 9,987.87  (7,919.28 to 12,079.16) | 3,031,330.78  (2,520,983.92 to 3,598,451.63) | 7,652.50  (6,011.55 to 9,300.39) | -0.69  (-1.11 to -0.27) |
| Saudi Arabia | 6,931,569.77  (5,635,030.68 to 8,226,132.70) | 34,541.21  (29,089.46 to 39,949.35) | 11,273,823.38  (9,292,099.51 to 13,812,787.52) | 34,675.45  (28,262.94 to 42,410.46) | 0.73  (0.32 to 1.13) |
| Singapore | 51,732.83  (44,436.87 to 59,513.33) | 2,113.27  (1,791.46 to 2,464.73) | 52,610.94  (45,863.93 to 60,014.21) | 1,179.40  (990.22 to 1,415.53) | -1.47  (-1.84 to -1.10) |
| Sri Lanka | 9,950,085.47  (8,623,504.27 to 11,357,991.77) | 57,021.96  (50,197.51 to 64,219.13) | 10,760,086.02  (8,993,667.38 to 12,723,876.56) | 50,602.54  (41,557.61 to 60,481.70) | -0.59  (-0.71 to -0.47) |
| Syrian Arab Republic | 6,870,591.71  (5,641,252.48 to 8,124,334.59) | 35,406.16  (29,249.64 to 41,684.76) | 4,327,859.56  (3,360,634.51 to 5,477,615.88) | 30,463.69  (24,028.13 to 37,866.85) | -0.47  (-0.61 to -0.32) |
| Taiwan  (Province of China) | 3,639,910.24  (3,072,264.56 to 4,283,388.42) | 21,028.78  (17,964.28 to 24,387.34) | 11,389,002.98  (9,640,518.44 to 13,172,400.34) | 52,627.32  (43,761.84 to 62,390.73) | 4.30  (3.76 to 4.85) |
| Tajikistan | 3,888,474.14  (3,444,629.99 to 4,372,701.60) | 48,709.37  (43,365.58 to 54,743.18) | 2,656,741.76  (2,315,847.13 to 2,996,858.99) | 23,243.57  (20,331.07 to 26,209.82) | -2.73  (-2.93 to -2.54) |
| Thailand | 34,330,386.65  (29,329,257.71 to 39,757,665.91) | 61,856.85  (53,463.30 to 70,612.20) | 36,414,952.97  (31,957,269.31 to 41,622,740.70) | 51,862.32  (44,765.83 to 59,970.17) | -0.57  (-0.76 to -0.37) |
| Timor-Leste | 730,882.09  (648,236.96 to 820,751.74) | 69,077.53  (61,245.36 to 77,756.29) | 942,292.16  (811,703.08 to 1,073,609.06) | 61,702.40  (53,933.67 to 69,586.27) | -0.26  (-0.39 to -0.14) |
| Turkey | 24,900,341.41  (20,363,276.43 to 29,702,705.22) | 36,700.75  (30,230.18 to 43,615.66) | 20,241,034.76  (16,240,344.60 to 25,009,616.19) | 26,231.76  (20,827.58 to 32,629.40) | -0.86  (-1.14 to -0.59) |
| Turkmenistan | 1,790,875.31  (1,561,980.81 to 2,034,866.48) | 34,229.33  (29,991.27 to 38,488.32) | 449,243.19  (365,251.44 to 546,769.87) | 8,473.51  (6,895.26 to 10,310.17) | -5.15  (-5.50 to -4.80) |
| United Arab Emirates | 493,350.53  (385,437.54 to 603,703.93) | 26,712.51  (21,635.17 to 32,248.79) | 1,979,198.79  (1,672,595.90 to 2,333,337.22) | 26,514.11  (22,222.75 to 31,597.81) | 0.11  (-0.21 to 0.43) |
| Uzbekistan | 6,760,025.61  (5,749,966.76 to 7,718,871.60) | 22,434.96  (19,150.29 to 25,653.80) | 1,387,482.96  (1,102,313.75 to 1,693,374.77) | 3,973.04  (3,140.17 to 4,868.80) | -5.22  (-5.44 to -5.00) |
| Viet Nam | 44,928,738.00  (36,590,242.44 to 53,161,248.14) | 54,654.60  (45,545.14 to 63,647.27) | 51,785,878.18  (43,764,037.43 to 60,394,187.93) | 54,736.85  (46,016.23 to 64,166.81) | 0.52  (0.29 to 0.74) |
| Yemen | 13,063,903.68  (11,530,471.09 to 14,900,642.12) | 56,022.80  (49,697.52 to 63,800.42) | 13,400,566.75  (11,227,762.85 to 15,780,440.01) | 34,036.75  (29,163.14 to 39,431.54) | -0.25  (-0.91 to 0.40) |
| **Prevalence** |  |  |  |  |  |
| Afghanistan | 71,218.24  (60,746.93 to 84,044.66) | 461.69  (391.03 to 542.17 ) | 136,220.12  (111,116.88 to 168,558.92) | 316.14  (261.51 to 388.95) | -0.93  (-1.33 to -0.53) |
| Armenia | 18,377.03  (15,933.87 to 21,334.86) | 506.68  (438.62 to 588.22) | 2,993.66  (2,501.63 to 3,546.56) | 121.24  (98.38 to 146.61) | -4.57  (-5.39 to -3.75) |
| Azerbaijan | 31,815.09  (28,215.41 to 35,787.23) | 374.66  (331.89 to 421.50) | 19,238.04  (15,618.40 to 23,456.86) | 203.94  (164.79 to 251.49) | -2.29  (-2.46 to -2.12) |
| Bahrain | 1,624.16  (1,259.27 to 2,030.86) | 314.3  (249.07 to 387.11) | 4,266.10  (3,535.65 to 5,138.69) | 349.79  (291.58 to 422.99) | -0.07  (-0.48 to 0.34) |
| Bangladesh | 2,241,184.37  (2,018,728.15 to 2,482,589.61) | 1,592.36  (1,452.01 to 1,739.77) | 1,342,776.87  (1,220,349.24 to 1,485,115.60) | 804.59  (733.08 to 886.73) | -1.95  (-2.06 to -1.84) |
| Bhutan | 7,395.19  (6,458.20 to 8,467.49) | 1,106.35  (1,000.92 to 1,225.97) | 5,908.10  (5,360.36 to 6,526.16) | 808.48  (735.92 to 895.57) | -1.52  (-1.71 to -1.32) |
| Brunei Darussalam | 353.67  (282.48 to 435.03) | 119.06  (96.10 to 146.54) | 368.14  (297.69 to 448.20) | 87.92  (70.04 to 108.34) | -1.00  (-1.39 to -0.61) |
| Cambodia | 149,446.14  (135,442.44 to 165,959.14) | 1,035.81  (948.36 to 1,135.13) | 106,073.31  (93,633.11 to 120,656.21) | 624.7  (555.87 to 705.97) | -2.06  (-2.31 to -1.81) |
| China | 4,755,249.09  (4,024,241.88 to 5,653,271.94) | 416.83  (351.87 to 495.41) | 1,097,246.70  (932,724.83 to 1,293,535.05) | 86.96  (72.30 to 104.11) | -5.86  (-6.31 to -5.40) |
| Cyprus | 3,331.22  (2,737.86 to 4,151.58) | 460.76  (376.32 to 577.11) | 7,097.34  (6,099.86 to 8,259.42) | 586.42  (481.96 to 713.42) | 1.39  (0.91 to 1.87) |
| Democratic People's  Republic of Korea | 61,497.78  (52,610.66 to 72,053.50) | 279.3  (238.67 to 327.25) | 244,072.05  (198,713.52 to 289,832.56) | 1,029.92  (830.46 to 1,246.68) | 5.69  (5.03 to 6.36) |
| Georgia | 21,395.88  (18,201.13 to 25,165.62) | 435.00  (370.30 to 511.67) | 5,001.98  (4,268.00 to 5,773.32) | 167.22  (140.77 to 195.60) | -2.19  (-2.94 to -1.44) |
| India | 18,300,563.54  (15,825,684.21 to 20,985,595.08) | 2,020.84  (1,800.21 to 2,262.06) | 29,167,174.06  (26,514,236.97 to 31,996,718.62) | 2,089.00  (1,901.89 to 2,290.47) | -0.05  (-0.13 to 0.03) |
| Indonesia | 3,506,047.34  (3,039,427.68 to 3,992,754.07) | 1,832.84  (1,619.69 to 2,048.88) | 2,326,076.53  (2,119,404.09 to 2,560,863.12) | 926.08  (847.23 to 1,025.23) | -2.26  (-2.33 to -2.19) |
| Iran  (Islamic Republic of) | 634,868.40  (511,932.92 to 771,339.40) | 800.97  (661.88 to 957.24) | 303,130.71  (246,006.62 to 373,591.23) | 375.00  (301.22 to 467.12) | -2.43  (-2.68 to -2.17) |
| Iraq | 181,537.05  (154,769.95 to 210,895.32) | 667.92  (576.06 to 769.52) | 176,469.03  (141,797.53 to 217,807.54) | 396.72  (324.98 to 482.28) | -1.52  (-1.75 to -1.29) |
| Israel | 21,278.07  (17,024.43 to 26,890.93) | 415.25  (333.78 to 522.49) | 39,211.30  (34,472.60 to 44,847.83) | 372.7  (322.48 to 432.30) | 0.09  (-0.75 to 0.95) |
| Japan | 627,640.32  (513,726.06 to 750,114.89) | 601.87  (483.99 to 737.86) | 781,093.78  (685,489.64 to 886,217.88) | 812.97  (674.68 to 958.95) | 1.46  (1.12 to 1.80) |
| Jordan | 20,243.48  (16,691.44 to 24,388.20) | 384.56  (320.96 to 459.37) | 36,577.11  (28,837.50 to 45,801.38) | 293.87  (238.89 to 363.12) | -0.21  (-0.78 to 0.38) |
| Kazakhstan | 60,818.78  (53,531.08 to 68,481.24) | 338.71  (298.06 to 381.14) | 26,162.14  (20,525.22 to 32,278.37) | 139.15  (109.17 to 172.20) | -2.79  (-3.09 to -2.49) |
| Kuwait | 7,872.44  (6,170.38 to 9,671.02) | 419.68  (333.76 to 512.80) | 14,357.40  (11,596.48 to 17,528.47) | 367.52  (286.05 to 460.12) | 0.60  (0.35 to 0.84) |
| Kyrgyzstan | 21,168.41  (18,827.49 to 23,978.88) | 362.26  (322.60 to 409.59) | 8,705.75  (7,140.58 to 10,665.53) | 117.43  (97.24 to 143.25) | -2.96  (-3.19 to -2.73) |
| Lao People's  Democratic Republic | 63,030.30  (58,031.15 to 68,755.54) | 1,062.99  (979.52 to 1,153.79) | 55,949.14  (51,002.92 to 62,023.59) | 739.06  (677.07 to 815.32) | -1.10  (-1.22 to -0.98) |
| Lebanon | 14,068.42  (11,318.45 to 17,190.68) | 408.13  (331.65 to 495.08) | 29,478.57  (24,129.26 to 36,402.53) | 573.19  (461.64 to 717.66) | 0.78  (0.60 to 0.97) |
| Malaysia | 99,247.78  (83,600.71 to 119,250.97) | 502.21  (432.83 to 595.27) | 349,571.13  (294,878.31 to 415,597.65) | 1,157.65  (972.88 to 1,384.41) | 3.04  (2.89 to 3.19) |
| Maldives | 2,906.59  (2,577.00 to 3,244.18) | 871.64  (783.62 to 966.46) | 3,861.02  (3,315.26 to 4,513.23) | 870.6  (740.94 to 1,022.15) | -0.06  (-0.20 to 0.08) |
| Mongolia | 9,000.05  (7,541.13 to 10,743.20) | 282.39  (236.38 to 336.04) | 1,370.73  (1,192.16 to 1,568.76) | 37.87  (33.02 to 43.10) | -6.03  (-7.18 to -4.86) |
| Myanmar | 467,832.06  (428,654.87 to 513,652.22) | 995.77  (917.91 to 1,086.33) | 350,630.03  (312,615.54 to 394,200.34) | 639.24  (571.61 to 714.82) | -1.82  (-1.99 to -1.65) |
| Nepal | 319,660.96  (286,991.29 to 353,231.16) | 1,300.25  (1,181.17 to 1,426.72) | 203,135.19  (185,878.50 to 223,842.16) | 661.31  (608.86 to 726.12) | -1.68  (-1.95 to -1.42) |
| Oman | 9,655.37  (8,162.00 to 11,355.32) | 394.47  (341.12 to 454.86) | 14,269.26  (12,264.98 to 16,745.72) | 377.7  (331.11 to 437.38) | 0.22  (-0.27 to 0.72) |
| Pakistan | 2,645,555.91  (2,281,736.38 to 3,053,451.39) | 2,001.27  (1,764.17 to 2,251.50) | 3,062,286.07  (2,687,346.88 to 3,471,182.32) | 1,250.59  (1,118.96 to 1,398.45) | -1.47  (-1.56 to -1.37) |
| Palestine | 10,163.26  (8,129.14 to 12,429.37) | 337.92  (275.23 to 406.57) | 12,017.48  (9,399.28 to 15,468.92) | 211.73  (170.61 to 265.12) | -0.31  (-0.96 to 0.34) |
| Philippines | 1,232,593.15  (1,049,995.85 to 1,437,180.75) | 1,573.67  (1,358.4 to 1,808.53) | 696,738.52  (608,100.58 to 801,876.63) | 601.95  (528.71 to 690.21) | -3.54  (-3.72 to -3.37) |
| Qatar | 1,123.11  (885.85 to 1,392.09) | 255.57  (201.69 to 319.38) | 6,325.91  (5,223.07 to 7,674.22) | 318.17  (263.81 to 383.64) | 0.34  (0.07 to 0.61) |
| Republic of Korea | 67,789.34  (54,945.10 to 81,349.07) | 156.07  (127.54 to 186.66) | 47,111.05  (40,365.06 to 54,816.20) | 118.63  (96.40 to 145.37) | -0.70  (-1.12 to -0.27) |
| Saudi Arabia | 107,668.62  (91,404.93 to 124,693.07) | 541.00  (467.47 to 615.08) | 166,528.38  (138,252.94 to 202,190.07) | 516.57  (426.54 to 635.11) | 0.64  (0.19 to 1.09) |
| Singapore | 747.14  (655.24 to 851.21) | 30.80  (26.51 to 35.88) | 755.79  (662.06 to 866.88) | 17.28  (14.60 to 21.01) | -1.45  (-1.79 to -1.11) |
| Sri Lanka | 155,012.31  (138,006.80 to 174,694.73) | 890.57  (805.63 to 990.28) | 156,525.39  (132,263.94 to 183,290.23) | 736.81  (612.74 to 875.63) | -0.82  (-0.95 to -0.69) |
| Syrian Arab Republic | 106,908.62  (90,925.78 to 124,944.73) | 547.82  (468.99 to 631.71) | 64,463.59  (50,333.94 to 82,271.03) | 447.73  (356.67 to 561.24) | -0.60  (-0.77 to -0.44) |
| Taiwan  (Province of China) | 60,376.19  (52,022.10 to 70,703.94) | 353.02  (307.11 to 407.84) | 176,408.07  (151,743.12 to 203,790.80) | 805.94  (677.52 to 967.45) | 4.12  (3.51 to 4.73) |
| Tajikistan | 70,533.23  (64,406.32 to 77,346.61) | 883.12  (806.56 to 972.36) | 45,662.40  (41,088.23 to 51,408.31) | 403.83  (363.51 to 454.06) | -2.98  (-3.17 to -2.80) |
| Thailand | 550,532.10  (488,542.43 to 624,582.23) | 991.69  (891.91 to 111.50) | 555,885.68  (508,017.98 to 610,697.96) | 779.45  (696.27 to 866.84) | -0.71  (-0.93 to -0.50) |
| Timor-Leste | 12,722.15  (11,420.40 to 14,022.89) | 1,176.26  (1,058.67 to 1,291.48) | 14,207.87  (12,601.57 to 15,959.81) | 924.47  (833.55 to 1,025.07) | -0.66  (-0.78 to -0.53) |
| Turkey | 389,956.50  (325,521.17 to 461,909.93) | 572.97  (482.21 to 673.25) | 291,617.63  (234,583.93 to 360,130.67) | 378.97  (301.57 to 473.24) | -1.08  (-1.38 to -0.78) |
| Turkmenistan | 30,592.89  (27,941.70 to 33,965.28) | 585.23  (534.52 to 648.50) | 7,043.46  (5,772.42 to 8,556.83) | 132.79  (108.94 to 161.60) | -5.51  (-5.87 to -5.14) |
| United Arab Emirates | 7,416.90  (5,943.75 to 9,015.43) | 412.42  (341.18 to 488.97) | 28,724.84  (24,821.50 to 33,279.18) | 392.5  (333.58 to 464.44) | -0.01  (-0.34 to 0.32) |
| Uzbekistan | 107,519.26  (95,601.96 to 121,265.99) | 356.43  (318.09 to 403.81) | 19,952.89  (15,913.28 to 24,821.19) | 57.27  (45.58 to 71.68) | -5.52  (-5.76 to -5.29) |
| Viet Nam | 712,365.94  (597,521.01 to 836,322.01) | 859.15  (734.41 to 995.23) | 758,456.85  (652,731.85 to 880,008.33) | 803.82  (688.24 to 942.32) | 0.42  (0.15 to 0.68) |
| Yemen | 236,608.99  (213,344.43 to 260,887.84) | 997.23  (898.01 to 1,103.05) | 201,294.11  (173,139.26 to 236,502.47) | 506.85  (447.91 to 586.28) | -0.63  (-1.4 to 0.16) |
| **DALY** |  |  |  |  |  |
| Afghanistan | 670,124.07  (436,310.53 to 958,614.07) | 3,583.24  (2,344.53 to 5,082.64) | 374,617.88  (249,728.78 to 554,263.21) | 683.83  (464.51 to 996.29) | -5.63  (-6.29 to -4.97) |
| Armenia | 36,763.65  (31,463.35 to 42,725.86) | 998.34  (854.92 to 1,159.57) | 776.46  (623.73 to 971.49) | 36.54  (29.20 to 45.74) | -12.11  (-12.67 to -11.54) |
| Azerbaijan | 139,679.31  (108,507.11 to 178,619.15) | 1,597.95  (1,242.61 to 2,041.99) | 18,799.18  (12,566.55 to 28,585.51) | 265.86  (175.32 to 404.66) | -6.54  (-6.96 to -6.12) |
| Bahrain | 1,031.41  (809.88 to 1,305.38) | 192.62  (154.91 to 242.26) | 807.86  (618.02 to 1,053.39) | 78.41  (61.55 to 100.12) | -2.78  (-2.98 to -2.58) |
| Bangladesh | 5,815,932.95  (4,465,018.46 to 8,204,011.49) | 4,358.68  (3,234.17 to 5,888.90) | 1,007,943.16  (643,543.85 to 1,868,472.61) | 719.9  (451.53 to 1,346.44) | -5.81  (-6.05 to -5.57) |
| Bhutan | 62,577.19  (28,018.06 to 105,758.84) | 8,752.83  (3,958.52 to 14,415.25) | 5,924.51  (3,380.77 to 11,064.12) | 964.86  (546.94 to 1,812.01) | -7.53  (-7.80 to -7.26) |
| Brunei Darussalam | 105.45  (83.04 to 131.81) | 44.06  (34.53 to 57.69) | 118.81  (89.64 to 157.39) | 35.54  (26.14 to 46.51) | -0.24  (-0.40 to -0.07) |
| Cambodia | 577,147.30  (424,169.23 to 765,087.75) | 3,960.41  (2,913.76 to 5,428.95) | 60,097.59  (43,686.04 to 80,779.42) | 407.7  (284.42 to 563.41) | -8.03  (-8.39 to -7.67) |
| China | 7,677,368.19  (5,929,499.15 to 9,430,911.30) | 703.47  (542.66 to 865.60) | 276,747.33  (220,744.16 to 361,161.33) | 26.68  (21.77 to 33.08) | -11.42  (-11.93 to -10.91) |
| Cyprus | 968.68  (712.32 to 1,269.79) | 148.62  (109.59 to 202.56) | 1,425.40  (1,073.46 to 1,925.51) | 106.6  (80.81 to 140.04) | -0.62  (-0.83 to -0.41) |
| Democratic People's  Republic of Korea | 19,596.94  (14,489.38 to 26,197.82) | 85.93  (64.13 to 114.77) | 31,452.32  (21,887.83 to 43,771.75) | 136.02  (94.96 to 190.85) | 2.62  (2.17 to 3.06) |
| Georgia | 21,433.51  (18,315.93 to 25,046.12) | 484.45  (413.01 to 566.19) | 866.66  (669.31 to 1,128.84) | 29.92  (23.28 to 39.06) | -9.59  (-10.22 to -8.95) |
| India | 67,429,969.99  (54,291,459.42 to 84,144,238.02) | 8,374.29  (6,642.22 to 11,176.58) | 16,762,075.73  (12,036,350.55 to 24,176,897.72) | 1,444.03  (1,029.88 to 2,082.79) | -5.66  (-5.82 to -5.49) |
| Indonesia | 13,219,611.65  (9,668,196.40 to 17,191,993.93) | 7,454.64  (5,098.81 to 10,212.87) | 2,010,710.90  (1,445,146.65 to 2,535,011.96) | 941.70  (649.51 to 1,190.89) | -6.38  (-6.61 to -6.14) |
| Iran  (Islamic Republic of) | 407,167.32  (287,259.75 to 648,165.78) | 547.35  (387.89 to 861.58) | 53,367.33  (40,036.75 to 71,437.59) | 71.31  (54.66 to 94.72) | -6.13  (-6.28 to -5.98) |
| Iraq | 232,374.37  (166,472.32 to 321,615.53) | 771.05  (567.52 to 1,045.73) | 71,634.65  (53,452.89 to 92,122.00) | 177.82  (132.97 to 230.67) | -5.13  (-5.3 to -4.95) |
| Israel | 3,759.49  (2,901.61 to 4,813.73) | 74.92  (58.24 to 95.37) | 9,430.45  (7,955.08 to 11,324.22) | 83.12  (69.02 to 102.43) | 1.13  (0.34 to 1.91) |
| Japan | 91,627.03  (67,046.17 to 121,737.08) | 88.32  (64.13 to 118.97) | 132,237.03  (104,064.07 to 166,253.18) | 109.53  (77.96 to 150.15) | 1.17  (0.83 to 1.5) |
| Jordan | 11,521.24  (8,652.63 to 15,035.65) | 206.41  (157.04 to 263.18) | 8,392.31  (6,502.56 to 10,839.86) | 75.50  (59.18 to 98.30) | -3.05  (-3.25 to -2.86) |
| Kazakhstan | 159,721.66  (141,055.32 to 178,084.43) | 883.06  (780.19 to 983.60) | 6,312.46  (4,930.69 to 8,125.59) | 32.84  (25.6 to 42.22) | -12.43  (-13.19 to -11.66) |
| Kuwait | 1,966.91  (1,636.73 to 2,384.22) | 110.9  (93.73 to 133.38) | 1,949.48  (1,377.86 to 2,662.74) | 54.04  (38.96 to 73.89) | -0.59  (-1.06 to -0.11) |
| Kyrgyzstan | 73,124.27  (62,855.98 to 83,066.85) | 1,174.45  (1,012.04 to 1,330.86) | 6,401.92  (5,238.63 to 7,826.97) | 84.28  (69.21 to 102.76) | -9.35  (-9.97 to -8.72) |
| Lao People's  Democratic Republic | 530,894.99  (356,058.48 to 740,654.92) | 8,292.37  (5,819.08 to 11,597.33) | 53,056.08  (36,435.66 to 76,875.97) | 736.50  (511.27 to 1,047.94) | -7.92  (-8.16 to -7.68) |
| Lebanon | 12,022.30  (7,754.48 to 16,679.51) | 327.57  (219.55 to 442.84) | 7,323.60  (5,667.13 to 9,492.25) | 151.75  (114.59 to 199.07) | -2.5  (-2.73 to -2.26) |
| Malaysia | 63,150.19  (49,143.66 to 83,688.19) | 361.40  (276.83 to 511.37) | 68,506.63  (51,424.72 to 89,427.56) | 242.73  (180.86 to 313.27) | -0.96  (-1.35 to -0.57) |
| Maldives | 13,316.19  (9,415.77 to 16,783.48) | 3,836.02  (2,834.91 to 4,892.76) | 976.83  (772.59 to 1,259.74) | 264.33  (207.61 to 339.44) | -8.4  (-9.21 to -7.58) |
| Mongolia | 19,609.98  (12,031.84 to 30,936.73) | 579.49  (356.21 to 912.90) | 2,233.02  (998.64 to 4,597.83) | 59.74  (26.99 to 122.81) | -7.48  (-7.69 to -7.27) |
| Myanmar | 2,748,076.35  (1,452,029.81 to 4,310,312.14) | 5,987.47  (3,249.66 to 9,470.97) | 283,738.77  (212,868.46 to 371,846.84) | 561.91  (420.58 to 738.04) | -7.79  (-8.01 to -7.56) |
| Nepal | 1,512,440.14  (1,162,272.11 to 1,907,952.19) | 5,812.99  (4,542.82 to 7,221.34) | 162,427.18  (109,647.38 to 244,435.87) | 620.28  (414.42 to 951.14) | -7.21  (-7.37 to -7.05) |
| Oman | 8,648.09  (5,132.25 to 13,755.23) | 306.95  (196.09 to 457.29) | 3,567.15  (2,710.32 to 4,707.65) | 98.56  (75.08 to 129.31) | -2.96  (-3.73 to -2.18) |
| Pakistan | 8,527,073.70  (6,848,650.73 to 10,822,249.34) | 5,683.35  (4,255.53 to 7,629.8) | 2,465,288.39  (1,829,006.22 to 3,387,869.07) | 1,119.84  (813.89 to 1,605.51) | -4.81  (-5.03 to -4.58) |
| Palestine | 15,250.06  (10,472.20 to 22,715.56) | 412.64  (296.57 to 595.25) | 3,066.86  (2,271.44 to 4,017.54) | 56.38  (42.71 to 73.28) | -5.35  (-5.74 to -4.96) |
| Philippines | 1,792,740.24  (1,331,584.90 to 2,280,281.82) | 2,210.83  (1,660.49 to 2,938.87) | 422,173.88  (332,514.00 to 531,798.61) | 407.88  (318.75 to 521.29) | -5.18  (-5.33 to -5.03) |
| Qatar | 434.07  (321.94 to 564.03) | 99.11  (75.38 to 126.44) | 1,056.42  (805.15 to 1,421.98) | 58.13  (44.81 to 77.21) | -1.83  (-1.99 to -1.66) |
| Republic of Korea | 28,734.35  (20,656.63 to 35,916.03) | 92.95  (62.96 to 120.88) | 23,419.34  (14,443.91 to 39,549.98) | 41.45  (28.97 to 60.16) | -2.08  (-2.3 to -1.86) |
| Saudi Arabia | 130,656.12  (90,546.67 to 193,042.33) | 620.42  (443.73 to 874.27) | 34,554.07  (26,044.07 to 45,547.39) | 129.16  (96.59 to 173.09) | -4.94  (-5.34 to -4.54) |
| Singapore | 1,018.79  (951.24 to 1,088.56) | 45.84  (42.65 to 49.07) | 1,019.26  (909.92 to 1,121.97) | 16.61  (14.81 to 18.32) | -2.14  (-2.57 to -1.71) |
| Sri Lanka | 163,206.35  (121,486.83 to 217,794.64) | 1,140.55  (809.44 to 1,559.12) | 38,157.13  (27,539.73 to 53,874.30) | 179.06  (132.52 to 247.27) | -5.59  (-6.12 to -5.06) |
| Syrian Arab Republic | 81,436.94  (58,604.34 to 114,610.90) | 401.86  (294.61 to 558.01) | 11,268.39  (7,933.96 to 15,096.19) | 88.29  (64.04 to 117.69) | -4.45  (-5.05 to -3.85) |
| Taiwan  (Province of China) | 16,579.59  (14,402.48 to 19,558.76) | 99.06  (86.54 to 116.30) | 21,424.38  (15,671.25 to 28,962.93) | 99.44  (70.54 to 140.72) | 1.23  (0.47 to 1.99) |
| Tajikistan | 314,748.78  (255,629.71 to 385,250.70) | 3,293.15  (2,672.57 to 4,028.79) | 126,780.59  (87,561.31 to 174,932.66) | 953.83  (661.16 to 1,323.04) | -4.76  (-5.11 to -4.4) |
| Thailand | 564,810.65  (245,268.48 to 1,179,029.93) | 1,270.77  (512.50 to 2,863.69) | 201,985.33  (137,083.89 to 287,181.81) | 284.50  (212.24 to 376.33) | -4.49  (-4.89 to -4.08) |
| Timor-Leste | 89,291.11  (52,357.68 to 121,524.45) | 7,015.47  (4,492.08 to 10,286.65) | 11,053.60  (7,603.31 to 15,812.82) | 721.66  (498.55 to 1,011.04) | -7.68  (-8.05 to -7.31) |
| Turkey | 531,300.66  (376,555.89 to 796,769.74) | 776.68  (551.89 to 1,155.53) | 64,454.05  (50,647.55 to 81,063.18) | 97.20  (75.68 to 122.85) | -6.71  (-6.93 to -6.5) |
| Turkmenistan | 146,158.04  (126,789.30 to 169,364.50) | 2,499.74  (2,171.97 to 2,890.05) | 5,125.25  (4,043.43 to 6,501.00) | 96.43  (76.21 to 122.16) | -12.11  (-12.81 to -11.41) |
| United Arab Emirates | 3,270.62  (2,364.87 to 4,296.57) | 177.17  (134.43 to 226.79) | 5,151.57  (3,987.31 to 6,699.30) | 91.96  (71.60 to 116.66) | -1.05  (-1.41 to -0.68) |
| Uzbekistan | 380,985.77  (336,221.05 to 429,830.37) | 1,145.12  (1,012.98 to 1,290.89) | 11,023.72  (8,971.11 to 13,780.52) | 29.86  (24.43 to 37.07) | -12.85  (-13.58 to -12.12) |
| Viet Nam | 472,239.72  (285,583.45 to 679,485.13) | 645.88  (385.34 to 923.14) | 121,397.04  (90,309.04 to 164,836.79) | 132.22  (98.03 to 179.30) | -4.75  (-5.21 to -4.29) |
| Yemen | 1,327,613.77  (891,505.36 to 1,894,796.28) | 4,816.06  (3,234.95 to 6,658.74) | 154,491.64  (74,261.93 to 275,165.79) | 377.41  (189.71 to 633.94) | -7.85  (-8.21 to -7.49) |
| **Mortality** |  |  |  |  |  |
| Afghanistan | 7,558.90  (4,902.26 to 10,769.45) | 41.85  (27.34 to 58.51) | 4,075.43  (2,667.69 to 6,072.12) | 7.79  (5.20 to 11.10) | -5.74  (-6.40 to -5.07) |
| Armenia | 399.79  (341.11 to 463.35) | 10.99  (9.41 to 12.71) | 6.97  (5.70 to 8.56) | 0.30  (0.24 to 0.38) | -13.45  (-14.26 to -12.63) |
| Azerbaijan | 1,544.47  (1,188.25 to 1,984.25) | 17.85  (13.74 to 22.90) | 202.83  (130.82 to 313.90) | 2.90  (1.86 to 4.50) | -6.63  (-7.07 to -6.20) |
| Bahrain | 10.74  (8.24 to 14.20) | 3.34  (2.27 to 4.52) | 7.11  (4.39 to 11.44) | 1.39  (0.79 to 2.17) | -2.76  (-2.9 to -2.62) |
| Bangladesh | 90,330.63  (65,995.86 to 122,837.80) | 122.58  (78.51 to 165.34) | 30,483.13  (15,861.86 to 66,979.08) | 26.77  (13.57 to 58.00) | -5.09  (-5.23 to -4.96) |
| Bhutan | 905.22  (400.29 to 1,501.01) | 238.71  (114.03 to 392.14) | 179.04  (77.22 to 381.96) | 33.21  (14.28 to 70.98) | -6.72  (-7.04 to -6.41) |
| Brunei Darussalam | 1.27  (0.90 to 1.86)) | 1.14  (0.70 to 1.85) | 2.12  (1.20 to 3.15) | 0.97  (0.50 to 1.48) | 0.30  (0.02 to 0.59) |
| Cambodia | 7,764.83  (5,652.61 to 10,580.12) | 93.34  (57.85 to 143.09) | 1,170.40  (680.64 to 1,682.83) | 11.59  (6.16 to 17.04) | -7.21  (-7.49 to -6.93) |
| China | 91,081.07  (67,515.15 to 112,726.82) | 9.32  (6.71 to 11.79) | 4,307.16  (2,722.84 to 7,397.87) | 0.34  (0.24 to 0.52) | -11.44  (-11.89 to -10.97) |
| Cyprus | 20.82  (12.02 to 38.13) | 4.42  (2.26 to 8.84) | 41.43  (21.61 to 61.92) | 2.55  (1.32 to 4.01) | -1.72  (-1.95 to -1.49) |
| Democratic People's  Republic of Korea | 174.39  (112.68 to 257.56) | 0.89  (0.57 to 1.30) | 93.86  (53.85 to 152.10) | 0.39  (0.23 to 0.61) | -2.44  (-2.55 to -2.33) |
| Georgia | 231.76  (197.12 to 270.05) | 5.20  (4.40 to 6.10) | 6.20  (5.33 to 7.24) | 0.17  (0.14 to 0.21) | -12.44  (-13.28 to -11.6) |
| India | 1,251,899.79  (979,085.13 to 1,721,782.46) | 262.47  (194.07 to 375.34) | 496,724.97  (303,569.73 to 791,339.64) | 51.95  (31.86 to 82.57) | -5.19  (-5.35 to -5.03) |
| Indonesia | 216,770.46  (145,799.02 to 313,369.98) | 193.03  (109.35 to 299.06) | 51,016.99  (26,865.31 to 70,115.05) | 31.71  (15.10 to 46.40) | -5.49  (-5.76 to -5.22) |
| Iran  (Islamic Republic of) | 4,052.16  (2,655.41 to 6,792.19) | 6.98  (4.46 to 11.3) | 583.42  (327.47 to 1,013.33) | 0.91  (0.53 to 1.58) | -6.13  (-6.29 to -5.97) |
| Iraq | 2,529.43  (1,803.13 to 3,476.79) | 9.94  (7.12 to 13.35) | 720.74  (517.07 to 1,008.47) | 2.39  (1.71 to 3.71) | -5.13  (-5.33 to -4.93) |
| Israel | 39.48  (35.76 to 43.00) | 0.93  (0.84 to 1.02) | 329.33  (266.76 to 376.37) | 2.32  (1.90 to 2.65) | 4.07  (3.12 to 5.02) |
| Japan | 971.68  (855.46 to 1,034.44) | 0.77  (0.67 to 0.82) | 3,580.90  (2,786.40 to 4,050.63) | 0.69  (0.58 to 0.76) | 0.26  (-0.10 to 0.63) |
| Jordan | 110.38  (79.23 to 149.21) | 2.65  (1.90 to 3.62) | 65.91  (44.90 to 94.38) | 0.88  (0.57 to 1.31) | -3.84  (-4.00 to -3.69) |
| Kazakhstan | 1,774.59  (1,566.70 to 1,975.54) | 9.97  (8.83 to 11.08) | 47.17  (37.88 to 57.97) | 0.25  (0.21 to 0.31) | -13.74  (-14.57 to -12.90) |
| Kuwait | 13.30  (11.58 to 15.24) | 1.04  (0.93 to 1.16) | 6.60  (5.54 to 7.77) | 0.28  (0.23 to 0.33) | -2.06  (-3.07 to -1.03) |
| Kyrgyzstan | 814.57  (703.61 to 925.68) | 13.46  (11.71 to 15.23) | 63.98  (51.24 to 78.86) | 0.87  (0.70 to 1.06) | -9.84  (-10.5 to -9.17) |
| Lao People's  Democratic Republic | 6,768.52  (4,669.68 to 9,456.41) | 158.41  (104.82 to 257.19) | 806.09  (539.31 to 1,200.97) | 16.14  (10.49 to 26.13) | -7.54  (-7.74 to -7.33) |
| Lebanon | 149.13  (97.70 to 208.24) | 5.25  (3.54 to 7.41) | 124.08  (77.74 to 186.72) | 2.13  (1.40 to 3.07) | -2.79  (-3.00 to -2.58) |
| Malaysia | 1,047.73  (750.72 to 1,664.16) | 9.31  (6.15 to 15.97) | 1,095.06  (547.89 to 1,555.34) | 4.77  (2.35 to 6.85) | -2.13  (-2.32 to -1.93) |
| Maldives | 163.47  (118.22 to 209.78) | 74.13  (50.61 to 104.60) | 12.85  (8.28 to 18.58) | 4.43  (2.78 to 6.42) | -8.87  (-9.63 to -8.1) |
| Mongolia | 209.72  (124.33 to 336.50) | 6.26  (3.74 to 10.00) | 23.67  (9.69 to 49.90) | 0.64  (0.27 to 1.34) | -7.58  (-7.75 to -7.41) |
| Myanmar | 37,444.90  (20,251.86 to 59,847.25) | 111.95  (60.27 to 189.61) | 5,278.60  (3,421.22 to 7,340.15) | 12.42  (7.74 to 17.52) | -7.28  (-7.52 to -7.04) |
| Nepal | 21,463.09  (16,698.26 to 26,812.14) | 144.70  (103.90 to 210.71) | 4,141.71  (2,383.34 to 7,520.76) | 21.46  (11.79 to 40.58) | -6.34  (-6.47 to -6.22) |
| Oman | 95.00  (54.46 to 153.21) | 5.02  (3.04 to 7.53) | 31.18  (20.14 to 46.14) | 1.44  (0.86 to 2.14) | -3.41  (-4.10 to -2.72) |
| Pakistan | 122,604.87  (90,003.24 to 167,722.92) | 133.59  (83.47 to 219.28) | 40,203.57  (26,628.69 to 61,473.87) | 31.46  (18.58 to 54.61) | -4.56  (-4.74 to -4.38) |
| Palestine | 169.05  (113.82 to 255.73) | 5.93  (4.12 to 8.43) | 24.39  (16.86 to 34.48) | 0.70  (0.44 to 0.98) | -6.50  (-6.81 to -6.19) |
| Philippines | 21,958.30  (16,159.31 to 30,070.74) | 39.87  (27.58 to 67.06) | 6,812.00  (4,714.27 to 10,063.82) | 8.67  (5.76 to 13.85) | -4.57  (-4.72 to -4.41) |
| Qatar | 3.85  (2.66 to 5.45) | 1.65  (1.11 to 2.38) | 5.70  (3.61 to 8.44) | 0.79  (0.46 to 1.26) | -2.56  (-2.78 to -2.33) |
| Republic of Korea | 616.34  (312.60 to 886.91) | 3.29  (1.50 to 5.08) | 1,184.26  (570.66 to 2,221.34) | 1.42  (0.72 to 2.60) | -2.13  (-2.62 to -1.63) |
| Saudi Arabia | 1,507.13  (1,019.87 to 2,200.37) | 10.78  (7.30 to 16.22) | 353.34  (209.35 to 579.06) | 2.70  (1.47 to 4.53) | -4.30  (-4.59 to -4.01) |
| Singapore | 28.46  (26.26 to 30.25) | 1.59  (1.45 to 1.71) | 55.27  (46.67 to 62.04) | 0.71  (0.61 to 0.80) | -1.48  (-1.90 to -1.06) |
| Sri Lanka | 3,387.34  (2,019.13 to 5,013.75) | 35.45  (20.49 to 53.58) | 854.15  (404.16 to 1,488.78) | 3.89  (1.88 to 6.62) | -6.63  (-7.17 to -6.08) |
| Syrian Arab Republic | 828.65  (560.89 to 1,208.53) | 5.11  (3.50 to 7.47) | 90.91  (57.29 to 146.24) | 1.01  (0.65 to 1.62) | -4.79  (-5.38 to -4.20) |
| Taiwan  (Province of China) | 199.17  (187.42 to 210.11) | 1.45  (1.35 to 1.54) | 99.44  (86.24 to 112.17) | 0.27  (0.24 to 0.30) | -4.58  (-5.42 to -3.73) |
| Tajikistan | 3,478.14  (2,819.31 to 4,269.99) | 37.08  (30.21 to 45.41) | 1,396.48  (956.54 to 1,962.56) | 10.69  (7.45 to 14.98) | -4.79  (-5.15 to -4.42) |
| Thailand | 12,367.47  (3,290.19 to 33,834.39) | 39.28  (11.78 to 112.60) | 6,716.65  (3,303.48 to 11,398.73) | 6.96  (3.76 to 11.32) | -5.37  (-5.83 to -4.90) |
| Timor-Leste | 1,088.90  (661.16 to 1,541.45) | 134.75  (80.72 to 239.24) | 164.32  (105.59 to 254.41) | 16.18  (9.97 to 27.58) | -7.19  (-7.56 to -6.82) |
| Turkey | 5,806.06  (4,111.78 to 8,746.25) | 9.28  (6.64 to 13.39) | 755.42  (486.91 to 1,024.47) | 1.12  (0.76 to 1.49) | -6.93  (-7.29 to -6.56) |
| Turkmenistan | 1,616.31  (1,402.72 to 1,878.02) | 28.13  (24.55 to 32.52) | 53.06  (40.96 to 68.94) | 1.02  (0.79 to 1.32) | -12.38  (-13.11 to -11.64) |
| United Arab Emirates | 31.20  (21.48 to 41.99) | 3.01  (1.92 to 4.22) | 34.62  (20.85 to 50.94) | 1.52  (0.85 to 2.22) | -0.49  (-0.99 to 0.02) |
| Uzbekistan | 4,195.80  (3,695.04 to 4,744.40) | 12.90  (11.42 to 14.52) | 112.31  (88.53 to 140.33) | 0.32  (0.25 to 0.39) | -13.16  (-13.92 to -12.4) |
| Viet Nam | 7,759.46  (3,889.68 to 12,807.38) | 16.48  (7.19 to 31.41) | 1,418.84  (615.69 to 2,473.99) | 1.81  (0.77 to 3.22) | -7.05  (-7.31 to -6.78) |
| Yemen | 15,029.79  (9,951.05 to 21,266.21) | 67.36  (43.26 to 94.46) | 1,717.77  (714.12 to 3,081.91) | 6.15  (2.95 to 10.69) | -7.73  (-8.13 to -7.33) |

DALY, disability-adjusted life years; EAPC, estimated annual percentage change; 95% UI, 95% uncertainty interval; 95% CI, 95% confidence interval.
