# Supplementary material for: The burden and etiologies of diarrhea in Asia and its countries from 1990 to 2021 and the forecast to 2040: analyses informed by the global burden of disease study 2021
Source: Front Public Health. 2025 Aug 6;13:1651315. doi: 10.3389/fpubh.2025.1651315 (PMC12364947; doi:10.3389/fpubh.2025.1651315)
Supplement: Supplementary file 3 [file Table_2.DOCX]

**Table S2** Temporal joinpoint analysis of diarrheal diseases in Asia and Asian countries.

|  | **APC (Segment1)** | | **APC (Segment2)** | | **APC (Segment3)** | | **APC (Segment4)** | | **APC (Segment5)** | | **APC (Segment6)** | | **AAPC**  **(95% CI)** |
| --- | --- | --- | --- | --- | --- | --- | --- | --- | --- | --- | --- | --- | --- |
|  | **Year** | **APC (95% CI)** | **Year** | **APC (95% CI)** | **Year** | **APC (95% CI)** | **Year** | **APC (95% CI)** | **Year** | **APC (95% CI)** | **Year** | **APC (95% CI)** |  |
| **Asia** |  | | | | | | | | | | | | |
| Incidence | 1990-1999 | -0.81^*^  (-0.87 to -0.75) | 1999-2006 | 0.13^*^  (0.02 to 0.23) | 2006-2011 | -0.33^*^  (-0.53 to -0.13) | 2011-2015 | -1.30 ^*^  (-1.60 to -0.99) | 2015-2019 | -0.29  (-0.59 to 0.02) | 2019-2021 | 1.39 ^*^  (0.80 to 1.99) | -0.38^*^  (-0.45 to -0.30) |
| Prevalence | 1990-1994 | -1.25 ^*^  (-1.39 to -1.12) | 1994-2000 | -0.82 ^*^  (-0.91 to -0.73 | 2000-2010 | -0.17^*^  (-0.20 to -0.14 | 2010-2015 | -1.38 ^*^  (-1.50 to -1.25) | 2015-2019 | -0.03  (-0.22 to 0.15) | 2019-2021 | 1.53 ^*^  (1.10 to 1.89) | -0.51^*^  (-0.55 to -0.46) |
| DALY | 1990-1997 | -4.11 ^*^  (-4.2 to -3.92) | 1997-2002 | -6.02 ^*^  (-6.48 to -5.56) | 2002-2008 | -5.04 ^*^  (-5.37 to -4.70) | 2008-2013 | -6.20 ^*^  (-6.68 to -5.72) | 2013-2016 | -7.73 ^*^  (-9.28 to -6.16) | 2016-2021 | -6.18 ^*^  (-6.56 to -5.80) | -5.62 ^*^  (-5.82 to -5.43) |
| Mortality | 1990-1995 | -3.58^*^  (-3.94 to -3.23) | 1995-2001 | -5.91^*^  (-6.27 to -5.54) | 2001-2007 | -4.37 ^*^  (-4.77 to -3.97) | 2007-2013 | -5.81 ^*^  (-6.23 to -5.40) | 2013-2016 | -7.65^*^  (-9.53 to -5.73) | 2016-2021 | -6.12 ^*^  (-6.55 to -5.70) | -5.43^*^  (-5.65 to -5.20) |
| **Afghanistan** |  | | | | | | | | | | | | |
| Incidence | 1990-1995 | 1.44 ^*^  (1.06 to 1.81) | 1995-2000 | -0.48  (-0.99 to 0.03) | 2000-2005 | 2.60^*^  (2.06 to 3.15) | 2005-2014 | -1.55^*^  (-1.74 to -1.36) | 2014-2021 | -4.93^*^  (-5.16 to -4.70) | ——————————— | | -1.02^*^  (-1.17 to -0.88) |
| Prevalence | 1990-1995 | 1.26^*^  (0.89 to 1.63) | 1995-2000 | -0.80^*^  (-1.30 to -0.29) | 2000-2005 | 2.61^*^  (2.07 to 3.15) | 2005-2014 | -1.85^*^  (-2.05 to -1.64) | 2014-2021 | -5.27^*^  (-5.53 to -5.02) | ——————————— | | -1.27^*^  (-1.41 to -1.12) |
| DALY | 1990-1998 | 0.45  (-0.35 to 1.25) | 1998-2005 | -3.12^*^  (-4.15 to -2.09) | 2005-2011 | -11.17^*^  (-12.46 to -9.87) | 2011-2016 | -4.91^*^  (-7.13 to -2.63) | 2016-2021 | -10.01^*^  (-11.63 to -8.36) | ——————————— | | -5.26^*^  (-5.83 to -4.70) |
| Mortality | 1990-1999 | 0.13  (-0.58 to 0.84) | 1999-2005 | -3.62^*^  (-5.12 to -2.08) | 2005-2011 | -11.22^*^  (-12.60 to -9.83) | 2011-2016 | -5.16^*^  (-7.52 to -2.74) | 2016-2021 | -9.89^*^  (-11.59 to -8.17) | ——————————— | | -5.36^*^  (-5.98 to -4.75) |
| **Armenia** |  |  |  |  |  |  |  |  |  |  |  | |  |
| Incidence | 1990-2000 | -0.31^*^  (-0.57 to -0.05) | 2000-2011 | -1.54^*^  (-1.83 to -1.25) | 2011-2015 | -15.37  (-17.15 to 13.56) | 2015-2018 | -9.31^*^  (-13.16 to -5.29) | 2018-2021 | -3.81^*^  (-5.88 to -1.69) | ——————————— | | -4.04^*^  (-4.54 to -3.54) |
| Prevalence | 1990-2001 | -0.79^*^  (-0.90 to -0.69) | 2001-2005 | -3.50^*^  (-4.32 to -2.68) | 2005-2011 | -0.81^*^  (-1.24 to -0.37) | 2011-2015 | -17.16^*^  (-18.03 to -16.29) | 2015-2018 | -9.33^*^  (-11.28 to -7.33) | 2018-2021 | -3.41^*^  (-4.45 to -2.35) | -4.51^*^  (-4.78 to -4.24) |
| DALY | 1990-1994 | 0.84  (-1.77 to 3.51) | 1994-1997 | -11.00^*^  (-17.07 to -4.49) | 1997-2003 | -14.38^*^  (-16.04 to -12.69) | 2003-2006 | -10.00  (-19.54 to 0.67) | 2006-2014 | -16.28^*^  (-17.66 to -14.87) | 2014-2021 | -4.69^*^  (-6.22 to -3.13) | -10.16^*^  (-11.38 to -8.92) |
| Mortality | 1990-1994 | 2.62  (-1.47 to 6.88) | 1994-2006 | -14.14^*^  (-14.96 to -13.32) | 2006-2013 | -20.44^*^  (-22.86 to -17.94) | 2013-2021 | -2.56^*^  (-4.41 to -0.68) | ——————————— | | ——————————— | | -10.77^*^  (-11.67 to -9.87) |
| **Azerbaijan** |  |  |  |  |  |  |  |  |  |  |  |  |  |
| Incidence | 1990-1994 | 1.26^*^  (0.92 to 1.61) | 1994-2006 | -0.88^*^  (-0.96 to -0.81) | 2006-2009 | -4.69^*^  (-5.92 to -3.44) | 2009-2021 | -2.86^*^  (-2.94 to -2.77) | ——————————— | | ——————————— | | -1.75^*^  (-1.88 to -1.62) |
| Prevalence | 1990-1993 | 1.00^*^  (0.71 to 1.28) | 1993-1996 | -0.56^*^  (-1.11 to -0.01) | 1996-2000 | -2.18^*^  (-2.46 to -1.90) | 2000-2005 | -1.34^*^  (-1.55 to -1.14) | 2005-2010 | -4.16^*^  (-4.39 to -3.93) | 2010-2021 | -2.38^*^  (-2.44 to -2.31) | -1.98^*^  (-2.06 to -1.90) |
| DALY | 1990-1996 | -2.62^*^  (-3.09 to -2.16) | 1996-1999 | -4.91^*^  (-7.29 to -2.47) | 1999-2002 | -11.14^*^  (-13.53 to -8.69) | 2002-2008 | -9.74^*^  (-10.38 to -9.09) | 2008-2012 | -5.61^*^  (-7.38 to -3.81) | 2012-2021 | -2.87^*^  (-3.26 to -2.48) | -5.56^*^  (-5.97 to -5.14) |
| Mortality | 1990-1996 | -2.62^*^  (-3.18 to -2.06) | 1996-1999 | -5.22^*^  (-8.05 to -2.30) | 1999-2007 | -10.60^*^  (-11.03 to -10.17) | 2007-2012 | -6.21^*^  (-7.59 to -4.81) | 2012-2021 | -2.79^*^  (-3.27 to -2.31) | ——————————— | | -5.62^*^  (-6.00 to -5.23) |
| **Bahrain** |  |  |  |  |  |  |  |  |  |  |  |  |  |
| Incidence | 1990-2000 | 2.10^*^  (2.01 to 2.19) | 2000-2004 | 3.08^*^  (2.54 to 3.62) | 2004-2007 | -0.70  (-1.74 to 0.35) | 2007-2015 | -3.32^*^  (-3.45 to -3.19) | 2015-2021 | 1.90^*^  (1.73 to 2.06) | ——————————— | | 0.49^*^  (0.36 to 0.61) |
| Prevalence | 1990-2000 | 2.19^*^  (2.09 to 2.30) | 2000-2003 | 3.38^*^  (2.13 to 4.65) | 2003-2006 | 1.00  (-0.26 to 2.29) | 2006-2015 | -3.70^*^  (-3.82 to -3.58) | 2015-2021 | 1.87^*^  (1.68 to 2.06) | ——————————— | | 0.38^*^  (0.21 to 0.55) |
| DALY | 1990-1998 | -4.92^*^  (-5.27 to -4.57) | 1998-2005 | 0.19  (-0.36 to 0.74) | 2005-2013 | -4.80^*^  (-5.24 to -4.36) | 2013-2021 | -1.62^*^  (-2.03 to -1.22) | ——————————— | | ——————————— | | -2.91^*^  (-3.11 to -2.70) |
| Mortality | 1990-1997 | -4.51^*^  (-4.93 to -4.09) | 1997-2005 | -1.33^*^  (-1.79 to -0.88) | 2005-2015 | -3.75^*^  (-4.08 to -3.41) | 2015-2021 | -1.02^*^  (-1.76 to -0.28) | ——————————— | | ——————————— | | -2.78^*^  (-3.00 to -2.56) |
| **Bangladesh** |  |  |  |  |  |  |  |  |  |  |  |  |  |
| Incidence | 1990-1997 | -2.68^*^  (-2.74 to -2.62) | 1997-2005 | -2.05^*^  (-2.10 to -1.99) | 2005-2010 | -0.52^*^  (-0.67 to -0.37) | 2010-2014 | -1.68^*^  (-1.92 to -1.44) | 2014-2019 | -1.27^*^  (-1.42 to -1.12) | 2019-2021 | -3.82^*^  (-4.29 to -3.35) | -1.89^*^  (-1.94 to -1.83) |
| Prevalence | 1990-1995 | -3.31^*^  (-3.38 to -3.24) | 1995-2005 | -2.47^*^  (-2.49 to -2.44) | 2005-2010 | -0.73^*^  (-0.83 to -0.62) | 2010-2015 | -1.92^*^  (-2.02 to -1.81) | 2015-2019 | -1.26^*^  (-1.43 to -1.10) | 2019-2021 | -3.94^*^  (-4.28 to -3.59) | -2.18^*^  (-2.22 to -2.14) |
| DALY | 1990-1992 | -5.40^*^  (-7.70 to -3.05) | 1992-2000 | -7.47^*^  (-7.76 to -7.17) | 2000-2013 | -5.87^*^  (-6.07 to -5.67) | 2013-2021 | -3.34^*^  (-3.85 to -2.83) | ——————————— | | ——————————— | | -5.61^*^  (-5.83 to -5.40) |
| Mortality | 1990-2008 | -5.07^*^  (-5.14 to -5.00) | 2008-2013 | -6.88^*^  (-7.91 to -5.85) | 2013-2021 | -2.95^*^  (-3.37 to -2.52) | ——————————— | | ——————————— | | ——————————— | | -4.82^*^  (-5.02 to -4.63) |
| **Bhutan** |  |  |  |  |  |  |  |  |  |  |  |  |  |
| Incidence | 1990-2006 | -0.29^*^  (-0.32 to -0.25) | 2006-2015 | -3.04^*^  (-3.14 to -2.95) | 2015-2019 | 0.40  (-0.04 to 0.83) | 2019-2021 | 2.73^*^  (1.77 to 3.69) | ——————————— | | ——————————— | | -0.82^*^  (-0.90 to -0.73) |
| Prevalence | 1990-2006 | -0.55^*^  (-0.59 to -0.50) | 2006-2015 | -3.51^*^  (-3.63 to -3.40) | 2015-2019 | 0.33  (-0.19 to 0.85) | 2019-2021 | 3.17^*^  (2.02 to 4.33) | ——————————— | | ——————————— | | -1.07^*^  (-1.17 to -0.97) |
| DALY | 1990-1994 | -4.54^*^  (-6.86 to -2.16) | 1994-2012 | -8.61^*^  (-8.89 to -8.33) | 2012-2021 | -4.49^*^  (-5.30 to -3.69) | ——————————— | | ——————————— | | ——————————— | | -6.91^*^  (-7.30 to -6.52) |
| Mortality | 1990-1005 | -5.38^*^  (-5.84 to -4.92) | 1995-1999 | -9.59^*^  (-10.68 to -8.50) | 1999-2012 | -7.49^*^  (-7.66 to -7.31) | 2012-2021 | -3.22^*^  (-3.51 to -2.93) | ——————————— | | ——————————— | | -6.20^*^  (-6.39 to -6.02) |
| **Brunei Darussalam** |  |  |  |  |  |  |  |  |  |  |  |  |  |
| Incidence | 1990-1993 | 1.71^*^  (1.29 to 2.12) | 1993-2005 | 0.68^*^  (0.62 to 0.73) | 2005-2008 | -0.35  (-1.17 to 0.48) | 2008-2011 | -1.37^*^  (-2.21 to -0.53) | 2011-2018 | -4.65^*^  (-4.78 to -4.52) | 2018-2021 | -1.30^*^  (-1.72 to -0.89) | -0.94^*^  (-1.06 to -0.82) |
| Prevalence | 1990-1994 | 1.48^*^  (1.23 to 1.72) | 1994-2005 | 0.63^*^  (0.56 to 0.69) | 2005-2008 | -0.34  (-1.11 to 0.43) | 2008-2011 | -1.44^*^  (-2.22 to -0.65) | 2011-2018 | -4.76^*^  (-4.89 to -4.64) | 2018-2021 | -1.25^*^  (-1.63 to -0.87) | -0.98^*^  (-1.09 to -0.87) |
| DALY | 1990-1999 | -0.85^*^  (-0.95 to -0.75) | 1999-2004 | 0.89^*^  (0.54 to 1.24) | 2004-2011 | 0.41^*^  (0.23 to 0.59) | 2011-2016 | -0.76^*^  (-1.09 to -0.43) | 2016-2019 | -2.62^*^  (-3.78 to -1.44) | 2019-2021 | -4.94^*^  (-6.10 to -3.75) | -0.72^*^  (-0.87 to -0.56) |
| Mortality | 1990-1997 | -1.81^*^  (-2.42 to -1.20) | 1997-2006 | -0.17  (-0.65 to 0.32) | 2006-2017 | 2.38^*^  (2.05 to 2.71) | 2017-2021 | -7.01^*^  (-8.31 to -5.68) | ——————————— | | ——————————— | | -0.56^*^  (-0.84 to -0.29) |
| **Cambodia** |  |  |  |  |  |  |  |  |  |  |  |  |  |
| Incidence | 1990-1995 | 0.28^*^  (0.20 to 0.36) | 1995-2006 | -0.59^*^  (-0.62 to -0.56) | 2006-2010 | -1.93^*^  (-2.13 to -1.74) | 2010-2015 | -4.58^*^  (-4.71 to -4.45) | 2015-2019 | -1.38^*^  (-1.61 to -1.15) | 2019-2021 | 2.29^*^  (1.82 to 2.76) | -1.20^*^  (-1.25 to -1.15) |
| Prevalence | 1990-1996 | -0.02  (-0.12 to 0.07) | 1996-2005 | -1.08^*^  (-1.14 to -1.03) | 2005-2010 | -2.40^*^  (-2.58 to -2.22) | 2010-2015 | -5.25^*^  (-5.46 to -5.04) | 2015-2019 | -1.57^*^  (-1.93 to -1.21) | 2019-2021 | 2.15^*^  (1.36 to 2.95) | -1.64^*^  (-1.72 to -1.56) |
| DALY | 1990-1995 | -3.10^*^  (-3.58 to -2.62) | 1995-1999 | -4.66^*^  (-5.64 to -3.67) | 1999-2002 | -9.02^*^  (-10.91 to -7.09) | 2002-2009 | -10.35^*^  (-10.70 to -10.01) | 2009-2015 | -8.99^*^  (-9.46 to -8.52) | 2015-2021 | -5.25^*^  (-5.65 to -4.84) | -7.11^*^  (-7.36 to -6.85) |
| Mortality | 1990-1994 | -3.50^*^  (-4.10 to -2.90) | 1994-1999 | -4.23^*^  (-4.80 to -3.66) | 1999-2002 | -7.96^*^  (-9.71 to -6.17) | 2002-2007 | -9.12^*^  (-9.66 to -8.58) | 2007-2014 | -8.41^*^  (-8.71 to -8.10) | 2014-2021 | -5.48^*^  (-5.75 to -5.22) | -6.53^*^  (-6.76 to -6.31) |
| **China** |  |  |  |  |  |  |  |  |  |  |  |  |  |
| Incidence | 1990-1994 | -2.20^*^  (-2.48 to -1.91) | 1994-2003 | -3.21^*^  (-3.31 to -3.11) | 2003-2006 | -4.68^*^  (-5.62 to -3.73) | 2006-2010 | -14.80^*^  (-15.22 to -14.37) | 2010-2014 | -3.54^*^  (-4.01 to -3.07) | 2014-2021 | -2.08^*^  (-2.22 to -1.95) | -4.60^*^  (-4.72 to -4.47) |
| Prevalence | 1990-1995 | -2.64^*^  (-2.83 to -2.45) | 1995-2003 | -3.80^*^  (-3.91 to -3.68) | 2003-2006 | -5.32^*^  (-6.20 to -4.42) | 2006-2010 | -15.25^*^  (-15.64 to -14.86) | 2010-2015 | -3.40^*^  (-3.68 to -3.12) | 2015-2021 | -2.02^*^  (-2.17 to -1.86) | -4.92^*^  (-5.03 to -4.81) |
| DALY | 1990-1999 | -8.87^*^  (-9.22 to -8.52) | 1999-2005 | -13.10^*^  (-13.83 to -12.36) | 2005-2010 | -16.66^*^  (-17.61 to -15.70) | 2010-2013 | -10.09^*^  (-13.31 to -6.74) | 2013-2019 | -6.11^*^  (-6.89 to -5.31) | 2019-2021 | 0.06  (-3.91 to 4.20) | -10.06^*^  (-10.50 to -9.61) |
| Mortality | 1990-1999 | -9.27^*^  (-9.63 to -8.91) | 1999-2005 | -13.49^*^  (-14.31 to -12.66) | 2005-2009 | -16.07^*^  (-18.13 to -13.96) | 2009-2013 | -11.04^*^  (-13.62 to -8.38) | 2013-2019 | -6.55^*^  (-7.95 to -5.12) | 2019-2021 | -1.22  (-8.00 to 6.06) | -10.23^*^  (-10.86 to -9.59) |
| **Cyprus** |  |  |  |  |  |  |  |  |  |  |  |  |  |
| Incidence | 1990-2001 | 2.30^*^  (2.22 to 2.38) | 2001-2009 | 3.26^*^  (3.11 to 3.40) | 2009-2015 | 0.10  (-0.12 to 0.33) | 2015-2019 | -8.02^*^  (-8.47 to -7.56) | 2019-2021 | -1.54^*^  (-2.53 to -0.55) | ——————————— | | 0.48^*^  (0.37 to 0.58) |
| Prevalence | 1990-2001 | 2.34^*^  (2.26 to 2.42) | 2001-2009 | 3.40^*^  (3.26 to 3.54) | 2009-2015 | 0.76^*^  (0.53 to 0.99) | 2015-2019 | -7.28^*^  (-7.75 to -6.80) | 2019-2021 | -1.57^*^  (-2.60 to -0.52) | ——————————— | | 0.75^*^  (0.65 to 0.86) |
| DALY | 1990-2000 | -1.06^*^  (-1.18 to -0.95) | 2000-2005 | -0.04  (-0.48 to 0.41) | 2005-2010 | 1.00^*^  (0.54 to 1.45) | 2010-2015 | -0.34  (-0.79 to 0.12) | 2015-2019 | -4.93^*^  (-5.64 to -4.20) | 2019-2021 | -3.24^*^  (-4.66 to -1.80) | -1.10^*^  (-1.28 to -0.93) |
| Mortality | 1990-1992 | 1.10  (-2.83 to 5.20) | 1992-1999 | -2.87^*^  (-3.48 to -2.27) | 1999-2006 | -3.51^*^  (-4.00 to -3.01) | 2006-2011 | 0.80  (-0.08 to 1.69) | 2011-2019 | -0.66^*^  (-1.02 to -0.29) | 2019-2021 | -5.47^*^  (-8.13 to -2.74) | -1.78^*^  (-2.15 to -1.42) |
| **Democratic People's Republic of Korea** |  |  |  |  |  |  |  |  |  |  |  |  |  |
| Incidence | 1990-2000 | 3.39^*^  (2.94 to 3.84) | 2000-2005 | 10.34^*^  (8.39 to 12.33) | 2005-2009 | 13.09^*^  (10.00 to 16.27) | 2009-2021 | 0.02  (-0.33 to 0.38) | ——————————— | | ——————————— | | 4.35^*^  (3.86 to 4.84) |
| Prevalence | 1990-2000 | 3.12^*^  (2.67 to 3.56) | 2000-2005 | 10.82^*^  (8.82 to 12.85) | 2005-2009 | 14.04^*^  (10.77 to 17.41) | 2009-2021 | 0.07  (-0.30 to 0.45) | ——————————— | | ——————————— | | 4.47^*^  (3.96 to 4.98) |
| DALY | 1990-2000 | -0.48^*^  (-0.89 to -0.07) | 2000-2005 | 4.66^*^  (2.98 to 6.37) | 2005-2009 | 10.47^*^  (7.46 to 13.57) | 2009-2021 | -0.49^*^  (-0.85 to -0.12) | ——————————— | | ——————————— | | 1.69^*^  (1.23 to 2.15) |
| Mortality | 1990-1994 | -3.25^*^  (-3.48 to -3.02) | 1994-2001 | -2.63^*^  (-2.75 to -2.51) | 2001-2005 | -4.07^*^  (-4.44 to -3.70) | 2005-2010 | -1.04^*^  (-1.29 to -0.80) | 2010-2019 | -2.15^*^  (-2.23 to -2.06) | 2019-2021 | -4.18^*^  (-4.99 to -3.36) | -2.60^*^  (-2.69 to -2.52) |
| **Georgia** |  |  |  |  |  |  |  |  |  |  |  |  |  |
| Incidence | 1990-2001 | 0.08  (-0.10 to 0.27) | 2001-2005 | -2.57^*^  (-4.06 to -1.05) | 2005-2010 | 3.74^*^  (2.74 to 4.75) | 2010-2015 | -5.94^*^  (-6.80 to -5.06) | 2015-2019 | -13.22^*^  (-14.40 to -12.03) | 2019-2021 | -5.10^*^  (-7.68 to -2.44) | -2.83^*^  (-3.18 to -2.48) |
| Prevalence | 1990-2001 | -0.11  (-0.33 to 0.11) | 2001-2005 | -2.52^*^  (-4.29 to -0.71) | 2005-2010 | 4.17^*^  (2.95 to 5.41) | 2010-2015 | -6.27^*^  (-7.27 to -5.26) | 2015-2019 | -13.97^*^  (-15.31 to -12.60) | 2019-2021 | -4.98*  (-7.94 to -1.92) | -2.98^*^  (-3.39 to -2.57) |
| DALY | 1990-1995 | 5.53^*^  (1.70 to 9.50) | 1995-2006 | -15.05^*^  (-16.32 to -13.76) | 2006-2012 | -3.08  (-8.62 to 2.80) | 2012-2021 | -10.71^*^  (-12.94 to -8.43) | ——————————— | | ——————————— | | -8.44^*^  (-9.78 to -7.07) |
| Mortality | 1990-1995 | 7.31^*^  (2.94 to 11.86) | 1995-2007 | -18.18^*^  (-19.38 to -16.97) | 2007-2021 | -8.84^*^  (-9.87 to -7.80) | ——————————— | | ——————————— | | ——————————— | | -10.25^*^  (-11.11 to -9.37) |
| **India** |  |  |  |  |  |  |  |  |  |  |  |  |  |
| Incidence | 1990-1999 | -0.39^*^  (-0.43 to -0.34) | 1999-2010 | 0.18^*^  (0.14 to 0.22) | 2010-2015 | -0.69^*^  (-0.85 to -0.53) | 2015-2019 | 0.62^*^  (0.38 to 0.86) | 2019-2021 | 2.72^*^  (2.26 to 3.19) | ——————————— | | 0.09^*^  (0.04 to 0.14) |
| Prevalence | 1990-2000 | -0.45^*^  (-0.48 to -0.41) | 2000-2010 | 0.12^*^  (0.07 to 0.16) | 2010-2015 | -0.79^*^  (-0.94 to -0.64) | 2015-2019 | 1.19^*^  (0.98 to 1.40) | 2019-2021 | 2.92^*^  (2.50 to 3.33) | ——————————— | | 0.10^*^  (0.06 to 0.15) |
| DALY | 1990-1992 | -2.15^*^  (-4.13 to -0.13) | 1992-1997 | -3.97^*^  (-4.61 to -3.33) | 1997-2001 | -6.77^*^  (-7.83 to -5.69) | 2001-2010 | -4.95^*^  (-5.20 to -4.70) | 2010-2021 | -6.93^*^  (-7.10 to -6.77) | ——————————— | | -5.56^*^  (-5.78 to -5.34) |
| Mortality | 1990-1996 | -2.89^*^  (-3.34 to -2.43) | 1996-2001 | -6.81^*^  (-7.71 to -5.89) | 2001-2007 | -3.80^*^  (-4.51 to -3.08) | 2007-2014 | -5.52^*^  (-6.09 to -4.94) | 2004-2021 | -6.77^*^  (-7.23 to -6.31) | ——————————— | | -5.18^*^  (-5.44 to -4.92) |
| **Indonesia** |  |  |  |  |  |  |  |  |  |  |  |  |  |
| Incidence | 1990-1997 | -2.61^*^  (-2.67 to -2.56) | 1997-2004 | -2.14^*^  (-2.20 to -2.07) | 2004-2011 | -1.35^*^  (-1.42 to -1.29) | 2011-2014 | -3.01^*^  (-3.38 to -2.63) | 2014-2018 | -1.94^*^  (-2.13 to -1.76) | 2018-2021 | 0.54^*^  (0.35 to 0.73) | -1.87^*^  (-1.92 to -1.82) |
| Prevalence | 1990-1995 | -2.94^*^  (-3.00 to -2.88) | 1995-2004 | -2.39^*^  (-2.42 to -2.36) | 2004-2011 | -1.76^*^  (-1.81 to -1.72) | 2011-2014 | -3.47^*^  (-3.73 to -3.21) | 2014-2018 | -2.24^*^  (-2.37 to -2.12) | 2018-2021 | 0.18^*^  (0.05 to 0.30) | -2.18^*^  (-2.21 to -2.15) |
| DALY | 1990-1997 | -9.19^*^  (-9.27 to -9.10) | 1997-2002 | -6.60^*^  (-6.78 to -6.42) | 2002-2009 | -5.84^*^  (-5.93 to -5.75) | 2009-2012 | -6.68^*^  (-7.20 to -6.15) | 2012-2016 | -5.54^*^  (-5.83 to -5.25) | 2016-2021 | -3.80^*^  (-3.94 to -3.66) | -6.45^*^  (-6.52 to -6.38) |
| Mortality | 1990-1997 | -8.75^*^  (-8.83 to -8.68) | 1997-2002 | -5.87^*^  (-6.04 to -5.71) | 2002-2008 | -4.74^*^  (-4.86 to -4.62) | 2008-2013 | -5.17^*^  (-5.35 to -5.00) | 2013-2016 | -4.62^*^  (-5.22 to -4.01) | 2016-2021 | -3.11^*^  (-3.26 to -2.97) | -5.64^*^  (-5.72 to -5.57) |
| **Iran (Islamic Republic of)** |  |  |  |  |  |  |  |  |  |  |  |  |  |
| Incidence | 1990-1994 | -4.35^*^  (-4.46 to -4.24) | 1994-1999 | -3.82^*^  (-3.93 to -3.72) | 1999-2003 | -2.60^*^  (-2.77 to -2.42) | 2003-2015 | -1.73^*^  (-1.76 to -1.70) | 2015-2018 | 0.11  (-0.29 to 0.51) | 2018-2021 | 0.57^*^  (0.37 to 0.77) | -2.13^*^  (-2.18 to -2.08) |
| Prevalence | 1990-1994 | -4.86^*^  (-4.98 to -4.74) | 1994-1999 | -4.25^*^  (-4.37 to -4.13) | 1999-2003 | -2.98^*^  (-3.18 to -2.78) | 2003-2015 | -1.96^*^  (-1.99 to -1.93) | 2015-2021 | 0.23^*^  (0.15 to 0.31) | ——————————— | | -2.43^*^  (-2.46 to -2.39) |
| DALY | 1990-1993 | -9.39^*^  (-12.30 to -6.38) | 1993-1999 | -5.42^*^  (-6.41 to -4.41) | 1999-2002 | -8.57^*^  (-11.97 to -5.05) | 2002-2010 | -6.14^*^  (-6.54 to -5.74) | 2010-2019 | -4.93^*^  (-5.28 to -4.58) | 2019-2021 | -8.13^*^  (-12.09 to -3.98) | -6.34^*^  (-6.87 to -5.81) |
| Mortality | 1990-1993 | -9.06^*^  (-10.97 to -7.11) | 1993-1999 | -5.14^*^  (-5.86 to -4.42) | 1999-2002 | -8.54^*^  (-11.40 to -5.59) | 2002-2010 | -6.46^*^  (-6.87 to -6.04) | 2010-2019 | -4.60^*^  (-5.02 to -4.18) | 2019-2021 | -10.09^*^  (-14.55 to -5.41) | -6.37^*^  (-6.84 to -5.90) |
| **Iraq** |  |  |  |  |  |  |  |  |  |  |  |  |  |
| Incidence | 1990-1996 | -0.63^*^  (-0.67 to -0.59) | 1996-2000 | -1.08^*^  (-1.19 to -0.96) | 2000-2009 | 0.13^*^  (0.11 to 0.16) | 2009-2012 | -2.18^*^  (-2.43 to -1.92) | 2012-2018 | -3.85^*^  (-3.91 to -3.80) | 2018-2021 | -1.78^*^  (-1.92 to -1.63) | -1.36^*^  (-1.39 to -1.33) |
| Prevalence | 1990-1996 | -0.92^*^  (-0.98 to -0.87) | 1996-2000 | -1.29^*^  (-1.45 to -1.13) | 2000-2010 | -0.30^*^  (-0.33 to -0.26) | 2010-2013 | -3.51^*^  (-3.92 to -3.10) | 2013-2018 | -4.22^*^  (-4.36 to -4.09) | 2018-2021 | -2.03^*^  (-2.25 to -1.80) | -1.67^*^  (-1.72 to -1.62) |
| DALY | 1990-1995 | -2.39^*^  (-4.00 to -0.75) | 1995-2008 | -5.05^*^  (-5.45 to -4.64) | 2008-2013 | -7.39^*^  (-9.25 to -5.49) | 2013-2021 | -3.84^*^  (-4.52 to -3.15) | ——————————— | | ——————————— | | -4.70^*^  (-5.14 to -4.25) |
| Mortality | 1990-1997 | -2.88^*^  (-3.56 to -2.19) | 1997-2001 | -6.92^*^  (-9.52 to -4.24) | 2001-2007 | -4.46^*^  (-5.69 to -3.21) | 2007-2013 | -7.53^*^  (-8.72 to -6.32) | 2013-2021 | -2.73^*^  (-3.41 to -2.04) | ——————————— | | -4.59^*^  (-5.09 to -4.08) |
| **Israel** |  |  |  |  |  |  |  |  |  |  |  |  |  |
| Incidence | 1990-2007 | 3.30^*^  (3.24 to 3.36) | 2007-2011 | 0.64  (-0.08 to 1.37) | 2011-2014 | -7.36^*^  (-8.67 to -6.03) | 2014-2019 | -8.92^*^  (-9.30 to -8.53) | 2019-2021 | -2.21^*^  (-3.49 to -0.91) | ——————————— | | -0.52^*^  (-0.71 to -0.34) |
| Prevalence | 1990-2007 | 3.46^*^  (3.37 to 3.55) | 2007-2011 | 0.88  (-0.16 to 1.94) | 2011-2019 | -7.97^*^  (-8.21 to -7.73) | 2019-2021 | -2.66^*^  (-4.50 to -0.79) | ——————————— | | ——————————— | | -0.34^*^  (-0.53 to -0.15) |
| DALY | 1990-1992 | 3.75^*^  (0.09 to 7.54) | 1992-1997 | 0.88  (-0.33 to 2.11) | 1997-2000 | 7.55^*^  (3.70 to 11.55) | 2000-2008 | 4.85^*^  (4.41 to 5.30) | 2008-2011 | -1.72  (-4.71 to 1.37) | 2011-2021 | -5.30^*^  (-5.51 to -5.09) | 0.38  (-0.14 to 0.91) |
| Mortality | 1990-1997 | 0.79  (-0.60 to 2.21) | 1997-2000 | 24.16^*^  (10.96 to 38.93) | 2000-2005 | 3.21  (-0.48 to 7.03) | 2005-2008 | 12.35^*^  (0.29 to 25.86) | 2008-2021 | -2.32^*^  (-3.03 to -1.61) | ——————————— | | 2.97^*^  (1.34 to 4.62) |
| **Japan** |  |  |  |  |  |  |  |  |  |  |  |  |  |
| Incidence | 1990-2000 | 0.21^*^  (0.12 to 0.31) | 2002-2004 | 9.94^*^  (9.28 to 10.59) | 2004-2007 | 1.52^*^  (0.38 to 2.67) | 2007-2021 | 1.52^*^  (0.38 to 2.67) | ——————————— | | ——————————— | | 1.20^*^  (1.07 to 1.34) |
| Prevalence | 1990-2000 | 0.15^*^  (0.05 to 0.25) | 2002-2004 | 8.47^*^  (7.86 to 9.08) | 2004-2007 | 1.45^*^  (0.41 to 2.51) | 2007-2021 | -0.54^*^  (-0.58 to -0.49) | ——————————— | | ——————————— | | 1.00^*^  (0.87 to 1.12) |
| DALY | 1990-2000 | 0.33^*^  (0.22 to 0.45) | 2000-2005 | 6.47^*^  (5.98 to 6.96) | 2005-2012 | -0.13  (-0.37 to 0.11) | 2012-2021 | -1.36^*^  (-1.50 to -1.23) | ——————————— | | ——————————— | | 0.69^*^  (0.59 to 0.79) |
| Mortality | 1990-2003 | -0.15  (-0.58 to 0.28) | 2003-2006 | 7.37  (-1.82 to 17.41) | 2006-2012 | 0.18  (-1.92 to 2.32) | 2012-2021 | -3.33^*^  (-4.22 to -2.42) | ——————————— | | ——————————— | | -0.32  (-1.27 to 0.63) |
| **Jordan** |  |  |  |  |  |  |  |  |  |  |  |  |  |
| Incidence | 1990-2003 | 0.83^*^  (0.78 to 0.88) | 2003-2010 | 2.32^*^  (2.14 to 2.51) | 2010-2015 | -0.45^*^  (-0.80 to -0.10) | 2015-2019 | -11.32^*^  (-11.81 to -10.82) | 2019-2021 | 1.16^*^  (0.13 to 2.20) | ——————————— | | -0.68^*^  (-0.79 to -0.57) |
| Prevalence | 1990-2002 | 0.71^*^  (0.64 to 0.78) | 2002-2010 | 2.26^*^  (2.07 to 2.44) | 2010-2015 | -0.57^*^  (-1.00 to -0.14) | 2015-2019 | -12.28^*^  (-12.88 to -11.68) | 2019-2021 | 1.15  (-0.20 to 2.51) | ——————————— | | -0.86^*^  (-1.00 to -0.71) |
| DALY | 1990-2005 | -2.55^*^  (-2.67 to -2.44) | 2005-2008 | -3.68^*^  (-6.05 to -1.24) | 2008-2014 | -1.66^*^  (-2.26 to -1.06) | 2014-2019 | -7.25^*^  (-8.12 to -6.37) | 2019-2021 | -1.03  (-4.15 to 2.19) | ———— | | -3.17^*^  (-3.51 to -2.83) |
| Mortality | 1990-2005 | -3.21^*^  (-3.37 to -3.04) | 2005-2008 | -7.97^*^  (-11.76 to -4.02) | 2008-2018 | -3.29^*^  (-3.71 to -2.87) | 2018-2021 | -0.88  (-3.57 to 1.89) | ——————————— | | ——————————— | | -3.48^*^  (-3.95 to -3.02) |
| **Kazakhstan** |  |  |  |  |  |  |  |  |  |  |  |  |  |
| Incidence | 1990-1996 | -2.69^*^  (-2.84 to -2.55) | 1996-2000 | -5.34^*^  (-5.76 to -4.92) | 2000-2005 | -3.39^*^  (-3.70 to -3.07) | 2005-2009 | -2.08^*^  (-2.66 to -1.50) | 2009-2015 | -0.09  (-0.38 to 0.21) | 2015-2021 | -3.46^*^  (-3.69 to -3.24) | -2.72^*^  (-2.84 to -2.60) |
| Prevalence | 1990-1995 | -3.23^*^  (-3.39 to -3.08) | 1995-2000 | -5.48^*^  (-5.72 to -5.25) | 2000-2004 | -4.08^*^  (-4.53 to -3.64) | 2004-2009 | -2.47^*^  (-2.81 to -2.13) | 2009-2015 | 0.15  (-0.14 to 0.43) | 2015-2021 | -2.80^*^  (-3.02 to -2.58) | -2.86^*^  (-2.97 to -2.75) |
| DALY | 1990-1994 | 4.42^*^  (2.18 to 6.70) | 1994-1997 | -3.80  (-11.82 to 4.95) | 1997-2004 | -20.62^*^  (-21.89 to -19.33) | 2004-2008 | -5.35^*^  (-9.80 to -0.68) | 2008-2013 | -18.73^*^  (-21.54 to -15.83) | 2013-2021 | -5.96^*^  (-7.30 to -4.60) | -10.12^*^  (-11.21 to -9.02) |
| Mortality | 1990-1994 | 5.13^*^  (2.26 to 8.07) | 1994-1997 | -3.59  (-13.69 to 7.69) | 1997-2004 | -21.04^*^  (-22.63 to -19.41) | 2004-2008 | -6.31^*^  (-11.99 to -0.26) | 2008-2014 | -20.97^*^  (-23.47 to -18.39) | 2014-2021 | -5.90^*^  (-7.61 to -4.16) | -11.14^*^  (-12.47 to -9.79) |
| **Kuwait** |  |  |  |  |  |  |  |  |  |  |  |  |  |
| Incidence | 1990-1994 | -3.93^*^  (-4.16 to -3.70) | 1994-2005 | 1.19^*^  (1.13 to 1.26) | 2005-2015 | 1.61^*^  (1.54 to 1.68) | 2015-2019 | -1.94^*^  (-2.30 to -1.58) | 2019-2021 | -6.24^*^  (-6.93 to -5.55) | ——————————— | | -0.25^*^  (-0.33 to -0.18) |
| Prevalence | 1990-1994 | -4.35^*^  (-4.58 to -4.13) | 1994-2006 | 1.20^*^  (1.14 to 1.25) | 2006-2015 | 1.63^*^  (1.54 to 1.72) | 2015-2019 | -2.23^*^  (-2.61 to -1.86) | 2019-2021 | -6.99^*^  (-7.71 to -6.26) | ——————————— | | -0.40^*^  (-0.48 to -0.33) |
| DALY | 1990-1996 | -10.74^*^  (-13.11 to -8.31) | 1996-2000 | 8.04  (-2.58 to 19.82) | 2000-2021 | -0.70^*^  (-1.24 to -0.17) | ——————————— | | ——————————— | | ——————————— | | -1.67^*^  (-3.04 to -0.28) |
| Mortality | 1990-1997 | -17.99^*^  (-21.89 to -13.89) | 1997-2000 | 34.97  (-1.58 to 85.09) | 2000-2021 | -3.63^*^  (-4.56 to -2.69) | ——————————— | | ——————————— | | ——————————— | | -4.00^*^  (-6.97 to -0.93) |
| **Kyrgyzstan** |  |  |  |  |  |  |  |  |  |  |  |  |  |
| Incidence | 1990-1996 | -1.90^*^  (-2.08 to -1.71) | 1996-2005 | -3.31^*^  (-3.43 to -3.19) | 2005-2015 | -1.55^*^  (-1.68 to -1.42) | 2015-2018 | -4.84^*^  (-6.36 to -3.29) | 2018-2021 | -11.19^*^  (-11.91 to -10.47) | ——————————— | | -3.42^*^  (-3.59 to -3.25) |
| Prevalence | 1990-1996 | -2.31^*^  (-2.40 to -2.21) | 1996-2005 | -3.57^*^  (-3.64 to -3.51) | 2005-2016 | -1.67^*^  (-1.73 to -1.61) | 2016-2019 | -6.71^*^  (-7.54 to -5.88) | 2019-2021 | -12.97^*^  (-13.75 to -12.18) | ——————————— | | -3.61^*^  (-3.71 to -3.51) |
| DALY | 1990-1999 | -0.84  (-2.13 to 0.46) | 1999-2004 | -16.54^*^  (-20.82 to -12.02) | 2004-2007 | -2.32  (-15.39 to 12.76) | 2007-2010 | -14.83^*^  (-24.92 to -3.38) | 2010-2021 | -10.33^*^  (-11.44 to -9.20) | ——————————— | | -8.43^*^  (-10.22 to -6.61) |
| Mortality | 1990-1999 | -0.90  (-2.17 to 0.39) | 1999-2004 | -17.00^*^  (-21.37 to -12.40) | 2004-2007 | -3.83  (-17.35 to 11.90) | 2007-2018 | -12.80^*^  (-13.89 to -11.70) | 2018-2021 | -4.14  (-13.94 to 6.77) | ——————————— | | -8.53^*^  (-10.29 to -6.73) |
| **Lao People's Democratic Republic** |  |  |  |  |  |  |  |  |  |  |  |  |  |
| Incidence | 1990-2001 | -1.43^*^  (-1.47 to -1.40) | 2001-2005 | -0.61^*^  (-0.89 to -0.34) | 2005-2010 | 1.11^*^  (0.94 to 1.29) | 2010-2016 | -1.45^*^  (-1.57 to -1.33) | 2016-2019 | -0.61^*^  (-1.17 to -0.06) | 2019-2021 | 0.94^*^  (0.37 to 1.52) | -0.69^*^  (-0.77 to -0.61) |
| Prevalence | 1990-2001 | -2.07^*^  (-2.10 to -2.04) | 2001-2005 | -1.15^*^  (-1.39 to -0.92) | 2005-2010 | 0.69^*^  (0.53 to 0.84) | 2010-2016 | -1.81^*^  (-1.92 to -1.70) | 2016-2019 | -0.87^*^  (-1.33 to -0.40) | 2019-2021 | 0.68^*^  (0.18 to 1.18) | -1.17^*^  (-1.23 to -1.10) |
| DALY | 1990-1994 | -4.09^*^  (-5.22 to -2.93) | 1994-2002 | -6.60^*^  (-7.02 to -6.18) | 2002-2017 | -8.91^*^  (-9.07 to -8.76) | 2017-2021 | -7.28^*^  (-8.47 to -6.06) | ——————————— | | ——————————— | | -7.50^*^  (-7.73 to -7.26) |
| Mortality | 1990-1994 | -4.42^*^  (-5.16 to -3.67) | 1994-2000 | -5.89^*^  (-6.36 to -5.42) | 2000-2017 | -8.33^*^  (-8.41 to -8.25) | 2017-2021 | -6.26^*^  (-7.01 to -5.51) | ——————————— | | ——————————— | | -7.10^*^  (-7.26 to -6.94) |
| **Lebanon** |  |  |  |  |  |  |  |  |  |  |  |  |  |
| Incidence | 1990-1993 | 4.71^*^  (4.46 to 4.96) | 1993-1998 | 2.07^*^  (1.93 to 2.22) | 1998-2005 | 1.04^*^  (0.97 to 1.12) | 2005-2015 | 0.23^*^  (0.18 to 0.27) | 2015-2018 | -0.47  (-0.93 to 0.00) | 2018-2021 | 0.90^*^  (0.64 to 1.15) | 1.13^*^  (1.07 to 1.19) |
| Prevalence | 1990-1993 | 4.91^*^  (4.67 to 5.15) | 1993-1999 | 1.92^*^  (1.81 to 2.02) | 1999-2005 | 0.89^*^  (0.79 to 0.99) | 2005-2015 | 0.15^*^  (0.10 to 0.19) | 2015-2018 | -0.56^*^  (-1.04 to -0.08) | 2018-2021 | 0.97^*^  (0.71 to 1.23) | 1.09^*^  (1.03 to 1.16) |
| DALY | 1990-2004 | -3.86^*^  (-4.33 to -3.39) | 2004-2021 | -1.48^*^  (-1.79 to -1.17) | ——————————— | | ——————————— | | ——————————— | | ——————————— | | -2.56^*^  (-2.82 to -2.30) |
| Mortality | 1990-1999 | -5.02^*^  (-5.76 to -4.28) | 1999-2021 | -2.20^*^  (-2.42 to -1.98) | ——————————— | | ——————————— | | ——————————— | | ——————————— | | -3.03^*^  (-3.28 to -2.77) |
| **Malaysia** |  |  |  |  |  |  |  |  |  |  |  |  |  |
| Incidence | 1990-1999 | 1.79^*^  (1.75 to 1.83) | 1999-2005 | 2.56^*^  (2.46 to 2.66) | 2005-2014 | 4.17^*^  (4.12 to 4.22) | 2014-2019 | 1.12^*^  (0.97 to 1.27) | 2019-2021 | 2.69^*^  (2.21 to 3.16) | ——————————— | | 2.57^*^  (2.53 to 2.62) |
| Prevalence | 1990-1998 | 1.94^*^  (1.87 to 2.01) | 1998-2005 | 2.61^*^  (2.50 to 2.72) | 2005-2014 | 4.55^*^  (4.47 to 4.62) | 2014-2019 | 1.06^*^  (0.84 to 1.29) | 2019-2021 | 2.34^*^  (1.63 to 3.06) | ——————————— | | 2.72^*^  (2.66 to 2.79) |
| DALY | 1990-2002 | -3.98^*^  (-4.31 to -3.64) | 2002-2021 | 0.62^*^  (0.46 to 0.78) | ——————————— | | ——————————— | | ——————————— | | ——————————— | | -1.19^*^  (-1.34 to -1.03) |
| Mortality | 1990-2002 | -3.51^*^  (-4.00 to -3.02) | 2002-2021 | -1.41^*^  (-1.64 to -1.18) | ——————————— | | ——————————— | | ——————————— | | ——————————— | | -2.23^*^  (-2.46 to -2.00) |
| **Maldives** |  |  |  |  |  |  |  |  |  |  |  |  |  |
| Incidence | 1990-1995 | 0.30^*^  (0.21 to 0.40) | 1995-2004 | -0.54^*^  (-0.59 to -0.48) | 2004-2011 | 1.34^*^  (1.25 to 1.43) | 2011-2019 | 0.00  (-0.08 to 0.07) | 2019-2021 | 2.53^*^  (1.93 to 3.13) | ——————————— | | 0.35^*^  (0.31 to 0.40) |
| Prevalence | 1990-1995 | -0.38^*^  (-0.50 to -0.26) | 1995-2002 | -1.39^*^  (-1.49 to -1.28) | 2002-2005 | -0.12  (-0.78 to 0.53) | 2005-2011 | 1.33^*^  (1.18 to 1.49) | 2011-2019 | -0.20^*^  (-0.30 to -0.10) | 2019-2021 | 2.41^*^  (1.61 to 3.22) | -0.03  (-0.12 to 0.06) |
| DALY | 1990-2002 | -13.33^*^  (-13.59 to -13.07) | 2002-2005 | -10.15^*^  (-14.12 to -6.01) | 2005-2011 | -5.77^*^  (-6.67 to -4.86) | 2011-2021 | -3.28^*^  (-3.62 to -2.94) | ——————————— | | ——————————— | | -8.43^*^  (-8.86 to -7.99) |
| Mortality | 1990-2003 | -13.37^*^  (-13.54 to -13.20) | 2003-2008 | -8.27^*^  (-9.33 to -7.20) | 2008-2014 | -5.14^*^  (-5.92 to -4.35) | 2014-2021 | -3.42^*^  (-3.93 to -2.90) | ——————————— | | ——————————— | | -8.80^*^  (-9.05 to -8.56) |
| **Mongolia** |  |  |  |  |  |  |  |  |  |  |  |  |  |
| Incidence | 1990-1994 | -5.80^*^  (-6.71 to -4.88) | 1994-2002 | 0.84^*^  (0.45 to 1.23) | 2002-2011 | -3.00^*^  (-3.34 to -2.66) | 2011-2015 | -21.23^*^  (-22.40 to -20.05) | 2015-2018 | -12.33^*^  (-14.47 to -10.13) | 2018-2021 | -3.61^*^  (-4.80 to -2.40) | -5.96^*^  (-6.29 to -5.63) |
| Prevalence | 1990-1994 | -6.46^*^  (-7.45 to -5.46) | 1994-2002 | 0.82^*^  (0.40 to 1.24) | 2002-2011 | -3.28^*^  (-3.65 to -2.91) | 2011-2015 | -21.82^*^  (-23.08 to -20.54) | 2015-2018 | -12.98^*^  (-15.33 to -10.57) | 2018-2021 | -3.57^*^  (-4.92 to -2.21) | -6.29^*^  (-6.65 to -5.93) |
| DALY | 1990-1996 | -5.14^*^  (-5.99 to -4.29) | 1996-2012 | -7.34^*^  (-7.60 to -7.08) | 2012-2016 | -11.28^*^  (-14.95 to -7.46) | 2016-2021 | -5.34^*^  (-7.25 to -3.39) | ——————————— | | ——————————— | | -7.12^*^  (-7.71 to -6.53) |
| Mortality | 1990-1996 | -4.82^*^  (-5.66 to -3.97) | 1996-2004 | -8.47^*^  (-9.21 to -7.72) | 2004-2013 | -7.24^*^  (-8.08 to -6.38) | 2013-2016 | -11.06^*^  (-18.38 to -3.08) | 2016-2021 | -4.98^*^  (-6.89 to -3.04) | ——————————— | | -7.11^*^  (-7.95 to -6.27) |
| **Myanmar** |  |  |  |  |  |  |  |  |  |  |  |  |  |
| Incidence | 1990-1996 | 0.27^*^  (0.22 to 0.32) | 1996-2001 | -0.31^*^  (-0.40 to -0.22) | 2001-2005 | -1.32^*^  (-1.47 to -1.17) | 2005-2015 | -2.28^*^  (-2.31 to -2.25) | 2015-2019 | 0.55^*^  (0.36 to 0.73) | 2019-2021 | -0.89^*^  (-1.26 to -0.52) | -0.90^*^  (-0.94 to -0.86) |
| Prevalence | 1990-1996 | 0.12^*^  (0.00 to 0.24) | 1996-2001 | -0.90^*^  (-1.12 to -0.68) | 2001-2005 | -2.26^*^  (-2.63 to -1.89) | 2005-2014 | -3.08^*^  (-3.17 to -2.98) | 2014-2021 | -0.42^*^  (-0.55 to -0.29) | ——————————— | | -1.41^*^  (-1.48 to -1.34) |
| DALY | 1990-1993 | -4.32^*^  (-5.44 to -3.19) | 1993-1999 | -7.70^*^  (-8.14 to -7.25) | 1999-2003 | -10.98^*^  (-11.78 to -10.17) | 2003-2010 | -7.95^*^  (-8.17 to -7.72) | 2010-2019 | -6.03^*^  (-6.18 to -5.89) | 2019-2021 | -7.71^*^  (-8.89 to -6.52) | -7.39^*^  (-7.57 to -7.20) |
| Mortality | 1990-1993 | -4.67^*^  (-5.43 to -3.91) | 1993-1999 | -7.36^*^  (-7.64 to -7.08) | 1999-2003 | -10.72^*^  (-11.26 to -10.19) | 2003-2006 | -6.96^*^  (-7.94 to -5.96) | 2006-2009 | -8.07^*^  (-9.03 to -7.11) | 2009-2021 | -5.49^*^  (-5.55 to -5.44) | -6.86^*^  (-7.02 to -6.70) |
| **Nepal** |  |  |  |  |  |  |  |  |  |  |  |  |  |
| Incidence | 1990-2000 | -0.87^*^  (-0.94 to -0.80) | 2000-2005 | -2.17^*^  (-2.44 to -1.91) | 2005-2010 | 2.49^*^  (2.19 to 2.80) | 2010-2018 | -3.70^*^  (-3.82 to -3.58) | 2018-2021 | -5.72^*^  (-6.15 to -5.28) | ——————————— | | -1.77^*^  (-1.85 to -1.69) |
| Prevalence | 1990-2000 | -1.41^*^  (-1.51 to -1.30) | 2000-2005 | -2.63^*^  (-3.01 to -2.24) | 2005-2010 | 2.54^*^  (2.12 to 2.96) | 2010-2019 | -4.23^*^  (-4.36 to -4.10) | 2019-2021 | -6.75^*^  (-8.01 to -5.47) | ——————————— | | -2.16^*^  (-2.29 to -2.04) |
| DALY | 1990-1997 | -5.61^*^  (-6.03 to -5.19) | 1997-2003 | -7.74^*^  (-8.48 to -6.99) | 2003-2009 | -5.68^*^  (-6.41 to -4.95) | 2009-2012 | -11.44^*^  (-14.71 to -8.04) | 2012-2021 | -7.05^*^  (-7.51 to -6.58) | ——————————— | | -7.03^*^  (-7.43 to -6.63) |
| Mortality | 1990-1996 | -4.60^*^  (-4.89 to -4.30) | 1996-2003 | -6.86^*^  (-7.16 to -6.56) | 2003-2009 | -5.47^*^  (-5.90 to -5.03) | 2009-2013 | -9.24^*^  (-10.26 to -8.21) | 2013-2021 | -5.19^*^  (-5.49 to -4.88) | ——————————— | | -6.04^*^  (-6.22 to -5.85) |
| **Oman** |  |  |  |  |  |  |  |  |  |  |  |  |  |
| Incidence | 1990-1994 | -4.85^*^  (-5.05 to -4.64) | 1994-1997 | -2.53^*^  (-3.19 to -1.86) | 1997-2005 | -1.37^*^  (-1.47 to -1.28) | 2005-2013 | 2.11^*^  (2.01 to 2.21) | 2013-2019 | 3.46^*^  (3.30 to 3.63) | 2019-2021 | -0.08  (-0.82 to 0.66) | -0.05  (-0.14 to 0.04) |
| Prevalence | 1990-1993 | -5.58^*^  (-5.92 to -5.24) | 1993-1996 | -3.62^*^  (-4.31 to -2.93) | 1996-2005 | -1.68^*^  (-1.76 to -1.60) | 2005-2013 | 2.09^*^  (1.98 to 2.20) | 2013-2019 | 3.80^*^  (3.63 to 3.98) | 2019-2021 | -0.13  (-0.84 to 0.59) | -0.16^*^  (-0.25 to -0.06) |
| DALY | 1990-1995 | -9.59^*^  (-10.26 to -8.90) | 1995-2001 | -7.31^*^  (-7.84 to -6.78) | 2001-2005 | -5.66^*^  (-6.57 to -4.75) | 2005-2013 | 1.02^*^  (0.76 to 1.27) | 2013-2018 | 1.69^*^  (1.07 to 2.31) | 2018-2021 | -3.12^*^  (-4.24 to -1.99) | -3.56^*^  (-3.79 to -3.32) |
| Mortality | 1990-1994 | -9.14^*^  (-10.33 to -7.94) | 1994-2005 | -7.14^*^  (-7.43 to -6.85) | 2005-2016 | 0.74^*^  (0.41 to 1.07) | 2016-2021 | -1.99^*^  (-2.87 to -1.10) | ——————————— | | ——————————— | | -3.85^*^  (-4.10 to -3.60) |
| **Pakistan** |  |  |  |  |  |  |  |  |  |  |  |  |  |
| Incidence | 1990-1995 | -1.58^*^  (-1.83 to -1.33) | 1995-2000 | -2.39^*^  (-2.74 to -2.05) | 2000-2004 | -1.09^*^  (-1.64 to -0.53) | 2004-2012 | -0.17^*^  (-0.32 to -0.02) | 2012-2019 | -2.11^*^  (-2.30 to -1.93) | 2019-2021 | 0.10  (-0.97 to 1.18) | -1.30^*^  (-1.42 to -1.18) |
| Prevalence | 1990-1995 | -1.70^*^  (-1.92 to -1.48) | 1995-2000 | -2.71^*^  (-3.02 to -2.40) | 2000-2004 | -1.37^*^  (-1.85 to -0.89) | 2004-2012 | -0.52^*^  (-0.65 to -0.38) | 2012-2019 | -2.25^*^  (-2.40 to -2.09) | 2019-2021 | 0.46  (-0.44 to 1.36) | -1.50^*^  (-1.61 to -1.40) |
| DALY | 1990-1993 | -3.19^*^  (-4.21 to -2.15) | 1993-2009 | -4.12^*^  (-4.20 to -4.04) | 2009-2012 | -6.21^*^  (-7.99 to -4.40) | 2012-2015 | -4.18^*^  (-6.19 to -2.13) | 2015-2019 | -7.77^*^  (-8.80 to -6.72) | 2019-2021 | -10.46^*^  (-12.65 to -8.22) | -5.14^*^  (-5.46 to -4.82) |
| Mortality | 1990-1995 | -2.22^*^  (-2.56 to -1.88) | 1995-2008 | -4.17^*^  (-4.26 to -4.08) | 2008-2012 | -5.89^*^  (-6.60 to -5.19) | 2012-2015 | -4.40^*^  (-5.75 to -3.03) | 2015-2018 | -5.81^*^  (-7.20 to -4.40) | 2018-2021 | -7.14^*^  (-7.93 to -6.35) | -4.56^*^  (-4.77 to -4.34) |
| **Palestine** |  |  |  |  |  |  |  |  |  |  |  |  |  |
| Incidence | 1990-1994 | -1.20^*^  (-1.56 to -0.84) | 1994-2012 | 1.54^*^  (1.50 to 1.59) | 2012-2016 | -1.46^*^  (-2.07 to -0.84) | 2016-2019 | -14.01^*^  (-15.11 to -12.91) | 2019-2021 | -6.74^*^  (-7.93 to -5.54) | ——————————— | | -1.36^*^  (-1.52 to -1.20) |
| Prevalence | 1990-1994 | -1.38^*^  (-1.76 to -1.00) | 1994-2012 | 1.52^*^  (1.47 to 1.57) | 2012-2016 | -1.59^*^  (-2.26 to -0.92) | 2016-2019 | -15.15^*^  (-16.31 to -13.97) | 2019-2021 | -7.28^*^  (-8.53 to -6.01) | ——————————— | | -1.58^*^  (-1.75 to -1.40) |
| DALY | 1990-1997 | -10.39^*^  (-11.06 to -9.72) | 1997-2000 | -7.16^*^  (-11.08 to -3.06) | 2000-2006 | -2.10^*^  (-3.00 to -1.19) | 2006-2015 | -4.28^*^  (-4.72 to -3.84) | 2015-2021 | -8.47^*^  (-9.22 to -7.73) | ——————————— | | -6.38^*^  (-6.84 to -5.92) |
| Mortality | 1990-1999 | -10.30^*^  (-10.59 to -10.00) | 1999-2007 | -4.11^*^  (-4.55 to -3.67) | 2007-2010 | -8.62^*^  (-11.74 to -5.39) | 2010-2018 | -5.90^*^  (-6.35 to -5.46) | 2018-2021 | -2.90^*^  (-4.92 to -0.84) | ——————————— | | -6.73^*^  (-7.11 to -6.35) |
| **Philippines** |  |  |  |  |  |  |  |  |  |  |  |  |  |
| Incidence | 1990-1995 | -1.57^*^  (-1.78 to -1.37) | 1995-1999 | -2.38^*^  (-2.84 to -1.91) | 1999-2010 | -3.26^*^  (-3.34 to -3.18) | 2010-2015 | -5.95^*^  (-6.26 to -5.65) | 2015-2018 | -1.47^*^  (-2.40 to -0.53) | 2018-2021 | 0.56^*^  (0.10 to 1.03) | -2.78^*^  (-2.91 to -2.66) |
| Prevalence | 1990-1997 | -2.01^*^  (-2.15 to -1.87) | 1997-2010 | -3.49^*^  (-3.56 to -3.43) | 2010-2015 | -6.15^*^  (-6.48 to -5.81) | 2015-2018 | -1.73^*^  (-2.74 to -0.70) | 2018-2021 | 0.31  (-0.19 to 0.80) | ——————————— | | -3.06^*^  (-3.18 to -2.94) |
| DALY | 1990-1998 | -7.27^*^  (-7.44 to -7.09) | 1998-2001 | -2.40^*^  (-3.93 to -0.86) | 2001-2004 | -6.92^*^  (-8.39 to -5.43) | 2004-2011 | -4.20^*^  (-4.43 to -3.97) | 2011-2014 | -7.17^*^  (-8.47 to -5.86) | 2014-2021 | -3.80^*^  (-3.99 to -3.60) | -5.30^*^  (-5.53 to -5.06) |
| Mortality | 1990-1997 | -7.16^*^  (-7.42 to -6.90) | 1997-2000 | -2.86^*^  (-4.85 to -0.82) | 2000-2004 | -5.13^*^  (-6.06 to -4.18) | 2004-2009 | -3.16^*^  (-3.75 to -2.55) | 2009-2015 | -5.33^*^  (-5.73 to -4.93) | 2015-2021 | -3.36^*^  (-3.69 to -3.04) | -4.76^*^  (-5.01 to -4.51) |
| **Qatar** |  |  |  |  |  |  |  |  |  |  |  |  |  |
| Incidence | 1990-1999 | 1.55^*^  (1.46 to 1.64) | 1999-2005 | 2.19^*^  (1.98 to 2.40) | 2005-2010 | -1.03^*^  (-1.30 to -0.76) | 2010-2015 | -3.05^*^  (-3.30 to -2.79) | 2015-2021 | 3.00^*^  (2.86 to 3.13) | ——————————— | | 0.77^*^  (0.70 to 0.85) |
| Prevalence | 1990-1999 | 1.48^*^  (1.39 to 1.58) | 1999-2005 | 2.24^*^  (2.02 to 2.46) | 2005-2010 | -1.21^*^  (-1.50 to -0.93) | 2010-2015 | -3.17^*^  (-3.43 to -2.90) | 2015-2021 | 3.08^*^  (2.94 to 3.22) | ——————————— | | 0.73^*^  (0.65 to 0.81) |
| DALY | 1990-1996 | -2.33^*^  (-2.67 to -1.99) | 1996-2006 | -0.59^*^  (-0.76 to -0.42) | 2006-2015 | -3.69^*^  (-3.90 to -3.49) | 2015-2021 | 0.46^*^  (0.09 to 0.82) | ——————————— | | ——————————— | | -1.64^*^  (-1.76 to -1.52) |
| Mortality | 1990-1996 | -2.87^*^  (-3.62 to -2.12) | 1996-2008 | -1.33^*^  (-1.62 to -1.04) | 2008-2015 | -5.82^*^  (-6.60 to -5.04) | 2015-2021 | 0.62  (-0.33 to 1.58) | ——————————— | | ——————————— | | -2.29^*^  (-2.59 to -2.00) |
| **Republic of Korea** |  |  |  |  |  |  |  |  |  |  |  |  |  |
| Incidence | 1990-1994 | 0.99^*^  (0.75 to 1.23) | 1994-2000 | 0.46^*^  (0.28 to 0.64) | 2000-2005 | 1.90^*^  (1.65 to 2.16) | 2005-2011 | -0.26^*^  (-0.43 to -0.08) | 2011-2018 | -5.03^*^  (-5.15 to -4.90) | 2018-2021 | -1.71^*^  (-2.09 to -1.34) | -0.86^*^  (-0.93 to -0.78) |
| Prevalence | 1990-2000 | 0.59^*^  (0.51 to 0.67) | 2000-2005 | 1.89^*^  (1.52 to 2.26) | 2005-2011 | -0.19  (-0.44 to 0.06) | 2011-2018 | -5.15^*^  (-5.33 to -4.98) | 2018-2021 | -1.75^*^  (-2.27 to -1.22) | ——————————— | | -0.91^*^  (-1.00 to -0.81) |
| DALY | 1990-1995 | -5.60^*^  (-5.81 to -5.39) | 1995-1999 | -3.98^*^  (-4.45 to -3.50) | 1999-2011 | -0.97^*^  (-1.04 to -0.89) | 2011-2015 | -2.76^*^  (-3.32 to -2.20) | 2015-2019 | -1.34^*^  (-1.94 to -0.74) | 2019-2021 | -3.34^*^  (-4.57 to -2.09) | -2.55^*^  (-2.69 to -2.41) |
| Mortality | 1990-1993 | -6.72^*^  (-7.77 to -5.67) | 1993-1996 | -8.19^*^  (-10.21 to -6.13) | 1996-2002 | -4.50^*^  (-5.05 to -3.94) | 2002-2006 | -1.54^*^  (-2.98 to -0.07) | 2006-2012 | 1.07^*^  (0.41 to 1.72) | 2012-2021 | -1.03^*^  (-1.28 to -0.77) | -2.65^*^  (-2.97 to -2.33) |
| **Saudi Arabia** |  |  |  |  |  |  |  |  |  |  |  |  |  |
| Incidence | 1990-1994 | -6.61^*^  (-6.95 to -6.26) | 1994-1998 | -2.40^*^  (-3.01 to -1.79) | 1998-2003 | 0.45^*^  (0.00 to 0.90) | 2003-2010 | 4.01^*^  (3.75 to 4.28) | 2010-2015 | -1.24^*^  (-1.70 to -0.77) | 2015-2021 | 2.40^*^  (2.14 to 2.67) | 0.02  (-0.12 to 0.17) |
| Prevalence | 1990-1994 | -7.59^*^  (-7.91 to -7.26) | 1994-1999 | -2.44^*^  (-2.82 to -2.06) | 1999-2003 | 0.79^*^  (0.06 to 1.52) | 2003-2010 | 4.22^*^  (3.94 to 4.50) | 2010-2015 | -1.48^*^  (-1.99 to -0.97) | 2015-2021 | 2.49^*^  (2.21 to 2.76) | -0.15  (-0.30 to 0.01) |
| DALY | 1990-1997 | -8.54^*^  (-8.68 to -8.40) | 1997-2005 | -5.87^*^  (-5.98 to -5.76) | 2005-2011 | -3.82^*^  (-4.00 to -3.63) | 2011-2014 | -5.10^*^  (-6.02 to -4.17) | 2014-2021 | -0.98^*^  (-1.12 to -0.84) | ——————————— | | -4.93^*^  (-5.04 to -4.83) |
| Mortality | 1990-1993 | -7.78^*^  (-8.08 to -7.48) | 1993-1997 | -6.84^*^  (-7.13 to -6.55) | 1997-2006 | -4.74^*^  (-4.81 to -4.68) | 2006-2013 | -3.80^*^  (-3.92 to -3.67) | 2013-2021 | -1.89^*^  (-1.99 to -1.80) | ——————————— | | -4.38^*^  (-4.44 to -4.32) |
| **Singapore** |  |  |  |  |  |  |  |  |  |  |  |  |  |
| Incidence | 1990-1994 | -9.19^*^  (-9.37 to -9.00) | 1994-1999 | -3.48^*^  (-3.69 to -3.27) | 1999-2011 | -0.10^*^  (-0.15 to -0.05) | 2011-2016 | -2.88^*^  (-3.12 to -2.65) | 2016-2019 | 1.02^*^  (0.32 to 1.73) | 2019-2021 | 5.61^*^  (4.84 to 6.38) | -1.86^*^  (-1.95 to -1.77) |
| Prevalence | 1990-1994 | -8.67^*^  (-8.84 to -8.50) | 1994-1999 | -3.34^*^  (-3.55 to -3.14) | 1999-2011 | -0.06^*^  (-0.11 to -0.02) | 2011-2016 | -2.95^*^  (-3.18 to -2.73) | 2016-2019 | 0.51  (-0.18 to 1.21) | 2019-2021 | 5.09^*^  (4.35 to 5.83) | -1.84^*^  (-1.93 to -1.75) |
| DALY | 1990-1992 | -13.29^*^  (-20.51 to -5.43) | 1992-2003 | -4.27^*^  (-4.99 to -3.55) | 2003-2010 | 2.93^*^  (1.07 to 4.83) | 2010-2021 | -4.19^*^  (-4.94 to -3.44) | ——————————— | | ——————————— | | -3.28^*^  (-4.01 to -2.55) |
| Mortality | 1990-1992 | -10.18^*^  (-16.67 to -3.19) | 1992-2003 | -3.64^*^  (-4.22 to -3.06) | 2003-2011 | 3.17^*^  (1.91 to 4.43) | 2011-2021 | -4.24^*^  (-5.07 to -3.40) | ——————————— | | ——————————— | | -2.57^*^  (-3.19 to -1.94) |
| **Sri Lanka** |  |  |  |  |  |  |  |  |  |  |  |  |  |
| Incidence | 1990-1996 | -0.92^*^  (-0.99 to -0.85) | 1996-2000 | -1.45^*^  (-1.67 to -1.23) | 2000-2004 | -0.96^*^  (-1.20 to -0.72) | 2004-2015 | -0.44^*^  (-0.48 to -0.40) | 2015-2019 | 0.51^*^  (0.24 to 0.78) | 2019-2021 | 2.89^*^  (2.31 to 3.48) | -0.40^*^  (-0.46 to -0.33) |
| Prevalence | 1990-1996 | -1.16^*^  (-1.21 to -1.10) | 1996-1999 | -1.80^*^  (-2.18 to -1.42) | 1999-2004 | -1.45^*^  (-1.58 to -1.32) | 2004-2015 | -0.57^*^  (-0.61 to -0.53) | 2015-2019 | 0.34^*^  (0.07 to 0.61) | 2019-2021 | 2.73^*^  (2.16 to 3.31) | -0.62^*^  (-0.68 to -0.56) |
| DALY | 1990-2002 | -9.58^*^  (-9.84 to -9.32) | 2002-2016 | -3.66^*^  (-3.88 to -3.44) | 2016-2021 | -2.18^*^  (-3.26 to -1.08) | ——————————— | | ——————————— | | ——————————— | | -5.76^*^  (-5.97 to -5.55) |
| Mortality | 1990-2002 | -10.83^*^  (-11.07 to -10.59) | 2002-2021 | -4.41^*^  (-4.55 to -4.27) | ——————————— | | ——————————— | | ——————————— | | ——————————— | | -6.95^*^  (-7.07 to -6.82) |
| **Syrian Arab Republic** |  |  |  |  |  |  |  |  |  |  |  |  |  |
| Incidence | 1990-1993 | -2.39^*^  (-2.58 to -2.19) | 1993-1999 | -1.57^*^  (-1.66 to -1.48) | 1999-2006 | 0.82^*^  (0.74 to 0.90) | 2006-2010 | -0.00  (-0.24 to 0.24) | 2010-2015 | -2.92^*^  (-3.07 to -2.76) | 2015-2021 | 1.88^*^  (1.79 to 1.97) | -0.47^*^  (-0.52 to -0.42) |
| Prevalence | 1990-1994 | -2.81^*^  (-2.94 to -2.68) | 1994-1999 | -1.69^*^  (-1.84 to -1.53) | 1999-2006 | 0.78^*^  (0.69 to 0.87) | 2006-2010 | -0.16  (-0.43 to 0.11) | 2010-2015 | -3.17^*^  (-3.35 to -2.98) | 2015-2021 | 1.95^*^  (1.84 to 2.06) | -0.63^*^  (-0.69 to -0.57) |
| DALY | 1990-1995 | -8.45^*^  (-9.33 to -7.57) | 1995-1998 | -10.95^*^  (-13.86 to -7.94) | 1998-2003 | -7.40^*^  (-8.25 to -6.55) | 2003-2006 | -1.08  (-4.05 to 1.98) | 2006-2021 | -2.05^*^  (-2.21 to -1.90) | ——————————— | | -4.78^*^  (-5.21 to -4.34) |
| Mortality | 1990-1995 | -8.34^*^  (-8.82 to -7.86) | 1995-1998 | -10.43^*^  (-12.20 to -8.63) | 1998-2004 | -7.58^*^  (-8.04 to -7.11) | 2004-2012 | -2.17^*^  (-2.49 to -1.85) | 2012-2015 | 0.19  (-2.39 to 2.84) | 2015-2021 | -3.60^*^  (-4.07 to -3.13) | -5.12^*^  (-5.44 to -4.80) |
| **Taiwan (Province of China)** |  |  |  |  |  |  |  |  |  |  |  |  |  |
| Incidence | 1990-1993 | -2.46^*^  (-3.05 to -1.85) | 1993-1998 | -1.03^*^  (-1.48 to -0.57) | 1998-2001 | 2.78^*^  (1.11 to 4.48) | 2001-2005 | 8.82^*^  (7.92 to 9.73) | 2005-2009 | 11.19^*^  (10.32 to 12.07) | 2009-2021 | 1.79^*^  (1.71 to 1.88) | 3.05^*^  (2.83 to 3.28) |
| Prevalence | 1990-1993 | -3.77^*^  (-4.35 to -3.19) | 1993-1998 | -2.13^*^  (-2.60 to -1.65) | 1998-2001 | 2.32^*^  (0.63 to 4.03) | 2001-2005 | 9.01^*^  (8.11 to 9.92) | 2005-2009 | 11.49^*^  (10.63 to 12.35) | 2009-2021 | 1.81^*^  (1.72 to 1.89) | 2.75^*^  (2.52 to 2.98) |
| DALY | 1990-2000 | -6.83^*^  (-7.40 to -6.26) | 2000-2010 | 6.00^*^  (4.82 to 7.20) | 2010-2021 | 1.19^*^  (0.22 to 2.17) | ——————————— | | ——————————— | | ——————————— | | 0.02  (-0.49 to 0.53) |
| Mortality | 1990-2000 | -11.79^*^  (-13.93 to -9.60) | 2000-2021 | -2.06^*^  (-3.04 to -1.07) | ——————————— | | ——————————— | | ——————————— | | ——————————— | | -5.31^*^  (-6.25 to -4.36) |
| **Tajikistan** |  |  |  |  |  |  |  |  |  |  |  |  |  |
| Incidence | 1990-1995 | 1.05^*^  (0.81 to 1.28) | 1995-2000 | -2.17^*^  (-2.50 to -1.85) | 2000-2006 | -3.84^*^  (-4.08 to -3.60) | 2006-2015 | -2.45^*^  (-2.57 to -2.32) | 2015-2019 | -5.14^*^  (-5.72 to -4.55) | 2019-2021 | -1.03  (-2.23 to 0.19) | -2.38^*^  (-2.51 to -2.25) |
| Prevalence | 1990-1995 | 1.03^*^  (0.68 to 1.38) | 1995-2000 | -2.56^*^  (-3.02 to -2.09) | 2000-2007 | -4.52^*^  (-4.78 to -4.26) | 2007-2015 | -2.31^*^  (-2.55 to -2.07) | 2015-2019 | -4.55^*^  (-5.41 to -3.67) | 2019-2021 | -0.78  (-2.66 to 1.13) | -2.52^*^  (-2.71 to -2.33) |
| DALY | 1990-1995 | 1.04^*^  (0.10 to 1.98) | 1995-2000 | -2.08^*^  (-3.38 to -0.76) | 2000-2006 | -7.41^*^  (-8.32 to -6.49) | 2006-2017 | -5.38^*^  (-5.76 to -4.99) | 2017-2021 | -2.88^*^  (-4.88 to -0.84) | ——————————— | | -3.92^*^  (-4.32 to -3.52) |
| Mortality | 1990-1995 | 1.10^*^  (0.20 to 2.01) | 1995-2000 | -1.90^*^  (-3.16 to -0.63) | 2000-2005 | -7.63^*^  (-8.85 to -6.39) | 2005-2017 | -5.64^*^  (-5.96 to -5.32) | 2017-2021 | -2.67^*^  (-4.62 to -0.67) | ——————————— | | -3.93^*^  (-4.33 to -3.53) |
| **Thailand** |  |  |  |  |  |  |  |  |  |  |  |  |  |
| Incidence | 1990-2001 | -1.28^*^  (-1.34 to -1.22) | 2001-2005 | 0.92^*^  (0.45 to 1.39) | 2005-2010 | 1.98^*^  (1.67 to 2.28) | 2010-2015 | -4.31^*^  (-4.59 to -4.03) | 2015-2019 | -0.20  (-0.64 to 0.24) | 2019-2021 | 2.59^*^  (1.67 to 3.51) | -0.59^*^  (-0.71 to -0.48) |
| Prevalence | 1990-2000 | -1.70^*^  (-1.77 to -1.64) | 2000-2004 | 0.23  (-0.22 to 0.68) | 2004-2010 | 2.10^*^  (1.89 to 2.30) | 2010-2015 | -4.63^*^  (-4.89 to -4.37) | 2015-2019 | -0.52^*^  (-0.92 to -0.12) | 2019-2021 | 2.40^*^  (1.61 to 3.21) | -0.80^*^  (-0.90 to -0.70) |
| DALY | 1990-1997 | -9.24^*^  (-9.81 to -8.66) | 1997-2005 | -4.62^*^  (-4.93 to -4.31) | 2005-2008 | -3.02^*^  (-4.56 to -1.45) | 2008-2014 | -4.14^*^  (-4.49 to -3.79) | 2014-2021 | -1.28^*^  (-1.50 to -1.06) | ——————————— | | -4.70^*^  (-4.92 to -4.49) |
| Mortality | 1990-1997 | -10.30^*^  (-10.63 to -9.97) | 1997-2000 | -5.19^*^  (-7.08 to -3.26) | 2000-2003 | -6.57^*^  (-8.08 to -5.04) | 2003-2013 | -4.94^*^  (-5.05 to -4.83) | 2013-2017 | -1.82^*^  (-2.43 to -1.21) | 2017-2021 | -0.81^*^  (-1.22 to -0.39) | -5.45^*^  (-5.70 to -5.20) |
| **Timor-Leste** |  |  |  |  |  |  |  |  |  |  |  |  |  |
| Incidence | 1990-1994 | 0.47^*^  (0.34 to 0.60) | 1994-2005 | -0.32^*^  (-0.36 to -0.29) | 2005-2010 | 1.44^*^  (1.30 to 1.59) | 2010-2016 | -1.70^*^  (-1.81 to -1.60) | 2016-2021 | -1.34^*^  (-1.44 to -1.25) | ——————————— | | -0.37^*^  (-0.41 to -0.34) |
| Prevalence | 1990-1995 | -0.04  (-0.16 to 0.08) | 1995-2005 | -0.81^*^  (-0.86 to -0.76) | 2005-2010 | 1.24^*^  (1.06 to 1.42) | 2010-2015 | -2.22^*^  (-2.41 to -2.04) | 2015-2021 | -1.77^*^  (-1.86 to -1.68) | ——————————— | | -0.78^*^  (-0.83 to -0.73) |
| DALY | 1990-1993 | -6.45^*^  (-7.96 to -4.91) | 1993-2003 | -8.57^*^  (-8.84 to -8.29) | 2003-2008 | -10.91^*^  (-11.83 to -9.98) | 2008-2013 | -5.84^*^  (-6.68 to -5.00) | 2013-2021 | -3.83^*^  (-4.12 to -3.54) | ——————————— | | -7.11^*^  (-7.37 to -6.85) |
| Mortality | 1990-1992 | -6.13^*^  (-8.50 to -3.71) | 1992-2003 | -7.99^*^  (-8.17 to -7.81) | 2003-2009 | -9.87^*^  (-10.36 to -9.38) | 2009-2015 | -4.67^*^  (-5.13 to -4.21) | 2015-2021 | -2.78^*^  (-3.15 to -2.41) | ——————————— | | -6.62^*^  (-6.83 to -6.41) |
| **Turkey** |  |  |  |  |  |  |  |  |  |  |  |  |  |
| Incidence | 1990-1995 | -0.42^*^  (-0.71 to -0.13) | 1995-2000 | -1.96^*^  (-2.39 to -1.54) | 2000-2005 | 2.72^*^  (2.25 to 3.19) | 2005-2014 | -1.27^*^  (-1.43 to -1.11) | 2014-2019 | -4.92^*^  (-5.39 to -4.45) | 2019-2021 | 0.89  (-0.74 to 2.54) | -1.08^*^  (-1.24 to -0.91) |
| Prevalence | 1990-1995 | -0.72^*^  (-1.02 to -0.43) | 1995-2000 | -2.33^*^  (-2.76 to -1.90) | 2000-2005 | 2.86^*^  (2.38 to 3.35) | 2005-2014 | -1.51^*^  (-1.68 to -1.35) | 2014-2019 | -5.42^*^  (-5.92 to -4.91) | 2019-2021 | 0.77  (-1.00 to 2.58) | -1.32^*^  (-1.50 to -1.15) |
| DALY | 1990-2006 | -7.52^*^  (-7.63 to -7.42) | 2006-2011 | -6.95^*^  (-7.59 to -6.31) | 2011-2019 | -4.93^*^  (-5.20 to -4.65) | 2019-2021 | -2.70^*^  (-4.91 to -0.43) | ——————————— | | ——————————— | | -6.46^*^  (-6.65 to -6.28) |
| Mortality | 1990-2003 | -8.06^*^  (-8.22 to -7.91) | 2003-2006 | -9.29^*^  (-11.88 to -6.61) | 2006-2012 | -7.06^*^  (-7.63 to -6.48) | 2012-2021 | -3.06^*^  (-3.32 to -2.80) | ——————————— | | ——————————— | | -6.56^*^  (-6.85 to -6.28) |
| **Turkmenistan** |  |  |  |  |  |  |  |  |  |  |  |  |  |
| Incidence | 1990-1998 | -1.50^*^  (-1.61 to -1.39) | 1998-2001 | -3.01^*^  (-4.06 to -1.96) | 2001-2004 | -7.54^*^  (-8.51 to -6.56) | 2004-2010 | -8.58^*^  (-8.82 to -8.33) | 2010-2021 | -3.74^*^  (-3.84 to -3.64) | ——————————— | | -4.43^*^  (-4.58 to -4.29) |
| Prevalence | 1990-1998 | -2.01^*^  (-2.09 to -1.93) | 1998-2001 | -3.35^*^  (-4.09 to -2.60) | 2001-2004 | -8.23^*^  (-8.96 to -7.50) | 2004-2010 | -9.35^*^  (-9.56 to -9.14) | 2010-2021 | -3.45^*^  (-3.53 to -3.36) | ——————————— | | -4.71^*^  (-4.82 to -4.61) |
| DALY | 1990-1995 | 1.23  (-0.43 to 2.92) | 1995-1999 | -10.30^*^  (-13.47 to -7.01) | 1999-2004 | -18.18^*^  (-20.44 to -15.86) | 2004-2009 | -8.73^*^  (-11.41 to -5.97) | 2009-2014 | -20.27^*^  (-23.01 to -17.44) | 2014-2021 | -3.73^*^  (-5.32 to -2.12) | -9.90^*^  (-10.80 to -9.00) |
| Mortality | 1990-1995 | 1.70^*^  (0.02 to 3.42) | 1995-1999 | -10.65^*^  (-13.83 to -7.35) | 1999-2004 | -18.64^*^  (-20.90 to -16.31) | 2004-2009 | -8.51^*^  (-11.30 to -5.63) | 2009-2014 | -21.35^*^  (-24.16 to -18.43) | 2014-2021 | -3.38^*^  (-5.08 to -1.66) | -10.05^*^  (-10.97 to -9.12) |
| **United Arab Emirates** |  |  |  |  |  |  |  |  |  |  |  |  |  |
| Incidence | 1990-2000 | 1.39^*^  (1.35 to 1.44) | 2000-2004 | 0.44^*^  (0.14 to 0.74) | 2004-2011 | 1.37^*^  (1.26 to 1.48) | 2011-2015 | -2.30^*^  (-2.59 to -2.01) | 2015-2019 | -4.81^*^  (-5.07 to -4.54) | 2019-2021 | 1.77^*^  (1.24 to 2.30) | -0.01  (-0.08 to 0.06) |
| Prevalence | 1990-2000 | 1.32^*^  (1.27 to 1.36) | 2000-2004 | 0.35^*^  (0.05 to 0.65) | 2004-2011 | 1.34^*^  (1.22 to 1.45) | 2011-2015 | -2.54^*^  (-2.83 to -2.24) | 2015-2019 | -5.17^*^  (-5.44 to -4.90) | 2019-2021 | 1.64^*^  (1.10 to 2.18) | -0.14^*^  (-0.22 to -0.07) |
| DALY | 1990-1996 | -5.67^*^  (-6.20 to -5.13) | 1996-2001 | 1.96^*^  (1.00 to 2.93) | 2001-2005 | -0.49  (-1.95 to 0.99) | 2005-2011 | 1.32^*^  (0.59 to 2.05) | 2011-2017 | -3.46^*^  (-4.19 to -2.71) | 2017-2021 | -6.60^*^  (-7.63 to -5.57) | -2.16^*^  (-2.49 to -1.83) |
| Mortality | 1990-1996 | -8.13^*^  (-9.22 to -7.02) | 1996-2000 | 5.27^*^  (1.34 to 9.35) | 2000-2008 | 1.55^*^  (0.52 to 2.60) | 2008-2018 | -1.32^*^  (-2.01 to -0.63) | 2018-2021 | -12.32^*^  (-15.87 to -8.62) | ——————————— | | -2.26^*^  (-2.94 to -1.56) |
| **Uzbekistan** |  |  |  |  |  |  |  |  |  |  |  |  |  |
| Incidence | 1990-2001 | -5.82^*^  (-5.95 to -5.70) | 2001-2005 | -7.86^*^  (-8.96 to -6.75) | 2005-2011 | -1.85^*^  (-2.45 to -1.24) | 2011-2015 | -4.56^*^  (-5.95 to -3.15) | 2015-2019 | -9.35^*^  (-10.65 to -8.03) | 2019-2021 | -2.24  (-4.98 to 0.58) | -5.41^*^  (-5.74 to -5.08) |
| Prevalence | 1990-2001 | -6.29^*^  (-6.46 to -6.13) | 2001-2005 | -8.25^*^  (-9.84 to -6.63) | 2005-2011 | -2.02^*^  (-2.94 to -1.09) | 2011-2015 | -4.79^*^  (-6.92 to -2.62) | 2015-2019 | -9.53^*^  (-11.53 to -7.48) | 2019-2021 | -1.85  (-6.23 to 2.75) | -5.69^*^  (-6.20 to -5.18) |
| DALY | 1990-1995 | -0.52  (-3.64 to 2.70) | 1995-2000 | -22.74^*^  (-27.23 to -17.97) | 2000-2004 | -8.20  (-17.98 to 2.75) | 2004-2007 | -24.46^*^  (-40.10 to -4.74) | 2007-2021 | -8.41^*^  (-9.35 to -7.46) | ——————————— | | -11.33^*^  (-13.72 to -8.88) |
| Mortality | 1990-1995 | 0.11  (-3.21 to 3.55) | 1995-2000 | -22.85^*^  (-27.48 to -17.92) | 2000-2004 | -7.49  (-17.77 to 4.08) | 2004-2007 | -26.87^*^  (-43.37 to -5.57) | 2007-2021 | -8.50^*^  (-9.53 to -7.47) | ——————————— | | -11.50^*^  (-14.07 to -8.85) |
| **Viet Nam** |  |  |  |  |  |  |  |  |  |  |  |  |  |
| Incidence | 1990-2006 | -0.72^*^  (-0.91 to -0.53) | 2006-2009 | 7.63^*^  (2.18 to 13.36) | 2009-2021 | -0.37^*^  (-0.68 to -0.06) | ——————————— | | ——————————— | | ——————————— | | 0.19  (-0.31 to 0.70) |
| Prevalence | 1990-2006 | -1.02^*^  (-1.23 to -0.80) | 2006-2009 | 8.67^*^  (2.72 to 14.96) | 2009-2021 | -0.58^*^  (-0.92 to -0.24) | ——————————— | | ——————————— | | ——————————— | | 0.05  (-0.49 to 0.60) |
| DALY | 1990-1998 | -8.12^*^  (-8.66 to -7.57) | 1998-2005 | -6.74^*^  (-7.42 to -6.05) | 2005-2021 | -2.42^*^  (-2.58 to -2.25) | ——————————— | | ——————————— | | ——————————— | | -4.90^*^  (-5.12 to -4.68) |
| Mortality | 1990-1992 | -7.88^*^  (-9.12 to -6.63) | 1992-1997 | -9.03^*^  (-9.34 to -8.71) | 1997-2010 | -7.66^*^  (-7.71 to -7.60) | 2010-2015 | -5.33^*^  (-5.66 to -5.00) | 2015-2021 | -4.33^*^  (-4.53 to -4.13) | ——————————— | | -6.89^*^  (-7.00 to -6.78) |
| **Yemen** |  |  |  |  |  |  |  |  |  |  |  |  |  |
| Incidence | 1990-2000 | 0.51^*^  (0.40 to 0.63) | 2000-2005 | -0.52  (-1.04 to 0.01) | 2005-2010 | 7.29^*^  (6.76 to 7.83) | 2010-2013 | -4.33^*^  (-5.73 to -2.91) | 2013-2019 | -6.63^*^  (-6.93 to -6.33) | 2019-2021 | -14.73^*^  (-16.15 to 13.28) | -1.56^*^  (-1.76 to -1.35) |
| Prevalence | 1900-1999 | 0.04  (-0.12 to 0.20) | 1999-2005 | -1.19^*^  (-1.62 to -0.75) | 2005-2010 | 9.47^*^  (8.86 to 10.08) | 2010-2013 | -5.75^*^  (-7.32 to -4.15) | 2013-2019 | -8.47^*^  (-8.81 to -8.14) | 2019-2021 | -15.64^*^  (-17.37 to -13.88) | -2.12^*^  (-2.35 to -1.89) |
| DALY | 1990-1996 | -6.09^*^  (-7.26 to -4.91) | 1996-2014 | -9.02^*^  (-9.31 to -8.73) | 2014-2017 | 7.08  (-2.36 to 17.42) | 2017-2021 | -15.59^*^  (-18.04 to -13.07) | ——————————— | | ——————————— | | -7.90^*^  (-8.78 to -7.02) |
| Mortality | 1990-2000 | -6.79^*^  (-7.31 to -6.26) | 2000-2014 | -9.65^*^  (-10.07 to -9.22) | 2014-2017 | 8.25  (-1.38 to 18.83) | 2017-2021 | -12.76^*^  (-15.32 to -10.13) | ——————————— | | ——————————— | | -7.54^*^  (-8.43 to -6.65) |

DALY, disability-adjusted life years; APC, annual percentage change; AAPC, average annual percentage change; 95% CI, 95% confidence interval; ^*^, *P* < 0.05 .
