# Supplementary material for: The burden and etiologies of diarrhea in Asia and its countries from 1990 to 2021 and the forecast to 2040: analyses informed by the global burden of disease study 2021
Source: Front Public Health. 2025 Aug 6;13:1651315. doi: 10.3389/fpubh.2025.1651315 (PMC12364947; doi:10.3389/fpubh.2025.1651315)
Supplement: Supplementary file 4 [file Table_3.DOCX]

**Table S3** Prediction of diarrheal diseases burden in Asia and Asian countries from 2022 to 2030.

|  | **Age-standardized rate per 100,000 No. (95%UI)** | | | | | | | | |
| --- | --- | --- | --- | --- | --- | --- | --- | --- | --- |
|  | **2022** | **2023** | **2024** | **2025** | **2026** | **2027** | **2028** | **2029** | **2030** |
| **Asia** |  |  |  |  |  |  |  |  |  |
| Incidence | 59,437.23 (56,980.10 to 61,894.37) | 59,425.38 (55,462.91 to 63,387.85) | 59,442.34 (53,552.79 to 65,331.88) | 59,460.75 (51,332.55 to 67,588.96) | 59,419.30 (48,797.81 to 70,040.80) | 59,310.98 (45,973.14 to 72,648.83) | 59,237.77 (42,972.86 to 75,502.67) | 59,232.94 (39,835.16 to 78,630.72) | 59,276.54 (36,550.12 to 82,002.95) |
| Prevalence | 902.14 (871.24  to  933.04) | 901.07 (858.63  to 943.51) | 899.97 (842.32  to 957.62) | 898.75 (823.16  to 974.34) | 897.03 (801.32  to 992.74) | 894.80 (777.10  to 1,012.50) | 892.87 (751.53  to 1,034.21) | 891.58 (725.07 to 1,058.10) | 890.86 (697.69 to 1,084.03) |
| DALY | 547.26 (513.03  to  581.48) | 511.25 (473.22  to  549.29) | 478.09 (434.74 to  521.44) | 447.58 (398.14  to  497.02) | 419.46 (363.64  to  475.27) | 393.49 (331.32  to 455.67) | 369.52 (301.20  to 437.85) | 347.40 (273.25  to 421.56) | 327.01 (247.37  to 406.64) |
| Mortality | 14.43 (13.85  to 15.01) | 13.58 (12.85 to 14.30) | 12.77 (11.87 to  13.67) | 12.01 (10.91 to 13.11) | 11.30 (10.00 to 12.60) | 10.63 (9.13 to 12.13) | 9.99  (8.31 to 11.68) | 9.39  (7.53  to  11.26) | 8.84  (6.80  to  10.87) |
| **Afghanistan** |  |  |  |  |  |  |  |  |  |
| Incidence | 18,843.93 (17,875.73  to  19,812.13) | 17,622.98 (16,477.72  to  18,768.24) | 16,489.07 (15,106.02  to  17,872.13) | 15,433.82 (13,784.60  to  17,083.04) | 14,447.71 (12,524.06  to  16,371.36) | 13,528.33 (11,333.49  to  15,723.18) | 12,680.31 (10,224.33  to  15,136.30) | 11,900.07 (9,196.55  to  14,603.59) | 11,179.82 (8,244.34  to  14,115.30) |
| Prevalence | 273.28 (256.28  to  290.28) | 254.70 (235.11  to  274.29) | 237.52 (214.49  to  260.54) | 221.59 (194.74  to  248.44) | 206.79 (176.01  to  237.56) | 193.06 (158.41  to  227.70) | 180.43 (142.09  to  218.77) | 168.84 (127.02  to  210.66) | 158.18 (113.13  to  203.23) |
| DALY | 661.43 (524.84  to  798.02) | 592.58 (444.50  to  740.65) | 531.01 (364.77  to  697.24) | 475.94 (289.18  to  662.71) | 426.70 (219.85  to  633.54) | 382.65 (157.66  to  607.64) | 343.25 (102.77  to  583.74) | 308.01 (54.94  to  561.07) | 276.47 (13.75  to  539.19) |
| Mortality | 7.30  (6.55  to  8.05) | 6.54  (5.53  to  7.54) | 5.86  (4.58  to  7.14) | 5.25  (3.72  to  6.78) | 4.71  (2.94  to  6.47) | 4.22  (2.26  to  6.17) | 3.78  (1.66  to  5.90) | 3.39  (1.14  to  5.63) | 3.04  (0.70  to  5.38) |
| **Armenia** |  |  |  |  |  |  |  |  |  |
| Incidence | 7,340.49 (6,533.96  to  8,147.03) | 6,963.53 (5,779.03  to  8,148.03) | 6,605.00 (4,956.89  to  8,253.12) | 6,264.79 (4,116.48  to  8,413.11) | 5,942.29 (3,281.70  to  8,602.88) | 5,636.55 (2,465.76  to  8,807.34) | 5,346.78 (1,677.04  to  9,016.51) | 5,072.38 (920.94  to  9,223.83) | 4,812.78 (200.78  to  9,424.77) |
| Prevalence | 110.94 (97.88  to  124.00) | 106.88 (84.74  to  129.02) | 102.95 (70.20  to  135.71) | 99.17 (54.96  to  143.38) | 95.52 (39.37  to  151.66) | 91.99 (23.66  to  160.32) | 88.59  (8.01  to  169.17) | 85.33  (-7.45  to  178.11) | 82.19  (-22.64  to  187.03) |
| DALY | 29.72 (21.13  to  38.30) | 27.64 (17.54  to  37.73) | 25.73  (13.61  to  37.85) | 23.98  (9.59  to  38.37) | 22.37  (5.66  to  39.08) | 20.89  (1.89  to  39.89) | 19.53  (-1.66  to  40.73) | 18.28  (-4.99  to  41.55) | 17.13  (-8.08  to  42.33) |
| Mortality | 0.15  (0.04  to  0.25) | 0.13  (0.02  to  0.25) | 0.12  (-0.01  to  0.25) | 0.11  (-0.03  to  0.24) | 0.10  (-0.04  to  0.24) | 0.09  (-0.06  to  0.24) | 0.08  (-0.07  to  0.23) | 0.07  (-0.08  to  0.23) | 0.07  (-0.09  to  0.22) |
| **Bahrain** |  |  |  |  |  |  |  |  |  |
| Incidence | 23,877.12 (22,961.00  to  24,793.25) | 23,971.22 (22,407.32  to  25,535.12) | 24,056.92 (21,670.48  to  26,443.37) | 24,136.53 (20,793.56  to  27,479.50) | 24,212.19 (19,797.27  to  28,627.12) | 24,285.48 (18,693.53  to  29,877.44) | 24,359.01 (17,492.90  to  31,225.12) | 24,434.26 (16,202.40  to  32,666.13) | 24,513.02 (14,827.35  to  34,198.69) |
| Prevalence | 355.99 (335.30  to  376.67) | 360.72 (325.64  to  395.80) | 365.33 (312.42  to  418.23) | 369.86 (296.22  to  443.49) | 374.33 (277.32  to  471.35) | 378.79 (255.87  to  501.71) | 383.25 (231.99  to  534.51) | 387.76 (205.77  to  569.74) | 392.34 (177.26  to  607.42) |
| DALY | 75.03 (66.96  to  83.10) | 74.05 (63.14  to  84.96) | 73.14 (58.77  to  87.50) | 72.31 (54.04  to  90.57) | 71.55 (49.03  to  94.08) | 70.88 (43.79  to  97.98) | 70.30 (38.36  to  102.24) | 69.81 (32.76  to  106.86) | 69.40 (26.98  to  111.82) |
| Mortality | 1.33  (0.42  to  2.23) | 1.46  (0.34  to  2.59) | 1.61  (0.21  to  3.01) | 1.78  (0.05  to  3.50) | 1.96  (-0.15  to  4.07) | 2.16  (-0.40  to  4.73) | 2.38  (-0.72  to  5.48) | 2.62  (-1.09  to  6.33) | 2.87  (-1.55  to  7.29) |
| **Bangladesh** |  |  |  |  |  |  |  |  |  |
| Incidence | 49,623.17  (46,964.22  to  52,282.13) | 47,343.40 (44,319.84  to  50,366.97) | 45,161.38  (41,633.12  to  48,689.64) | 43,071.13 (38,949.84  to  47,192.41) | 41,075.48 (36,309.20  to  45,841.76) | 39,200.63 (33,759.28  to  44,641.98) | 37,482.86 (31,353.02  to  43,612.69) | 35,920.46 (29,097.35  to  42,743.56) | 34,500.27 (26,982.20  to  42,018.34) |
| Prevalence | 690.14 (650.51  to  729.77) | 657.36 (612.13  to  702.59) | 625.93 (572.93  to  678.94) | 595.79 (533.67  to  657.90) | 567.01 (495.05  to  638.97) | 539.96 (457.75  to  622.16) | 515.14 (422.53  to  607.75) | 492.55 (389.50  to  595.60) | 472.03 (358.56  to  585.51) |
| DALY | 668.42  (619.15  to  717.69) | 638.03 (582.06  to  694.00) | 609.40 (544.25  to  674.55) | 582.47 (506.56  to  658.38) | 557.10 (469.52  to  644.68) | 533.18 (433.48  to  632.88) | 510.70 (398.73  to  622.67) | 489.64 (365.42  to  613.85) | 469.94 (333.61  to  606.27) |
| Mortality | 26.40 (24.35  to  28.44) | 25.59 (23.22  to  27.97) | 24.81 (21.99  to  27.63) | 24.05 (20.71  to  27.39) | 23.31 (19.40  to  27.23) | 22.60 (18.07  to  27.13) | 21.90 (16.74  to  27.06) | 21.23 (15.43  to  27.03) | 20.59  (14.14  to  27.04) |
| **Bhutan** |  |  |  |  |  |  |  |  |  |
| Incidence | 55,897.09 (53,864.10  to  57,930.08) | 56,392.20 (53,195.23  to  59,589.18) | 56,897.53 (52,198.36  to  61,596.70) | 57,405.88 (50,943.25  to  63,868.51) | 57,916.28 (49,459.88  to  66,372.68) | 58,401.53 (47,734.86  to  69,068.21) | 58,935.07 (45,846.91  to  72,023.24) | 59,541.60 (43,822.19  to  75,261.01) | 60,228.96 (41,663.53  to  78,794.39) |
| Prevalence | 793.81 (754.40  to  833.21) | 809.04 (746.64  to  871.43) | 824.49 (733.28  to  915.69) | 840.26 (715.33  to  965.19) | 856.34 (693.04  to  1,019.64) | 872.48 (666.27  to  1,078.69) | 889.45 (635.73  to  1,143.18) | 907.73 (601.71  to  1,213.75) | 927.58 (564.17  to  1,290.98) |
| DALY | 918.41 (852.45  to  984.38) | 881.81 (794.52  to  969.09) | 847.49 (733.55  to  961.43) | 815.38 (671.80  to  958.97) | 785.32 (610.38  to  960.25) | 757.11 (549.85  to  964.38) | 730.60 (490.53  to  970.67) | 705.69 (432.64  to  978.74) | 682.35 (376.30  to  988.40) |
| Mortality | 31.81 (27.78  to  35.85) | 30.83 (25.80  to  35.85) | 29.87 (23.75  to  35.99) | 28.94 (21.67  to  36.22) | 28.04 (19.57  to  36.51) | 27.16 (17.48  to  36.84) | 26.31 (15.42  to  37.21) | 25.49 (13.39  to  37.60) | 24.70  (11.40  to  38.00) |
| **Brunei Darussalam** |  |  |  |  |  |  |  |  |  |
| Incidence | 5,684.13 (5,518.88  to  5,849.38) | 5,593.12 (5,312.90  to  5,873.34) | 5,501.97 (5,085.25  to  5,918.69) | 5,410.69 (4,842.26  to  5,979.11) | 5,318.54 (4,586.74  to  6,050.34) | 5,225.44 (4,321.06  to  6,129.82) | 5,132.36 (4,048.27  to  6,216.46) | 5,039.94 (3,770.56  to  6,309.32) | 4,948.16 (3,489.25  to  6,407.06) |
| Prevalence | 81.33 (73.66  to  88.99) | 78.44 (68.84  to  88.04) | 75.63 (63.90  to  87.37) | 72.90 (58.92  to  86.88) | 70.25 (53.98  to  86.52) | 67.67 (49.10  to  86.24) | 65.17 (44.32  to  86.02) | 62.75 (39.66  to  85.83) | 60.40 (35.13  to  85.66) |
| DALY | 35.18 (30.42  to  39.95) | 34.44 (28.82  to  40.07) | 33.72 (27.14  to  40.31) | 33.02 (25.39  to  40.64) | 32.33 (23.61  to  41.05) | 31.65 (21.80  to  41.51) | 31.00 (19.98  to  42.02) | 30.35 (18.15  to  42.56) | 29.73 (16.32  to  43.13) |
| Mortality | 2.50*10^-8^  (-2.82*10^-5^  to  2.83*10^-5^) | 2.50*10^-8^  (-2.82*10^-5^  to  2.83*10^-5^) | 2.50*10^-8^  (-2.82*10^-5^  to  2.83*10^-5^) | 2.50*10^-8^  (-2.82*10^-5^  to  2.83*10^-5^) | 2.50*10^-8^  (-2.82*10^-5^  to  2.83*10^-5^) | 2.50*10^-8^  (-2.82*10^-5^  to  2.83*10^-5^) | 2.50*10^-8^  (-2.82*10^-5^  to  2.83*10^-5^) | 2.50*10^-8^  (-2.82*10^-5^  to  2.83*10^-5^) | 2.50*10^-8^  (-2.82*10^-5^  to  2.83*10^-5^) |
| **Cambodia** |  |  |  |  |  |  |  |  |  |
| Incidence | 40,522.84 (39,311.41  to  41,734.26) | 40,410.23 (38,570.43  to  42,250.03) | 40,332.06 (37,674.54  to  42,989.58) | 40,290.00 (36,680.01  to  43,899.99) | 40,266.80 (35,594.17  to  44,939.42) | 40,251.21 (34,416.92  to  46,085.50) | 40,284.39 (33,194.93  to  47,373.86) | 40,382.89 (31,945.53  to  48,820.25) | 40,549.27 (30,668.81  to  50,429.74) |
| Prevalence | 578.18 (555.53  to  600.84) | 574.76  (541.95  to  607.56) | 571.85 (525.90  to  617.80) | 569.50 (508.29  to  630.70) | 567.52 (489.36  to  645.69) | 565.84 (469.23  to  662.45) | 564.93 (448.48  to  681.38) | 565.01 (427.34  to  702.68) | 566.12 (405.81  to  726.43) |
| DALY | 377.72 (347.75  to  407.69) | 355.87 (321.53  to  390.22) | 335.80 (295.26  to  376.34) | 317.33 (269.57  to  365.10) | 300.33 (244.84  to  355.82) | 284.67 (221.28  to  348.06) | 270.23 (198.95  to  341.50) | 256.90 (177.87  to  335.93) | 244.58 (157.99  to  331.18) |
| Mortality | 10.94 (10.23  to  11.66) | 10.40 (9.45  to  11.35) | 9.88  (8.68  to  11.08) | 9.39  (7.92  to  10.86) | 8.92  (7.19  to  10.65) | 8.48  (6.49  to  10.47) | 8.06  (5.82  to  10.30) | 7.66  (5.18  to  10.14) | 7.28  (4.57  to  9.99) |
| **China** |  |  |  |  |  |  |  |  |  |
| Incidence | 5,376.17 (4,873.86  to  5,878.48) | 5,188.94 (4,571.09  to  5,806.79) | 5,007.70 (4,237.19  to  5,778.21) | 4,832.43 (3,886.37  to  5,778.49) | 4,662.97 (3,527.98  to  5,797.96) | 4,499.05 (3,167.87  to  5,830.22) | 4,340.57 (2,810.10  to  5,871.03) | 4,187.45 (2,457.46  to  5,917.44) | 4,039.60 (2,111.87  to  5,967.33) |
| Prevalence | 78.92 (71.47  to  86.36) | 76.11 (66.72  to  85.51) | 73.40 (61.44  to  85.36) | 70.77 (55.88  to  85.66) | 68.24 (50.21  to  86.27) | 65.79 (44.51  to  87.07) | 63.43 (38.86  to  88.00) | 61.14 (33.28  to  89.00) | 58.94 (27.83  to  90.06) |
| DALY | 21.11 (18.46  to  23.76) | 19.45 (16.48  to  22.43) | 17.97 (14.49  to  21.46) | 16.65 (12.58  to  20.73) | 15.47 (10.77  to  20.17) | 14.40  (9.08  to  19.73) | 13.45 (7.52  to  19.38) | 12.59  (6.08  to  19.11) | 11.82  (4.75  to  18.90) |
| Mortality | 0.28  (0.27  to  0.30) | 0.27  (0.24  to  0.29) | 0.25  (0.22  to  0.29) | 0.24  (0.19  to  0.29) | 0.23  (0.17  to  0.28) | 0.22  (0.15  to  0.28) | 0.21  (0.13  to  0.28) | 0.20  (0.11  to  0.28) | 0.19  (0.09  to  0.28) |
| **Cyprus** |  |  |  |  |  |  |  |  |  |
| Incidence | 34,487.84 (32,582.43  to  36,393.24) | 33,815.71 (30,551.86  to  37,079.56) | 33,151.04 (28,202.54  to  38,099.54) | 32,494.45 (25,650.27  to  39,338.62) | 31,845.51 (22,952.75  to  40,738.27) | 31,203.77 (20,147.03  to  42,260.51) | 30,570.01 (17,261.83  to  43,878.19) | 29,945.24 (14,319.79  to  45,570.69) | 29,329.61 (11,338.89  to  47,320.33) |
| Prevalence | 564.51 (532.06  to  596.97) | 549.91 (494.58  to  605.24) | 535.59 (453.52  to  617.66) | 521.55 (410.27  to  632.83) | 507.78 (365.59  to  649.97) | 494.25 (319.97  to  668.53) | 480.99 (273.85  to  688.13) | 468.02 (227.58  to  708.46) | 455.33 (181.40  to  729.27) |
| DALY | 98.39 (92.07  to  104.71) | 93.78 (85.37  to  102.18) | 89.39 (78.66  to  100.11) | 85.21 (72.06  to  98.36) | 81.24 (65.63  to  96.84) | 77.46 (59.41  to  95.50) | 73.86 (53.43  to  94.29) | 70.44 (47.71  to  93.18) | 67.20 (42.24  to  92.15) |
| Mortality | 2.54  (2.02  to  3.06) | 2.52  (1.93  to  3.10) | 2.49  (1.84  to  3.15) | 2.47  (1.73  to  3.21) | 2.45  (1.62  to  3.27) | 2.42  (1.50  to  3.35) | 2.40  (1.38  to  3.42) | 2.38  (1.26  to  3.51) | 2.36  (1.13  to  3.59) |
| **Democratic People's Republic of Korea** |  |  |  |  |  |  |  |  |  |
| Incidence | 56,405.65 (51,131.55  to  61,679.76) | 54,008.38 (45,988.62  to  62,028.15) | 51,734.61 (40,370.76  to  63,098.45) | 49,572.08 (34,574.34  to  64,569.83) | 47,508.12 (28,739.78  to  66,276.47) | 45,549.15 (22,954.41  to  68,143.89) | 43,694.08 (17,271.18  to  70,116.99) | 41,933.16 (11,720.78  to  72,145.55) | 40,254.04 (6,323.95  to  74,184.13) |
| Prevalence | 916.27 (816.70  to  1,015.83) | 877.31 (729.34  to  1,025.28) | 840.44 (633.40  to  1,047.47) | 805.43 (534.10  to  1,076.76) | 772.07 (433.98  to  1,110.15) | 740.43 (334.57  to  1,146.28) | 710.49 (236.81  to  1,184.16) | 682.07 (141.24  to  1,222.91) | 654.98 (48.23  to  1,261.72) |
| DALY | 124.79 (109.76  to  139.82) | 120.32 (100.27  to  140.36) | 116.15 (89.66  to  142.64) | 112.26 (78.48  to  146.04) | 108.60 (67.03  to  150.18) | 105.19 (55.49  to  154.88) | 102.00 (43.98  to  160.02) | 99.00 (32.52  to  165.47) | 96.16 (21.17  to  171.16) |
| Mortality | 0.39  (0.34  to  0.45) | 0.38  (0.32  to  0.45) | 0.37  (0.30  to  0.45) | 0.36  (0.28  to  0.45) | 0.36  (0.26  to  0.45) | 0.35  (0.24  to  0.45) | 0.34  (0.22  to  0.46) | 0.33  (0.21  to  0.46) | 0.33  (0.19  to  0.46) |
| **Georgia** |  |  |  |  |  |  |  |  |  |
| Incidence | 9,329.73 (8,469.35  to  10,190.11) | 8,598.95 (7,355.48  to  9,842.43) | 7,924.01 (6,231.70  to  9,616.32) | 7,301.26 (5,151.38  to  9,451.14) | 6,726.67 (4,136.36  to  9,316.98) | 6,196.49 (3,195.86  to  9,197.12) | 5,707.83 (2,333.77  to  9,081.88) | 5,257.82 (1,550.39  to  8,965.25) | 4,843.52 (843.81  to  8,843.23) |
| Prevalence | 143.30 (129.17  to  157.43) | 132.29 (110.76  to  153.83) | 122.12 (92.40  to  151.84) | 112.71 (74.83  to  150.60) | 104.01 (58.33  to  149.69) | 95.96 (43.03  to  148.89) | 88.53 (29.00  to  148.05) | 81.67 (16.24  to  147.10) | 75.35  (4.73  to  145.96) |
| DALY | 23.23 (18.03  to  28.43) | 20.54 (14.66  to  26.43) | 18.20 (11.43  to  24.97) | 16.15 (8.48  to  23.82) | 14.36 (5.86  to  22.86) | 12.79 (3.57  to  22.01) | 11.41 (1.60  to  21.22) | 10.20  (-0.08  to  20.48) | 9.14  (-1.49  to  19.77) |
| Mortality | 0.09  (0.02  to  0.16) | 0.08  (0.00  to  0.15) | 0.07  (-0.01  to  0.14) | 0.06  (-0.02  to  0.14) | 0.05  (-0.03  to  0.13) | 0.04  (-0.04  to  0.12) | 0.04  (-0.04  to  0.12) | 0.03  (-0.05  to  0.11) | 0.03  (-0.05  to  0.11) |
| **India** |  |  |  |  |  |  |  |  |  |
| Incidence | 132,572.80 (128,663.22  to  136,482.30) | 134,901.80 (128,387.58  to  141,416.00) | 137,271.10 (127,376.43  to  147,165.80) | 139,638.50 (125,729.21  to  153,547.70) | 141,929.10 (123,430.93  to  160,427.40) | 144,147.40 (120,507.82  to  167,786.90) | 146,460.30 (117,132.44  to  175,788.20) | 148,923.20 (113,354.38  to  184,491.90) | 151,512.30 (109,145.88  to  193,878.60) |
| Prevalence | 2,049.59 (1,993.93  to  2,105.25) | 2,090.52 (2,002.48  to  2,178.56) | 2,131.67 (2,000.99  to  2,262.35) | 2,172.80 (1,991.26  to  2,354.35) | 2,213.37 (1,973.57  to  2,453.18) | 2,253.40 (1,948.19  to  2,558.61) | 2,294.71 (1,917.06  to  2,672.35) | 2,338.04 (1,880.90  to  2,795.18) | 2,383.38 (1,839.55  to  2,927.21) |
| DALY | 1,352.06 (1,290.72  to  1,413.40) | 1,276.26 (1,195.80  to  1,356.73) | 1,205.63 (1,101.28  to  1,309.98) | 1,140.02 (1,009.64  to  1,270.40) | 1,079.15 (921.97  to  1,236.34) | 1,022.54 (838.54  to  1,206.53) | 969.61 (759.35  to  1,179.88) | 920.11 (684.42  to  1,155.79) | 873.96 (613.82  to  1,134.09) |
| Mortality | 48.07 (46.21  to  49.93) | 45.25 (42.77  to  47.73) | 42.58 (39.33  to  45.83) | 40.07 (36.00  to  44.14) | 37.71 (32.80  to  42.62) | 35.48 (29.75  to  41.22) | 33.38 (26.85  to  39.90) | 31.39 (24.13  to  38.66) | 29.53 (21.57  to  37.49) |
| **Indonesia** |  |  |  |  |  |  |  |  |  |
| Incidence | 61,369.28 (59,229.04  to  63,509.53) | 61,564.89 (58,677.80  to  64,451.99) | 61,740.07 (57,851.21  to  65,628.93) | 61,898.77 (56,812.53  to  66,985.02) | 62,044.11 (55,601.08  to  68,487.14) | 62,175.70 (54,238.69  to  70,112.71) | 62,293.99 (52,741.82  to  71,846.16) | 62,401.43 (51,123.51  to  73,679.34) | 62,500.70 (49,393.72  to  75,607.69) |
| Prevalence | 935.76 (902.13  to  969.38) | 934.75 (890.01  to  979.50) | 933.41 (873.81  to  993.01) | 931.80 (854.52  to  1,009.07) | 929.97 (832.78  to  1,027.16) | 927.94 (808.95  to  1,046.93) | 925.70 (783.30  to  1,068.10) | 923.30 (756.04  to  1,090.55) | 920.78 (727.37  to  1,114.19) |
| DALY | 895.25 (858.58  to  931.93) | 860.20 (812.66  to  907.75) | 826.83 (764.90  to  888.76) | 795.03 (716.72  to  873.33) | 764.75 (668.96  to  860.53) | 735.96 (622.11  to  849.80) | 708.56 (576.42  to  840.69) | 682.43 (532.02  to  832.85) | 657.51 (488.98  to  826.04) |
| Mortality | 30.67 (29.97  to  31.38) | 29.78 (28.60  to  30.96) | 28.91 (27.18  to  30.63) | 28.06 (25.73  to  30.38) | 27.22 (24.27  to  30.18) | 26.41 (22.80  to  30.01) | 25.62 (21.35  to  29.89) | 24.85 (19.91  to  29.79) | 24.10 (18.49  to  29.71) |
| **Iran**  **(Islamic Republic of)** |  |  |  |  |  |  |  |  |  |
| Incidence | 20,465.49 (18,826.38  to  22,104.60) | 19,879.39 (18,062.72  to  21,696.07) | 19,323.02 (17,247.50  to  21,398.54) | 18,796.59 (16,399.47  to  21,193.71) | 18,296.18 (15,531.80  to  21,060.55) | 17,824.55 (14,659.69  to  20,989.41) | 17,391.07 (13,800.73  to  20,981.41) | 16,997.37 (12,961.68  to  21,033.05) | 16,639.70 (12,142.23  to  21,137.17) |
| Prevalence | 300.74 (275.88  to  325.61) | 291.77 (264.23  to  319.32) | 283.26 (251.81  to  314.71) | 275.19 (238.90  to  311.49) | 267.53 (225.71  to  309.34) | 260.31 (212.48  to  308.14) | 253.68 (199.48  to  307.89) | 247.67 (186.80  to  308.55) | 242.23 (174.44  to  310.01) |
| DALY | 60.97 (56.57  to  65.37) | 56.80 (52.00  to  61.59) | 53.19 (47.77  to  58.62) | 50.07 (43.88  to  56.25) | 47.33 (40.29  to  54.37) | 44.96 (37.02  to  52.90) | 42.92 (34.03  to  51.80) | 41.17 (31.31  to  51.03) | 39.66 (28.80  to  50.52) |
| Mortality | 0.87  (0.79  to  0.94) | 0.82  (0.72  to  0.91) | 0.77  (0.65  to  0.89) | 0.73  (0.59  to  0.88) | 0.70  (0.53  to  0.86) | 0.66  (0.47  to  0.85) | 0.63  (0.42  to  0.85) | 0.60  (0.37  to  0.84) | 0.58  (0.32  to  0.84) |
| **Iraq** |  |  |  |  |  |  |  |  |  |
| Incidence | 24,442.20 (23,349.46  to  25,534.94) | 23,512.64 (22,081.08  to  24,944.19) | 22,628.00 (20,753.70  to  24,502.30) | 21,784.88 (19,409.65  to  24,160.11) | 20,981.62 (18,072.32  to  23,890.92) | 20,221.51 (16,758.44  to  23,684.58) | 19,512.71 (15,485.02  to  23,540.39) | 18,854.38 (14,256.69  to  23,452.07) | 18,242.03 (13,072.18  to  23,411.87) |
| Prevalence | 348.92 (330.02  to  367.81) | 333.86 (309.91  to  357.82) | 319.58 (289.07  to  350.09) | 306.01 (268.12  to  343.91) | 293.12 (247.42  to  338.82) | 280.94 (227.23  to  334.64) | 269.58 (207.79  to  331.37) | 259.04 (189.18  to  328.90) | 249.26 (171.38  to  327.13) |
| DALY | 159.14 (141.85  to  176.44) | 150.12 (131.80  to  168.44) | 141.71 (121.79  to  161.64) | 133.88 (111.97  to  155.80) | 126.59 (102.45  to  150.73) | 119.81 (93.34  to  146.28) | 113.51 (84.69  to  142.32) | 107.64 (76.53  to  138.76) | 102.19 (68.84  to  135.55) |
| Mortality | 2.25  (2.06  to  2.45) | 2.19  (1.93  to  2.44) | 2.12  (1.80  to  2.45) | 2.06  (1.67  to  2.46) | 2.00  (1.53  to  2.48) | 1.95  (1.39  to  2.50) | 1.90  (1.26  to  2.53) | 1.84  (1.13  to  2.56) | 1.80  (0.99  to  2.60) |
| **Israel** |  |  |  |  |  |  |  |  |  |
| Incidence | 22,435.30 (20,995.80  to  23,874.80) | 21,752.55 (19,510.01  to  23,995.09) | 21,089.28 (17,854.57  to  24,323.98) | 20,444.42 (16,109.78  to  24,779.06) | 19,815.60 (14,315.06  to  25,316.13) | 19,201.93 (12,495.31  to  25,908.56) | 18,603.96 (10,669.33  to  26,538.60) | 18,022.03 (8,851.09  to  27,192.98) | 17,455.48 (7,050.96  to  27,860.00) |
| Prevalence | 358.46 (333.79  to  383.14) | 346.64 (309.57  to  383.72) | 335.18 (282.86  to  387.51) | 324.06 (254.91  to  393.21) | 313.25 (226.37  to  400.13) | 302.72 (197.61  to  407.83) | 292.49 (168.94  to  416.04) | 282.54 (140.55  to  424.53) | 272.88 (112.62  to  433.14) |
| DALY | 80.80 (75.70  to  85.90) | 77.08 (68.80  to  85.35) | 73.50 (61.57  to  85.43) | 70.08 (54.27  to  85.89) | 66.80 (47.02  to  86.57) | 63.66 (39.92  to  87.39) | 60.65 (33.02  to  88.28) | 57.77 (26.37  to  89.17) | 55.01 (19.98  to  90.03) |
| Mortality | 2.23  (1.93  to  2.53) | 2.14  (1.72  to  2.57) | 2.06  (1.50  to  2.62) | 1.98  (1.27  to  2.69) | 1.91  (1.05  to  2.76) | 1.83  (0.82  to  2.84) | 1.76  (0.60  to  2.93) | 1.70  (0.39  to  3.01) | 1.63  (0.18  to  3.09) |
| **Japan** |  |  |  |  |  |  |  |  |  |
| Incidence | 50,771.18 (48,406.19  to  53,136.16) | 50,169.13 (46,016.82  to  54,321.45) | 49,558.91 (43,172.48  to  55,945.34) | 48,941.30 (40,012.54  to  57,870.06) | 48,317.25 (36,607.68  to  60,026.83) | 47,688.76 (33,004.72  to  62,372.80) | 47,058.19 (29,239.97  to  64,876.41) | 46,426.16 (25,341.34  to  67,510.98) | 45,792.97 (21,332.01  to  70,253.94) |
| Prevalence | 805.54 (771.06  to  840.01) | 798.15 (742.58  to  853.72) | 790.51 (707.95  to  873.06) | 782.63 (669.06  to  896.21) | 774.56 (626.86  to  922.27) | 766.35 (581.97  to  950.72) | 758.00 (534.83  to  981.16) | 749.53 (485.80  to  1,013.25) | 740.95 (435.16  to  1,046.73) |
| DALY | 105.97 (100.01  to  111.93) | 104.35 (95.60  to  113.11) | 102.71 (90.35  to  115.07) | 101.05 (84.55  to  117.55) | 99.38 (78.36  to  120.40) | 97.70 (71.88  to  123.52) | 96.02 (65.17  to  126.87) | 94.34 (58.29  to  130.38) | 92.67 (51.30  to  134.03) |
| Mortality | 0.68  (0.63  to  0.74) | 0.68  (0.57  to  0.78) | 0.67  (0.51  to  0.83) | 0.66  (0.44  to  0.88) | 0.65  (0.37  to  0.94) | 0.65  (0.29  to  1.00) | 0.64  (0.21  to  1.07) | 0.63  (0.13  to  1.14) | 0.63  (0.04  to  1.22) |
| **Jordan** |  |  |  |  |  |  |  |  |  |
| Incidence | 19,790.66 (18,435.77  to  21,145.54) | 19,503.29 (17,161.09  to  21,845.50) | 19,212.75 (15,642.17  to  22,783.34) | 18,922.31 (13,962.00  to  23,882.62) | 18,629.02 (12,157.81  to  25,100.22) | 18,333.00 (10,255.42  to  26,410.58) | 18,041.49 (8,278.87  to  27,804.10) | 17,761.48 (6,245.28  to  29,277.69) | 17,494.90 (4,164.52  to  30,825.29) |
| Prevalence | 277.38 (252.34  to  302.42) | 273.87 (230.85  to  316.89) | 270.28 (205.05  to  335.50) | 266.63 (176.33  to  356.94) | 262.91 (145.33  to  380.49) | 259.12 (112.52  to  405.72) | 255.36 (78.28  to  432.44) | 251.72 (42.89  to  460.54) | 248.21 (6.53  to  489.90) |
| DALY | 64.50 (56.34  to  72.66) | 60.79 (51.68  to  69.89) | 57.30 (46.86  to  67.74) | 54.03 (42.02  to  66.05) | 50.97 (37.27  to  64.67) | 48.09 (32.69  to  63.49) | 45.39 (28.33  to  62.45) | 42.86 (24.21  to  61.51) | 40.49 (20.34  to  60.63) |
| Mortality | 0.84  (0.69  to  0.98) | 0.82  (0.65  to  0.98) | 0.80  (0.60  to  0.99) | 0.78  (0.56  to  1.00) | 0.76  (0.52  to  1.01) | 0.75  (0.47  to  1.02) | 0.73  (0.43  to  1.04) | 0.72  (0.39  to  1.05) | 0.71  (0.34  to  1.07) |
| **Kazakhstan** |  |  |  |  |  |  |  |  |  |
| Incidence | 7,670.18 (7,039.04  to  8,301.33) | 7,242.47 (6,495.19  to  7,989.75) | 6,842.06 (5,938.24  to  7,745.88) | 6,467.57 (5,385.91  to  7,549.22) | 6,117.60 (4,848.99  to  7,386.22) | 5,790.79 (4,333.33  to  7,248.25) | 5,485.98 (3,842.38  to  7,129.59) | 5,201.64 (3,377.30  to  7,025.98) | 4,936.15 (2,938.02  to  6,934.29) |
| Prevalence | 120.50 (109.95  to  131.05) | 114.92 (102.65  to  127.18) | 109.67 (95.10  to  124.24) | 104.75 (87.52  to  121.98) | 100.14 (80.05  to  120.22) | 95.81 (72.77  to  118.86) | 91.78 (65.75  to  117.81) | 88.01 (59.00  to  117.01) | 84.48 (52.54  to  116.43) |
| DALY | 27.09 (20.70  to  33.47) | 24.71 (17.19  to  32.23) | 22.70 (13.53  to  31.88) | 21.00  (9.94  to  32.07) | 19.56  (6.51  to  32.61) | 18.34  (3.26  to  33.42) | 17.30  (0.17  to  34.43) | 16.41  (-2.79  to  35.61) | 15.65  (-5.65  to  36.95) |
| Mortality | 0.23  (0.15  to  0.32) | 0.22  (0.11  to  0.34) | 0.21  (0.06  to  0.36) | 0.20  (0.02  to  0.39) | 0.19  (-0.03  to  0.42) | 0.19  (-0.08  to  0.45) | 0.18  (-0.12  to  0.48) | 0.17  (-0.17  to  0.50) | 0.16  (-0.21  to  0.53) |
| **Kuwait** |  |  |  |  |  |  |  |  |  |
| Incidence | 23,237.21 (21,854.21  to  24,620.21) | 21,818.05 (20,051.98  to  23,584.11) | 20,481.11 (18,227.75  to  22,734.47) | 19,223.02 (16,440.43  to  22,005.61) | 18,041.93 (14,723.09  to  21,360.77) | 16,935.95 (13,092.63  to  20,779.26) | 15,901.52 (11,556.82  to  20,246.22) | 14,933.96 (10,117.45  to  19,750.47) | 14,028.76 (8,773.54  to  19,283.99) |
| Prevalence | 328.49 (306.27  to  350.72) | 305.46 (276.64  to  334.28) | 284.01 (247.28  to  320.73) | 264.03 (219.05  to  309.01) | 245.43 (192.36  to  298.50) | 228.17 (167.42  to  288.91) | 212.18 (144.30  to  280.05) | 197.37 (122.99  to  271.75) | 183.66 (103.40  to  263.91) |
| DALY | 48.08 (42.77  to  53.39) | 44.82 (37.76  to  51.87) | 41.78 (32.69  to  50.87) | 38.96 (27.75  to  50.16) | 36.33 (23.05  to  49.61) | 33.90 (18.64  to  49.17) | 31.66 (14.52  to  48.81) | 29.59 (10.70  to  48.47) | 27.66  (7.18  to  48.14) |
| Mortality | 0.23  (0.10  to  0.37) | 0.25  (0.08  to  0.41) | 0.26  (0.05  to  0.47) | 0.28  (0.03  to  0.53) | 0.29  (-0.01  to  0.60) | 0.31  (-0.05  to  0.68) | 0.34  (-0.10  to  0.78) | 0.36  (-0.16  to  0.88) | 0.39  (-0.23  to  1.00) |
| **Kyrgyzstan** |  |  |  |  |  |  |  |  |  |
| Incidence | 6,420.13 (5,983.54  to  6,856.73) | 5,626.36 (5,079.72  to  6,173.01) | 4,932.10 (4,260.35  to  5,603.86) | 4,324.95 (3,536.07  to  5,113.83) | 3,794.44 (2,905.28  to  4,683.60) | 3,330.94  (2,360.96  to  4,300.92) | 2,925.86 (1,894.66  to  3,957.06) | 2,571.58 (1,497.52  to  3,645.64) | 2,261.54 (1,161.08  to  3,361.99) |
| Prevalence | 97.67 (89.11  to  106.23) | 85.47 (74.86  to  96.09) | 74.83 (61.99  to  87.67) | 65.54 (50.67  to  80.41) | 57.43 (40.86  to  74.01) | 50.36 (32.45  to  68.28) | 44.20 (25.30  to  63.10) | 38.82 (19.26  to  58.37) | 34.11 (14.18  to  54.05) |
| DALY | 64.69 (51.52  to  77.85) | 56.63 (40.78  to  72.47) | 49.64 (30.59  to  68.68) | 43.56 (21.44  to  65.69) | 38.29 (13.46  to  63.11) | 33.69  (6.65  to  60.74) | 29.70  (0.92  to  58.48) | 26.22  (-3.84  to  56.28) | 23.19  (-7.75  to  54.12) |
| Mortality | 0.64  (0.44  to  0.83) | 0.58  (0.33  to  0.82) | 0.52  (0.23  to  0.82) | 0.48  (0.14  to  0.82) | 0.43  (0.05  to  0.81) | 0.39  (-0.03  to  0.81) | 0.36  (-0.09  to  0.81) | 0.32  (-0.15  to  0.80) | 0.29  (-0.20  to  0.79) |
| **Lao People's Democratic Republic** |  |  |  |  |  |  |  |  |  |
| Incidence | 46,787.46 (45,098.19  to  48,476.73) | 46,269.37 (43,972.14  to  48,566.60) | 45,768.74 (42,665.82  to  48,871.65) | 45,283.79 (41,238.29  to  49,329.29) | 44,806.82 (39,716.40  to  49,897.23) | 44,341.85 (38,123.37  to  50,560.33) | 43,913.03 (36,496.03  to  51,330.04) | 43,528.49 (34,849.72  to  52,207.27) | 43,186.31 (33,186.63  to  53,185.98) |
| Prevalence | 681.11 (646.67  to  715.55) | 669.59 (625.71  to  713.47) | 658.49 (602.19  to  714.80) | 647.81 (577.00  to  718.63) | 637.49 (550.65  to  724.33) | 627.60 (523.57  to  731.63) | 618.39 (496.23  to  740.55) | 609.94 (468.84  to  751.04) | 602.21 (441.44  to  762.99) |
| DALY | 679.44 (619.03  to  739.85) | 624.09 (559.09  to  689.09) | 574.16 (502.18  to  646.13) | 529.03 (448.90  to  609.17) | 488.26 (399.65  to  576.88) | 451.45 (354.51  to  548.38) | 418.19 (313.39  to  522.98) | 388.05 (275.99  to  500.12) | 360.69 (242.01  to  479.37) |
| Mortality | 14.69 (13.68  to  15.70) | 13.65 (12.43  to  14.87) | 12.68 (11.24  to  14.12) | 11.78 (10.13  to  13.44) | 10.95  (9.09  to  12.82) | 10.19  (8.13  to  12.24) | 9.47  (7.24  to  11.71) | 8.81  (6.42  to  11.21) | 8.20  (5.67  to  10.73) |
| **Lebanon** |  |  |  |  |  |  |  |  |  |
| Incidence | 34,270.89 (33,152.59  to  35,389.18) | 33,638.19 (32,251.15  to  35,025.24) | 33,029.61 (31,276.13  to  34,783.08) | 32,439.37 (30,251.03  to  34,627.72) | 31,859.70 (29,186.94  to  34,532.45) | 31,294.09 (28,098.85  to  34,489.33) | 30,767.06 (27,019.75  to  34,514.37) | 30,284.01 (25,960.35  to  34,607.67) | 29,839.70 (24,918.60  to  34,760.80) |
| Prevalence | 523.70 (503.69  to  543.71) | 515.80 (490.90  to  540.70) | 508.21 (476.87  to  539.56) | 500.86 (461.95  to  539.77) | 493.63 (446.30  to  540.96) | 486.59 (430.17  to  543.01) | 480.14 (414.09  to  546.20) | 474.39 (398.22  to  550.57) | 469.27 (382.54  to  555.99) |
| DALY | 140.06 (126.81  to  153.31) | 136.38 (121.77  to  150.98) | 132.85 (116.36  to  149.34) | 129.49 (110.70  to  148.28) | 126.29 (104.89  to  147.68) | 123.24 (99.02  to  147.46) | 120.35 (93.14  to  147.55) | 117.60 (87.31  to  147.89) | 115.01 (81.56  to  148.47) |
| Mortality | 2.04  (1.78  to  2.29) | 1.98  (1.68  to  2.28) | 1.93  (1.59  to  2.28) | 1.88  (1.49  to  2.28) | 1.83  (1.38  to  2.28) | 1.78  (1.28  to  2.29) | 1.74  (1.18  to  2.29) | 1.69  (1.08  to  2.30) | 1.64  (0.98  to  2.31) |
| **Malaysia** |  |  |  |  |  |  |  |  |  |
| Incidence | 71,885.84 (68,874.64  to  74,897.03) | 72,718.68 (68,880.24  to  76,557.12) | 73,528.04 (68,556.60  to  78,499.47) | 74,316.75 (67,958.06  to  80,675.44) | 75,082.31 (67,120.48  to  83,044.13) | 75,824.44 (66,069.54  to  85,579.34) | 76,555.81 (64,835.86  to  88,275.77) | 77,283.89 (63,437.65  to  91,130.13) | 78,011.15 (61,883.75  to  94,138.54) |
| Prevalence | 1,094.68 (1,043.40  to  1,145.96) | 1,103.32 (1,038.08  to  1,168.55) | 1,111.54 (1,027.38  to  1,195.70) | 1,119.39 (1,012.25  to  1,226.53) | 1,126.84 (993.33  to  1,260.35) | 1,133.93 (971.10  to  1,296.75) | 1,140.81 (946.03  to  1,335.59) | 1,147.59 (918.41  to  1,376.77) | 1,154.31 (888.42  to  1,420.20) |
| DALY | 226.05 (209.07  to  243.03) | 223.66 (203.23  to  244.08) | 221.33 (196.32  to  246.34) | 219.07 (188.58  to  249.56) | 216.89 (180.24  to  253.54) | 214.80 (171.43  to  258.16) | 212.79 (162.26  to  263.32) | 210.90 (152.81  to  268.98) | 209.10 (143.12  to  275.07) |
| Mortality | 4.55  (4.25  to  4.85) | 4.46  (4.06  to  4.86) | 4.38  (3.87  to  4.89) | 4.29  (3.66  to  4.93) | 4.21  (3.44  to  4.98) | 4.14  (3.23  to  5.04) | 4.06  (3.01  to  5.11) | 3.98  (2.78  to  5.18) | 3.91  (2.56  to  5.26) |
| **Maldives** |  |  |  |  |  |  |  |  |  |
| Incidence | 53,785.61 (50,462.16  to  57,109.06) | 53,585.69 (49,605.79  to  57,565.58) | 53,388.58 (48,508.75  to  58,268.41) | 53,197.02 (47,220.67  to  59,173.37) | 53,010.07 (45,779.69  to  60,240.46) | 52,828.19 (44,214.84  to  61,441.54) | 52,660.07 (42,555.59  to  62,764.55) | 52,510.01 (40,819.70  to  64,200.33) | 52,378.84 (39,016.59  to  65,741.09) |
| Prevalence | 789.81 (723.52  to  856.10) | 782.86 (705.66  to  860.06) | 776.12 (684.57  to  867.67) | 769.63 (660.95  to  878.32) | 763.39 (635.35  to  891.42) | 757.39 (608.20  to  906.59) | 751.76 (579.91  to  923.61) | 746.53 (550.74  to  942.32) | 741.72 (520.84  to  962.61) |
| DALY | 237.82 (200.31  to  275.32) | 228.62 (185.56  to  271.67) | 220.13 (169.58  to  270.68) | 212.30 (152.95  to  271.65) | 205.10 (136.09  to  274.10) | 198.47 (119.24  to  277.70) | 192.38 (102.55  to  282.22) | 186.80 (86.09  to  287.52) | 181.70 (69.88  to  293.52) |
| Mortality | 3.04  (1.68  to  4.40) | 2.90  (1.34  to  4.45) | 2.75  (1.01  to  4.49) | 2.60  (0.67  to  4.53) | 2.45  (0.35  to  4.55) | 2.30  (0.04  to  4.55) | 2.14  (-0.24  to  4.52) | 1.99  (-0.50  to  4.47) | 1.83  (-0.73  to  4.40) |
| **Mongolia** |  |  |  |  |  |  |  |  |  |
| Incidence | 2,471.41 (2,168.32  to  2,774.49) | 2,324.37 (1,842.13  to  2,806.61) | 2,187.29 (1,492.81  to  2,881.78) | 2,059.76 (1,140.98  to  2,978.54) | 1,941.14 (795.78  to  3,086.50) | 1,830.60 (461.89  to  3,199.31) | 1,727.46 (142.14  to  3,312.78) | 1,631.25  (-161.85  to  3,424.36) | 1,541.61  (-449.30  to  3,532.52) |
| Prevalence | 35.95 (30.51  to  41.38) | 34.63 (25.73  to  43.54) | 33.38 (20.42  to  46.33) | 32.18 (14.85  to  49.52) | 31.05  (9.13  to  52.97) | 29.96  (3.34  to  56.58) | 28.93  (-2.44  to  60.30) | 27.94  (-8.20  to  64.09) | 27.01  (-13.89  to  67.91) |
| DALY | 53.64 (43.14  to  64.14) | 51.06 (37.77  to  64.36) | 48.61 (31.88  to  65.35) | 46.28 (25.78  to  66.78) | 44.06 (19.66  to  68.46) | 41.95 (13.62  to  70.27) | 39.93  (7.75  to  72.12) | 38.02  (2.08  to  73.96) | 36.19  (-3.35  to  75.74) |
| Mortality | 0.50  (0.37  to  0.63) | 0.45  (0.32  to  0.59) | 0.42  (0.27  to  0.56) | 0.38  (0.23  to  0.53) | 0.35  (0.19  to  0.50) | 0.32  (0.15  to  0.48) | 0.29  (0.12  to  0.45) | 0.26  (0.09  to  0.43) | 0.24  (0.07  to  0.41) |
| **Myanmar** |  |  |  |  |  |  |  |  |  |
| Incidence | 42,331.15  (40,892.12  to  43,770.19) | 41,993.70  (40,113.42  to  43,873.98) | 41,658.14  (39,180.33  to  44,135.94) | 41,324.99  (38,135.98  to  44,514.00) | 40,995.04  (37,007.79  to  44,982.30) | 40,670.42  (35,814.20  to  45,526.64) | 40,354.76  (34,570.82  to  46,138.69) | 40,048.86  (33,286.58  to  46,811.13) | 39,752.99  (31,967.31  to  47,538.66) |
| Prevalence | 604.69 (577.24  to  632.14) | 595.94 (562.16  to  629.71) | 587.38 (545.00  to  629.77) | 579.04 (526.36  to  631.71) | 570.89 (506.69  to  635.09) | 562.97 (486.30  to  639.65) | 555.35 (465.44  to  645.25) | 548.02 (444.27  to  651.76) | 540.98 (422.88  to  659.08) |
| DALY | 525.19 (478.25  to  572.14) | 486.46 (434.61  to  538.32) | 451.18 (392.04  to  510.31) | 419.00 (351.34  to  486.65) | 389.63 (313.01  to  466.24) | 362.79 (277.27  to  448.31) | 338.28 (244.18  to  432.37) | 315.89 (213.69  to  418.08) | 295.41 (185.66  to  405.15) |
| Mortality | 11.67 (11.13  to  12.22) | 11.01 (10.19  to  11.83) | 10.39  (9.27  to  11.52) | 9.81  (8.37  to  11.25) | 9.26  (7.51  to  11.02) | 8.75  (6.69  to  10.81) | 8.27  (5.91  to  10.63) | 7.82  (5.18  to  10.46) | 7.39  (4.49  to  10.30) |
| **Nepal** |  |  |  |  |  |  |  |  |  |
| Incidence | 43,902.52 (41,721.47  to  46,083.57) | 41,193.07 (37,721.10  to  44,665.03) | 38,670.33 (33,721.49  to  43,619.18) | 36,316.30 (29,834.19  to  42,798.40) | 34,119.58 (26,107.77  to  42,131.39) | 32,080.34 (22,570.64  to  41,590.03) | 30,202.57 (19,244.69  to  41,160.45) | 28,474.57 (16,129.26  to  40,819.89) | 26,881.72 (13,215.82  to  40,547.62) |
| Prevalence | 595.63 (560.51  to  630.75) | 556.92 (502.42  to  611.41) | 520.99 (444.43  to  597.54) | 487.61 (388.28  to  586.94) | 456.60 (334.69  to  578.51) | 427.92 (284.06  to  571.77) | 401.56 (236.66  to  566.46) | 377.36 (192.45  to  562.27) | 355.12 (151.31  to  558.93) |
| DALY | 574.74 (541.16  to  608.32) | 534.50 (492.14  to  576.86) | 498.07 (444.51  to  551.64) | 465.04 (399.32  to  530.76) | 435.05 (356.97  to  513.13) | 407.75 (317.49  to  498.02) | 382.86 (280.81  to  484.92) | 360.12 (246.78  to  473.46) | 339.30 (215.23  to  463.38) |
| Mortality | 20.39 (19.36  to  21.41) | 19.41 (17.89  to  20.92) | 18.48 (16.42  to  20.55) | 17.61 (14.96  to  20.25) | 16.78 (13.55  to  20.01) | 16.00 (12.18  to  19.81) | 15.26 (10.86  to  19.65) | 14.55  (9.60  to  19.51) | 13.89  (8.39  to  19.38) |
| **Oman** |  |  |  |  |  |  |  |  |  |
| Incidence | 24,896.23 (23,678.22  to  26,114.25) | 25,101.48 (23,420.91  to  26,782.05) | 25,312.46 (23,018.98  to  27,605.94) | 25,532.21 (22,506.81  to  28,557.61) | 25,764.52 (21,905.01  to  29,624.03) | 26,013.41 (21,225.95  to  30,800.88) | 26,281.20 (20,477.52  to  32,084.88) | 26,569.29 (19,663.10  to  33,475.49) | 26,879.02 (18,783.41  to  34,974.62) |
| Prevalence | 377.95 (354.67  to  401.24) | 382.93 (350.53  to  415.34) | 388.05 (343.74  to  432.36) | 393.36 (334.88  to  451.85) | 398.95 (324.26  to  473.64) | 404.87 (312.05  to  497.68) | 411.14 (298.36  to  523.92) | 417.78 (283.19  to  552.38) | 424.83 (266.54  to  583.12) |
| DALY | 96.69 (86.05  to  107.34) | 94.77 (82.00  to  107.55) | 92.92 (77.31  to  108.53) | 91.13 (72.19  to  110.08) | 89.42 (66.78  to  112.06) | 87.77 (61.17  to  114.37) | 86.19 (55.44  to  116.95) | 84.68 (49.62  to  119.75) | 83.24 (43.74  to  122.73) |
| Mortality | 1.61  (1.14  to  2.07) | 1.60  (1.03  to  2.17) | 1.59  (0.90  to  2.28) | 1.58  (0.76  to  2.40) | 1.57  (0.61  to  2.54) | 1.56  (0.45  to  2.68) | 1.55  (0.28  to  2.83) | 1.54  (0.10  to  2.98) | 1.53  (-0.08  to  3.14) |
| **Pakistan** |  |  |  |  |  |  |  |  |  |
| Incidence | 84,788.29 (83,004.41  to  86,572.18) | 84,368.10 (81,317.18  to  87,419.02) | 83,888.07 (79,254.47  to  88,521.67) | 83,466.56 (77,019.29  to  89,913.83) | 83,033.53 (74,580.55  to  91,486.50) | 82,470.43 (71,843.81  to  93,097.04) | 81,776.07 (68,854.60  to  94,697.54) | 81,102.84 (65,770.90  to  96,434.77) | 80,589.89 (62,715.53  to  98,464.26) |
| Prevalence | 1,251.14 (1,220.80  to  1,281.48) | 1,245.10 (1,194.62  to  1,295.59) | 1,238.43 (1,162.68  to  1,314.18) | 1,232.31 (1,127.55  to  1,337.07) | 1,225.43 (1,088.63  to  1,362.24) | 1,216.64 (1,045.20  to  1,388.09) | 1,206.36 (998.32  to  1,414.40) | 1,196.56 (950.06  to  1,443.07) | 1,188.87 (901.84  to  1,475.91) |
| DALY | 1,026.46 (975.48  to  1,077.44) | 939.37 (879.98  to  998.76) | 861.85 (791.56  to  932.14) | 792.65 (710.70  to  874.60) | 730.83 (637.31  to  824.35) | 675.67 (571.05  to  780.29) | 626.34 (511.31  to  741.37) | 581.94 (457.29  to  706.58) | 541.83 (408.36  to  675.31) |
| Mortality | 29.21 (28.15  to  30.27) | 27.27 (25.82  to  28.72) | 25.49 (23.59  to  27.39) | 23.84 (21.47  to  26.22) | 22.33 (19.49  to  25.17) | 20.94 (17.63  to  24.24) | 19.65 (15.91  to  23.40) | 18.46 (14.30  to  22.62) | 17.35 (12.81  to  21.90) |
| **Palestine** |  |  |  |  |  |  |  |  |  |
| Incidence | 14,630.77 (13,567.03  to  15,694.52) | 13,712.15 (12,146.93  to  15,277.38) | 12,840.04 (10,677.94  to  15,002.13) | 12,021.13 (9,239.35  to  14,802.90) | 11,257.53 (7,864.59  to  14,650.47) | 10,543.54 (6,563.31  to  14,523.77) | 9,875.67 (5,341.35  to  14,409.99) | 9,254.51 (4,202.63  to  14,306.38) | 8,681.03 (3,147.57  to  14,214.50) |
| Prevalence | 191.63 (176.04  to  207.23) | 181.06 (153.31  to  208.80) | 170.93 (129.86  to  212.00) | 161.34 (106.59  to  216.10) | 152.30 (83.90  to  220.70) | 143.70 (61.98  to  225.42) | 135.57 (41.03  to  230.10) | 127.96 (21.20  to  234.71) | 120.89 (2.52  to  239.26) |
| DALY | 45.66 (38.25  to  53.07) | 40.98 (33.25  to  48.71) | 36.81 (28.53  to  45.09) | 33.10 (24.17  to  42.03) | 29.79 (20.21  to  39.37) | 26.84 (16.67  to  37.02) | 24.21 (13.52  to  34.90) | 21.87 (10.75  to  32.99) | 19.78  (8.33  to  31.23) |
| Mortality | 0.50  (0.35  to  0.65) | 0.47  (0.31  to  0.63) | 0.44  (0.26  to  0.62) | 0.41  (0.22  to  0.60) | 0.39  (0.18  to  0.59) | 0.36  (0.15  to  0.58) | 0.34  (0.11  to  0.57) | 0.32  (0.08  to  0.56) | 0.30  (0.05  to  0.55) |
| **Philippines** |  |  |  |  |  |  |  |  |  |
| Incidence | 39,221.23 (37,873.31  to  40,569.15) | 39,093.83 (37,140.34  to  41,047.31) | 38,956.52 (36,196.33  to  41,716.71) | 38,812.35 (35,101.59  to  42,523.10) | 38,661.42 (33,886.78  to  43,436.06) | 38,503.61 (32,569.56  to  44,437.65) | 38,341.44 (31,165.36  to  45,517.52) | 38,177.56 (29,685.72  to  46,669.41) | 38,013.29 (28,138.58  to  47,888.01) |
| Prevalence | 590.90 (571.54  to  610.25) | 586.86 (558.05  to  615.66) | 582.70 (541.56  to  623.83) | 578.46 (522.99  to  633.93) | 574.13 (502.76  to  645.51) | 569.71 (481.13  to  658.29) | 565.25 (458.36  to  672.15) | 560.82 (434.65  to  686.99) | 556.41 (410.10  to  702.72) |
| DALY | 389.74 (367.96  to  411.53) | 374.75 (348.34  to  401.16) | 360.43 (327.65  to  393.20) | 346.72 (306.50  to  386.95) | 333.61 (285.31  to  381.90) | 321.05 (264.35  to  377.75) | 309.05 (243.81  to  374.30) | 297.60 (223.80  to  371.40) | 286.65 (204.36  to  368.93) |
| Mortality | 8.46  (8.08  to  8.84) | 8.20  (7.65  to  8.74) | 7.94  (7.20  to  8.68) | 7.68  (6.73  to  8.64) | 7.44  (6.27  to  8.62) | 7.20  (5.80  to  8.61) | 6.98  (5.34  to  8.61) | 6.76  (4.89  to  8.62) | 6.54  (4.44  to  8.64) |
| **Qatar** |  |  |  |  |  |  |  |  |  |
| Incidence | 22,053.45 (20,640.07  to  23,466.84) | 22,441.51 (20,365.12  to  24,517.89) | 22,828.57 (19,857.46  to  25,799.68) | 23,216.37 (19,164.79  to  27,267.94) | 23,606.90 (18,311.43  to  28,902.36) | 24,001.85 (17,309.49  to  30,694.21) | 24,402.72 (16,166.08  to  32,639.35) | 24,810.60 (14,884.53  to  34,736.67) | 25,226.93 (13,466.12  to  36,987.73) |
| Prevalence | 329.23 (309.48  to  348.99) | 339.07 (304.84  to  373.29) | 349.01 (296.18  to  401.83) | 359.09 (283.99  to  434.20) | 369.33 (268.38  to  470.28) | 379.75 (249.39  to  510.11) | 390.41 (227.03  to  553.78) | 401.36 (201.27  to  601.44) | 412.65 (172.02  to  653.29) |
| DALY | 56.72 (51.68  to  61.75) | 56.54 (49.51  to  63.58) | 56.39  (46.92  to  65.85) | 56.24 (44.01  to  68.48) | 56.13 (40.83  to  71.42) | 56.04 (37.43  to  74.65) | 55.98 (33.82  to  78.13) | 55.95 (30.03  to  81.87) | 55.96 (26.05  to  85.87) |
| Mortality | 0.14  (0.06  to  0.23) | 0.14  (0.05  to  0.23) | 0.13  (0.04  to  0.22) | 0.13  (0.03  to  0.22) | 0.12  (0.02  to  0.22) | 0.11  (0.01  to  0.22) | 0.11  (-0.00  to  0.22) | 0.10  (-0.01  to  0.22) | 0.10  (-0.02  to  0.22) |
| **Republic of Korea** |  |  |  |  |  |  |  |  |  |
| Incidence | 7,568.54 (7,111.96  to  8,025.12) | 7,404.80 (6,774.35  to  8,035.25) | 7,243.68 (6,386.61  to  8,100.76) | 7,085.24 (5,967.79  to  8,202.69) | 6,929.50 (5,528.86  to  8,330.14) | 6,776.44 (5,076.50  to  8,476.37) | 6,626.03 (4,615.37  to  8,636.69) | 6,478.33 (4,148.90  to  8,807.75) | 6,333.33 (3,679.74  to  8,986.93) |
| Prevalence | 117.67 (110.54  to  124.79) | 115.18 (105.37  to  124.99) | 112.73 (99.44  to  126.02) | 110.31 (93.04  to  127.58) | 107.94 (86.34  to  129.53) | 105.60 (79.44  to  131.76) | 103.30 (72.40  to  134.20) | 101.04 (65.27  to  136.81) | 98.82 (58.10  to  139.53) |
| DALY | 38.97 (36.52  to  41.42) | 38.05 (35.01  to  41.08) | 37.14 (33.33  to  40.95) | 36.25 (31.56  to  40.95) | 35.38 (29.72  to  41.05) | 34.53 (27.84  to  41.23) | 33.70 (25.94  to  41.46) | 32.89 (24.04  to  41.74) | 32.09 (22.14  to  42.04) |
| Mortality | 1.37  (1.27  to  1.47) | 1.35  (1.21  to  1.49) | 1.33  (1.14  to  1.52) | 1.30  (1.07  to  1.54) | 1.28  (0.99  to  1.58) | 1.26  (0.91  to  1.61) | 1.24  (0.82  to  1.65) | 1.21  (0.74  to  1.68) | 1.19  (0.66  to  1.72) |
| **Saudi Arabia** |  |  |  |  |  |  |  |  |  |
| Incidence | 32,120.43 (30,086.76  to  34,154.11) | 32,346.05 (29,565.31  to  35,126.79) | 32,580.14 (28,795.61  to  36,364.67) | 32,824.77 (27,835.54  to  37,813.99) | 33,079.34 (26,715.39  to  39,443.29) | 33,348.85 (25,455.76  to  41,241.94) | 33,641.47 (24,073.17  to  43,209.76) | 33,961.22 (22,574.80  to  45,347.64) | 34,308.97 (20,961.64  to  47,656.30) |
| Prevalence | 477.01 (441.40  to  512.62) | 481.31 (431.68  to  530.95) | 485.76 (417.41  to  554.11) | 490.39 (399.63  to  581.16) | 495.22 (378.86  to  611.57) | 500.32 (355.44  to  645.20) | 505.82 (329.59  to  682.04) | 511.78 (301.42  to  722.14) | 518.24 (270.91  to  765.56) |
| DALY | 117.96 (106.76  to  129.16) | 115.29 (102.28  to  128.30) | 112.99 (97.40  to  128.57) | 111.02 (92.28  to  129.76) | 109.37 (87.02  to  131.71) | 108.01 (81.69  to  134.33) | 106.94 (76.33  to  137.56) | 106.15 (70.92  to  141.37) | 105.61 (65.47  to  145.74) |
| Mortality | 2.72  (2.44  to  3.00) | 2.68  (2.33  to  3.04) | 2.65  (2.21  to  3.09) | 2.61  (2.08  to  3.15) | 2.58  (1.94  to  3.22) | 2.55  (1.80  to  3.29) | 2.52  (1.66  to  3.38) | 2.49  (1.51  to  3.47) | 2.46  (1.36  to  3.56) |
| **Singapore** |  |  |  |  |  |  |  |  |  |
| Incidence | 1,260.49 (1,142.77  to  1,378.21) | 1,324.85 (1,149.11  to  1,500.60) | 1,393.03 (1,133.46  to  1,652.59) | 1,465.43 (1,098.35  to  1,832.50) | 1,542.35 (1,044.14  to  2,040.56) | 1,624.03 (969.99  to  2,278.07) | 1,710.82 (874.53  to  2,547.11) | 1,803.17 (755.99  to  2,850.35) | 1,901.57 (612.19  to  3,190.96) |
| Prevalence | 17.61 (15.71  to  19.50) | 18.16 (15.31  to  21.02) | 18.74 (14.68  to  22.81) | 19.35 (13.85  to  24.85) | 19.98 (12.81  to  27.14) | 20.63 (11.58  to  29.69) | 21.32 (10.13  to  32.50) | 22.04  (8.47  to  35.60) | 22.78  (6.58  to  38.99) |
| DALY | 15.28 (13.73  to  16.83) | 14.71 (12.33  to  17.09) | 14.16 (10.83  to  17.49) | 13.63  (9.29  to  17.98) | 13.12  (7.73  to  18.51) | 12.63  (6.17  to  19.08) | 12.15  (4.64  to  19.66) | 11.69  (3.13  to  20.26) | 11.25  (1.66  to  20.85) |
| Mortality | 0.66  (0.52  to  0.79) | 0.63  (0.46  to  0.79) | 0.60  (0.41  to  0.80) | 0.58  (0.35  to  0.80) | 0.55  (0.30  to  0.81) | 0.53  (0.24  to  0.81) | 0.51  (0.19  to  0.82) | 0.48  (0.14  to  0.83) | 0.46  (0.09  to  0.83) |
| **Sri Lanka** |  |  |  |  |  |  |  |  |  |
| Incidence | 48,462.10 (46,815.58  to  50,108.62) | 48,918.12 (46,690.80  to  51,145.45) | 49,362.55 (46,348.99  to  52,376.11) | 49,798.94 (45,836.99  to  53,760.89) | 50,224.88 (45,179.46  to  55,270.30) | 50,638.41 (44,390.79  to  56,886.04) | 51,052.92 (43,495.33  to  58,610.51) | 51,475.74 (42,506.12  to  60,445.35) | 51,909.00 (41,427.92  to  62,390.08) |
| Prevalence | 705.27 (680.14  to  730.40) | 709.94 (676.10  to  743.79) | 714.36 (668.87  to  759.86) | 718.59 (659.13  to  778.04) | 722.58 (647.28  to  797.88) | 726.32 (633.53  to  819.11) | 730.03 (618.29  to  841.77) | 733.85 (601.78  to  865.91) | 737.80 (584.09  to  891.51) |
| DALY | 165.94 (156.14  to  175.75) | 161.71 (148.57  to  174.84) | 157.68 (140.26  to  175.09) | 153.84 (131.53  to  176.15) | 150.20 (122.60  to  177.81) | 146.75 (113.55  to  179.94) | 143.48 (104.48  to  182.47) | 140.40 (95.43  to  185.37) | 137.50 (86.43  to  188.58) |
| Mortality | 3.64  (3.35  to  3.92) | 3.49  (3.11  to  3.88) | 3.35  (2.85  to  3.85) | 3.22  (2.60  to  3.84) | 3.09  (2.34  to  3.83) | 2.96  (2.10  to  3.83) | 2.84  (1.85  to  3.83) | 2.73  (1.62  to  3.84) | 2.62  (1.39  to  3.84) |
| **Syrian Arab Republic** |  |  |  |  |  |  |  |  |  |
| Incidence | 25,962.35 (24,308.80  to  27,615.90) | 25,627.41 (23,619.23  to  27,635.59) | 25,313.60 (22,812.29  to  27,814.91) | 25,022.10 (21,923.35  to  28,120.85) | 24,748.14 (20,972.57  to  28,523.70) | 24,493.63 (19,977.52  to  29,009.73) | 24,271.25 (18,960.14  to  29,582.36) | 24,084.77 (17,929.02  to  30,240.52) | 23,931.48 (16,884.24  to  30,978.71) |
| Prevalence | 382.63 (355.58  to  409.69) | 377.77 (345.45  to  410.09) | 373.24 (333.65  to  412.84) | 369.07 (320.65  to  417.50) | 365.20 (306.75  to  423.65) | 361.66 (292.21  to  431.11) | 358.63 (277.34  to  439.92) | 356.18 (262.29  to  450.07) | 354.27 (247.04  to  461.49) |
| DALY | 80.27 (71.14  to  89.41) | 77.59 (67.78  to  87.40) | 75.10 (64.27  to  85.94) | 72.80 (60.66  to  84.93) | 70.67 (57.03  to  84.31) | 68.71 (53.41  to  84.00) | 66.92 (49.86  to  83.98) | 65.30 (46.40  to  84.21) | 63.85 (43.02  to  84.67) |
| Mortality | 1.06  (0.88  to  1.25) | 1.04  (0.80  to  1.27) | 1.01  (0.73  to  1.29) | 0.98  (0.65  to  1.32) | 0.96  (0.57  to  1.35) | 0.93  (0.49  to  1.38) | 0.91  (0.41  to  1.42) | 0.89  (0.33  to  1.46) | 0.87  (0.24  to  1.49) |
| **Taiwan**  **(Province of China)** |  |  |  |  |  |  |  |  |  |
| Incidence | 54,160.82 (45,996.21  to  62,325.42) | 54,508.84 (43,687.51  to  65,330.17) | 54,857.48 (40,390.29  to  69,324.67) | 55,207.44 (36,316.78  to  74,098.10) | 55,561.53 (31,602.09  to  79,520.97) | 55,920.42 (26,325.25  to  85,515.59) | 56,282.38 (20,534.53  to  92,030.22) | 56,645.93 (14,261.64  to  99,030.23) | 57,011.08 (7,528.97  to  106,493.19) |
| Prevalence | 827.89 (695.47  to  960.31) | 832.46 (655.93  to  1,008.98) | 837.08 (600.21  to  1,073.94) | 841.76 (531.87  to  1,151.64) | 846.54 (453.16  to  1,239.93) | 851.45 (365.35  to  1,337.55) | 856.44 (269.24  to  1,443.65) | 861.51 (165.33  to  1,557.68) | 866.63 (53.99  to  1,679.28) |
| DALY | 97.37 (85.80  to  108.93) | 96.77 (80.27  to  113.28) | 96.20 (73.12  to  119.28) | 95.63 (64.83  to  126.43) | 95.08 (55.67  to  134.50) | 94.56 (45.78  to  143.34) | 94.05 (35.25  to  152.85) | 93.57 (24.16  to  162.98) | 93.11 (12.56  to  173.66) |
| Mortality | 0.25  (0.21  to  0.30) | 0.24  (0.19  to  0.30) | 0.24  (0.17  to  0.31) | 0.23  (0.15  to  0.31) | 0.22  (0.13  to  0.32) | 0.22  (0.11  to  0.33) | 0.21  (0.08  to  0.33) | 0.20  (0.06  to  0.34) | 0.20  (0.04  to  0.35) |
| **Tajikistan** |  |  |  |  |  |  |  |  |  |
| Incidence | 20,106.71 (19,276.62  to  20,936.80) | 19,082.80 (17,933.40  to  20,232.20) | 18,121.08 (16,574.79  to  19,667.38) | 17,211.87 (15,234.38  to  19,189.35) | 16,340.98 (13,920.76  to  18,761.19) | 15,507.04 (12,645.09  to  18,369.00) | 14,728.29 (11,431.81  to  18,024.76) | 14,007.52 (10,287.16  to  17,727.89) | 13,336.59 (9,205.95  to  17,467.22) |
| Prevalence | 356.27 (336.29  to  376.26) | 341.15 (314.22  to  368.07) | 326.79 (291.26  to  362.32) | 313.11 (268.12  to  358.09) | 299.96 (245.13  to  354.79) | 287.35 (222.53  to  352.18) | 275.53 (200.70  to  350.37) | 264.54 (179.76  to  349.32) | 254.27 (159.67  to  348.86) |
| DALY | 897.18 (781.20  to  1,013.16) | 853.45 (723.98  to  982.92) | 811.91 (663.40  to  960.41) | 772.42 (601.22  to  943.62) | 734.90 (538.93  to  930.87) | 699.24 (477.59  to  920.90) | 665.36 (417.89  to  912.83) | 633.17 (360.30  to  906.04) | 602.58 (305.10  to  900.06) |
| Mortality | 10.35  (9.40  to  11.30) | 9.97  (8.75  to  11.18) | 9.60  (8.08  to  11.12) | 9.25  (7.39  to  11.10) | 8.90  (6.70  to  11.11) | 8.58  (6.02  to  11.13) | 8.26  (5.35  to  11.17) | 7.96  (4.69  to  11.22) | 7.66  (4.05  to  11.27) |
| **Thailand** |  |  |  |  |  |  |  |  |  |
| Incidence | 51,219.00 (49,553.13  to  52,884.86) | 52,282.41 (49,297.58  to  55,267.25) | 53,377.06 (48,682.51  to  58,071.61) | 54,504.52 (47,769.85  to  61,239.19) | 55,659.16 (46,574.47  to  64,743.86) | 56,835.36 (45,097.47  to  68,573.26) | 58,040.96 (43,347.75  to  72,734.17) | 59,279.54 (41,326.16  to  77,232.93) | 60,550.81 (39,027.74  to  82,073.88) |
| Prevalence | 767.79 (739.44  to  796.15) | 781.01 (730.77  to  831.25) | 794.58 (716.14  to  873.01) | 808.49 (696.59  to  920.39) | 822.68 (672.45  to  972.92) | 837.09 (643.82  to  1,030.37) | 851.80 (610.87  to  1,092.74) | 866.85 (573.63  to  1,160.07) | 882.21 (532.08  to  1,232.34) |
| DALY | 281.20 (269.92  to  292.47) | 280.56 (263.10  to  298.03) | 280.03 (254.52  to  305.53) | 279.59 (244.69  to  314.48) | 279.24 (233.84  to  324.63) | 278.98 (222.10  to  335.86) | 278.81 (209.56  to  348.07) | 278.73 (196.27  to  361.20) | 278.74 (182.28  to  375.19) |
| Mortality | 6.87  (6.57  to  7.18) | 6.83  (6.35  to  7.32) | 6.80  (6.08  to  7.51) | 6.76  (5.79  to  7.73) | 6.72  (5.48  to  7.97) | 6.69  (5.14  to  8.24) | 6.66  (4.79  to  8.52) | 6.62  (4.42  to  8.83) | 6.60  (4.03  to  9.16) |
| **Timor-Leste** |  |  |  |  |  |  |  |  |  |
| Incidence | 57,332.37 (55,447.95  to  59,216.79) | 55,900.66 (53,210.91  to  58,590.40) | 54,513.05 (50,801.31  to  58,224.79) | 53,164.53 (48,298.85  to  58,030.22) | 51,849.64 (45,741.82  to  57,957.46) | 50,572.78 (43,159.01  to  57,986.56) | 49,361.08 (40,594.74  to  58,127.43) | 48,217.93 (38,064.03  to  58,371.84) | 47,137.36 (35,568.88  to  58,705.84) |
| Prevalence | 878.75 (839.20  to  918.31) | 855.29 (801.79  to  908.79) | 832.72 (762.12  to  903.32) | 811.00 (721.31  to  900.69) | 790.10 (679.99  to  900.21) | 770.11 (638.61  to  901.62) | 751.33 (597.73  to  904.93) | 733.76 (557.54  to  909.98) | 717.30 (518.02  to  916.58) |
| DALY | 698.03 (613.85  to  782.20) | 665.76 (570.54  to  760.98) | 635.73 (524.70  to  746.76) | 607.79 (477.84  to  737.74) | 581.82 (431.10  to  732.55) | 557.68 (385.15  to  730.20) | 535.23 (340.41  to  730.05) | 514.36 (297.08  to  731.63) | 494.95 (255.26  to  734.65) |
| Mortality | 15.96 (13.75  to  18.18) | 15.54 (12.81  to  18.28) | 15.14 (11.83  to  18.45) | 14.75 (10.81  to  18.68) | 14.37  (9.78  to  18.96) | 14.00  (8.74  to  19.26) | 13.65  (7.70  to  19.59) | 13.30  (6.67  to  19.94) | 12.97  (5.64  to  20.30) |
| **Turkey** |  |  |  |  |  |  |  |  |  |
| Incidence | 22,609.51 (21,057.96  to  24,161.07) | 21,776.71 (19,815.28  to  23,738.13) | 20,987.04 (18,478.77  to  23,495.30) | 20,238.85 (17,102.54  to  23,375.17) | 19,528.90 (15,716.92  to  23,340.89) | 18,858.50 (14,342.17  to  23,374.82) | 18,235.85 (12,997.12  to  23,474.58) | 17,661.37 (11,688.21  to  23,634.54) | 17,130.63 (10,414.89  to  23,846.38) |
| Prevalence | 325.02 (301.28  to  348.77) | 312.23 (282.78  to  341.68) | 300.11 (263.02  to  337.21) | 288.65 (242.75  to  334.56) | 277.79 (222.42  to  333.17) | 267.55 (202.34  to  332.77) | 258.05 (182.78  to  333.32) | 249.29 (163.84  to  334.75) | 241.22 (145.50  to  336.94) |
| DALY | 81.19 (69.47  to  92.91) | 75.85 (64.10  to  87.61) | 71.03 (58.91  to  83.16) | 66.68 (53.96  to  79.41) | 62.76 (49.28  to  76.24) | 59.22 (44.88  to  73.55) | 56.03 (40.79  to  71.28) | 53.17 (36.99  to  69.35) | 50.59 (33.46  to  67.72) |
| Mortality | 1.04  (0.96  to  1.12) | 1.00  (0.90  to  1.11) | 0.97  (0.84  to  1.10) | 0.94  (0.78  to  1.10) | 0.91  (0.72  to  1.09) | 0.88  (0.67  to  1.09) | 0.85  (0.61  to  1.10) | 0.83  (0.55  to  1.10) | 0.80  (0.50  to  1.10) |
| **Turkmenistan** |  |  |  |  |  |  |  |  |  |
| Incidence | 7,092.49 (6,571.53  to  7,613.46) | 6,653.60 (6,019.90  to  7,287.31) | 6,246.66 (5,466.10  to  7,027.21) | 5,869.02 (4,925.95  to  6,812.09) | 5,517.58 (4,407.20  to  6,627.97) | 5,190.30 (3,913.98  to  6,466.61) | 4,886.81 (3,449.59  to  6,324.03) | 4,605.60 (3,014.52  to  6,196.68) | 4,344.48 (2,607.73  to  6,081.23) |
| Prevalence | 116.17 (105.07  to  127.28) | 110.49 (96.12  to  124.86) | 105.17 (86.77  to  123.58) | 100.19 (77.37  to  123.02) | 95.52 (68.10  to  122.93) | 91.12 (59.07  to  123.18) | 87.02 (50.35  to  123.69) | 83.18 (41.96  to  124.39) | 79.59 (33.93  to  125.24) |
| DALY | 82.97 (61.39  to  104.54) | 75.94 (47.75  to  104.13) | 69.65 (33.11  to  106.19) | 64.03 (18.67  to  109.39) | 58.99  (4.92  to  113.06) | 54.47  (-7.92  to  116.87) | 50.42  (-19.80  to  120.64) | 46.79  (-30.72  to  124.31) | 43.54  (-40.75  to  127.82) |
| Mortality | 0.97  (0.66  to  1.29) | 0.94  (0.50  to  1.37) | 0.90  (0.33  to  1.48) | 0.87  (0.15  to  1.59) | 0.84  (-0.03  to  1.71) | 0.81  (-0.22  to  1.83) | 0.78  (-0.40  to  1.96) | 0.75  (-0.58  to  2.09) | 0.73  (-0.76  to  2.21) |
| **United Arab Emirates** |  |  |  |  |  |  |  |  |  |
| Incidence | 25,354.22 (24,357.68  to  26,350.77) | 25,119.12 (23,625.99  to  26,612.24) | 24,900.13 (22,768.98  to  27,031.28) | 24,697.00 (21,831.98  to  27,562.02) | 24,503.72 (20,831.88  to  28,175.56) | 24,318.89 (19,778.94  to  28,858.84) | 24,150.38 (18,688.21  to  29,612.55) | 24,002.55 (17,567.68  to  30,437.43) | 23,874.21 (16,418.45  to  31,329.96) |
| Prevalence | 380.48 (362.18  to  398.78) | 378.46 (350.09  to  406.82) | 376.65 (335.64  to  417.66) | 375.06 (319.61  to  430.52) | 373.64 (302.27  to  445.00) | 372.36 (283.80  to  460.92) | 371.31 (264.38  to  478.25) | 370.55 (244.12  to  496.98) | 370.06 (223.03  to  517.08) |
| DALY | 88.11 (82.22  to  94.00) | 83.57 (75.83  to  91.31) | 79.28 (69.33  to  89.23) | 75.24  (62.92  to  87.56) | 71.45 (56.70  to  86.20) | 67.90 (50.72  to  85.07) | 64.56 (45.01  to  84.11) | 61.43 (39.58  to  83.28) | 58.50 (34.43  to  82.57) |
| Mortality | 1.38  (0.96  to  1.81) | 1.32  (0.85  to  1.78) | 1.25  (0.74  to  1.77) | 1.19  (0.63  to  1.75) | 1.13  (0.52  to  1.74) | 1.07  (0.41  to  1.73) | 1.02  (0.31  to  1.72) | 0.96  (0.22  to  1.71) | 0.91  (0.12  to  1.70) |
| **Uzbekistan** |  |  |  |  |  |  |  |  |  |
| Incidence | 3,382.59 (3,060.55  to  3,704.64) | 3,152.17 (2,770.25  to  3,534.09) | 2,938.75 (2,477.30  to  3,400.20) | 2,741.12 (2,191.37  to  3,290.86) | 2,557.79 (1,917.94  to  3,197.63) | 2,387.51 (1,659.86  to  3,115.17) | 2,229.50 (1,418.65  to  3,040.35) | 2,082.98 (1,194.72  to  2,971.23) | 1,947.04 (987.76  to  2,906.32) |
| Prevalence | 46.55 (41.53  to  51.56) | 43.20 (37.19  to  49.22) | 40.12 (32.84  to  47.41) | 37.28 (28.61  to  45.94) | 34.64 (24.60  to  44.69) | 32.21 (20.84  to  43.58) | 29.95 (17.35  to  42.56) | 27.87 (14.13  to  41.61) | 25.95 (11.19  to  40.71) |
| DALY | 26.20 (18.18  to  34.22) | 24.23 (13.56  to  34.90) | 22.44  (8.33  to  36.55) | 20.82  (3.00  to  38.64) | 19.34  (-2.20  to  40.88) | 17.99  (-7.16  to  43.15) | 16.77  (-11.83  to  45.37) | 15.66  (-16.19  to  47.50) | 14.64  (-20.24  to  49.52) |
| Mortality | 0.30  (0.20  to  0.41) | 0.29  (0.12  to  0.46) | 0.28  (0.03  to  0.52) | 0.27  (-0.05  to  0.59) | 0.25  (-0.14  to  0.65) | 0.24  (-0.23  to  0.72) | 0.23  (-0.32  to  0.79) | 0.22  (-0.41  to  0.85) | 0.21  (-0.49  to  0.92) |
| **Viet Nam** |  |  |  |  |  |  |  |  |  |
| Incidence | 53,339.38  (49,176.22  to  57,502.54) | 52,814.13  (47,691.34  to  57,936.93) | 52,285.02  (46,332.25  to  58,237.79) | 51,751.23  (45,050.96  to  58,451.51) | 51,211.53  (43,822.21  to  58,600.86) | 50,672.54  (42,637.52  to  58,707.55) | 50,135.98  (41,490.03  to  58,781.94) | 49,601.25  (40,373.37  to  58,829.14) | 49,066.93  (39,282.13  to  58,851.72) |
| Prevalence | 775.27 (705.72  to  844.82) | 765.88 (680.21  to  851.56) | 756.48 (657.01  to  855.96) | 747.06 (635.28  to  858.83) | 737.58 (614.58  to  860.59) | 728.16 (594.73  to  861.59) | 718.82 (575.61  to  862.03) | 709.55 (557.11  to  862.00) | 700.33 (539.13  to  861.53) |
| DALY | 119.64 (106.98  to  132.30) | 113.29 (99.64  to  126.93) | 107.39 (92.94  to  121.83) | 101.90 (86.80  to  117.00) | 96.79 (81.16  to  112.43) | 92.05 (75.98  to  108.13) | 87.66 (71.23  to  104.08) | 83.57 (66.87  to  100.27) | 79.76 (62.84  to  96.68) |
| Mortality | 1.63  (1.52  to  1.74) | 1.52  (1.39  to  1.64) | 1.41  (1.27  to  1.55) | 1.31  (1.17  to  1.46) | 1.22  (1.07  to  1.37) | 1.14  (0.98  to  1.29) | 1.06  (0.90  to  1.21) | 0.98  (0.83  to  1.14) | 0.91  (0.76  to  1.07) |
| **Yemen** |  |  |  |  |  |  |  |  |  |
| Incidence | 36,269.52 (33,793.41  to  38,745.62) | 33,489.19 (30,710.23  to  36,268.16) | 30,883.49 (27,702.10  to  34,064.87) | 28,463.33 (24,834.10  to  32,092.56) | 26,238.81 (22,152.96  to  30,324.65) | 24,212.61 (19,689.56  to  28,735.65) | 22,354.41 (17,434.97  to  27,273.85) | 20,652.45 (15,381.70  to  25,923.21) | 19,103.55 (13,521.48  to  24,685.63) |
| Prevalence | 532.65 (487.51  to  577.79) | 484.85 (435.43  to  534.27) | 440.94 (385.48  to  496.40) | 400.90 (338.73  to  463.06) | 364.67 (295.83  to  433.50) | 332.08 (257.03  to  407.14) | 302.67 (222.12  to  383.22) | 276.17 (190.91  to  361.43) | 252.41 (163.15  to  341.68) |
| DALY | 347.42 (281.96  to  412.88) | 303.43 (233.11  to  373.76) | 265.29 (186.79  to  343.80) | 232.22 (144.74  to  319.71) | 203.56 (107.75  to  299.37) | 178.68 (75.85  to  281.51) | 157.07 (48.74  to  265.40) | 138.28 (25.97  to  250.58) | 121.93 (7.05  to  236.81) |
| Mortality | 5.33  (4.38  to  6.29) | 4.63  (3.22  to  6.05) | 4.03  (2.16  to  5.89) | 3.50  (1.25  to  5.76) | 3.05  (0.47  to  5.63) | 2.66  (-0.17  to  5.49) | 2.32  (-0.70  to  5.33) | 2.02  (-1.12  to  5.17) | 1.77  (-1.45  to  4.98) |

DALY, disability-adjusted life years; 95% UI, 95% uncertainty interval.
