# Supplementary material for: The burden and etiologies of diarrhea in Asia and its countries from 1990 to 2021 and the forecast to 2040: analyses informed by the global burden of disease study 2021
Source: Front Public Health. 2025 Aug 6;13:1651315. doi: 10.3389/fpubh.2025.1651315 (PMC12364947; doi:10.3389/fpubh.2025.1651315)
Supplement: Supplementary file 5 [file Table_4.DOCX]

**Table S4** Prediction of diarrheal diseases burden in Asia and Asian countries from 2031 to 2040.

|  | **Age-standardized rate per 100,000 No. (95%UI)** | | | | | | | | | |
| --- | --- | --- | --- | --- | --- | --- | --- | --- | --- | --- |
|  | **2031** | **2032** | **2033** | **2034** | **2035** | **2036** | **2037** | **2038** | **2039** | **2040** |
| **Asia** |  |  |  |  |  |  |  |  |  |  |
| Incidence | 59,308.94 (33,081.97 to 85,535.90) | 59,310.58 (29,424.16 to 89,197.00) | 59,357.05 (25,628.45 to 93,085.66) | 59,470.89 (21,704.34 to 97,237.44) | 59,632.64 (17,638.57 to 101,626.71) | 59,786.24 (13,409.46 to 106,163.02) | 59,908.08 (9,012.78 to 110,803.38) | 60,066.88 (4,468.72 to 115,665.03) | 60,278.86 (-225.27  to 120,782.99) | 60,520.17 (-5,076.61 to 126,116.95) |
| Prevalence | 890.30 (669.10  to 1,111.49) | 889.73 (639.19  to 1,140.26) | 889.70 (608.48  to 1,170.93) | 890.42 (577.10  to 1,203.74) | 891.74 (544.95  to 1,238.54) | 893.25 (511.70  to 1,274.80) | 894.70 (477.22  to 1,312.19) | 896.56 (441.80 to 1,351.32) | 898.94 (405.50  to 1,392.37) | 901.67 (368.21  to 1,435.12) |
| DALY | 308.15 (223.40  to 392.90) | 290.68 (201.18  to 380.18) | 274.50 (180.62  to 368.39) | 259.53 (161.61  to  357.45) | 245.68 (144.04  to 347.33) | 232.85 (127.77 to 337.93) | 220.94 (112.69  to 329.19) | 209.89 (98.71  to 321.07) | 199.64 (85.75  to 313.53) | 190.12 (73.71  to  306.53) |
| Mortality | 8.31  (6.13  to  10.49) | 7.81  (5.49  to  10.14) | 7.35  (4.90  to  9.80) | 6.91  (4.35  to  9.47) | 6.50  (3.84  to  9.17) | 6.12  (3.37  to  8.88) | 5.77  (2.94  to  8.60) | 5.44  (2.54  to  8.34) | 5.13  (2.17  to  8.09) | 4.84  (1.84  to  7.85) |
| **Afghanistan** |  |  |  |  |  |  |  |  |  |  |
| Incidence | 10,511.60 (7,360.82  to  13,662.37) | 9,891.42 (6,541.95  to  13,240.90) | 9,318.39 (5,786.24  to  12,850.55) | 8,788.14 (5,089.05  to  12,487.24) | 8,294.94 (4,444.70  to  12,145.18) | 7,833.62 (3,848.04  to  11,819.20) | 7,401.73 (3,295.74  to  11,507.72) | 6,998.30 (2,785.71  to  11,210.89) | 6,620.35 (2,314.76  to  10,925.94) | 6,264.30 (1,879.61  to  10,648.99) |
| Prevalence | 148.33 (100.31  to  196.36) | 139.23 (88.49  to  189.98) | 130.84 (77.62  to  184.06) | 123.08 (67.63  to  178.52) | 115.88 (58.45  to  173.30) | 109.16 (49.99  to  168.34) | 102.90 (42.19  to  163.60) | 97.05 (35.03  to  159.07) | 91.58 (28.45  to  154.70) | 86.43 (22.40  to  150.46) |
| DALY | 248.24  (-21.34  to  517.82) | 222.97  (-50.90  to  496.84) | 200.36  (-75.48  to  476.20) | 180.10  (-95.65  to  455.86) | 161.96  (-111.92  to  435.84) | 145.70  (-124.77  to  416.17) | 131.13  (-134.65  to  396.91) | 118.08  (-141.95  to  378.10) | 106.37  (-147.04  to  359.77) | 95.87  (-150.23  to  341.96) |
| Mortality | 2.72  (0.31  to  5.13) | 2.44  (-0.01  to  4.89) | 2.19  (-0.28  to  4.65) | 1.96  (-0.50  to  4.42) | 1.76  (-0.68  to  4.20) | 1.57  (-0.83  to  3.98) | 1.41  (-0.94  to  3.77) | 1.27  (-1.03  to  3.56) | 1.13  (-1.09  to  3.36) | 1.02  (-1.14  to  3.17) |
| **Armenia** |  |  |  |  |  |  |  |  |  |  |
| Incidence | 4,567.14  (-481.54  to  9,615.83) | 4,334.47  (-1,125.01  to  9,793.95) | 4,113.81  (-1,729.01  to  9,956.62) | 3,904.53  (-2,293.49  to  10,102.55) | 3,706.15  (-2,818.90  to  10,231.19) | 3,518.06  (-3,305.96  to  10,342.07) | 3,339.51  (-3,755.40  to  10,434.41) | 3,169.74  (-4,167.90  to  10,507.38) | 3,008.33  (-4,544.51  to  10,561.18) | 2,854.97  (-4,886.57  to  10,596.50) |
| Prevalence | 79.17  (-37.48  to  195.82) | 76.26  (-51.91  to  204.43) | 73.45  (-65.88  to  212.78) | 70.75  (-79.35  to  220.85) | 68.15  (-92.31  to  228.61) | 65.64  (-104.73  to  236.01) | 63.22  (-116.58  to  243.02) | 60.88  (-127.86  to  249.62) | 58.62  (-138.55  to  255.79) | 56.44  (-148.66  to  261.54) |
| DALY | 16.06  (-10.93  to  43.06) | 15.07  (-13.56  to  43.71) | 14.16  (-15.97  to  44.29) | 13.31  (-18.16  to  44.79) | 12.53  (-20.16  to  45.21) | 11.80  (-21.97  to  45.56) | 11.11  (-23.60  to  45.83) | 10.48  (-25.07  to  46.03) | 9.89  (-26.38  to  46.15) | 9.33  (-27.55  to  46.21) |
| Mortality | 0.06  (-0.10  to  0.22) | 0.05  (-0.10  to  0.21) | 0.05  (-0.11  to  0.21) | 0.04  (-0.11  to  0.20) | 0.04  (-0.11  to  0.19) | 0.04  (-0.11  to  0.19) | 0.03  (-0.11  to  0.18) | 0.03  (-0.11  to  0.17) | 0.03  (-0.11  to  0.16) | 0.02  (-0.11  to  0.16) |
| **Bahrain** |  |  |  |  |  |  |  |  |  |  |
| Incidence | 24,597.76 (13,371.72  to  35,823.80) | 24,689.01 (11,836.95  to  37,541.07) | 24,785.72 (10,224.21  to  39,347.23) | 24,886.69 (8,534.32  to  41,239.07) | 24,992.43 (6,768.34  to  43,216.52) | 25,105.12 (4,926.89  to  45,283.34) | 25,224.67 (3,009.12  to  47,440.21) | 25,348.57 (1,014.67  to  49,682.47) | 25,474.39  (-1,056.30  to  52,005.07) | 25,602.02  (-3,203.41  to  54,407.45) |
| Prevalence | 397.03 (146.47  to  647.58) | 401.84 (113.41  to  690.27) | 406.77 (78.05  to  735.49) | 411.82 (40.37  to  783.27) | 417.01 (0.34  to  833.68) | 422.35  (-42.09  to  886.78) | 427.83  (-86.97  to  942.64) | 433.45  (-134.36  to  1,001.25) | 439.18  (-184.29  to  1,062.64) | 445.02  (-236.82  to  1,126.86) |
| DALY | 69.09 (21.04  to  117.15) | 68.88 (14.92  to  122.83) | 68.75  (8.61  to  128.88) | 68.70 (2.10  to  135.31) | 68.75  (-4.63  to  142.12) | 68.87  (-11.59  to  149.34) | 69.08  (-18.81  to  156.98) | 69.37  (-26.31  to  165.04) | 69.72  (-34.10  to  173.53) | 70.14  (-42.20  to  182.47) |
| Mortality | 3.15  (-2.08  to  8.37) | 3.44  (-2.70  to  9.57) | 3.75  (-3.41  to  10.91) | 4.08  (-4.23  to  12.39) | 4.42  (-5.17  to  14.02) | 4.79  (-6.22  to  15.80) | 5.16  (-7.40  to  17.73) | 5.56  (-8.71  to  19.82) | 5.96  (-10.15  to  22.07) | 6.38  (-11.73  to  24.48) |
| **Bangladesh** |  |  |  |  |  |  |  |  |  |  |
| Incidence | 33,208.33 (24,993.36  to  41,423.30) | 32,040.44 (23,123.82  to  40,957.06) | 30,989.47 (21,368.44  to  40,610.49) | 30,039.45 (19,712.50  to  40,366.40) | 29,173.55 (18,139.42  to  40,207.68) | 28,375.06 (16,632.94  to  40,117.19) | 27,637.61 (15,184.88  to  40,090.34) | 26,950.46 (13,789.22  to  40,111.70) | 26,301.19 (12,438.18  to  40,164.20) | 25,678.99 (11,124.59  to  40,233.40) |
| Prevalence | 453.42 (329.54  to  577.31) | 436.65 (302.32  to  570.98) | 421.58 (276.78  to  566.38) | 408.00 (252.72  to  563.28) | 395.66 (229.89  to  561.44) | 384.37 (208.09  to  560.65) | 374.00 (187.17  to  560.82) | 364.36 (167.01  to  561.70) | 355.27 (147.52  to  563.02) | 346.59 (128.59  to  564.59) |
| DALY | 451.49 (303.23  to  599.75) | 434.18 (274.23  to  594.14) | 417.98 (246.58  to  589.38) | 402.85 (220.26  to  585.45) | 388.75 (195.18  to  582.33) | 375.58 (171.24  to  579.91) | 363.24 (148.34  to  578.13) | 351.69 (126.43  to  576.94) | 340.91 (105.45  to  576.38) | 330.87 (85.31  to  576.43) |
| Mortality | 19.97 (12.88  to  27.06) | 19.37 (11.64  to  27.10) | 18.80 (10.44  to  27.17) | 18.26 (9.27  to  27.25) | 17.74  (8.13  to  27.35) | 17.25  (7.02  to  27.47) | 16.78  (5.95  to  27.60) | 16.33  (4.91  to  27.75) | 15.90  (3.90  to  27.91) | 15.50  (2.92  to  28.09) |
| **Bhutan** |  |  |  |  |  |  |  |  |  |  |
| Incidence | 61,008.02 (39,365.52  to  82,650.51) | 61,839.87 (36,885.14  to  86,794.60) | 62,741.54 (34,231.06  to  91,252.02) | 63,716.12 (31,398.73  to  96,033.52) | 64,772.36 (28,380.65  to  101,164.07) | 65,927.86 (25,164.11  to  106,691.61) | 67,133.01 (21,706.06  to  112,559.97) | 68,383.82 (18,000.06  to  118,767.58) | 69,670.74 (14,037.39  to  125,304.09) | 70,997.76 (9,809.84  to  132,185.68) |
| Prevalence | 949.04 (522.79  to  1,375.30) | 971.79 (476.97  to  1,466.62) | 996.04 (426.60  to  1,565.48) | 1,022.02 (371.48  to  1,672.55) | 1,049.93 (311.22  to  1,788.64) | 1,079.78 (245.26  to  1,914.31) | 1,111.01 (172.89  to  2,049.13) | 1,143.55 (93.79  to  2,193.32) | 1,177.48 (7.59  to  2,347.38) | 1,212.94  (-86.17  to  2,512.04) |
| DALY | 660.49 (321.53  to  999.45) | 639.97 (268.26  to  1,011.68) | 620.64 (216.46  to  1,024.83) | 602.45 (166.09  to  1,038.82) | 585.41 (117.10  to  1,053.71) | 569.44 (69.41  to  1,069.48) | 554.44 (22.90  to  1,085.99) | 540.28  (-22.49  to  1,103.06) | 526.93  (-66.84  to  1,120.69) | 514.41  (-110.23  to  1,139.05) |
| Mortality | 23.93 (9.45  to  38.40) | 23.18 (7.55  to  38.81) | 22.46 (5.70  to  39.22) | 21.77 (3.91  to  39.62) | 21.10  (2.17  to  40.02) | 20.45  (0.49  to  40.41) | 19.83  (-1.13  to  40.79) | 19.23  (-2.70  to  41.16) | 18.65  (-4.21  to  41.52) | 18.10  (-5.67  to  41.88) |
| **Brunei Darussalam** |  |  |  |  |  |  |  |  |  |  |
| Incidence | 4,856.42 (3,205.02  to  6,507.81) | 4,764.62 (2,918.79  to  6,610.45) | 4,673.40 (2,632.02  to  6,714.77) | 4,583.15 (2,345.82  to  6,820.48) | 4,493.82 (2,060.84  to  6,926.80) | 4,404.95 (1,777.50  to  7,032.41) | 4,316.44 (1,496.34  to  7,136.54) | 4,228.74 (1,218.17  to  7,239.30) | 4,142.15 (943.62  to  7,340.68) | 4,056.62 (673.09  to  7,440.14) |
| Prevalence | 58.13 (30.75  to  85.50) | 55.93 (26.53  to  85.33) | 53.80 (22.46  to  85.14) | 51.74 (18.55  to  84.93) | 49.75 (14.81  to  84.69) | 47.83 (11.24  to  84.42) | 45.97  (7.84  to  84.11) | 44.18  (4.60  to  83.76) | 42.45  (1.53  to  83.37) | 40.78  (-1.38  to  82.94) |
| DALY | 29.11 (14.50  to  43.72) | 28.51 (12.69  to  44.33) | 27.93 (10.90  to  44.95) | 27.35 (9.12  to  45.58) | 26.79  (7.37  to  46.22) | 26.24  (5.63  to  46.85) | 25.71  (3.92  to  47.49) | 25.18  (2.23  to  48.13) | 24.67  (0.58  to  48.76) | 24.17  (-1.06  to  49.39) |
| Mortality | 2.50*10^-8^  (-2.82*10^-5^  to  2.83*10^-5^) | 2.50*10^-8^  (-2.82*10^-5^  to  2.83*10^-5^) | 2.50*10^-8^  (-2.82*10^-5^  to  2.83*10^-5^) | 2.50*10^-8^  (-2.82*10^-5^  to  2.83*10^-5^) | 2.50*10^-8^  (-2.82*10^-5^  to  2.83*10^-5^) | 2.50*10^-8^  (-2.82*10^-5^  to  2.83*10^-5^) | 2.50*10^-8^  (-2.82*10^-5^  to  2.83*10^-5^) | 2.50*10^-8^  (-2.82*10^-5^  to  2.83*10^-5^) | 2.50*10^-8^  (-2.82*10^-5^  to  2.83*10^-5^) | 2.50*10^-8^  (-2.82*10^-5^  to  2.83*10^-5^) |
| **Cambodia** |  |  |  |  |  |  |  |  |  |  |
| Incidence | 40,769.37 (29,348.29  to  52,190.44) | 41,026.85 (27,966.01  to  54,087.69) | 41,336.66 (26,532.03  to  56,141.29) | 41,705.60 (25,047.38  to  58,363.81) | 42,134.27 (23,506.09  to  60,762.45) | 42,607.46 (21,891.02  to  63,323.90) | 43,107.76 (20,185.06  to  66,030.47) | 43,642.80 (18,389.84  to  68,895.75) | 44,218.18 (16,503.82  to  71,932.55) | 44,834.66 (14,521.00  to  75,148.33) |
| Prevalence | 568.09 (383.66  to  752.52) | 570.75 (360.68  to  780.82) | 574.22 (336.94  to  811.51) | 578.57 (312.40  to  844.74) | 583.79 (286.95  to  880.62) | 589.70 (260.37  to  919.03) | 596.12 (232.44  to  959.79) | 603.04 (203.12  to  1,002.96) | 610.51 (172.35  to  1,048.67) | 618.55 (140.05  to  1,097.05) |
| DALY | 233.20 (139.25  to  327.14) | 222.66 (121.59  to  323.73) | 212.89 (104.92  to  320.85) | 203.82 (89.20  to  318.45) | 195.39 (74.33  to  316.46) | 187.55 (60.26  to  314.84) | 180.24 (46.92  to  313.57) | 173.42 (34.26  to  312.58) | 167.04 (22.22  to  311.86) | 161.07 (10.77  to  311.37) |
| Mortality | 6.92  (3.99  to  9.85) | 6.58  (3.45  to  9.72) | 6.26  (2.94  to  9.58) | 5.95  (2.46  to  9.45) | 5.67  (2.00  to  9.33) | 5.39  (1.58  to  9.20) | 5.13  (1.18  to  9.08) | 4.88  (0.81  to  8.96) | 4.65  (0.47  to  8.84) | 4.43  (0.15  to  8.71) |
| **China** |  |  |  |  |  |  |  |  |  |  |
| Incidence | 3,896.81 (1,774.70  to  6,018.93) | 3,758.79 (1,446.89  to  6,070.70) | 3,625.36 (1,129.27  to  6,121.46) | 3,496.42 (822.46  to  6,170.38) | 3,371.86 (526.90  to  6,216.82) | 3,251.49 (242.83  to  6,260.15) | 3,135.06  (-29.54  to  6,299.66) | 3,022.41  (-290.06  to  6,334.88) | 2,913.44  (-538.65  to  6,365.54) | 2,808.08  (-775.32  to  6,391.49) |
| Prevalence | 56.82 (22.51  to  91.13) | 54.78 (17.35  to  92.20) | 52.80 (12.35  to  93.25) | 50.89 (7.53  to  94.26) | 49.06  (2.89  to  95.22) | 47.28  (-1.57  to  96.13) | 45.57  (-5.84  to  96.97) | 43.91  (-9.91  to  97.74) | 42.31  (-13.80  to  98.42) | 40.76  (-17.49  to  99.02) |
| DALY | 11.13  (3.52  to  18.73) | 10.50 (2.38  to  18.61) | 9.93  (1.33  to  18.52) | 9.41  (0.35  to  18.47) | 8.93  (-0.57  to  18.43) | 8.50  (-1.43  to  18.42) | 8.10  (-2.23  to  18.43) | 7.73  (-2.98  to  18.44) | 7.39  (-3.69  to  18.47) | 7.07  (-4.36  to  18.51) |
| Mortality | 0.18  (0.07  to  0.28) | 0.17  (0.05  to  0.28) | 0.16  (0.04  to  0.28) | 0.15  (0.02  to  0.28) | 0.15  (0.01  to  0.28) | 0.14  (0.00  to  0.28) | 0.13  (-0.01  to  0.28) | 0.13  (-0.03  to  0.28) | 0.12  (-0.04  to  0.28) | 0.12  (-0.05  to  0.28) |
| **Cyprus** |  |  |  |  |  |  |  |  |  |  |
| Incidence | 28,722.70 (8,333.94  to  49,111.45) | 28,124.02 (5,317.63  to  50,930.41) | 27,534.16 (2,301.57  to  52,766.75) | 26,953.77  (-704.35  to  54,611.89) | 26,382.83  (-3,691.65  to  56,457.31) | 25,820.88  (-6,652.88  to  58,294.64) | 25,267.51  (-9,581.31  to  60,116.32) | 24,723.24  (-12,471.09  to  61,917.58) | 24,188.57  (-15,317.31  to  63,694.45) | 23,663.33  (-18,115.49  to  65,442.15) |
| Prevalence | 442.92 (135.53  to  750.30) | 430.75 (90.15  to  771.36) | 418.86 (45.42  to  792.30) | 407.24 (1.48  to  813.01) | 395.90  (-41.56  to  833.36) | 384.81  (-83.62  to  853.23) | 373.96  (-124.60  to  872.52) | 363.37  (-164.45  to  891.18) | 353.04  (-203.10  to  909.17) | 342.96  (-240.51  to  926.43) |
| DALY | 64.11 (37.04  to  91.18) | 61.17 (32.09  to  90.25) | 58.38 (27.40  to  89.35) | 55.72 (22.97  to  88.48) | 53.20 (18.79  to  87.61) | 50.79 (14.84  to  86.75) | 48.51 (11.13  to  85.88) | 46.33  (7.64  to  85.01) | 44.25  (4.37  to  84.13) | 42.28  (1.31  to  83.24) |
| Mortality | 2.34  (1.00  to  3.68) | 2.32  (0.86  to  3.78) | 2.30  (0.73  to  3.88) | 2.28  (0.59  to  3.98) | 2.27  (0.45  to  4.08) | 2.25  (0.31  to  4.19) | 2.23  (0.16  to  4.30) | 2.21  (0.02  to  4.41) | 2.20  (-0.13  to  4.52) | 2.18  (-0.28  to  4.63) |
| **Democratic People's Republic of Korea** |  |  |  |  |  |  |  |  |  |  |
| Incidence | 38,645.00 (1,096.98  to  76,193.01) | 37,110.04 (-3,947.12  to  78,167.21) | 35,645.99 (-8,799.43  to  80,091.41) | 34,244.17  (-13,452.68  to  81,941.03) | 32,894.68  (-17,898.78  to  83,688.15) | 31,588.62  (-22,128.62  to  85,305.86) | 30,330.84  (-26,142.58  to  86,804.26) | 29,120.64  (-29,939.57  to  88,180.86) | 27,952.31  (-33,514.71  to  89,419.33) | 26,818.71  (-36,859.81  to  90,497.23) |
| Prevalence | 628.99  (-41.90  to  1,299.88) | 604.17  (-128.94  to  1,337.28) | 580.47  (-212.71  to  1,373.65) | 557.76  (-293.05  to  1,408.58) | 535.87  (-369.80  to  1,441.54) | 514.63  (-442.77  to  1,472.03) | 494.14  (-511.94  to  1,500.23) | 474.40  (-577.29  to  1,526.08) | 455.30  (-638.71  to  1,549.32) | 436.75  (-696.04  to  1,569.53) |
| DALY | 93.47 (9.93  to  177.00) | 90.90  (-1.17  to  182.98) | 88.46  (-12.12  to  189.05) | 86.12  (-22.89  to  195.14) | 83.86  (-33.48  to  201.20) | 81.65  (-43.85  to  207.15) | 79.51  (-54.00  to  213.02) | 77.42  (-63.91  to  218.76) | 75.38  (-73.56  to  224.32) | 73.37  (-82.91  to  229.64) |
| Mortality | 0.32  (0.17  to  0.47) | 0.31  (0.15  to  0.47) | 0.31  (0.13  to  0.48) | 0.30  (0.12  to  0.48) | 0.29  (0.10  to  0.49) | 0.29  (0.08  to  0.49) | 0.28  (0.06  to  0.50) | 0.27  (0.05  to  0.50) | 0.27  (0.03  to  0.50) | 0.26  (0.02  to  0.51) |
| **Georgia** |  |  |  |  |  |  |  |  |  |  |
| Incidence | 4,461.95 (210.87  to  8,713.03) | 4,110.30  (-352.23  to  8,572.83) | 3,786.19  (-849.49  to  8,421.86) | 3,487.55  (-1,285.20  to  8,260.29) | 3,212.41  (-1,663.77  to  8,088.59) | 2,958.85  (-1,989.58  to  7,907.28) | 2,725.04  (-2,266.82  to  7,716.90) | 2,509.37  (-2,499.46  to  7,518.20) | 2,310.50  (-2,691.46  to  7,312.47) | 2,127.18  (-2,846.63  to  7,101.00) |
| Prevalence | 69.51  (-5.58  to  144.60) | 64.12  (-14.74  to  142.98) | 59.14  (-22.83  to  141.11) | 54.55  (-29.91  to  139.00) | 50.31  (-36.04  to  136.66) | 46.39  (-41.30  to  134.08) | 42.77  (-45.75  to  131.29) | 39.43  (-49.45  to  128.32) | 36.35  (-52.48  to  125.17) | 33.50  (-54.90  to  121.89) |
| DALY | 8.19  (-2.69  to  19.08) | 7.36  (-3.68  to  18.40) | 6.62  (-4.49  to  17.74) | 5.97  (-5.15  to  17.09) | 5.38  (-5.68  to  16.45) | 4.86  (-6.10  to  15.82) | 4.39  (-6.41  to  15.20) | 3.98  (-6.64  to  14.59) | 3.60  (-6.79  to  13.99) | 3.26  (-6.87  to  13.40) |
| Mortality | 0.02  (-0.05  to  0.10) | 0.02  (-0.05  to  0.09) | 0.02  (-0.05  to  0.09) | 0.02  (-0.05  to  0.08) | 0.01  (-0.05  to  0.07) | 0.01  (-0.04  to  0.07) | 0.01  (-0.04  to  0.06) | 0.01  (-0.04  to  0.06) | 0.01  (-0.04  to  0.05) | 0.01  (-0.04  to  0.05) |
| **India** |  |  |  |  |  |  |  |  |  |  |
| Incidence | 154,147.70 (104,432.00  to  203,863.40) | 156,797.50 (99,173.18  to  214,421.80) | 159,565.10 (93,436.32  to  225,693.80) | 162,483.40 (87,226.15  to  237,740.60) | 165,537.90 (80,511.02  to  250,564.70) | 168,660.30 (73,229.30  to  264,091.20) | 171,799.30 (65,332.66  to  278,266.00) | 175,036.70 (56,846.66  to  293,226.80) | 178,390.50 (47,758.59  to  309,022.50) | 181,839.10 (38,038.23  to  325,640.00) |
| Prevalence | 2,430.06 (1,792.21  to  3,067.91) | 2,477.66 (1,738.24  to  3,217.09) | 2,527.22 (1,678.30  to  3,376.14) | 2,579.13 (1,612.46  to  3,545.81) | 2,633.43 (1,540.40  to  3,726.46) | 2,689.50 (1,461.32  to  3,917.68) | 2,746.60 (1,374.36  to  4,118.84) | 2,805.43 (1,279.72  to  4,331.14) | 2,866.16 (1,177.20  to  4,555.12) | 2,928.71 (1,066.42  to  4,790.99) |
| DALY | 831.00 (547.39  to  1,114.61) | 790.90 (484.86  to  1,096.95) | 753.28 (425.95  to  1,080.61) | 717.94 (370.50  to  1,065.38) | 684.85 (318.39  to  1,051.30) | 653.89  (269.43  to  1,038.34) | 624.84 (223.37  to  1,026.31) | 597.46 (180.03  to  1,014.90) | 571.63 (139.25  to  1,004.00) | 547.31 (100.92  to  993.71) |
| Mortality | 27.78 (19.17  to  36.38) | 26.13 (16.94  to  35.32) | 24.58 (14.85  to  34.30) | 23.11 (12.92  to  33.31) | 21.74 (11.13  to  32.35) | 20.45  (9.47  to  31.43) | 19.24  (7.94  to  30.54) | 18.10  (6.54  to  29.67) | 17.04  (5.24  to  28.83) | 16.03  (4.06  to  28.01) |
| **Indonesia** |  |  |  |  |  |  |  |  |  |  |
| Incidence | 62,594.00 (47,559.74  to  77,628.25) | 62,679.83 (45,625.02  to  79,734.64) | 62,757.96 (43,594.63  to  81,921.28) | 62,829.36 (41,473.64  to  84,185.08) | 62,895.41 (39,266.44  to  86,524.37) | 62,957.45 (36,976.60  to  88,938.30) | 63,013.23  (34,605.19  to  91,421.27) | 63,062.06 (32,155.19  to  93,968.93) | 63,104.42 (29,629.83  to  96,579.01) | 63,141.35 (27,032.10  to  99,250.60) |
| Prevalence | 918.18 (697.39  to  1,138.96) | 915.48 (666.20  to  1,164.77) | 912.69 (633.87  to  1,191.50) | 909.80 (600.50  to  1,219.11) | 906.85 (566.16  to  1,247.55) | 903.86 (530.92  to  1,276.80) | 900.80 (494.81  to  1,306.79) | 897.65 (457.89  to  1,337.42) | 894.43 (420.20  to  1,368.66) | 891.14 (381.81  to  1,400.47) |
| DALY | 633.78 (447.41  to  820.14) | 611.21 (407.33  to  815.08) | 589.70 (368.72  to  810.68) | 569.16 (331.54  to  806.78) | 549.53 (295.77  to  803.30) | 530.83 (261.42  to  800.25) | 513.03 (228.44  to  797.62) | 496.04 (196.78  to  795.29) | 479.76 (166.38  to  793.15) | 464.18 (137.21  to  791.16) |
| Mortality | 23.36 (17.09  to  29.64) | 22.65 (15.72  to  29.58) | 21.96 (14.39  to  29.54) | 21.29 (13.09  to  29.50) | 20.64 (11.82  to  29.46) | 20.01 (10.59  to  29.43) | 19.40  (9.40  to  29.40) | 18.81  (8.24  to  29.37) | 18.23  (7.13  to  29.34) | 17.68  (6.05  to  29.30) |
| **Iran**  **(Islamic Republic of)** |  |  |  |  |  |  |  |  |  |  |
| Incidence | 16,310.18 (11,337.67  to  21,282.70) | 16,006.56 (10,547.10  to  21,466.03) | 15,731.89 (9,773.40  to  21,690.37) | 15,484.48 (9,015.03  to  21,953.94) | 15,259.25 (8,267.85  to  22,250.65) | 15,047.82 (7,526.29  to  22,569.35) | 14,847.61 (6,788.68  to  22,906.53) | 14,661.27 (6,056.53  to  23,266.02) | 14,487.57 (5,328.86  to  23,646.29) | 14,322.39 (4,603.51  to  24,041.26) |
| Prevalence | 237.22 (162.33  to  312.11) | 232.62 (150.45  to  314.80) | 228.48 (138.85  to  318.11) | 224.76 (127.48  to  322.03) | 221.38 (116.30  to  326.45) | 218.22 (105.21  to  331.22) | 215.23 (94.19  to  336.27) | 212.46 (83.26  to  341.67) | 209.88 (72.40  to  347.37) | 207.44 (61.57  to  353.30) |
| DALY | 38.34 (26.45  to  50.23) | 37.20 (24.25  to  50.16) | 36.23 (22.18  to  50.29) | 35.40 (20.21  to  50.59) | 34.67 (18.31  to  51.02) | 34.01 (16.47  to  51.56) | 33.44 (14.67  to  52.20) | 32.93 (12.90  to  52.95) | 32.47 (11.16  to  53.78) | 32.05  (9.44  to  54.67) |
| Mortality | 0.55  (0.27  to  0.84) | 0.53  (0.23  to  0.83) | 0.51  (0.19  to  0.83) | 0.49  (0.15  to  0.83) | 0.47  (0.11  to  0.83) | 0.45  (0.07  to  0.84) | 0.44  (0.04  to  0.84) | 0.42  (0.01  to  0.84) | 0.41  (-0.03  to  0.84) | 0.39  (-0.06  to  0.84) |
| **Iraq** |  |  |  |  |  |  |  |  |  |  |
| Incidence | 17,672.81 (11,929.67  to  23,415.94) | 17,144.98 (10,827.32  to  23,462.64) | 16,656.14 (9,763.78  to  23,548.51) | 16,201.67  (8,735.66  to  23,667.68) | 15,777.38 (7,739.40  to  23,815.36) | 15,381.70 (6,772.44  to  23,990.97) | 15,012.78 (5,831.91  to  24,193.65) | 14,665.67 (4,914.95  to  24,416.39) | 14,335.54 (4,019.15  to  24,651.93) | 14,019.50 (3,143.00  to  24,895.99) |
| Prevalence | 240.15 (154.33  to  325.97) | 231.69 (138.01  to  325.37) | 223.85 (122.39  to  325.31) | 216.57 (107.42  to  325.72) | 209.79 (93.05  to  326.53) | 203.46 (79.22  to  327.70) | 197.53 (65.88  to  329.19) | 191.96 (52.99  to  330.93) | 186.68 (40.51  to  332.84) | 181.65 (28.43  to  334.86) |
| DALY | 97.13 (61.64  to  132.62) | 92.43 (54.89  to  129.96) | 88.05 (48.59  to  127.51) | 83.98 (42.69  to  125.26) | 80.19 (37.18  to  123.19) | 76.66 (32.03  to  121.29) | 73.38 (27.21  to  119.54) | 70.32 (22.70  to  117.93) | 67.46 (18.47  to  116.45) | 64.79 (14.50  to  115.09) |
| Mortality | 1.75  (0.87  to  2.63) | 1.70  (0.74  to  2.67) | 1.66  (0.61  to  2.71) | 1.62  (0.49  to  2.74) | 1.58  (0.37  to  2.78) | 1.54  (0.25  to  2.82) | 1.50  (0.14  to  2.86) | 1.47  (0.03  to  2.91) | 1.43  (-0.08  to  2.95) | 1.40  (-0.19  to  2.99) |
| **Israel** |  |  |  |  |  |  |  |  |  |  |
| Incidence | 16,902.56 (5,276.92  to  28,528.19) | 16,362.75 (3,536.17  to  29,189.33) | 15,836.87 (1,835.19  to  29,838.55) | 15,325.26 (178.95  to  30,471.56) | 14,827.35  (-1,428.64  to  31,083.34) | 14,341.74  (-2,984.21  to  31,667.69) | 13,868.05  (-4,484.81  to  32,220.91) | 13,407.12  (-5,928.34  to  32,742.58) | 12,959.21  (-7,313.35  to  33,231.77) | 12,523.76  (-8,638.59  to  33,686.11) |
| Prevalence | 263.47 (85.25  to  441.69) | 254.30 (58.56  to  450.05) | 245.39 (32.63  to  458.14) | 236.73 (7.55  to  465.91) | 228.31  (-16.64  to  473.27) | 220.12  (-39.89  to  480.14) | 212.15  (-62.17  to  486.47) | 204.41  (-83.43  to  492.24) | 196.89  (-103.67  to  497.45) | 189.60  (-122.87  to  502.07) |
| DALY | 52.36 (13.89  to  90.83) | 49.83  (8.11  to  91.55) | 47.40  (2.64  to  92.16) | 45.08  (-2.50  to  92.66) | 42.85  (-7.32  to  93.02) | 40.72  (-11.81  to  93.25) | 38.68  (-15.97  to  93.32) | 36.72  (-19.81  to  93.25) | 34.84  (-23.34  to  93.03) | 33.05  (-26.56  to  92.66) |
| Mortality | 1.57  (-0.03  to  3.18) | 1.51  (-0.23  to  3.26) | 1.46  (-0.42  to  3.34) | 1.41  (-0.60  to  3.42) | 1.36  (-0.78  to  3.49) | 1.31  (-0.95  to  3.57) | 1.26  (-1.12  to  3.64) | 1.22  (-1.27  to  3.71) | 1.18  (-1.42  to  3.77) | 1.14  (-1.57  to  3.84) |
| **Japan** |  |  |  |  |  |  |  |  |  |  |
| Incidence | 45,159.20 (17,231.90  to  73,086.50) | 44,526.19 (13,058.47  to  75,993.90) | 43,895.32 (8,827.53  to  78,963.11) | 43,266.59 (4,552.69  to  81,980.48) | 42,639.85 (246.30  to  85,033.41) | 42,015.32  (-4,080.43  to  88,111.07) | 41,393.85  (-8,417.53  to  91,205.22) | 40,776.36  (-12,755.78  to  94,308.49) | 40,162.56  (-17,086.58  to  97,411.70) | 39,552.10  (-21,401.80  to  100,505.99) |
| Prevalence | 732.29 (383.18  to  1,081.40) | 723.58 (330.06  to  1,117.09) | 714.82 (276.01  to  1,153.63) | 706.02 (221.19  to  1,190.85) | 697.18 (165.77  to  1,228.58) | 688.31 (109.89  to  1,266.73) | 679.44 (53.67  to  1,305.20) | 670.56  (-2.75  to  1,343.88) | 661.68  (-59.27  to  1,382.62) | 652.78  (-115.78  to  1,421.34) |
| DALY | 91.00 (44.22  to  137.78) | 89.34 (37.08  to  141.61) | 87.70 (29.91  to  145.49) | 86.07 (22.74  to  149.41) | 84.46 (15.59  to  153.34) | 82.86  (8.46  to  157.26) | 81.28  (1.39  to  161.17) | 79.72  (-5.61  to  165.06) | 78.18  (-12.54  to  168.89) | 76.65  (-19.38  to  172.67) |
| Mortality | 0.62  (-0.05  to  1.29) | 0.62  (-0.14  to  1.38) | 0.61  (-0.24  to  1.46) | 0.61  (-0.33  to  1.55) | 0.60  (-0.43  to  1.64) | 0.60  (-0.53  to  1.73) | 0.59  (-0.64  to  1.82) | 0.59  (-0.74  to  1.92) | 0.59  (-0.85  to  2.02) | 0.58  (-0.96  to  2.12) |
| **Jordan** |  |  |  |  |  |  |  |  |  |  |
| Incidence | 17,238.85 (2,042.52  to  32,435.18) | 16,991.77  (-115.34  to  34,098.89) | 16,755.85  (-2,303.99  to  35,815.68) | 16,534.12  (-4,520.86  to  37,589.10) | 16,326.56  (-6,764.96  to  39,418.09) | 16,129.82  (-9,034.57  to  41,294.21) | 15,941.59  (-11,327.74  to  43,210.92) | 15,762.04  (-13,643.38  to  45,167.46) | 15,592.84  (-15,982.56  to  47,168.23) | 15,433.35  (-18,345.33  to  49,212.03) |
| Prevalence | 244.81  (-30.69  to  520.31) | 241.51  (-68.64  to  551.66) | 238.34  (-107.23  to  583.92) | 235.35  (-146.42  to  617.11) | 232.51  (-186.19  to  651.21) | 229.81  (-226.46  to  686.08) | 227.20  (-267.21  to  721.62) | 224.71  (-308.41  to  757.83) | 222.35  (-350.07  to  794.78) | 220.11  (-392.18  to  832.41) |
| DALY | 38.26 (16.72  to  59.80) | 36.17 (13.33  to  59.00) | 34.20 (10.18  to  58.22) | 32.36  (7.26  to  57.46) | 30.63  (4.55  to  56.70) | 29.00  (2.05  to  55.95) | 27.47  (-0.26  to  55.20) | 26.02  (-2.39  to  54.44) | 24.66  (-4.35  to  53.68) | 23.39  (-6.15  to  52.92) |
| Mortality | 0.70  (0.30  to  1.10) | 0.69  (0.26  to  1.12) | 0.68  (0.21  to  1.15) | 0.68  (0.17  to  1.18) | 0.67  (0.13  to  1.22) | 0.67  (0.09  to  1.25) | 0.67  (0.04  to  1.29) | 0.67  (0.00  to  1.34) | 0.67  (-0.04  to  1.38) | 0.67  (-0.09  yo  1.43) |
| **Kazakhstan** |  |  |  |  |  |  |  |  |  |  |
| Incidence | 4,688.14 (2,523.91  to  6,852.36) | 4,456.12 (2,134.00  to  6,778.25) | 4,238.55 (1,767.30  to  6,709.80) | 4,034.03 (1,422.75  to  6,645.32) | 3,841.42 (1,099.28  to  6,583.56) | 3,659.80 (795.83  to  6,523.77) | 3,488.23 (511.39  to  6,465.06) | 3,325.58 (245.14  to  6,406.02) | 3,170.98  (-3.66  to  6,345.62) | 3,023.78  (-235.71  to  6,283.28) |
| Prevalence | 81.19 (46.35  to  116.02) | 78.09 (40.42  to  115.77) | 75.19 (34.74  to  115.63) | 72.44 (29.31  to  115.58) | 69.85 (24.10  to  115.60) | 67.39 (19.12  to  115.66) | 65.04 (14.33  to  115.76) | 62.80  (9.75  to  115.86) | 60.65  (5.36  to  115.94) | 58.58  (1.17  to  116.00) |
| DALY | 15.00  (-8.42  to  38.42) | 14.43  (-11.14  to  40.01) | 13.94  (-13.82  to  41.69) | 13.49  (-16.46  to  43.45) | 13.10  (-19.08  to  45.28) | 12.74  (-21.68  to  47.17) | 12.41  (-24.26  to  49.08) | 12.10  (-26.80  to  51.01) | 11.81  (-29.31  to  52.93) | 11.53  (-31.77  to  54.83) |
| Mortality | 0.16  (-0.25  to  0.56) | 0.15  (-0.29  to  0.59) | 0.14  (-0.33  to  0.61) | 0.14  (-0.36  to  0.64) | 0.13  (-0.40  to  0.66) | 0.13  (-0.43  to  0.69) | 0.12  (-0.47  to  0.71) | 0.12  (-0.50  to  0.73) | 0.11  (-0.53  to  0.75) | 0.11  (-0.55  to  0.77) |
| **Kuwait** |  |  |  |  |  |  |  |  |  |  |
| Incidence | 13,182.93 (7,523.24  to  18,842.63) | 12,393.39 (6,363.44  to  18,423.34) | 11,655.43 (5,289.90  to  18,020.95) | 10,964.53 (4,298.13  to  17,630.92) | 10,316.94 (3,383.75  to  17,250.13) | 9,710.33 (2,542.71  to  16,877.95) | 9,142.48 (1,770.82  to  16,514.13) | 8,609.84 (1,063.85  to  16,155.82) | 8,109.21 (417.91  to  15,800.51) | 7,638.03  (-170.64  to  15,446.69) |
| Prevalence | 170.95 (85.47  to  256.43) | 159.18 (69.09  to  249.26) | 148.28 (54.19  to  242.37) | 138.18 (40.67  to  235.69) | 128.80 (28.44  to  229.17) | 120.09 (17.39  to  222.79) | 112.00 (7.45  to  216.56) | 104.49  (-1.46  to  210.44) | 97.50  (-9.42  to  204.42) | 90.98  (-16.50  to  198.46) |
| DALY | 25.88  (3.94  to  47.81) | 24.23  (0.97  to  47.49) | 22.70  (-1.74  to  47.15) | 21.29  (-4.21  to  46.79) | 19.97  (-6.45  to  46.39) | 18.75  (-8.48  to  45.97) | 17.61  (-10.31  to  45.53) | 16.56  (-11.96  to  45.07) | 15.57  (-13.44  to  44.58) | 14.65  (-14.76  to  44.05) |
| Mortality | 0.41  (-0.31  to  1.13) | 0.44  (-0.40  to  1.28) | 0.47  (-0.50  to  1.45) | 0.51  (-0.61  to  1.63) | 0.54  (-0.74  to  1.83) | 0.58  (-0.88  to  2.04) | 0.62  (-1.04  to  2.27) | 0.66  (-1.21  to  2.52) | 0.70  (-1.40  to  2.79) | 0.74  (-1.60  to  3.08) |
| **Kyrgyzstan** |  |  |  |  |  |  |  |  |  |  |
| Incidence | 1,990.27 (877.62  to  3,102.92) | 1,752.81 (640.04  to  2,865.59) | 1,544.54 (441.99  to  2,647.09) | 1,361.60 (277.98  to  2,445.21) | 1,200.76 (143.19  to  2,258.34) | 1,059.40 (33.38  to  2,085.41) | 935.10  (-55.15  to  1,925.36) | 825.58  (-125.59  to  1,776.75) | 728.94  (-180.65  to  1,638.53) | 643.63  (-222.70  to  1,509.96) |
| Prevalence | 30.00  (9.94  to  50.07) | 26.41  (6.42  to  46.40) | 23.26  (3.51  to  43.00) | 20.49  (1.15  to  39.84) | 18.07  (-0.77  to  36.90) | 15.93  (-2.30  to  34.16) | 14.06  (-3.50  to  31.62) | 12.41  (-4.43  to  29.24) | 10.95  (-5.12  to  27.02) | 9.67  (-5.62  to  24.95) |
| DALY | 20.54  (-10.91  to  51.99) | 18.22  (-13.43  to  49.88) | 16.19  (-15.41  to  47.80) | 14.41  (-16.93  to  45.75) | 12.85  (-18.05  to  43.76) | 11.48  (-18.86  to  41.81) | 10.27  (-19.38  to  39.92) | 9.20  (-19.68  to  38.07) | 8.25  (-19.79  to  36.29) | 7.41  (-19.75  to  34.56) |
| Mortality | 0.27  (-0.24  to  0.78) | 0.24  (-0.28  to  0.76) | 0.22  (-0.31  to  0.75) | 0.20  (-0.33  to  0.73) | 0.18  (-0.35  to  0.71) | 0.16  (-0.36  to  0.69) | 0.15  (-0.37  to  0.67) | 0.14  (-0.37  to  0.65) | 0.12  (-0.38  to  0.62) | 0.11  (-0.38  to  0.60) |
| **Lao People's**  **Democratic Republic** |  |  |  |  |  |  |  |  |  |  |
| Incidence | 42,878.02 (31,501.21  to  54,254.84) | 42,601.17 (29,791.93  to  55,410.40) | 42,363.70 (28,067.13  to  56,660.28) | 42,166.45 (26,327.71  to  58,005.18) | 42,005.02 (24,569.53  to  59,440.51) | 41,869.55 (22,784.45  to  60,954.64) | 41,755.29 (20,967.89  to  62,542.69) | 41,665.30 (19,122.03  to  64,208.57) | 41,598.43 (17,245.75  to  65,951.10) | 41,549.69 (15,335.55  to  67,763.84) |
| Prevalence | 595.12 (413.98  to  776.27) | 588.64 (386.46  to  790.82) | 582.81 (358.93  to  806.69) | 577.61 (331.40  to  823.83) | 572.98 (303.79  to  842.18) | 568.81 (276.01  to  861.60) | 565.02 (248.01  to  882.03) | 561.62 (219.77  to  903.46) | 558.56 (191.29  to  925.83) | 555.77 (162.50  to  949.05) |
| DALY | 335.86 (211.19  to  460.52) | 313.33 (183.27  to  443.40) | 292.88 (157.96  to  427.81) | 274.25 (134.97  to  413.52) | 257.20 (114.06  to  400.35) | 241.63 (95.02  to  388.24) | 227.40 (77.67  to  377.14) | 214.41 (61.83  to  366.98) | 202.46 (47.34  to  357.58) | 191.44 (34.05  to  348.83) |
| Mortality | 7.64  (4.98  to  10.29) | 7.11  (4.35  to  9.87) | 6.62  (3.77  to  9.47) | 6.17  (3.25  to  9.09) | 5.75  (2.77  to  8.73) | 5.36  (2.33  to  8.39) | 5.00  (1.94  to  8.06) | 4.66  (1.58  to  7.75) | 4.35  (1.26  to  7.45) | 4.06  (0.97  to  7.16) |
| **Lebanon** |  |  |  |  |  |  |  |  |  |  |
| Incidence | 29,428.24 (23,890.10  to  34,966.37) | 29,047.02 (22,873.00  to  35,221.04) | 28,703.52 (21,875.38  to  35,531.67) | 28,395.51 (20,895.95  to  35,895.06) | 28,117.25 (19,929.73  to  36,304.76) | 27,864.96 (18,972.58  to  36,757.34) | 27,634.87 (18,020.57  to  37,249.17) | 27,429.29 (17,075.64  to  37,782.93) | 27,243.34 (16,134.32  to  38,352.36) | 27,071.29 (15,192.36  to  38,950.23) |
| Prevalence | 464.68 (366.97  to  562.38) | 460.60 (351.49  to  569.70) | 457.14 (336.21  to  578.06) | 454.27 (321.12  to  587.43) | 451.92 (306.12  to  597.73) | 450.02 (291.14  to  608.90) | 448.52 (276.11  to  620.92) | 447.43 (261.05  to  633.80) | 446.67 (245.89  to  647.45) | 446.16 (230.56  to  661.76) |
| DALY | 112.57 (75.89  to  149.25) | 110.26 (70.31  to  150.21) | 108.09 (64.84  to  151.34) | 106.05 (59.47  to  152.62) | 104.12 (54.20  to  154.04) | 102.31 (49.03  to  155.60) | 100.62 (43.95  to  157.29) | 99.02 (38.95  to  159.10) | 97.53 (34.03  to  161.03) | 96.12 (29.17  to  163.07) |
| Mortality | 1.60  (0.89  to  2.31) | 1.56  (0.79  to  2.32) | 1.51  (0.70  to  2.33) | 1.47  (0.61  to  2.33) | 1.43  (0.52  to  2.34) | 1.39  (0.43  to  2.35) | 1.35  (0.35  to  2.35) | 1.31  (0.27  to  2.35) | 1.27  (0.19  to  2.36) | 1.24  (0.11  to  2.36) |
| **Malaysia** |  |  |  |  |  |  |  |  |  |  |
| Incidence | 78,733.81 (60,175.04  to  97,292.58) | 79,449.13 (58,312.44  to  100,585.81) | 80,163.63 (56,305.01  to  104,022.25) | 80,880.73 (54,157.55  to  107,603.92) | 81,600.38 (51,871.03  to  111,329.72) | 82,317.02 (49,442.26  to  115,191.78) | 83,026.56 (46,869.45  to  119,183.67) | 83,733.39 (44,157.35  to  123,309.43) | 84,439.38 (41,308.13  to  127,570.63) | 85,143.33 (38,321.54  to  131,965.11) |
| Prevalence | 1,160.91 (856.10  to  1,465.73) | 1,167.38 (821.50  to  1,513.25) | 1,173.79 (784.79  to  1,562.79) | 1,180.18 (746.03  to  1,614.34) | 1,186.55 (705.27  to  1,667.84) | 1,192.83 (662.50  to  1,723.17) | 1,198.97 (617.71  to  1,780.23) | 1,205.03 (571.00  to  1,839.05) | 1,211.01 (522.41  to  1,899.60) | 1,216.90 (471.96  to  1,961.83) |
| DALY | 207.41 (133.23  to  281.59) | 205.82 (123.14  to  288.50) | 204.35 (112.90  to  295.80) | 202.97 (102.49  to  303.45) | 201.70 (91.94  to  311.46) | 200.52 (81.24  to  319.80) | 199.43 (70.38  to  328.48) | 198.43 (59.38  to  337.48) | 197.52 (48.23  to  346.80) | 196.67 (36.92  to  356.43) |
| Mortality | 3.84  (2.34  to  5.34) | 3.77  (2.11  to  5.43) | 3.70  (1.89  to  5.52) | 3.64  (1.67  to  5.61) | 3.57  (1.45  to  5.70) | 3.51  (1.23  to  5.79) | 3.45  (1.01  to  5.89) | 3.39  (0.80  to  5.99) | 3.33  (0.58  to  6.08) | 3.27  (0.37  to  6.18) |
| **Maldives** |  |  |  |  |  |  |  |  |  |  |
| Incidence | 52,263.58 (37,149.44  to  67,377.72) | 52,161.55 (35,220.40  to  69,102.70) | 52,075.98 (33,236.24  to  70,915.71) | 52,007.71 (31,200.08  to  72,815.34) | 51,955.39 (29,112.20  to  74,798.59) | 51,914.28 (26,970.28  to  76,858.29) | 51,880.21 (24,772.83  to  78,987.58) | 51,854.95 (22,522.77  to  81,187.13) | 51,838.40 (20,221.08  to  83,455.72) | 51,828.47 (17,867.23  to  85,789.72) |
| Prevalence | 737.28 (490.24  to  984.31) | 733.16 (459.00  to  1,007.32) | 729.39 (427.19  to  1,031.58) | 725.96 (394.86  to  1,057.06) | 722.85 (362.01  to  1,083.70) | 720.00 (328.59  to  1,111.41) | 717.34 (294.60  to  1,140.07) | 714.86 (260.05  to  1,169.67) | 712.56 (224.97  to  1,200.15) | 710.40 (189.33  to  1,231.47) |
| DALY | 177.04 (53.92  to  300.16) | 172.79 (38.18  to  307.40) | 168.91 (22.64  to  315.19) | 165.39 (7.25  to  323.53) | 162.20  (-8.01  to  332.41) | 159.31  (-23.20  to  341.81) | 156.69  (-38.35  to  351.73) | 154.33  (-53.51  to  362.17) | 152.20  (-68.72  to  373.11) | 150.28  (-83.99  to  384.56) |
| Mortality | 1.68  (-0.94  to  4.31) | 1.54  (-1.11  to  4.19) | 1.40  (-1.26  to  4.06) | 1.27  (-1.38  to  3.93) | 1.15  (-1.48  to  3.78) | 1.04  (-1.56  to  3.64) | 0.94  (-1.62  to  3.50) | 0.84  (-1.67  to  3.36) | 0.76  (-1.71  to  3.23) | 0.69  (-1.73  to  3.11) |
| **Mongolia** |  |  |  |  |  |  |  |  |  |  |
| Incidence | 1,458.05  (-720.02  to  3,636.13) | 1,379.96  (-974.06  to  3,733.99) | 1,306.79  (-1,211.53  to  3,825.10) | 1,238.18  (-1,432.82  to  3,909.19) | 1,173.94  (-1,638.57  to  3,986.45) | 1,113.76  (-1,829.44  to  4,056.96) | 1,057.20  (-2,005.86  to  4,120.27) | 1,003.85  (-2,168.12  to  4,175.82) | 953.50  (-2,316.76  to  4,223.75) | 906.02  (-2,452.59  to  4,264.63) |
| Prevalence | 26.12  (-19.50  to  71.75) | 25.28  (-25.01  to  75.56) | 24.47  (-30.41  to  79.34) | 23.70  (-35.69  to  83.08) | 22.96  (-40.85  to  86.77) | 22.26  (-45.88  to  90.41) | 21.59  (-50.78  to  93.96) | 20.95  (-55.55  to  97.44) | 20.33  (-60.17  to  100.83) | 19.74  (-64.67  to  104.14) |
| DALY | 34.46  (-8.52  to  77.43) | 32.80  (-13.42  to  79.02) | 31.23  (-18.03  to  80.49) | 29.73  (-22.37  to  81.82) | 28.30  (-26.42  to  83.02) | 26.94  (-30.19  to  84.07) | 25.65  (-33.69  to  84.98) | 24.41  (-36.92  to  85.74) | 23.24  (-39.89  to  86.36) | 22.12  (-42.60  to  86.84) |
| Mortality | 0.22  (0.05  to  0.39) | 0.20  (0.03  to  0.37) | 0.18  (0.01  to  0.35) | 0.17  (-0.00  to  0.33) | 0.15  (-0.01  to  0.32) | 0.14  (-0.02  to  0.30) | 0.13  (-0.03  to  0.29) | 0.12  (-0.04  to  0.27) | 0.11  (-0.04  to  0.26) | 0.10  (-0.05  to  0.24) |
| **Myanmar** |  |  |  |  |  |  |  |  |  |  |
| Incidence | 39,467.40  (30,617.09  to  48,317.72) | 39,192.17  (29,238.96  to  49,145.38) | 38,926.25  (27,835.73  to  50,016.77) | 38,668.65  (26,409.46  to  50,927.83) | 38,419.32  (24,962.19  to  51,876.46) | 38,178.42  (23,495.38  to  52,861.45) | 37,945.28  (22,009.68  to  53,880.88) | 37,717.46  (20,505.47  to  54,929.45) | 37,493.33  (18,983.48  to  56,003.18) | 37,272.69  (17,444.90  to  57,100.48) |
| Prevalence | 534.22 (401.31  to  667.13) | 527.73 (379.61  to  675.85) | 521.49 (357.82  to  685.17) | 515.50 (335.97  to  695.03) | 509.73 (314.07  to  705.38) | 504.17 (292.14  to  716.19) | 498.80 (270.17  to  727.44) | 493.61 (248.18  to  739.03) | 488.55 (226.16  to  750.93) | 483.61 (204.13  to  763.10) |
| DALY | 276.66 (159.93  to  393.38) | 259.46 (136.31  to  382.61) | 243.69 (114.66  to  372.73) | 229.23 (94.81  to  363.65) | 215.95 (76.61  to  355.28) | 203.72 (59.90  to  347.54) | 192.44 (44.54  to  340.35) | 182.05 (30.41  to  333.69) | 172.47 (17.40  to  327.53) | 163.61 (5.41  to  321.81) |
| Mortality | 6.99  (3.84  to  10.15) | 6.62  (3.23  to  10.00) | 6.26  (2.66  to  9.87) | 5.93  (2.13  to  9.73) | 5.62  (1.63  to  9.60) | 5.32  (1.17  to  9.47) | 5.04  (0.74  to  9.34) | 4.78  (0.35  to  9.21) | 4.53  (-0.02  to  9.09) | 4.30  (-0.36  to  8.96) |
| **Nepal** |  |  |  |  |  |  |  |  |  |  |
| Incidence | 25,410.31 (10,492.48  to  40,328.14) | 24,053.54 (7,947.70  to  40,159.37) | 22,801.84 (5,571.46  to  40,032.22) | 21,642.21 (3,352.13  to  39,932.28) | 20,562.02 (1,278.95  to  39,845.08) | 19,549.25  (-657.75  to  39,756.25) | 18,600.22  (-2,466.92  to  39,667.36) | 17,708.92  (-4,154.54  to  39,572.38) | 16,867.20  (-5,725.40  to  39,459.79) | 16,066.97  (-7,182.72  to  39,316.67) |
| Prevalence | 334.63 (113.05  to  556.22) | 315.80 (77.48  to  554.11) | 298.45 (44.44  to  552.46) | 282.42 (13.74  to  551.10) | 267.54  (-14.77  to  549.84) | 253.63  (-41.24  to  548.50) | 240.64  (-65.80  to  547.09) | 228.47  (-88.57  to  545.51) | 217.00  (-109.60  to  543.61) | 206.14  (-128.96  to  541.24) |
| DALY | 320.23 (185.97  to  454.49) | 302.72 (158.82  to  446.62) | 286.59 (133.59  to  439.59) | 271.70 (110.14  to  433.25) | 257.91 (88.31  to  427.50) | 245.14 (67.99  to  422.30) | 233.30 (49.03  to  417.58) | 222.27 (31.33  to  413.22) | 211.97 (14.80  to  409.13) | 202.32  (-0.65  to  405.28) |
| Mortality | 13.26  (7.24  to  19.27) | 12.66  (6.15  to  19.17) | 12.09  (5.11  to  19.08) | 11.56  (4.12  to  18.99) | 11.04  (3.19  to  18.90) | 10.56  (2.31  to  18.81) | 10.10  (1.48  to  18.72) | 9.66  (0.69  to  18.63) | 9.25  (-0.04  to  18.54) | 8.85  (-0.74  to  18.44) |
| **Oman** |  |  |  |  |  |  |  |  |  |  |
| Incidence | 27,210.86 (17,836.90  to  36,584.82) | 27,566.87 (16,822.10  to  38,311.63) | 27,945.94 (15,736.51  to  40,155.37) | 28,347.27 (14,576.92  to  42,117.62) | 28,769.98 (13,339.27  to  44,200.69) | 29,211.17 (12,018.03  to  46,404.31) | 29,671.92 (10,609.42  to  48,734.42) | 30,150.43 (9,109.82  to  51,191.03) | 30,645.59 (7,515.52  to  53,775.66) | 31,155.95 (5,822.23  to  56,489.67) |
| Prevalence | 432.33 (248.37  to  616.28) | 440.29 (228.61  to  651.97) | 448.69 (207.17  to  690.21) | 457.52 (183.97  to  731.06) | 466.76 (158.90  to  774.63) | 476.43 (131.85  to  821.01) | 486.53 (102.70  to  870.36) | 497.01 (71.36  to  922.66) | 507.84 (37.70  to  977.98) | 519.02 (1.63  to  1,036.42) |
| DALY | 81.85 (37.82  to  125.88) | 80.53 (31.88  to  129.19) | 79.27 (25.92  to  132.63) | 78.07 (19.96  to  136.18) | 76.92 (14.00  to  139.85) | 75.82  (8.03  to  143.62) | 74.78  (2.06  to  147.49) | 73.77  (-3.90  to  151.45) | 72.81  (-9.86  to  155.49) | 71.89  (-15.81  to  159.60) |
| Mortality | 1.52  (-0.27  to  3.30) | 1.51  (-0.46  to  3.47) | 1.50  (-0.65  to  3.65) | 1.49  (-0.85  to  3.83) | 1.48  (-1.06  to  4.01) | 1.47  (-1.26  to  4.20) | 1.46  (-1.47  to  4.39) | 1.45  (-1.69  to  4.59) | 1.44  (-1.91  to  4.80) | 1.44  (-2.13  to  5.01) |
| **Pakistan** |  |  |  |  |  |  |  |  |  |  |
| Incidence | 80,208.43 (59,657.08  to  100,759.79) | 79,866.54 (56,510.73  to  103,222.36) | 79,518.97 (53,259.34  to  105,778.61) | 79,249.43 (49,968.71  to  108,530.15) | 79,144.73 (46,689.44  to  111,600.02) | 79,162.12 (43,378.69  to  114,945.55) | 79,246.00 (39,984.25  to  118,507.76) | 79,333.02 (36,478.59  to  122,187.45) | 79,474.96 (32,890.24  to  126,059.69) | 79,727.74 (29,236.96  to  130,218.52) |
| Prevalence | 1,182.57 (852.99  to  1,512.14) | 1,176.76 (802.72  to  1,550.80) | 1,171.22 (751.09  to  1,591.35) | 1,167.06 (698.92  to  1,635.20) | 1,165.26 (646.70  to  1,683.82) | 1,164.89 (593.65  to  1,736.13) | 1,165.43 (539.23  to  1,791.62) | 1,166.40 (483.29  to  1,849.52) | 1,168.46 (426.09  to  1,910.82) | 1,172.11 (367.72  to  1,976.51) |
| DALY | 505.61 (364.00  to  647.21) | 472.96 (323.80  to  622.11) | 443.44 (287.29  to  599.59) | 416.54 (253.97  to  579.12) | 391.94 (223.48  to  560.41) | 369.44 (195.54  to  543.34) | 348.92 (169.91  to  527.93) | 330.15 (146.37  to  513.93) | 312.82 (124.65  to  501.00) | 296.76 (104.56  to  488.96) |
| Mortality | 16.32 (11.42  to  21.23) | 15.37 (10.13  to  20.62) | 14.49  (8.94  to  20.04) | 13.66  (7.83  to  19.50) | 12.89  (6.80  to  18.98) | 12.17  (5.85  to  18.48) | 11.50  (4.97  to  18.02) | 10.87  (4.16  to  17.58) | 10.28  (3.41  to  17.15) | 9.72  (2.71  to  16.73) |
| **Palestine** |  |  |  |  |  |  |  |  |  |  |
| Incidence | 8,153.92 (2,172.72  to  14,135.13) | 7,666.80 (1,272.10  to  14,061.49) | 7,214.07 (441.43  to  13,986.72) | 6,793.91  (-322.98  to  13,910.81) | 6,405.54  (-1,025.63  to  13,836.70) | 6,047.27  (-1,671.66  to  13,766.21) | 5,714.26  (-2,265.25  to  13,693.77) | 5,401.96  (-2,808.68  to  13,612.60) | 5,109.16  (-3,304.90  to  13,523.21) | 4,835.69  (-3,757.77  to  13,429.15) |
| Prevalence | 114.34  (-15.03  to  243.70) | 108.20  (-31.48  to  247.89) | 102.46  (-46.86  to  251.79) | 97.12  (-61.21  to  255.45) | 92.15  (-74.59  to  258.90) | 87.53  (-87.07  to  262.13) | 83.18  (-98.65  to  265.02) | 79.09  (-109.38  to  267.56) | 75.24  (-119.30  to  269.78) | 71.63  (-128.47  to  271.74) |
| DALY | 17.91  (6.21  to  29.61) | 16.24  (4.38  to  28.11) | 14.74  (2.78  to  26.71) | 13.40  (1.41  to  25.39) | 12.20  (0.23  to  24.16) | 11.12  (-0.78  to  23.01) | 10.14  (-1.64  to  21.92) | 9.26  (-2.37  to  20.89) | 8.47  (-2.98  to  19.91) | 7.75  (-3.49  to  18.99) |
| Mortality | 0.28  (0.02  to  0.53) | 0.26  (-0.00  to  0.52) | 0.24  (-0.02  to  0.51) | 0.23  (-0.04  to  0.50) | 0.21  (-0.06  to  0.49) | 0.20  (-0.08  to  0.47) | 0.19  (-0.09  to  0.46) | 0.17  (-0.10  to  0.45) | 0.16  (-0.11  to  0.44) | 0.15  (-0.12  to  0.42) |
| **Philippines** |  |  |  |  |  |  |  |  |  |  |
| Incidence | 37,847.95 (26,528.71  to  49,167.18) | 37,679.98 (24,859.50  to  50,500.46) | 37,509.38 (23,135.95  to  51,882.81) | 37,337.06 (21,362.91  to  53,311.20) | 37,163.63 (19,544.36  to  54,782.91) | 36,988.43 (17,682.90  to  56,293.95) | 36,809.63 (15,780.47  to  57,838.79) | 36,626.59 (13,840.22  to  59,412.96) | 36,440.04 (11,865.67  to  61,014.41) | 36,250.76 (9,859.89  to  62,641.63) |
| Prevalence | 552.02 (384.80  to  719.24) | 547.62 (358.80  to  736.44) | 543.21 (332.18  to  754.23) | 538.81 (305.02  to  772.59) | 534.42 (277.38  to  791.46) | 530.03 (249.31  to  810.76) | 525.62 (220.83  to  830.41) | 521.17 (192.00  to  850.33) | 516.69 (162.87  to  870.51) | 512.21 (133.50  to  890.91) |
| DALY | 276.17 (185.55  to  366.79) | 266.15 (167.37  to  364.93) | 256.57 (149.86  to  363.29) | 247.43 (133.01  to  361.84) | 238.68 (116.83  to  360.54) | 230.31 (101.29  to  359.33) | 222.30 (86.39  to  358.20) | 214.63 (72.11  to  357.14) | 207.29 (58.45  to  356.13) | 200.26 (45.37  to  355.15) |
| Mortality | 6.34  (4.01  to  8.66) | 6.14  (3.58  to  8.69) | 5.94  (3.17  to  8.71) | 5.75  (2.77  to  8.74) | 5.57  (2.38  to  8.77) | 5.40  (2.00  to  8.80) | 5.23  (1.63  to  8.82) | 5.06  (1.28  to  8.85) | 4.91  (0.94  to  8.87) | 4.75  (0.61  to  8.89) |
| **Qatar** |  |  |  |  |  |  |  |  |  |  |
| Incidence | 25,654.05 (11,910.83  to  39,397.27) | 26,093.05 (10,216.65  to  41,969.45) | 26,543.86 (8,381.08  to  44,706.65) | 27,005.93 (6,400.87  to  47,610.99) | 27,479.63 (4,272.49  to  50,686.77) | 27,967.29 (1,991.85  to  53,942.73) | 28,469.24  (-446.33  to  57,384.81) | 28,984.22  (-3,046.91  to  61,015.35) | 29,510.58  (-5,814.61  to  64,835.77) | 30,048.41  (-8,754.44  to  68,851.26) |
| Prevalence | 424.33 (139.14  to  709.51) | 436.42 (102.47  to  770.36) | 448.94 (61.83  to  836.05) | 461.94 (17.04  to  906.84) | 475.44  (-32.13  to  983.00) | 489.47  (-85.93  to  1,064.86) | 504.04  (-144.63  to  1,152.72) | 519.18  (-208.52  to  1,246.87) | 534.86  (-277.86  to  1,347.58) | 551.11  (-352.98  to  1,455.19) |
| DALY | 56.01 (21.90  to  90.12) | 56.10 (17.57  to  94.63) | 56.23 (13.06  to  99.39) | 56.40  (8.37  to  104.43) | 56.61  (3.50  to  109.73) | 56.87  (-1.58  to  115.32) | 57.17  (-6.85  to  121.20) | 57.52  (-12.34  to  127.38) | 57.90  (-18.05  to  133.86) | 58.33  (-23.99  to  140.66) |
| Mortality | 0.09  (-0.03  to  0.22) | 0.09  (-0.03  to  0.21) | 0.09  (-0.04  to  0.21) | 0.08  (-0.05  to  0.21) | 0.08  (-0.05  to  0.21) | 0.08  (-0.06  to  0.21) | 0.07  (-0.06  to  0.21) | 0.07  (-0.07  to  0.21) | 0.07  (-0.07  to  0.20) | 0.06  (-0.08  to  0.20) |
| **Republic of Korea** |  |  |  |  |  |  |  |  |  |  |
| Incidence | 6,191.08 (3,209.99  to  9,172.16) | 6,051.51 (2,741.38  to  9,361.65) | 5,914.59 (2,275.40  to  9,553.79) | 5,780.33 (1,813.37  to  9,747.28) | 5,648.70 (1,356.37  to  9,941.03) | 5,519.73 (905.32  to  10,134.13) | 5,393.35 (461.00  to  10,325.69) | 5,269.51 (24.11  to  10,514.91) | 5,148.20  (-404.73  to  10,701.13) | 5,029.40  (-825.02  to  10,883.81) |
| Prevalence | 96.64 (50.92  to  142.35) | 94.49 (43.75  to  145.23) | 92.39 (36.62  to  148.16) | 90.33 (29.55  to  151.10) | 88.30 (22.55  to  154.05) | 86.32 (15.64  to  157.00) | 84.37  (8.82  to  159.92) | 82.46  (2.12  to  162.80) | 80.59  (-4.47  to  165.65) | 78.76  (-10.93  to  168.44) |
| DALY | 31.32 (20.26  to  42.38) | 30.56 (18.39  to  42.73) | 29.82 (16.54  to  43.09) | 29.09 (14.72  to  43.46) | 28.39 (12.93  to  43.84) | 27.70 (11.17  to  44.22) | 27.03  (9.45  to  44.60) | 26.37  (7.77  to  44.98) | 25.74  (6.12  to  45.36) | 25.12  (4.51  to  45.73) |
| Mortality | 1.17  (0.57  to  1.76) | 1.14  (0.48  to  1.80) | 1.12  (0.40  to  1.84) | 1.10  (0.31  to  1.88) | 1.08  (0.23  to  1.92) | 1.05  (0.15  to  1.96) | 1.03  (0.06  to  2.00) | 1.01  (-0.02  to  2.04) | 0.99  (-0.10  to  2.08) | 0.97  (-0.18  to  2.11) |
| **Saudi Arabia** |  |  |  |  |  |  |  |  |  |  |
| Incidence | 34,682.16 (19,230.24  to  50,134.08) | 35,082.71 (17,378.66  to  52,786.77) | 35,512.64 (15,405.07  to  55,620.21) | 35,972.50 (13,305.30  to  58,639.70) | 36,461.13 (11,073.22  to  61,849.05) | 36,974.01 (8,701.17  to  65,246.86) | 37,511.62 (6,182.99  to  68,840.26) | 38,073.67 (3,513.22  to  72,634.11) | 38,659.40 (685.69  to  76,633.12) | 39,266.91  (-2,306.31  to  80,840.12) |
| Prevalence | 525.16 (238.00  to  812.33) | 532.60 (202.61  to  862.59) | 540.56 (164.66  to  916.47) | 549.08 (124.03  to  974.12) | 558.12 (80.59  to  1,035.66) | 567.66 (34.17  to  1,101.14) | 577.68  (-15.39  to  1,170.75) | 588.18  (-68.23  to  1,244.59) | 599.15  (-124.51  to  1,322.80) | 610.55  (-184.40  to  1,405.49) |
| DALY | 105.30 (59.95  to  150.65) | 105.21 (54.32  to  156.10) | 105.32 (48.56  to  162.09) | 105.63 (42.64  to  168.62) | 106.11 (36.54  to  175.68) | 106.75 (30.21  to  183.28) | 107.53 (23.63  to  191.43) | 108.45 (16.78  to  200.13) | 109.50 (9.62  to  209.38) | 110.66 (2.14  to  219.19) |
| Mortality | 2.44  (1.21  to  3.66) | 2.41  (1.05  to  3.77) | 2.39  (0.90  to  3.88) | 2.37  (0.74  to  4.00) | 2.35  (0.58  to  4.12) | 2.33  (0.42  to  4.24) | 2.31  (0.26  to  4.37) | 2.30  (0.09  to  4.50) | 2.28  (-0.08  to  4.64) | 2.27  (-0.25  to  4.78) |
| **Singapore** |  |  |  |  |  |  |  |  |  |  |
| Incidence | 2,006.46 (440.49  to  3,572.42) | 2,118.13 (237.87  to  3,998.38) | 2,237.07 (1.07  to  4,473.07) | 2,363.87  (-273.57  to  5,001.31) | 2,499.20  (-590.19  to  5,588.59) | 2,643.74  (-953.42  to  6,240.89) | 2,797.90  (-1,368.37  to  6,964.16) | 2,962.32  (-1,840.58  to  7,765.23) | 3,137.77  (-2,376.20  to  8,651.74) | 3,325.11  (-2,982.13  to  9,632.35) |
| Prevalence | 23.57  (4.45  to  42.69) | 24.39  (2.05  to  46.72) | 25.24  (-0.61  to  51.10) | 26.14  (-3.58  to  55.85) | 27.08  (-6.85  to  61.01) | 28.06  (-10.47  to  66.58) | 29.08  (-14.45  to  72.61) | 30.16  (-18.81  to  79.12) | 31.28  (-23.59  to  86.15) | 32.45  (-28.81  to  93.72) |
| DALY | 10.83  (0.23  to  21.43) | 10.42  (-1.16  to  21.99) | 10.02  (-2.50  to  22.54) | 9.64  (-3.78  to  23.07) | 9.28  (-5.01  to  23.57) | 8.93  (-6.19  to  24.04) | 8.59  (-7.31  to  24.49) | 8.27  (-8.37  to  24.91) | 7.95  (-9.38  to  25.29) | 7.65  (-10.34  to  25.65) |
| Mortality | 0.44  (0.05  to  0.84) | 0.42  (0.01  to  0.84) | 0.40  (-0.03  to  0.84) | 0.39  (-0.07  to  0.84) | 0.37  (-0.11  to  0.85) | 0.35  (-0.14  to  0.85) | 0.34  (-0.17  to  0.85) | 0.32  (-0.20  to  0.84) | 0.31  (-0.23  to  0.84) | 0.31  (-0.23  to  0.84) |
| **Sri Lanka** |  |  |  |  |  |  |  |  |  |  |
| Incidence | 52,348.94 (40,258.58  to  64,439.31) | 52,790.56 (38,994.79  to  66,586.33) | 53,240.32 (37,644.04  to  68,836.61) | 53,701.31 (36,209.58  to  71,193.05) | 54,173.22 (34,690.91  to  73,655.54) | 54,650.61 (33,083.46  to  76,217.77) | 55,126.97 (31,382.56  to  78,871.38) | 55,606.56 (29,592.16  to  81,620.97) | 56,091.29 (27,713.72  to  84,468.86) | 56,580.07 (25,746.20  to  87,413.93) |
| Prevalence | 741.84 (565.19  to  918.49) | 745.88 (545.03  to  946.74) | 750.04 (523.75  to  976.33) | 754.36 (501.40  to  1,007.31) | 758.83 (478.00  to  1,039.67) | 763.38 (453.46  to  1,073.30) | 767.91 (427.74  to  1,108.07) | 772.46 (400.89  to  1,144.02) | 777.06 (372.95  to  1,181.17) | 781.71 (343.91  to  1,219.51) |
| DALY | 134.79 (77.48  to  192.10) | 132.23 (68.58  to  195.88) | 129.82 (59.73  to  199.90) | 127.57 (50.96  to  204.17) | 125.46 (42.23  to  208.68) | 123.48 (33.56  to  213.40) | 121.61 (24.92  to  218.31) | 119.86 (16.30  to  223.41) | 118.21 (7.72  to  228.70) | 116.67  (-0.84  to  234.18) |
| Mortality | 2.51  (1.17  to  3.85) | 2.41  (0.96  to  3.86) | 2.31  (0.76  to  3.86) | 2.22  (0.57  to  3.87) | 2.13  (0.39  to  3.87) | 2.04  (0.22  to  3.87) | 1.96  (0.05  to  3.87) | 1.88  (-0.11  to  3.87) | 1.81  (-0.25  to  3.87) | 1.73  (-0.39  to  3.86) |
| **Syrian Arab Republic** |  |  |  |  |  |  |  |  |  |  |
| Incidence | 23,803.37 (15,820.62  to  31,786.13) | 23,698.25 (14,736.95  to  32,659.56) | 23,621.17 (13,637.27  to  33,605.07) | 23,571.99 (12,520.55  to  34,623.42) | 23,546.17 (11,382.42  to  35,709.91) | 23,534.81 (10,216.63  to  36,853.00) | 23,535.44 (9,020.91  to  38,049.97) | 23,552.43 (7,796.77  to  39,308.09) | 23,585.91 (6,542.88  to  40,628.93) | 23,631.81 (5,256.20  to  42,007.42) |
| Prevalence | 352.77 (231.51  to  474.03) | 351.65 (215.68  to  487.62) | 350.98 (199.60  to  502.37) | 350.75 (183.23  to  518.26) | 350.88 (166.52  to  535.25) | 351.26 (149.36  to  553.16) | 351.83 (131.71  to  571.96) | 352.66 (113.58  to  591.75) | 353.75 (94.97  to  612.54) | 355.04 (75.80  to  634.29) |
| DALY | 62.54 (39.73  to  85.35) | 61.38 (36.52  to  86.24) | 60.35 (33.38  to  87.32) | 59.45 (30.30  to  88.60) | 58.67 (27.27  to  90.08) | 58.01 (24.28  to  91.73) | 57.44 (21.30  to  93.57) | 56.97 (18.34  to  95.59) | 56.58 (15.37  to  97.80) | 56.29 (12.38  to  100.19) |
| Mortality | 0.85  (0.16  to  1.54) | 0.83  (0.09  to  1.58) | 0.81  (0.01  to  1.62) | 0.80  (-0.07  to  1.66) | 0.78  (-0.15  to  1.71) | 0.76  (-0.22  to  1.75) | 0.75  (-0.30  to  1.80) | 0.73  (-0.37  to  1.84) | 0.72  (-0.45  to  1.89) | 0.71  (-0.52  to  1.93) |
| **Taiwan**  **(Province of China)** |  |  |  |  |  |  |  |  |  |  |
| Incidence | 57,380.20 (352.57  to  114,407.84) | 57,753.44  (-7,256.61  to  122,763.50) | 58,128.39  (-15,289.39  to  131,546.17) | 58,503.15  (-23,737.75  to  140,744.05) | 58,877.53  (-32,595.19  to  150,350.26) | 59,253.98  (-41,858.25  to  160,366.21) | 59,632.52  (-51,523.87  to  170,788.92) | 60,010.53  (-61,586.66  to  181,607.72) | 60,386.00  (-72,041.01  to  192,813.00) | 60,758.77  (-82,883.05  to  204,400.59) |
| Prevalence | 871.87  (-64.54  to  1,808.27) | 877.20  (-190.08  to  1,944.49) | 882.60  (-322.51  to  2,087.71) | 888.04  (-461.69  to  2,237.77) | 893.50  (-607.54  to  2,394.55) | 899.04  (-760.01  to  2,558.08) | 904.64  (-919.06  to  2,728.33) | 910.26  (-1,084.63  to  2,905.14) | 915.87  (-1,256.61  to  3,088.36) | 921.47  (-1,434.97  to  3,277.91) |
| DALY | 92.67  (0.47  to  184.86) | 92.25  (-12.06  to  196.55) | 91.85  (-25.02  to  208.71) | 91.47  (-38.38  to  221.31) | 91.10  (-52.12  to  234.31) | 90.74  (-66.22  to  247.70) | 90.40  (-80.67  to  261.48) | 90.07  (-95.47  to  275.62) | 89.76  (-110.59  to  290.10) | 89.44  (-126.01  to  304.89) |
| Mortality | 0.19  (0.02  to  0.36) | 0.18  (0.00  to  0.36) | 0.18  (-0.01  to  0.37) | 0.17  (-0.03  to  0.37) | 0.16  (-0.05  to  0.38) | 0.16  (-0.07  to  0.38) | 0.15  (-0.08  to  0.39) | 0.15  (-0.10  to  0.39) | 0.14  (-0.11  to  0.39) | 0.14  (-0.12  to  0.40) |
| **Tajikistan** |  |  |  |  |  |  |  |  |  |  |
| Incidence | 12,703.54 (8,179.73  to  17,227.35) | 12,103.55 (7,205.36  to  17,001.74) | 11,543.21 (6,287.53  to  16,798.90) | 11,021.96 (5,424.77  to  16,619.15) | 10,533.58 (4,612.00  to  16,455.16) | 10,069.68 (3,843.60  to  16,295.75) | 9,626.65 (3,116.90  to  16,136.40) | 9,208.45 (2,432.63  to  15,984.27) | 8,814.45 (1,789.03  to  15,839.86) | 8,440.21 (1,183.30  to  15,697.12) |
| Prevalence | 244.57 (140.34  to  348.80) | 235.38 (121.73  to  349.03) | 226.77 (103.90  to  349.63) | 218.71 (86.84  to  350.59) | 211.13 (70.47  to  351.79) | 203.90 (54.73  to  353.06) | 196.96 (39.59  to  354.33) | 190.36 (25.06  to  355.65) | 184.07 (11.13  to  357.00) | 178.03  (-2.23  to  358.29) |
| DALY | 573.50 (252.45  to  894.55) | 545.86 (202.45  to  889.27) | 519.59 (155.15  to  884.03) | 494.62 (110.54  to  878.69) | 470.87 (68.60  to  873.15) | 448.30 (29.28  to  867.31) | 426.82  (-7.49  to  861.12) | 406.39  (-41.77  to  854.56) | 386.97  (-73.64  to  847.58) | 368.49  (-103.20  to  840.18) |
| Mortality | 7.38  (3.43  to  11.33) | 7.11  (2.84  to  11.38) | 6.85  (2.26  to  11.44) | 6.60  (1.70  to  11.49) | 6.35  (1.17  to  11.53) | 6.12  (0.66  to  11.58) | 5.89  (0.18  to  11.61) | 5.68  (-0.29  to  11.65) | 5.47  (-0.73  to  11.67) | 5.27  (-1.15  to  11.69) |
| **Thailand** |  |  |  |  |  |  |  |  |  |  |
| Incidence | 61,847.59 (36,441.21  to  87,253.97) | 63,162.11 (33,554.51  to  92,769.72) | 64,500.22 (30,365.18  to  98,635.25) | 65,864.60 (26,867.30  to  104,861.90) | 67,253.93 (23,051.99  to  111,455.87) | 68,658.80 (18,907.29  to  118,410.31) | 70,069.27 (14,422.40  to  125,716.13) | 71,493.17 (9,593.43  to  133,392.91) | 72,934.76 (4,413.76  to  141,455.77) | 74,392.23  (-1,124.83  to  149,909.28) |
| Prevalence | 897.80 (486.11  to  1,309.48) | 913.52 (435.61  to  1,391.42) | 929.42 (380.56  to  1,478.28) | 945.55 (320.93  to  1,570.16) | 961.85 (256.62  to  1,667.08) | 978.21 (187.54  to  1,768.88) | 994.51 (113.58  to  1,875.44) | 1,010.84 (34.75  to  1,986.94) | 1,027.24  (-49.02  to  2,103.50) | 1,043.67  (-137.77  to  2,225.11) |
| DALY | 278.82 (167.62  to  390.03) | 279.00 (152.30  to  405.70) | 279.25 (136.33  to  422.16) | 279.56 (119.74  to  439.38) | 279.94 (102.53  to  457.36) | 280.39 (84.70  to  476.08) | 280.90 (66.26  to  495.55) | 281.46 (47.20  to  515.72) | 282.05 (27.53  to  536.57) | 282.69 (7.26  to  558.12) |
| Mortality | 6.57  (3.63  to  9.50) | 6.54  (3.22  to  9.86) | 6.52  (2.80  to  10.24) | 6.50  (2.36  to  10.64) | 6.48  (1.91  to  11.05) | 6.46  (1.45  to  11.47) | 6.45  (0.98  to  11.91) | 6.43  (0.50  to  12.37) | 6.42  (0.00  to  12.84) | 6.41  (-0.50  to  13.33) |
| **Timor-Leste** |  |  |  |  |  |  |  |  |  |  |
| Incidence | 46,112.78 (33,107.54  to  59,118.03) | 45,140.90 (30,679.21  to  59,602.60) | 44,229.36 (28,293.30  to  60,165.42) | 43,373.26 (25,948.17  to  60,798.36) | 42,564.33 (23,638.78  to  61,489.87) | 41,795.26 (21,359.94  to  62,230.58) | 41,061.05 (19,107.63  to  63,014.46) | 40,364.98 (16,884.68  to  63,845.28) | 39,700.69 (14,688.45  to  64,712.93) | 39,059.87 (12,515.59  to  65,604.16) |
| Prevalence | 701.89 (479.16  to  924.62) | 687.50 (440.93  to  934.07) | 674.12 (403.37  to  944.86) | 661.63 (366.43  to  956.82) | 649.90 (330.01  to  969.79) | 638.86 (294.04  to  983.69) | 628.47 (258.45  to  998.49) | 618.64 (223.22  to  1,014.05) | 609.25 (188.32  to  1,030.18) | 600.20 (153.69  to  1,046.70) |
| DALY | 476.93 (214.94  to  738.92) | 460.19 (176.09  to  744.28) | 444.63 (138.65  to  750.61) | 430.17 (102.53  to  757.80) | 416.74 (67.65  to  765.83) | 404.26 (33.89  to  774.63) | 392.68 (1.16  to  784.20) | 381.92  (-30.65  to  794.48) | 371.92  (-61.62  to  805.46) | 362.64  (-91.87  to  817.14) |
| Mortality | 12.65  (4.63  to  20.68) | 12.34  (3.63  to  21.06) | 12.04  (2.64  to  21.44) | 11.75  (1.67  to  21.83) | 11.479  (0.72  to  22.23) | 11.20  (-0.22  to  22.62) | 10.94  (-1.13  to  23.01) | 10.68  (-2.03  to  23.39) | 10.43  (-2.91  to  23.78) | 10.19  (-3.77  to  24.15) |
| **Turkey** |  |  |  |  |  |  |  |  |  |  |
| Incidence | 16,638.86 (9,174.66  to  24,103.06) | 16,183.32 (7,965.55  to  24,401.09) | 15,764.70 (6,787.68  to  24,741.73) | 15,379.64 (5,638.03  to  25,121.24) | 15,023.03 (4,512.62  to  25,533.45) | 14,690.81 (3,408.02  to  25,973.60) | 14,380.30 (2,321.60  to  26,439.01) | 14,091.17 (1,252.09  to  26,930.25) | 13,820.25 (197.58  to  27,442.93) | 13,563.67  (-843.52  to  27,970.87) |
| Prevalence | 233.75 (127.73  to  339.77) | 226.84 (110.48  to  343.20) | 220.50 (93.76  to  347.23) | 214.67 (77.51  to  351.83) | 209.28 (61.67  to  356.89) | 204.27 (46.19  to  362.36) | 199.60 (31.02  to  368.17) | 195.24 (16.14  to  374.35) | 191.17 (1.52  to  380.83) | 187.33  (-12.87  to  387.53) |
| DALY | 48.28 (30.19  to  66.36) | 46.19 (27.14  to  65.24) | 44.31 (24.30  to  64.33) | 42.62 (21.64  to  63.60) | 41.09 (19.13  to  63.05) | 39.70 (16.75  to  62.65) | 38.45 (14.49  to  62.41) | 37.31 (12.33  to  62.29) | 36.27 (10.25  to  62.29) | 35.32  (8.24  to  62.40) |
| Mortality | 0.78  (0.45  to  1.11) | 0.75  (0.39  to  1.11) | 0.73  (0.34  to  1.12) | 0.71  (0.30  to  1.13) | 0.69  (0.25  to  1.13) | 0.67  (0.20  to  1.14) | 0.65  (0.16  to  1.15) | 0.63  (0.11  to  1.15) | 0.62  (0.07  to  1.16) | 0.60  (0.03  to  1.17) |
| **Turkmenistan** |  |  |  |  |  |  |  |  |  |  |
| Incidence | 4,101.04 (2,227.58  to  5,974.50) | 3,873.46 (1,872.67  to  5,874.26) | 3,660.95 (1,542.26  to  5,779.63) | 3,462.29 (1,235.14  to  5,689.44) | 3,276.10 (949.90  to  5,602.29) | 3,100.81 (685.13  to  5,516.50) | 2,935.35 (439.68  to  5,431.02) | 2,779.29 (212.78  to  5,345.80) | 2,632.00 (3.54  to  5,260.44) | 2,492.64  (-188.96  to  5,174.25) |
| Prevalence | 76.21 (26.23  to  126.19) | 73.02 (18.86  to  127.18) | 70.01 (11.82  to  128.20) | 67.16  (5.11  to  129.22) | 64.47  (-1.28  to  130.22) | 61.90  (-7.37  to  131.16) | 59.43  (-13.16  to  132.03) | 57.08  (-18.64  to  132.80) | 54.82  (-23.83  to  133.48) | 52.65  (-28.73  to  134.03) |
| DALY | 40.61  (-49.95  to  131.16) | 37.97  (-58.39  to  134.33) | 35.59  (-66.16  to  137.33) | 33.44  (-73.32  to  140.20) | 31.50  (-79.97  to  142.96) | 29.74  (-86.13  to  145.61) | 28.13  (-91.87  to  148.14) | 26.67  (-97.24  to  150.58) | 25.34  (-102.28  to  152.96) | 24.12  (-107.05  to  155.29) |
| Mortality | 0.70  (-0.93  to  2.34) | 0.68  (-1.10  to  2.47) | 0.66  (-1.27  to  2.59) | 0.64  (-1.44  to  2.72) | 0.62  (-1.60  to  2.84) | 0.60  (-1.76  to  2.96) | 0.58  (-1.91  to  3.08) | 0.57  (-2.06  to  3.20) | 0.55  (-2.21  to  3.31) | 0.54  (-2.35  to  3.43) |
| **United Arab Emirates** |  |  |  |  |  |  |  |  |  |  |
| Incidence | 23,759.93 (15,237.44  to  32,282.42) | 23,656.80 (14,023.36  to  33,290.25) | 23,567.93 (12,780.04  to  34,355.83) | 23,494.99 (11,509.10  to  35,480.87) | 23,436.06 (10,209.25  to  36,662.88) | 23,386.26 (8,877.53  to  37,895.00) | 23,342.48 (7,512.31  to  39,172.65) | 23,306.85 (6,115.33  to  40,498.38) | 23,280.83 (4,687.14  to  41,874.52) | 23,262.66 (3,226.84  to  43,298.49) |
| Prevalence | 369.78 (201.09  to  538.47) | 369.70 (178.29  to  561.11) | 369.83 (154.65  to  585.00) | 370.18 (130.18  to  610.17) | 370.72 (104.86  to  636.58) | 371.42 (78.66  to  664.17) | 372.23 (51.54  to  692.91) | 373.17 (23.52  to  722.82) | 374.25  (-5.40  to  753.90) | 375.43  (-35.26  to  786.12) |
| DALY | 55.76 (29.55  to  81.98) | 53.21 (24.95  to  81.47) | 50.82 (20.60  to  81.05) | 48.59 (16.49  to  80.68) | 46.49 (12.61  to  80.36) | 44.53  (8.95  to  80.10) | 42.69  (5.49  to  79.89) | 40.97  (2.22  to  79.72) | 39.35  (-0.87  to  79.58) | 37.82  (-3.80  to  79.45) |
| Mortality | 0.86  (0.04  to  1.69) | 0.82  (-0.04  to  1.67) | 0.77  (-0.11  to  1.65) | 0.73  (-0.18  to  1.63) | 0.68  (-0.24  to  1.60) | 0.64  (-0.29  to  1.58) | 0.60  (-0.34  to  1.55) | 0.57  (-0.38  to  1.52) | 0.53  (-0.42  to  1.49) | 0.50  (-0.45  to  1.45) |
| **Uzbekistan** |  |  |  |  |  |  |  |  |  |  |
| Incidence | 1,820.64 (797.02  to  2,844.25) | 1,702.87 (621.72  to  2,784.02) | 1,593.09 (461.18  to  2,725.00) | 1,490.79 (314.64  to  2,666.93) | 1,395.41 (181.30  to  2,609.51) | 1,306.27 (60.30  to  2,552.23) | 1,222.81  (-49.10  to  2,494.72) | 1,144.63  (-147.58  to  2,436.84) | 1,071.45  (-235.81  to  2,378.71) | 1,002.92  (-314.49  to  2,320.32) |
| Prevalence | 24.16  (8.50  to  39.83) | 22.51  (6.04  to  38.97) | 20.97  (3.82  to  38.12) | 19.54  (1.81  to  37.27) | 18.22  (0.00  to  36.43) | 16.98  (-1.62  to  35.58) | 15.83  (-3.07  to  34.73) | 14.75  (-4.35  to  33.86) | 13.75  (-5.49  to  32.99) | 12.82  (-6.48  to  32.12) |
| DALY | 13.71  (-23.99  to  51.41) | 12.86  (-27.45  to  53.17) | 12.09  (-30.64  to  54.81) | 11.38  (-33.58  to  56.33) | 10.72  (-36.29  to  57.73) | 10.12  (-38.78  to  59.01) | 9.56  (-41.06  to  60.19) | 9.05  (-43.17  to  61.26) | 8.57  (-45.10  to  62.24) | 8.13  (-46.87  to  63.13) |
| Mortality | 0.20  (-0.57  to  0.98) | 0.20  (-0.65  to  1.04) | 0.19  (-0.73  to  1.10) | 0.18  (-0.80  to  1.16) | 0.17  (-0.87  to  1.21) | 0.17  (-0.93  to  1.27) | 0.16  (-1.00  to  1.32) | 0.15  (-1.06  to  1.37) | 0.15  (-1.12  to  1.41) | 0.14  (-1.17  to  1.46) |
| **Viet Nam** |  |  |  |  |  |  |  |  |  |  |
| Incidence | 48,531.20  (38,211.77  to  58,850.63) | 47,998.73 (37,163.76  to  58,833.70) | 47,468.87 (36,136.46  to  58,801.29) | 46,940.08 (35,127.68  to  58,752.48) | 46,410.73 (34,135.35  to  58,686.11) | 45,879.02 (33,157.46  to  58,600.58) | 45,348.97 (32,196.50  to  58,501.44) | 44,819.38 (31,251.50  to  58,387.26) | 44,288.50 (30,321.04  to  58,255.96) | 43,754.73 (29,403.78  to  58,105.68) |
| Prevalence | 691.13 (521.59  to  860.66) | 682.01 (504.51  to  859.51) | 672.98 (487.86  to  858.10) | 664.01 (471.60  to  856.42) | 655.07 (455.68  to  854.45) | 646.13 (440.09  to  852.17) | 637.26 (424.85  to  849.68) | 628.44 (409.94  to  846.94) | 619.64 (395.33  to  843.94) | 610.83 (381.02  to  840.64) |
| DALY | 76.21 (59.13  to  93.29) | 72.90 (55.71  to  90.09) | 69.81 (52.54  to  87.09) | 66.93 (49.61  to  84.24) | 64.22 (46.88  to  81.55) | 61.66 (44.34  to  78.98) | 59.26 (41.98  to  76.55) | 57.00 (39.77  to  74.24) | 54.86 (37.70  to  72.03) | 52.83 (35.75  to  69.91) |
| Mortality | 0.85  (0.70  to  1.00) | 0.79  (0.64  to  0.94) | 0.74  (0.59  to  0.89) | 0.69  (0.54  to  0.83) | 0.64  (0.50  to  0.78) | 0.59  (0.46  to  0.73) | 0.55  (0.42  to  0.69) | 0.51  (0.39  to  0.64) | 0.48  (0.35  to  0.60) | 0.45  (0.33  to  0.57) |
| **Yemen** |  |  |  |  |  |  |  |  |  |  |
| Incidence | 17,704.29 (11,844.58  to  23,563.99) | 16,449.12 (10,340.87  to  22,557.36) | 15,317.90 (8,990.39  to  21,645.42) | 14,297.67 (7,776.36  to  20,818.98) | 13,378.53 (6,683.35  to  20,073.70) | 12,550.19 (5,696.53  to  19,403.84) | 11,804.06 (4,803.53  to  18,804.59) | 11,129.37 (3,992.68  to  18,266.06) | 10,517.44 (3,253.67  to  17,781.20) | 9,960.02 (2,577.06  to  17,342.99) |
| Prevalence | 231.23 (138.56  to  323.90) | 212.42 (116.85  to  307.99) | 195.65 (97.65  to  293.64) | 180.69 (80.66  to  280.71) | 167.35 (65.60  to  269.09) | 155.44 (52.21  to  258.66) | 144.78 (40.27  to  249.30) | 135.21 (29.58  to  240.85) | 126.59 (19.98  to  233.21) | 118.79 (11.33  to  226.26) |
| DALY | 107.71  (-8.53  to  223.95) | 95.32  (-21.23  to  211.87) | 84.50  (-31.47  to  200.47) | 75.05  (-39.62  to  189.71) | 66.77  (-46.01  to  179.55) | 59.53  (-50.94  to  169.99) | 53.17  (-54.63  to  160.98) | 47.58  (-57.30  to  152.47) | 42.66  (-59.13  to  144.44) | 38.31  (-60.25  to  136.88) |
| Mortality | 1.55  (-1.70  to  4.79) | 1.35  (-1.88  to  4.59) | 1.19  (-2.01  to  4.38) | 1.04  (-2.09  to  4.17) | 0.91  (-2.14  to  3.96) | 0.80  (-2.15  to  3.76) | 0.71  (-2.14  to  3.55) | 0.62  (-2.11  to  3.35) | 0.55  (-2.06  to  3.15) | 0.48  (-2.00  to  2.96) |

DALY, disability-adjusted life years; 95% UI, 95% uncertainty interval.
