# Supplementary material for: The burden and etiologies of diarrhea in Asia and its countries from 1990 to 2021 and the forecast to 2040: analyses informed by the global burden of disease study 2021
Source: Front Public Health. 2025 Aug 6;13:1651315. doi: 10.3389/fpubh.2025.1651315 (PMC12364947; doi:10.3389/fpubh.2025.1651315)
Supplement: Supplementary file 6 [file Table_5.DOCX]

**Table S5** Etiologies for the cause of DALY and mortality of diarrhoeal disease in Asia and Asian countries in 1990 and 2021.

|  | **1990** | | | | | | **2021** | | | | | | **Percentage change from 1990 to 2021** | |
| --- | --- | --- | --- | --- | --- | --- | --- | --- | --- | --- | --- | --- | --- | --- |
|  | **ASDR per 100,000** | | | **ASMR per 100,000** | | | **ASDR** **per 100,000** | | | **ASMR** **per 100,000** | | | **ASDR** | **ASMR** |
|  | **val** | **Lower of 95% UI** | **Upper of 95% UI** | **val** | **Lower of 95% UI** | **Upper of 95% UI** | **val** | **Lower of 95% UI** | **Upper of 95% UI** | **val** | **Lower of 95% UI** | **Upper of 95% UI** |  |  |
| ***Adenovirus*** |  |  |  |  |  |  |  |  |  |  |  |  |  |  |
| Asia | 590.61 | 360.35 | 906.92 | 8.31 | 5.30 | 12.12 | 92.25 | 55.13 | 144.00 | 1.34 | 0.83 | 2.06 | -84.38% | -83.92% |
| Afghanistan | 287.76 | 135.46 | 503.88 | 3.25 | 1.55 | 5.63 | 54.17 | 25.73 | 102.14 | 0.60 | 0.29 | 1.14 | -81.18% | -81.37% |
| Armenia | 70.16 | 39.51 | 117.06 | 0.77 | 0.43 | 1.29 | 1.80 | 1.00 | 3.08 | 0.02 | 0.01 | 0.03 | -97.44% | -97.78% |
| Azerbaijan | 145.33 | 79.33 | 241.53 | 1.61 | 0.87 | 2.68 | 22.37 | 10.59 | 42.09 | 0.25 | 0.12 | 0.46 | -84.61% | -84.76% |
| Bahrain | 12.01 | 7.08 | 19.59 | 0.16 | 0.09 | 0.25 | 2.95 | 1.67 | 4.48 | 0.04 | 0.02 | 0.08 | -75.42% | -72.00% |
| Bangladesh | 683.88 | 405.77 | 1,103.39 | 10.25 | 6.03 | 15.62 | 71.67 | 39.53 | 120.29 | 1.48 | 0.71 | 2.98 | -89.52% | -85.58% |
| Bhutan | 873.39 | 315.70 | 1,678.31 | 12.74 | 4.98 | 23.79 | 69.88 | 32.12 | 136.55 | 1.25 | 0.60 | 2.56 | -92.00% | -90.19% |
| Brunei Darussalam | 0.49 | 0.27 | 0.80 | 0.01 | 0.00 | 0.01 | 0.43 | 0.23 | 0.72 | 0.01 | 0.00 | 0.01 | -13.58% | -11.81% |
| Cambodia | 255.28 | 133.64 | 451.14 | 3.35 | 1.91 | 5.74 | 17.96 | 9.51 | 30.42 | 0.27 | 0.15 | 0.44 | -92.96% | -92.05% |
| China | 32.93 | 18.02 | 54.43 | 0.37 | 0.20 | 0.60 | 0.90 | 0.50 | 1.45 | 0.01 | 0.01 | 0.02 | -97.27% | -97.44% |
| Cyprus | 3.35 | 1.68 | 5.97 | 0.04 | 0.02 | 0.08 | 1.36 | 0.67 | 2.23 | 0.02 | 0.01 | 0.03 | -59.39% | -64.08% |
| Democratic People's Republic of Korea | 2.46 | 1.23 | 4.67 | 0.02 | 0.01 | 0.04 | 1.37 | 0.62 | 2.30 | 0.01 | 0.00 | 0.01 | -44.47% | -71.71% |
| Georgia | 40.66 | 22.89 | 68.83 | 0.44 | 0.25 | 0.74 | 1.36 | 0.70 | 2.38 | 0.01 | 0.01 | 0.02 | -96.67% | -97.82% |
| India | 1,451.71 | 895.30 | 2,196.64 | 23.68 | 15.36 | 33.89 | 226.27 | 128.82 | 349.50 | 4.05 | 2.52 | 6.36 | -84.41% | -82.90% |
| Indonesia | 760.28 | 426.06 | 1,244.32 | 10.32 | 5.79 | 16.28 | 71.54 | 42.20 | 114.56 | 1.13 | 0.65 | 1.79 | -90.59% | -89.02% |
| Iran (Islamic Republic of) | 38.44 | 19.80 | 71.05 | 0.43 | 0.22 | 0.80 | 2.39 | 1.28 | 3.70 | 0.03 | 0.02 | 0.04 | -93.77% | -93.61% |
| Iraq | 57.04 | 30.44 | 97.55 | 0.65 | 0.37 | 1.09 | 10.82 | 5.74 | 18.01 | 0.13 | 0.07 | 0.21 | -81.03% | -80.59% |
| Israel | 1.53 | 0.84 | 2.48 | 0.01 | 0.01 | 0.02 | 1.14 | 0.68 | 1.75 | 0.02 | 0.01 | 0.02 | -25.44% | +18.59% |
| Japan | 2.15 | 1.02 | 3.65 | 0.01 | 0.01 | 0.02 | 2.21 | 0.92 | 3.94 | 0.01 | 0.01 | 0.01 | +3.02% | -24.45% |
| Jordan | 13.83 | 7.80 | 23.49 | 0.15 | 0.09 | 0.25 | 3.81 | 2.06 | 6.24 | 0.04 | 0.02 | 0.07 | -72.44% | -73.13% |
| Kazakhstan | 77.41 | 43.03 | 126.86 | 0.85 | 0.48 | 1.40 | 1.67 | 0.91 | 2.85 | 0.01 | 0.01 | 0.03 | -97.84% | -98.25% |
| Kuwait | 6.37 | 3.64 | 9.84 | 0.06 | 0.04 | 0.09 | 1.89 | 0.90 | 3.08 | 0.01 | 0.01 | 0.02 | -70.25% | -79.42% |
| Kyrgyzstan | 102.49 | 56.64 | 166.39 | 1.14 | 0.63 | 1.85 | 6.51 | 3.40 | 11.12 | 0.07 | 0.04 | 0.12 | -93.65% | -93.90% |
| Lao People's Democratic Republic | 1,291.37 | 657.99 | 2,200.95 | 15.98 | 8.53 | 26.71 | 87.12 | 43.37 | 153.00 | 1.14 | 0.61 | 1.87 | -93.25% | -92.87% |
| Lebanon | 22.08 | 10.96 | 38.13 | 0.27 | 0.15 | 0.45 | 7.41 | 3.91 | 12.39 | 0.09 | 0.05 | 0.15 | -66.42% | -67.36% |
| Malaysia | 28.31 | 16.30 | 49.38 | 0.39 | 0.24 | 0.68 | 10.72 | 6.02 | 17.33 | 0.13 | 0.07 | 0.21 | -62.13% | -66.00% |
| Maldives | 460.90 | 247.06 | 782.24 | 5.68 | 3.09 | 9.37 | 21.57 | 12.16 | 36.44 | 0.24 | 0.14 | 0.40 | -95.32% | -95.71% |
| Mongolia | 51.26 | 24.04 | 102.48 | 0.56 | 0.26 | 1.13 | 5.20 | 1.75 | 12.52 | 0.06 | 0.02 | 0.14 | -89.85% | -89.85% |
| Myanmar | 1,632.60 | 686.81 | 3,083.24 | 20.07 | 8.87 | 37.34 | 140.22 | 83.55 | 223.90 | 1.76 | 1.05 | 2.72 | -91.41% | -91.24% |
| Nepal | 303.17 | 166.27 | 511.92 | 4.20 | 2.50 | 6.77 | 19.83 | 10.72 | 34.01 | 0.37 | 0.20 | 0.64 | -93.46% | -91.26% |
| Oman | 12.51 | 5.75 | 22.19 | 0.16 | 0.08 | 0.27 | 2.94 | 1.60 | 4.84 | 0.04 | 0.02 | 0.06 | -76.50% | -76.07% |
| Pakistan | 409.06 | 222.49 | 673.05 | 5.47 | 3.17 | 8.72 | 58.69 | 30.04 | 101.46 | 0.89 | 0.51 | 1.57 | -85.65% | -83.68% |
| Palestine | 29.83 | 15.53 | 53.73 | 0.36 | 0.20 | 0.63 | 2.79 | 1.48 | 4.57 | 0.03 | 0.02 | 0.05 | -90.64% | -91.05% |
| Philippines | 283.30 | 155.68 | 474.53 | 3.41 | 1.97 | 5.73 | 41.89 | 24.58 | 66.30 | 0.53 | 0.32 | 0.81 | -85.22% | -84.53% |
| Qatar | 5.65 | 3.12 | 9.38 | 0.07 | 0.04 | 0.11 | 2.09 | 1.15 | 3.37 | 0.03 | 0.01 | 0.04 | -62.92% | -64.56% |
| Republic of Korea | 0.46 | 0.26 | 0.77 | 0.01 | 0.00 | 0.01 | 0.16 | 0.09 | 0.26 | 0.00 | 0.00 | 0.00 | -66.12% | -64.69% |
| Saudi Arabia | 38.22 | 19.32 | 66.16 | 0.49 | 0.27 | 0.83 | 4.09 | 2.28 | 6.45 | 0.07 | 0.04 | 0.12 | -89.30% | -86.01% |
| Singapore | 0.60 | 0.35 | 0.92 | 0.01 | 0.01 | 0.01 | 0.17 | 0.09 | 0.26 | 0.00 | 0.00 | 0.00 | -72.08% | -66.02% |
| Sri Lanka | 87.33 | 48.39 | 139.76 | 1.36 | 0.81 | 2.13 | 8.76 | 4.94 | 13.98 | 0.12 | 0.06 | 0.20 | -89.96% | -91.42 |
| Syrian Arab Republic | 28.60 | 15.64 | 48.96 | 0.32 | 0.17 | 0.53 | 3.43 | 1.78 | 5.46 | 0.04 | 0.02 | 0.06 | -87.99% | -88.43% |
| Taiwan | 1.89 | 1.11 | 3.05 | 0.02 | 0.01 | 0.03 | 0.96 | 0.42 | 1.71 | 0.00 | 0.00 | 0.00 | -49.13% | -86.60% |
| Tajikistan | 275.30 | 151.95 | 464.72 | 3.06 | 1.68 | 5.18 | 76.95 | 40.45 | 136.39 | 0.86 | 0.45 | 1.52 | -72.05% | -72.03% |
| Thailand | 53.99 | 21.19 | 107.87 | 0.83 | 0.30 | 1.83 | 8.48 | 5.08 | 13.37 | 0.13 | 0.08 | 0.20 | -84.30% | -84.73% |
| Timor-Leste | 850.63 | 391.24 | 1,540.03 | 10.50 | 5.13 | 18.45 | 66.48 | 34.42 | 118.71 | 0.87 | 0.45 | 1.46 | -92.18% | -91.74% |
| Turkey | 108.19 | 56.33 | 196.20 | 1.21 | 0.64 | 2.19 | 8.81 | 4.91 | 14.11 | 0.10 | 0.05 | 0.16 | -91.86% | -92.01% |
| Turkmenistan | 221.57 | 118.62 | 369.28 | 2.46 | 1.32 | 4.09 | 7.22 | 3.64 | 12.52 | 0.08 | 0.04 | 0.13 | -96.74% | -96.85% |
| United Arab Emirates | 9.80 | 5.30 | 16.58 | 0.13 | 0.07 | 0.21 | 3.62 | 2.06 | 5.71 | 0.05 | 0.03 | 0.08 | -63.01% | -60.06% |
| Uzbekistan | 143.17 | 79.69 | 231.11 | 1.58 | 0.89 | 2.56 | 2.67 | 1.55 | 4.33 | 0.03 | 0.02 | 0.05 | -98.14% | -98.23% |
| Viet Nam | 34.46 | 15.64 | 66.28 | 0.46 | 0.21 | 0.84 | 3.13 | 1.60 | 5.01 | 0.03 | 0.01 | 0.05 | -90.91% | -93.86% |
| Yemen | 374.03 | 184.19 | 655.91 | 4.45 | 2.23 | 7.52 | 24.39 | 9.10 | 52.58 | 0.31 | 0.11 | 0.63 | -93.48% | -93.05% |
| ***Aeromonas*** |  |  |  |  |  |  |  |  |  |  |  |  |  |  |
| Asia | 80.35 | 38.19 | 144.70 | 0.94 | 0.44 | 1.69 | 10.41 | 5.17 | 18.83 | 0.12 | 0.06 | 0.22 | -87.05% | -87.22% |
| Afghanistan | 136.44 | 54.89 | 275.69 | 1.53 | 0.61 | 3.10 | 23.10 | 8.93 | 51.13 | 0.26 | 0.10 | 0.57 | -83.07% | -83.30% |
| Armenia | 19.94 | 7.14 | 41.73 | 0.22 | 0.08 | 0.46 | 0.56 | 0.26 | 1.05 | 0.01 | 0.00 | 0.01 | -97.20% | -97.59% |
| Azerbaijan | 34.00 | 12.13 | 74.09 | 0.38 | 0.13 | 0.83 | 5.31 | 1.84 | 11.41 | 0.06 | 0.02 | 0.13 | -84.37% | -84.54% |
| Bahrain | 4.93 | 2.33 | 8.95 | 0.05 | 0.02 | 0.10 | 1.20 | 0.62 | 2.16 | 0.01 | 0.01 | 0.02 | -75.60% | -78.66% |
| Bangladesh | 200.50 | 98.30 | 345.66 | 2.39 | 1.19 | 4.08 | 18.99 | 9.35 | 36.89 | 0.25 | 0.12 | 0.50 | -90.53% | -89.70% |
| Bhutan | 293.67 | 84.36 | 605.83 | 3.51 | 1.05 | 7.21 | 21.36 | 8.02 | 45.52 | 0.27 | 0.10 | 0.56 | -92.73% | -92.38% |
| Brunei Darussalam | 0.17 | 0.09 | 0.30 | 0.00 | 0.00 | 0.00 | 0.14 | 0.07 | 0.26 | 0.00 | 0.00 | 0.00 | -20.15% | -18.28% |
| Cambodia | 61.77 | 27.06 | 119.33 | 0.72 | 0.32 | 1.36 | 4.01 | 1.89 | 7.70 | 0.05 | 0.02 | 0.09 | -93.50% | -93.63% |
| China | 26.93 | 11.72 | 51.39 | 0.30 | 0.13 | 0.57 | 0.59 | 0.29 | 1.09 | 0.01 | 0.00 | 0.01 | -97.80% | -98.03% |
| Cyprus | 0.38 | 0.17 | 0.71 | 0.00 | 0.00 | 0.01 | 0.17 | 0.08 | 0.37 | 0.00 | 0.00 | 0.00 | -54.27% | -70.53% |
| Democratic People's Republic of Korea | 1.42 | 0.69 | 2.64 | 0.01 | 0.01 | 0.03 | 0.91 | 0.41 | 1.85 | 0.00 | 0.00 | 0.01 | -36.02% | -69.80% |
| Georgia | 9.48 | 3.81 | 19.27 | 0.10 | 0.04 | 0.21 | 0.35 | 0.17 | 0.63 | 0.00 | 0.00 | 0.00 | -96.32% | -97.52% |
| India | 100.57 | 48.11 | 185.47 | 1.24 | 0.61 | 2.26 | 10.33 | 5.15 | 19.07 | 0.13 | 0.06 | 0.24 | -89.73% | -89.25% |
| Indonesia | 124.51 | 52.00 | 241.03 | 1.47 | 0.62 | 2.81 | 10.67 | 5.17 | 19.39 | 0.13 | 0.06 | 0.23 | -91.43% | -91.31% |
| Iran (Islamic Republic of) | 19.24 | 8.20 | 40.77 | 0.20 | 0.08 | 0.45 | 1.17 | 0.63 | 2.00 | 0.01 | 0.00 | 0.02 | -93.92% | -95.14% |
| Iraq | 24.64 | 10.44 | 49.67 | 0.27 | 0.11 | 0.54 | 4.74 | 2.15 | 9.02 | 0.05 | 0.02 | 0.10 | -80.74% | -81.56% |
| Israel | 0.19 | 0.09 | 0.36 | 0.00 | 0.00 | 0.00 | 0.14 | 0.07 | 0.25 | 0.00 | 0.00 | 0.00 | -28.10% | -8.75% |
| Japan | 0.31 | 0.15 | 0.63 | 0.00 | 0.00 | 0.00 | 0.32 | 0.14 | 0.69 | 0.00 | 0.00 | 0.00 | +2.03% | -29.98% |
| Jordan | 5.95 | 2.79 | 11.63 | 0.06 | 0.03 | 0.12 | 1.62 | 0.79 | 2.89 | 0.01 | 0.01 | 0.03 | -72.87% | -75.51% |
| Kazakhstan | 19.56 | 8.19 | 38.62 | 0.22 | 0.09 | 0.43 | 0.52 | 0.25 | 0.94 | 0.00 | 0.00 | 0.01 | -97.35% | -97.76% |
| Kuwait | 2.87 | 1.53 | 4.98 | 0.02 | 0.01 | 0.04 | 0.81 | 0.38 | 1.58 | 0.00 | 0.00 | 0.01 | -71.88% | -81.91% |
| Kyrgyzstan | 25.33 | 10.32 | 50.95 | 0.28 | 0.11 | 0.57 | 1.90 | 0.85 | 3.64 | 0.02 | 0.01 | 0.04 | -92.49% | -92.71% |
| Lao People's Democratic Republic | 152.44 | 61.47 | 311.84 | 1.75 | 0.72 | 3.56 | 9.87 | 4.23 | 20.76 | 0.11 | 0.05 | 0.23 | -93.53% | -93.64% |
| Lebanon | 8.78 | 3.62 | 17.60 | 0.09 | 0.04 | 0.20 | 2.94 | 1.35 | 5.53 | 0.03 | 0.01 | 0.06 | -66.53% | -70.86% |
| Malaysia | 3.94 | 1.92 | 7.31 | 0.04 | 0.02 | 0.08 | 1.43 | 0.72 | 2.58 | 0.01 | 0.01 | 0.02 | -63.79% | -74.49% |
| Maldives | 72.81 | 30.76 | 135.03 | 0.84 | 0.35 | 1.55 | 3.08 | 1.54 | 5.50 | 0.03 | 0.01 | 0.06 | -95.77% | -96.37% |
| Mongolia | 13.81 | 5.36 | 28.40 | 0.15 | 0.06 | 0.31 | 1.34 | 0.42 | 3.69 | 0.01 | 0.00 | 0.04 | -90.27% | -90.29% |
| Myanmar | 114.30 | 37.77 | 248.51 | 1.31 | 0.43 | 2.80 | 7.78 | 3.41 | 15.34 | 0.09 | 0.04 | 0.17 | -93.20% | -93.38% |
| Nepal | 200.02 | 92.70 | 359.95 | 2.36 | 1.12 | 4.23 | 10.99 | 5.15 | 21.27 | 0.14 | 0.06 | 0.26 | -94.51% | -94.06% |
| Oman | 8.88 | 3.75 | 19.73 | 0.10 | 0.04 | 0.22 | 1.86 | 0.90 | 3.52 | 0.02 | 0.01 | 0.04 | -79.00% | -80.41% |
| Pakistan | 296.86 | 136.02 | 566.69 | 3.47 | 1.63 | 6.53 | 44.77 | 20.29 | 85.64 | 0.53 | 0.23 | 0.99 | -84.92% | -84.66% |
| Palestine | 10.67 | 4.63 | 21.11 | 0.12 | 0.05 | 0.24 | 1.00 | 0.49 | 1.87 | 0.01 | 0.00 | 0.02 | -90.62% | -91.81% |
| Philippines | 48.93 | 21.45 | 95.15 | 0.55 | 0.24 | 1.07 | 6.70 | 3.15 | 12.20 | 0.07 | 0.03 | 0.13 | -86.31% | -86.57% |
| Qatar | 2.38 | 1.19 | 4.62 | 0.02 | 0.01 | 0.05 | 0.85 | 0.43 | 1.55 | 0.01 | 0.00 | 0.01 | -64.23% | -72.07% |
| Republic of Korea | 0.37 | 0.18 | 0.69 | 0.00 | 0.00 | 0.01 | 0.12 | 0.06 | 0.21 | 0.00 | 0.00 | 0.00 | -67.50% | -70.38% |
| Saudi Arabia | 19.05 | 8.25 | 39.40 | 0.21 | 0.09 | 0.44 | 1.87 | 0.94 | 3.34 | 0.02 | 0.01 | 0.04 | -90.18% | -91.41% |
| Singapore | 0.20 | 0.10 | 0.36 | 0.00 | 0.00 | 0.00 | 0.05 | 0.03 | 0.09 | 0.00 | 0.00 | 0.00 | -73.46% | -72.68% |
| Sri Lanka | 14.00 | 6.49 | 25.17 | 0.17 | 0.08 | 0.30 | 1.23 | 0.64 | 2.20 | 0.01 | 0.01 | 0.02 | -91.21% | -93.29% |
| Syrian Arab Republic | 12.90 | 5.64 | 25.33 | 0.14 | 0.05 | 0.28 | 1.79 | 0.87 | 3.28 | 0.02 | 0.01 | 0.03 | -86.11% | -88.49% |
| Taiwan | 0.86 | 0.47 | 1.47 | 0.01 | 0.00 | 0.01 | 0.43 | 0.17 | 0.97 | 0.00 | 0.00 | 0.00 | -49.36% | -89.84% |
| Tajikistan | 82.66 | 35.98 | 152.48 | 0.92 | 0.40 | 1.71 | 21.59 | 8.63 | 44.86 | 0.24 | 0.09 | 0.50 | -73.88% | -73.89% |
| Thailand | 13.78 | 4.80 | 30.68 | 0.16 | 0.05 | 0.38 | 2.37 | 1.27 | 4.04 | 0.03 | 0.01 | 0.05 | -82.78% | -83.93% |
| Timor-Leste | 132.04 | 50.96 | 258.49 | 1.52 | 0.59 | 2.95 | 9.75 | 4.11 | 19.81 | 0.11 | 0.05 | 0.22 | -92.61% | -92.77% |
| Turkey | 25.81 | 11.37 | 52.04 | 0.28 | 0.12 | 0.57 | 2.17 | 1.10 | 3.73 | 0.02 | 0.01 | 0.04 | -91.61% | -92.70% |
| Turkmenistan | 64.70 | 27.75 | 122.74 | 0.72 | 0.31 | 1.37 | 2.15 | 0.94 | 4.21 | 0.02 | 0.01 | 0.05 | -96.68% | -96.78% |
| United Arab Emirates | 4.44 | 2.07 | 8.42 | 0.05 | 0.02 | 0.09 | 1.53 | 0.76 | 2.73 | 0.01 | 0.01 | 0.03 | -65.58% | -68.21% |
| Uzbekistan | 27.01 | 11.97 | 51.18 | 0.30 | 0.13 | 0.57 | 0.57 | 0.27 | 1.13 | 0.01 | 0.00 | 0.01 | -97.88% | -97.97% |
| Viet Nam | 8.08 | 3.06 | 16.73 | 0.09 | 0.03 | 0.19 | 0.78 | 0.36 | 1.61 | 0.00 | 0.00 | 0.01 | -90.28% | -95.14% |
| Yemen | 164.19 | 70.03 | 359.65 | 1.85 | 0.78 | 4.04 | 10.72 | 3.51 | 26.88 | 0.12 | 0.04 | 0.30 | -93.47% | -93.66% |
| ***Campylobacter*** | |  |  |  |  |  |  |  |  |  |  |  |  |  |
| Asia | 310.75 | 134.51 | 569.04 | 6.63 | 2.22 | 13.97 | 50.99 | 22.65 | 89.54 | 1.19 | 0.33 | 2.80 | -83.59% | -82.02% |
| Afghanistan | 168.26 | 65.32 | 332.89 | 1.93 | 0.76 | 3.77 | 32.62 | 12.19 | 64.08 | 0.37 | 0.13 | 0.73 | -80.62% | -80.87% |
| Armenia | 65.33 | 29.07 | 122.08 | 0.71 | 0.30 | 1.33 | 2.00 | 1.04 | 3.50 | 0.02 | 0.01 | 0.03 | -96.94% | -97.57% |
| Azerbaijan | 67.69 | 28.34 | 134.03 | 0.75 | 0.30 | 1.49 | 10.67 | 4.36 | 22.97 | 0.12 | 0.04 | 0.25 | -84.23% | -84.45% |
| Bahrain | 8.08 | 3.92 | 14.45 | 0.14 | 0.05 | 0.27 | 2.54 | 1.35 | 4.08 | 0.05 | 0.01 | 0.12 | -68.57% | -62.05% |
| Bangladesh | 492.52 | 209.64 | 918.35 | 11.70 | 3.65 | 25.80 | 64.79 | 22.80 | 152.83 | 2.39 | 0.40 | 7.15 | -86.85% | -79.61% |
| Bhutan | 856.53 | 254.20 | 1,936.68 | 20.39 | 5.34 | 49.63 | 85.54 | 29.30 | 190.89 | 2.72 | 0.53 | 7.63 | -90.01% | -86.63% |
| Brunei Darussalam | 0.57 | 0.29 | 0.98 | 0.01 | 0.00 | 0.03 | 0.47 | 0.22 | 0.84 | 0.01 | 0.00 | 0.03 | -17.14% | -14.68% |
| Cambodia | 139.52 | 57.70 | 276.23 | 2.62 | 0.91 | 5.57 | 12.29 | 5.37 | 23.73 | 0.29 | 0.08 | 0.64 | -91.19% | -88.97% |
| China | 14.14 | 5.38 | 28.83 | 0.17 | 0.06 | 0.34 | 0.46 | 0.22 | 0.80 | 0.01 | 0.00 | 0.01 | -96.75% | -96.95% |
| Cyprus | 7.94 | 4.00 | 14.10 | 0.23 | 0.05 | 0.63 | 5.13 | 2.73 | 8.49 | 0.13 | 0.02 | 0.34 | -35.36% | -43.69% |
| Democratic People's Republic of Korea | 1.76 | 0.92 | 3.10 | 0.02 | 0.01 | 0.03 | 2.07 | 1.16 | 3.30 | 0.01 | 0.00 | 0.02 | +17.52% | -61.04% |
| Georgia | 19.76 | 9.40 | 35.56 | 0.21 | 0.09 | 0.38 | 0.88 | 0.49 | 1.42 | 0.01 | 0.00 | 0.01 | -95.56% | -97.51% |
| India | 736.62 | 299.78 | 1,387.82 | 20.68 | 5.48 | 46.95 | 113.76 | 44.04 | 232.07 | 3.99 | 0.90 | 10.63 | -84.56% | -80.70% |
| Indonesia | 251.52 | 99.82 | 480.13 | 5.14 | 1.67 | 11.27 | 28.27 | 12.45 | 54.84 | 0.77 | 0.20 | 1.98 | -88.76% | -85.05% |
| Iran (Islamic Republic of) | 16.70 | 7.18 | 35.36 | 0.21 | 0.08 | 0.43 | 1.40 | 0.78 | 2.26 | 0.02 | 0.01 | 0.05 | -91.60% | -89.68% |
| Iraq | 35.66 | 14.81 | 71.37 | 0.44 | 0.17 | 0.85 | 7.22 | 3.36 | 13.49 | 0.10 | 0.04 | 0.20 | -79.74% | -77.68% |
| Israel | 2.16 | 1.27 | 3.29 | 0.03 | 0.01 | 0.06 | 2.30 | 1.26 | 3.68 | 0.07 | 0.01 | 0.16 | +6.20% | +132.68% |
| Japan | 2.05 | 1.18 | 3.31 | 0.02 | 0.00 | 0.04 | 2.38 | 1.31 | 3.95 | 0.01 | 0.00 | 0.04 | +16.03% | -13.15% |
| Jordan | 7.08 | 3.54 | 13.13 | 0.09 | 0.04 | 0.18 | 2.16 | 1.14 | 3.62 | 0.03 | 0.01 | 0.06 | -69.55% | -68.67% |
| Kazakhstan | 36.11 | 17.26 | 67.77 | 0.40 | 0.18 | 0.75 | 0.97 | 0.52 | 1.56 | 0.01 | 0.00 | 0.02 | -97.33% | -98.07% |
| Kuwait | 4.47 | 2.49 | 7.29 | 0.04 | 0.02 | 0.08 | 1.57 | 0.91 | 2.46 | 0.01 | 0.00 | 0.02 | -64.86% | -74.70% |
| Kyrgyzstan | 48.02 | 22.91 | 86.74 | 0.53 | 0.25 | 0.95 | 3.11 | 1.51 | 5.68 | 0.03 | 0.01 | 0.06 | -93.53% | -94.02% |
| Lao People's Democratic Republic | 499.36 | 196.61 | 952.59 | 7.98 | 2.76 | 15.77 | 41.27 | 17.92 | 79.53 | 0.75 | 0.24 | 1.67 | -91.74% | -90.56% |
| Lebanon | 14.51 | 6.21 | 27.37 | 0.22 | 0.08 | 0.43 | 5.47 | 2.83 | 9.41 | 0.08 | 0.03 | 0.18 | -62.31% | -61.49% |
| Malaysia | 15.57 | 7.21 | 28.58 | 0.33 | 0.10 | 0.82 | 8.26 | 4.51 | 13.05 | 0.16 | 0.04 | 0.39 | -46.93% | -53.24% |
| Maldives | 198.94 | 82.49 | 374.94 | 3.17 | 1.16 | 6.26 | 11.65 | 6.13 | 19.59 | 0.17 | 0.06 | 0.37 | -94.14% | -94.56% |
| Mongolia | 23.44 | 8.98 | 49.61 | 0.25 | 0.09 | 0.54 | 2.41 | 0.79 | 6.59 | 0.03 | 0.01 | 0.07 | -89.73% | -89.74% |
| Myanmar | 403.40 | 155.11 | 906.93 | 6.19 | 2.14 | 13.60 | 33.75 | 14.55 | 62.50 | 0.61 | 0.20 | 1.30 | -91.63% | -90.16% |
| Nepal | 358.86 | 145.21 | 705.96 | 7.80 | 2.50 | 16.79 | 33.14 | 12.70 | 68.85 | 1.09 | 0.23 | 3.00 | -90.77% | -86.04% |
| Oman | 24.38 | 10.77 | 49.41 | 0.37 | 0.13 | 0.79 | 6.59 | 3.52 | 11.08 | 0.10 | 0.03 | 0.24 | -72.97% | -72.33% |
| Pakistan | 1,012.42 | 442.94 | 1,855.60 | 20.32 | 6.55 | 45.00 | 172.56 | 75.37 | 323.47 | 4.35 | 1.06 | 10.59 | -82.96% | -78.58% |
| Palestine | 14.27 | 5.78 | 27.79 | 0.20 | 0.07 | 0.39 | 1.54 | 0.76 | 2.70 | 0.02 | 0.01 | 0.05 | -89.22% | -89.22% |
| Philippines | 126.17 | 54.88 | 255.10 | 1.91 | 0.69 | 3.86 | 20.96 | 10.03 | 38.78 | 0.37 | 0.12 | 0.80 | -83.39% | -80.74% |
| Qatar | 3.96 | 1.93 | 6.91 | 0.07 | 0.02 | 0.14 | 1.87 | 1.06 | 2.94 | 0.03 | 0.01 | 0.07 | -52.73% | -54.66% |
| Republic of Korea | 0.71 | 0.31 | 1.41 | 0.02 | 0.00 | 0.06 | 0.30 | 0.14 | 0.59 | 0.01 | 0.00 | 0.03 | -57.33% | -57.10% |
| Saudi Arabia | 29.12 | 12.48 | 58.35 | 0.48 | 0.17 | 1.01 | 4.30 | 2.18 | 7.28 | 0.11 | 0.02 | 0.27 | -85.25% | -77.90% |
| Singapore | 0.66 | 0.27 | 1.22 | 0.02 | 0.01 | 0.05 | 0.23 | 0.09 | 0.43 | 0.01 | 0.00 | 0.02 | -65.06% | -55.39% |
| Sri Lanka | 47.32 | 19.35 | 92.33 | 1.24 | 0.33 | 3.08 | 6.32 | 3.33 | 10.60 | 0.13 | 0.03 | 0.33 | -86.64% | -89.51% |
| Syrian Arab Republic | 17.99 | 8.33 | 36.14 | 0.22 | 0.08 | 0.45 | 2.60 | 1.33 | 4.35 | 0.04 | 0.01 | 0.08 | -85.56% | -83.70% |
| Taiwan | 2.61 | 1.44 | 4.29 | 0.03 | 0.01 | 0.07 | 2.32 | 1.31 | 3.84 | 0.01 | 0.00 | 0.01 | -10.93% | -82.26% |
| Tajikistan | 103.31 | 45.35 | 191.84 | 1.15 | 0.49 | 2.14 | 28.93 | 11.02 | 55.47 | 0.32 | 0.12 | 0.62 | -72.00% | -72.01% |
| Thailand | 113.00 | 37.74 | 263.65 | 3.13 | 0.64 | 10.16 | 25.26 | 12.17 | 44.99 | 0.56 | 0.15 | 1.39 | -77.65% | -82.08% |
| Timor-Leste | 371.07 | 144.79 | 730.67 | 5.87 | 2.11 | 12.14 | 33.38 | 14.07 | 63.62 | 0.63 | 0.20 | 1.44 | -91.00% | -89.32% |
| Turkey | 36.62 | 14.63 | 73.11 | 0.43 | 0.16 | 0.84 | 3.45 | 1.78 | 6.09 | 0.04 | 0.02 | 0.09 | -90.59% | -89.59% |
| Turkmenistan | 99.16 | 43.47 | 190.00 | 1.10 | 0.47 | 2.12 | 3.45 | 1.66 | 6.36 | 0.04 | 0.02 | 0.07 | -96.52% | -96.71% |
| United Arab Emirates | 6.80 | 3.39 | 12.43 | 0.11 | 0.04 | 0.25 | 3.03 | 1.62 | 4.97 | 0.06 | 0.02 | 0.13 | -55.50% | -51.09% |
| Uzbekistan | 38.28 | 17.40 | 71.07 | 0.42 | 0.19 | 0.79 | 0.83 | 0.40 | 1.45 | 0.01 | 0.00 | 0.02 | -97.84% | -97.96% |
| Viet Nam | 18.63 | 8.10 | 37.38 | 0.38 | 0.10 | 0.94 | 2.77 | 1.51 | 4.37 | 0.04 | 0.01 | 0.10 | -85.15% | -90.25% |
| Yemen | 226.01 | 89.71 | 434.84 | 2.99 | 1.15 | 5.86 | 15.95 | 5.29 | 33.87 | 0.25 | 0.07 | 0.56 | -92.94% | -91.71% |
| ***Vibrio cholerae*** |  |  |  |  |  |  |  |  |  |  |  |  |  |  |
| Asia | 148.34 | 118.45 | 176.65 | 3.53 | 2.81 | 4.20 | 29.72 | 22.13 | 36.60 | 0.68 | 0.52 | 0.84 | -79.96% | -80.73% |
| Afghanistan | 236.77 | 180.86 | 299.07 | 4.22 | 2.80 | 6.30 | 40.30 | 25.30 | 57.32 | 0.77 | 0.45 | 1.18 | -82.98% | -81.71% |
| Armenia | 5.57 | 3.79 | 7.62 | 0.09 | 0.06 | 0.12 | 1.26 | 0.83 | 1.79 | 0.02 | 0.01 | 0.03 | -77.45% | -75.20% |
| Azerbaijan | 11.10 | 7.39 | 15.45 | 0.17 | 0.12 | 0.23 | 4.20 | 2.79 | 5.81 | 0.07 | 0.05 | 0.10 | -62.19% | -57.63% |
| Bahrain | 18.95 | 13.54 | 25.22 | 0.55 | 0.38 | 0.74 | 9.60 | 6.83 | 12.54 | 0.32 | 0.22 | 0.44 | -49.34% | -41.91% |
| Bangladesh | 237.72 | 150.97 | 324.24 | 5.71 | 3.61 | 7.86 | 8.33 | 4.69 | 11.98 | 0.25 | 0.13 | 0.37 | -96.49% | -95.58% |
| Bhutan | 846.81 | 619.18 | 1,145.12 | 22.76 | 17.46 | 30.91 | 48.05 | 34.95 | 65.09 | 1.42 | 1.01 | 1.94 | -94.33% | -93.77% |
| Brunei Darussalam | 0.00 | 0.00 | 0.00 | 0.00 | 0.00 | 0.00 | 0.00 | 0.00 | 0.00 | 0.00 | 0.00 | 0.00 | 0.00 | 0.00 |
| Cambodia | 1,430.88 | 1,122.35 | 1,692.39 | 36.16 | 27.65 | 42.30 | 169.70 | 119.89 | 211.22 | 5.55 | 3.72 | 6.90 | -88.14% | -84.67% |
| China | 1.22 | 0.84 | 1.73 | 0.03 | 0.02 | 0.04 | 0.22 | 0.14 | 0.31 | 0.01 | 0.00 | 0.01 | -82.05% | -77.44% |
| Cyprus | 0.00 | 0.00 | 0.00 | 0.00 | 0.00 | 0.00 | 0.00 | 0.00 | 0.00 | 0.00 | 0.00 | 0.00 | 0.00 | 0.00 |
| Democratic People's Republic of Korea | 0.31 | 0.21 | 0.42 | 0.01 | 0.01 | 0.01 | 0.13 | 0.09 | 0.18 | 0.00 | 0.00 | 0.00 | -57.27% | -51.48% |
| Georgia | 2.32 | 1.51 | 3.24 | 0.04 | 0.02 | 0.05 | 1.00 | 0.67 | 1.36 | 0.02 | 0.01 | 0.03 | -57.08% | -50.38% |
| India | 198.91 | 146.10 | 258.22 | 5.32 | 3.84 | 6.94 | 22.23 | 15.25 | 29.06 | 0.64 | 0.43 | 0.86 | -88.83% | -87.95% |
| Indonesia | 292.95 | 232.56 | 351.53 | 8.61 | 6.83 | 10.31 | 119.26 | 90.70 | 145.73 | 3.60 | 2.66 | 4.50 | -59.29% | -58.19% |
| Iran (Islamic Republic of) | 66.61 | 50.53 | 84.99 | 1.55 | 1.13 | 1.99 | 14.45 | 10.28 | 18.00 | 0.43 | 0.29 | 0.55 | -78.31% | -72.44% |
| Iraq | 34.25 | 24.73 | 45.29 | 0.84 | 0.59 | 1.11 | 7.04 | 4.51 | 9.96 | 0.19 | 0.12 | 0.27 | -79.45% | -77.23% |
| Israel | 0.00 | 0.00 | 0.00 | 0.00 | 0.00 | 0.00 | 0.00 | 0.00 | 0.00 | 0.00 | 0.00 | 0.00 | 0.00 | 0.00 |
| Japan | 0.00 | 0.00 | 0.00 | 0.00 | 0.00 | 0.00 | 0.00 | 0.00 | 0.00 | 0.00 | 0.00 | 0.00 | 0.00 | 0.00 |
| Jordan | 3.63 | 2.46 | 4.91 | 0.09 | 0.06 | 0.12 | 2.09 | 1.35 | 2.93 | 0.06 | 0.04 | 0.09 | -42.51% | -32.10% |
| Kazakhstan | 2.03 | 1.39 | 2.85 | 0.03 | 0.02 | 0.04 | 0.47 | 0.32 | 0.68 | 0.01 | 0.01 | 0.01 | -76.65% | -72.35% |
| Kuwait | 6.14 | 4.03 | 8.86 | 0.15 | 0.10 | 0.21 | 3.40 | 2.44 | 4.37 | 0.10 | 0.07 | 0.13 | -44.68% | -32.91% |
| Kyrgyzstan | 4.03 | 2.72 | 5.52 | 0.06 | 0.04 | 0.08 | 1.22 | 0.81 | 1.72 | 0.02 | 0.01 | 0.03 | -69.81% | -64.04% |
| Lao People's Democratic Republic | 3,141.32 | 2,512.35 | 3,936.53 | 72.47 | 54.39 | 94.58 | 260.19 | 195.16 | 328.80 | 7.94 | 5.78 | 10.30 | -91.72% | -89.04% |
| Lebanon | 88.28 | 62.60 | 118.04 | 1.96 | 1.40 | 2.54 | 27.43 | 19.87 | 35.42 | 0.76 | 0.55 | 0.98 | -68.93% | -61.21% |
| Malaysia | 185.54 | 137.36 | 235.82 | 6.27 | 4.45 | 8.08 | 56.97 | 38.04 | 75.53 | 2.02 | 1.33 | 2.79 | -69.29% | -67.78% |
| Maldives | 836.03 | 660.68 | 997.79 | 23.19 | 18.42 | 27.91 | 54.64 | 39.59 | 68.89 | 1.75 | 1.26 | 2.24 | -93.46% | -92.44% |
| Mongolia | 220.55 | 154.38 | 291.37 | 2.60 | 1.84 | 3.43 | 27.94 | 17.64 | 40.73 | 0.33 | 0.22 | 0.48 | -87.33% | -87.21% |
| Myanmar | 2,039.24 | 1,516.79 | 2,720.44 | 51.39 | 32.38 | 73.19 | 232.00 | 172.55 | 293.52 | 7.05 | 4.66 | 9.13 | -88.62% | -86.29% |
| Nepal | 726.80 | 436.19 | 1,203.57 | 19.89 | 11.60 | 33.94 | 28.88 | 14.60 | 49.31 | 0.84 | 0.43 | 1.54 | -96.03% | -95.76% |
| Oman | 18.68 | 8.82 | 32.77 | 0.53 | 0.24 | 0.95 | 3.22 | 1.34 | 5.74 | 0.10 | 0.04 | 0.18 | -82.79% | -81.40% |
| Pakistan | 298.39 | 212.61 | 387.02 | 7.72 | 5.67 | 9.92 | 103.86 | 71.03 | 140.58 | 2.59 | 1.80 | 3.54 | -65.19% | -66.41% |
| Palestine | 55.45 | 39.97 | 71.56 | 1.42 | 1.02 | 1.80 | 10.89 | 7.24 | 14.27 | 0.32 | 0.20 | 0.44 | -80.35% | -77.42% |
| Philippines | 184.75 | 148.30 | 222.04 | 4.94 | 3.94 | 5.93 | 70.87 | 55.20 | 86.66 | 2.03 | 1.55 | 2.53 | -61.64% | -58.95% |
| Qatar | 3.96 | 2.61 | 5.68 | 0.10 | 0.06 | 0.14 | 2.21 | 1.42 | 3.12 | 0.07 | 0.04 | 0.10 | -44.20% | -31.45% |
| Republic of Korea | 0.00 | 0.00 | 0.00 | 0.00 | 0.00 | 0.00 | 0.00 | 0.00 | 0.00 | 0.00 | 0.00 | 0.00 | 0.00 | 0.00 |
| Saudi Arabia | 11.42 | 8.07 | 15.21 | 0.32 | 0.22 | 0.42 | 2.23 | 1.47 | 3.12 | 0.07 | 0.05 | 0.11 | -80.46% | -76.61% |
| Singapore | 1.84 | 1.56 | 2.12 | 0.04 | 0.03 | 0.04 | 0.00 | 0.00 | 0.00 | 0.00 | 0.00 | 0.00 | -100.00% | -100.00% |
| Sri Lanka | 107.89 | 77.25 | 140.93 | 3.08 | 2.22 | 4.01 | 26.83 | 19.08 | 36.39 | 0.91 | 0.64 | 1.27 | -75.13% | -70.44% |
| Syrian Arab Republic | 44.17 | 32.48 | 57.33 | 1.01 | 0.73 | 1.28 | 10.09 | 7.16 | 12.97 | 0.29 | 0.20 | 0.39 | -77.16% | -70.93% |
| Taiwan (Province of China) | 0.16 | 0.11 | 0.23 | 0.00 | 0.00 | 0.01 | 0.06 | 0.04 | 0.09 | 0.00 | 0.00 | 0.00 | -63.03% | -61.45% |
| Tajikistan | 93.06 | 64.59 | 125.14 | 1.49 | 1.05 | 1.96 | 37.96 | 25.34 | 51.28 | 0.66 | 0.45 | 0.88 | -59.21% | -55.84% |
| Thailand | 153.64 | 105.31 | 221.14 | 4.74 | 3.30 | 6.88 | 41.15 | 27.30 | 58.72 | 1.37 | 0.90 | 1.99 | -73.22% | -71.14% |
| Timor-Leste | 1,318.96 | 1,048.81 | 1,590.09 | 35.66 | 27.34 | 43.98 | 171.03 | 135.69 | 204.15 | 5.17 | 4.02 | 6.37 | -87.03% | -85.50% |
| Turkey | 58.35 | 42.54 | 74.75 | 1.29 | 0.96 | 1.62 | 13.43 | 9.03 | 17.70 | 0.40 | 0.26 | 0.53 | -76.99% | -69.37% |
| Turkmenistan | 57.29 | 38.28 | 76.84 | 0.88 | 0.61 | 1.16 | 12.40 | 8.92 | 15.90 | 0.22 | 0.16 | 0.27 | -78.35% | -75.00% |
| United Arab Emirates | 7.94 | 5.39 | 11.05 | 0.23 | 0.16 | 0.33 | 5.33 | 3.60 | 7.49 | 0.17 | 0.11 | 0.25 | -32.83% | -28.21% |
| Uzbekistan | 3.01 | 1.98 | 4.31 | 0.05 | 0.03 | 0.06 | 0.63 | 0.41 | 0.90 | 0.01 | 0.01 | 0.02 | -79.13% | -75.18% |
| Viet Nam | 221.26 | 166.19 | 268.88 | 6.93 | 4.67 | 8.68 | 22.40 | 15.43 | 28.61 | 0.81 | 0.52 | 1.08 | -89.87% | -88.29% |
| Yemen | 135.08 | 60.43 | 213.17 | 3.45 | 1.55 | 5.34 | 15.41 | 6.53 | 23.98 | 0.42 | 0.18 | 0.65 | -88.59% | -87.71% |
| ***Clostridium difficile*** | |  |  |  |  |  |  |  |  |  |  |  |  |  |
| Asia | 0.95 | 0.68 | 1.27 | 0.04 | 0.03 | 0.06 | 1.08 | 0.82 | 1.42 | 0.06 | 0.04 | 0.07 | +14.05% | +27.64% |
| Afghanistan | 0.42 | 0.23 | 0.67 | 0.01 | 0.01 | 0.02 | 0.51 | 0.29 | 0.79 | 0.02 | 0.01 | 0.03 | +22.81% | +27.15% |
| Armenia | 0.64 | 0.43 | 0.91 | 0.03 | 0.02 | 0.04 | 0.83 | 0.61 | 1.13 | 0.04 | 0.03 | 0.05 | +29.37% | +27.15% |
| Azerbaijan | 1.22 | 0.83 | 1.73 | 0.05 | 0.03 | 0.07 | 1.55 | 1.12 | 2.19 | 0.06 | 0.05 | 0.09 | +26.62% | +27.70% |
| Bahrain | 0.54 | 0.33 | 0.81 | 0.02 | 0.01 | 0.03 | 0.74 | 0.48 | 1.13 | 0.03 | 0.02 | 0.04 | +36.20% | +44.60% |
| Bangladesh | 0.52 | 0.30 | 0.78 | 0.02 | 0.01 | 0.03 | 0.64 | 0.39 | 0.95 | 0.02 | 0.01 | 0.03 | +23.98% | +36.71% |
| Bhutan | 0.45 | 0.27 | 0.68 | 0.02 | 0.01 | 0.02 | 0.64 | 0.39 | 0.93 | 0.02 | 0.01 | 0.04 | +41.40% | +52.30% |
| Brunei Darussalam | 6.33 | 4.85 | 7.82 | 0.22 | 0.17 | 0.29 | 6.53 | 5.00 | 8.19 | 0.26 | 0.20 | 0.33 | +3.11% | +13.84% |
| Cambodia | 0.47 | 0.27 | 0.71 | 0.02 | 0.01 | 0.03 | 0.49 | 0.28 | 0.73 | 0.02 | 0.01 | 0.03 | +4.95% | +5.98% |
| China | 0.42 | 0.25 | 0.63 | 0.02 | 0.01 | 0.02 | 0.50 | 0.31 | 0.73 | 0.02 | 0.01 | 0.03 | +19.20% | +22.28% |
| Cyprus | 4.42 | 3.44 | 5.89 | 0.22 | 0.17 | 0.29 | 5.88 | 4.69 | 7.66 | 0.29 | 0.23 | 0.38 | +33.13% | +34.46% |
| Democratic People's Republic of Korea | 0.74 | 0.48 | 1.06 | 0.03 | 0.02 | 0.04 | 0.89 | 0.59 | 1.28 | 0.04 | 0.03 | 0.06 | +20.67% | +23.54% |
| Georgia | 2.04 | 1.44 | 2.84 | 0.08 | 0.05 | 0.10 | 1.94 | 1.58 | 2.35 | 0.06 | 0.05 | 0.07 | -4.87% | -21.25% |
| India | 0.47 | 0.28 | 0.70 | 0.01 | 0.01 | 0.02 | 0.67 | 0.42 | 0.97 | 0.02 | 0.01 | 0.03 | +44.01% | +56.25% |
| Indonesia | 0.61 | 0.38 | 0.90 | 0.02 | 0.01 | 0.03 | 0.67 | 0.42 | 0.99 | 0.02 | 0.02 | 0.04 | +9.59% | +10.41% |
| Iran (Islamic Republic of) | 0.54 | 0.33 | 0.80 | 0.02 | 0.01 | 0.03 | 0.79 | 0.50 | 1.15 | 0.03 | 0.02 | 0.04 | +45.25% | +55.37% |
| Iraq | 0.45 | 0.27 | 0.69 | 0.02 | 0.01 | 0.02 | 0.69 | 0.44 | 1.01 | 0.03 | 0.02 | 0.04 | +53.40% | +61.55% |
| Israel | 4.45 | 3.45 | 5.71 | 0.22 | 0.16 | 0.29 | 5.61 | 4.32 | 7.18 | 0.27 | 0.21 | 0.36 | +26.08% | +25.19% |
| Japan | 4.69 | 3.59 | 5.91 | 0.18 | 0.14 | 0.24 | 4.73 | 3.93 | 5.73 | 0.21 | 0.16 | 0.27 | +0.91% | +12.28% |
| Jordan | 0.49 | 0.30 | 0.72 | 0.02 | 0.01 | 0.03 | 0.68 | 0.44 | 1.01 | 0.03 | 0.02 | 0.04 | +38.88% | +46.38% |
| Kazakhstan | 1.25 | 0.87 | 1.81 | 0.05 | 0.04 | 0.07 | 1.51 | 1.13 | 2.11 | 0.06 | 0.05 | 0.07 | +20.47% | +15.10% |
| Kuwait | 0.57 | 0.36 | 0.85 | 0.02 | 0.01 | 0.03 | 0.76 | 0.51 | 1.13 | 0.03 | 0.02 | 0.04 | +32.50% | 43.63% |
| Kyrgyzstan | 1.07 | 0.72 | 1.53 | 0.04 | 0.03 | 0.06 | 1.24 | 0.88 | 1.78 | 0.05 | 0.04 | 0.06 | +16.38% | 13.40% |
| Lao People's Democratic Republic | 0.48 | 0.27 | 0.73 | 0.02 | 0.01 | 0.03 | 0.54 | 0.31 | 0.78 | 0.02 | 0.01 | 0.03 | +11.94% | +14.53% |
| Lebanon | 0.45 | 0.28 | 0.65 | 0.02 | 0.01 | 0.02 | 0.66 | 0.44 | 0.97 | 0.03 | 0.02 | 0.04 | +48.69% | +55.95% |
| Malaysia | 0.56 | 0.35 | 0.81 | 0.02 | 0.01 | 0.04 | 0.61 | 0.39 | 0.89 | 0.03 | 0.02 | 0.04 | +8.90% | +9.30% |
| Maldives | 0.42 | 0.25 | 0.62 | 0.02 | 0.01 | 0.03 | 0.56 | 0.34 | 0.81 | 0.02 | 0.01 | 0.03 | +33.39% | +33.06% |
| Mongolia | 1.07 | 0.71 | 1.52 | 0.04 | 0.03 | 0.06 | 0.97 | 0.66 | 1.33 | 0.03 | 0.02 | 0.04 | -9.51% | -34.22% |
| Myanmar | 0.51 | 0.29 | 0.75 | 0.02 | 0.01 | 0.03 | 0.57 | 0.34 | 0.84 | 0.02 | 0.01 | 0.03 | +11.80% | +13.37% |
| Nepal | 0.77 | 0.49 | 1.12 | 0.03 | 0.02 | 0.04 | 0.86 | 0.58 | 1.23 | 0.03 | 0.02 | 0.04 | +11.92% | +23.38% |
| Oman | 0.50 | 0.30 | 0.75 | 0.02 | 0.01 | 0.03 | 0.84 | 0.55 | 1.23 | 0.03 | 0.02 | 0.05 | +68.02% | +77.14% |
| Pakistan | 0.63 | 0.38 | 0.93 | 0.02 | 0.01 | 0.03 | 0.79 | 0.50 | 1.15 | 0.03 | 0.02 | 0.04 | +24.85% | +34.39% |
| Palestine | 0.36 | 0.21 | 0.56 | 0.01 | 0.01 | 0.02 | 0.57 | 0.35 | 0.85 | 0.02 | 0.01 | 0.03 | +56.36% | +66.47% |
| Philippines | 0.70 | 0.45 | 1.04 | 0.03 | 0.02 | 0.04 | 0.66 | 0.42 | 0.98 | 0.02 | 0.02 | 0.04 | -5.89% | -5.51% |
| Qatar | 0.61 | 0.37 | 0.89 | 0.02 | 0.02 | 0.03 | 0.85 | 0.57 | 1.22 | 0.03 | 0.02 | 0.05 | +40.15% | +45.98% |
| Republic of Korea | 6.35 | 4.73 | 8.49 | 0.21 | 0.16 | 0.27 | 6.70 | 4.99 | 8.59 | 0.26 | 0.19 | 0.33 | +5.55% | +21.86% |
| Saudi Arabia | 0.53 | 0.33 | 0.79 | 0.02 | 0.01 | 0.03 | 0.88 | 0.58 | 1.28 | 0.03 | 0.02 | 0.05 | +66.22% | +73.84% |
| Singapore | 5.69 | 4.30 | 7.37 | 0.20 | 0.15 | 0.26 | 5.28 | 4.38 | 6.34 | 0.23 | 0.18 | 0.29 | -7.14% | +13.03% |
| Sri Lanka | 0.55 | 0.33 | 0.81 | 0.02 | 0.01 | 0.03 | 0.57 | 0.36 | 0.82 | 0.02 | 0.01 | 0.03 | +3.86% | +4.11% |
| Syrian Arab Republic | 0.42 | 0.26 | 0.64 | 0.02 | 0.01 | 0.02 | 0.63 | 0.41 | 0.95 | 0.02 | 0.02 | 0.04 | +49.64% | +61.43% |
| Taiwan (Province of China) | 1.82 | 1.33 | 2.55 | 0.07 | 0.05 | 0.10 | 2.56 | 2.10 | 3.08 | 0.11 | 0.09 | 0.14 | +40.89% | +58.10% |
| Tajikistan | 1.08 | 0.73 | 1.54 | 0.04 | 0.03 | 0.06 | 1.26 | 0.87 | 1.76 | 0.05 | 0.04 | 0.08 | +16.29% | +23.02% |
| Thailand | 0.54 | 0.33 | 0.80 | 0.02 | 0.01 | 0.03 | 0.56 | 0.35 | 0.83 | 0.02 | 0.01 | 0.03 | +4.54% | +6.14% |
| Timor-Leste | 0.44 | 0.24 | 0.67 | 0.02 | 0.01 | 0.02 | 0.52 | 0.31 | 0.76 | 0.02 | 0.01 | 0.03 | +17.94% | +20.65% |
| Turkey | 0.43 | 0.27 | 0.65 | 0.02 | 0.01 | 0.02 | 0.58 | 0.38 | 0.82 | 0.02 | 0.02 | 0.03 | +32.87% | +43.44% |
| Turkmenistan | 1.21 | 0.84 | 1.73 | 0.05 | 0.03 | 0.07 | 1.49 | 1.08 | 2.11 | 0.06 | 0.04 | 0.08 | +22.66% | +17.96% |
| United Arab Emirates | 0.61 | 0.38 | 0.91 | 0.02 | 0.01 | 0.03 | 0.92 | 0.62 | 1.34 | 0.04 | 0.02 | 0.05 | +50.51% | +62.51% |
| Uzbekistan | 1.14 | 0.76 | 1.66 | 0.05 | 0.03 | 0.07 | 1.32 | 0.94 | 1.87 | 0.05 | 0.04 | 0.06 | +16.37% | +4.54% |
| Viet Nam | 0.46 | 0.28 | 0.68 | 0.02 | 0.01 | 0.03 | 0.53 | 0.32 | 0.78 | 0.02 | 0.01 | 0.03 | +14.55% | +14.75% |
| Yemen | 0.39 | 0.21 | 0.61 | 0.01 | 0.01 | 0.02 | 0.55 | 0.33 | 0.82 | 0.02 | 0.01 | 0.03 | +41.92% | +59.51% |
| ***Cryptosporidium*** | |  |  |  |  |  |  |  |  |  |  |  |  |  |
| Asia | 250.74 | 156.70 | 383.57 | 5.39 | 3.38 | 8.26 | 34.13 | 21.64 | 53.61 | 0.90 | 0.55 | 1.58 | -86.39% | -83.24% |
| Afghanistan | 373.43 | 196.72 | 648.19 | 4.29 | 2.36 | 7.34 | 69.78 | 37.70 | 124.67 | 0.80 | 0.44 | 1.41 | -81.31% | -81.31% |
| Armenia | 62.61 | 37.20 | 100.81 | 0.71 | 0.42 | 1.13 | 1.52 | 0.91 | 2.34 | 0.02 | 0.01 | 0.03 | -97.58% | -97.56% |
| Azerbaijan | 98.56 | 55.16 | 164.55 | 1.11 | 0.63 | 1.85 | 15.09 | 7.63 | 27.19 | 0.17 | 0.09 | 0.31 | -84.69% | -84.43% |
| Bahrain | 16.06 | 9.84 | 25.09 | 0.31 | 0.18 | 0.49 | 3.98 | 2.42 | 5.90 | 0.12 | 0.06 | 0.21 | -75.25% | -59.96% |
| Bangladesh | 251.93 | 154.68 | 388.65 | 6.80 | 3.84 | 10.66 | 31.99 | 17.52 | 64.17 | 1.50 | 0.63 | 3.74 | -87.30% | -77.93% |
| Bhutan | 582.96 | 225.10 | 1,076.03 | 14.88 | 6.37 | 26.30 | 54.56 | 26.32 | 99.27 | 2.07 | 0.88 | 4.51 | -90.64% | -86.07% |
| Brunei Darussalam | 0.21 | 0.12 | 0.33 | 0.01 | 0.00 | 0.01 | 0.18 | 0.11 | 0.29 | 0.01 | 0.00 | 0.01 | -12.70% | -12.75% |
| Cambodia | 141.54 | 82.38 | 226.24 | 3.47 | 1.87 | 6.06 | 11.90 | 6.74 | 19.27 | 0.44 | 0.21 | 0.83 | -91.59% | -87.34% |
| China | 78.28 | 42.21 | 134.07 | 1.02 | 0.56 | 1.67 | 1.67 | 1.03 | 2.80 | 0.03 | 0.02 | 0.05 | -97.86% | -97.14% |
| Cyprus | 3.92 | 2.11 | 6.36 | 0.13 | 0.07 | 0.27 | 1.66 | 1.04 | 2.61 | 0.07 | 0.03 | 0.13 | -57.53% | -46.43% |
| Democratic People's Republic of Korea | 5.58 | 3.10 | 9.50 | 0.07 | 0.04 | 0.13 | 3.27 | 1.95 | 5.05 | 0.03 | 0.01 | 0.05 | -41.39% | -61.34% |
| Georgia | 27.18 | 16.10 | 42.64 | 0.31 | 0.19 | 0.48 | 0.81 | 0.51 | 1.23 | 0.01 | 0.01 | 0.01 | -97.03% | -97.30% |
| India | 576.07 | 368.05 | 870.62 | 16.88 | 10.17 | 26.55 | 80.98 | 48.17 | 132.63 | 3.30 | 1.83 | 6.03 | -85.94% | -80.48% |
| Indonesia | 201.02 | 115.02 | 310.36 | 5.02 | 2.43 | 8.71 | 21.90 | 12.64 | 33.29 | 0.85 | 0.35 | 1.61 | -89.11% | -83.13% |
| Iran (Islamic Republic of) | 53.82 | 27.85 | 96.06 | 0.73 | 0.40 | 1.22 | 2.99 | 1.93 | 4.67 | 0.08 | 0.04 | 0.14 | -94.44% | -89.46% |
| Iraq | 73.51 | 40.75 | 123.85 | 0.97 | 0.57 | 1.54 | 13.77 | 7.87 | 22.53 | 0.22 | 0.13 | 0.36 | -81.26% | -77.11% |
| Israel | 2.33 | 1.53 | 3.41 | 0.05 | 0.03 | 0.06 | 2.49 | 1.67 | 3.41 | 0.10 | 0.06 | 0.15 | +6.80% | +112.28% |
| Japan | 0.26 | 0.17 | 0.39 | 0.01 | 0.00 | 0.01 | 0.25 | 0.16 | 0.37 | 0.00 | 0.00 | 0.01 | -5.24% | -18.40% |
| Jordan | 16.15 | 9.16 | 26.28 | 0.23 | 0.14 | 0.36 | 4.29 | 2.55 | 6.94 | 0.08 | 0.04 | 0.13 | -73.43% | -67.68% |
| Kazakhstan | 52.55 | 31.63 | 82.87 | 0.60 | 0.36 | 0.94 | 1.09 | 0.66 | 1.72 | 0.01 | 0.01 | 0.02 | -97.93% | -97.83% |
| Kuwait | 4.94 | 3.15 | 7.64 | 0.06 | 0.04 | 0.09 | 1.18 | 0.75 | 1.68 | 0.02 | 0.01 | 0.02 | -76.05% | -73.47% |
| Kyrgyzstan | 69.47 | 42.44 | 108.99 | 0.80 | 0.49 | 1.24 | 4.41 | 2.61 | 7.05 | 0.05 | 0.03 | 0.08 | -93.66% | -93.70% |
| Lao People's Democratic Republic | 344.86 | 199.54 | 568.61 | 6.24 | 3.54 | 10.96 | 26.80 | 15.04 | 44.13 | 0.63 | 0.34 | 1.09 | -92.23% | -89.99% |
| Lebanon | 29.30 | 15.92 | 50.20 | 0.50 | 0.29 | 0.80 | 9.33 | 5.52 | 14.79 | 0.20 | 0.12 | 0.32 | -68.15% | -60.08% |
| Malaysia | 6.80 | 4.08 | 11.18 | 0.20 | 0.11 | 0.41 | 2.83 | 1.70 | 4.31 | 0.11 | 0.05 | 0.20 | -58.33% | -45.66% |
| Maldives | 94.81 | 53.85 | 148.69 | 1.75 | 1.04 | 2.85 | 4.55 | 2.79 | 7.19 | 0.11 | 0.06 | 0.18 | -95.20% | -93.81% |
| Mongolia | 34.77 | 17.39 | 67.80 | 0.39 | 0.19 | 0.75 | 3.58 | 1.20 | 8.93 | 0.04 | 0.01 | 0.10 | -89.69% | -89.62% |
| Myanmar | 119.36 | 56.46 | 220.68 | 2.19 | 0.99 | 4.06 | 9.42 | 5.57 | 14.61 | 0.23 | 0.12 | 0.39 | -92.10% | -89.34% |
| Nepal | 273.36 | 169.35 | 411.27 | 6.36 | 3.79 | 10.15 | 21.08 | 12.35 | 34.43 | 0.87 | 0.44 | 1.68 | -92.29% | -86.36% |
| Oman | 26.15 | 12.83 | 51.73 | 0.44 | 0.23 | 0.77 | 5.89 | 3.30 | 9.66 | 0.13 | 0.07 | 0.22 | -77.46% | -70.92% |
| Pakistan | 550.06 | 331.14 | 880.30 | 11.94 | 6.72 | 20.64 | 82.59 | 49.97 | 138.11 | 2.55 | 1.37 | 4.87 | -84.99% | -78.66% |
| Palestine | 39.17 | 21.42 | 67.99 | 0.58 | 0.34 | 0.92 | 3.43 | 2.05 | 5.44 | 0.06 | 0.04 | 0.11 | -91.23% | -88.91% |
| Philippines | 58.95 | 34.71 | 94.65 | 1.07 | 0.61 | 1.92 | 9.18 | 5.61 | 14.18 | 0.22 | 0.13 | 0.41 | -84.43% | -79.68% |
| Qatar | 17.29 | 10.61 | 28.33 | 0.35 | 0.21 | 0.56 | 6.10 | 3.84 | 8.87 | 0.16 | 0.08 | 0.29 | -64.70% | -53.14% |
| Republic of Korea | 0.51 | 0.29 | 0.79 | 0.02 | 0.01 | 0.04 | 0.19 | 0.11 | 0.30 | 0.01 | 0.00 | 0.02 | -63.70% | -57.86% |
| Saudi Arabia | 132.51 | 74.80 | 222.79 | 2.35 | 1.40 | 3.74 | 14.39 | 8.87 | 22.45 | 0.53 | 0.25 | 0.97 | -89.14% | -77.55% |
| Singapore | 0.26 | 0.18 | 0.38 | 0.01 | 0.01 | 0.01 | 0.09 | 0.06 | 0.12 | 0.00 | 0.00 | 0.01 | -66.77% | -52.57% |
| Sri Lanka | 55.68 | 35.06 | 87.51 | 1.97 | 1.03 | 3.47 | 5.72 | 3.35 | 9.22 | 0.22 | 0.10 | 0.41 | -89.72% | -88.81% |
| Syrian Arab Republic | 36.56 | 20.14 | 64.35 | 0.50 | 0.29 | 0.84 | 3.93 | 2.32 | 6.37 | 0.09 | 0.05 | 0.15 | -89.26% | -82.43% |
| Taiwan (Province of China) | 5.54 | 3.74 | 8.05 | 0.11 | 0.08 | 0.16 | 2.56 | 1.56 | 4.07 | 0.02 | 0.01 | 0.03 | -53.79% | -80.98% |
| Tajikistan | 279.49 | 161.92 | 459.37 | 3.18 | 1.85 | 5.21 | 79.77 | 43.88 | 135.26 | 0.91 | 0.51 | 1.53 | -71.46% | -71.40% |
| Thailand | 25.00 | 9.45 | 60.96 | 0.93 | 0.25 | 2.78 | 4.24 | 2.55 | 6.46 | 0.15 | 0.07 | 0.28 | -83.03% | -83.76% |
| Timor-Leste | 177.20 | 89.35 | 295.44 | 3.35 | 1.70 | 6.54 | 14.91 | 8.34 | 24.02 | 0.38 | 0.20 | 0.68 | -91.58% | -88.62% |
| Turkey | 77.27 | 40.95 | 129.47 | 0.95 | 0.53 | 1.52 | 5.94 | 3.51 | 9.50 | 0.10 | 0.06 | 0.17 | -92.32% | -89.07% |
| Turkmenistan | 152.32 | 88.55 | 246.31 | 1.72 | 1.01 | 2.77 | 4.92 | 2.84 | 8.10 | 0.06 | 0.03 | 0.09 | -96.77% | -96.72% |
| United Arab Emirates | 12.85 | 7.56 | 20.63 | 0.26 | 0.15 | 0.40 | 4.77 | 2.96 | 7.10 | 0.13 | 0.07 | 0.22 | -62.87% | -48.80% |
| Uzbekistan | 49.24 | 29.02 | 80.79 | 0.56 | 0.33 | 0.91 | 0.94 | 0.59 | 1.51 | 0.01 | 0.01 | 0.02 | -98.09% | -97.94% |
| Viet Nam | 6.42 | 2.95 | 11.26 | 0.18 | 0.07 | 0.40 | 0.59 | 0.36 | 0.90 | 0.02 | 0.01 | 0.04 | -90.77% | -89.47% |
| Yemen | 493.42 | 261.03 | 833.84 | 6.65 | 3.65 | 10.70 | 32.33 | 10.72 | 66.87 | 0.56 | 0.21 | 1.09 | -93.45% | -91.54% |
| ***Entamoeba*** |  |  |  |  |  |  |  |  |  |  |  |  |  |  |
| Asia | 120.59 | 61.12 | 221.19 | 1.99 | 0.96 | 3.60 | 17.61 | 9.08 | 31.79 | 0.32 | 0.15 | 0.59 | -85.39% | -84.10% |
| Afghanistan | 108.24 | 43.64 | 210.34 | 1.24 | 0.51 | 2.39 | 20.10 | 7.95 | 43.10 | 0.23 | 0.09 | 0.49 | -81.43% | -81.57% |
| Armenia | 38.05 | 17.33 | 75.09 | 0.43 | 0.19 | 0.84 | 1.08 | 0.51 | 2.04 | 0.01 | 0.00 | 0.02 | -97.16% | -97.45% |
| Azerbaijan | 62.24 | 26.11 | 126.06 | 0.70 | 0.29 | 1.42 | 9.92 | 4.18 | 21.73 | 0.11 | 0.05 | 0.25 | -84.06% | -84.05% |
| Bahrain | 4.70 | 2.25 | 9.44 | 0.07 | 0.03 | 0.13 | 1.32 | 0.68 | 2.29 | 0.02 | 0.01 | 0.04 | -71.97% | -68.19% |
| Bangladesh | 207.22 | 100.46 | 390.98 | 4.02 | 1.88 | 7.73 | 30.17 | 13.55 | 60.35 | 0.74 | 0.29 | 1.74 | -85.44% | -81.58% |
| Bhutan | 297.99 | 101.35 | 630.61 | 5.50 | 2.01 | 11.81 | 28.24 | 11.61 | 65.02 | 0.65 | 0.23 | 1.57 | -90.52% | -88.26% |
| Brunei Darussalam | 0.78 | 0.39 | 1.35 | 0.02 | 0.01 | 0.03 | 0.63 | 0.32 | 1.10 | 0.01 | 0.01 | 0.03 | -18.90% | -17.31% |
| Cambodia | 139.91 | 67.91 | 258.78 | 2.42 | 1.13 | 4.51 | 11.97 | 5.93 | 21.36 | 0.25 | 0.11 | 0.50 | -91.44% | -89.53% |
| China | 54.87 | 25.50 | 102.62 | 0.67 | 0.30 | 1.24 | 1.57 | 0.84 | 2.82 | 0.02 | 0.01 | 0.04 | -97.14% | -97.05% |
| Cyprus | 0.81 | 0.39 | 1.44 | 0.02 | 0.01 | 0.04 | 0.42 | 0.18 | 0.80 | 0.01 | 0.00 | 0.02 | -48.27% | -50.76% |
| Democratic People's Republic of Korea | 4.64 | 2.19 | 8.62 | 0.05 | 0.02 | 0.11 | 4.59 | 1.63 | 10.23 | 0.02 | 0.01 | 0.04 | -1.18% | -60.33% |
| Georgia | 17.98 | 8.09 | 33.84 | 0.20 | 0.09 | 0.38 | 0.70 | 0.32 | 1.27 | 0.01 | 0.00 | 0.01 | -96.10% | -97.11% |
| India | 195.51 | 95.76 | 375.31 | 4.13 | 1.93 | 7.45 | 28.58 | 14.19 | 54.58 | 0.72 | 0.30 | 1.49 | -85.38% | -82.51% |
| Indonesia | 246.26 | 122.24 | 452.86 | 4.56 | 2.07 | 8.58 | 26.47 | 13.21 | 47.25 | 0.63 | 0.26 | 1.26 | -89.25% | -86.24% |
| Iran (Islamic Republic of) | 16.74 | 7.25 | 34.31 | 0.20 | 0.08 | 0.44 | 1.30 | 0.62 | 2.33 | 0.02 | 0.01 | 0.03 | -92.22% | -91.64% |
| Iraq | 21.46 | 9.83 | 43.35 | 0.26 | 0.12 | 0.52 | 4.28 | 2.11 | 8.07 | 0.05 | 0.02 | 0.10 | -80.03% | -79.31% |
| Israel | 0.36 | 0.17 | 0.66 | 0.00 | 0.00 | 0.01 | 0.35 | 0.18 | 0.62 | 0.01 | 0.00 | 0.02 | -3.18% | +97.01% |
| Japan | 1.22 | 0.51 | 2.55 | 0.01 | 0.01 | 0.02 | 1.40 | 0.49 | 3.11 | 0.01 | 0.00 | 0.02 | 14.28% | -9.71% |
| Jordan | 5.21 | 2.41 | 10.20 | 0.06 | 0.03 | 0.12 | 1.52 | 0.73 | 2.73 | 0.02 | 0.01 | 0.03 | -70.78% | -71.62% |
| Kazakhstan | 34.33 | 14.87 | 66.84 | 0.39 | 0.17 | 0.76 | 0.92 | 0.44 | 1.64 | 0.01 | 0.00 | 0.02 | -97.33% | -97.66% |
| Kuwait | 2.36 | 1.23 | 4.27 | 0.02 | 0.01 | 0.05 | 0.77 | 0.34 | 1.54 | 0.01 | 0.00 | 0.01 | -67.24% | -78.35% |
| Kyrgyzstan | 45.75 | 20.38 | 89.75 | 0.53 | 0.23 | 1.03 | 3.06 | 1.41 | 6.04 | 0.03 | 0.01 | 0.07 | -93.30% | -93.55% |
| Lao People's Democratic Republic | 312.11 | 143.95 | 586.00 | 4.68 | 2.25 | 9.11 | 24.92 | 11.48 | 50.61 | 0.42 | 0.18 | 0.85 | -92.02% | -91.01% |
| Lebanon | 8.50 | 3.61 | 17.08 | 0.11 | 0.05 | 0.23 | 3.01 | 1.44 | 5.58 | 0.04 | 0.02 | 0.07 | -64.61% | -65.84% |
| Malaysia | 11.14 | 5.77 | 20.07 | 0.22 | 0.10 | 0.45 | 5.25 | 2.46 | 9.35 | 0.10 | 0.04 | 0.20 | -52.92% | -56.89% |
| Maldives | 143.83 | 70.12 | 259.32 | 2.18 | 1.05 | 3.95 | 7.38 | 3.83 | 13.07 | 0.11 | 0.05 | 0.21 | -94.87% | -95.01% |
| Mongolia | 22.30 | 9.22 | 49.06 | 0.25 | 0.10 | 0.54 | 2.23 | 0.66 | 5.42 | 0.02 | 0.01 | 0.06 | -89.99% | -90.00% |
| Myanmar | 225.73 | 96.42 | 478.91 | 3.32 | 1.38 | 6.77 | 18.76 | 9.03 | 35.68 | 0.32 | 0.15 | 0.60 | -91.69% | -90.44% |
| Nepal | 182.94 | 87.46 | 341.85 | 3.12 | 1.45 | 5.99 | 15.23 | 7.49 | 28.50 | 0.35 | 0.15 | 0.73 | -91.67% | -88.80% |
| Oman | 7.98 | 3.42 | 16.68 | 0.11 | 0.05 | 0.22 | 1.88 | 0.94 | 3.45 | 0.03 | 0.01 | 0.05 | -76.38% | -76.20% |
| Pakistan | 255.45 | 119.20 | 469.21 | 4.16 | 1.96 | 7.85 | 44.29 | 20.36 | 85.26 | 0.83 | 0.35 | 1.76 | -82.66% | -80.02% |
| Palestine | 11.39 | 4.88 | 23.43 | 0.14 | 0.06 | 0.29 | 1.15 | 0.56 | 2.16 | 0.01 | 0.01 | 0.03 | -89.89% | -90.21% |
| Philippines | 93.40 | 47.60 | 177.87 | 1.34 | 0.65 | 2.45 | 15.18 | 7.83 | 27.67 | 0.25 | 0.11 | 0.47 | -83.74% | -81.25% |
| Qatar | 1.08 | 0.52 | 2.02 | 0.02 | 0.01 | 0.03 | 0.42 | 0.20 | 0.78 | 0.01 | 0.00 | 0.01 | -60.82% | -62.01% |
| Republic of Korea | 1.66 | 0.82 | 3.00 | 0.04 | 0.02 | 0.09 | 0.64 | 0.30 | 1.14 | 0.02 | 0.01 | 0.04 | -61.22% | -59.10% |
| Saudi Arabia | 16.59 | 7.21 | 32.91 | 0.23 | 0.10 | 0.45 | 2.24 | 1.08 | 3.96 | 0.04 | 0.02 | 0.08 | -86.49% | -82.49% |
| Singapore | 0.91 | 0.45 | 1.64 | 0.02 | 0.01 | 0.04 | 0.29 | 0.15 | 0.51 | 0.01 | 0.00 | 0.02 | -68.01% | -60.51% |
| Sri Lanka | 36.21 | 18.14 | 63.77 | 0.79 | 0.36 | 1.54 | 4.21 | 1.92 | 7.62 | 0.08 | 0.03 | 0.17 | -88.36% | -89.84% |
| Syrian Arab Republic | 10.75 | 4.82 | 21.49 | 0.13 | 0.06 | 0.26 | 1.55 | 0.70 | 2.75 | 0.02 | 0.01 | 0.04 | -85.59% | -85.88% |
| Taiwan (Province of China) | 4.84 | 2.51 | 8.15 | 0.07 | 0.04 | 0.13 | 2.79 | 0.91 | 6.35 | 0.01 | 0.01 | 0.02 | -42.29% | -84.12% |
| Tajikistan | 130.43 | 57.38 | 258.82 | 1.48 | 0.65 | 2.93 | 37.05 | 15.19 | 71.60 | 0.42 | 0.17 | 0.82 | -71.59% | -71.44% |
| Thailand | 28.55 | 9.32 | 68.61 | 0.63 | 0.17 | 1.70 | 5.61 | 2.93 | 10.07 | 0.11 | 0.05 | 0.22 | -80.37% | -81.82% |
| Timor-Leste | 264.88 | 119.92 | 534.12 | 3.95 | 1.76 | 7.84 | 23.69 | 11.68 | 46.07 | 0.41 | 0.18 | 0.83 | -91.05% | -89.62% |
| Turkey | 22.00 | 9.22 | 44.26 | 0.26 | 0.11 | 0.51 | 1.97 | 0.94 | 3.53 | 0.02 | 0.01 | 0.05 | -91.03% | -91.01% |
| Turkmenistan | 99.70 | 42.85 | 192.63 | 1.13 | 0.48 | 2.19 | 3.54 | 1.65 | 6.84 | 0.04 | 0.02 | 0.08 | -96.45% | -96.43% |
| United Arab Emirates | 4.02 | 1.99 | 7.55 | 0.06 | 0.03 | 0.11 | 1.61 | 0.81 | 2.76 | 0.02 | 0.01 | 0.05 | -59.96% | -57.80% |
| Uzbekistan | 45.04 | 19.85 | 86.15 | 0.51 | 0.22 | 0.98 | 1.04 | 0.50 | 1.91 | 0.01 | 0.01 | 0.02 | -97.70% | -97.65% |
| Viet Nam | 31.94 | 14.55 | 60.11 | 0.61 | 0.25 | 1.24 | 4.14 | 1.76 | 8.00 | 0.06 | 0.02 | 0.12 | -87.03% | -90.67% |
| Yemen | 141.14 | 60.55 | 298.20 | 1.75 | 0.75 | 3.59 | 9.75 | 3.48 | 23.11 | 0.13 | 0.05 | 0.32 | -93.09% | -92.37% |
| ***Enteropathogenic E coli*** | |  |  |  |  |  |  |  |  |  |  |  |  |  |
| Asia | 192.44 | 111.75 | 302.89 | 2.18 | 1.28 | 3.42 | 25.79 | 13.36 | 44.04 | 0.29 | 0.14 | 0.49 | -86.60% | -86.92% |
| Afghanistan | 124.07 | 61.09 | 209.12 | 1.38 | 0.68 | 2.33 | 23.67 | 11.18 | 44.35 | 0.26 | 0.12 | 0.49 | -80.92% | -81.07% |
| Armenia | 23.84 | 13.44 | 38.95 | 0.26 | 0.15 | 0.43 | 0.53 | 0.32 | 0.91 | 0.01 | 0.00 | 0.01 | -97.77% | -98.04% |
| Azerbaijan | 40.37 | 21.88 | 69.31 | 0.45 | 0.24 | 0.77 | 6.27 | 3.16 | 12.08 | 0.07 | 0.03 | 0.13 | -84.46% | -84.57% |
| Bahrain | 4.81 | 2.68 | 7.89 | 0.05 | 0.03 | 0.08 | 0.93 | 0.54 | 1.48 | 0.01 | 0.00 | 0.01 | -80.61% | -83.11% |
| Bangladesh | 161.20 | 88.18 | 273.01 | 1.84 | 0.99 | 3.09 | 15.22 | 8.09 | 26.67 | 0.18 | 0.09 | 0.31 | -90.56% | -90.34% |
| Bhutan | 497.38 | 186.47 | 930.43 | 5.68 | 2.11 | 10.56 | 36.36 | 15.66 | 75.69 | 0.42 | 0.18 | 0.86 | -92.69% | -92.64% |
| Brunei Darussalam | 0.60 | 0.36 | 0.96 | 0.01 | 0.00 | 0.01 | 0.53 | 0.30 | 0.93 | 0.01 | 0.00 | 0.01 | -12.19% | -9.34% |
| Cambodia | 43.44 | 24.23 | 74.26 | 0.49 | 0.27 | 0.83 | 2.87 | 1.56 | 5.15 | 0.03 | 0.02 | 0.06 | -93.40% | -93.68% |
| China | 22.24 | 11.80 | 37.86 | 0.24 | 0.13 | 0.42 | 0.49 | 0.27 | 0.87 | 0.00 | 0.00 | 0.01 | -97.81% | -98.01% |
| Cyprus | 1.30 | 0.65 | 2.17 | 0.01 | 0.01 | 0.02 | 0.52 | 0.31 | 0.86 | 0.00 | 0.00 | 0.00 | -59.74% | -77.57% |
| Democratic People's Republic of Korea | 1.69 | 0.91 | 2.97 | 0.02 | 0.01 | 0.03 | 0.87 | 0.48 | 1.40 | 0.00 | 0.00 | 0.01 | -48.69% | -74.40% |
| Georgia | 11.89 | 6.73 | 19.40 | 0.13 | 0.07 | 0.21 | 0.33 | 0.20 | 0.53 | 0.00 | 0.00 | 0.00 | -97.25% | -98.09% |
| India | 419.56 | 242.02 | 653.99 | 4.84 | 2.85 | 7.46 | 47.57 | 24.67 | 79.76 | 0.54 | 0.27 | 0.91 | -88.66% | -88.80% |
| Indonesia | 136.32 | 74.94 | 219.14 | 1.54 | 0.86 | 2.50 | 11.48 | 6.54 | 20.07 | 0.13 | 0.07 | 0.22 | -91.58% | -91.80% |
| Iran (Islamic Republic of) | 36.08 | 18.53 | 68.98 | 0.38 | 0.18 | 0.74 | 1.57 | 0.93 | 2.52 | 0.01 | 0.01 | 0.02 | -95.65% | -96.66% |
| Iraq | 24.13 | 12.54 | 42.90 | 0.26 | 0.13 | 0.47 | 4.31 | 2.31 | 7.73 | 0.04 | 0.02 | 0.08 | -82.13% | -82.75% |
| Israel | 0.87 | 0.54 | 1.34 | 0.01 | 0.00 | 0.01 | 0.60 | 0.37 | 0.93 | 0.00 | 0.00 | 0.01 | -31.01% | -25.67% |
| Japan | 1.08 | 0.66 | 1.72 | 0.01 | 0.00 | 0.01 | 1.08 | 0.61 | 1.83 | 0.00 | 0.00 | 0.01 | -0.59% | -31.91% |
| Jordan | 4.22 | 2.17 | 7.24 | 0.04 | 0.02 | 0.08 | 1.05 | 0.57 | 1.87 | 0.01 | 0.00 | 0.02 | -75.21% | -76.82% |
| Kazakhstan | 22.60 | 12.84 | 36.77 | 0.25 | 0.14 | 0.41 | 0.42 | 0.25 | 0.69 | 0.00 | 0.00 | 0.01 | -98.16% | -98.47% |
| Kuwait | 2.43 | 1.50 | 3.85 | 0.02 | 0.01 | 0.03 | 0.57 | 0.34 | 0.87 | 0.00 | 0.00 | 0.01 | -76.64% | -83.26% |
| Kyrgyzstan | 30.13 | 17.15 | 49.01 | 0.33 | 0.19 | 0.54 | 1.80 | 1.02 | 3.00 | 0.02 | 0.01 | 0.03 | -94.03% | -94.22% |
| Lao People's Democratic Republic | 141.99 | 75.49 | 238.64 | 1.60 | 0.85 | 2.68 | 9.87 | 4.91 | 18.06 | 0.11 | 0.05 | 0.20 | -93.05% | -93.25% |
| Lebanon | 9.08 | 4.60 | 16.11 | 0.10 | 0.05 | 0.17 | 2.72 | 1.47 | 4.76 | 0.03 | 0.01 | 0.05 | -70.09% | -72.76% |
| Malaysia | 9.49 | 5.18 | 15.49 | 0.10 | 0.05 | 0.17 | 2.98 | 1.83 | 4.89 | 0.02 | 0.01 | 0.04 | -68.55% | -76.83% |
| Maldives | 85.37 | 47.46 | 139.88 | 0.96 | 0.52 | 1.56 | 3.82 | 2.21 | 6.55 | 0.04 | 0.02 | 0.07 | -95.53% | -96.12% |
| Mongolia | 14.07 | 7.34 | 26.03 | 0.15 | 0.08 | 0.29 | 1.27 | 0.45 | 3.16 | 0.01 | 0.00 | 0.03 | -90.99% | -91.01% |
| Myanmar | 246.77 | 106.80 | 474.42 | 2.77 | 1.18 | 5.32 | 19.49 | 10.52 | 34.27 | 0.21 | 0.11 | 0.37 | -92.10% | -92.41% |
| Nepal | 278.79 | 158.30 | 448.73 | 3.15 | 1.80 | 5.05 | 16.14 | 8.25 | 28.90 | 0.19 | 0.09 | 0.34 | -94.21% | -94.13% |
| Oman | 12.70 | 6.06 | 24.27 | 0.13 | 0.06 | 0.27 | 2.60 | 1.48 | 4.57 | 0.03 | 0.01 | 0.05 | -79.52% | -80.57% |
| Pakistan | 533.32 | 302.57 | 824.84 | 6.00 | 3.42 | 9.28 | 70.18 | 33.90 | 129.68 | 0.79 | 0.37 | 1.44 | -86.84% | -86.90% |
| Palestine | 12.57 | 6.22 | 23.25 | 0.14 | 0.07 | 0.25 | 1.03 | 0.55 | 1.82 | 0.01 | 0.01 | 0.02 | -91.80% | -92.64% |
| Philippines | 51.95 | 29.76 | 84.96 | 0.57 | 0.33 | 0.94 | 7.53 | 4.51 | 13.14 | 0.08 | 0.05 | 0.14 | -85.50% | -85.93% |
| Qatar | 2.20 | 1.22 | 3.75 | 0.02 | 0.01 | 0.04 | 0.66 | 0.39 | 1.13 | 0.01 | 0.00 | 0.01 | -69.99% | -76.53% |
| Republic of Korea | 1.32 | 0.76 | 2.15 | 0.01 | 0.01 | 0.02 | 0.42 | 0.25 | 0.71 | 0.00 | 0.00 | 0.01 | -68.04% | -71.70% |
| Saudi Arabia | 17.34 | 8.70 | 32.83 | 0.19 | 0.09 | 0.36 | 1.31 | 0.72 | 2.21 | 0.01 | 0.01 | 0.02 | -92.45% | -93.52% |
| Singapore | 0.72 | 0.44 | 1.16 | 0.01 | 0.00 | 0.01 | 0.20 | 0.12 | 0.33 | 0.00 | 0.00 | 0.00 | -72.85% | -73.83% |
| Sri Lanka | 14.90 | 8.66 | 24.19 | 0.17 | 0.10 | 0.27 | 1.32 | 0.78 | 2.18 | 0.01 | 0.01 | 0.02 | -91.12% | -93.11% |
| Syrian Arab Republic | 11.97 | 6.37 | 21.40 | 0.13 | 0.06 | 0.23 | 1.11 | 0.61 | 1.84 | 0.01 | 0.00 | 0.02 | -90.76% | -92.58% |
| Taiwan (Province of China) | 1.43 | 0.89 | 2.19 | 0.01 | 0.01 | 0.02 | 0.69 | 0.39 | 1.16 | 0.00 | 0.00 | 0.00 | -51.61% | -88.36% |
| Tajikistan | 83.87 | 46.55 | 140.10 | 0.93 | 0.51 | 1.55 | 24.08 | 12.25 | 43.23 | 0.27 | 0.14 | 0.48 | -71.29% | -71.26% |
| Thailand | 12.48 | 4.93 | 24.02 | 0.14 | 0.05 | 0.26 | 2.03 | 1.13 | 3.35 | 0.02 | 0.01 | 0.03 | -83.70% | -84.81% |
| Timor-Leste | 155.92 | 79.45 | 258.70 | 1.75 | 0.89 | 2.92 | 11.76 | 6.30 | 21.99 | 0.13 | 0.07 | 0.24 | -92.46% | -92.69% |
| Turkey | 25.83 | 13.48 | 46.67 | 0.28 | 0.14 | 0.51 | 1.82 | 1.01 | 3.08 | 0.02 | 0.01 | 0.03 | -92.96% | -93.68% |
| Turkmenistan | 64.01 | 36.54 | 102.45 | 0.71 | 0.40 | 1.13 | 1.98 | 1.07 | 3.49 | 0.02 | 0.01 | 0.04 | -96.91% | -97.00% |
| United Arab Emirates | 3.73 | 2.03 | 6.44 | 0.04 | 0.02 | 0.07 | 1.20 | 0.69 | 1.95 | 0.01 | 0.01 | 0.02 | -67.94% | -70.37% |
| Uzbekistan | 33.09 | 18.80 | 55.02 | 0.37 | 0.21 | 0.61 | 0.65 | 0.37 | 1.08 | 0.01 | 0.00 | 0.01 | -98.02% | -98.11% |
| Viet Nam | 5.52 | 2.62 | 9.89 | 0.06 | 0.03 | 0.11 | 0.41 | 0.23 | 0.67 | 0.00 | 0.00 | 0.00 | -92.63% | -96.33% |
| Yemen | 160.78 | 80.27 | 297.62 | 1.79 | 0.89 | 3.31 | 9.99 | 3.46 | 22.18 | 0.11 | 0.03 | 0.24 | -93.79% | -93.99% |
| ***Enterotoxigenic E coli*** | |  |  |  |  |  |  |  |  |  |  |  |  |  |
| Asia | 293.76 | 190.58 | 440.88 | 7.18 | 4.68 | 10.94 | 42.18 | 27.03 | 62.16 | 1.20 | 0.71 | 1.99 | -85.64% | -83.34% |
| Afghanistan | 278.26 | 135.81 | 490.99 | 3.19 | 1.60 | 5.58 | 51.68 | 26.51 | 95.40 | 0.59 | 0.31 | 1.07 | -81.43% | -81.55% |
| Armenia | 81.30 | 47.98 | 135.52 | 0.90 | 0.53 | 1.49 | 2.10 | 1.29 | 3.31 | 0.02 | 0.01 | 0.03 | -97.42% | -97.66% |
| Azerbaijan | 134.59 | 72.79 | 241.91 | 1.50 | 0.81 | 2.70 | 20.71 | 10.01 | 35.86 | 0.23 | 0.11 | 0.40 | -84.61% | -84.54% |
| Bahrain | 12.46 | 7.76 | 20.56 | 0.23 | 0.14 | 0.38 | 3.74 | 2.51 | 5.63 | 0.09 | 0.05 | 0.17 | -70.01% | -58.86% |
| Bangladesh | 388.80 | 250.88 | 598.20 | 12.10 | 7.02 | 19.33 | 59.35 | 32.08 | 111.78 | 2.79 | 1.14 | 6.83 | -84.73% | -76.92% |
| Bhutan | 759.48 | 323.37 | 1,413.33 | 20.10 | 9.08 | 37.22 | 72.68 | 36.40 | 143.72 | 2.81 | 1.12 | 6.34 | -90.43% | -86.00% |
| Brunei Darussalam | 0.61 | 0.40 | 0.93 | 0.02 | 0.01 | 0.04 | 0.52 | 0.33 | 0.83 | 0.02 | 0.01 | 0.03 | -15.00% | -13.15% |
| Cambodia | 235.44 | 143.28 | 388.23 | 6.09 | 3.36 | 10.32 | 20.75 | 12.57 | 33.83 | 0.78 | 0.36 | 1.36 | -91.19% | -87.11% |
| China | 13.04 | 6.35 | 25.74 | 0.17 | 0.09 | 0.31 | 0.31 | 0.19 | 0.54 | 0.00 | 0.00 | 0.01 | -97.59% | -97.10% |
| Cyprus | 1.74 | 1.06 | 2.80 | 0.07 | 0.03 | 0.16 | 1.02 | 0.66 | 1.64 | 0.04 | 0.02 | 0.08 | -41.52% | -40.77% |
| Democratic People's Republic of Korea | 1.19 | 0.66 | 2.06 | 0.01 | 0.01 | 0.02 | 0.75 | 0.47 | 1.16 | 0.01 | 0.00 | 0.01 | -37.10% | -60.83% |
| Georgia | 38.38 | 22.46 | 61.49 | 0.42 | 0.24 | 0.68 | 1.35 | 0.84 | 2.09 | 0.01 | 0.01 | 0.02 | -96.49% | -97.50% |
| India | 560.30 | 363.65 | 827.06 | 18.10 | 11.45 | 29.74 | 81.70 | 50.63 | 126.95 | 3.63 | 1.87 | 6.77 | -85.42% | -79.92% |
| Indonesia | 1,076.27 | 637.76 | 1,656.32 | 31.64 | 15.45 | 53.74 | 134.05 | 79.19 | 206.23 | 5.76 | 2.31 | 10.80 | -87.54% | -81.80% |
| Iran (Islamic Republic of) | 95.12 | 51.54 | 170.31 | 1.31 | 0.70 | 2.29 | 7.37 | 4.98 | 10.92 | 0.17 | 0.09 | 0.31 | -92.25% | -87.35% |
| Iraq | 55.00 | 30.32 | 94.93 | 0.71 | 0.40 | 1.15 | 10.85 | 6.34 | 18.90 | 0.17 | 0.10 | 0.27 | -80.26% | -76.60% |
| Israel | 0.75 | 0.52 | 1.07 | 0.02 | 0.01 | 0.02 | 0.92 | 0.64 | 1.30 | 0.04 | 0.02 | 0.06 | +22.50% | +147.89% |
| Japan | 1.05 | 0.70 | 1.58 | 0.02 | 0.01 | 0.02 | 1.11 | 0.71 | 1.70 | 0.01 | 0.01 | 0.02 | +5.95% | -15.41% |
| Jordan | 13.86 | 8.40 | 24.47 | 0.19 | 0.12 | 0.31 | 4.00 | 2.48 | 6.19 | 0.06 | 0.04 | 0.10 | -71.13% | -67.56% |
| Kazakhstan | 73.84 | 42.75 | 120.40 | 0.82 | 0.48 | 1.34 | 1.65 | 0.99 | 2.59 | 0.02 | 0.01 | 0.03 | -97.77% | -97.99% |
| Kuwait | 5.76 | 3.77 | 8.89 | 0.06 | 0.04 | 0.09 | 1.76 | 1.11 | 2.64 | 0.02 | 0.01 | 0.02 | -69.36% | -73.45% |
| Kyrgyzstan | 96.97 | 55.93 | 156.64 | 1.09 | 0.64 | 1.75 | 6.25 | 3.56 | 10.68 | 0.07 | 0.04 | 0.12 | -93.55% | -93.74% |
| Lao People's Democratic Republic | 641.14 | 368.89 | 1,076.73 | 12.72 | 7.18 | 23.07 | 49.87 | 28.34 | 86.01 | 1.31 | 0.75 | 2.40 | -92.22% | -89.69% |
| Lebanon | 21.66 | 12.11 | 36.27 | 0.36 | 0.22 | 0.60 | 7.83 | 4.68 | 12.93 | 0.15 | 0.09 | 0.25 | -63.86% | -59.13% |
| Malaysia | 19.27 | 11.99 | 30.21 | 0.62 | 0.34 | 1.22 | 8.83 | 5.58 | 13.55 | 0.35 | 0.14 | 0.63 | -54.17% | -43.64% |
| Maldives | 246.99 | 143.23 | 408.47 | 5.15 | 3.06 | 8.53 | 13.07 | 8.40 | 20.89 | 0.33 | 0.18 | 0.55 | -94.71% | -93.60% |
| Mongolia | 47.87 | 23.56 | 85.76 | 0.52 | 0.25 | 0.95 | 4.68 | 1.67 | 10.53 | 0.05 | 0.02 | 0.12 | -90.22% | -90.16% |
| Myanmar | 613.54 | 270.79 | 1,183.51 | 11.88 | 5.58 | 23.20 | 53.13 | 33.59 | 86.88 | 1.37 | 0.78 | 2.22 | -91.34% | -88.47% |
| Nepal | 346.84 | 216.90 | 535.96 | 8.42 | 5.30 | 13.63 | 29.75 | 17.85 | 49.80 | 1.23 | 0.60 | 2.58 | -91.42% | -85.35% |
| Oman | 20.38 | 10.32 | 35.59 | 0.33 | 0.18 | 0.56 | 5.13 | 3.20 | 8.21 | 0.10 | 0.05 | 0.16 | -74.82% | -70.57% |
| Pakistan | 688.76 | 423.44 | 1,082.92 | 15.71 | 8.92 | 27.64 | 113.25 | 67.36 | 194.35 | 3.51 | 1.89 | 6.83 | -83.56% | -77.66% |
| Palestine | 29.51 | 16.23 | 51.05 | 0.43 | 0.25 | 0.73 | 2.96 | 1.78 | 4.69 | 0.05 | 0.03 | 0.08 | -89.98% | -88.61% |
| Philippines | 146.56 | 87.86 | 228.70 | 2.94 | 1.73 | 5.11 | 23.88 | 14.92 | 38.24 | 0.62 | 0.37 | 1.14 | -83.71% | -78.86% |
| Qatar | 6.13 | 3.75 | 10.18 | 0.11 | 0.07 | 0.19 | 2.69 | 1.79 | 4.03 | 0.05 | 0.03 | 0.10 | -56.18% | -51.97% |
| Republic of Korea | 1.48 | 0.92 | 2.33 | 0.07 | 0.03 | 0.13 | 0.59 | 0.36 | 1.00 | 0.03 | 0.01 | 0.06 | -60.42% | -57.09% |
| Saudi Arabia | 43.58 | 23.77 | 76.49 | 0.76 | 0.44 | 1.29 | 5.68 | 3.65 | 8.57 | 0.18 | 0.09 | 0.32 | -86.98% | -76.81% |
| Singapore | 0.75 | 0.53 | 1.03 | 0.03 | 0.02 | 0.04 | 0.27 | 0.18 | 0.37 | 0.01 | 0.01 | 0.02 | -64.03% | -51.16% |
| Sri Lanka | 63.82 | 39.02 | 101.00 | 2.58 | 1.34 | 4.53 | 7.09 | 4.46 | 10.89 | 0.28 | 0.13 | 0.56 | -88.89% | -89.01% |
| Syrian Arab Republic | 28.67 | 17.04 | 52.62 | 0.37 | 0.22 | 0.64 | 3.89 | 2.38 | 6.09 | 0.07 | 0.04 | 0.12 | -86.43% | -81.51% |
| Taiwan (Province of China) | 1.23 | 0.82 | 1.82 | 0.02 | 0.01 | 0.03 | 0.76 | 0.49 | 1.16 | 0.00 | 0.00 | 0.01 | -38.03% | -80.88% |
| Tajikistan | 244.20 | 138.19 | 413.13 | 2.74 | 1.54 | 4.66 | 70.23 | 35.54 | 122.49 | 0.79 | 0.41 | 1.38 | -71.24% | -71.13% |
| Thailand | 34.17 | 14.14 | 74.96 | 1.36 | 0.40 | 3.77 | 6.40 | 4.18 | 9.80 | 0.24 | 0.12 | 0.48 | -81.28% | -82.22% |
| Timor-Leste | 458.28 | 232.90 | 742.60 | 9.34 | 4.85 | 17.04 | 41.41 | 23.49 | 69.88 | 1.16 | 0.61 | 2.19 | -90.96% | -87.63% |
| Turkey | 58.46 | 31.72 | 113.87 | 0.70 | 0.39 | 1.30 | 5.13 | 3.13 | 8.14 | 0.08 | 0.05 | 0.12 | -91.22% | -88.72% |
| Turkmenistan | 216.34 | 124.16 | 351.79 | 2.42 | 1.40 | 3.91 | 6.97 | 3.90 | 12.01 | 0.08 | 0.04 | 0.13 | -96.78% | -96.82% |
| United Arab Emirates | 10.74 | 6.66 | 17.56 | 0.20 | 0.11 | 0.32 | 4.44 | 2.95 | 6.86 | 0.10 | 0.05 | 0.17 | -58.64% | -48.46% |
| Uzbekistan | 108.33 | 63.49 | 175.88 | 1.21 | 0.71 | 1.95 | 2.19 | 1.29 | 3.46 | 0.02 | 0.01 | 0.04 | -97.98% | -97.96% |
| Viet Nam | 19.03 | 9.45 | 31.93 | 0.59 | 0.23 | 1.28 | 2.03 | 1.31 | 3.08 | 0.07 | 0.02 | 0.13 | -89.35% | -88.88% |
| Yemen | 362.71 | 188.77 | 625.91 | 4.91 | 2.67 | 8.64 | 24.92 | 9.40 | 49.75 | 0.42 | 0.16 | 0.81 | -93.13% | -91.39% |
| ***Non-typhoidal Salmonella*** | |  |  |  |  |  |  |  |  |  |  |  |  |  |
| Asia | 76.26 | 10.00 | 166.51 | 1.33 | 0.10 | 3.11 | 10.19 | 1.86 | 22.73 | 0.21 | 0.01 | 0.52 | -86.64% | -84.60% |
| Afghanistan | 148.31 | 24.83 | 333.45 | 1.71 | 0.27 | 3.85 | 26.82 | 5.61 | 60.16 | 0.31 | 0.06 | 0.68 | -81.92% | -82.04% |
| Armenia | 5.42 | 1.18 | 11.74 | 0.06 | 0.01 | 0.13 | 0.15 | 0.05 | 0.31 | 0.00 | 0.00 | 0.00 | -97.19% | -97.42% |
| Azerbaijan | 9.26 | 1.94 | 21.26 | 0.10 | 0.02 | 0.24 | 1.45 | 0.33 | 3.31 | 0.02 | 0.00 | 0.04 | -84.31% | -84.24% |
| Bahrain | 6.48 | 1.44 | 13.60 | 0.10 | 0.01 | 0.23 | 1.86 | 0.54 | 3.73 | 0.03 | 0.00 | 0.09 | -71.28% | -66.04% |
| Bangladesh | 63.52 | 7.13 | 151.75 | 1.46 | 0.07 | 3.69 | 8.93 | 0.87 | 28.20 | 0.28 | 0.01 | 1.02 | -85.95% | -80.77% |
| Bhutan | 119.66 | 10.68 | 316.74 | 2.54 | 0.11 | 7.58 | 11.14 | 1.15 | 30.93 | 0.31 | 0.01 | 1.08 | -90.69% | -87.80% |
| Brunei Darussalam | 1.20 | 0.26 | 2.66 | 0.03 | 0.00 | 0.08 | 1.00 | 0.22 | 2.18 | 0.02 | 0.00 | 0.06 | -17.21% | -16.36% |
| Cambodia | 98.76 | 13.94 | 219.52 | 1.69 | 0.15 | 4.04 | 7.91 | 1.40 | 17.90 | 0.17 | 0.01 | 0.46 | -92.00% | -89.80% |
| China | 37.05 | 5.58 | 80.31 | 0.45 | 0.05 | 1.00 | 0.95 | 0.26 | 1.97 | 0.01 | 0.00 | 0.03 | -97.45% | -97.30% |
| Cyprus | 5.38 | 1.47 | 10.95 | 0.13 | 0.01 | 0.37 | 2.65 | 0.81 | 5.09 | 0.06 | 0.00 | 0.19 | -50.69% | -50.02% |
| Democratic People's Republic of Korea | 2.64 | 0.70 | 6.11 | 0.03 | 0.00 | 0.08 | 1.91 | 0.79 | 3.45 | 0.01 | 0.00 | 0.03 | -27.56% | -63.73% |
| Georgia | 2.63 | 0.62 | 5.72 | 0.03 | 0.01 | 0.06 | 0.09 | 0.03 | 0.17 | 0.00 | 0.00 | 0.00 | -96.41% | -97.19% |
| India | 128.44 | 13.59 | 296.86 | 3.02 | 0.14 | 7.54 | 17.96 | 2.65 | 42.51 | 0.53 | 0.02 | 1.51 | -86.01% | -82.44% |
| Indonesia | 214.79 | 29.54 | 468.46 | 3.91 | 0.30 | 10.28 | 22.46 | 3.55 | 50.11 | 0.54 | 0.03 | 1.49 | -89.54% | -86.17% |
| Iran (Islamic Republic of) | 29.27 | 6.09 | 64.04 | 0.36 | 0.05 | 0.81 | 2.43 | 0.73 | 4.82 | 0.03 | 0.00 | 0.09 | -91.69% | -90.38% |
| Iraq | 29.34 | 5.95 | 61.41 | 0.36 | 0.05 | 0.78 | 5.93 | 1.40 | 12.72 | 0.08 | 0.01 | 0.19 | -79.80% | -78.63% |
| Israel | 1.34 | 0.49 | 2.37 | 0.02 | 0.00 | 0.04 | 1.36 | 0.37 | 2.57 | 0.04 | 0.00 | 0.09 | +1.36% | +99.50% |
| Japan | 1.62 | 0.65 | 2.84 | 0.02 | 0.00 | 0.04 | 1.76 | 0.69 | 3.11 | 0.02 | 0.00 | 0.04 | +8.01% | -10.35% |
| Jordan | 4.64 | 1.13 | 9.66 | 0.06 | 0.01 | 0.13 | 1.37 | 0.43 | 2.63 | 0.02 | 0.00 | 0.04 | -70.53% | -70.60% |
| Kazakhstan | 5.10 | 1.03 | 10.58 | 0.06 | 0.01 | 0.12 | 0.13 | 0.03 | 0.26 | 0.00 | 0.00 | 0.00 | -97.52% | -97.73% |
| Kuwait | 3.35 | 1.09 | 6.16 | 0.04 | 0.00 | 0.07 | 1.09 | 0.46 | 1.92 | 0.01 | 0.00 | 0.02 | -67.37% | -77.26% |
| Kyrgyzstan | 6.75 | 1.44 | 14.05 | 0.08 | 0.02 | 0.16 | 0.45 | 0.09 | 0.95 | 0.01 | 0.00 | 0.01 | -93.28% | -93.46% |
| Lao People's Democratic Republic | 247.97 | 35.75 | 542.38 | 3.65 | 0.40 | 8.78 | 18.68 | 3.43 | 43.42 | 0.31 | 0.03 | 0.80 | -92.46% | -91.40% |
| Lebanon | 11.56 | 2.54 | 23.91 | 0.16 | 0.02 | 0.37 | 4.16 | 1.25 | 8.28 | 0.06 | 0.01 | 0.14 | -64.00% | -64.32% |
| Malaysia | 12.03 | 2.14 | 26.71 | 0.24 | 0.02 | 0.68 | 5.07 | 1.36 | 10.03 | 0.11 | 0.00 | 0.30 | -57.84% | -56.80% |
| Maldives | 103.17 | 13.65 | 230.61 | 1.55 | 0.15 | 3.71 | 4.95 | 1.31 | 10.37 | 0.08 | 0.01 | 0.19 | -95.20% | -95.09% |
| Mongolia | 3.37 | 0.60 | 7.79 | 0.04 | 0.01 | 0.09 | 0.34 | 0.06 | 0.94 | 0.00 | 0.00 | 0.01 | -89.77% | -89.75% |
| Myanmar | 79.93 | 10.14 | 183.72 | 1.16 | 0.11 | 2.97 | 6.25 | 1.11 | 13.97 | 0.11 | 0.01 | 0.25 | -92.19% | -90.91% |
| Nepal | 42.86 | 5.32 | 96.16 | 0.84 | 0.06 | 2.18 | 3.71 | 0.42 | 9.60 | 0.11 | 0.00 | 0.36 | -91.35% | -87.30% |
| Oman | 6.40 | 1.28 | 15.00 | 0.09 | 0.01 | 0.23 | 1.55 | 0.43 | 3.04 | 0.02 | 0.00 | 0.06 | -75.77% | -75.00% |
| Pakistan | 117.54 | 16.64 | 256.65 | 2.20 | 0.19 | 6.25 | 19.51 | 2.56 | 47.63 | 0.45 | 0.02 | 1.32 | -83.40% | -79.55% |
| Palestine | 21.83 | 4.05 | 50.07 | 0.28 | 0.04 | 0.65 | 2.22 | 0.65 | 4.36 | 0.03 | 0.00 | 0.07 | -89.85% | -89.83% |
| Philippines | 60.63 | 8.22 | 129.69 | 0.87 | 0.07 | 2.09 | 9.41 | 1.57 | 20.73 | 0.16 | 0.01 | 0.38 | -84.48% | -81.88% |
| Qatar | 3.08 | 0.83 | 6.23 | 0.05 | 0.00 | 0.11 | 1.28 | 0.43 | 2.39 | 0.02 | 0.00 | 0.05 | -58.65% | -58.93% |
| Republic of Korea | 1.15 | 0.20 | 2.63 | 0.03 | 0.00 | 0.09 | 0.43 | 0.09 | 0.98 | 0.01 | 0.00 | 0.04 | -62.74% | -59.40% |
| Saudi Arabia | 37.92 | 6.79 | 83.09 | 0.55 | 0.06 | 1.27 | 5.25 | 1.20 | 11.53 | 0.11 | 0.00 | 0.31 | -86.16% | -80.96% |
| Singapore | 3.54 | 0.48 | 7.18 | 0.09 | 0.00 | 0.22 | 1.10 | 0.16 | 2.27 | 0.04 | 0.00 | 0.09 | -69.01% | -61.51% |
| Sri Lanka | 23.81 | 2.87 | 55.86 | 0.53 | 0.02 | 1.44 | 2.51 | 0.60 | 5.69 | 0.05 | 0.00 | 0.15 | -89.48% | -89.97% |
| Syrian Arab Republic | 14.90 | 3.37 | 31.79 | 0.18 | 0.03 | 0.40 | 2.20 | 0.56 | 4.81 | 0.03 | 0.00 | 0.08 | -85.20% | -84.51% |
| Taiwan (Province of China) | 1.96 | 0.50 | 3.85 | 0.03 | 0.00 | 0.06 | 1.01 | 0.44 | 1.70 | 0.00 | 0.00 | 0.01 | -48.56% | -84.12% |
| Tajikistan | 20.89 | 3.50 | 46.32 | 0.24 | 0.04 | 0.52 | 5.76 | 1.09 | 13.47 | 0.07 | 0.01 | 0.15 | -72.43% | -72.28% |
| Thailand | 25.64 | 3.29 | 74.87 | 0.58 | 0.03 | 2.27 | 4.62 | 0.87 | 10.21 | 0.10 | 0.00 | 0.28 | -81.97% | -82.78% |
| Timor-Leste | 190.52 | 24.70 | 422.09 | 2.83 | 0.27 | 6.97 | 16.17 | 2.82 | 37.04 | 0.28 | 0.02 | 0.71 | -91.51% | -90.02% |
| Turkey | 30.11 | 6.09 | 64.19 | 0.35 | 0.06 | 0.77 | 2.73 | 0.76 | 5.40 | 0.03 | 0.00 | 0.08 | -90.94% | -90.46% |
| Turkmenistan | 15.09 | 2.32 | 32.05 | 0.17 | 0.02 | 0.36 | 0.51 | 0.10 | 1.08 | 0.01 | 0.00 | 0.01 | -96.59% | -96.57% |
| United Arab Emirates | 5.66 | 1.31 | 12.26 | 0.09 | 0.01 | 0.22 | 2.28 | 0.63 | 4.58 | 0.04 | 0.00 | 0.10 | -59.74% | -56.11% |
| Uzbekistan | 7.37 | 1.41 | 15.54 | 0.08 | 0.01 | 0.18 | 0.16 | 0.04 | 0.34 | 0.00 | 0.00 | 0.00 | -97.84% | -97.78% |
| Viet Nam | 14.26 | 2.43 | 32.53 | 0.28 | 0.02 | 0.84 | 1.52 | 0.51 | 2.97 | 0.02 | 0.00 | 0.08 | -89.37% | -90.95% |
| Yemen | 191.74 | 29.50 | 427.46 | 2.44 | 0.31 | 5.48 | 13.24 | 2.26 | 32.72 | 0.19 | 0.01 | 0.51 | -93.10% | -92.14% |
| ***Norovirus*** |  |  |  |  |  |  |  |  |  |  |  |  |  |  |
| Asia | 346.95 | 103.65 | 586.47 | 9.17 | 1.93 | 16.52 | 59.43 | 20.37 | 102.08 | 1.69 | 0.30 | 3.25 | -82.87% | -81.57% |
| Afghanistan | 280.03 | 93.74 | 512.69 | 3.35 | 1.10 | 6.21 | 49.10 | 18.95 | 95.14 | 0.58 | 0.20 | 1.10 | -82.47% | -82.76% |
| Armenia | 73.42 | 33.24 | 132.78 | 0.82 | 0.32 | 1.48 | 3.18 | 1.61 | 5.18 | 0.03 | 0.01 | 0.05 | -95.67% | -96.62% |
| Azerbaijan | 112.41 | 45.07 | 220.28 | 1.27 | 0.48 | 2.46 | 19.31 | 7.78 | 37.28 | 0.22 | 0.07 | 0.42 | -82.82% | -82.90% |
| Bahrain | 15.10 | 6.17 | 25.13 | 0.31 | 0.08 | 0.56 | 6.60 | 2.85 | 10.79 | 0.15 | 0.02 | 0.29 | -56.30% | -53.01% |
| Bangladesh | 549.12 | 144.22 | 940.67 | 16.88 | 3.00 | 30.27 | 95.31 | 23.08 | 208.85 | 3.89 | 0.44 | 10.12 | -82.64% | -76.93% |
| Bhutan | 834.25 | 180.43 | 1,761.61 | 25.17 | 4.36 | 52.50 | 93.09 | 22.54 | 204.16 | 3.59 | 0.55 | 8.75 | -88.84% | -85.73% |
| Brunei Darussalam | 4.93 | 1.98 | 8.39 | 0.15 | 0.02 | 0.32 | 3.89 | 1.45 | 6.56 | 0.13 | 0.02 | 0.25 | -21.10% | -16.57% |
| Cambodia | 214.04 | 62.14 | 373.62 | 5.82 | 1.17 | 11.58 | 22.87 | 8.00 | 40.01 | 0.75 | 0.13 | 1.46 | -89.32% | -87.04% |
| China | 78.79 | 26.78 | 145.01 | 1.10 | 0.32 | 2.05 | 2.71 | 1.21 | 4.51 | 0.04 | 0.01 | 0.08 | -96.56% | -96.31% |
| Cyprus | 13.75 | 5.75 | 25.02 | 0.53 | 0.09 | 1.35 | 9.83 | 4.29 | 16.49 | 0.31 | 0.04 | 0.65 | -28.49% | -40.81% |
| Democratic People's Republic of Korea | 9.14 | 4.05 | 15.08 | 0.10 | 0.03 | 0.19 | 12.68 | 4.14 | 22.72 | 0.05 | 0.01 | 0.10 | +38.78% | -52.40% |
| Georgia | 35.28 | 16.48 | 62.09 | 0.39 | 0.15 | 0.68 | 2.63 | 1.13 | 4.44 | 0.02 | 0.00 | 0.03 | -92.54% | -95.60% |
| India | 794.73 | 213.51 | 1,373.31 | 27.33 | 4.71 | 50.36 | 140.39 | 42.17 | 254.78 | 5.54 | 0.84 | 11.29 | -82.33% | -79.75% |
| Indonesia | 654.62 | 186.19 | 1,198.27 | 19.06 | 3.51 | 37.92 | 85.23 | 24.54 | 152.36 | 3.21 | 0.58 | 6.73 | -86.98% | -83.17% |
| Iran (Islamic Republic of) | 46.16 | 18.74 | 85.49 | 0.65 | 0.18 | 1.26 | 6.62 | 2.95 | 11.11 | 0.11 | 0.02 | 0.21 | -85.65% | -83.80% |
| Iraq | 58.63 | 24.09 | 101.87 | 0.83 | 0.26 | 1.57 | 14.22 | 5.94 | 24.85 | 0.22 | 0.06 | 0.42 | -75.75% | -73.28% |
| Israel | 7.35 | 3.32 | 11.90 | 0.12 | 0.02 | 0.21 | 9.22 | 4.15 | 14.33 | 0.33 | 0.05 | 0.60 | +25.35% | +166.04% |
| Japan | 18.17 | 6.78 | 30.62 | 0.20 | 0.04 | 0.34 | 21.96 | 6.54 | 37.98 | 0.18 | 0.03 | 0.32 | +20.88% | -9.11% |
| Jordan | 16.18 | 6.94 | 26.95 | 0.23 | 0.07 | 0.41 | 6.01 | 2.82 | 9.90 | 0.08 | 0.02 | 0.16 | -62.86% | -64.06% |
| Kazakhstan | 68.30 | 28.32 | 122.65 | 0.79 | 0.30 | 1.41 | 3.13 | 1.43 | 5.12 | 0.03 | 0.01 | 0.05 | -95.41% | -96.44% |
| Kuwait | 9.18 | 4.70 | 15.26 | 0.10 | 0.03 | 0.16 | 4.27 | 1.40 | 7.61 | 0.03 | 0.01 | 0.05 | -53.55% | -70.86% |
| Kyrgyzstan | 88.57 | 37.90 | 157.12 | 1.05 | 0.41 | 1.85 | 7.56 | 3.19 | 12.84 | 0.08 | 0.03 | 0.14 | -91.46% | -92.29% |
| Lao People's Democratic Republic | 684.18 | 198.34 | 1,267.68 | 15.17 | 3.38 | 31.96 | 59.29 | 20.94 | 102.94 | 1.57 | 0.34 | 3.09 | -91.33% | -89.64% |
| Lebanon | 24.76 | 10.29 | 42.45 | 0.47 | 0.13 | 0.90 | 11.96 | 5.52 | 19.76 | 0.21 | 0.05 | 0.39 | -51.70% | -55.82% |
| Malaysia | 39.65 | 12.78 | 71.35 | 1.16 | 0.20 | 2.37 | 25.58 | 11.22 | 41.90 | 0.61 | 0.10 | 1.28 | -35.49% | -47.90% |
| Maldives | 411.64 | 126.46 | 708.00 | 8.73 | 2.19 | 16.35 | 27.16 | 12.37 | 45.46 | 0.53 | 0.10 | 1.00 | -93.40% | -93.88% |
| Mongolia | 47.45 | 17.58 | 94.19 | 0.51 | 0.17 | 1.05 | 4.80 | 1.62 | 11.54 | 0.05 | 0.02 | 0.13 | -89.89% | -89.91% |
| Myanmar | 673.53 | 181.80 | 1,367.12 | 13.85 | 2.88 | 29.79 | 62.89 | 22.45 | 109.44 | 1.58 | 0.34 | 2.97 | -90.66% | -88.61% |
| Nepal | 436.69 | 130.82 | 736.42 | 12.35 | 2.38 | 23.33 | 48.99 | 13.01 | 97.52 | 1.95 | 0.28 | 4.64 | -88.78% | -84.18% |
| Oman | 25.74 | 9.62 | 50.96 | 0.48 | 0.11 | 0.95 | 8.02 | 3.74 | 13.44 | 0.14 | 0.03 | 0.28 | -68.86% | -69.69% |
| Pakistan | 573.84 | 173.75 | 1,042.38 | 15.87 | 2.93 | 35.10 | 122.15 | 36.12 | 226.22 | 3.90 | 0.59 | 8.24 | -78.71% | -75.40% |
| Palestine | 32.08 | 12.65 | 60.68 | 0.51 | 0.15 | 0.97 | 4.61 | 2.11 | 7.77 | 0.07 | 0.02 | 0.13 | -85.64% | -86.69% |
| Philippines | 275.17 | 84.99 | 483.31 | 5.38 | 1.14 | 10.65 | 50.57 | 16.82 | 84.51 | 1.19 | 0.24 | 2.30 | -81.62% | -77.81% |
| Qatar | 8.18 | 3.77 | 13.80 | 0.16 | 0.04 | 0.31 | 4.83 | 2.24 | 7.86 | 0.08 | 0.01 | 0.16 | -41.00% | -48.19% |
| Republic of Korea | 5.95 | 1.96 | 10.39 | 0.24 | 0.04 | 0.51 | 2.64 | 0.99 | 4.86 | 0.11 | 0.02 | 0.24 | -55.68% | -55.94% |
| Saudi Arabia | 51.47 | 18.68 | 95.82 | 1.01 | 0.26 | 1.98 | 11.45 | 4.95 | 19.32 | 0.29 | 0.05 | 0.57 | -77.75% | -71.59% |
| Singapore | 5.50 | 1.54 | 8.65 | 0.21 | 0.04 | 0.36 | 2.06 | 0.59 | 3.30 | 0.10 | 0.02 | 0.18 | -62.59% | -53.99% |
| Sri Lanka | 135.83 | 35.25 | 238.73 | 4.50 | 0.76 | 8.58 | 19.45 | 8.38 | 33.98 | 0.50 | 0.07 | 1.04 | -85.68% | -88.87% |
| Syrian Arab Republic | 31.89 | 12.78 | 57.13 | 0.44 | 0.13 | 0.84 | 7.85 | 3.11 | 13.49 | 0.11 | 0.02 | 0.22 | -75.39% | -75.15% |
| Taiwan (Province of China) | 10.57 | 4.89 | 16.23 | 0.18 | 0.03 | 0.29 | 9.30 | 2.52 | 16.29 | 0.03 | 0.01 | 0.06 | -12.04% | -81.24% |
| Tajikistan | 218.93 | 81.24 | 399.26 | 2.51 | 0.90 | 4.55 | 59.14 | 22.43 | 110.61 | 0.68 | 0.24 | 1.28 | -72.99% | -72.77% |
| Thailand | 196.70 | 46.50 | 530.91 | 6.76 | 0.86 | 22.78 | 40.90 | 15.16 | 66.59 | 1.15 | 0.19 | 2.20 | -79.21% | -82.92% |
| Timor-Leste | 723.13 | 202.51 | 1,373.94 | 15.67 | 3.17 | 35.81 | 75.68 | 24.96 | 131.09 | 1.95 | 0.36 | 3.97 | -89.53% | -87.53% |
| Turkey | 57.75 | 22.74 | 104.87 | 0.73 | 0.24 | 1.33 | 7.88 | 3.64 | 13.54 | 0.11 | 0.02 | 0.21 | -86.36% | -85.28% |
| Turkmenistan | 218.93 | 78.63 | 397.39 | 2.50 | 0.87 | 4.54 | 8.87 | 3.55 | 15.11 | 0.10 | 0.03 | 0.17 | -95.95% | -96.04% |
| United Arab Emirates | 15.19 | 6.17 | 25.84 | 0.30 | 0.06 | 0.58 | 7.78 | 3.56 | 12.42 | 0.16 | 0.03 | 0.32 | -48.78% | -46.91% |
| Uzbekistan | 116.77 | 45.15 | 197.20 | 1.34 | 0.47 | 2.25 | 3.41 | 1.45 | 5.51 | 0.04 | 0.01 | 0.07 | -97.08% | -97.06% |
| Viet Nam | 88.59 | 30.70 | 167.46 | 2.61 | 0.43 | 6.33 | 17.64 | 6.68 | 30.32 | 0.30 | 0.05 | 0.71 | -80.09% | -88.57% |
| Yemen | 363.99 | 128.87 | 683.41 | 5.60 | 1.65 | 10.19 | 30.53 | 10.82 | 63.96 | 0.57 | 0.13 | 1.26 | -91.61% | -89.79% |
| ***Rotavirus*** |  |  |  |  |  |  |  |  |  |  |  |  |  |  |
| Asia | 911.86 | 702.56 | 1,150.42 | 13.01 | 10.06 | 16.73 | 100.47 | 75.28 | 139.55 | 1.55 | 1.12 | 2.25 | -88.98% | -88.07% |
| Afghanistan | 1,223.93 | 734.16 | 1,796.26 | 13.74 | 8.24 | 20.14 | 182.73 | 116.13 | 293.02 | 2.01 | 1.25 | 3.25 | -85.07% | -85.37% |
| Armenia | 421.11 | 317.00 | 544.01 | 4.61 | 3.48 | 5.98 | 7.74 | 5.59 | 10.52 | 0.07 | 0.05 | 0.10 | -98.16% | -98.52% |
| Azerbaijan | 402.70 | 295.38 | 531.54 | 4.46 | 3.26 | 5.92 | 61.71 | 38.71 | 96.72 | 0.68 | 0.42 | 1.07 | -84.68% | -84.87% |
| Bahrain | 51.30 | 38.29 | 69.70 | 0.56 | 0.41 | 0.77 | 9.54 | 6.88 | 13.10 | 0.09 | 0.06 | 0.14 | -81.41% | -83.99% |
| Bangladesh | 969.77 | 703.96 | 1,371.47 | 17.58 | 12.02 | 24.28 | 147.37 | 93.19 | 248.78 | 3.42 | 1.82 | 6.98 | -84.80% | -80.52% |
| Bhutan | 2,103.65 | 888.81 | 3,589.57 | 32.68 | 14.29 | 54.60 | 174.55 | 93.94 | 303.03 | 3.30 | 1.69 | 6.06 | -91.70% | -89.89% |
| Brunei Darussalam | 8.90 | 6.51 | 11.95 | 0.12 | 0.08 | 0.17 | 7.60 | 5.35 | 10.44 | 0.10 | 0.07 | 0.14 | -14.68% | -13.40% |
| Cambodia | 1,050.09 | 711.45 | 1,459.60 | 13.58 | 9.08 | 18.64 | 76.61 | 52.47 | 107.19 | 1.05 | 0.68 | 1.47 | -92.70% | -92.25% |
| China | 321.46 | 234.13 | 421.03 | 3.64 | 2.66 | 4.75 | 7.46 | 5.58 | 10.09 | 0.08 | 0.06 | 0.11 | -97.68% | -97.84% |
| Cyprus | 29.08 | 18.36 | 40.19 | 0.35 | 0.21 | 0.53 | 15.43 | 10.72 | 22.60 | 0.13 | 0.08 | 0.19 | -46.93% | -62.76% |
| Democratic People's Republic of Korea | 35.16 | 23.55 | 52.11 | 0.33 | 0.20 | 0.52 | 20.96 | 14.35 | 29.93 | 0.08 | 0.04 | 0.12 | -40.39% | -76.47% |
| Georgia | 141.62 | 107.28 | 189.07 | 1.53 | 1.14 | 2.05 | 3.35 | 2.42 | 4.60 | 0.02 | 0.01 | 0.03 | -97.64% | -98.72% |
| India | 1,505.41 | 1,142.44 | 1,989.60 | 27.30 | 19.92 | 37.56 | 161.57 | 107.26 | 228.75 | 3.50 | 2.17 | 5.47 | -89.27% | -87.18% |
| Indonesia | 2,364.09 | 1,646.26 | 3,133.03 | 31.92 | 21.49 | 42.97 | 214.32 | 158.67 | 289.14 | 3.30 | 2.22 | 4.41 | -90.93% | -89.65% |
| Iran (Islamic Republic of) | 266.56 | 174.19 | 434.56 | 2.77 | 1.73 | 4.64 | 20.26 | 14.90 | 27.94 | 0.15 | 0.11 | 0.22 | -92.40% | -94.43% |
| Iraq | 203.05 | 138.40 | 298.81 | 2.21 | 1.49 | 3.20 | 30.26 | 21.47 | 41.73 | 0.31 | 0.21 | 0.45 | -85.10% | -86.12% |
| Israel | 27.27 | 20.30 | 37.73 | 0.18 | 0.15 | 0.21 | 16.73 | 12.96 | 23.21 | 0.22 | 0.18 | 0.28 | -38.65% | 25.12% |
| Japan | 10.41 | 7.66 | 14.84 | 0.06 | 0.05 | 0.07 | 11.04 | 7.87 | 16.20 | 0.04 | 0.03 | 0.05 | +6.04% | -23.71% |
| Jordan | 75.07 | 52.20 | 104.97 | 0.76 | 0.51 | 1.07 | 14.88 | 10.44 | 20.73 | 0.12 | 0.08 | 0.18 | -80.18% | -84.07% |
| Kazakhstan | 281.46 | 218.12 | 351.43 | 3.11 | 2.40 | 3.89 | 6.60 | 4.85 | 8.87 | 0.06 | 0.04 | 0.08 | -97.66% | -98.16% |
| Kuwait | 28.77 | 22.51 | 36.88 | 0.23 | 0.18 | 0.29 | 8.54 | 5.66 | 12.10 | 0.03 | 0.03 | 0.04 | -70.33% | -85.76% |
| Kyrgyzstan | 301.80 | 241.57 | 372.46 | 3.35 | 2.68 | 4.15 | 12.63 | 9.62 | 16.21 | 0.13 | 0.10 | 0.17 | -95.82% | -96.04% |
| Lao People's Democratic Republic | 2,742.91 | 1,718.29 | 3,913.68 | 34.82 | 22.06 | 49.79 | 208.40 | 132.86 | 322.37 | 2.70 | 1.74 | 4.10 | -92.40% | -92.23% |
| Lebanon | 92.60 | 53.59 | 139.89 | 1.02 | 0.57 | 1.57 | 33.59 | 23.06 | 47.08 | 0.30 | 0.20 | 0.45 | -63.73% | -70.15% |
| Malaysia | 81.91 | 57.90 | 110.88 | 1.09 | 0.78 | 1.56 | 38.25 | 27.42 | 53.01 | 0.36 | 0.22 | 0.50 | -53.31% | -67.23% |
| Maldives | 1,438.41 | 949.63 | 1,951.77 | 17.68 | 12.37 | 23.96 | 72.57 | 52.44 | 99.85 | 0.73 | 0.52 | 1.07 | -94.95% | -95.85% |
| Mongolia | 188.43 | 109.42 | 302.93 | 2.06 | 1.18 | 3.33 | 18.76 | 7.52 | 37.54 | 0.20 | 0.08 | 0.42 | -90.04% | -90.05% |
| Myanmar | 3,236.00 | 1,636.67 | 5,256.02 | 39.81 | 21.00 | 64.74 | 209.46 | 148.17 | 290.80 | 2.69 | 1.86 | 3.78 | -93.53% | -93.24% |
| Nepal | 1,431.13 | 1,037.14 | 1,979.23 | 21.13 | 15.22 | 29.02 | 87.76 | 57.87 | 125.84 | 1.87 | 1.11 | 3.12 | -93.87% | -91.13% |
| Oman | 98.91 | 57.18 | 170.93 | 1.12 | 0.63 | 1.92 | 23.36 | 16.16 | 33.87 | 0.24 | 0.16 | 0.36 | -76.38% | -78.78% |
| Pakistan | 1,180.60 | 882.03 | 1,533.46 | 16.05 | 11.58 | 21.54 | 146.50 | 97.94 | 222.19 | 2.29 | 1.48 | 3.43 | -87.59% | -85.71% |
| Palestine | 127.42 | 84.25 | 202.82 | 1.41 | 0.93 | 2.26 | 8.87 | 6.23 | 12.75 | 0.08 | 0.05 | 0.11 | -93.04% | -94.69% |
| Philippines | 536.44 | 366.86 | 735.54 | 6.23 | 4.28 | 8.52 | 78.44 | 56.08 | 105.98 | 0.92 | 0.65 | 1.25 | -85.38% | -85.27% |
| Qatar | 24.63 | 17.30 | 35.50 | 0.25 | 0.16 | 0.37 | 6.25 | 4.19 | 8.97 | 0.04 | 0.03 | 0.07 | -74.61% | -82.31% |
| Republic of Korea | 21.34 | 15.75 | 28.28 | 0.33 | 0.21 | 0.45 | 7.88 | 5.65 | 10.62 | 0.12 | 0.07 | 0.19 | -63.07% | -63.82% |
| Saudi Arabia | 148.69 | 94.00 | 234.42 | 1.70 | 1.07 | 2.65 | 13.03 | 9.17 | 18.17 | 0.13 | 0.08 | 0.21 | -91.23% | -92.15% |
| Singapore | 13.40 | 11.18 | 16.03 | 0.21 | 0.18 | 0.24 | 3.76 | 3.00 | 4.73 | 0.07 | 0.05 | 0.08 | -71.91% | -68.10% |
| Sri Lanka | 291.55 | 211.40 | 392.81 | 4.28 | 2.98 | 6.08 | 34.47 | 24.26 | 48.70 | 0.36 | 0.22 | 0.59 | -88.18% | -91.55% |
| Syrian Arab Republic | 122.92 | 84.37 | 186.13 | 1.29 | 0.86 | 1.98 | 17.78 | 12.16 | 24.65 | 0.13 | 0.08 | 0.20 | -85.53% | -89.70% |
| Taiwan (Province of China) | 33.77 | 28.57 | 40.79 | 0.36 | 0.30 | 0.41 | 19.91 | 13.89 | 30.03 | 0.05 | 0.04 | 0.06 | -41.04% | -86.26% |
| Tajikistan | 1,606.25 | 1,230.71 | 2,089.17 | 17.85 | 13.61 | 23.20 | 281.85 | 178.23 | 408.57 | 3.13 | 1.97 | 4.56 | -82.45% | -82.49% |
| Thailand | 308.20 | 134.02 | 574.67 | 4.99 | 1.86 | 11.53 | 52.62 | 39.61 | 69.93 | 0.76 | 0.51 | 1.08 | -82.93% | -84.73% |
| Timor-Leste | 2,672.39 | 1,487.28 | 3,903.28 | 32.82 | 18.98 | 48.51 | 171.49 | 109.35 | 256.42 | 2.22 | 1.40 | 3.43 | -93.58% | -93.24% |
| Turkey | 215.59 | 147.28 | 327.97 | 2.34 | 1.57 | 3.58 | 19.04 | 13.77 | 25.50 | 0.17 | 0.11 | 0.24 | -91.17% | -92.81% |
| Turkmenistan | 786.61 | 618.72 | 987.62 | 8.75 | 6.87 | 10.99 | 24.57 | 17.17 | 34.43 | 0.26 | 0.18 | 0.37 | -96.88% | -96.99% |
| United Arab Emirates | 43.07 | 28.53 | 61.21 | 0.46 | 0.31 | 0.65 | 9.64 | 6.96 | 13.25 | 0.09 | 0.06 | 0.14 | -77.61% | -79.58% |
| Uzbekistan | 385.28 | 303.91 | 492.32 | 4.27 | 3.36 | 5.46 | 5.20 | 3.88 | 7.00 | 0.05 | 0.04 | 0.07 | -98.65% | -98.74% |
| Viet Nam | 209.38 | 118.10 | 320.83 | 2.64 | 1.38 | 4.05 | 22.55 | 15.22 | 33.03 | 0.14 | 0.08 | 0.23 | -89.23% | -94.53% |
| Yemen | 1,561.22 | 998.97 | 2,385.78 | 17.82 | 11.41 | 26.95 | 78.57 | 33.39 | 153.50 | 0.89 | 0.34 | 1.73 | -94.97% | -95.01% |
| ***Shigella*** |  |  |  |  |  |  |  |  |  |  |  |  |  |  |
| Asia | 381.51 | 235.34 | 598.03 | 5.26 | 3.46 | 8.03 | 57.54 | 35.62 | 94.39 | 0.80 | 0.48 | 1.26 | -84.92% | -84.88% |
| Afghanistan | 311.32 | 154.14 | 570.69 | 3.52 | 1.75 | 6.41 | 57.50 | 28.86 | 102.71 | 0.63 | 0.31 | 1.14 | -81.53% | -81.99% |
| Armenia | 95.07 | 55.83 | 163.24 | 1.03 | 0.60 | 1.78 | 2.80 | 1.73 | 4.61 | 0.02 | 0.01 | 0.04 | -97.06% | -97.63% |
| Azerbaijan | 166.48 | 93.65 | 316.42 | 1.84 | 1.03 | 3.50 | 26.20 | 13.41 | 49.89 | 0.28 | 0.14 | 0.54 | -84.26% | -84.59% |
| Bahrain | 13.83 | 8.59 | 22.65 | 0.16 | 0.10 | 0.26 | 4.29 | 2.71 | 6.73 | 0.04 | 0.02 | 0.07 | -69.02% | -72.96% |
| Bangladesh | 431.16 | 271.28 | 731.18 | 6.68 | 4.20 | 11.09 | 59.18 | 34.71 | 102.54 | 1.09 | 0.56 | 2.28 | -86.27% | -83.68% |
| Bhutan | 1,101.92 | 420.00 | 2,110.90 | 16.06 | 6.44 | 30.88 | 92.91 | 43.65 | 188.26 | 1.58 | 0.73 | 3.20 | -91.57% | -90.17% |
| Brunei Darussalam | 0.38 | 0.23 | 0.63 | 0.00 | 0.00 | 0.01 | 0.31 | 0.18 | 0.53 | 0.00 | 0.00 | 0.01 | -17.85% | -15.11% |
| Cambodia | 332.44 | 192.27 | 585.07 | 4.24 | 2.54 | 7.20 | 24.41 | 14.15 | 41.43 | 0.33 | 0.18 | 0.55 | -92.66% | -92.31% |
| China | 65.40 | 37.35 | 112.41 | 0.72 | 0.41 | 1.25 | 1.86 | 1.13 | 3.07 | 0.02 | 0.01 | 0.03 | -97.16% | -97.65% |
| Cyprus | 3.81 | 2.29 | 6.40 | 0.05 | 0.03 | 0.08 | 1.93 | 1.17 | 3.22 | 0.02 | 0.01 | 0.03 | -49.33% | -63.19% |
| Democratic People's Republic of Korea | 3.68 | 2.11 | 6.31 | 0.03 | 0.02 | 0.06 | 3.40 | 1.89 | 5.65 | 0.01 | 0.00 | 0.02 | -7.76% | -69.31% |
| Georgia | 47.72 | 28.45 | 82.72 | 0.51 | 0.29 | 0.88 | 1.98 | 1.19 | 3.25 | 0.01 | 0.01 | 0.02 | -95.84% | -97.71% |
| India | 777.80 | 484.51 | 1,213.12 | 12.67 | 8.22 | 19.21 | 107.05 | 63.59 | 171.75 | 1.92 | 1.12 | 3.22 | -86.24% | -84.86% |
| Indonesia | 547.29 | 306.25 | 915.43 | 7.19 | 4.13 | 11.74 | 51.97 | 30.96 | 86.23 | 0.76 | 0.43 | 1.26 | -90.51% | -89.50% |
| Iran (Islamic Republic of) | 97.20 | 54.48 | 185.95 | 1.01 | 0.54 | 1.99 | 10.59 | 6.59 | 16.49 | 0.08 | 0.05 | 0.13 | -89.11% | -92.35% |
| Iraq | 62.76 | 32.81 | 115.85 | 0.69 | 0.37 | 1.28 | 13.21 | 7.89 | 22.76 | 0.14 | 0.08 | 0.23 | -78.95% | -80.48% |
| Israel | 2.88 | 1.76 | 4.70 | 0.02 | 0.01 | 0.03 | 2.21 | 1.44 | 3.58 | 0.03 | 0.02 | 0.04 | -23.35% | 24.88% |
| Japan | 0.80 | 0.47 | 1.36 | 0.00 | 0.00 | 0.01 | 0.92 | 0.51 | 1.59 | 0.00 | 0.00 | 0.00 | +14.22% | -22.23% |
| Jordan | 11.91 | 6.73 | 19.96 | 0.12 | 0.07 | 0.21 | 3.88 | 2.42 | 6.26 | 0.03 | 0.02 | 0.05 | -67.44% | -73.22% |
| Kazakhstan | 91.37 | 54.10 | 157.10 | 1.00 | 0.59 | 1.72 | 2.41 | 1.46 | 4.06 | 0.02 | 0.01 | 0.03 | -97.36% | -98.07% |
| Kuwait | 8.14 | 5.07 | 13.37 | 0.06 | 0.04 | 0.10 | 3.35 | 1.90 | 5.53 | 0.01 | 0.01 | 0.02 | -58.83% | -80.09% |
| Kyrgyzstan | 120.96 | 71.38 | 207.02 | 1.34 | 0.79 | 2.29 | 8.27 | 4.97 | 13.92 | 0.09 | 0.05 | 0.15 | -93.17% | -93.55% |
| Lao People's Democratic Republic | 730.34 | 378.08 | 1,352.80 | 8.90 | 4.93 | 15.87 | 51.55 | 27.92 | 88.63 | 0.63 | 0.34 | 1.06 | -92.94% | -92.87% |
| Lebanon | 24.47 | 13.27 | 42.25 | 0.28 | 0.15 | 0.48 | 9.97 | 5.88 | 16.54 | 0.09 | 0.05 | 0.15 | -59.27% | -67.98% |
| Malaysia | 17.49 | 10.46 | 27.62 | 0.23 | 0.13 | 0.36 | 8.19 | 5.01 | 13.30 | 0.07 | 0.04 | 0.12 | -53.19% | -67.49% |
| Maldives | 277.48 | 154.03 | 476.80 | 3.36 | 1.89 | 5.74 | 13.84 | 8.35 | 22.94 | 0.14 | 0.08 | 0.23 | -95.01% | -95.90% |
| Mongolia | 61.76 | 30.02 | 121.05 | 0.67 | 0.32 | 1.33 | 6.14 | 2.32 | 14.78 | 0.07 | 0.02 | 0.16 | -90.06% | -90.07% |
| Myanmar | 657.85 | 293.73 | 1,315.46 | 7.94 | 3.77 | 15.68 | 47.42 | 26.75 | 84.82 | 0.58 | 0.33 | 1.00 | -92.79% | -92.71% |
| Nepal | 916.74 | 546.81 | 1,540.63 | 12.81 | 8.05 | 20.33 | 69.03 | 42.69 | 109.98 | 1.23 | 0.71 | 2.14 | -92.47% | -90.40% |
| Oman | 34.84 | 17.30 | 62.54 | 0.41 | 0.21 | 0.75 | 9.12 | 5.71 | 15.21 | 0.09 | 0.05 | 0.16 | -73.81% | -77.29% |
| Pakistan | 833.98 | 485.79 | 1,372.05 | 11.11 | 6.69 | 17.79 | 130.85 | 76.31 | 225.34 | 1.89 | 1.11 | 3.19 | -84.31% | -82.96% |
| Palestine | 40.69 | 21.22 | 75.25 | 0.46 | 0.24 | 0.85 | 4.69 | 2.91 | 7.69 | 0.04 | 0.02 | 0.07 | -88.48% | -91.08% |
| Philippines | 179.70 | 103.87 | 305.19 | 2.10 | 1.19 | 3.52 | 26.98 | 15.95 | 46.66 | 0.32 | 0.19 | 0.54 | -84.99% | -84.89% |
| Qatar | 6.90 | 4.18 | 11.63 | 0.07 | 0.04 | 0.12 | 3.23 | 2.00 | 5.24 | 0.02 | 0.01 | 0.04 | -53.22% | -65.86% |
| Republic of Korea | 0.46 | 0.28 | 0.78 | 0.01 | 0.00 | 0.01 | 0.17 | 0.11 | 0.28 | 0.00 | 0.00 | 0.00 | -62.80% | -64.78% |
| Saudi Arabia | 56.09 | 29.53 | 102.35 | 0.68 | 0.36 | 1.24 | 8.44 | 5.17 | 13.72 | 0.09 | 0.05 | 0.16 | -84.95% | -86.25% |
| Singapore | 0.63 | 0.39 | 1.04 | 0.01 | 0.01 | 0.02 | 0.19 | 0.12 | 0.31 | 0.00 | 0.00 | 0.01 | -69.45% | -65.64% |
| Sri Lanka | 55.68 | 33.80 | 98.18 | 0.79 | 0.47 | 1.32 | 6.44 | 3.99 | 10.77 | 0.07 | 0.03 | 0.11 | -88.44% | -91.75% |
| Syrian Arab Republic | 32.53 | 18.64 | 59.61 | 0.34 | 0.19 | 0.64 | 5.79 | 3.36 | 9.58 | 0.04 | 0.02 | 0.07 | -82.21% | -87.83% |
| Taiwan (Province of China) | 2.32 | 1.52 | 3.76 | 0.02 | 0.01 | 0.03 | 1.71 | 0.90 | 3.01 | 0.00 | 0.00 | 0.00 | -26.48% | -87.68% |
| Tajikistan | 498.05 | 288.66 | 862.29 | 5.52 | 3.20 | 9.62 | 139.06 | 75.57 | 236.93 | 1.54 | 0.83 | 2.64 | -72.085 | -72.13% |
| Thailand | 46.69 | 20.66 | 95.24 | 0.64 | 0.26 | 1.42 | 7.80 | 4.92 | 12.52 | 0.10 | 0.06 | 0.16 | -83.285 | -84.69% |
| Timor-Leste | 505.87 | 255.45 | 907.01 | 6.15 | 3.17 | 10.85 | 40.66 | 21.50 | 71.68 | 0.50 | 0.27 | 0.84 | -91.96% | -91.89% |
| Turkey | 64.41 | 34.49 | 122.57 | 0.70 | 0.37 | 1.34 | 6.68 | 4.15 | 10.88 | 0.06 | 0.03 | 0.10 | -89.63% | -91.98% |
| Turkmenistan | 271.56 | 160.99 | 453.42 | 3.01 | 1.78 | 5.01 | 9.19 | 5.22 | 15.36 | 0.10 | 0.05 | 0.17 | -96.62% | -96.78% |
| United Arab Emirates | 12.13 | 7.20 | 19.60 | 0.14 | 0.08 | 0.22 | 5.15 | 3.29 | 8.13 | 0.05 | 0.03 | 0.09 | -57.54% | -61.66% |
| Uzbekistan | 88.40 | 52.31 | 147.93 | 0.98 | 0.57 | 1.63 | 1.91 | 1.18 | 3.12 | 0.02 | 0.01 | 0.03 | -97.84% | -98.00% |
| Viet Nam | 32.46 | 16.06 | 63.13 | 0.40 | 0.19 | 0.78 | 4.17 | 2.46 | 6.71 | 0.02 | 0.01 | 0.04 | -87.16% | -93.87% |
| Yemen | 396.37 | 219.80 | 711.54 | 4.64 | 2.58 | 8.18 | 27.91 | 11.67 | 54.83 | 0.32 | 0.13 | 0.65 | -92.96% | -93.03% |

DALY, disability-adjusted life years; ASDR, Age-standardised DALYs rate; ASMR, Age-standardized mortality rate; 95% UI, 95% uncertainty interval.
